# Cationic Group VI Metal Imido Alkylidene *N*‐Heterocyclic Carbene Nitrile Complexes: Bench‐Stable, Functional‐Group‐Tolerant Olefin Metathesis Catalysts

**DOI:** 10.1002/anie.202011666

**Published:** 2020-11-19

**Authors:** Mathis J. Benedikter, Janis V. Musso, Wolfgang Frey, Roman Schowner, Michael R. Buchmeiser

**Affiliations:** ^1^ Institut für Polymerchemie Universität Stuttgart Pfaffenwaldring 55 70569 Stuttgart Germany; ^2^ Institut für Organische Chemie Universität Stuttgart Pfaffenwaldring 55 70569 Stuttgart Germany

**Keywords:** alkylidenes, metathesis, molybdenum, *N*-heterocyclic carbene, tungsten

## Abstract

Despite their excellent selectivities and activities, Mo‐and W‐based catalysts for olefin metathesis have not gained the same widespread use as Ru‐based systems, mainly due to their inherent air sensitivity. Herein, we describe the synthesis of air‐stable cationic‐at‐metal molybdenum and tungsten imido alkylidene NHC nitrile complexes. They catalyze olefin metathesis reactions of substrates containing functional groups such as (thio‐) esters, (thio‐) ethers and alcohols without the need for prior activation, for example, by a Lewis acid. The presence of a nitrile ligand was found to be essential for their stability towards air, while no decrease in activity and productivity could be observed upon coordination of a nitrile. Variations of the imido and anionic ligand revealed that alkoxide complexes with electron‐withdrawing imido ligands offer the highest reactivities and excellent stability compared to analogous triflate and halide complexes.

## Graphical abstract

The presence of a nitrile ligand renders cationic group VI metal imido alkylidene NHC complexes air‐stable, functional‐group‐tolerant, and olefin metathesis‐active. No pre‐activation is required and no loss of activity, productivity, or selectivity in olefin metathesis is observed due to the presence of a nitrile ligand.

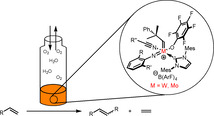

## Introduction

While the first observations on olefin metathesis were already reported in the middle of the 20^th^ century, interest in the topic increased dramatically upon discovery of the first well‐defined (pre‐) catalysts. Currently, well‐defined olefin metathesis catalysts are mainly based on ruthenium‐based Grubbs‐catalysts as well as molybdenum‐ and tungsten‐based Schrock‐type catalysts.

**Scheme 1:**
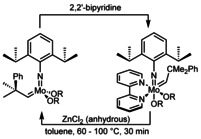
Protection and deprotection of Schrock‐type bisalkoxide catalysts as reported by Fürstner et al.^[6]^

Grubbs‐catalysts have become popular for many organic and polymer chemists, particularly because of their air stability^[1]^ and thus easy handling, as well as their excellent stability towards many functional groups, including protic ones such as alcohols.^[2]^ Schrock catalysts allow for impressive activities, excellent stereo‐ and enantioselectivity^[3]^ and can tolerate a number of different functional groups. However, due to their inherent sensitivity to air and protic functional groups,^[4]^ their use essentially requires the use of a glovebox. Therefore, they yet have not gained the same widespread use as their ruthenium‐based counterparts. Boncella et al. reported on several molybdenum and tungsten alkylidene complexes bearing hydridotris(pyrazolyl)borate ligands, that are tolerant versus air, moisture and heat for a long time. However, these 18‐electron complexes were not active in olefin metathesis and required the addition of AlCl_3_ to result in ROMP (ring‐opening metathesis polymerization) active species. Also, the mechanism of activation and the active species itself could not be identified.^[5]^ Fürstner et al. reacted Schrock‐type bisalkoxide catalysts with 2,2′‐bipyridine and 1,10‐phenanthroline leading to octahedral complexes that are stable in air for several weeks. To regenerate the parent compounds, these complexes require activation, for example, by treatment with anhydrous ZnCl_2_ in toluene at up to 100 °C for 30 minutes (Scheme 1).^[6]^ This offers an elegant solution for the creation of air‐stable Schrock pre‐catalysts, however, due to the hygroscopic nature of ZnCl_2_, this process most likely still requires a glovebox. Also, during activation, an air‐ and moisture sensitive Schrock catalyst is reformed.

**Scheme 2:**
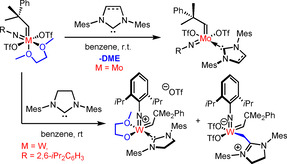
The use of [Mo(*N*R)(CHCMe_2_Ph)(DME)(OTf)_2_] complexes as precursors for the coordination of NHCs and the products of DME activation in the case of the analogous tungsten compounds.

In recent years, molybdenum imido and, to a lesser extent, tungsten oxo/imido alkylidene NHC complexes have been developed by our group, which exhibit outstanding activities and excellent functional group tolerance.^[7]^ Furthermore, excellent stereoselectivities in ring‐opening metathesis polymerization (ROMP) as well as ring‐opening‐cross metathesis (ROCM) could be achieved. Upon introduction of a chelating carboxylate ligand, air‐stable (for at least 5 days) cationic molybdenum imido alkylidene NHC complexes were obtained, however, at the cost of a significantly reduced productivity compared to other, monodentate ligands.^[8]^ In molybdenum chemistry, the universal bistriflate complexes of the general formula [Mo(*N*R)(CHCMe_2_Ph)(DME)(OTf)_2_] (R=*t*Bu, Ad, 2,6‐Me_2_C_6_H_3_, 2,6‐iPr_2_C_6_H_3_, 2,6‐Cl_2_C_6_H_3_, 3,5‐Me_2_C_6_H_3_, 2‐*t*BuC_6_H_4_, 2‐CF_3_C_6_H_4_; DME = 1,2‐dimethoxyethane) are typically used as starting material for the coordination of NHCs.[^7b^, ^7e^] However, these reactions cannot be transferred to tungsten chemistry, because transmethylation of the DME ligand is observed upon addition of the free NHC to the precursor (Scheme 2).^[9]^

For this reason, most of the work reported so far focused on molybdenum‐based complexes. Only a few examples of tungsten imido alkylidene NHC complexes have been reported, all of which contain the 2,6‐diisopropylphenylimido or the 1,3‐diisopropylimidazol‐2‐ylidene (**IiPr**) ligand as the NHC.^[10]^ Since it was previously shown that both, the imido and the NHC ligand have a pronounced influence on the reactivity and selectivity of a catalyst, we were eager to find a way to prepare tungsten imido alkylidene NHC complexes containing tailored imido‐ and NHC ligands.[^8a^, ^8c^]

Herein, we report the routes we discovered for the synthesis of such compounds, as well as their productivity, activity and stability. Most important, we were able to transfer the results found for tungsten to molybdenum to create a set of bench‐, that is, air‐stable, functional‐group‐tolerant Mo‐ and W‐based olefin metathesis catalysts that do not require activation by a Lewis acid.

## Results and Discussion

### Synthesis of Metal Complexes

**Scheme 3:**
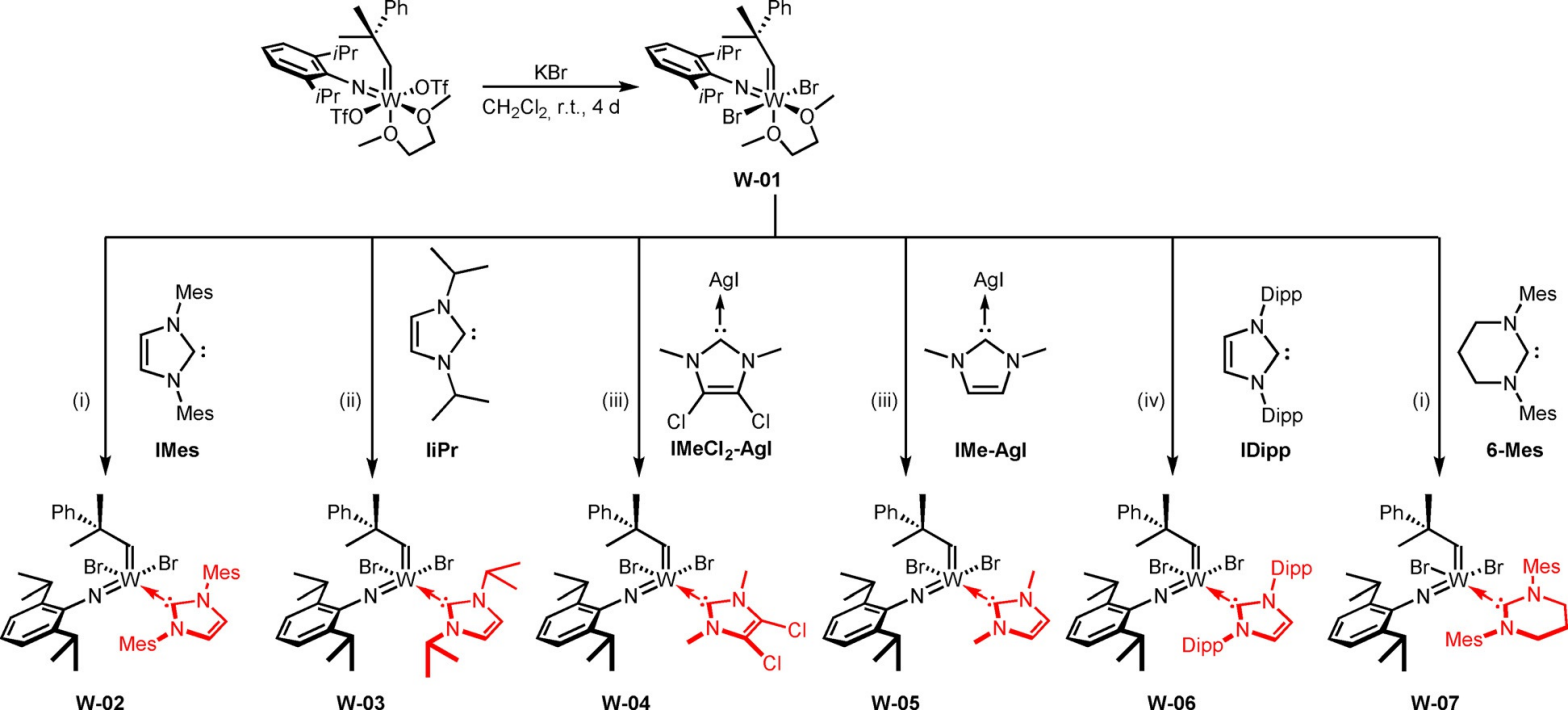
Synthesis of the dibromide complex **W‐01** and coordination of different NHC ligands to the precursor. Reaction conditions: (i) toluene, r.t., 1 h; (ii) Et_2_O, −35 °C to r.t., 1 h; (iii) CH_2_Cl_2_, r.t., 20 min; (iv) benzene, r.t., 15 min.

After unveiling the reaction cascade that commences upon treating tungsten imido alkylidene bistriflate complexes with 1,3‐dimesityl‐4,5‐dihydroimidazol‐2‐ylidene (**IMesH_2_**), which presumably also applies to other NHCs, we assumed that these reactions are only possible because the triflate ligand represents an excellent leaving group.^[9]^ Therefore, the triflate ligands were exchanged for alkoxide and 2,5‐dimethylpyrrolide ligands, respectively, which indeed enabled the coordination of **IiPr**.^[10]^ However, coordination of more sterically demanding NHCs was still not possible, presumably for steric reasons. Therefore, we set out to find other anionic ligands that were sterically less demanding but provided sufficient stability. We already reported that some molybdenum bistriflate complexes [Mo(*N*R)(CHCMe_2_Ph)(DME)(OTf)_2_] can undergo salt metathesis with KBr to yield the corresponding dibromide complexes, which even react with highly basic 1,3‐dimesityl‐3,4,5,6‐tetrahydropyrimidin‐2‐ylidene (**6‐Mes**) to form the desired NHC complex.^[8b]^ Encouraged by these results, we reacted [W(*N*Dipp)(CHCMe_2_Ph)(DME)(OTf)_2_] with KBr in CH_2_Cl_2_, which resulted in the formation of the dibromide complex **W‐01** (Scheme 3). The use of excess KBr as well as finely powdering the reagent were found to speed up the reaction significantly. However, on a larger scale of several grams, reaction times of up to 4 days were sometimes necessary to achieve full conversion due to the heterogeneous nature of the reaction. However, no side‐products were formed and the dibromide complex could be stored at −35 °C under a N_2_‐atmosphere for months. Due to the apparent equivalency of the DME protons in ^1^H‐ and ^13^C‐NMR, an octahedral structure with the bromide ligands *trans* to each other, similar to the corresponding bistriflate complexes, can be assumed. Reaction of the dibromide complex with 1,3‐dimesitylimidazol‐2‐ylidene (**IMes**) in benzene led to displacement of DME by the NHC, yielding the tungsten imido alkylidene NHC dibromide complex **W‐02**. The reaction again proceeded cleanly without formation of any side products.

**Scheme 4:**
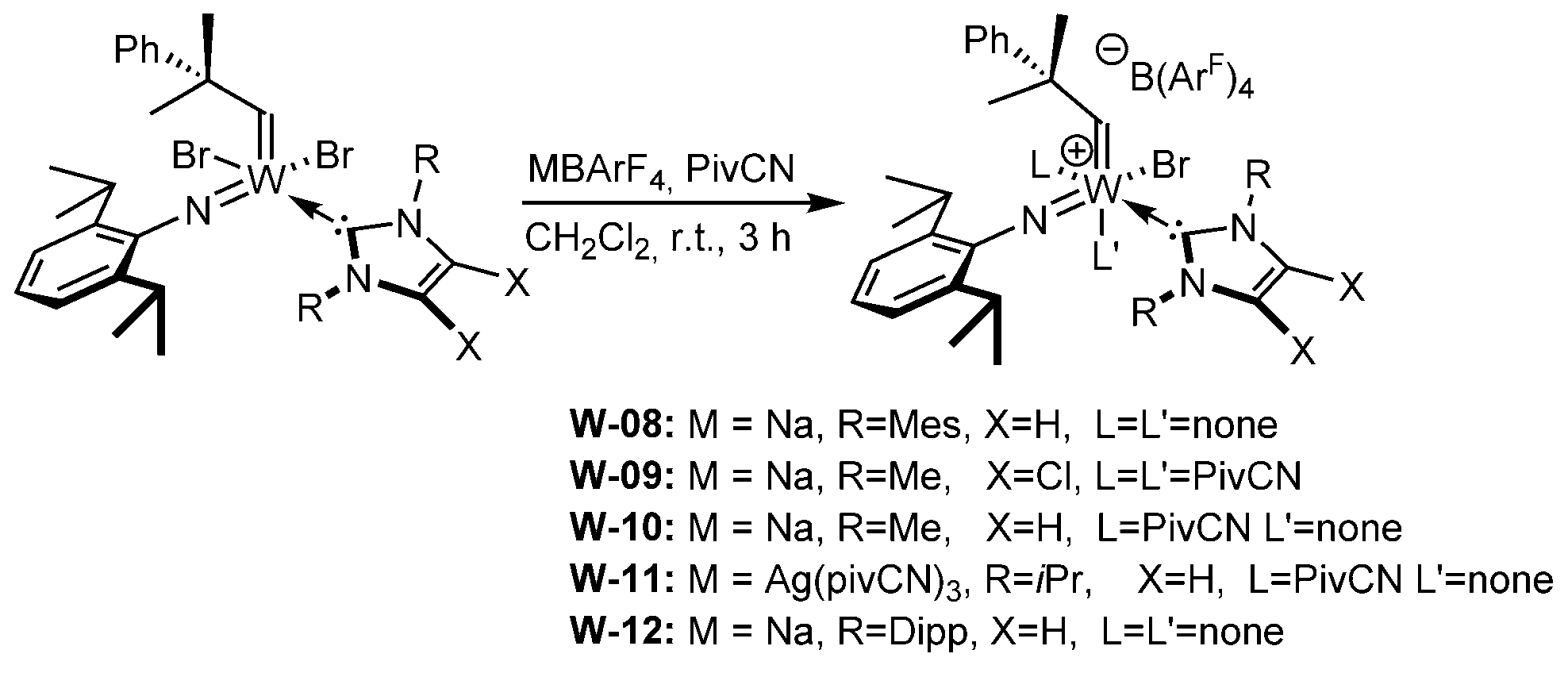
Synthesis of the cationic monobromide complexes **W‐08**–**W‐12** by salt metathesis of the dibromo precursors with NaB(Ar^F^)_4_ or [Ag(PivCN)_3_ B(Ar^F^)_4_].

Encouraged by this observation, we successfully coordinated other NHCs to **W‐01**, namely **IiPr**, the sterically very demanding 1,3‐bis(2,6‐diisopropylphenyl)imidazol‐2‐ylidene (**IDipp**) and the highly basic **6‐Mes** (p*K*_a_ of the corresponding pyrimidinium salt=24.2 in DMSO).^[11]^ 1,3‐Dimethyl‐4,5‐dichloroimidazol‐2‐ylidene (**IMeCl_2_**) and 1,3‐dimethylimidazol‐2‐ylidene (**IMe**) were introduced via the corresponding NHC‐AgI complexes. Since the resulting compounds are all 16‐electron complexes, we did not expect them to be catalytically highly active and thus explored the synthesis of corresponding cationic complexes by introduction of the weakly coordinating anion tetrakis[3,5‐bis(trifluoromethyl)phenyl]borate, (B(Ar^F^)_4_). In the case of **W‐02**, **W‐04**, **W‐05** and **W‐06**, this was achieved by using NaB(Ar^F^)_4_. However, **W‐04** and **W‐05** required the presence of an additional coordinating nitrile ligand such as pivalonitrile (PivCN) to obtain pure, isolable cationic compounds (Scheme 4).

**Scheme 5:**
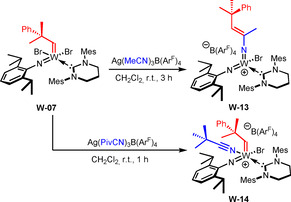
Nitrile metathesis with the alkylidene ligand as well as the synthesis of cationic complex **W‐14**.

Interestingly, in case the reaction of **W‐06** with NaB(Ar^F^)_4_ was conducted in the presence of PivCN, decomposition was observed, likely due to steric overcrowding of the metal center. The attempted reaction of **W‐07** with NaB(Ar^F^)_4_ also led to decomposition of the compound, even in the presence of a nitrile. However, if [Ag(MeCN)_3_ B(Ar^F^)_4_] was employed, the reaction proceeded cleanly, though the product did not exhibit an alkylidene signal in the ^1^H‐NMR. Instead, a new signal with an integral of one appeared at *δ*=5.49 ppm, indicating the presence of an olefinic proton. This can be attributed to nitrile metathesis, that is, the insertion of acetonitrile into the alkylidene‐bond via an azatungstacyclobutene leading to the formation of the mixed bisimido complex **W‐13** (Scheme 5).

**Figure 1:**
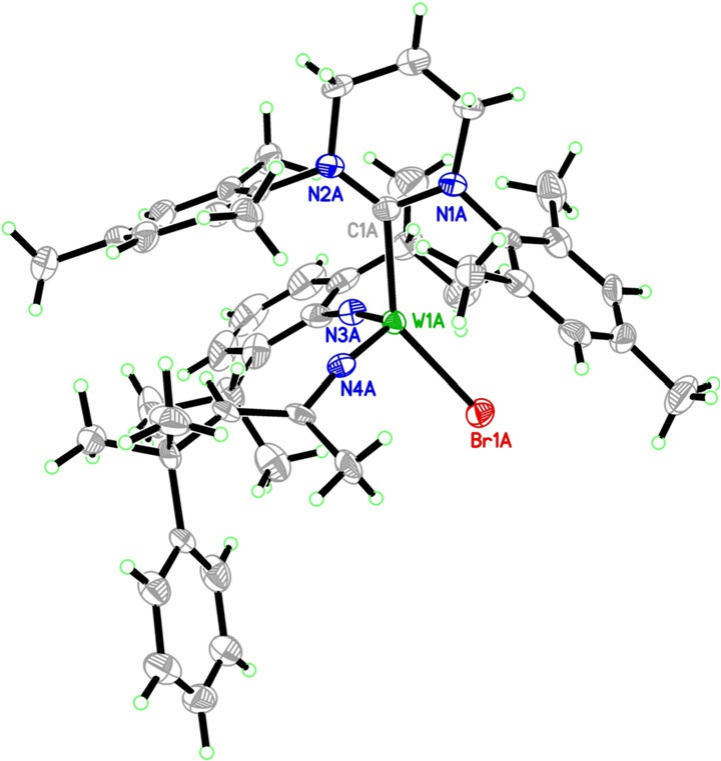
Single crystal X‐ray structure of **W‐13**. Anion omitted for clarity. Selected bond lengths [pm] and angles [°]: W(1A)–N(4A) 176.1(8), W(1A)–N(3A) 176.2(8), W(1A)–C(1A) 220.7(9), W(1A)–Br(1A) 245.94(11), N(4A)‐W(1A)‐N(3A) 114.8(4), N(4A)‐W(1A)‐C(1A) 101.0(3), N(3A)‐W(1A)‐C(1A) 97.3(4), N(4A)‐W(1A)‐Br(1A) 102.2(2), N(3A)‐W(1A)‐Br(1A) 100.2(3), C(1A)‐W(1A)‐Br(1A) 141.5(3).

Similar observations have previously been made for tungsten complexes by Schrock et al.^[12]^ We were able to obtain a single crystal X‐ray structure of the insertion product (Figure 1) that confirms its proposed structure.

Compound **W‐13** crystallizes in the monoclinic space group *P*c with *a*=2757.25(10) pm, *b*=1247.05(4) pm, *c*=2375.91(7) pm; *α*=*γ*=90°, *β*=91.523(2)°. The compound's geometry is distorted tetrahedral. Surprisingly, it does not contain an acetonitrile ligand. Despite the different nature of the imido ligands (aliphatic vs. aromatic), the bond lengths are essentially identical.

**Figure 2:**
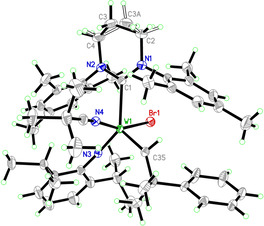
Single crystal X‐ray structure of **W‐14**. Anion omitted for clarity. Selected bond lengths [pm] and angles [°]: W(1)–N(3) 174.42(16), W(1)–C(35) 188.12(18), W(1)–N(4) 216.00(17), W(1)–C(1) 234.65(19), W(1)–Br(1) 251.40(2), N(3)‐W(1)‐C(35) 101.70(8), N(3)‐W(1)‐N(4) 96.97(7), C(35)‐W(1)‐N(4) 90.19(7), N(3)‐W(1)‐C(1) 136.38(7), C(35)‐W(1)‐C(1) 121.63(8), N(4)‐W(1)‐C(1) 79.41(6), N(3)‐W(1)‐Br(1) 99.63(5), C(35)‐W(1)‐Br(1) 94.77(6), N(4)‐W(1)‐Br(1) 161.31(4), C(1)‐W(1)‐Br(1) 82.66(5).

Next, in order to avoid insertion of the nitrile into the W=C bond, the sterically more demanding PivCN was employed. [Ag(PivCN)_3_ B(Ar^F^)_4_] was prepared by simply dissolving [Ag(MeCN)_3_ B(Ar^F^)_4_] in an excess of PivCN followed by removal of all excess nitriles. Reaction of **W‐07** with [Ag(PivCN)_3_ B(Ar^F^)_4_] indeed yielded the expected nitrile‐coordinated cationic alkylidene complex **W‐14** (Scheme 5), the structure of which was also confirmed by single crystal X‐ray diffraction (Figure 2). Compound **W‐14** crystallizes in the triclinic space group $P\bar{1}$ with *a*=1277.49(7) pm, *b*=1728.68(9) pm, *c*=1882.93(10) pm; *α*=85.646(2)°, *β*=87.387(2)°, *γ*=85.634(3)°. The complex adopts a distorted square pyramidal geometry (*τ*_5_=0.416). Notably, [Ag(PivCN)_3_ B(Ar^F^)_4_] can also be used to prepare other cationic monohalide complexes, as was shown in the case of **W‐11** (Scheme 4).

**Scheme 6:**
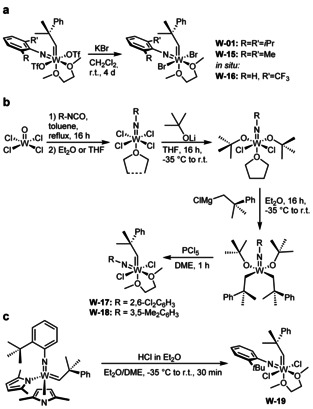
Synthetic routes for the preparation of W imido alkylidene dihalide DME complexes **W‐01** and **W‐15**–**W‐19**.

To determine the influence of the imido ligand on selectivity and reactivity, we attempted to prepare the dibromide complexes from other bistriflates. Reactions with KBr were successful for the 2,6‐dimethylphenylimido and the 2‐trifluoromethylphenylimido ligand, but not for the 2‐*tert*‐butylphenylimido and 2,6‐dichlorophenylimido ligand (Scheme 6 a).

However, the tungsten 2,6‐dichlorophenylimido and 3,5‐dimethylphenylimido alkylidene dichloride complexes were prepared following our recently published alternative synthetic procedure,^[13]^ in which the imido ligand is introduced by reaction WOCl_4_ with an isocyanate (Scheme 6 b).^[14]^

**Scheme 7:**
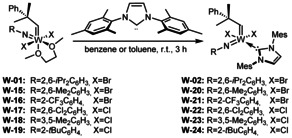
Synthesis of tungsten imido alkylidene IMes dihalide complexes **W‐02** and **W‐20**–**W‐24**.

The tungsten 2‐*tert*‐butylphenylimido alkylidene dichloride complex **W‐19** was obtained by protonation of the corresponding bispyrrolide complex with HCl in diethyl ether in the presence of DME (Scheme 6 c). All tungsten imido alkylidene dihalide DME complexes were then reacted with **IMes** (Scheme 7). In all cases, clean formation of the desired NHC complexes and no DME activation was observed. Notably, the dihalide DME complexes do not necessarily need to be isolated and purified prior to the reaction with **IMes**, as successfully demonstrated for the tungsten 2‐trifluoromethylphenylimido alkylidene IMes dibromide comlex **W‐21**.

**Scheme 8:**
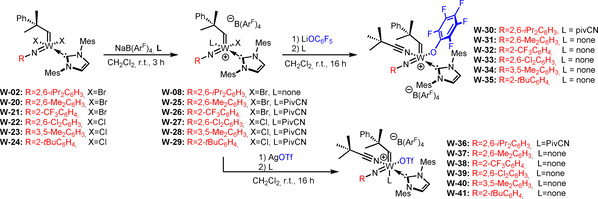
Synthesis of cationic W imido alkylidene monohalide complexes **W‐08** and **W‐25**–**W‐29** and subsequent salt metathesis with AgOTf and LiOC_6_F_5_.

The resulting **IMes** complexes **W‐02** and **W‐20**–**W‐24** were then successfully transformed into their cationic counterparts by salt metathesis with NaB(Ar^F^)_4_ (Scheme 8). Based on our previous findings (vide supra), PivCN was added to all reactions except the one employing **W‐02**. The cationic monohalide complexes **W‐08** and **W‐25**–**W‐29** were reacted with AgOTf to yield the triflate complexes **W‐36**–**W‐41**; reaction with LiOC_6_F_5_ yielded the pentafluorophenoxide complexes **W‐30**–**W‐35** (Scheme 8). In the case of **W‐08**, the addition of pivalonitrile was necessary to obtain crystalline products. The triflate anion was chosen because the related molybdenum complexes have proven highly reactive and tolerant towards alcohol‐containing substrates and, in some cases, air‐stable for several hours.^[7a]^ The pentafluorophenoxide was chosen in view of an earlier observation that cationic tungsten imido alkylidene NHC complexes containing the pentafluorophenoxide ligand by far outperformed all other tungsten compounds in terms of productivity.^[10]^ Furthermore, alkoxide ligands allow for the immobilization of catalysts on silica, analogously to what has been published for molybdenum.^[15]^

### Reactivity

**Figure 3:**
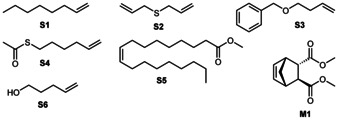
Substrates employed in benchmark metathesis reactions employing cationic tungsten imido alkylidene NHC complexes.

All cationic complexes were employed in a set of benchmark olefin metathesis reactions, typically ring‐closing metathesis (RCM) or homometathesis (HM), using substrates containing ether, ester, thioester, thioether, and alcohol groups. Substrates are displayed in Figure 3. The productivities of the catalysts expressed in turnover numbers (TONs) are listed in Table 1.

**Table 1:** TONs for benchmark metathesis reactions of **W‐08** and **W‐25**–**W‐41**. *E*‐content [%] of the product is given in parentheses.

	**S1**	**S2**	**S3**	**S4**	**S5**	**S6**
**Cationic tungsten imido alkylidene NHC halide complexes**						
**W‐08**	1190 (88)	0	0	0	0	0
**W‐25**	1320 (82)	160	110	390	0	0
**W‐26**	1060 (89)	740	0	380	0	910
**W‐27**	1140 (91)	300	100	710	0	0
**W‐28**	1170 (90)	60	0	110	0	450
**W‐29**	1320 (91)	170	0	400	0	0
**Cationic tungsten imido alkylidene NHC triflate complexes**						
**W‐36**	660 (68)	0	0	110	0	220
**W‐37**	1120 (87)	70	0	0	0	730
**W‐38**	1060 (90)	210	260	260	0	1400
**W‐39**	1140 (90)	120	210	180	0	760
**W‐40**	420 (64)	50	110	0	0	1340
**W‐41**	1120 (89)	100	0	0	0	600
**Cationic tungsten imido alkylidene NHC pentafluorophenoxide complexes**						
**W‐30**	1300 (91)	1500	880	270	0	300
**W‐31**	1210 (90)	400	1310	800	250	560
**W‐32**	1150 (91)	1240	870	1130	360	990
**W‐33**	1240 (90)	810	1090	1070	50	880
**W‐34**	1270 (90)	80	0	380	70	200
**W‐35**	1230 (91)	320	380	930	0	460

Reaction conditions: catalyst:substrate=1:2500, room temperature, 3 h, 1,2‐dichloroethane, 0.5 M in substrate, internal standard: dodecane, conversion determined via GC‐MS.

The productivities of the monohalide complexes containing the 2,6‐diisopropylphenylimido ligand (**W‐08**–**W‐12**, **W14**) are summarized in Table S1 (Supporting Information). Generally, these complexes show rather low productivities. A possible explanation could be, that the bromide ligand does not withdraw enough electron density from the metal center. 1‐Octene was the only substrate that was metathesized by all complexes, with complex **W‐08** displaying the highest productivity. **W‐14** is the only complex that displays notable activity in the metathesis of other substrates such as diallyl sulfide, methyl oleate and pent‐4‐en‐1‐ol. Gratifyingly, variation of the imido ligand as well as exchange of the halide ligand for a more electron‐withdrawing ligand increased productivities (Table 1). Productivities for the other halide complexes **W‐25**–**W‐29** were still moderate, but in all cases exceeded those of the previously examined complex **W‐08** bearing the 2,6‐diisopropylphenylimido ligand.

Most halide compounds showed no activity in the metathesis of ethers, esters and alcohols. The corresponding triflate complexes behaved similarly, however, significantly increased TONs for the metathesis of alcohols were observed, likely due to the more electron‐withdrawing nature of the triflate ligand. In line with our concept, complexes containing the pentafluorophenoxide ligand were found to be the most active ones, as judged by the significantly higher TONs for allyl sulfide and the ether containing substrate **S3**, as well as activity in the metathesis of methyl oleate. The productivity in the metathesis of pent‐4‐en‐1‐ol was similar or slightly lower than for the triflate complexes, this is in line with what has been observed for the analogous molybdenum complexes.^[7a]^ Overall, tungsten complexes based on electron‐withdrawing imido ligands, that is, 2,6‐dichlorophenylimido and 2‐trifluoromethylphenylimido offered the highest productivities, likely due to the more electrophilic metal centers.

### Air Stability

Encouraged by the air stability of cationic molybdenum imido alkylidene NHC complexes,^[7a]^ we set out to explore the stability of their tungsten analogs. For this purpose, a sample of the solid complex was exposed to air for up to two weeks. The sample was then dissolved in dry deuterated solvent and the stability was assessed by ^1^H‐ and ^19^F‐NMR. After 16 hours, almost none of the complexes showed signs of decomposition. The most notable exception was **W‐08**, which decomposed completely, forming one equivalent of imidazolium salt, with B(Ar^F^)_4_ acting as the anion,^[16]^ and further, unknown side products. This is surprising for two reasons: first, all analogous cationic halide complexes containing the **IMes** ligand (**W‐25** to **W‐29**) showed no or only very minor signs of decomposition after storage in air for two weeks (longer periods were not tested); second, **W‐12** did not decompose either, even though all ligands except the NHC are identical to that of **W‐08**. This indicates that air stability is most likely dependent on the steric environment of the metal center. Thus, the **IMes** complexes **W‐25** to **W‐29** are sterically protected by the additional nitrile ligand and coordinatively saturated, while **W‐12** bears the sterically significantly more demanding **IDipp**. This poses the question, whether addition of a nitrile ligand to four‐coordinate cationic complexes could potentially present a general strategy to obtain air‐stable olefin metathesis (pre‐) catalysts and, if yes, would this increased stability be realized at the expense of activity, productivity or stereoselectivity. Indeed, the pentafluorophenoxide complexes, all of which are pentacoordinate and contain a nitrile ligand, showed no (**W‐31**, **W‐32**, **W‐33**, **W‐35**) or very minor (**W‐30** and **W‐34**) signs of decomposition after exposure to air overnight. After two weeks in air, **W‐30** and **W‐34** were 80 % and completely decomposed, respectively. The other alkoxide complexes **W‐31**, **W‐32**, **W‐33**, **W‐35**, however, were at least 95 % intact. This illustrates the importance of exploring different imido ligands. In the case of **W‐34**, this significantly reduced stability might be explained by an insufficient steric shielding of the metal center due to the sterically less demanding imido ligand. The instability of **W‐30** compared to **W‐31**, however, appears very counterintuitive and cannot be explained with the available data. The triflate complexes were found to be less stable than the halide and alkoxide compounds. Thus, **W‐36** exhibited minor signs of decomposition and changes in chemical shift after exposure to air overnight, both of which can be explained by uptake and coordination of water. After two weeks, however, complete decomposition and formation of one equivalent of imidazolium B(Ar^F^)_4_ was observed. Similar observations were also made for the other triflate complexes. Interestingly, for **W‐36** and **W‐41** one equivalent of the corresponding anilinium triflate could be identified as co‐product of the reaction with air. For the other triflate complexes, no signals originating from the imido fragment or from the triflate could be detected, which can be explained by the insolubility of the corresponding anilinium triflates in CDCl_3_. To substantiate this hypothesis, we prepared 2,6‐diisopropylanilinium triflate and 2‐trifluoromethylanilinium triflate, and indeed, the solubility of the former was sufficient to record NMR‐spectra in CDCl_3_, while the latter was completely insoluble in CDCl_3_. The reduced stability of the triflate complexes might be caused by the excellent leaving group character of the triflate anion. While the triflate complexes should not be stored in air, they can be handled in air for limited time without significant decomposition. The NHC ligand also influences the stability of these cationic complexes. Complexes **W‐10**, **W‐09**, **W‐11** bearing the sterically less demanding **IMe**, **IMeCl_2_** and **IiPr**, respectively, were significantly less stable. A large fraction of **W‐09** decomposed after one night in air, while **W‐10** and **W‐11** showed only minor signs of decomposition. After two weeks, however, complete decomposition was observed. Contrarily, **W‐14** bearing the sterically demanding **6‐Mes** was still intact after two weeks. Despite the fact that it is only tetracoordinate, **W‐12** was more stable than the complexes bearing small NHCs, being 90 % intact after 16 h and 15 % intact after two weeks in air.

### Extended Air‐Stability of Cationic Molybdenum Imido Alkylidene NHC Nitrile Complexes

**Figure 4:**
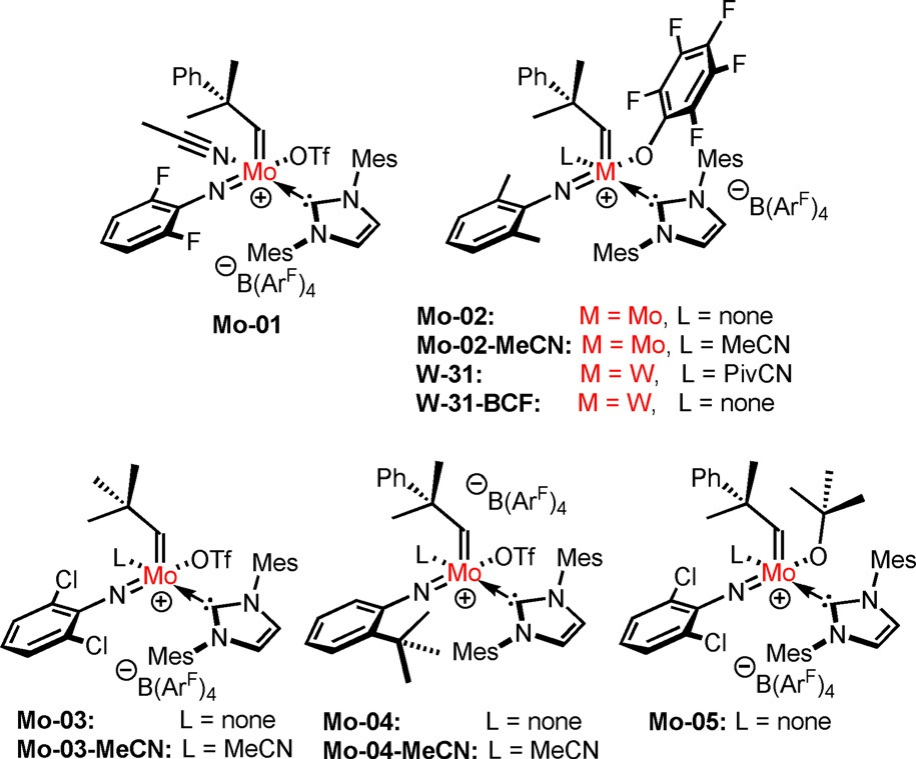
Nitrile‐containing and nitrile‐free cationic tungsten and molybdenum imido alkylidene NHC complexes used for comparing catalytic activity.

Having realized the influence of the nitrile ligand on the air stability of cationic tungsten imido alkylidene NHC complexes, we explored whether this concept could eventually be transferred to the analogous molybdenum compounds. First, we reevaluated the results of our previously conducted experiments on air stability of cationic molybdenum imido alkylidene NHC complexes.^[7a]^ Interestingly, upon storage in air for 16 h, the nitrile‐free complexes all showed the uptake and coordination of water, accompanied by varying amounts of decomposition. The nitrile‐coordinated complex **Mo‐01** (Figure 4), however, showed no signs of decomposition or uptake of water after exposure to air overnight. Therefore, a number of molybdenum imido alkylidene NHC complexes with and without a nitrile ligand were prepared, the structures of which are depicted in Figure 4. Introduction of a nitrile ligand was accomplished by simply adding an excess of nitrile, typically acetonitrile, to a solution of the complex. Usually, no purification except removal of the solvent and excess nitrile under reduced pressure was necessary. As expected, the tetracoordinate compounds **Mo‐02**, **Mo‐03** and **Mo‐05** all showed signs of water uptake in the ^1^H‐NMR spectrum after storing samples in air overnight. Furthermore, partial decomposition was observed for all compounds except for **Mo‐04**. By contrast, the nitrile‐coordinated analogues **Mo‐02‐MeCN**, **Mo‐03‐MeCN** and **Mo‐04‐MeCN** displayed excellent stability and showed no signs of decomposition after being exposed to air overnight. After two weeks, however, **Mo‐04‐MeCN** and **Mo‐03‐MeCN** had decomposed while **Mo‐02‐MeCN** was still intact.

### Influence of the Nitrile Ligand on Activity, Productivity and Selectivity

Evidently, introduction of a nitrile ligand reduces the reactivity of these complexes towards water and oxygen. This posed the question whether this also leads to a reduced productivity in olefin metathesis. This seemed not unlikely, since dissociation of the nitrile ligand at some stage might be part of the reaction mechanism. However, it is currently not known, whether dissociation of the nitrile takes place before or after coordination of the substrate, that is, whether the mechanism is associative or dissociative. To investigate the influence of the nitrile ligand on productivity, the TONs of complexes **Mo‐02**, **Mo‐03** and **Mo‐04** were compared to those of the nitrile‐coordinated analogues **Mo‐02‐MeCN**, **Mo‐03‐MeCN** and **Mo‐04‐MeCN** in a set of benchmark olefin metathesis reactions (Figure 4). A summary of the results obtained is provided in Table 2.

**Table 2:** TONs for benchmark olefin metathesis reactions of **Mo‐02**, **Mo‐02‐MeCN**, **Mo‐03**, **Mo‐03‐MeCN**, **Mo‐04**, **Mo‐04‐MeCN** and the 2^nd^‐generation Grubbs’ catalyst (**G2**). *E*‐content (%) of the products is given in parentheses.

	**S1**	**S2**	**S3**	**S4**	**S5**	**S6**
**Mo‐02**	1580 (91)	1870	1660	1480	370	1410
**Mo‐02‐MeCN**	1450 (92)	1950	1550	1480	410	1430
**Mo‐03**	1120 (91)	0	990	440	0	1350
**Mo‐03‐MeCN**	1330 (93)	0	700	460	0	1520
**Mo‐04**	1120 (92)	0	560	450	270	1150
**Mo‐04‐MeCN**	1100 (91)	0	620	450	0	1230
**G2**	1200 (88)	360	190	330	1250	1130

Reaction conditions: catalyst:substrate=1:2500, room temperature, 3 h, 1,2‐dichloroethane, 0.5 M in substrate, internal standard: dodecane, conversion determined via GC‐MS.

In general, the molybdenum complexes appear to be more active than their tungsten counterparts. Surprisingly, nitrile coordination does not appear to significantly reduce productivities of the molybdenum complexes. This is attributable to the large excess of substrate employed in the reaction, that is, even a weakly coordinating substrate such as 1‐octene could displace the nitrile ligand for stoichiometric reasons. To simulate the influence of a substrate bearing a coordinating functional group, 100 equivalents of CD_3_OD were added to a solution of **W‐33** in CDCl_3_ (Figures S239 and S240, S.I.). Changes in chemical shifts and a broadening of all signals was observed. The chemical shift of PivCN changed from *δ*=0.87 ppm in **W‐33** to *δ*=1.30 ppm, almost the shift of free PivCN (CDCl_3_, *δ*=1.36 ppm). Together with the observed broadening of the signal, this suggests coordination of CD_3_OD and an equilibrium between loosely bound and free PivCN, which could easily be displaced by an olefin. The productivities of the cationic molybdenum and tungsten imido alkylidene NHC were also compared to the productivities obtained with the 2^nd^‐generation Grubbs catalyst (**G2**, Table 2). For most substrates, **G2** exhibited lower productivities than the pentafluorophenoxide complexes. However, comparable productivities were obtained for 1‐octene and **G2** markedly outperformed the tungsten and molybdenum complexes in the self‐metathesis of methyl oleate. This is a prime example for how different catalyst systems complement each other.

**Mo‐03**, **Mo‐04** and their nitrile coordinated analogues **Mo‐03‐MeCN** and **Mo‐04‐MeCN** were also employed in the ROMP of 100 equivalents of enantiomerically pure *endo,exo*‐2,3‐dicarbomethoxynorborn‐5‐ene (DCMNBE, **M1**, Figure 3) to yield >98 % *trans*‐isotactic polymers (Figure S189–S192, S.I.).^[8c]^ All complexes allowed for full conversion after 24 h at room temperature and no influence of the nitrile on selectivity was observed. However, a slight influence on both the number‐average molecular weight, *M_n_*, and the polydispersity, *Đ,* was noticed, with the nitrile free complex **Mo‐04** (*M*_n_=49 kg mol^−1^, *Đ*=1.18) resulting in a slightly lower *M_n_* and *Đ* than obtained with its nitrile‐coordinated analogue **Mo‐04‐MeCN** (*M*_n_=54 kg mol^−1^, *Đ*=1.33). Similar observations were made for **Mo‐03** (*M*_n_=50 kg mol^−1^, *Đ*=1.36) and **Mo‐03‐MeCN** (*M*_n_=74 kg mol^−1^, *Đ*=1.31).

**Figure 5:**
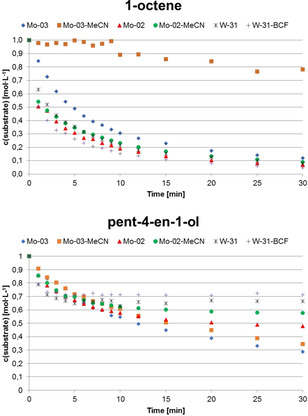
Substrate concentration vs. time for the metathesis of 1‐octene (top) and pent‐4‐en‐1‐ol (bottom) catalyzed by **Mo‐03**, **Mo‐04**, **Mo‐04‐MeCN**, **Mo‐03‐MeCN**, **W‐31** and **W‐31‐BCF**. General conditions: 1,2‐dichloroethane, 1 M in substrate, room temperature. Conversion was determined via GC‐MS using *n*‐dodecane as internal standard. Substrate loading for **Mo**‐compounds: 10 000 equiv. Substrate loading for 1‐octene with **W**‐compounds: 5000 equiv. Substrate loading for pent‐4‐en‐1‐ol with **W**‐compounds: 2500 equiv.

In order to compare the activity of nitrile‐containing complexes to their nitrile‐free analogues, kinetic measurements for the metathesis of 1‐octene and pent‐4‐en‐1‐ol were conducted (Figure 5). Compounds **Mo‐03**, **Mo‐04**, **Mo‐03‐MeCN** and **Mo‐04‐MeCN** were employed. In the case of tungsten, the nitrile‐free complex was obtained from nitrile‐containing complex **W‐31** by reaction with six equivalents of the strong Lewis‐acid tris(pentafluorophenyl)borane (**BCF**). This led to abstraction of the nitrile ligand, that is, formation of the nitrile free complex, and formation of the PivCN adduct of **BCF**, as shown by ^1^H‐NMR (Figure S193–S196, S.I.). While the nitrile could be removed completely from **W‐31**, in some cases, for example, **W‐33**, only partial removal was possible, even upon adding a large excess of borane. Interestingly, only very minor differences for the activity of nitrile‐ligated and nitrile‐free complexes could be observed. So far, we cannot fully rationalize this similar behavior. Only **Mo‐03‐MeCN** showed reduced activity in the metathesis of 1‐octene compared to the nitrile‐free **Mo‐03**. No pronounced differences in activity for the metathesis of pent‐4‐en‐1‐ol were observed.

We do not know yet, whether the nitrile ligand dissociates prior to or after coordination of the substrate, that is, if the reaction proceeds via a dissociative or associative mechanism with the chosen substrates.^[17]^ Investigations regarding this topic are currently underway.

## Conclusion

A synthetic route to cationic tungsten imido alkylidene NHC complexes has been elaborated. It is applicable to a large variety of imido‐ and NHC ligands and allows for both, the preparation of the tungsten analogues of previously reported cationic molybdenum imido alkylidene NHC compounds and the investigation of the influence of the metal on the catalytic performance in olefin metathesis. Introduction of a nitrile ligand to cationic molybdenum and tungsten complexes results in air‐stable complexes, usually without any loss in activity, productivity or selectivity. Unlike previously reported air‐stable 18‐electron Schrock‐type alkylidene complexes, no activation with Lewis acids is required. Cationic complexes containing a nitrile, an electron‐withdrawing imido and an alkoxide ligand appear particularly promising, since they display high productivity while also being very stable towards air. Owing to their excellent stability, they represent user‐friendly group VI metal‐based olefin metathesis initiators. Furthermore, they offer access to metathesis products with a high *E*‐content and form *trans*‐isotactic poly(norbornene)s and are therefore complementary to commonly used ruthenium‐based initiators.

## Conflict of interest

The authors declare no conflict of interest.

## Supporting information

As a service to our authors and readers, this journal provides supporting information supplied by the authors. Such materials are peer reviewed and may be re‐organized for online delivery, but are not copy‐edited or typeset. Technical support issues arising from supporting information (other than missing files) should be addressed to the authors.

Supplementary
