# Supplementary material for: Cationic Group VI Metal Imido Alkylidene N‐Heterocyclic Carbene Nitrile Complexes: Bench‐Stable, Functional‐Group‐Tolerant Olefin Metathesis Catalysts
Source: Angew Chem Int Ed Engl. 2020 Nov 19;60(3):1374–82. doi: 10.1002/anie.202011666 (PMC7839548; doi:10.1002/anie.202011666)
Supplement: Supplementary file 1 — Supplementary [file ANIE-60-1374-s001.pdf]

Angewandte Chemie
Eine Zeitschrift der Gesellschaft Deutscher Chemiker

Supporting Information

Cationic Group VI Metal Imido Alkylidene *N*-Heterocyclic Carbene Nitrile Complexes: Bench-Stable, Functional-Group-Tolerant Olefin Metathesis Catalysts

*Mathis J. Benedikter, Janis V. Musso, Wolfgang Frey, Roman Schowner, and Michael R. Buchmeiser**

anie_202011666_sm_miscellaneous_information.pdf

Supporting Information

Table of Contents

1. Catalytic Data 2
2. Experimental Details 2
3. NMR Spectra of Organic Compounds 22
4. NMR-Spectra of Tungsten and Molybdenum Complexes 33
5. NMR spectra of Air-Stability Experiments 150
6. NMR Spectra of Polymerizations 210
7. NMR Experiments 214
8. Single-Crystal X-Ray Data 220
9. References 298

1. Catalytic Data

Table S 1: Turnover numbers (TON) for benchmark metathesis reactions of W-08, W-09, W-10, W-11, W-12 and W-14. *E*-content (%) of the product in parentheses.

	S1	S2	S3	S4	S5	S6
**W-08**	1190 (88)	0	0	0	0	0
**W-09**	150 (59)	0	0	0	0	0
**W-10**	600 (67)	0	0	0	0	0
**W-11**	830 (65)	0	0	0	0	0
**W-12**	1170 (90)	0	200	0	0	0
**W-14**	370 (69)	510	0	0	1490	240

Reaction conditions: catalyst: substrate 1:2500, 1,2-dichloroethane, room temperature, 3 h, 0.5 M in substrate, internal standard: dodecane, conversion determined *via* GC-MS.

2. Experimental Details

General Information

All Reactions were carried out inside a N_2-filled glovebox (LabMaster 130, MBraun) or by using standard Schlenk technique. Diethyl ether, CH_2Cl_2, pentane and toluene were dried by a solvent purification system (SPS, MBraun). 1,2-Dichloroethane (DCE), pivalonitrile (Sigma Aldrich, 98 %) and Novec 7100 (Sigma Aldrich) were distilled from CaH_2 and stored over molecular sieves 3A. 1,2-Dimethoxyethane (DME) and benzene were destilled from LiH and stored over 3Å molecular sieves. Substrates and n-dodecane were distilled from CaH_2 and stored over 3Å molecular sieves for at least 24 h before use. KBr (ABCR, ultra dry; 99.98%, metals basis) and HCl in Et_2O (Alfa Aesar, 2 M) were used as received. Deuterated solvents were purchased from Eurisotop and dried by storage over molecular sieves 3Å. The following compounds were prepared according to literature procedures: 5-hexenyl thioacetate[1], 3-butenyl benzyl ether[2], **M1**[3], IMes[4], IDipp[5], IiPr[6], $[(IMe)_2Ag]_2[Ag_4I_6]$[7], $IMeCl_2$-AgI[8], W(N-2,6-*i*$Pr_2C_6H_3$)($CHCMe_2Ph$)$(OTf)_2$(DME)[9], W(N-2-$CF_3C_6H_4$)($CHCMe_2Ph$)$(OTf)_2$(DME)[10], W(N-2-*t*BuC_6H_4)($CHCMe_2Ph$)$(Me_2Pyr)_2$[11], W(N-2,6-$Cl_2C_6H_3$)($CHCMe_2Ph$)Cl_2(DME) (**W-17**), W(N-3,5-$Me_2C_6H_3$)($CHCMe_2Ph$)Cl_2(DME) (**W-18**)[12], Mo(N-2,6-$Me_2C_6H_3$)($CHCMe_2Ph$)(OC_6F_5)(OTf)(IMes)[13], [Mo(N-2,6-$Me_2C_6H_3$)($CHCMe_2Ph$)(OC_6F_5)(IMes)(MeCN)$^+$ $B(Ar^F)_4^-$] (**Mo-02-MeCN**)[14], [Mo(N-2,6-$Cl_2C_6H_3$)($CHCMe_3$)(OTf)(IMes)$^+$ $B(Ar^F)_4^-$] (**Mo-03**)[15], [Mo(N-2-*t*Bu-C_6H_3)($CHCMe_2Ph$)(OTf)(IMes)$^+$ $B(Ar^F)_4^-$] (**Mo-04**)[15], [Mo(N-2-*t*Bu-C_6H_3)($CHCMe_2Ph$)(OTf)(IMes)(MeCN)$^+$ $B(Ar^F)_4^-$] (**Mo-04-MeCN**)[15], $NaB(Ar^F)_4$[16], $Ag(MeCN)_3B(Ar^F)_4$[17] and WO_2Cl_2[18]. NMR measurements were recorded on a Bruker Avance III 400. Chemical shifts are reported in ppm relative to the solvent signal; coupling constants are listed in Hz. The SEC system was operated in $CHCl_3$ and consisted of a 1260 Infinity System (*Agilent Technologies Inc.*) equipped with a pre-column (8×50 mm) and three consecutive separation columns (8 × 300 mm, *PSS*, Mainz, Germany, porosity 1000 Å, 100 000 Å and 1 000 000 Å, particle size 5 µm) and with an Agilent 1200 Series G1362A RI detector. A flow rate of 1.0 mL/min and a column oven temperature of 35 °C were used. The injection volume was set to 100 µL. The system was calibrated using narrow

polystyrene standards (800 ≤ M_n ≤ 3 000 000 g/mol). Crystal data have been deposited with the Cambridge Crystallographic Data Centre (CCDC): **W-13** CCDC 2023958, **W-14** CCDC 2023959.

Metathesis Reactions. To a 1.5 mL screw-top vial equipped with a stir bar was added a stock solution of substrate (0.5 M in 1,2-Dichloroethane, 300 µL, 150 µmol, 2500 equiv.) containing *n*-dodecane (~0.25 M) as internal standard. An aliquot of a stock solution of catalyst (~10 µL, 0.06 µmol, 1 equiv.) was added to the vigorously stirred substrate solution. The vial was capped and the resulting solution was stirred at room temperature for three hours. A sample (10 µL) of the reaction was withdrawn, diluted with pentane (~1.5 mL) and analyzed by GC-MS.

Kinetics Measurements. To a 1.5 mL screw-top vial equipped with a stir bar was added a stock solution of substrate (1 M in 1,2-dichloroethane, 500 µL, 500 µmol, 2500 equiv. to 10000 equiv.) containing *n*-dodecane (0.25 M) as internal standard. A stock solution of catalyst (in 1,2-dichloroethane, ca. 10 µL, 1 equiv.) was added to the stirred solution. The vial was capped and vigorously shaken to ensure complete mixing. Samples (10 µL) were withdrawn after 1, 2, 3, 4, 5, 6, 7, 8, 9, 10, 12, 15, 20, 30, 60 and 120 minutes and quenched by addition to a mixture of pentane (1.5 mL) and non-dry ethyl acetate (10 µL). Conversion was determined using GC-MS.

ROMP of (+)-*endo,exo*-2,3-dicarbomethoxynorborn-5-ene. To a well stirred solution of a pre-catalyst (2.5 µmol, 1 equiv.) in $CDCl_3$ (500 µL), a solution of (+)-*endo*,*exo*-2,3-dicarbomethoxynorborn-5-ene (52.5 mg, 0.25 mmol, 100 equiv.) in $CDCl_3$ (500 µL) was quickly added. After 24 h, the visibly viscous reaction mixture was transferred to an NMR-tube and a ^{1}H-NMR was recorded to determine conversion and tacticity. The solution was added to stirred methanol (50 mL) dropwise to precipitate the polymer. The resulting suspension was centrifuged, the solvent decanted and the polymer dried *in vacuo*.

Air Stability Experiments. Air stability experiments were conducted by weighing a sample of the complex (typically 15 to 20 mg) into a screw-top vial inside the glovebox. The vial was transferred out of the glovebox, the lid was removed and the open vial was left to stand on the bench overnight or for the time indicated in the main paper. The open vial was then transferred back into the glovebox, the complex dissolved in dry deuterated solvent. ^{1}H- and ^{19}F-NMR-spectra were recorded to determine the degree of decomposition.

2,6-Diisopropylanilinium triflate. Triflic acid (248 µL, 2.82 mmol, 1 equiv.) was added to a solution of 2,6-diisopropylaniline (531 µL, 2.82 mmol) in diethyl ether (40 mL). The reaction mixture was stirred at room temperature for one hour, during which time the formation of a white suspension was observed. The suspension was filtered and the solid was washed with a small amount of diethyl ether and extensively with pentane to give the product as a white solid. Yield: 375 mg (41 %). The compound was sparingly soluble in $CDCl_3$, therefore, 13C-spectra were recorded in acetone-d6. ^{1}H NMR (400 MHz, $CDCl_3$) δ 8.75 (s, 3H), 7.32 – 7.25 (m, 1H), 7.18 – 7.13 (m, 2H), 3.14 (hept, J = 6.6 Hz, 2H), 1.17 (d, J = 6.7 Hz, 12H) ppm. ^{19}F NMR (376 MHz, $CDCl_3$) δ -78.28 ppm. ^{1}H NMR (400 MHz, acetone-d_6) δ 7.58 (dd, J = 8.4, 7.1 Hz, 1H), 7.48 – 7.42 (m, 2H), 2.93 (hept, J = 6.8 Hz, 2H), 1.23 (d, J = 6.8 Hz, 6H), 1.20 (d, J = 6.7 Hz, 6H) ppm. ^{19}F NMR (376 MHz, acetone-d_6) δ -79.08 ppm. ^{13}C NMR (101 MHz, acetone-d_6) δ 144.4, 132.1, 131.2, 125.7, 121.8 (q, $^1J_{CF}$ = 320.5 Hz), 29.2, 24.4, 23.2 ppm. Elemental analysis (%) calc. for $C_{13}H_{20}F_3NO_3S$: C 47.40, H 6,16, N 4.28; found: C 47.82, H 6.201, N 4.43.

2-Trifluoromethylanilinium triflate. Triflic acid (272 µL, 3.10 mmol, 0.73 equiv.) was added to a solution of 2-trifluormethylaniline (531 µL, 4.23 mmol) in diethyl ether (30 mL). The reaction mixture was stirred at room temperature for one hour, during which time the formation of a white suspension was observed. The suspension was filtered and the solid was washed with a small amount of diethyl ether and extensively with pentane to give the product as a white solid. Yield: 640 mg (66 ‘%). The compound is insoluble in $CDCl_3$, therefore, all spectra were recorded in acetone-d6. ^{1}H NMR (400 MHz, acetone-d_6) δ 8.05 (d, J = 7.9 Hz, 1H), 8.02 – 7.97 (m, 1H), 7.95 – 7.85 (m, 2H) ppm. ^{19}F NMR (376 MHz, acetone-d_6) δ -60.98 (s, 3F), -79.14 (s, 3F) ppm. ^{13}C NMR (101 MHz, acetone-d_6) δ 135.6, 133.1, 132.6, 128.9, 128.6 (q, $^{3}J_{CF}$ = 4.9 Hz), 125.8 (q, $^{2}J_{CF}$ = 31.8 Hz), 123.7 (q, $^{1}J_{CF}$ = 272.9 Hz), 121.9 (q, $^{1}J_{CF}$ = 320.5 Hz) ppm. Elemental analysis (%) calc. for $C_8H_7F_6NO_3S$: C 30.88, H 2.27, N 4.50; found: C 30.89, H 2.367, N 4.65.

[Ag(pivCN)$_3^+$ B(Ar^F)$_4^-$]. [Ag(MeCN)$_3^+$ B(Ar^F)$_4^-$].(0.50 g, 0.46 mmol) was dissolved in pivalonitrile (2 mL). The solvent was removed *in vacuo* and the residue was first coevaporated with diethyl ether followed by pentane. The resulting oil crystallized to form a colorless solid while being stored at -35 °C for several days Yield: 560 mg (99 %). ^{1}H NMR (400 MHz, $CDCl_3$) δ 7.76 (s, 8H), 7.59 (s, 4H), 1.32 (s, 27H) ppm. ^{19}F NMR (376 MHz, $CDCl_3$) δ -62.48 ppm. ^{13}C NMR (101 MHz, C_6D_6) δ 161.1 (q, $^{1}J_{BC}$ = 49.7 Hz), 134.4, 129.6 (qq, $^{2}J_{CF}$ = 31.9 Hz, $^{3}J_{CB}$ = 3.5 Hz), 126.9, 124.5 (q, $^{1}J_{CF}$ = 272.6 Hz), 118.1, 28.7, 27.7. ppm. Elemental analysis (%) calcd. for $C_{47}H_{39}BF_{24}N_3Ag$: C 46.25, H 3.22, N 3.44; found: C 46.25, H 3.322, N 3.51.

W(N-2,6-Me$_2$C$_6$H$_3$)$_2$(Cl)$_2$(DME). Trimethylsilyl chloride (TMSCl, 56.8 g, 0523 mmol, 10 equiv.), 2,6-lutidine (25.2 g, 235 mmol, 4.5 equiv.) and 2,6-dimethylaniline (12.7 g, 105 mmol, 2 equiv.) were added to a suspension of WO_2Cl_2 (15.0 g, 52 mmol, 1 equiv.) in DME (100 mL). The resulting orange suspension was heated to 70 °C overnight. The suspension was left to cool to room temperature and was then filtered through a plug of celite. The solid was washed with DME until the runoff was colorless and the solvent was removed from the filtrate *in vacuo*. The residue was stirred with pentane overnight to yield an orange suspension. The suspension was filtered, the solid was washed with pentane to yield the product as an orange powder. Yield: 26.7 g (87 %). ^{1}H NMR (400 MHz, C_6D_6) δ 6.91 (d, J = 7.5 Hz, 4H), 6.63 (t, J = 7.5 Hz, 2H), 3.46 (s, 6H), 3.15 (s, 4H), 2.71 (s, 12H) ppm. ^{13}C NMR (101 MHz, C_6D_6) δ 154.5, 134.9, 127.8, 125.5, 71.1, 64.1, 18.9 ppm. Elemental analysis (%) calcd. for $C_{20}H_{28}Cl_2N_2O_2W$: C 41.19, H 4.84, N 4.80; found: C 41.15, H 4.851, N 4.88.

W(N-2,6-Me$_2$C$_6$H$_3$)$_2$(CHCMe$_2$Ph)$_2$. A solution of neophyl magnesium chloride in diethyl ether (1.96 M, 46.7 mL, 91 mmol, 2 eq) was added to a cold (-30 °C) suspension of W(N-2,6-Me$_2$C$_6$H$_3$)$_2$(Cl)$_2$(DME) (26.7 g, 46 mmol) in diethyl ether (150 mL). The resulting yellow suspension was stirred overnight at room temperature and filtered through a plug of celite. The celite was washed with diethyl ether and the solvent was removed from the filtrate *in vacuo*. The residue was stirred with pentane (ca. 100 mL) for three hours and the resulting suspension was filtered. The solid was washed with pentane and dried *in vacuo* to yield the product as a bright

yellow powder. Yield: 22.4 g (71 %). ^{1}H NMR (400 MHz, C_6D_6) δ 7.37 (dd, *J* = 8.4, 1.2 Hz, 4H), 7.16 (t, *J* = 7.6 Hz, 4H), 7.06 (t, *J* = 7.3 Hz, 2H), 6.97 (d, *J* = 7.9 Hz, 4H), 6.81 (t, *J* = 7.5 Hz, 2H), 2.18 (s, 12H), 1.63 (s, 4H), 1.41 (s, 12H) ppm. ^{13}C NMR (101 MHz, C_6D_6) δ 155.3, 150.9, 132.8, 128.7, 126.6, 126.4, 124.5, 87.9, 39.9, 33.0, 18.8 ppm. Elemental analysis (%) calcd. for $C_{36}H_{44}N_2W$: C 62.79, H 6.44, N 4.07; found: C 62.39, H 6.572, N 4.14.

W(N-2,6-$Me_2C_6H_3$)($CHCMe_2Ph$)($OTf)_2$(DME). This compound was synthesized analogously to the literature-known neopentylidene compound.[19] A cold (-35 °C) solution of triflic acid (4.57 g, 30 mmol, 3 equiv.) in DME (10 mL) was added to a cold solution (-35 °C) of W(N-2,6-$Me_2C_6H_3)_2$(CH_2CMe_2Ph) (7.00 g, 10 mmol) in a mixture of DME (200 mL) and pentane (40 mL). The resulting solution was allowed to warm to room temperature and stirred overnight. The solvent was removed *in vacuo* and the residue coevaporated with diethyl ether followed by pentane. The residue was extracted with CH_2Cl_2, filtered through a pad of celite and the solvent removed *in vacuo* again. The residue is triturated with a mixture of pentane and diethyl ether to give the product as a pale-yellow solid in 33 % yield. The product consisted of a mixture of a *cis*- and *trans* isomer in varying ratios. *Cis*-Isomer: ^{1}H NMR (400 MHz, C_6D_6) δ 12.10 (s, 1H), 7.40 (dd, *J* = 8.5, 1.2 Hz, 2H), 7.06 (dd, *J* = 8.1, 7.5 Hz, 2H), 6.91 – 6.84 (m, 3H), 6.69 (dd, *J* = 8.8, 6.0 Hz, 1H), 3.29 (s, 3H), 3.15 – 3.11 (m, 1H), 3.09 (s, 3H), 2.86 – 2.80 (m, 1H), 2.57 – 2.50 (m, 1H), 2.22 – 2.15 (m, 1H), 1.83 (s, 3H), 1.57 (s, 3H) ppm. ^{19}F NMR (376 MHz, C_6D_6) δ -76.51 (q, *J* = 2.6 Hz), -77.19 (q, *J* = 2.7 Hz) ppm. *Trans*-Isomer: ^{1}H NMR (400 MHz, C_6D_6) δ 11.17 (s, 1H), 7.66 (dd, *J* = 8.5, 1.2 Hz, 2H), 7.10 (dd, *J* = 8.3, 7.5 Hz, 2H), 6.81 (t, *J* = 7.4 Hz, 1H), 6.79 – 6.73 (m, 3H), 3.74 (s, 3H), 3.17 (dd, *J* = 5.7, 4.7 Hz, 2H), 2.75 (dd, *J* = 5.7, 4.7 Hz, 2H), 2.70 (s, 3H), 2.43 (s, 6H), 1.85 (s, 6H) ppm. ^{19}F NMR (376 MHz, C_6D_6) δ -76.93 ppm. ^{13}C NMR (101 MHz, C_6D_6) δ 290.1, 152.0, 151.2, 139.3, 126.9, 126.3, 121.3, 118.1, 74.0, 70.1, 66.5, 62.6, 54.2, 33.5, 19.2 ppm. Elemental analysis (%) calcd. for $C_{24}H_{31}F_6NO_8S_2W$: C 35.01, H 3.79, N 1.70; found: C 34.99, H 3.834, N 1.80.

W(N-2,6-$^iPr_2C_6H_3$)($CHCMe_2Ph$)Br_2(DME) (W-01). Finely ground KBr (3.25 g, 27.3 mmol, 6 equiv.) was added to a solution of W(N-2,6-*i*$Pr_2C_6H_3$)($CHCMe_2Ph$)($OTf)_2$(DME) (4.00 g, 4.55 mmol) in CH_2Cl_2 (100 mL) and stirred at room temperature for 72 h. The progress was checked by taking small samples, removing the solvent and measuring ^{1}H-NMR until the alkylidene signal of the starting material could no longer be observed. The suspension was filtered through a pad of celite and the solvent was removed *in vacuo*. The residue was triturated with pentane to produce a yellow solid. The suspension was filtered, the solid was washed with pentane and dried *in vacuo*. Yield: 3.08 g (91 %). This product was sufficiently pure for further reactions, but could be crystallized from a mixture of CH_2Cl_2 and pentane. ^{1}H NMR (400 MHz, C_6D_6) δ 10.41 (s, 1H), 7.72 (d, *J* = 7.2 Hz, 2H), 7.31 (t, *J* = 7.8 Hz, 2H), 7.09 (t, *J* = 6.5 Hz, 3H), 7.01 – 6.95 (m, 1H), 4.78 (hept., *J* = 6.8 Hz, 2H), 3.16 (s, 6H), 3.11 (s, 4H), 1.82 (s, 6H), 1.41 (d, *J* = 6.8 Hz, 12H) ppm. ^{13}C NMR (101 MHz, C_6D_6) δ 289.4, 154.4, 150.9, 148.7, 128.7, 127.7, 126.8, 126.2, 123.9, 71.6, 62.8, 54.6, 32.1, 28.1, 25.2 ppm. Elemental analysis (%) calcd. for $C_{26}H_{39}Br_2NO_2W$: C 42.13, H 5.30, N 1.89; found: C 42.00, H 5.442, N 1.93.

W(N-2,6-^{i}Pr$_2$C$_6$H$_3$)(CHCMe$_2$Ph)Br$_2$(IMes) (W-02). A solution of IMes (329 mg, 1.08 mmol, 1 equiv.) in toluene (5 mL) was added to a stirred solution of W(N-2,6-iPr$_2$C$_6$H$_3$)(CHCMe$_2$Ph)Br$_2$(DME) (**W-01**, 800 mg, 1.08 mmol) in toluene (10 mL). The solution was stirred at room temperature for one hour. The solvent was removed *in vacuo* and the oily residue was triturated with pentane (15 mL). The resulting suspension was filtered, the solid was washed with pentane and dried *in vacuo*. Yield: 940 mg (91 %). This solid was sufficiently pure for further reactions, but the compound could be recrystallized from a mixture of CH_2Cl_2 and pentane to yield orange crystals in 57 % yield. ^{1}H NMR (400 MHz, C_6D_6) δ 7.51 (s, 1H), 7.43 (d, *J* = 7.2 Hz, 2H), 7.22 (t, *J* = 7.7 Hz, 2H), 7.09 – 7.03 (m, 3H), 6.97 (dd, *J* = 8.3, 6.8 Hz, 1H), 6.60 (s, 2H), 6.51 (s, 2H), 6.11 (d, *J* = 1.7 Hz, 1H), 6.05 (d, *J* = 1.7 Hz, 1H), 4.18 (hept., *J* = 6.7 Hz, 2H), 2.34 (d, *J* = 10.1 Hz, 12H), 1.96 (s, 3H), 1.87 (s, 3H), 1.63 (s, 6H), 1.29 (d, *J* = 6.7 Hz, 12H) ppm. ^{13}C NMR (101 MHz, C_6D_6) δ 288.8, 207.3, 152.6, 152.4, 146.3, 140.2, 139.6, 136.9, 136.1, 135.2, 134.9, 129.7, 129.6, 126.6, 125.9, 125.6, 123.7, 123.1, 122.6, 54.4, 30.5, 28.2, 25.6, 21.0, 20.8, 20.6, 20.3 ppm. Elemental analysis (%) calcd. for $C_{43}H_{53}Br_2N_3W$: C 54.05, H 5.59, N 4.40; found: C 53.88, H 5.655, N 4.37.

W(N-2,6-^{i}Pr$_2$C$_6$H$_3$)(CHCMe$_2$Ph)Br$_2$(IiPr) (W-03). A cold (-35 °C) solution of IiPr (41.1mg, 0.27 mmol, 1 equiv.) in diethyl ether (1 mL) was added to a stirred cold solution of W(N-2,6-*i*Pr$_2$C$_6$H$_3$)(CHCMe$_2$Ph)Br$_2$(DME) (**W-01**, 200 mg, 0.27 mmol) in diethyl ether (7 mL). The solution was stirred at room temperature for one hour; during that time a small amount of solid precipitated. The supernatant solution was decanted into a clean vial and the product was crystallized by storing the solution at -35 °C overnight. A second fraction of crystals was obtained after concentrating the solution and storing it -35 °C overnight. Yield: 144 mg (66 %). ^{1}H NMR (400 MHz, $CDCl_3$) δ 10.30 (s, 1H), 7.50 (d, *J* = 7.3 Hz, 2H), 7.34 (t, *J* = 7.7 Hz, 2H), 7.21 (t, *J* = 7.3 Hz, 1H), 7.18 – 6.95 (m, 5H), 4.53 – 4.19 (m, 3H), 3.35 (s, 1H), 1.79 (s, 3H), 1.64 (s, 3H), 1.40 (d, *J* = 6.5 Hz, 3H), 1.34 – 1.16 (m, 12H), 0.99 (d, *J* = 6.7 Hz, 3H), 0.77 (d, *J* = 6.5 Hz, 3H), 0.27 (s, 3H) ppm. ^{13}C NMR (101 MHz, $CDCl_3$) δ 282.3, 186.9, 149.7, 149.4, 147.9, 143.9, 128.5, 126.6, 126.3, 125.8, 123.4, 118.5, 118.2, 54.0, 53.8, 53.2, 32.3, 28.8, 28.4, 27.3, 26.2, 24.5, 23.8, 23.3, 23.2, 23.1, 22.8, 21.7 ppm. Elemental analysis (%) calcd. for $C_{31}H_{45}Br_2N_3W$: C 46.35, H 5.65, N 5.23; found: C 46.06, H 5.518, N 5.23.

W(N-2,6-^{i}Pr$_2$C$_6$H$_3$)(CHCMe$_2$Ph)Br$_2$(IMeCl$_2$) (W-04). A solution of W(N-2,6-^{i}Pr$_2$C$_6$H$_3$)(CHCMe$_2$Ph)Br$_2$(DME) (**W-01**, 1000 mg, 1.35 mmol) in CH_2Cl_2 (10 mL) was added to a stirred suspension of IMeCl$_2$-AgI (676 mg, 1.7 mmol, 1.25 equiv.) in CH_2Cl_2 (10 mL). After stirring for 20 minutes at room temperature, the suspension was filtered through a plug of celite and the solvent was removed *in vacuo*. The residue was triturated with pentane (20 mL) and the resulting suspension was filtered. The solid was recrystallized from a mixture of CH_2Cl_2 and pentane. The solvent was decanted and the yellow crystals were dissolved in a minimum amount of CH_2Cl_2, filtered through a plug of celite and the solvent removed again to yield a yellow solid. Yield: 945 mg (86 %). ^{1}H NMR (400 MHz, $CDCl_3$) δ 10.18 (s, 1H), 7.48 (d, *J* = 7.3 Hz, 2H), 7.33 (t, *J* = 7.7 Hz, 2H), 7.21 (t, *J* = 7.3 Hz, 1H), 7.20 – 7.10 (m, 3H), 3.77 (s, 2H), 3.40 (s, 6H), 1.75 (s, 3H), 1.66 (s, 3H), 1.27 (d, *J* = 6.8 Hz, 6H), 0.84 (s, 6H) ppm. ^{13}C NMR (101 MHz, $CDCl_3$) δ 284.7, 189.9, 149.7, 149.0, 145.8, 128.6, 126.9, 126.6, 125.9, 123.5, 118.8, 54.4,

36.6, 31.9, 28.7, 28.0, 24.8, 23.9 ppm. Elemental analysis (%) calcd. for $C_{27}H_{35}Br_2Cl_2N_3W$: C 39.74, H 4.32, N 5.02; found: C 39.66, H 4.350, N 4.97.

W(N-2,6-^{i}Pr$_2$C$_6$H$_3$)(CHCMe$_2$Ph)Br$_2$(IMe) (W-05). A solution of W(N-2,6-^{i}Pr$_2$C$_6$H$_3$)(CHCMe$_2$Ph)Br$_2$(DME) (**W-01**, 1000 mg, 1.35 mmol) in CH_2Cl_2 (10 mL) was added to a stirred suspension of [(IMe)$_2$Ag]$_2$[Ag$_4$I$_6$] (606 mg, 0.34 mmol, 0.25 equiv.) in CH_2Cl_2 (10 mL). After stirring for 20 minutes at room temperature, the suspension was filtered through a plug of celite and the solvent was removed *in vacuo*. The residue was triturated with pentane (20 mL) and the resulting suspension was filtered. The pentane was decanted and the solid was stirred with diethyl ether (20 mL) for five minutes. The suspension was filtered and the solid was recrystallized from a mixture of CH_2Cl_2 and pentane. Yield: 395 mg (39 %). ^{1}H NMR (400 MHz, $CDCl_3$) δ 10.06 (s, 1H), 7.54 – 7.48 (m, 2H), 7.31 (t, *J* = 7.7 Hz, 2H), 7.21 – 7.10 (m, 4H), 6.93 (s, 2H), 3.83 (s, 2H), 3.43 (s, 6H), 1.75 (s, 3H), 1.67 (s, 3H), 1.25 (d, *J* = 6.8 Hz, 6H), 0.83 (s, 6H) ppm. ^{13}C NMR (101 MHz, $CDCl_3$) δ 283.6, 189.8, 149.9, 149.6, 145.8, 128.5, 126.6, 126.4, 125.9, 123.3, 123.1, 54.2, 38.8, 32.2, 28.9, 28.1, 25.0, 23.9 ppm. Elemental analysis (%) calcd. for $C_{27}H_{37}Br_2N_3W$: C 43.40, H 4.99, N 5.62; found: C 43.51, H 4.973, N 5.55.

W(N-2,6-^{i}Pr$_2$C$_6$H$_3$)(CHCMe$_2$Ph)Br$_2$(IDipp) (W-06). A solution of IDipp (213 mg, 0.55 mmol, 1 equiv.) in benzene (3 mL) was added to a solution of W(N-2,6-^{i}Pr$_2$C$_6$H$_3$)(CHCMe$_2$Ph)Br$_2$(DME) (**W-01**, 407 mg, 0.55 mmol) in benzene (5 mL). The reaction was stirred at room temperature for 15 minutes and then cooled to -35 °C. The benzene was removed by sublimation and the residue was crystallized from a mixture of CH_2Cl_2, diethyl ether and pentane to yield the product as orange crystals. Yield: 439 mg (77 %). ^{1}H NMR (400 MHz, $CDCl_3$) δ 7.41 (t, *J* = 7.8 Hz, 1H), 7.29 (d, *J* = 1.7 Hz, 1H), 7.24 – 7.09 (m, 11H), 6.99 – 6.91 (m, 3H), 6.87 (s, 1H), 3.50 (hept, *J* = 6.8 Hz, 2H), 3.36 (hept, *J* = 6.6 Hz, 2H), 3.20 (hept, *J* = 6.7 Hz, 2H), 1.39 (d, *J* = 6.6 Hz, 6H), 1.31 (s, 6H), 1.27 (d, *J* = 6.6 Hz, 6H), 1.08 (d, *J* = 6.9 Hz, 12H), 0.90 (d, *J* = 6.7 Hz, 12H) ppm. ^{13}C NMR (101 MHz, $CDCl_3$) δ 285.6, 207.7, 151.9, 151.1, 147.5, 147.1, 146.1, 135.0, 134.6, 131.1, 131.1, 127.8, 126.4, 125.5, 125.2, 124.8, 124.3, 124.2, 123.9, 122.6, 53.6, 30.4, 29.2, 29.1, 27.6, 27.0, 26.9, 25.4, 23.2, 22.6 ppm. Elemental analysis (%) calcd. for $C_{49}H_{65}Br_2N_3W$: C 56.61, H 6.30, N 4.04; found: C 56.67, H 6.411, N 4.00.

W(N-2,6-^{i}Pr$_2$C$_6$H$_3$)(CHCMe$_2$Ph)Br$_2$(6-Mes) (W-07). A solution of 6-Mes (346 mg, 1.08 mmol, 1 equiv.) in toluene (4 mL) was added to a stirred solution of W(N-2,6-iPr$_2$C$_6$H$_3$)(CHCMe$_2$Ph)Br$_2$(DME) (**W-01**, 800 mg, 1.08 mmol) in toluene (6 mL). The solution was stirred for three hours at room temperature and the solvent was removed *in vacuo*. The residue was coevaporated with pentane and crystallized from a mixture of CH_2Cl_2 and pentane. Yield: 765 mg (73 %). ^{1}H NMR (400 MHz, C_6D_6) δ 7.38 (d, *J* = 7.2 Hz, 2H), 7.22 (t, *J* = 7.7 Hz, 2H), 7.07 (t, *J* = 7.3 Hz, 1H), 7.04 – 7.00 (m, 2H), 6.93 (dd, *J* = 8.2, 6.9 Hz, 1H), 6.87 (s, 1H, W=CH), 6.64 (s, 2H), 6.50 (s, 2H), 4.13 (hept., *J* = 6.7 Hz, 2H), 2.67 (q, *J* = 6.4 Hz, 4H), 2.60 (s, 6H), 2.51 (s, 6H), 1.63 (d, *J* = 4.0 Hz, 6H), 1.58 (s, 6H), 1.50 – 1.45 (m, 2H), 1.43 (d, *J* = 6.7 Hz, 6H), 1.33 (d, *J* = 6.7 Hz, 6H). ^{13}C NMR (101 MHz, C_6D_6) δ 292.8, 225.4, 154.0, 152.2, 145.9, 139.7, 139.7, 139.5, 138.8, 138.2, 137.0, 130.0, 129.9, 126.8, 125.8, 124.7, 122.9, 54.9, 47.2, 47.2,

30.4, 27.8, 25.7, 24.1, 21.7, 20.5, 20.5, 19.3 ppm. Elemental analysis (%) calcd. for $C_{44}H_{57}Br_2N_3W$: C 54.39, H 5.91, N 4.32; found: C 53.99, H 6.054, N 4.36.

$[W(N\text{-}2,6\text{-}^iPr_2C_6H_3)(CHCMe_2Ph)Br(IMes)]^+[B(Ar^F)_4]^-$ (W-08). A solution of W(N-2,6-$iPr_2C_6H_3$)($CHCMe_2Ph$)Br_2(IMes) (**W-02**, 466 mg, 0.49 mmol) in CH_2Cl_2 (6 mL) was added to a stirred suspension of $NaB(Ar^F)_4$ (432 mg, 0.49 mmol, 1 equiv.) in CH_2Cl_2 (4 mL). The red suspension was stirred at room temperature for three hours and then filtered through a pad of celite. The solvent was removed *in vacuo* to yield a red foam. Yield: 775 mg (91 %). ^1H NMR (400 MHz, $CDCl_3$) δ 9.44 (s, 1H), 7.73 (s, 8H), 7.53 (s, 4H), 7.43 (s, 2H), 7.32 – 7.14 (m, 6H), 7.12 (s, 2H), 7.03 – 6.98 (m, 2H), 6.90 (s, 2H), 3.17 (hept, J = 6.6 Hz, 2H), 2.35 (s, 6H), 2.04 (s, 6H), 1.95 (s, 6H), 1.48 (s, 3H), 1.17 (d, J = 6.7 Hz, 6H), 0.98 (d, J = 6.8 Hz, 6H), 0.81 (s, 3H) ppm. ^{19}F NMR (376 MHz, $CDCl_3$) δ 62.39 ppm. ^{13}C NMR (101 MHz, $CDCl_3$) δ 286.39, 187.33, 161.85 (q, $^1J_{BC}$ = 49.8 Hz), 152.39, 148.24, 146.14, 143.03, 135.77, 134.95, 134.49, 132.12, 131.08, 130.93, 130.37, 129.04 (qq, $^2J_{CF}$ = 31.5 Hz, $^3J_{CB}$ = 2.8 Hz), 128.70, 127.49, 127.06, 125.05, 124.70 (q, $^1J_{CF}$ = 272.5 Hz), 123.69, 117.60, 54.88, 33.62, 28.80, 28.27, 24.39, 24.14, 21.21, 18.22, 17.78 ppm. Elemental analysis (%) calcd. for $C_{75}H_{65}BBrF_{24}N_3W$: C 51.80, H 3.77, N 2.42; found: C 51.72, H 3.875, N 2.46.

$[W(N\text{-}2,6\text{-}^iPr_2C_6H_3)(CHCMe_2Ph)Br(IMeCl_2)(pivCN)_2]^+[B(Ar^F)_4]^-$ (W-09). $NaB(Ar^F)_4$ (651 mg, 0.74 mmol, 1 equiv.) was added to a solution of W(N-2,6-$^iPr_2C_6H_3$)($CHCMe_2Ph$)Br_2($IMeCl_2$) (**W-04**, 600 mg, 0.74 mmol) and pivalonitrile (200 µl) in CH_2Cl_2 (12 mL). The suspension was stirred at room temperature for three hours and the solvent was removed *in vacuo*. The residue was coevaporated with pentane and extracted with CH_2Cl_2. The resulting suspension was filtered through a pad of celite and the solvent was removed again to yield a yellow foam. Yield: 1140 mg (88 %). ^1H NMR (400 MHz, $CDCl_3$) δ 11.38 (s, 1H), 7.72 (s, 8H), 7.54 (s, 4H), 7.38 – 7.33 (m, 4H), 7.30 – 7.17 (m, 6H), 3.45 (s, 6H), 3.28 (h, J = 6.7 Hz, 2H), 1.75 (s, 3H), 1.71 (s, 3H), 1.45 (s, 9H), 1.36 (s, 9H), 1.28 (d, J = 6.8 Hz, 6H), 0.92 (d, J = 6.9 Hz, 6H) ppm. ^{19}F NMR (376 MHz, $CDCl_3$) δ -62.34 ppm. ^{13}C NMR (101 MHz, $CDCl_3$) δ 304.00, 188.06, 161.83 (q, $^1J_{BC}$ = 49.8 Hz), 149.09, 146.94, 145.05, 141.44, 134.95, 129.59, 129.12, 129.02 (qq, $^2J_{CF}$ = 31.4 Hz, $^3J_{CB}$ = 2.9 Hz), 127.59, 125.67, 124.70 (q, $^1J_{CF}$ = 272.5 Hz), 124.04, 120.18, 117.62, 55.94, 37.66, 31.02, 30.50, 29.61, 28.51, 28.10, 27.08, 24.32, 23.57 ppm. Elemental analysis (%) calcd. for $C_{69}H_{65}BBrCl_2F_{24}N_5W$: C 46.94, H 3.71, N 3.97; found: C 46.93, H 3.769, N 3.89.

$[W(N\text{-}2,6\text{-}^iPr_2C_6H_3)(CHCMe_2Ph)Br(IMe)(pivCN)]^+[B(Ar^F)_4]^-$ (W-10). A solution of W(N-2,6-$iPr_2C_6H_3$)($CHCMe_2Ph$)Br_2(IMe) (**W-05**, 200 mg, 0.268 mmol, 1 equiv.) in CH_2Cl_2 (4 mL) was quickly added to a stirred suspension of $NaB(Ar^F)_4$ (237 mg, 0.268 mmol) in CH_2Cl_2 (6 mL) and pivalonitrile (300 µl). After stirring the suspension at room temperature for three hours the solvent was removed *in vacuo*. The oily residue was stirred with pentane (8 mL) which resulted in the formation of a yellow suspension. The pentane was decanted, fresh pentane (6 mL) was added and decanted and the solid was dried *in vacuo*. The solid was dissolved in CH_2Cl_2, filtered through a pad of celite and the solvent was removed again. The residue was stirred with pentane again to give the product as a yellow solid. Yield: 365 mg (84 %). ^1H NMR (400 MHz,

$CDCl_3$) δ 11.01 (s, 1H), 7.74 (s, 8H), 7.55 (s, 4H), 7.42 – 7.33 (m, 4H), 7.29 – 7.14 (m, 4H), 6.91 (s, 2H), 3.42 (s, 6H), 3.31 (hept, J = 6.7 Hz, 2H), 1.78 (s, 3H), 1.74 (s, 3H), 1.46 (s, 9H), 1.28 (d, J = 6.9 Hz, 6H), 0.89 (d, J = 6.9 Hz, 6H) ppm. ^{19}F NMR (376 MHz, $CDCl_3$) δ -62,31 ppm. ^{13}C NMR (101 MHz, $CDCl_3$) δ 301.2, 188.4, 161.9 (q, $^1J_{BC}$ = 49.8 Hz), 149.4, 146.9, 144.8, 141.1, 134.9, 129.1, 129.1 (qq, $^2J_{CF}$ = 31.5 Hz, $^3J_{CB}$ =2.8 Hz), 127.5, 126.8, 125.7, 124.7 (q, $^1J_{CF}$ = 272.5 Hz), 124.0, 123.9, 122.6, 117.7, 55.7, 39.4, 31.7, 31.0, 30.5, 29.6, 28.7, 27.1, 24.3, 23.4 ppm. Elemental analysis (%) calcd. for $C_{64}H_{58}BBrF_{24}N_4W$: C 47.64, H 3.62, N 3.47; found: C 47.80, H 3.691, N 3.55.

[W(N-2,6-^{i}Pr$_2$C$_6$H$_3$)(CHCMe$_2$Ph)Br(IiPr)(pivCN)]$^+$[B(Ar^F)$_4$]$^-$ (W-11). To a solution of W(N-2,6-^{i}Pr$_2$C$_6$H$_3$)(CHCMe$_2$Ph)Br$_2$(IiPr) (**W-03**, 76.2 mg, 0.10 mmol, 1 equiv.) in CH_2Cl_2 (2 mL) was added a solution of [Ag(pivCN)$_3$][B(Ar^F)$_4$] (116 mg, 0.10 mmol, 1 equiv.) in CH_2Cl_2 (0.5 mL). After stirring the resulting suspension for one hour, it was filtered through a pad of celite and the solvent was removed under reduced pressure. The residue was stirred with pentane (10 mL) for three hours. The pentane was decanted and the residue was dried under reduced pressure to yield a yellow foam. Yield: 149 mg (94 %). ^{1}H NMR (400 MHz, CD_2Cl_2) δ 11.34 (s, 1H), 7.75 (s, 8H), 7.58 (s, 4H), 7.48 – 7.39 (m, 4H), 7.34 – 7.29 (m, 1H), 7.23 (d, J = 7.6 Hz, 3H), 7.16 (s, 2H), 4.16 (hept, J = 6.7 Hz, 2H), 3.41 (hept, J = 6.2 Hz, 2H), 1.87 (s, 3H), 1.72 (s, 3H), 1.53 (s, 9H), 1.36 (s, 3H), 1.33 (d, J = 6.8 Hz, 6H), 1.24 (s, 6H), 0.97 (s, 6H), 0.39 (s, 3H) ppm. ^{19}F NMR (376 MHz, CD_2Cl_2) δ -62.79 ppm. ^{13}C NMR (101 MHz, CD_2Cl_2) δ 300.1, 186.1, 162.3 (q, $^1J_{BC}$ = 49.9 Hz), 149.8, 147.4, 145.5, 141.7, 135.4, 129.6, 129.5 (qq, $^2J_{CF}$ = 31.5 Hz, $^3J_{CB}$ =2.9 Hz), 127.9, 126.2, 125.2 (q, $^1J_{CF}$ = 272.6 Hz), 124.3, 119.8, 118.1, 55.7, 55.0, 32.0, 31.0, 29.3, 27.5, 23.8, 22.4. ppm. Elemental analysis (%) calcd. for $C_{68}H_{66}BBrF_{24}N_4W$: C 48.91, H 3.98, N 3.36; found: C 49.04, H 4.069, N 3.53.

[W(N-2,6-^{i}Pr$_2$C$_6$H$_3$)(CHCMe$_2$Ph)Br(IDipp)]$^+$[B(Ar^F)$_4$]$^-$ (W-12). A solution of W(N-2,6-^{i}Pr$_2$C$_6$H$_3$)(CHCMe$_2$Ph)Br$_2$(IDipp) (**W-06**, 124 mg, 0.14 mmol) in CH_2Cl_2 (4 mL) was quickly added to a stirred suspension of NaB(Ar^F)$_4$ (101 mg, 0.14 mmol, 1 equiv.) in CH_2Cl_2 (2 mL). The red suspension was stirred at room temperature for two hours and then filtered through a pad of celite. The solvent was removed under reduced pressure to give a red foam. The foam was stirred with pentane until an orange powder formed. The pentane was decanted and the solid was dried *in vacuo*. Yield: 198 mg (78 %). The compound can be recrystallized from a mixture of CH_2Cl_2 and *n*-pentane. ^{1}H NMR (400 MHz, CD_2Cl_2) δ 8.98 (s, 1H), 7.75 (s, 8H), 7.70 – 7.64 (m, 4H), 7.58 (s, 4H), 7.52 (dd, J = 7.9, 1.2 Hz, 3H), 7.29 (dd, J = 7.8, 1.4 Hz, 2H), 7.27 – 7.11 (m, 6H), 7.02 – 6.98 (m, 2H), 1.45 (d, J = 2.4 Hz, 6H), 1.43 (s, 3H), 1.22 (d, J = 6.7 Hz, 6H), 1.18 (d, J = 6.7 Hz, 6H), 1.04 (d, J = 6.6 Hz, 6H), 1.01 (d, J = 6.7 Hz, 6H), 0.84 (s, 6H), 0.75 (s, 3H). ppm. ^{19}F NMR (376 MHz, CD_2Cl_2) δ -62.78 ppm. ^{13}C NMR (101 MHz, CD_2Cl_2) δ 283.6, 192.1, 162.4 (q, $^1J_{BC}$ = 49.8 Hz), 153.0, 148.2, 146.9, 145.0, 135.4, 133.5, 131.4, 129.5 (qq, $^2J_{CF}$ = 31.5 Hz, $^3J_{CB}$ =2.9 Hz), 129.4, 129.0, 127.5, 126.8, 125.7, 125.7, 125.2 (q, $^1J_{CF}$ = 272.7 Hz), 124.4, 118.1, 55.5, 33.2, 30.8, 29.7, 29.0, 28.3, 27.4, 26.6, 24.6, 23.8, 22.7, 20.5 ppm. Elemental analysis (%) calcd. for $C_{81}H_{77}BBrF_{24}N_3W$: C 53.37, H 4.26, N 2.30; found: C 53.50, H 4.276, N 2.35.

[W(N-2,6-^{i}Pr$_2$C$_6$H$_3$)(NCMeCH(CMe$_2$Ph))Br(6-Mes)]$^+$[B(Ar^F)$_4$]$^-$ (W-13). A solution of [Ag(MeCN)$_3$][B(Ar^F)$_4$] (113 mg, 0.10 mmol, 1 equiv.) in CH_2Cl_2 (2 mL) was added to a solution of W(N-2,6-^{i}Pr$_2$C$_6$H$_3$)(CHCMe$_2$Ph)Br$_2$(6-Mes) (**W-07**, 100 mg, 0.10 mmol) in CH_2Cl_2 (2 mL) and stirred for three hours. The suspension was filtered through celite and the solvent was removed *in vacuo* to yield an orange foam. Yield: 160 mg (87 %). Crystals suitable for single crystal X-ray diffraction were obtained using the following procedure: The foam was stirred with Novec 7100 engineered fluid, which led to the formation of a yellow suspension. ThW-13e suspension was filtered and the solid was washed with Novec 7100 and heptane. The solid was then dissolved by addition of 1,2-dichloroethane and the solution collected in an empty vial. The solution was layered with heptane and kept at -35 °C for several days; during that time yellow crystals of the product formed. ^{1}H NMR (400 MHz, CDCl$_3$) δ 7.75 (s, 8H), 7.55 (s, 4H), 7.38 – 7.31 (m, 4H), 7.28 – 7.22 (m, 1H), 7.17 – 7.10 (m, 3H), 7.03 (s, 2H), 6.76 (s, 2H), 5.49 (s, 1H), 3.58 – 3.41 (m, 4H), 3.10 (hept, J = 6.6 Hz, 2H), 2.42 (s, 6H), 2.34 (s, 6H), 2.22 (s, 6H), 1.59 (s, 3H), 1.57 (s, 3H), 1.26 (s, 3H), 1.15 (d, J = 6.8 Hz, 6H), 1.07 (d, J = 6.8 Hz, 6H) ppm.^{19}F NMR (376 MHz, CDCl$_3$) δ -62.35 ppm. ^{13}C NMR (101 MHz, CDCl$_3$) δ 200.0, 161.8 (q, $^1J_{BC}$ = 49.8 Hz), 153.1, 149.9, 148.7, 148.6, 144.8, 142.9, 137.8, 135.0, 132.9, 132.5, 130.7, 129.1 (qq, $^2J_{CF}$ = 31.5 Hz, $^3J_{CB}$ = 2.8 Hz), 128.7, 126.6, 126.1, 124.7 (q, $^1J_{CF}$ = 272.4 Hz), 121.8, 117.6, 49.2, 39.3, 32.8, 29.5, 28.5, 24.0, 23.0, 21.5, 19.9, 18.1, 17.6, 16.9 ppm. Elemental analysis (%) calcd. for $C_{78}H_{72}BBrF_{24}N_4W$: C 52.16, H 4.04, N 3.12; found: C 52.23, H 3.989, N 3.20.

[W(N-2,6-^{i}Pr$_2$C$_6$H$_3$)(CHCMe$_2$Ph)Br(6-Mes)(pivCN)]$^+$[B(Ar^F)$_4$]$^-$ (W-14). A solution of [Ag(pivCN)$_3$][B(Ar^F)$_4$] (754 mg, 0.62 mmol, 1 equiv.) in CH_2Cl_2 (10 mL) was added to a stirred solution of W(N-2,6-^{i}Pr$_2$C$_6$H$_3$)(CHCMe$_2$Ph)Br$_2$(6-Mes) (**W-07**, 600 mg, 0.62 mmol) in CH_2Cl_2 (10 mL). After stirring for one hour at room temperature, the suspension was filtered through a pad of celite and the solvent was removed *in vacuo*. The residue was dissolved in CH_2Cl_2 (ca. 6 mL) and pentane (ca. 20 mL) was slowly added until slight turbidity was observed. Upon standing at room temperature, orange crystals of the desired product formed. Yield: 1077 mg (95 %). The compound forms a second isomer, the NMR-signal of which appears at 8.68 ppm. This signal was identified as an isomer by variable-temperature NMR. ^{1}H NMR (400 MHz, **-40 °C**, CDCl$_3$) δ 8.68 (s, 0.03H, W=CH, minor isomer), 8.00 (s, 0.97H, W=CH. major isomer), 7.73 (s, 8H), 7.54 (s, 4H), 7.34 – 7.19 (m, 3H), 7.12 – 7.01 (m, 3H), 6.97 (d, J = 7.1 Hz, 2H), 6.79 (s, 1H), 6.66 (s, 1H), 6.63 (s, 1H), 6.54 (s, 1H), 3.63 – 3.31 (m, 6H), 2.71 (hept, J = 6.8 Hz, 1H), 2.49 (s, 3H), 2.46 (s, 3H), 2.38 (s, 3H), 2.30 (s, 6H), 1.89 (s, 3H), 1.49 (s, 9H), 1.43 (s, 3H), 1.38 – 1.09 (m, 18H), 0.95 (d, J = 6.7 Hz, 3H) ppm.^{19}F NMR (376 MHz, CDCl$_3$) δ -62.33 ppm. ^{13}C NMR (101 MHz, CDCl$_3$) δ 303.7, 221.3, 161.8 (q, $^1J_{BC}$ = 49.8 Hz), 150.0, 149.4, 147.1, 145.6, 142.7, 141.7, 141.2, 139.1, 138.9, 137.7, 136.7, 134.9, 134.2, 133.9, 132.4, 131.6, 131.4, 129.6, 129.4, 129.0 (qq, $^2J_{CF}$ = 34.5 Hz, $^3J_{CB}$ = 3.0 Hz), 128.5, 126.9, 126.8, 125.6, 125.0, 124.7 (q, $^1J_{CF}$ = 272.5 Hz), 124.2, 123.8, 122.5, 117.6, 55.7, 53.6, 47.9, 47.8, 31.7, 30.0, 28.8, 28.6, 28.5, 27.9, 27.7, 27.6, 25.1, 24.9, 24.6, 24.1, 23.6, 22.8, 21.1, 20.8, 20.3, 19.6, 19.6, 19.2 ppm. Elemental analysis (%) calcd. for $C_{81}H_{78}BBrF_{24}N_4W$: C 52.93, H 4.28, N 3.05; found: C 53.31, H 4.458, N 3.12.

W(N-2,6-Me$_2$C$_6$H$_3$)(CHCMe$_2$Ph)Br$_2$(DME) (W-15). This compound was prepared analogously to **W-01** from W(N-2,6-Me$_2$C$_6$H$_3$)(CHCMe$_2$Ph)(OTf)$_2$(DME) (2.00 g, 2.4 mmol) and KBr (1.734 g,

14.6 mmol, 6 equiv.) in 50 mL CH_2Cl_2. After washing with pentane, the compound was crystallized from a mixture of CH_2Cl_2 and pentane to obtain analytically pure product as red crystals. Yield: 1.05 g (63 %). ^{1}H NMR (400 MHz, C_6D_6) δ 10.42 (s, 1H), 7.73 (d, J = 7.4 Hz, 2H), 7.28 (t, J = 7.7 Hz, 2H), 7.05 (t, J = 7.3 Hz, 1H), 6.83 (d, J = 7.4 Hz, 2H), 6.76 (t, J = 6.5 Hz, 1H), 3.17 (s, 6H), 3.15 (s, 4H), 2.92 (s, 6H), 1.78 (s, 6H) ppm. ^{13}C NMR (101 MHz, C_6D_6) δ 289.2, 154.0, 153.6, 139.1, 128.6, 126.9, 126.7, 126.2, 71.7, 62.8, 54.3, 32.0, 22.2 ppm. Elemental analysis (%) calcd. for $C_{22}H_{31}Br_2NO_2W$: C 38.57, H 4.56, N 2.04; found: C 38.26, H 4.609, N 2.16.

W(N-2-*t*BuC$_6$H$_4$)(CHCMe$_2$Ph)Cl$_2$(DME) (W-19). Cold (-35 °C) HCl (2 M in Et_2O, 1.82 mL, 3.64 mmol, 2 equiv.) was added to a cold suspension of W(N-2-*t*BuC$_6$H$_4$)(CHCMe$_2$Ph)(Me$_2$Pyr)$_2$ (1.19 g, 1.82 mmol) in a mixture of DME (2 mL) and diethyl ether (40 mL). The reaction was stirred at room temperature for 30 minutes during which time it turned into an orange suspension. The suspension was filtered and the solid was washed with a small amount of cold diethyl ether to yield the product as a yellow solid. Yield:0.94 g (82 %). ^{1}H NMR (400 MHz, C_6D_6) δ 10.18 (s, 1H), 8.44 (d, J = 7.7 Hz, 1H), 7.69 (d, J = 7.3 Hz, 2H), 7.29 (t, J = 7.8 Hz, 2H), 7.25 (d, J = 6.8 Hz, 1H), 7.05 (q, J = 7.5 Hz, 2H), 6.88 (t, J = 6.9 Hz, 1H), 3.16 (s, 6H), 3.09 (s, 4H), 1.83 (s, 6H), 1.72 (s, 9H) ppm. ^{13}C NMR (101 MHz, C_6D_6) δ 281.4, 155.4, 154.4, 144.6, 131.4, 128.6, 127.4, 126.8, 126.6, 126.1, 126.0, 71.8, 62.4, 54.4, 35.7, 32.8, 30.6 ppm. Elemental analysis (%) calcd. for $C_{24}H_{35}Cl_2NO_2W$: C 46.17, H 5.65, N 2.24; found: C 45.91, H 5.684, N 2.40.

W(N-2,6-Me$_2$C$_6$H$_3$)(CHCMe$_2$Ph)Br$_2$(IMes) (W-20). A solution of IMes (222 mg, 0.730 mmol) in toluene (4 mL) was added to a solution of W(N-2,6-Me$_2$C$_6$H$_3$)(CHCMe$_2$Ph)Br$_2$(DME) (**W-15**, 500 mg, 0.730 mmol) in toluene (10 mL) and stirred for two hours at room temperature. During this time, an orange precipitate formed, which was filtered off, washed with pentane and dried *in vacuo*. Yield: 590 mg (90 %). ^{1}H NMR (400 MHz, CD_2Cl_2) δ 8.62 (s, 1H), 7.32 – 6.72 (m, 14H), 2.64 (s, 3H), 2.51 – 1.94 (m, 18H), 1.87 (s, 3H), 1.79 (s, 3H), 1.51 (s, 3H) ppm. ^{13}C NMR (101 MHz, CD_2Cl_2) δ 290.8, 194.4, 153.3, 151.6, 140.1, 140.0, 135.9, 135.4, 132.5, 130.1, 129.3, 128.5, 128.2, 127.3, 127.3, 126.7, 126.0, 125.7, 125.2, 35.1, 29.1, 21.8, 21.2, 20.3, 19.8 ppm. Elemental analysis (%) calcd. for $C_{39}H_{45}Br_2N_3W$: C 52.08, H 5.04, N 4.67; found: C 52.06, H 4.957, N 4.58.

W(N-2-CF$_3$C$_6$H$_4$)(CHCMe$_2$Ph)Br$_2$(IMes) (W-21). Solid KBr (1.24 g, 10.4 mmol, 6 eq) was added to a solution of W(N-2-CF$_3$C$_6$H$_4$)(CHCMe$_2$Ph)(OTf)$_2$(DME) (1.50 g, 1.7 mmol) in CH_2Cl_2 (50 mL). The resulting suspension was stirred at room temperature until ^{1}H-NMR indicated full conversion (three days). The solvent was removed under reduced pressure and the residue was dissolved in benzene (50 mL). A solution of IMes (0.53 g, 1.7 mmol) in benzene (10 mL) was added and the red solution was stirred at room temperature for one hour. The solvent was removed *in vacuo* and the residue was recrystallized from a mixture of CH_2Cl_2 and pentane to yield the product as red crystals. Yield: 1.55 g (95 %). In $CDCl_3$, the compound exists as a mixture of two isomers at a ratio of 9:1. Only the signals for the major isomer are given below. ^{1}H NMR (400 MHz, $CDCl_3$) δ 9.09 (s, 1H), 7.40 (d, J = 7.7 Hz, 1H), 7.29 (t, J = 7.5 Hz, 1H), 7.24 –

7.12 (m, 7H), 7.08 (s, 2H), 6.56 (s, 2H), 6.46 (s, 2H), 2.11 (s, 6H), 2.08 (s, 12H), 1.69 (s, 3H), 1.64 (s, 3H) ppm. ^{19}F NMR (376 MHz, $CDCl_3$) δ -55.17 ppm. ^{13}C NMR (101 MHz, $CDCl_3$) δ 291.8, 194.6, 150.7, 150.4, 139.5, 135.6, 135.3, 135.0, 132.6, 131.2, 129.9, 129.3, 128.1, 126.3 (q, *J* = 3.8 Hz), 125.9, 125.9, 125.8, 124.7, 53.8, 35.0, 29.6, 21.1, 19.7, 19.5 ppm. Elemental analysis (%) calcd. for $C_{38}H_{40}Br_2F_3N_3W$: C 48.59, H 4.29, N 4.47; found: C 48.44, H 4.269, N 4.44.

W(N-2,6-$Cl_2C_6H_3$)($CHCMe_2Ph$)Cl_2(IMes) (W-22). A solution of IMes (294 mg, 0.97 mmol) in toluene (4 mL) was added to a solution of W(N-2,6-$Cl_2C_6H_3$)($CHCMe_2Ph$)Cl_2(DME) (**W-17**, 615 mg, 0.97 mmol) in toluene (15 mL). The mixture was stirred at room temperature for three hours. During this time, an orange precipitate formed. The suspension was filtered and the precipitate was washed with a small amount of toluene and pentane to yield the product as a yellow solid. Yield: 691 mg (84 %). ^{1}H NMR (400 MHz, $CDCl_3$) δ 9.45 (s, 1H), 7.25 – 7.01 (m, 9H), 6.84 (t, *J* = 8.0 Hz, 1H), 6.56 (s, 2H), 6.53 (s, 2H), 2.15 (s, 6H), 2.05 (s, 6H), 2.00 (s, 6H), 1.88 (s, 3H), 1.80 (s, 3H) ppm. ^{13}C NMR (101 MHz, $CDCl_3$) δ 295.1, 195.8, 151.7, 149.6, 139.4, 137.9, 135.6, 135.1, 129.7, 128.8, 127.9, 127.3, 126.1, 125.6, 125.6, 124.4, 53.2, 36.6, 29.4, 21.1, 19.4, 19.3 ppm. Elemental analysis (%) calcd. for $C_{37}H_{39}Cl_4N_3W$: C 52.20, H 4.62, N 4.94; found: C 52.44, H 4.413, N 4.79.

W(N-3,5-$Me_2C_6H_3$)($CHCMe_2Ph$)Cl_2(IMes) (W-23). A solution of IMes (153 mg, 0.50 mmol) in toluene (3 mL) was added to a suspension of W(N-3,5-$Me_2C_6H_3$)($CHCMe_2Ph$)Cl_2(DME) (**W-18**, 300 mg, 0.50 mmol) in toluene (3 mL). A clear red solution formed, which was stirred for two hours at room temperature. Pentane was added (ca. 4 mL) and the solution was stored at -35 °C overnight. During this time, orange needles formed. The supernatant solution was decanted and the solid was washed with pentane three times and dried *in vacuo* to give the product as orange crystals. Yield: 370 mg (91 %). ^{1}H NMR (400 MHz, $CDCl_3$) δ 10.12 (s, 1H), 7.27 – 7.13 (m, 5H), 7.03 (s, 2H), 6.70 (s, 1H), 6.52 (s, 2H), 6.49 (s, 2H), 6.46 (s, 2H), 2.21 (s, 6H), 2.11 (s, 6H), 2.09 (s, 6H), 2.00 (s, 6H), 1.72 (s, 3H), 1.43 (s, 3H) ppm. ^{13}C NMR (101 MHz, $CDCl_3$) δ 286.3, 194.7, 154.0, 150.4, 139.3, 136.7, 135.5, 135.3, 134.5, 129.4, 129.4, 128.2, 127.9, 126.2, 125.6, 124.6, 124.2, 52.4, 35.1, 30.1, 21.3, 21.1, 19.2, 19.0 ppm. Elemental analysis (%) calcd. for $C_{39}H_{45}Cl_2N_3W$: C 57.79, H 5.60, N 5.18; found: C 57.86, H 5.474, N 5.11.

W(N-2-*t*BuC_6H_4)($CHCMe_2Ph$)Cl_2(IMes) (W-24). A cold (-35 °C) solution of IMes (390 mg, 1.28 mmol, 1 equiv.) in toluene (6 mL) was added to a cold solution of W(N-2-*t*BuC_6H_4)($CHCMe_2Ph$)Cl_2(DME) (**W-19**, 800 mg, 1.28 mmol) in toluene (30 mL). The orange solution was stirred at room temperature for two hours. The solvent was removed under reduced pressure and the residue was stirred with pentane until a solid formed. The solid was recrystallized from a mixture of CH_2Cl_2 and pentane to yield the product as orange crystals. Yield: 1029 mg (96 %). ^{1}H NMR (400 MHz, C_6D_6) δ 8.06 (dd, *J* = 8.0, 1.5 Hz, 1H), 7.40 (dd, *J* = 8.4, 1.2 Hz, 2H), 7.32 (s, 1H, W=CH), 7.23 – 7.17 (m, 4H), 7.06 (t, *J* = 7.3 Hz, 1H), 6.94 (t, *J* = 7.6 Hz, 0H), 6.80 (td, *J* = 7.5, 1.5 Hz, 1H), 6.12 (d, *J* = 1.8 Hz, 1H), 6.04 (d, *J* = 1.8 Hz, 1H), 2.31 (s, 12H), 1.95 (s, 3H), 1.84 (s, 3H), 1.58 (s, 6H), 1.47 (s, 9H) ppm. ^{13}C NMR (101 MHz, C_6D_6) δ 281.7, 207.4, 155.8, 153.5, 143.1, 140.3, 139.5, 137.0, 136.2, 135.2, 134.4, 129.6, 128.7, 126.5,

126.3, 125.8, 125.3, 124.9, 123.2, 122.0, 54.2, 35.3, 31.2, 30.4, 20.9, 20.9, 19.6, 19.1 ppm. Elemental analysis (%) calcd. for $C_{41}H_{49}Cl_2N_3W$: C 58.72, H 5.89, N 5.01; found: C 58.52, H 5.902, N 4.93.

[W(N-2,6-$Me_2C_6H_3$)($CHCMe_2Ph$)Br(IMes)(pivCN)$^+$ B(Ar^F)$_4^-$] (W-25). A solution of W(N-2,6-$Me_2C_6H_3$)($CHCMe_2Ph$)Br_2(IMes) (**W-20**, 250 mg, 0.278 mmol) in CH_2Cl_2 (4 mL) was added to a suspension of NaB(Ar^F)$_4$ (246 mg, 0.278 mmol) in CH_2Cl_2 (4 mL). The suspension was stirred at room temperature for two hours and filtered through a pad of celite. Pivalonitrile (300 µL) was added to the red solution, which led to an instantaneous color change to orange. The solvent was removed *in vacuo* and the residue was triturated with pentane to yield a yellow suspension. The pentane was decanted and the solid was recrystallized at -35 °C from a mixture of CH_2Cl_2, diethyl ether and pentane to yield the product as a single orange crystal. Yield: 365 mg (74 %). ^{1}H NMR (400 MHz, CD_2Cl_2) δ 9.72 (s, 1H), 7.75 (s, 8H), 7.58 (s, 4H), 7.31 – 6.95 (m, 10H), 6.91 (s, 2H), 6.59 (s, 2H), 2.70 (s, 3H), 2.20 (s, 6H), 2.13 (s, 6H), 2.05 (s, 6H), 1.73 (s, 6H), 1.39 (s, 3H), 1.22 (s, 9H) ppm. ^{19}F NMR (376 MHz, CD_2Cl_2) δ -62.81 ppm. ^{13}C NMR (101 MHz, CD_2Cl_2) δ 300.1, 193.7, 162.3 (q, $^1J_{BC}$ = 49.9 Hz), 153.4, 149.6, 141.3, 139.9, 138.9, 136.0, 135.4, 135.4, 133.9, 130.7, 130.0, 129.5, 129.5 (qq, $^2J_{CF}$ = 31.5 Hz, $^3J_{CB}$ = 2.9 Hz), 129.1, 128.4, 127.7, 127.2, 126.1, 125.5, 125.2 (q, $^1J_{CF}$ = 272.6 Hz), 118.1, 55.2, 33.4, 30.5, 30.1, 27.2, 21.3, 20.6, 19.6, 19. ppm. Elemental analysis (%) calcd. for $C_{76}H_{66}BBrF_{24}N_4W$: C 51.69, H 3.77, N 3.17; found: C 51.90, H 3.904, N 3.19.

[W(N-2-$CF_3C_6H_4$)($CHCMe_2Ph$)Br(IMes)(pivCN)$^+$ B(Ar^F)$_4^-$] (W-26). A solution of W(N-2-$CF_3C_6H_4$)($CHCMe_2Ph$)Br_2(IMes) (**W-21**, 700 mg, 0.96 mmol) in CH_2Cl_2 (10 mL) was added to NaB(Ar^F)$_4$ in CH_2Cl_2 (5 mL) and pivalonitrile (500 µl). The reaction was stirred at room temperature for three hours and the solvent was removed *in vacuo*. The residue was stirred with pentane until a suspension formed. The pentane was decanted and the solid dried under reduced pressure. The solid was redissolved in CH_2Cl_2 and filtered through a pad of celite. Pentane and diethyl ether were added and the solution was stored at -35 °C overnight during which time the product crystallized. Yield: 1.16 g (86 %). ^{1}H NMR (400 MHz, $CDCl_3$) δ 10.11 (s, 1H), 7.71 (s, 8H), 7.52 (s, 4H), 7.51 – 7.47 (m, 1H), 7.42 (d, *J* = 7.9 Hz, 1H), 7.33 (t, *J* = 7.6 Hz, 1H), 7.31 – 7.19 (m, 4H), 7.08 (d, *J* = 6.9 Hz, 2H), 6.66 (d, *J* = 1.8 Hz, 2H), 6.49 (s, 2H), 2.12 (s, 6H), 2.06 (s, 6H), 2.00 (s, 6H), 1.64 (s, 3H), 1.52 (s, 3H), 1.18 (s, 9H) ppm. ^{19}F NMR (376 MHz, $CDCl_3$) δ -57.57 (3F), -62.41 (24F) ppm. ^{13}C NMR (101 MHz, $CDCl_3$) δ 304.5, 192.8, 161.9 (q, $^1J_{BC}$ = 49.8 Hz), 149.8, 147.7, 140.6, 139.4, 135.0, 134.9, 134.8, 134.6, 131.8, 130.4, 129.6, 129.0 (qq, $^2J_{CF}$ = 31.5 Hz, $^3J_{CB}$ = 2.7 Hz), 128.7, 128.6, 127.0, 126.9 (q, *J* = 5.1 Hz), 125.7, 125.6, 124.7 (q, $^1J_{CF}$ = 272.3 Hz), 117.6, 55.5, 33.2, 30.9, 30.0, 26.7, 21.0, 19.3, 19.2 ppm. Elemental analysis (%) calcd. for $C_{75}H_{61}BBrF_{27}N_4W$: C 49.88, H 3.40, N 3.10; found: C 50.05, H 3.495, N 3.12.

[W(N-2,6-$Cl_2C_6H_3$)($CHCMe_2Ph$)Cl(IMes)(pivCN)$^+$ B(Ar^F)$_4^-$] (W-27). A solution of W(N-2,6-$Cl_2C_6H_3$)($CHCMe_2Ph$)Cl_2(IMes) (**W-22**, 500 mg, 0.587 mmol) in CH_2Cl_2 (4 mL) was added to a suspension of NaB(Ar^F)$_4$ (521 mg, 0.587 mmol) in CH_2Cl_2 (8 mL) and pivalonitrile (100 µl). The suspension was stirred at room temperature for three hours. The solvent was removed *in vacuo*

and the residue was coevaporated with pentane twice. The residue was extracted with CH_2Cl_2, filtered through a pad of celite and the solvent removed again. The residue was triturated with pentane (25 mL) and the resulting suspension was filtered. The solid was washed with pentane to yield the product as a yellow solid. Yield: 975 mg (94 %). ^{1}H NMR (400 MHz, $CDCl_3$) δ 10.44 (s, 1H), 7.73 (s, 8H), 7.54 (s, 4H), 7.33 – 7.16 (m, 74H), 7.09 (d, J = 7.1 Hz, 2H), 6.98 (t, J = 8.1 Hz, 1H), 6.69 (s, 4H), 2.13 (s, 6H), 2.11 (s, 6H), 1.99 (s, 6H), 1.77 (s, 3H), 1.64 (s, 3H), 1.17 (s, 9H) ppm. ^{19}F NMR (376 MHz, $CDCl_3$) δ -62.42 ppm ^{13}C NMR (101 MHz, $CDCl_3$) δ 304.6, 193.6, 161.9 (q, $^1J_{BC}$ = 49.8 Hz), 149.1, 149.0, 140.7, 135.6, 135.0, 134.8, 130.3, 129.4, 129.0, 129.0 (qq, $^2J_{CF}$ = 31.5 Hz, $^3J_{CB}$ =2.9 Hz), 128.6, 126.9, 125.4, 125.2, 124.7 (q, $^1J_{CF}$ = 272.5 Hz), 117.6, 54.1, 34.2, 29.9, 29.8, 26.9, 21.1, 19.0, 18.9 ppm. Elemental analysis (%) calcd. for $C_{64}H_{60}BCl_3F_{24}N_4W$: C 50.44, H 3.43, N 3.18; found: C 50.44, H 3.597, N 3.15.

[W(N-3,5-Me$_2$C$_6$H$_3$)(CHCMe$_2$Ph)Cl(IMes)(pivCN)$^+$ B(Ar^F)$_4^-$] (W-28). A solution of W(N-3,5-Me$_2$C$_6$H$_3$)(CHCMe$_2$Ph)Cl$_2$(IMes) (**W-23**, 288 mg, 0.355 mmol) in CH_2Cl_2 (5 mL) was added to a suspension of NaB(Ar^F)$_4$ (315 mg, 0.355 mmol) in CH_2Cl_2 (4 mL) and a few drops of pivalonitrile. The mixture was stirred for three hours at room temperature, the solvent was removed *in vacuo* and the residue was triturated with pentane to yield a yellow suspension. The pentane was decanted and the solid was dried. The solid was dissolved in CH_2Cl_2 and the solution was filtered through a pad of celite. The solvent was removed again and the residue triturated with pentane. The pentane was decanted, yielding the product as a yellow solid. Yield: 544 mg (89 %). ^{1}H NMR (400 MHz, $CDCl_3$) δ 10.70 (s, 1H), 7.73 (s, 8H), 7.54 (s, 4H), 7.30 – 7.20 (m, 3H), 7.17 (s, 2H), 7.15 – 7.11 (m, 2H), 6.83 (s, 1H), 6.62 (s, 4H), 6.29 (s, 2H), 2.22 (s, 6H), 2.12 (s, 6H), 2.10 (s, 6H), 1.93 (s, 6H), 1.62 (s, 3H), 1.59 (s, 3H), 1.13 (s, 9H) ppm. ^{19}F NMR (376 MHz, $CDCl_3$) δ -62.37 ppm. ^{13}C NMR (101 MHz, $CDCl_3$) δ 303.0, 192.9, 161.8 (q, $^1J_{BC}$ = 49.8 Hz), 153.2, 148.4, 140.5, 138.0, 135.9, 135.2, 135.0, 134.8, 134.1, 131.1, 129.9, 129.8, 129.0 (qq, $^2J_{CF}$ = 31.5 Hz, $^3J_{CB}$ = 2.9 Hz), 128.6, 126.8, 125.8, 125.0, 124.7, 124.7 (q, $^1J_{CF}$ = 272.5 Hz), 117.6, 53.7, 33.8, 31.0, 29.6, 27.0, 21.0, 18.6, 18.6 ppm. Elemental analysis (%) calcd. for $C_{76}H_{66}BClF_{24}N_4W$: C 53.03, H 3.86, N 3.25; found: C 53.14, H 3.940, N 3.33.

[W(N-2-*t*BuC$_6$H$_4$)(CHCMe$_2$Ph)Cl(IMes)(pivCN)$^+$ B(Ar^F)$_4^-$] (W-29). A solution of NaB(Ar^F)$_4$ (634 mg, 0.72 mmol, 1 equiv.) in CH_2Cl_2 (4 mL) and pivalonitrile (200 µL) was added to a solution of W(N-2-*t*BuC$_6$H$_4$)(CHCMe$_2$Ph)Cl$_2$(IMes) (**W-24**, 600 mg, 0.72 mmol) in CH_2Cl_2 (4 mL). After stirring the reaction at room temperature for two hours, the solvent was removed under reduced pressure. The residue was stirred with pentane to yield a yellow suspension. The pentane was decanted and the solid was dried *in vacuo*. The solid was dissolved in CH_2Cl_2 and filtered through a pad of celite. The solvent was removed again and the residue stirred with pentane until a solid formed. The pentane was decanted and the solid was dried to yield the product as a yellow solid. Yield: 1136 mg (91 %). ^{1}H NMR (400 MHz, CD_2Cl_2) δ 10.17 (s, 1H), 7.76 (s, 8H), 7.59 (s, 4H), 7.35 – 7.08 (m, 11H), 6.83 (s, 2H), 6.63 (s, 2H), 2.20 (s, 6H), 2.13 (s, 6H), 2.06 (s, 6H), 1.71 (s, 3H), 1.30 (s, 3H), 1.22 (s, 9H), 1.20 (s, 9H) ppm. ^{19}F NMR (376 MHz, CD_2Cl_2) δ -62.79 ppm. ^{13}C NMR (101 MHz, CD_2Cl_2) δ 302.0, 192.3, 162.4 (q, $^1J_{BC}$ = 49.8 Hz), 153.5, 149.0, 145.9, 140.9, 136.0, 135.5, 135.4, 135.1, 130.8, 130.2, 129.5 (qq, $^2J_{CF}$ = 31.5 Hz, $^3J_{CB}$ = 2.9 Hz), 129.2, 129.0, 127.2, 126.6, 126.3, 126.0, 125.2 (q, $^1J_{CF}$ = 272.4 Hz), 118.1, 56.8,

36.2, 33.0, 31.5, 31.2, 30.5, 27.3, 21.2, 19.6, 19.5 ppm. Elemental analysis (%) calcd. for $C_{78}H_{70}BClF_{24}N_4W$: C 53.55, H 4.03, N 3.20; found: C 53.50, H 4.060, N 3.21.

[W(N-2,6-^{i}Pr$_2$C$_6$H$_3$)(CHCMe$_2$Ph)(OC$_6$F$_5$)(IMes)(pivCN)$^+$ B(Ar^F)$_4^-$] (W-30). A solution of [W(N-2,6-^{i}Pr$_2$C$_6$H$_3$)(CHCMe$_2$Ph)Br(IMes)$^+$ B(Ar^F)$_4^-$] (**W-08**, 150 mg, 0.086 mmol) in CH_2Cl_2 (5 mL) was added to a suspension of F_5C_6OLi (16.4 mg, 0.086 mmol) in CH_2Cl_2 (3 mL). After stirring at room temperature overnight, a sample was withdrawn from the solution and conversion was checked via ^{1}H-NMR. The suspension was filtered through celite and pivalonitrile (50 µl) was added. After 15 minutes of stirring, the solvent was removed *in vacuo* and the residue was triturated with pentane to yield the product as a dark yellow solid. Yield: 144 mg (90 %). Besides the alkylidene signals, only the NMR data for the *anti*-isomer are listed below. ^{1}H NMR (400 MHz, $CDCl_3$) δ 13.12 (s, 1H, W=CH, *anti*), 10.98 (s, 1H, W=CH, *syn*), 7.70 (s, 8H), 7.51 (s, 4H), 7.36 – 7.05 (m, 10H), 6.76 (s, 2H), 6.66 (s, 2H), 3.49 (d, J = 7.5 Hz, 1H), 3.00 (d, J = 6.5 Hz, 1H), 2.19 (s, 6H), 1.88 (s, 3H), 1.85 (s, 6H), 1.77 (s, 5H), 1.68 (s, 3H), 1.29 – 1.02 (m, 12H), 0.84 (s, 9H) ppm.^{19}F NMR (376 MHz, $CDCl_3$) δ -62.44 (24F), -155.79 (1F), -162.13 (1F), -163.37 (1F), -165.74 (1F), -167.68 – -168.17 (m, 1F) ppm. ^{13}C NMR (101 MHz, $CDCl_3$) δ 314.66, 189.22, 161.84 (d, $^1J_{BC}$ = 49.8 Hz), 152.04, 149.62, 148.59, 147.76, 139.79, 135.84, 135.00, 134.93, 133.60, 129.80, 129.39, 129.17, 128.84 (qq, J = 28.2, 3.4 Hz), 128.60, 126.90, 126.43, 125.68, 124.69 (q, $^1J_{CF}$ = 272.5 Hz), 123.49, 122.54, 117.56, 51.68, 33.26, 31.22, 29.61, 29.37, 28.97, 26.76, 25.55, 23.00, 21.90, 21.00, 18.14, 17.99 ppm. Elemental analysis (%) calcd. for $C_{86}H_{74}BF_{29}N_4OW$: C 53.65, H 3.87, N 2.91; found: C 53.60, H 3.996, N 2.98.

[W(N-2,6-Me$_2$C$_6$H$_3$)(CHCMe$_2$Ph)(OC$_6$F$_5$)(IMes)(pivCN)]$^+$ B(Ar^F)$_4$]$^-$ (W-31). A solution of [W(N-2,6-Me$_2$C$_6$H$_3$)(CHCMe$_2$Ph)Br(IMes)(pivCN)$^+$ B(Ar^F)$_4$]$^-$ (**W-25**, 115 mg, 0.065 mmol) in CH_2Cl_2 (2 mL) was added to a suspension of F_5C_6OLi (12.4 mg, 0.065 mmol) in CH_2Cl_2 (1 mL). The suspension was stirred at room temperature for three hours and filtered through a pad of celite. The solvent was removed *in vacuo* and the residue was triturated with pentane (6 mL) which led to the formation of a yellow suspension. The solid was recrystallized from a mixture of CH_2Cl_2, diethyl ether and pentane. The supernatant solution was decanted and the solid was dried *in vacuo* to yield the product as a yellow solid. Yield: 100 mg (82 %). ^{1}H NMR (400 MHz, $CDCl_3$) δ 11.17 (s, 1H), 7.72 (s, 8H), 7.52 (s, 4H), 7.32 – 7.17 (m, 3H), 7.21 (s, 2H), 7.09 – 6.91 (m, 5H), 6.73 (s, 2H), 6.69 (s, 2H), 2.23 (s, 6H), 2.17 (s, 6H), 1.94 (s, 6H), 1.93 (s, 6H), 1.67 (s, 3H), 1.52 (s, 3H), 0.88 (s, 9H) ppm. ^{19}F NMR (376 MHz, $CDCl_3$) δ -62.44 (24F), -160.55 (2F), -163.90 (2F), -167.85 (1F) ppm. ^{13}C NMR (101 MHz, $CDCl_3$) δ 296.7, 191.2, 161.8 (q, $^1J_{BC}$ = 49.8 Hz), 152.5, 149.2, 141.1, 135.3, 134.9, 134.5, 134.5, 130.0, 129.5, 129.0 (qq, $^2J_{CF}$ = 31.5 Hz, $^3J_{CB}$ = 2.9 Hz), 128.7, 128.7, 127.7, 126.9, 125.2, 125.1, 124.7 (q, $^1J_{CF}$ = 272.5 Hz), 117.6, 53.9, 33.9, 30.5, 29.7, 26.7, 21.1, 20.0, 18.2, 17.8 ppm. Elemental analysis (%) calcd. for $C_{82}H_{66}BF_{29}N_4OW$: C 52.70, H 3.56, N 3,00; found: C 52.71, H 3.601, N 2.96.

[W(N-2-CF$_3$C$_6$H$_4$)(CHCMe$_2$Ph)(OC$_6$F$_5$)(IMes)(pivCN)$^+$ B(Ar^F)$_4^-$] (W-32). A solution of [W(N-2-CF$_3$C$_6$H$_4$)(CHCMe$_2$Ph)Br(IMes)(pivCN)$^+$ B(Ar^F)$_4^-$] (**W-26**, 300 mg, 0.17 mmol) in CH_2Cl_2 (4 mL) was added to a suspension of $LiOC_6F_5$ (40.1 mg, 0.21 mmol, 1.27 equiv.) in CH_2Cl_2 (3 mL). The suspension was stirred at room temperature overnight and then filtered through a pad of celite.

The solvent was removed under reduced pressure and the product was crystallized from a mixture of CH_2Cl_2, diethyl ether and pentane to yield the product as a yellow microcrystalline solid. Yield: 230 mg (73 %). ^{1}H NMR (400 MHz, $CDCl_3$) δ 11.59 (s, 1H), 7.72 (s, 8H), 7.52 (s, 4H), 7.47 (t, J = 7.8 Hz, 1H), 7.41 (d, J = 7.9 Hz, 1H), 7.31 (t, J = 7.3 Hz, 2H), 7.27 – 7.20 (m, 2H), 7.18 (s, 2H), 7.16 (d, J = 7.2 Hz, 2H), 7.13 (d, J = 8.1 Hz, 1H), 6.64 (s, 2H), 6.53 (s, 2H), 2.13 (s, 6H), 1.93 (s, 6H), 1.86 (s, 6H), 1.74 (s, 3H), 1.66 (s, 3H), 0.88 (s, 9H) ppm. ^{19}F NMR (376 MHz, $CDCl_3$) δ -57.54 (3F), -62.45 (24F), -160.46 (2F), -163.95 (2F), -167.44 (1F) ppm. ^{13}C NMR (101 MHz, $CDCl_3$) δ 301.4, 190.9, 161.8 (q, $^1J_{BC}$ = 49.8 Hz), 149.7, 148.2, 140.6, 136.7, 134.9, 134.8, 134.6, 134.3, 131.7, 129.9, 129.4, 129.0 (qq, $^2J_{CF}$ = 31.5 Hz, $^3J_{CB}$ = 2.9 Hz), 128.8, 128.4, 128.3, 127.4, 127.2, 127.0, 126.9 (q, J = 4.7 Hz), 125.6, 125.3, 125.1, 124.7 (q, $^1J_{CF}$ = 272.7 Hz), 122.3, 117.6, 33.9, 31.6, 29.5, 26.5, 21.0, 18.0, 17.8 ppm. Elemental analysis (%) calcd. for $C_{81}H_{61}BF_{32}N_4OW$: C 50.96, H3.22, N 2.93; found: C 50.83, H 3.344, N 2.98.

[W(N-2,6-$Cl_2C_6H_3$)($CHCMe_2Ph$)(OC_6F_5)(IMes)(pivCN)$^+$ B(Ar^F)$_4^-$] (W-33). A solution of [W(N-2,6-$Cl_2C_6H_3$)($CHCMe_2Ph$)Cl(IMes)(pivCN)$^+$ B(Ar^F)$_4^-$] (**W-27**, 200 mg, 0.113 mmol) in CH_2Cl_2 (4 mL) was added to a suspension of $LiOC_6F_5$ (21.6 mg, 0.113 mmol) in CH_2Cl_2 (4 mL). The suspension was stirred for 24 hours at room temperature followed by filtration through a pad of celite. The solvent was removed *in vacuo* and the residue was triturated with pentane to give a yellow solid. The pentane was decanted and the solid was recrystallized from a mixture of CH_2Cl_2 and pentane. After decanting the supernatant solution, the product was obtained as orange crystals which were dried *in vacuo*. Yield: 144 mg (66 %). ^{1}H NMR (400 MHz, $CDCl_3$) δ 11.59 (s, 1H), 7.71 (s, 8H), 7.52 (s, 4H), 7.35 (t, J = 7.4 Hz, 2H), 7.30 – 7.26 (m, 1H), 7.24 (s, 2H), 7.21 (dd, J = 7.6, 4.1 Hz, 4H), 6.90 (t, J = 8.1 Hz, 1H), 6.71 (s, 2H), 6.49 (s, 2H), 2.07 (s, 6H), 2.03 (s, 6H), 2.01 (s, 3H), 1.83 (s, 6H), 1.72 (s, 3H), 0.87 (s, 9H) ppm. ^{19}F NMR (376 MHz, $CDCl_3$) δ -62.43 (24F), -160.47 (2F), -163.56 (2F), -166.49 (1F) ppm. ^{13}C NMR (101 MHz, $CDCl_3$) δ 302.5, 191.9, 161.8 (q, $^1J_{BC}$ = 49.8 Hz), 149.5, 148.3, 140.6, 136.9, 135.4, 134.9, 134.5, 134.2, 129.9, 129.3, 129.0 (qq, $^2J_{CF}$ = 31.6 Hz, $^3J_{CB}$ = 3.0 Hz), 128.7, 127.6, 126.9, 125.5, 125.0, 124.7 (q, $^1J_{CF}$ = 272.6 Hz), 117.6, 53.9, 35.8, 30.3, 29.5, 26.6, 21.1, 18.5, 18.1 ppm. Elemental analysis (%) calcd. for $C_{80}H_{60}BCl_2F_{29}N_4OW$: C 50.31, H 3.17, N 2.93; found: C 50.33, H 3.271, N 2.90.

[W(N-3,5-$Me_2C_6H_3$)($CHCMe_2Ph$)(OC_6F_5)(IMes)(pivCN)$^+$ B(Ar^F)$_4^-$] (W-34). A solution of [W(N-3,5-$Me_2C_6H_3$)($CHCMe_2Ph$)Cl(IMes)(pivCN)$^+$ B(Ar^F)$_4^-$] (**W-28**, 200 mg, 0.12 mmol) in CH_2Cl_2 (4 mL) was added to a suspension of $LiOC_6F_5$ (22.1 mg, 0.12 mmol, 1 equiv.) in CH_2Cl_2 (4 mL). The reaction was stirred overnight, filtered through a pad of celite and the solvent was reduced *in vacuo*. The residue was stirred with pentane to give an orange oil. The biphasic mixture was left to stand at room temperature overnight during which time the oil crystallized. The pentane was decanted and the crystals dried under reduced pressure to give the product as an orange solid. Yield: 205 mg (94 %). ^{1}H NMR (400 MHz, $CDCl_3$) δ 11.60 (s, 1H), 7.69 (s, 8H), 7.50 (s, 4H), 7.27 – 7.17 (m, 3H), 7.16 (s, 2H), 7.12 (d, J = 7.1 Hz, 2H), 6.77 (s, 1H), 6.67 (s, 2H), 6.64 (s, 2H), 6.27 (s, 2H), 2.18 (s, 6H), 2.13 (s, 6H), 1.96 (s, 6H), 1.82 (s, 6H), 1.62 (s, 3H), 1.57 (s, 3H), 0.84 (s, 9H) ppm. ^{19}F NMR (376 MHz, $CDCl_3$) δ -62.44 (24F), -160.93 (2F), -163.93 (2F), -168.00 (1F) ppm. ^{13}C NMR (101 MHz, $CDCl_3$) δ 299.6, 191.1, 161.8 (q, $^1J_{BC}$ = 49.9 Hz), 153.3, 148.8, 140.7, 137.9, 135.2, 135.0, 134.7, 133.9, 130.8, 129.8, 129.7, 129.0 (qq,

$^2J_{CF}$ = 31.5 Hz, $^3J_{CB}$ = 2.9 Hz), 128.6, 126.7, 125.7, 125.1, 124.9, 124.7 (q, $^1J_{CF}$ = 272.5 Hz), 117.6, 53.2, 33.8, 31.4, 29.3, 26.7, 21.1, 21.0, 17.9, 17.7 ppm. Elemental analysis (%) calcd. for $C_{82}H_{66}BF_{29}N_4OW$: C 52.70, H 3.56, N 3.00; found: C 52.72, H 3.866, N 2.90.

[W(N-2-*t*BuC$_6$H$_4$)(CHCMe$_2$Ph)(OC$_6$F$_5$)(IMes)(pivCN)$^+$ B(Ar^F)$_4^-$] (W-35). A solution of [W(N-2-*t*BuC$_6$H$_4$)(CHCMe$_2$Ph)Cl(IMes)(pivCN)$^+$ B(Ar^F)$_4^-$] (**W-29,** 250 mg, 0.14 mmol) in CH_2Cl_2 (4 mL) was added to a suspension of $LiOC_6F_5$ (27.2 mg, 0.14 mmol, 1 equiv.) in CH_2Cl_2 (2 mL). The reaction was stirred at room temperature for 48 hours, filtered through a pad of celite and the solvent was removed under reduced pressure. The residue was recrystallized from a mixture of CH_2Cl_2, diethyl ether and pentane. Yield:148 mg (55 %). In $CDCl_3$, the compound exists as a mixture of *syn*- and *anti*-isomer. *Syn*-isomer: ^{1}H NMR (400 MHz, $CDCl_3$) δ 11.53 (s, 1H), 7.72 (s, 8H), 7.52 (s, 4H), 7.43 – 6.93 (m, 11H), 6.77 (s, 2H), 6.73 (s, 2H), 2.22 (s, 6H), 1.99 (s, 6H), 1.89 (s, 6H), 1.65 (s, 3H), 1.26 (s, 9H), 1.21 (s, 3H), 0.85 (s, 9H) ppm. ^{19}F NMR (376 MHz, $CDCl_3$) δ -62.43 (24F), -160.41 (2F), -164.00 (2F), -168.39 (1F) ppm. *Anti*-isomer: ^{1}H NMR (400 MHz, $CDCl_3$) δ12.97 (s, 1H), 7.72 (s, 8H), 7.52 (s, 4H), 7.43 – 6.95 (m, 11H), 6.83 (s, 2H), 6.75 (s, 2H), 2.22 (s, 6H), 1.96 (s, 6H), 1.93 (s, 6H), 1.76 (s, 3H), 1.44 (s, 3H), 1.36 (s, 9H), 0.66 (s, 9H) ppm. ^{19}F NMR (376 MHz, $CDCl_3$) δ -62.43 (24F), -159.43 (2F), -163.74 (2F), -167.73 (1F) ppm. Both isomers: ^{13}C NMR (101 MHz, $CDCl_3$) δ 314.0, 299.2, 189.3, 188.4, 161.9 (q, $^1J_{BC}$ = 49.9 Hz), 153.5, 153.2, 149.4, 148.5, 145.3, 142.7, 140.9, 140.1, 135.5, 135.3, 135.2, 134.9, 134.6, 134.5, 134.0, 130.1, 130.0, 129.6, 129.0 (qq, J = 31.5, 2.9 Hz), 128.7, 128.7, 128.4, 126.9, 126.7, 126.6, 126.5, 126.4, 125.7, 125.7, 125.7, 125.6, 124.7 (q, $^1J_{CF}$ = 272.5 Hz), 117.6, 56.0, 50.9, 35.7, 35.5, 32.4, 31.6, 31.3, 31.2, 30.7, 30.5, 29.7, 29.2, 26.7, 26.5, 21.0, 20.9, 18.4, 18.3, 18.0, 17.8 ppm. Elemental analysis (%) calcd. for $C_{84}H_{70}BF_{29}N_4OW$: C 53.18, H 3.72, N 2.95; found: C 53.27, H 3.752, N 3.01.

[W(N-2,6-^{i}Pr$_2$C$_6$H$_3$)(CHCMe$_2$Ph)(OTf)(IMes)(pivCN)$_2^+$ B(Ar^F)$_4^-$] (W-36). A solution of [W(N-2,6-^{i}Pr$_2$C$_6$H$_3$)(CHCMe$_2$Ph)Br(IMes)$^+$ B(Ar^F)$_4$]$^-$ (**W-08**, 100 mg, 0.056 mmol) in CH_2Cl_2 (3 mL) was added to a suspension of AgOTf (14.4 mg, 0.056 mmol) in CH_2Cl_2 (3 mL). The mixture was stirred at room temperature for two hours, during that time a yellow solid precipitated and the solution turned orange. The suspension was filtered through a pad of celite and the solvent was removed *in vacuo*. The residue was stirred with pentane (10 mL), the pentane was decanted from the red oil and the oil was dissolved in CH_2Cl_2 (2 mL). Four drops of pivalonitrile were added to the solution. The solution was stirred for five minutes at room temperature and then the solvent was removed *in vacuo*. The residue was triturated with pentane (5 mL) which led to the formation of a yellow suspension. The solid was recrystallized at -35 °C from a mixture of $CDCl_3$, CH_2Cl_2, diethyl ether and pentane. Yield: 66 mg (60 %). ^{1}H NMR (400 MHz, $CDCl_3$) δ 11.26 (s, 1H), 7.72 (s, 8H), 7.53 (s, 4H), 7.30 – 7.15 (m, 8H), 7.01 (s, 2H), 6.93 (d, *J* = 7.2 Hz, 2H), 6.88 (s, 2H), 3.86 (brs, 1H), 3.12 (brs, 1H), 2.33 (s, 6H), 2.14 (s, 6H), 1.87 (s, 6H), 1.78 (s, 3H), 1.37 (s, 9H), 1.26 (s, 3H), 1.15 (s, 9H), 1.10 (d, *J* = 6.6 Hz, 6H) ppm. ^{19}F NMR (376 MHz, $CDCl_3$) δ -62.40 (s, 24F), -75.80 (s, 3F) ppm. ^{13}C NMR (101 MHz, $CDCl_3$) δ 308.1, 189.3, 161.8 (q, $^1J_{BC}$ = 49.9 Hz), 150.1, 149.8, 141.2, 140.3, 136.0, 135.8, 135.6, 134.9, 130.4, 130.2, 130.1, 129.0 (qq, $^2J_{CF}$ = 31.6 Hz, $^3J_{CB}$ = 3.0 Hz), 128.8, 127.0, 126.6, 125.0, 124.7 (q, $^1J_{CF}$ = 272.5 Hz), 117.6, 55.7, 32.1, 30.3, 29.1, 27.9, 26.9, 21.1, 18.6, 18.5 ppm. Elemental analysis (%) calcd. for $C_{86}H_{83}BF_{27}N_5O_3SW$: C 52.32, H 4.24, N 3.55; found: C 52.37, H 4.348, N 3.57.

[W(N-2,6-$Me_2C_6H_3$)($CHCMe_2Ph$)(OTf)(IMes)(pivCN)$^+$ $B(Ar^F)_4^-$] (W-37). A solution of [W(N-2,6-$Me_2C_6H_3$)($CHCMe_2Ph$)Br(IMes)(pivCN)$^+$ $B(Ar^F)_4^-$] (**W-25**, 100 mg, 0.057 mmol) in CH_2Cl_2 (2 mL) was added to a suspension of AgOTf (14.6 mg, 0.057 mmol) in CH_2Cl_2 (1 mL). The suspension was stirred at room temperature for three hours during which time a yellow solid formed. The reaction was filtered through a pad of celite and the solvent was removed *in vacuo*. The residue was redissolved in CH_2Cl_2 (1 mL) and cooled to -35 °C for two hours. The solution was filtered once again and crystallized from a mixture of CH_2Cl_2, diethyl ether and pentane. Yield: 75 mg (72 %). ^{1}H NMR (400 MHz, $CDCl_3$) δ 10.70 (s, 1H), 7.72 (s, 8H), 7.52 (s, 4H), 7.27 (s, 2H), 7.25 – 7.13 (m, 3H), 7.07 – 6.91 (m, 7H), 6.65 (s, 2H), 2.53 (s, 3H), 2.26 (s, 6H), 2.10 (s, 6H), 1.91 (s, 6H), 1.76 (s, 3H), 1.63 (s, 3H), 1.24 (s, 9H), 1.23 (s, 3H) ppm. ^{19}F NMR (376 MHz, $CDCl_3$) δ -62.40 (24F), -74.74 (3F) ppm ^{13}C NMR (101 MHz, $CDCl_3$) δ 297.8, 191.2, 161.8 (q, $^1J_{BC}$ = 49.8 Hz), 151.8, 147.8, 141.6, 140.8, 139.1, 135.6, 134.9, 134.7, 134.6, 134.0, 130.3, 129.8, 129.8, 129.0 (qq, $^2J_{CF}$ = 31.5 Hz, $^3J_{CB}$ = 2.9 Hz), 128.8, 128.2, 127.6, 127.3, 124.9, 124.7 (q, $^1J_{CF}$ = 272.5 Hz), 117.6, 54.8, 31.4, 30.3, 29.6, 26.7, 21.1, 19.8, 18.2, 18.0 ppm. Elemental analysis (%) calcd. for $C_{77}H_{66}BF_{27}N_4O_3SW$: C 50.40, H 3.63, N 3.05; found: C 50.39, H 3.764, N 3.08.

[W(N-2-$CF_3C_6H_4$)($CHCMe_2Ph$)(OTf)(IMes)(pivCN)$^+$ $B(Ar^F)_4^-$] (W-38). A solution of [W(N-2-$CF_3C_6H_4$)($CHCMe_2Ph$)Br(IMes)(pivCN)$^+$ $B(Ar^F)_4^-$] (**W-26**, 300 mg, 0.17 mmol) in CH_2Cl_2 (4 mL) was added to a suspension of AgOTf (42.7 mg, 0.17 mmol, 1 equiv.) in CH_2Cl_2 (3 mL). The suspension was stirred at room temperature for two hours and then filtered through a pad of celite. The solvent was removed under reduced pressure and the residue was stirred with pentane until a yellow solid formed. The pentane was decanted and the solid was recrystallized from a mixture of CH_2Cl_2 and pentane. The solid was obtained as yellow crystals. Yield: 265 mg (85 %). ^{1}H NMR (400 MHz, $CDCl_3$) δ 11.16 (s, 1H), 7.73 (s, 8H), 7.53 (s, 4H), 7.52 – 7.48 (m, 2H), 7.35 (t, J = 7.7 Hz, 1H), 7.24 (d, J = 10.6 Hz, 4H), 7.19 – 7.15 (m, 1H), 7.09 (d, J = 8.1 Hz, 1H), 7.07 – 7.02 (m, 2H), 6.88 (s, 2H), 6.62 (s, 2H), 2.21 (s, 6H), 2.07 (s, 6H), 1.93 (s, 6H), 1.60 (s, 3H), 1.21 (s, 3H), 1.20 (s, 9H) ppm. ^{19}F NMR (376 MHz, $CDCl_3$) δ -58.92 (3F), -62.42 (24F), -74.72 (3F) ppm. ^{13}C NMR (101 MHz, $CDCl_3$) δ 301.1, 190.2, 161.9 (q, $^1J_{BC}$ = 49.8 Hz), 149.6, 146.8, 141.4, 140.4, 135.1, 134.9, 134.2, 133.9, 133.0, 132.0, 130.6, 130.5, 129.8, 129.0 (qq, $^2J_{CF}$ = 31.5 Hz, $^3J_{CB}$ = 3.0 Hz), 128.8, 127.3, 127.0, 126.7, 126.6 (q, J = 4.8 Hz), 126.2, 125.6, 124.7 (q, $^1J_{CF}$ = 272.5 Hz), 124.4, 122.5, 121.7, 121.4, 121.1, 117.6, 55.7, 31.7, 30.5, 30.2, 26.4, 20.9, 18.3, 18.2 ppm. Elemental analysis (%) calcd. for $C_{76}H_{61}BF_{30}N_4O_3SW$: C 48.68, H 3.28, N 2.99; found: C 48.58, H 3.657, N 3.01.

[W(N-2,6-$Cl_2C_6H_3$)($CHCMe_2Ph$)(OTf)(IMes)(pivCN)$^+$ $B(Ar^F)_4^-$] (W-39). A solution of [W(N-2,6-$Cl_2C_6H_3$)($CHCMe_2Ph$)Cl(IMes)(pivCN)$^+$ $B(Ar^F)_4^-$] (**W-27**, 200 mg, 0.113 mmol) in CH_2Cl_2 (4 mL) was added to a suspension of AgOTf (29.2 mg, 0.113 mmol) in CH_2Cl_2 (4 mL). The mixture was stirred at room temperature for two hours during which time a yellow solid precipitated. The suspension was filtered and the solvent was removed *in vacuo*. The residue was triturated with pentane to give the product as a yellow solid. Yield: 200 mg (94 %). ^{1}H NMR (400 MHz, $CDCl_3$) δ 10.78 (s, 1H), 7.72 (s, 8H), 7.52 (s, 4H), 7.32 – 7.15 (m, 7H), 7.06 – 6.95 (m, 3H), 6.85 (s, 2H),

6.68 (s, 2H), 2.17 (s, 6H), 2.04 (s, 12H), 1.76 (s, 3H), 1.45 (s, 3H), 1.19 (s, 9H) ppm. ^{19}F NMR (376 MHz, $CDCl_3$) δ -62.40 (24F), - 74.65 (3F) ppm. ^{13}C NMR (101 MHz, $CDCl_3$) δ 302.5, 192.0, 161.8 (q, $^{1}J_{BC}$ = 49.8 Hz), 148.0, 147.9, 141.4, 137.8, 135.5, 134.9, 134.7, 134.2, 130.6, 130.0, 129.9, 129.5, 129.0 (qq, $^{2}J_{CF}$ = 31.5 Hz, $^{3}J_{CB}$ = 2.9 Hz), 128.8, 127.5, 127.3, 125.9, 125.1, 124.7 (q, $^{1}J_{CF}$ = 272.5 Hz), 117.6, 54.5, 32.6, 30.1, 29.5, 26.6, 21.1, 18.4, 18.3 ppm. Elemental analysis (%) calcd. for $C_{75}H_{60}BCl_2F_{27}N_4O_3SW$: C 48.02, H 3.22, N 2.99; found: C 48.23, H 3.271, N 3.05.

[W(N-3,5-Me$_2$C$_6$H$_3$)(CHCMe$_2$Ph)(OTf)(IMes)(pivCN)$^+$ B(Ar^F)$_4^-$] (W-40). A solution of [W(N-3,5-Me$_2$C$_6$H$_3$)(CHCMe$_2$Ph)Cl(IMes)(pivCN)$^+$ B(Ar^F)$_4^-$] (**W-28**, 200 mg, 0.12 mmol) in CH_2Cl_2 (4 mL) was added to a suspension of AgOTf (29.9 mg, 0.12 mmol, 1 equiv.) in CH_2Cl_2 (4 mL). The reaction was stirred at room temperature for three hours. The suspension was filtered through a pad of celite and the solvent was removed *in vacuo*. The residue was stirred with pentane to yield a red oil. The pentane was decanted and the oil was dissolved in Novec 7100. The solution was stored at -35 °C overnight during which time a small amount of solid precipitated. The cold solution was filtered and the solvent was removed under reduced pressure. The residue was stirred with pentane until a suspension formed. The pentane was decanted and the solid was dried *in vacuo* to yield the product as a yellow solid. Yield:165 mg (77 %). ^{1}H NMR (400 MHz, $CDCl_3$) δ 11.27 (s, 1H), 7.74 (s, 8H), 7.55 (s, 4H), 7.27 (s, 2H), 7.27 – 7.17 (m, 3H), 7.08 (d, *J* = 7.1 Hz, 2H), 6.91 (s, 1H), 6.82 (s, 2H), 6.73 (s, 2H), 6.35 (s, 2H), 2.24 (s, 6H), 2.21 (s, 6H), 2.04 (s, 6H), 2.01 (s, 6H), 1.62 (s, 3H), 1.37 (s, 3H), 1.15 (s, 9H) ppm. ^{19}F NMR (376 MHz, $CDCl_3$) δ -62.40 (24F), -75.46 (3F) ppm. ^{13}C NMR (101 MHz, $CDCl_3$) δ 301.8, 190.9, 161.8 (q, $^{1}J_{BC}$ = 50.0 Hz), 152.6, 148.0, 141.2, 138.3, 135.5, 134.9, 134.3, 134.1, 132.1, 130.0, 129.0 (qq, $^{2}J_{CF}$ = 31.4 Hz, $^{3}J_{CB}$ = 2.9 Hz), 128.6, 126.9, 125.8, 125.6, 125.1, 124.7 (q, $^{1}J_{CF}$ = 272.5 Hz), 117.6, 54.0, 32.4, 30.7, 29.8, 26.7, 21.1, 21.1, 21.0, 18.1, 18.0 ppm. Elemental analysis (%) calcd. for $C_{77}H_{66}BF_{27}N_4O_3SW$: C 50.40, H 3.63, N 3.05; found: C 50.48, H 3.640, N 3.16.

[W(N-2-*t*BuC$_6$H$_4$)(CHCMe$_2$Ph)OTf(IMes)(pivCN)$^+$ B(Ar^F)$_4^-$] (W-41). A solution of [W(N-2-*t*BuC$_6$H$_4$)(CHCMe$_2$Ph)Cl(IMes)(pivCN)$^+$ B(Ar^F)$_4^-$] (**W-29,** 250 mg, 0.14 mmol) in CH_2Cl_2 (4 mL) was added to a suspension of AgOTf (36.7 mg, 0.14 mmol, 1 equiv.) in CH_2Cl_2 (2 mL). The reaction was stirred at room temperature for three hours, during which time a brownish precipitate formed. The suspension was filtered through a pad of celite and the solvent was removed *in vacuo*. The residue was stirred with pentane until a solid formed. The pentane was removed *in vacuo* to give the product as a yellow solid. Yield: 222 mg (83 %). ^{1}H NMR (400 MHz, $CDCl_3$) δ 11.56 (s, 1H), 7.34 (dd, *J* = 7.9, 1.3 Hz, 1H), 7.26 – 7.11 (m, 5H), 7.03 (d, *J* = 7.2 Hz, 2H), 6.97 (s, 2H), 6.92 (d, *J* = 7.5 Hz, 1H), 6.78 (s, 2H), 2.25 (s, 6H), 2.19 (s, 6H), 1.92 (s, 6H), 1.51 (s, 3H), 1.24 (s, 9H), 1.15 (s, 9H), 0.95 (s, 3H) ppm. ^{19}F NMR (376 MHz, $CDCl_3$) δ -62.38 (24F), -74.57 (3F) ppm. ^{13}C NMR (101 MHz, $CDCl_3$) δ 161.9 (q, $^{1}J_{BC}$ = 49.9 Hz), 152.2, 147.4, 145.8, 141.5, 136.0, 135.6, 134.9, 134.6, 133.4, 130.5, 130.3, 129.8, 129.0 (qq, $^{2}J_{CF}$ = 31.5 Hz, $^{3}J_{CB}$ = 2.9 Hz), 128.7, 127.1, 126.4, 126.4, 126.0, 125.6, 124.7 (q, $^{1}J_{CF}$ = 272.5 Hz), 117.6, 56.9, 35.4, 30.8, 30.6, 30.4, 26.8, 21.0, 18.6, 18.5 ppm. Elemental analysis (%) calcd. for $C_{79}H_{70}BF_{27}N_4O_3SW$: C 50.93, H 3.79, N 3.01; found: C 51.07, H 3.832, N 3.00.

[Mo(N-2,6-$Me_2C_6H_3$)($CHCMe_2Ph$)(OC_6F_5)(IMes)$^+$ B(Ar^F)$_4^-$] (Mo-02). A solution of Mo(N-2,6-$Me_2C_6H_3$)($CHCMe_2Ph$)(OC_6F_5)(OTf)(IMes) (200 mg, 0.20 mmol) in CH_2Cl_2 (3 mL) was added to a stirred suspension of NaB(Ar^F)$_4$ (180 mg, 0.20 mmol, 1 equiv.) in CH_2Cl_2 (3 mL). The resulting red suspension was stirred at room temperature for two hours and then filtered through a pad of celite. The solvent was partially removed under reduced pressure and pentane was added. The solution was stored at -35 °C overnight during which time the product crystallized as dark orange crystals. Yield: 214 mg (62 %). ^{1}H NMR (400 MHz, $CDCl_3$) δ 12.46 (s, 1H), 7.73 (s, 8H), 7.52 (s, 4H), 7.46 (s, 2H), 7.16 – 7.08 (m, 1H), 7.03 (s, 2H), 6.99 (d, J = 7.5 Hz, 2H), 6.96 – 6.91 (m, 2H), 6.91 – 6.85 (m, 1H), 6.86 (s, 2H), 6.84 – 6.79 (m, 2H), 2.29 (s, 6H), 2.04 (s, 12H), 1.97 (s, 6H), 1.41 (s, 3H), 0.81 (s, 3H) ppm. ^{19}F NMR (376 MHz, $CDCl_3$) δ -62.42 (24F), -159.25 (2F), -162.98 (2F), -165.13 (1F) ppm. ^{13}C NMR (101 MHz, $CDCl_3$) δ 310.4, 179.3, 161.9 (q, $^1J_{BC}$ = 49.9 Hz), 156.0, 146.1, 142.6, 140.6, 139.3, 138.5, 138.1, 137.6, 136.8, 135.0, 134.9, 132.7, 130.8, 130.6, 130.2, 129.0 (qq, $^2J_{CF}$ = 31.5 Hz, $^3J_{CB}$ = 2.9 Hz), 128.4, 128.3, 126.9, 126.7, 125.4, 124.7 (q, $^1J_{CF}$ = 272.5 Hz), 117.6, 56.3, 34.3, 28.4, 28.2, 22.5, 21.0, 21.0, 19.2, 17.5, 14.2 ppm. Elemental analysis (%) calcd. for $C_{77}H_{57}BF_{29}MoN_3O$: C 54.47, H 3.38, N 2.47; found: C 54.48, H 3.432, N 2.57.

[Mo(N-2,6-$Cl_2C_6H_3$)($CHCMe_3$)(OTf)(IMes)(MeCN)$^+$ B(Ar^F)$_4^-$] (Mo-03-MeCN). Acetonitrile (3.2 µl, 0.06 mmol, 2 equiv.) was added to a stirred solution of [Mo(N-2,6-$Cl_2C_6H_3$)($CHCMe_3$)(OTf)(IMes)$^+$ B(Ar^F)$_4^-$] (**Mo-03**, 50 mg 0.03 mmol) in CH_2Cl_2 (1 mL) resulting in a slight color change. The solvent was removed under reduced pressure and the residue was extracted with pentane until a light-yellow solid formed. The pentane was decanted and the solid was dried *in vacuo*. Yield: 50 mg (98 %). ^{1}H NMR (400 MHz, $CDCl_3$) δ 12.95 (s, 1H), 7.70 (s, 8H), 7.51 (s, 4H), 7.24 (s, 2H), 7.22 (s, 1H), 7.15 – 7.03 (m, 2H), 6.98 (s, 2H), 6.70 (s, 2H), 2.23 (s, 3H), 2.23 (s, 6H), 2.07 (s, 6H), 2.04 (s, 6H), 1.04 (s, 9H) ppm. ^{19}F NMR (376 MHz, $CDCl_3$) δ -62.40 (24F), -75.33 (3F) ppm. ^{13}C NMR (101 MHz, $CDCl_3$) δ 335.3, 183.4, 161.8 (q, $^1J_{BC}$ = 49.8 Hz), 148.6, 141.3, 140.2, 135.7, 135.1, 134.9, 134.5, 132.0, 131.3, 130.6, 129.4, 129.0 (qq, $^2J_{CF}$ = 31.6 Hz, $^3J_{CB}$ = 2.8 Hz), 128.2, 125.5, 124.7 (q, $^1J_{CF}$ = 272.5 Hz), 120.9, 117.6, 51.2, 29.9, 21.1, 21.0, 18.2, 18.2, 3.2, 3.2 ppm. Elemental analysis (%) calcd. for $C_{67}H_{52}BCl_2F_{27}MoN_4O_3S$: C 47.79, H 3.11, N 3.33; found: C 47.81, H 3.158, N 3.40.

[Mo(N-2,6-$Cl_2C_6H_3$)($CHCMe_3$)(O*t*Bu)(IMes)$^+$ B(Ar^F)$_4^-$] (Mo-05). A solution of [Mo(N-2,6-Cl_2-C_6H_3)($CHCMe_3$)(IMes)(OTf)$^+$ B(Ar^F)$_4^-$] (**Mo-03**, 120 mg, 0.07 mmol) in CH_2Cl_2 (2 mL) was added to a suspension of $LiOC_4H_9$ (5.8 mg, 0.07 mmol, 1 equiv.) in CH_2Cl_2 (2 mL). The suspension was stirred for three hours at room temperature and filtered through a pad of celite. The solvent was partially removed *in vacuo* and pentane was added. The solution was stored at -35 °C overnight, during which time orange crystals of the product were obtained. Yield: 94 mg (82 %). ^{1}H NMR (400 MHz, CD_2Cl_2) δ 12.28 (s, 1H), 7.73 (s, 8H), 7.57 (s, 4H), 7.47 (s, 2H), 7.29 (d, J = 8.4 Hz, 2H), 7.13 (dd, J = 8.6, 7.6 Hz, 1H), 7.03 (s, 2H), 6.85 (s, 2H), 2.25 (s, 6H), 2.07 (s, 6H), 2.05 (s, 6H), 1.34 (s, 9H), 0.94 (s, 9H) ppm. ^{19}F NMR (376 MHz, $CDCl_3$) δ -62.83 ppm. ^{13}C NMR (101 MHz, CD_2Cl_2) δ 308.3, 180.1, 162.3 (q, $^1J_{BC}$ = 49.8 Hz), 150.1, 142.1, 135.4, 135.3, 135.0, 134.6, 133.4, 130.8, 130.2, 129.4 (qq, $^2J_{CF}$ = 31.4 Hz, $^3J_{CB}$ = 3.0 Hz), 129.3, 128.6, 126.7, 125.2 (q, $^1J_{CF}$ = 272.5 Hz), 118.0, 87.7, 48.8, 32.4, 31.4, 21.4, 21.4, 18.6, 18.5 ppm. Elemental analysis

(%) calcd. for for $C_{68}H_{58}BCl_2F_{24}MoN_3O$: C, 52.13; H, 3.73; N, 2.68. Found: C, 52.33; H, 3.801; N, 2.90.

3. NMR Spectra of Organic Compounds

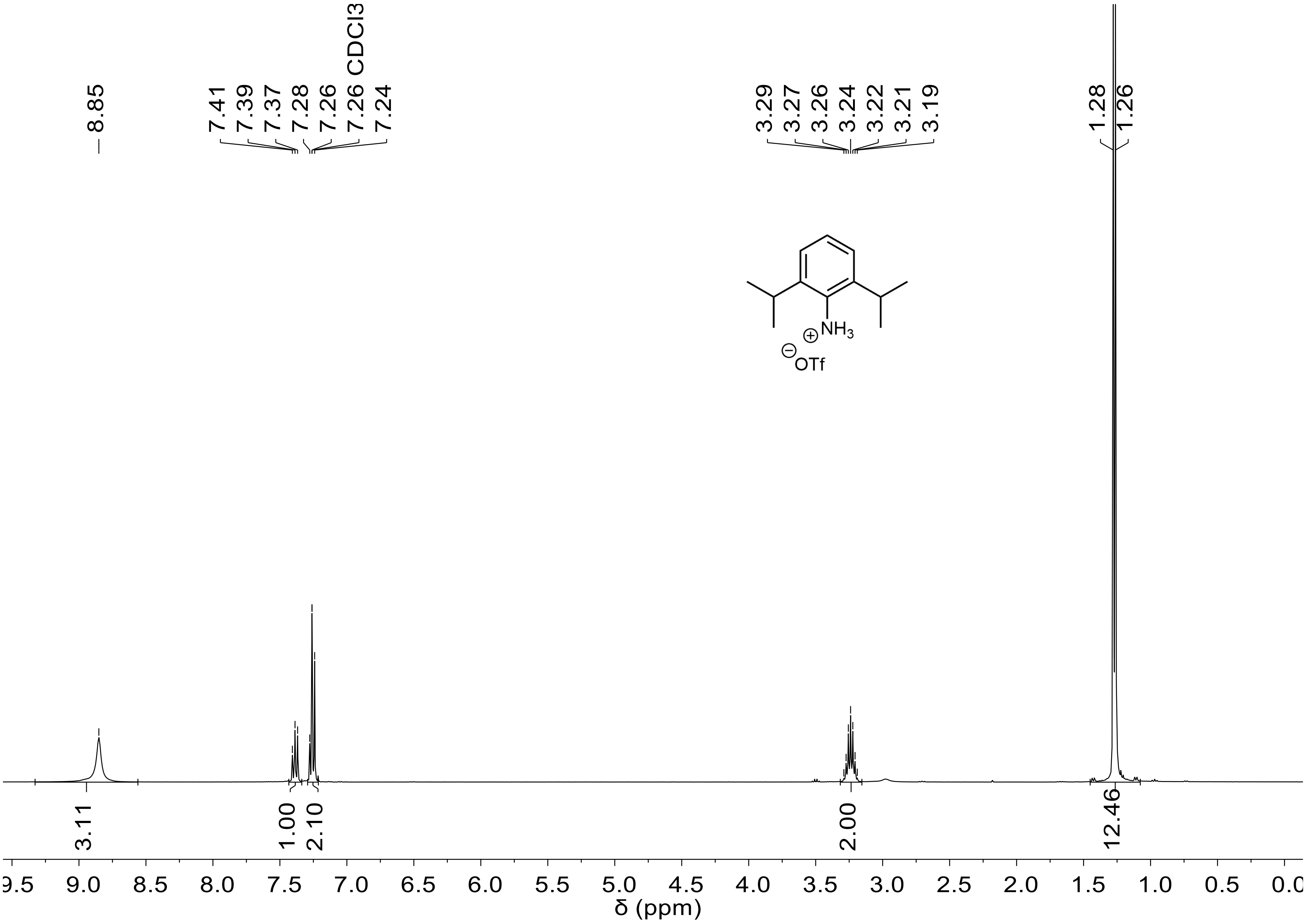

Figure S 1: ^{1}H-NMR (400 MHz, 25 °C, $CDCl_3$) spectrum of 2,6-diisopropylanilinium triflate.

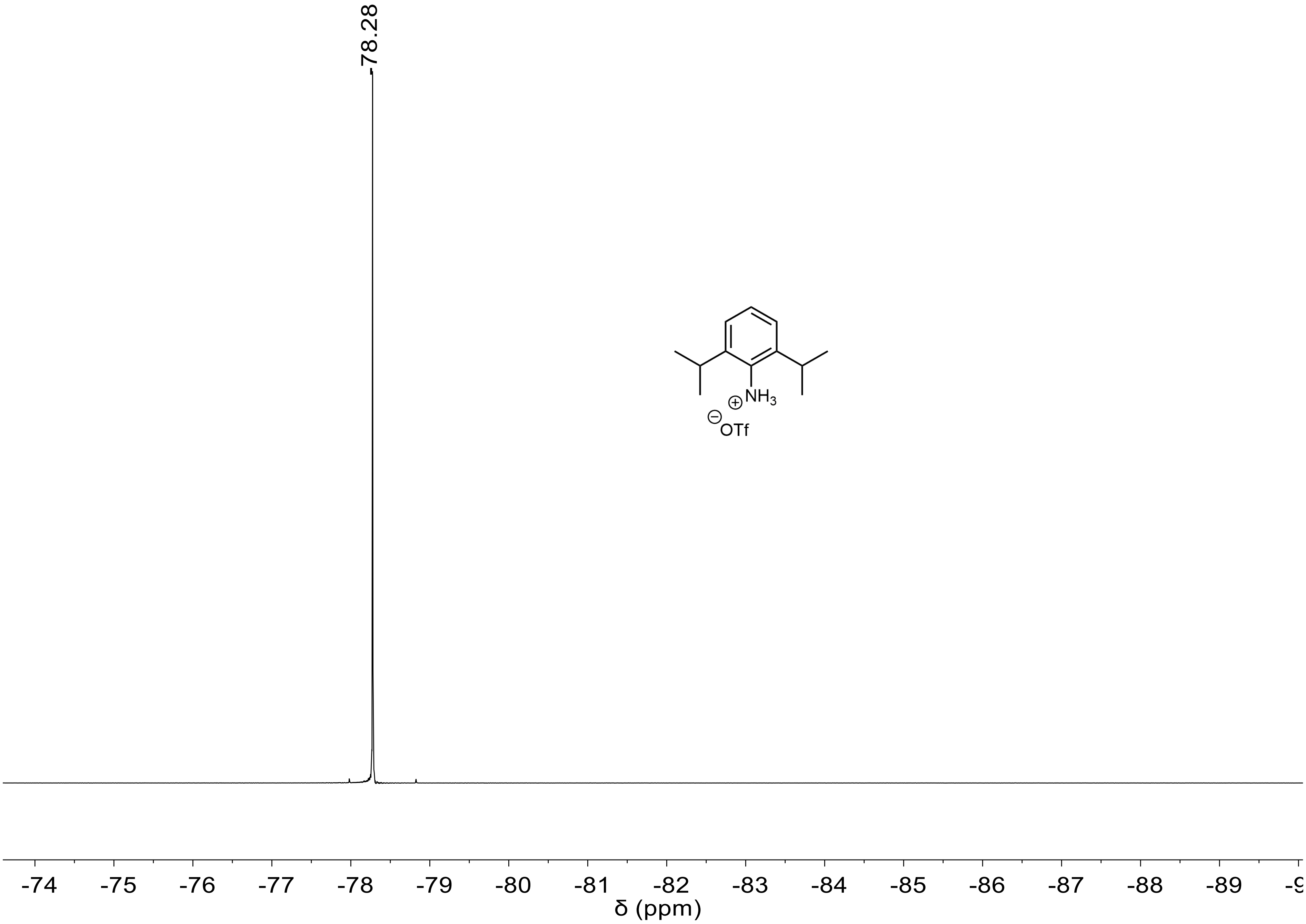

Figure S 2. ^{19}**F-NMR (376 MHz, 25 °C, CDCl$_3$) spectrum of 2,6-diisopropylanilinium triflate.**

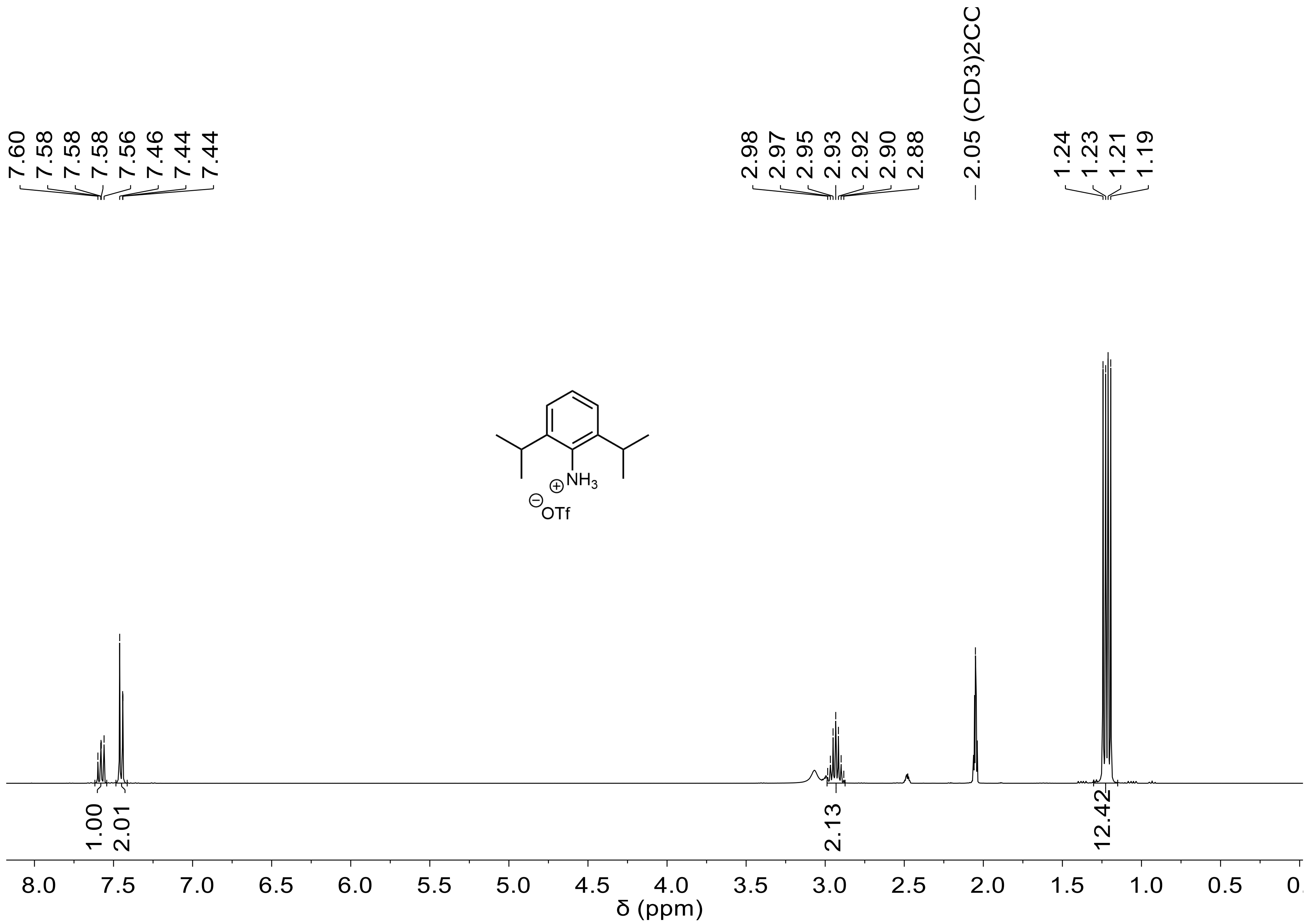

Figure S 3: ^{1}H-NMR (400 MHz, 25 °C, acetone-d_6) spectrum of 2,6-diisopropylanilinium triflate.

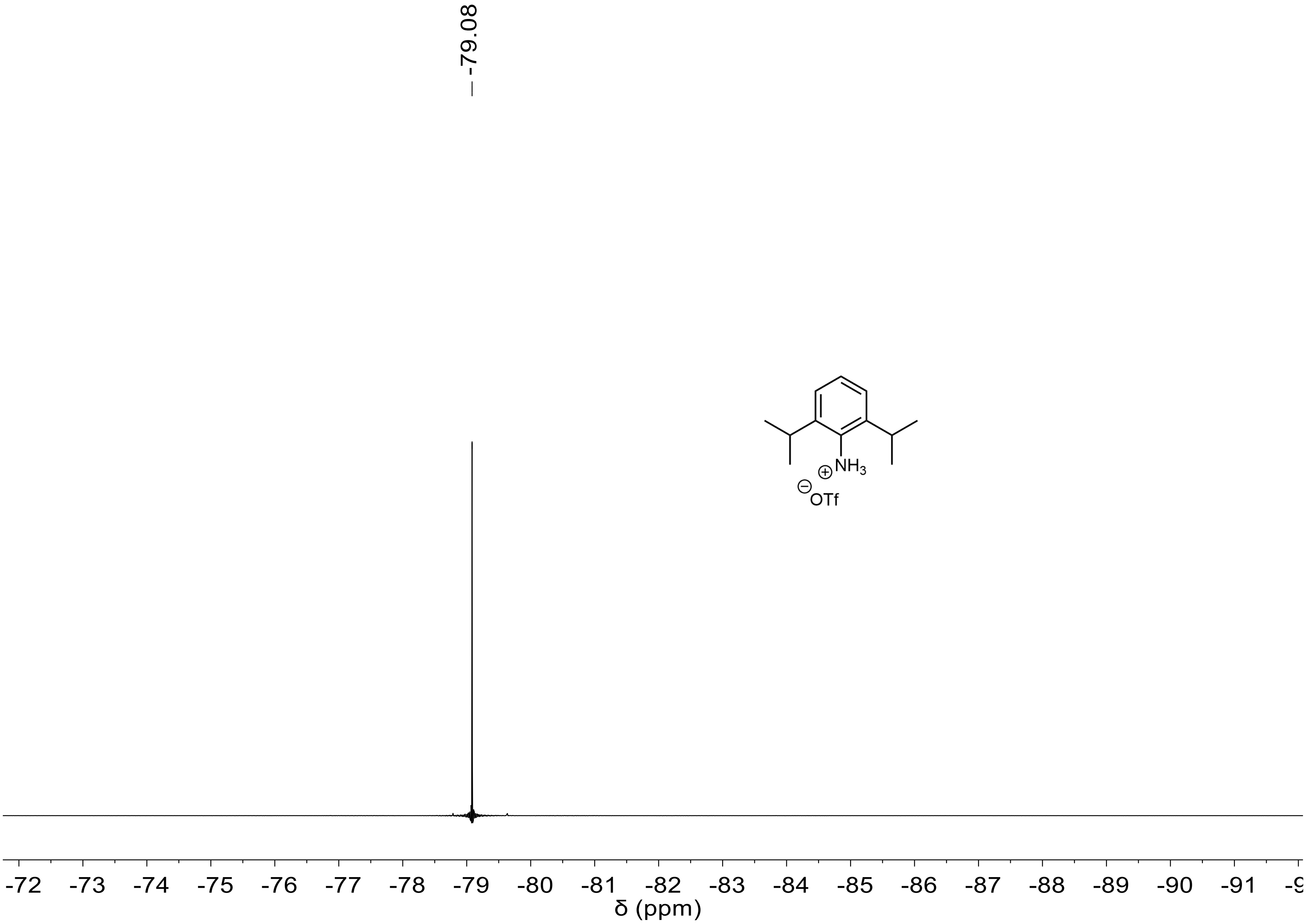

Figure S 4: ^{19}F-NMR (376 MHz, 25 °C, acetone-d_6) spectrum of 2,6-diisopropylanilinium triflate.

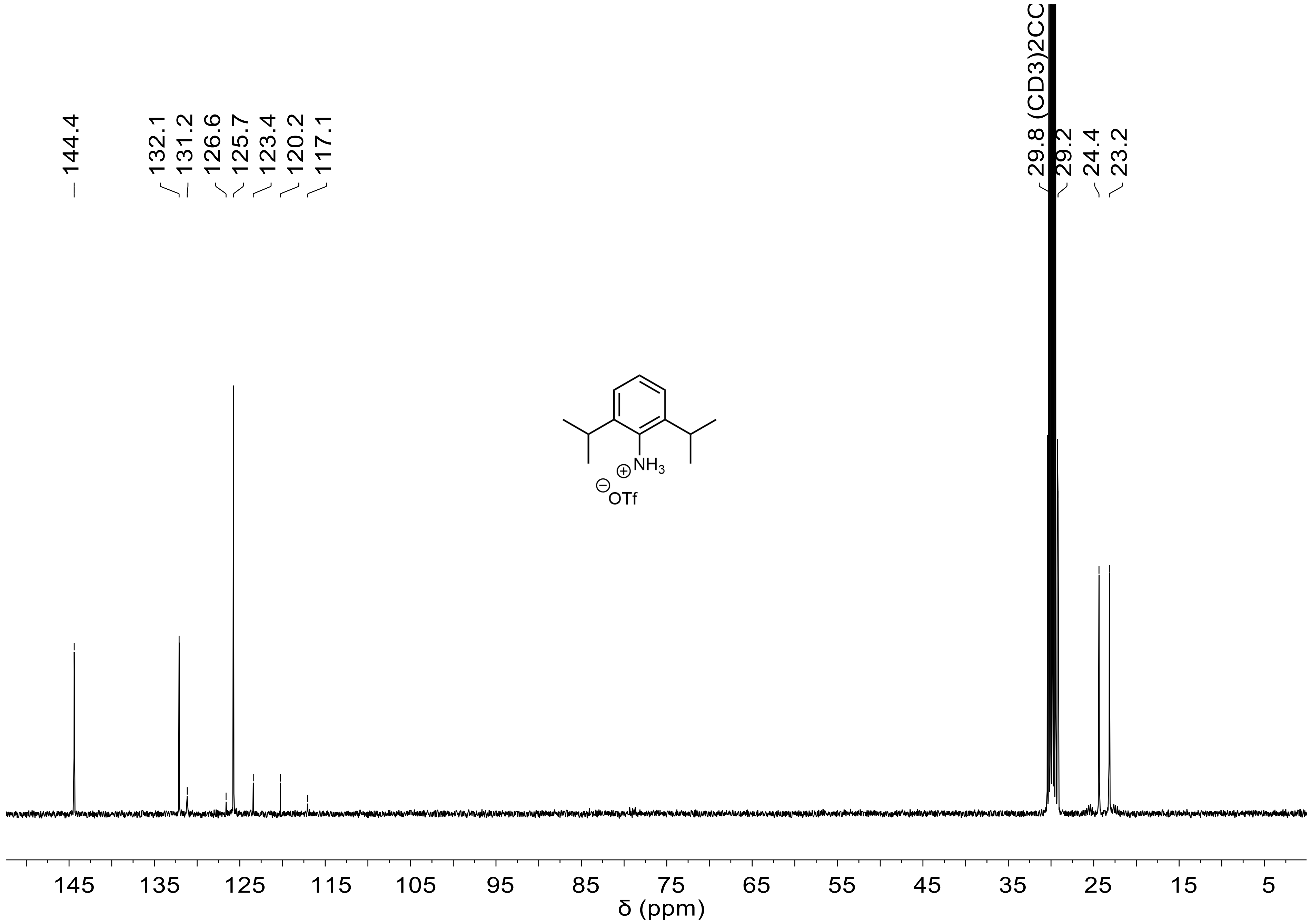

Figure S 5: ^{13}C-NMR (101 MHz, 25 °C, acetone-d_6) spectrum of 2,6-diisopropylanilinium triflate.

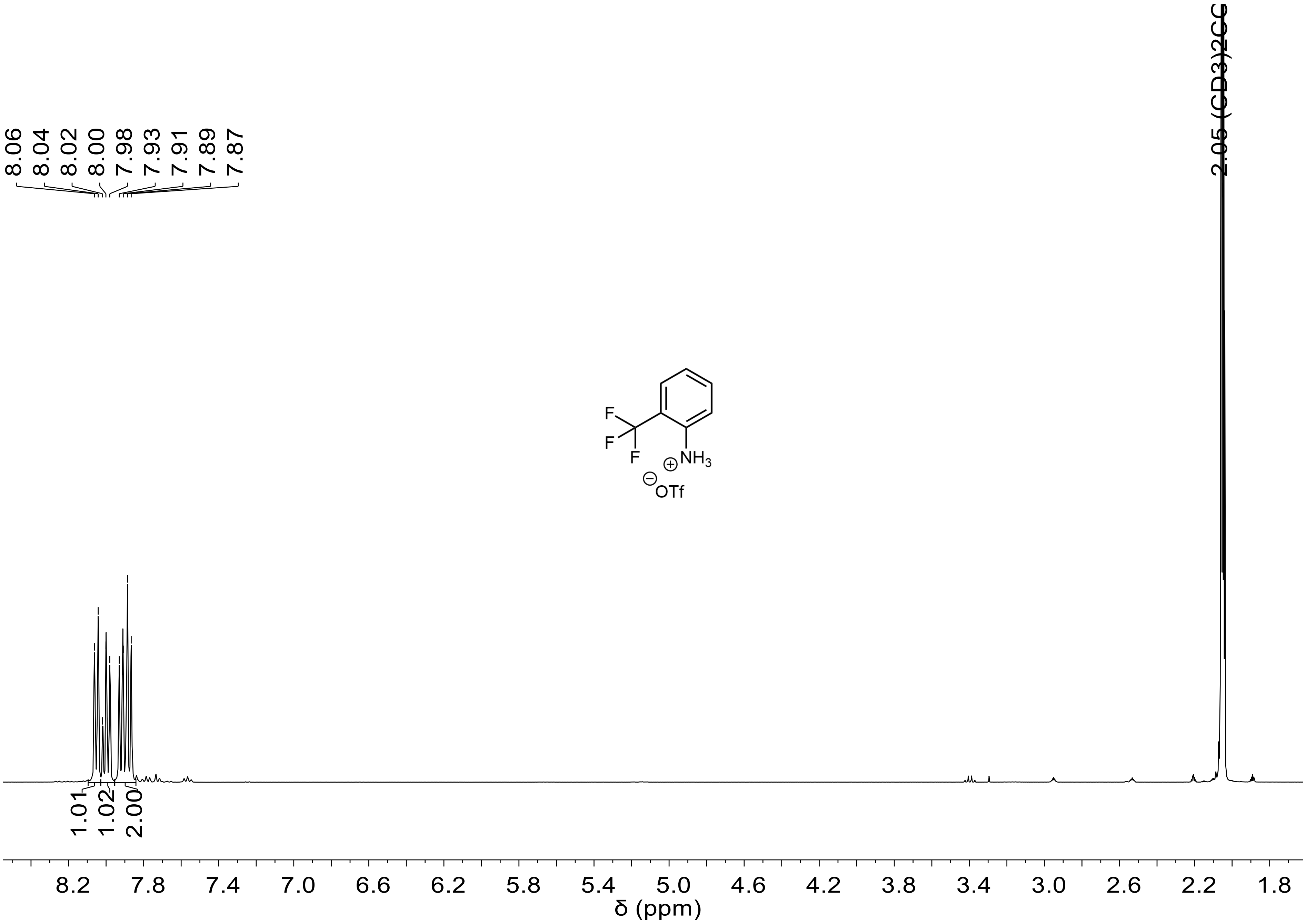

Figure S 6: ^{1}H-NMR (400 MHz, 25 °C, acetone-d_6) spectrum of 2-trifluormethylanilinium triflate.

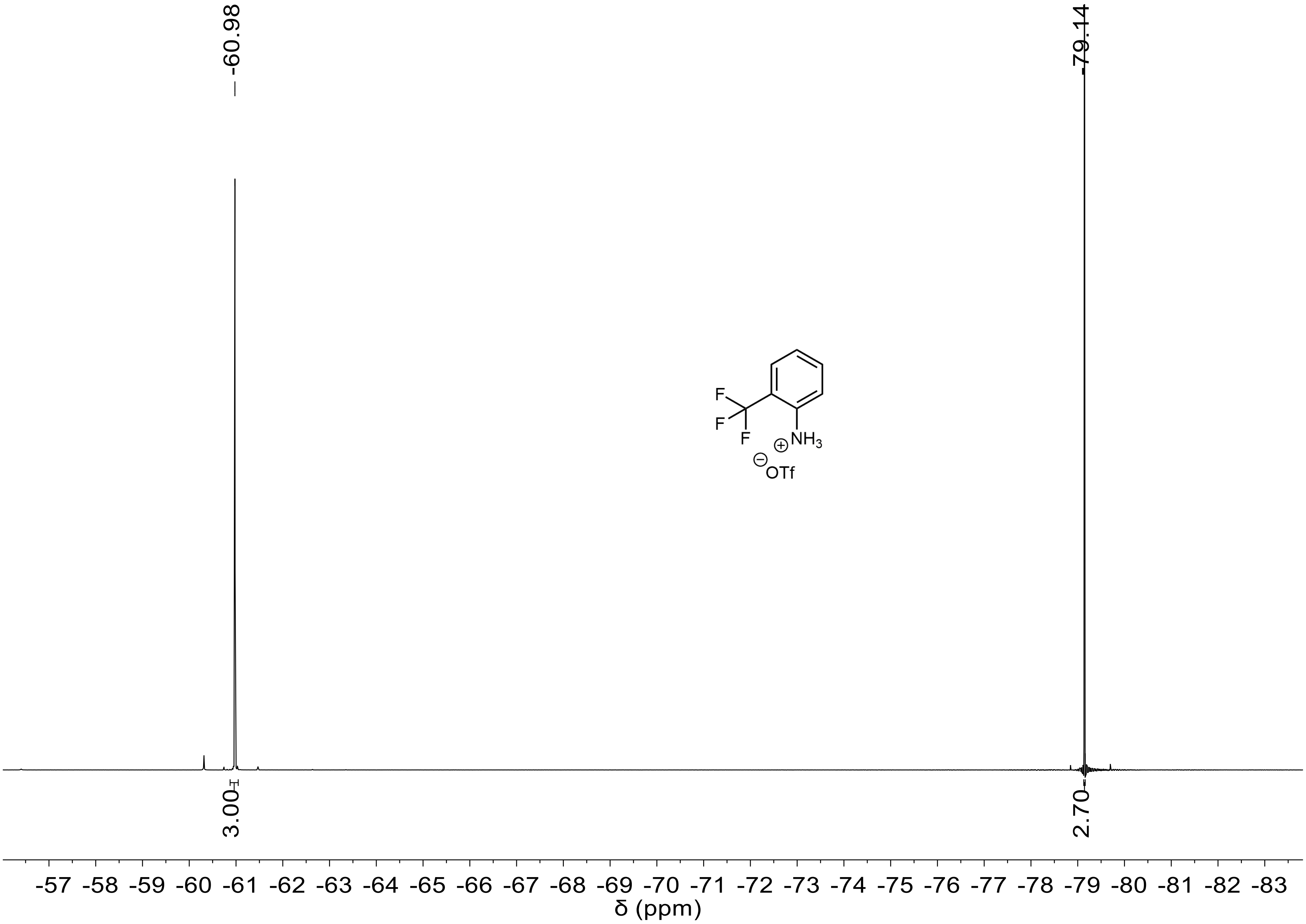

Figure S 7: ^{19}F-NMR (376 MHz, 25 °C, acetone-d$_6$) spectrum of 2-trifluormethylanilinium triflate.

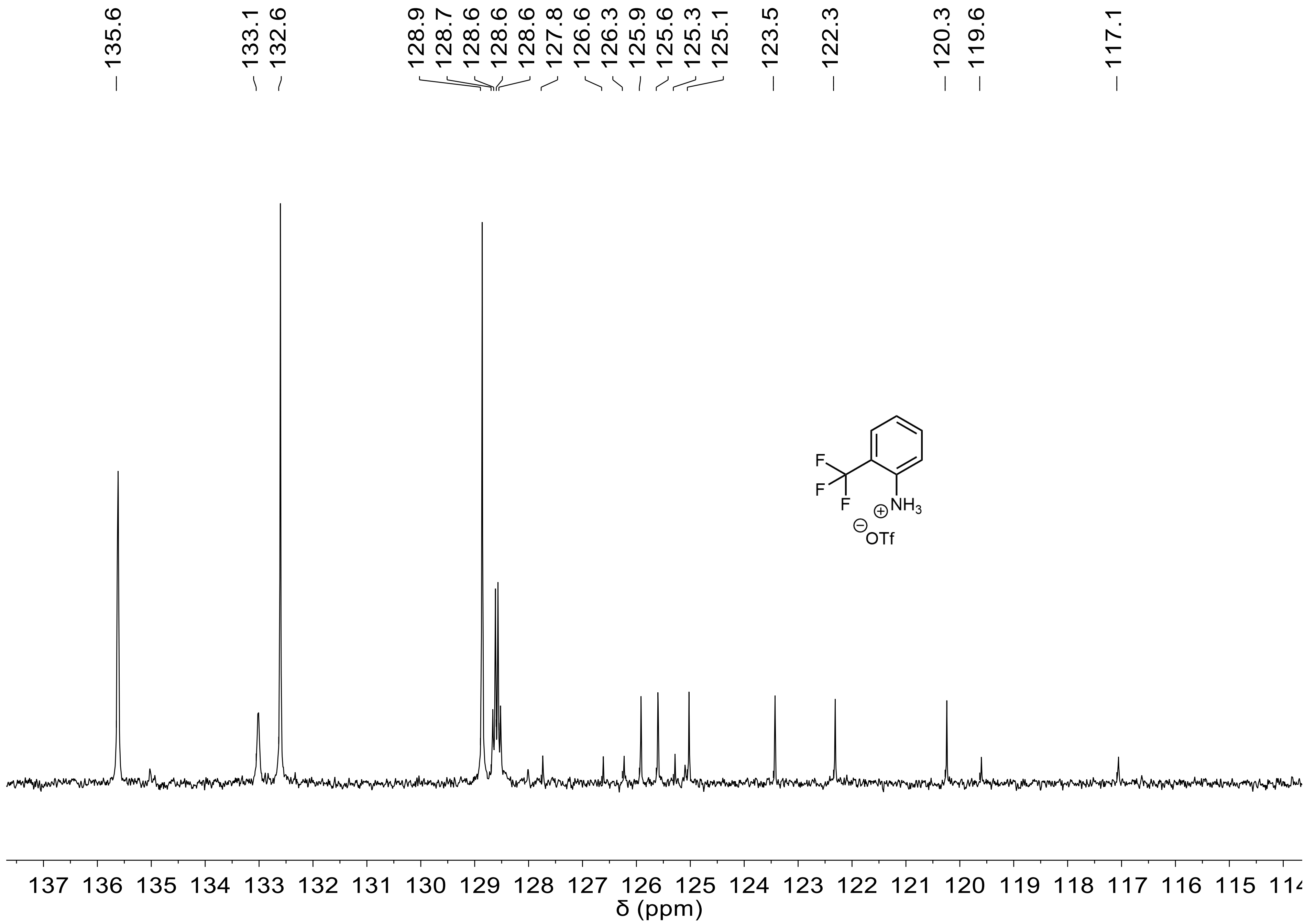

Figure S 8: ^{13}C-NMR (101 MHz, 25 °C, acetone-d_6) spectrum of 2-trifluormethylanilinium triflate.

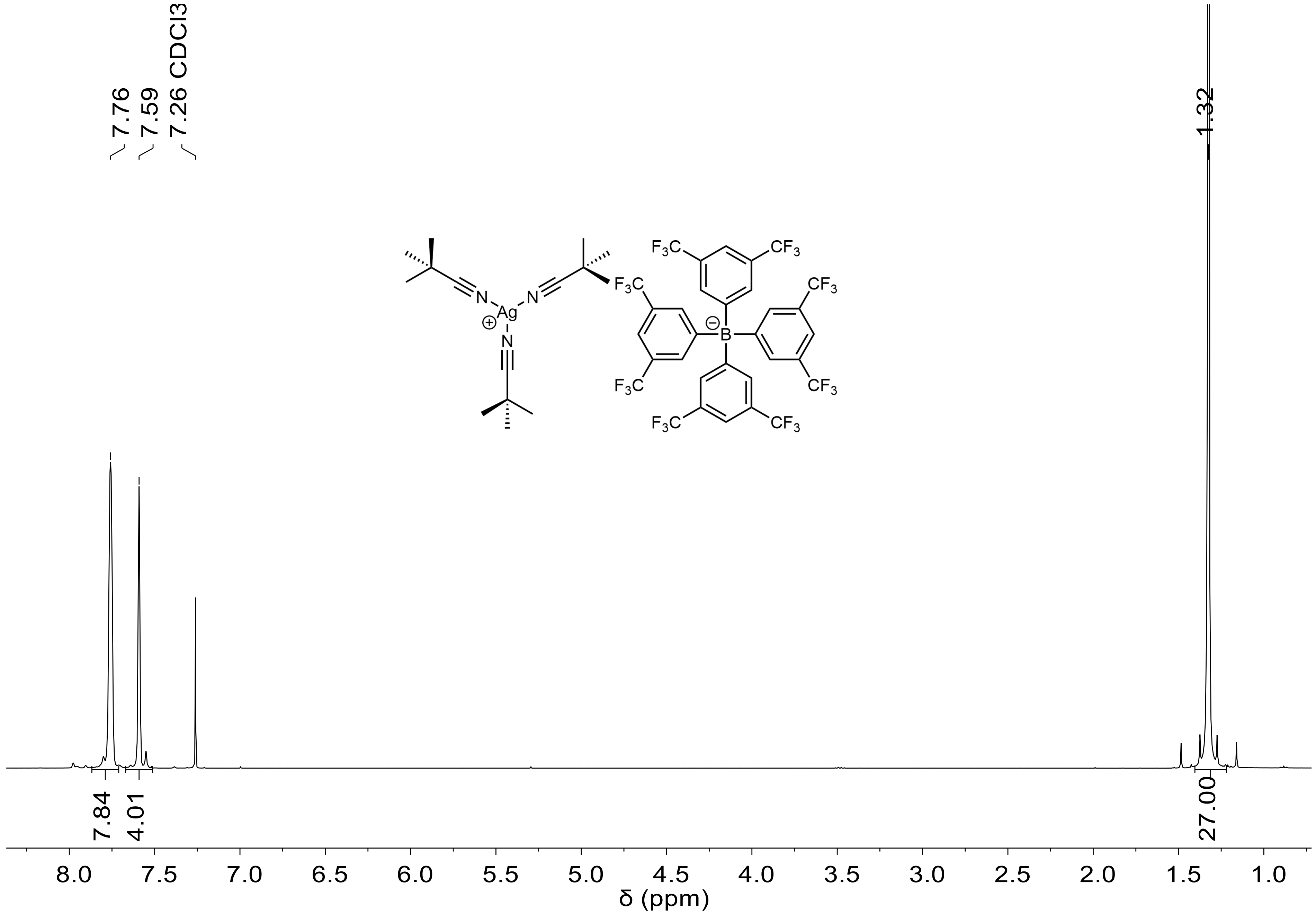

Figure S9: ^{1}H-NMR (400 MHz, 25 °C, $CDCl_3$) of [Ag(pivCN)$_3$$^+$ B(Ar^F)$_4$$^-$].

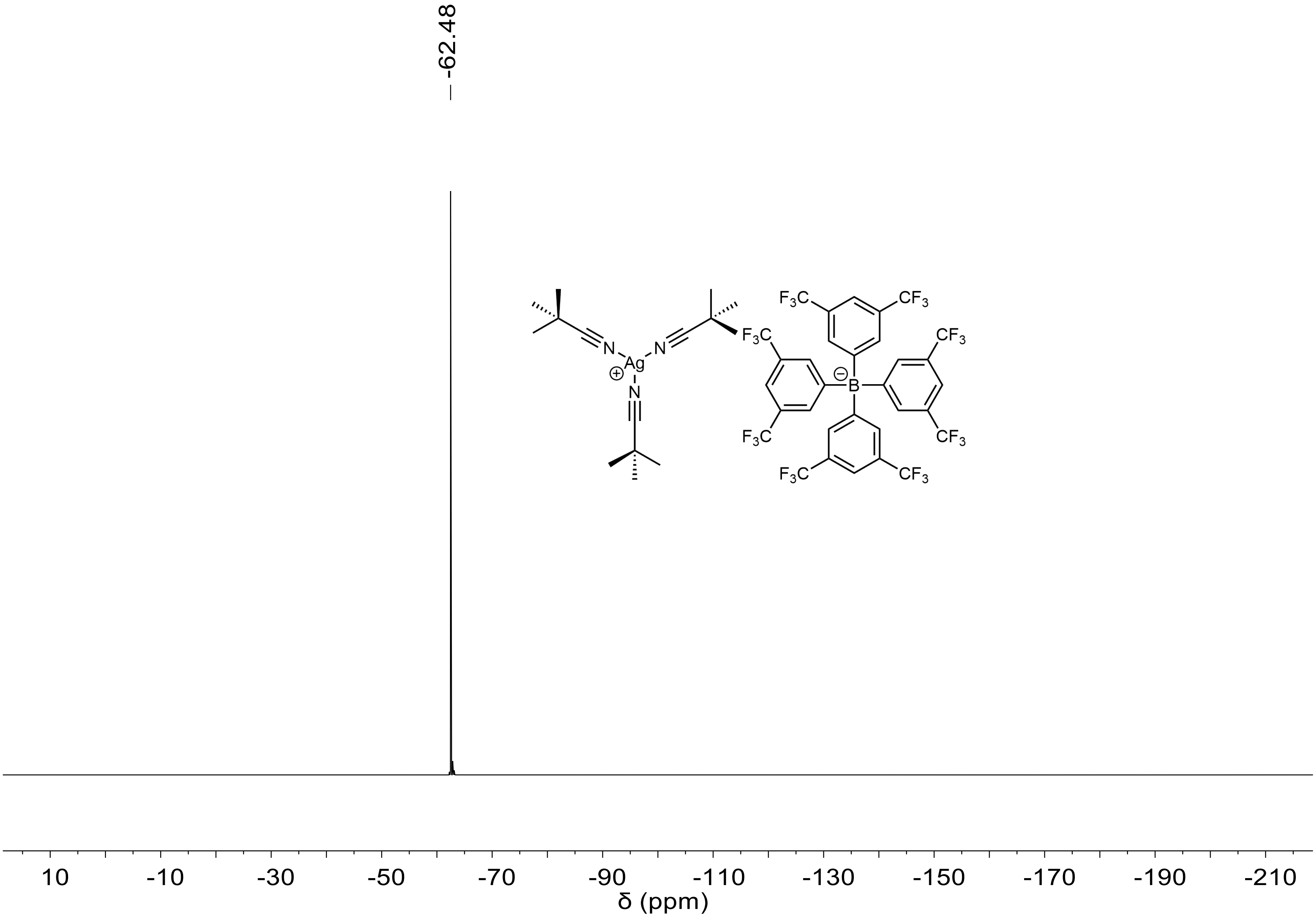

Figure S10: ^{19}F-NMR (376 MHz, 25 °C, CDCl$_3$) of [Ag(pivCN)$_3^+$ B(Ar^F)$_4^-$].

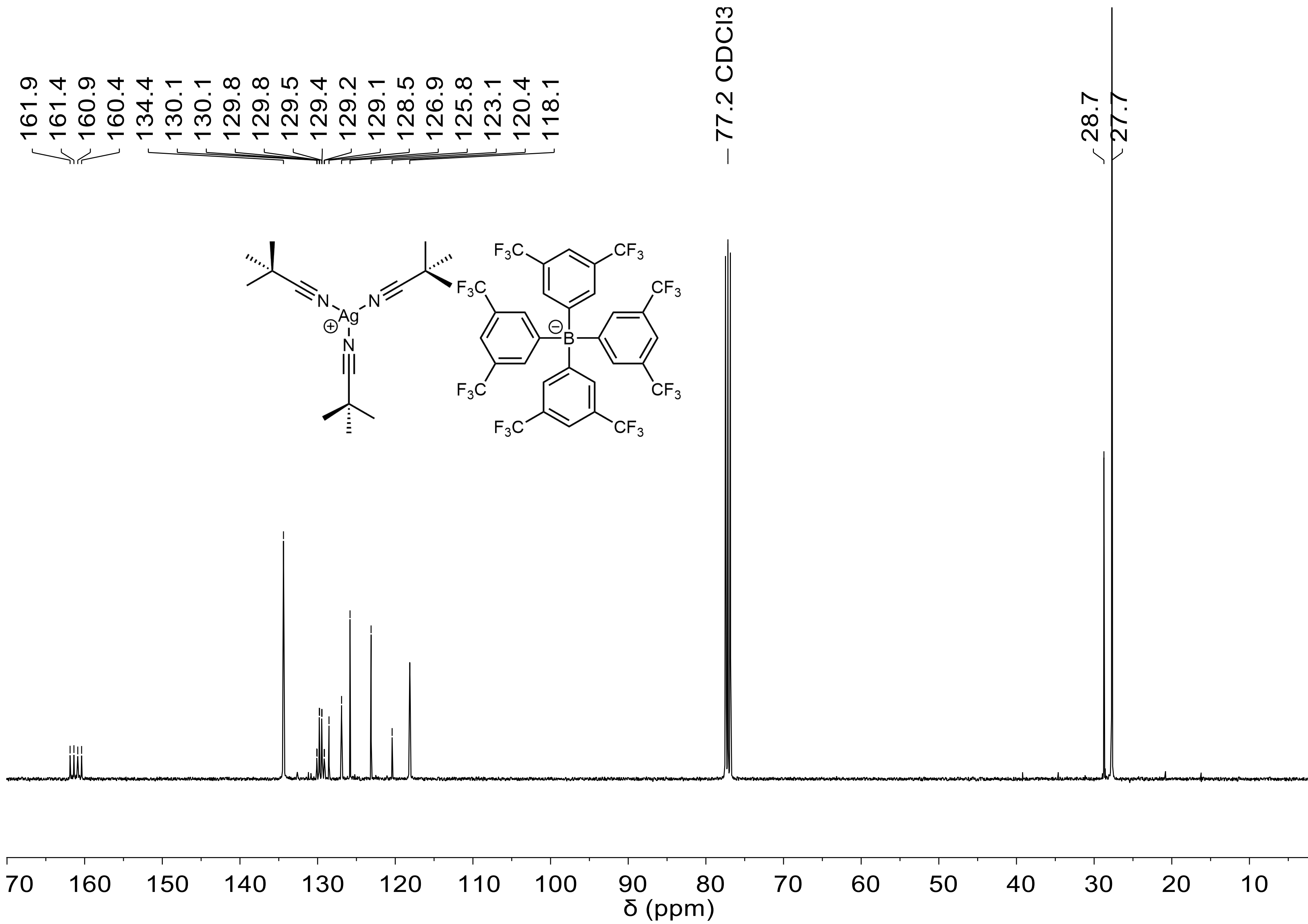

Figure S11: ^{13}C-NMR (101 MHz, 25 °C, $CDCl_3$) of [Ag(pivCN)$_3$$^+$ B(Ar^F)$_4$$^-$].

4. NMR-Spectra of Tungsten and Molybdenum Complexes

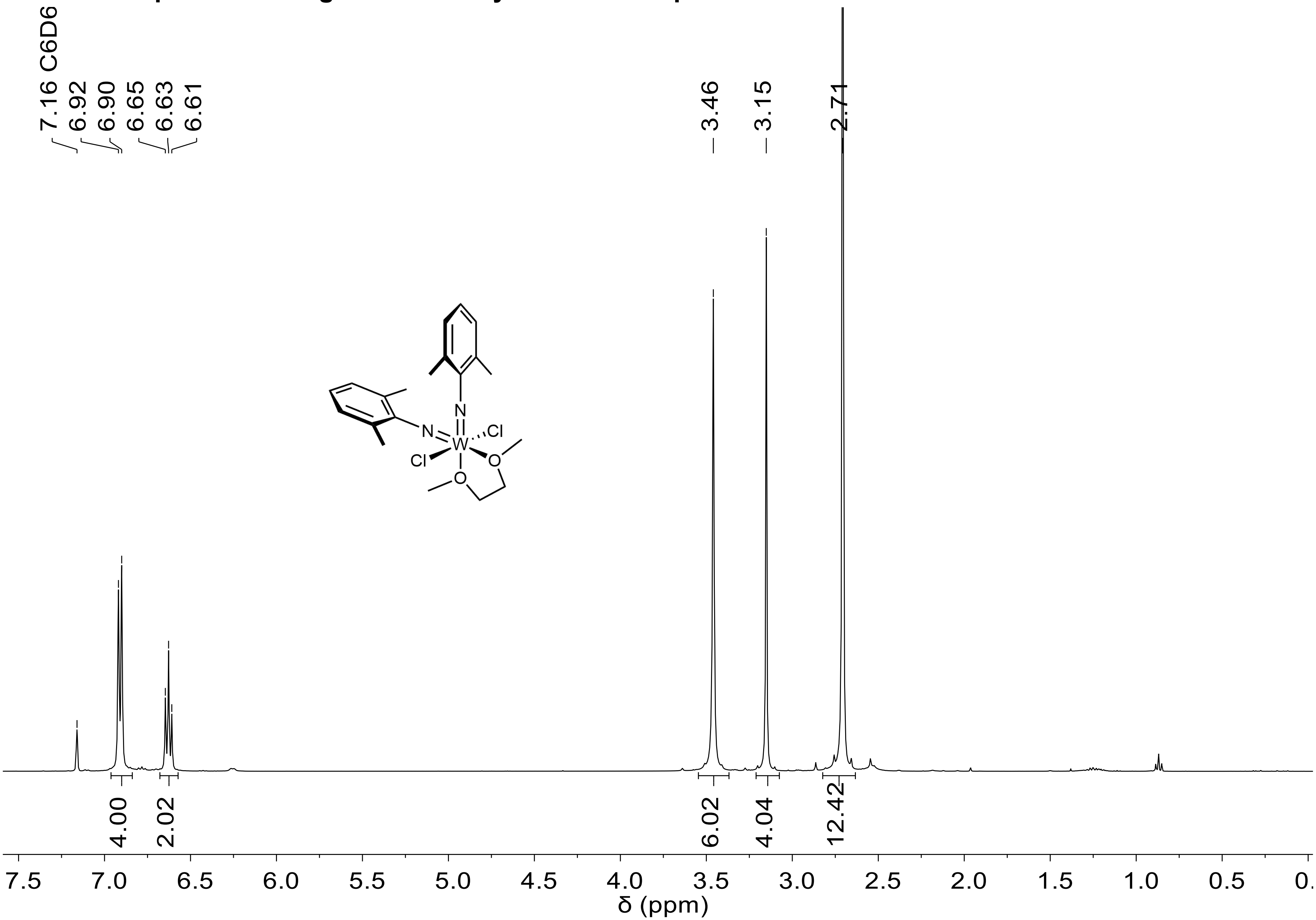

Figure S12: ^{1}H-NMR (400 MHz, 25 °C, C_6D_6) of W(N-2,6-$Me_2C_6H_3)_2(Cl)_2$(DME).

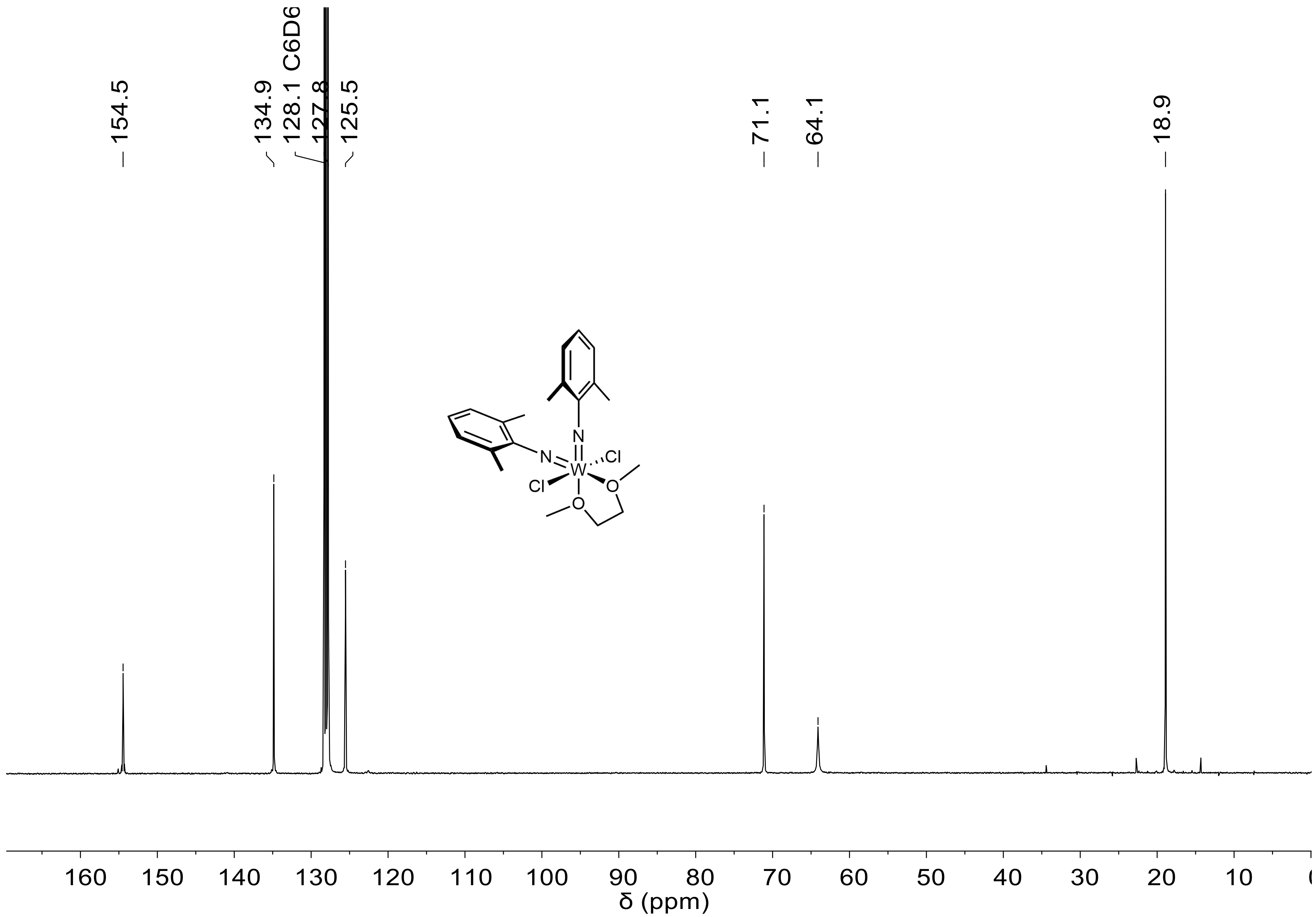

Figure S13: ^{13}C-NMR (101 MHz, 25 °C, C_6D_6) of W(N-2,6-$Me_2C_6H_3$)$_2$(Cl)$_2$(DME).

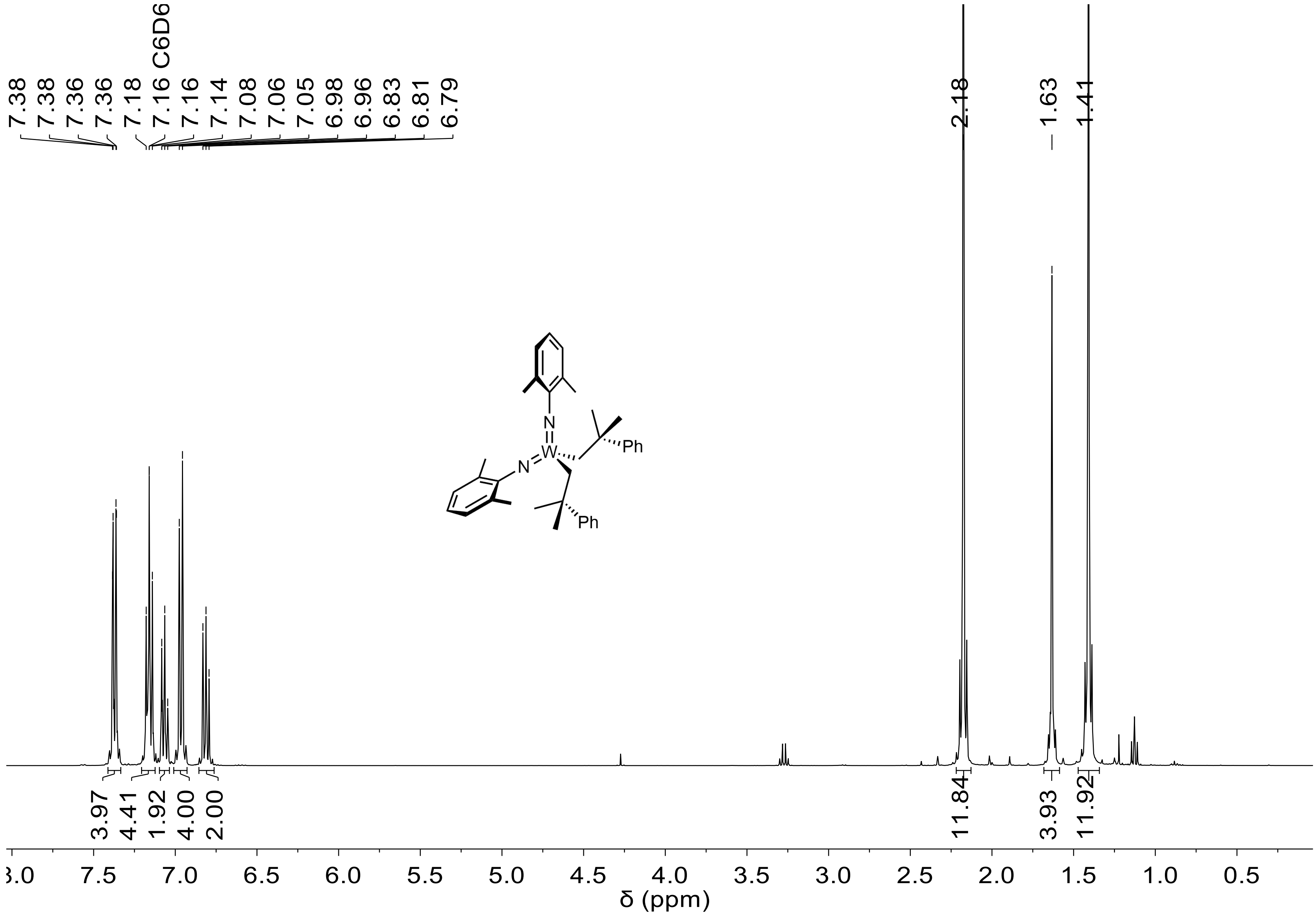

Figure S14: ^{1}H-NMR (400 MHz, 25 °C, C_6D_6) of $W(N\text{-}2,6\text{-}Me_2C_6H_3)_2(CHCMe_2Ph)_2$.

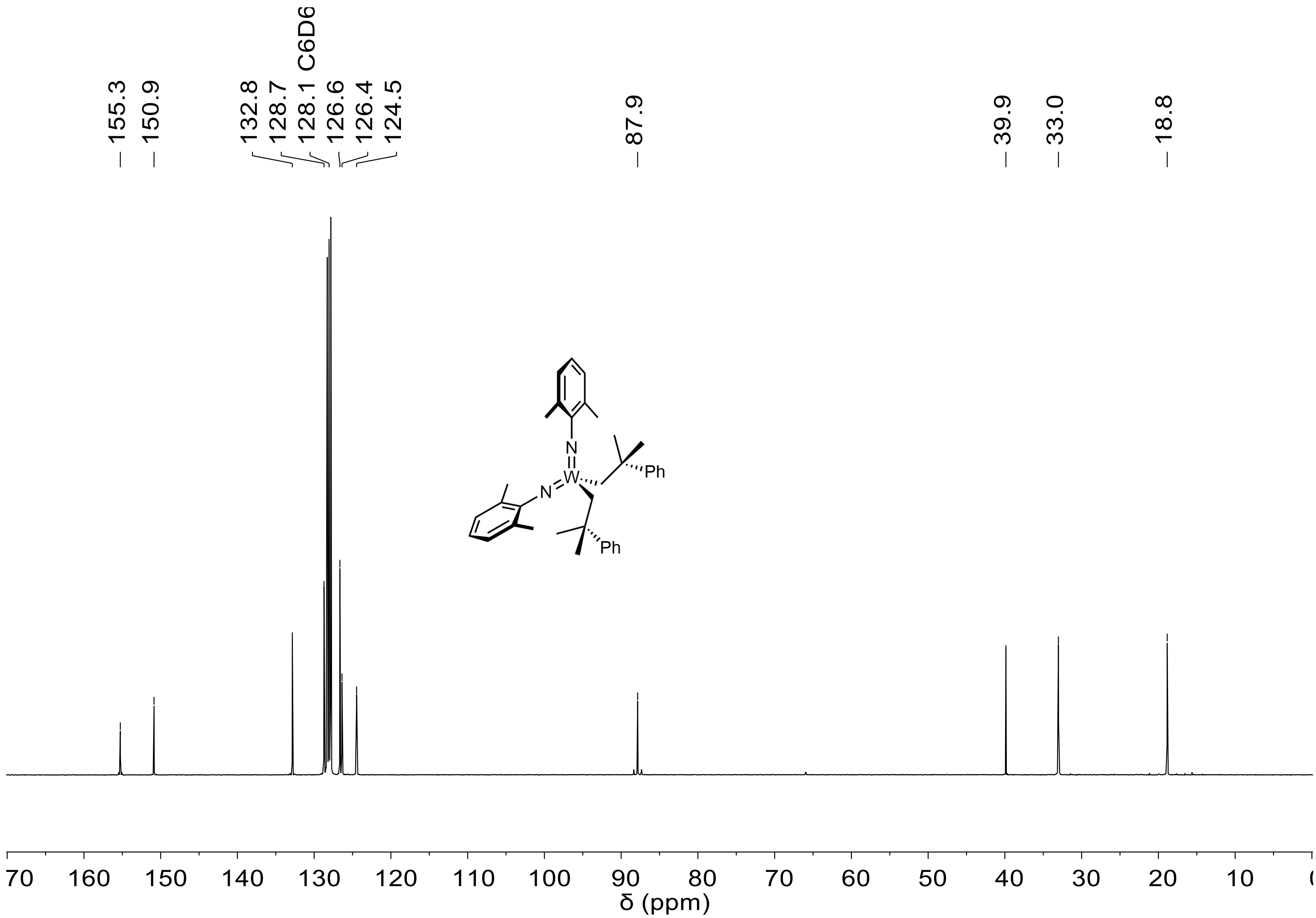

Figure S15: ^{13}C-NMR (101 MHz, 25 °C, C_6D_6) of $W(N\text{-}2,6\text{-}Me_2C_6H_3)_2(CHCMe_2Ph)_2$.

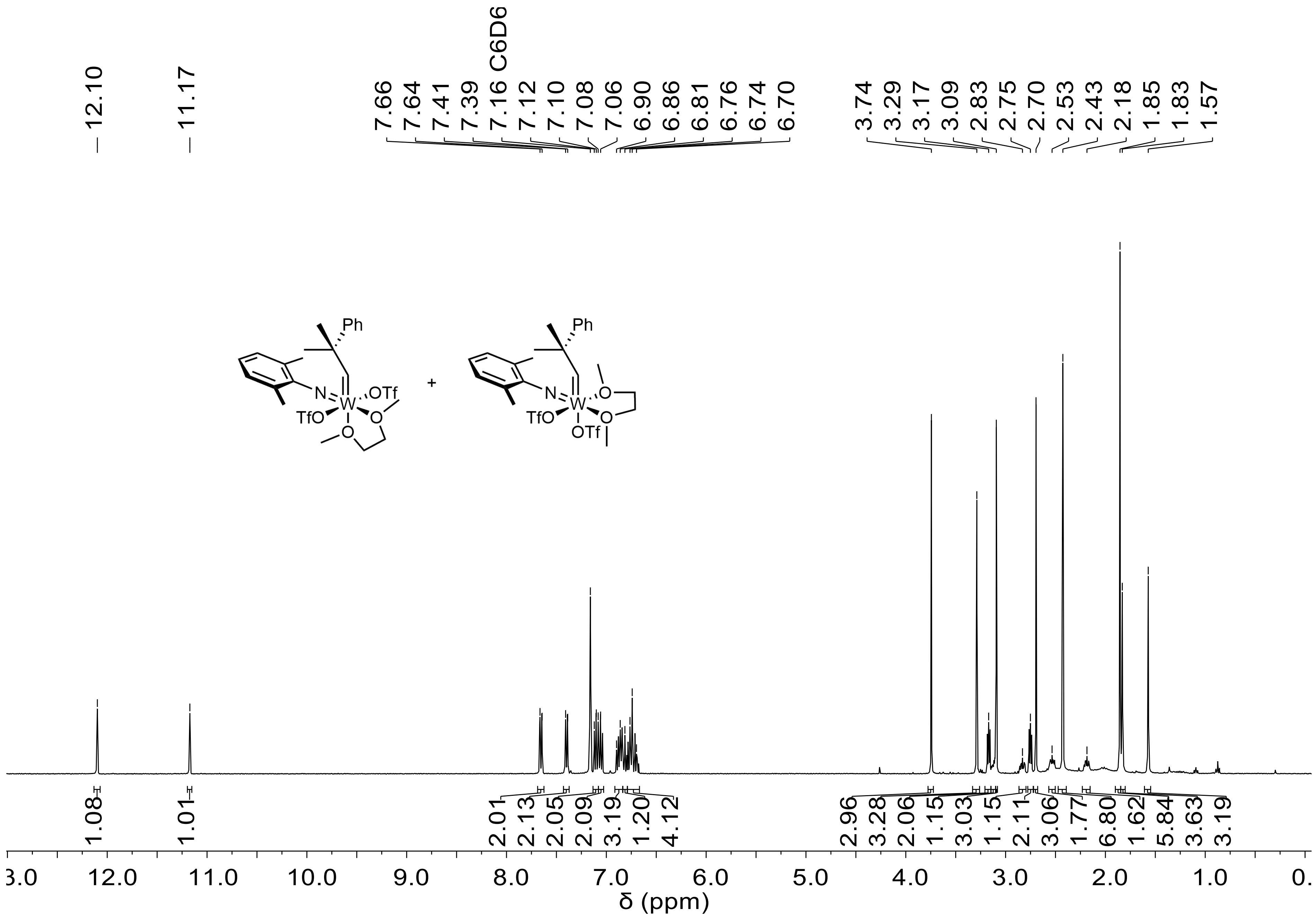

Figure S16: ^{1}H-NMR (400 MHz, 25 °C, C_6D_6) of W(N-2,6-$Me_2C_6H_3$)($CHCMe_2Ph$)$(OTf)_2$(DME). The sample contained equal amounts of *cis*- and *trans*-isomer.

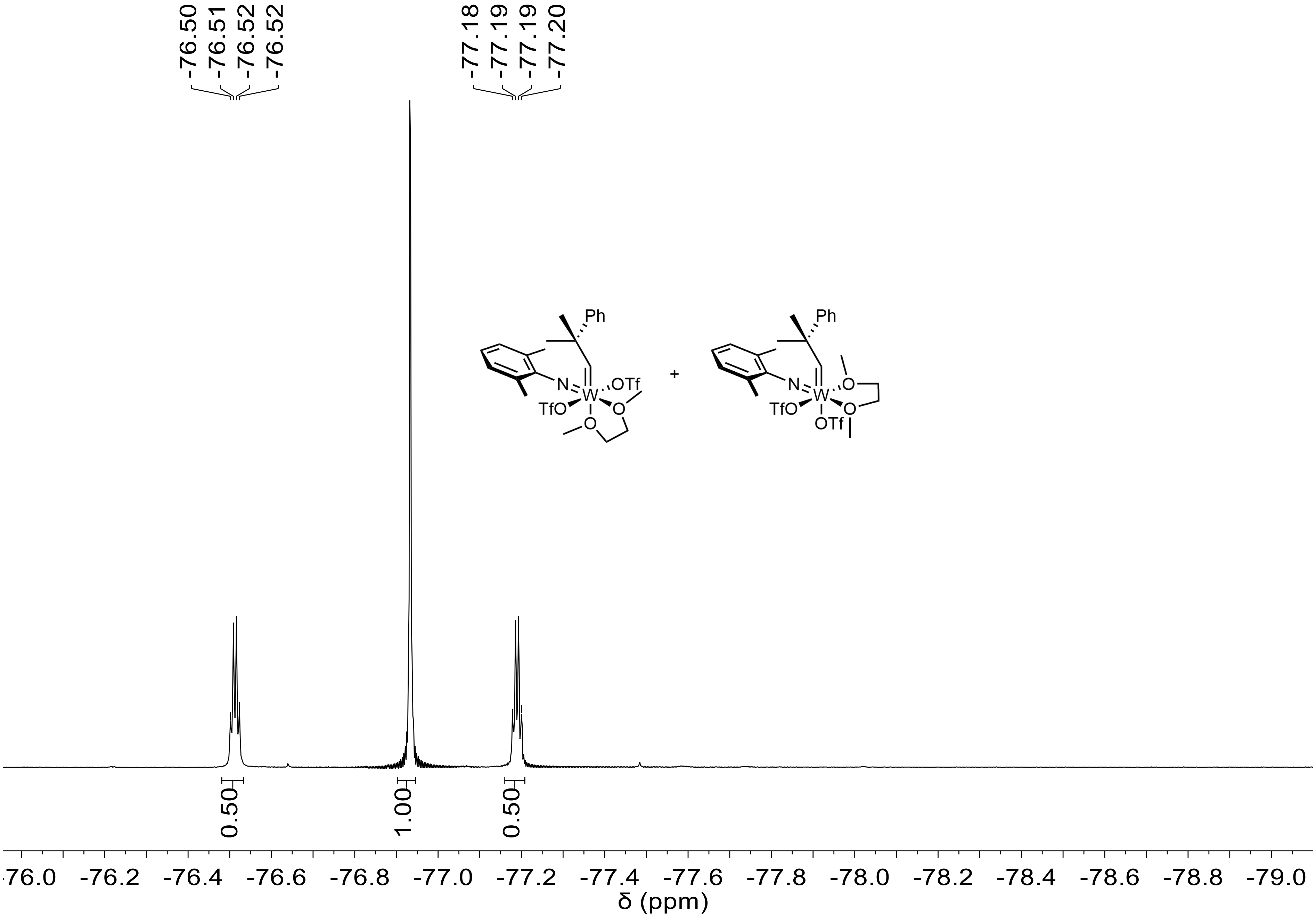

Figure S17: ^{19}F-NMR (376 MHz, 25 °C, C_6D_6) of W(N-2,6-$Me_2C_6H_3$)($CHCMe_2Ph$)$(OTf)_2$(DME). The sample contained equal amounts of *cis*- and *trans*-isomer.

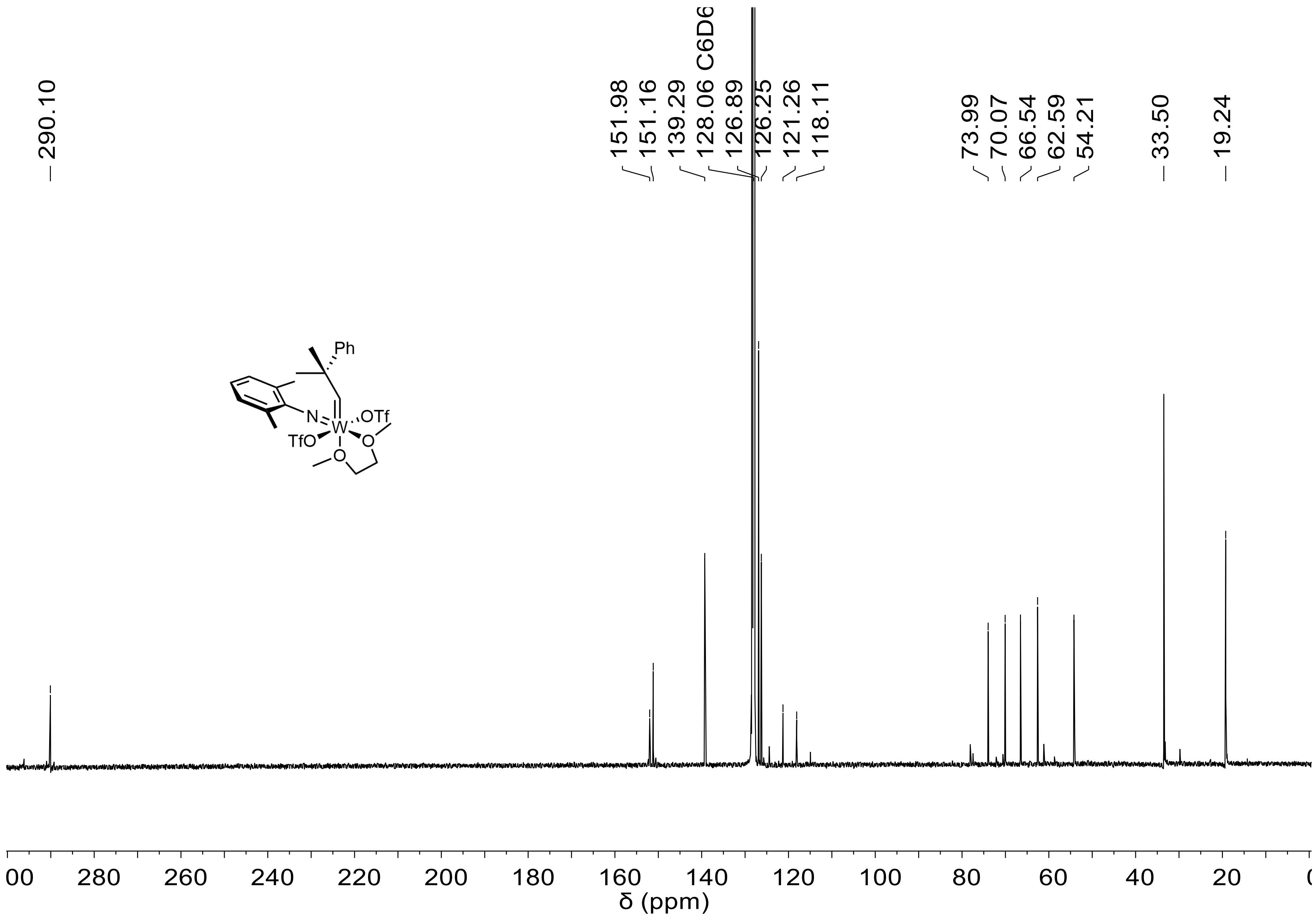

Figure S18: ^{13}C-NMR (101 MHz, 25 °C, C_6D_6) of W(N-2,6-$Me_2C_6H_3$)($CHCMe_2Ph$)($OTf)_2$(DME). The sample contained almost exclusively the *trans*-isomer.

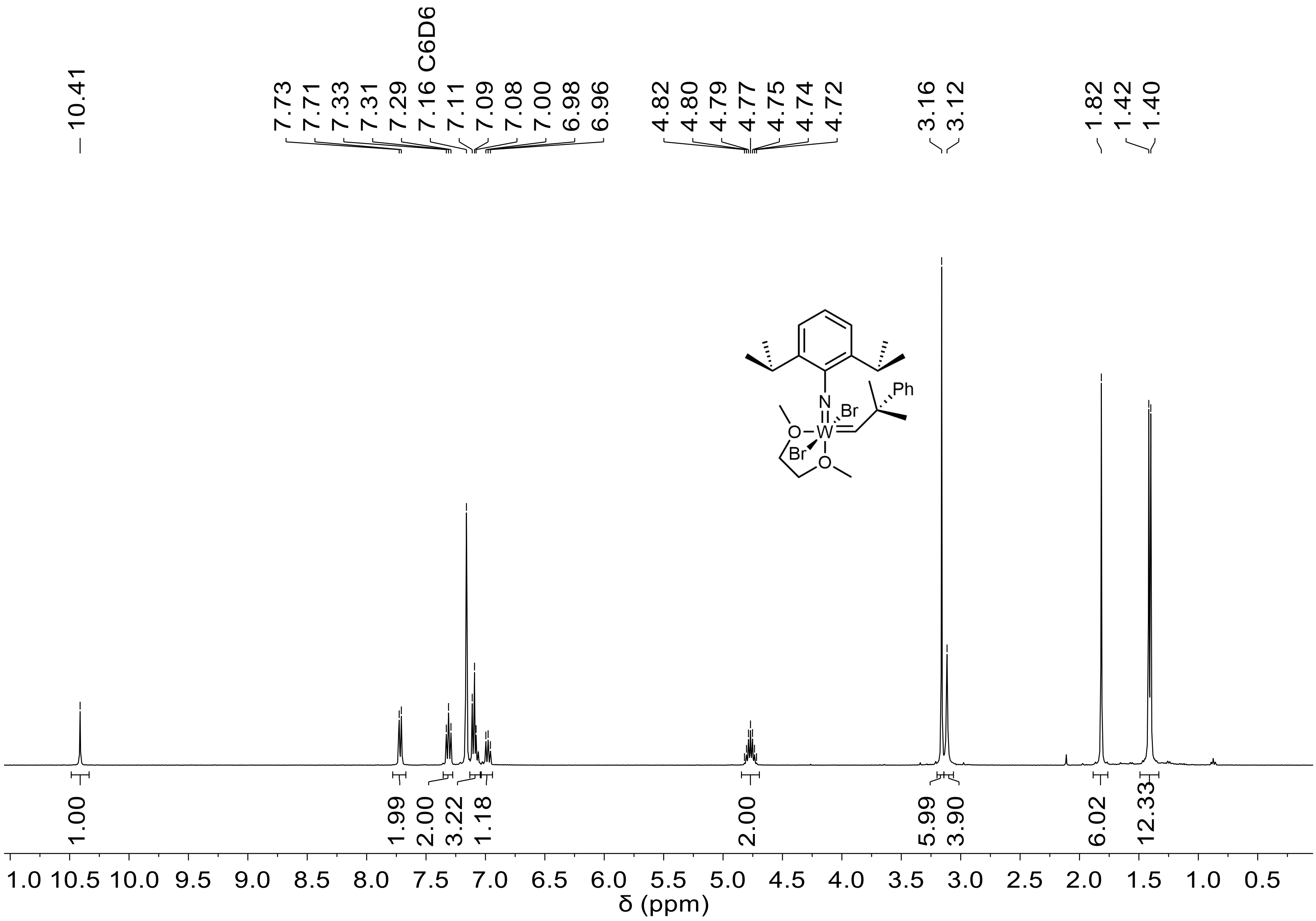

Figure S19: ^{1}H-NMR (400 MHz, 25 °C, C_6D_6) spectrum of W-01.

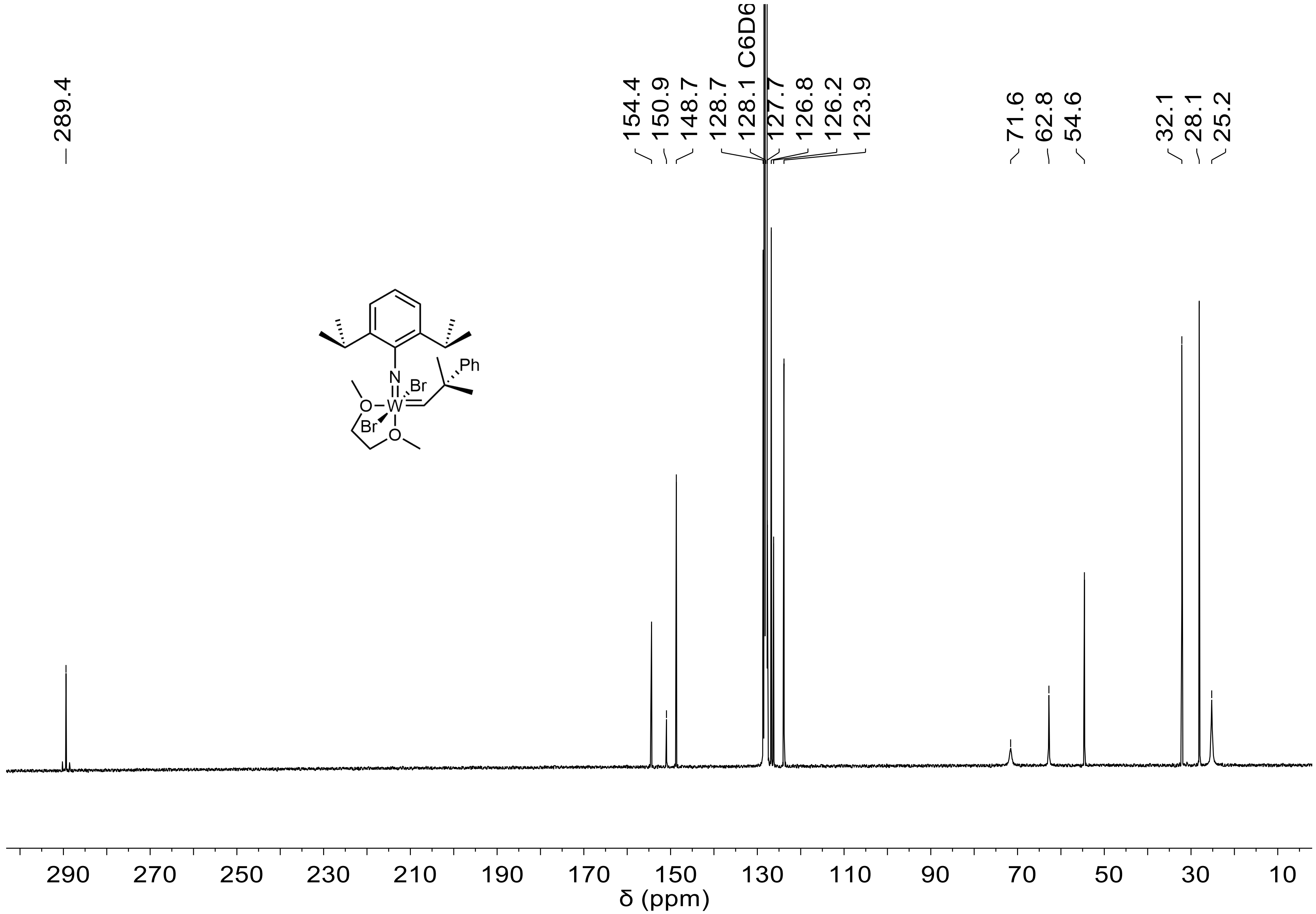

Figure S20: ^{13}C-NMR (101 MHz, 25 °C, C_6D_6) of W-01.

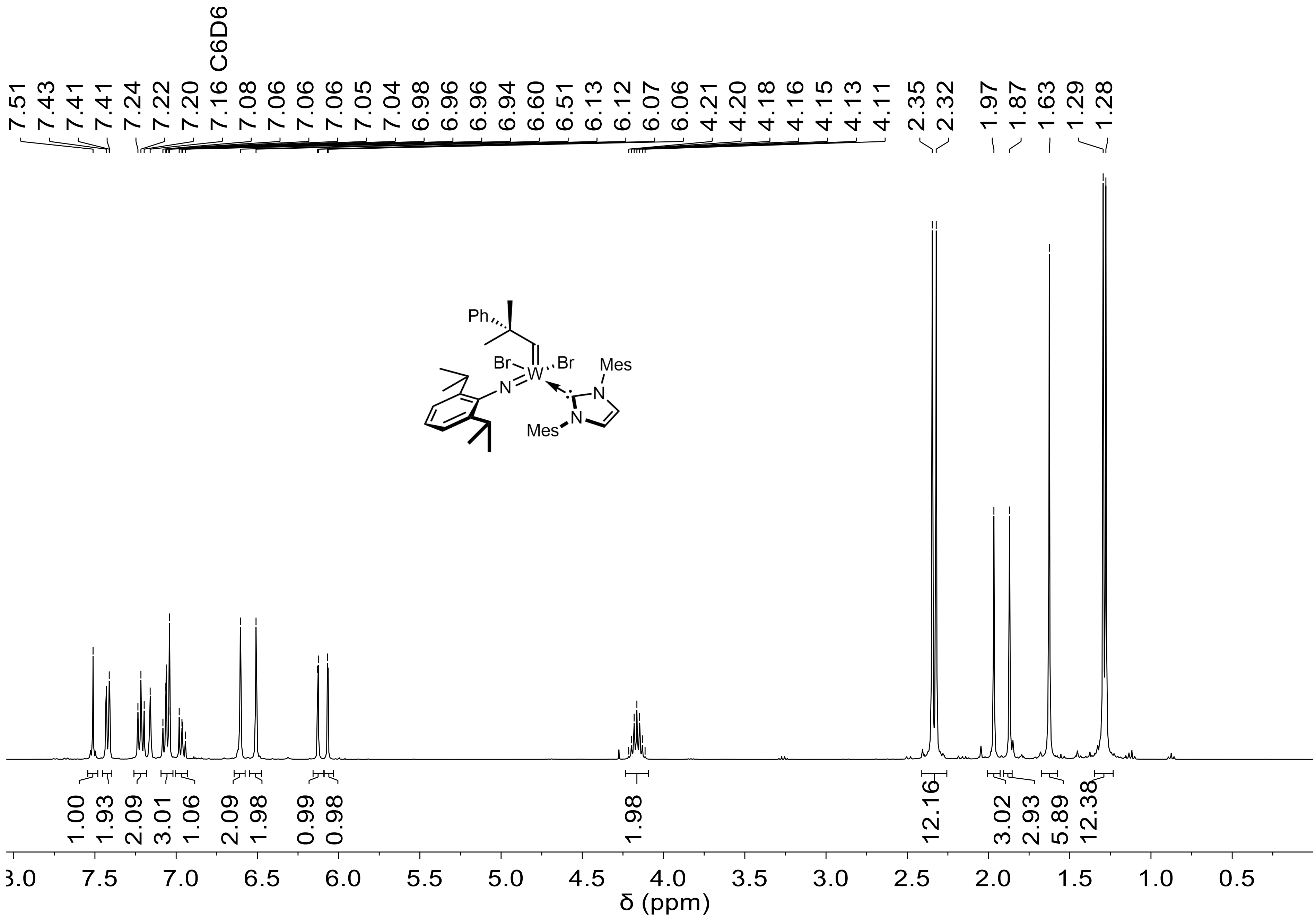

Figure S21: ^{1}H-NMR (400 MHz, 25 °C, C_6D_6) spectrum of W-02.

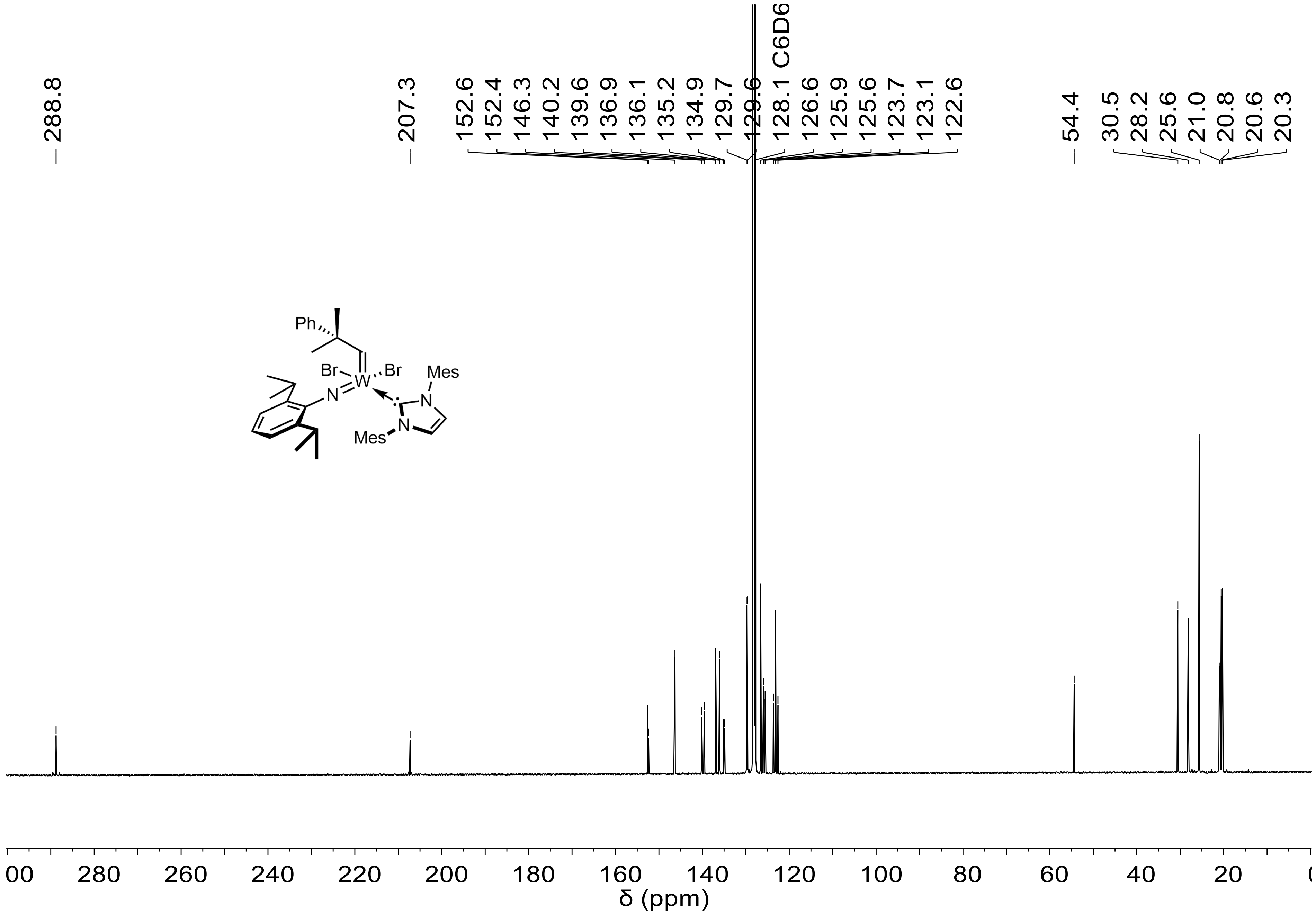

Figure S22: ^{13}C-NMR (101 MHz, 25 °C, C_6D_6) of W-02.

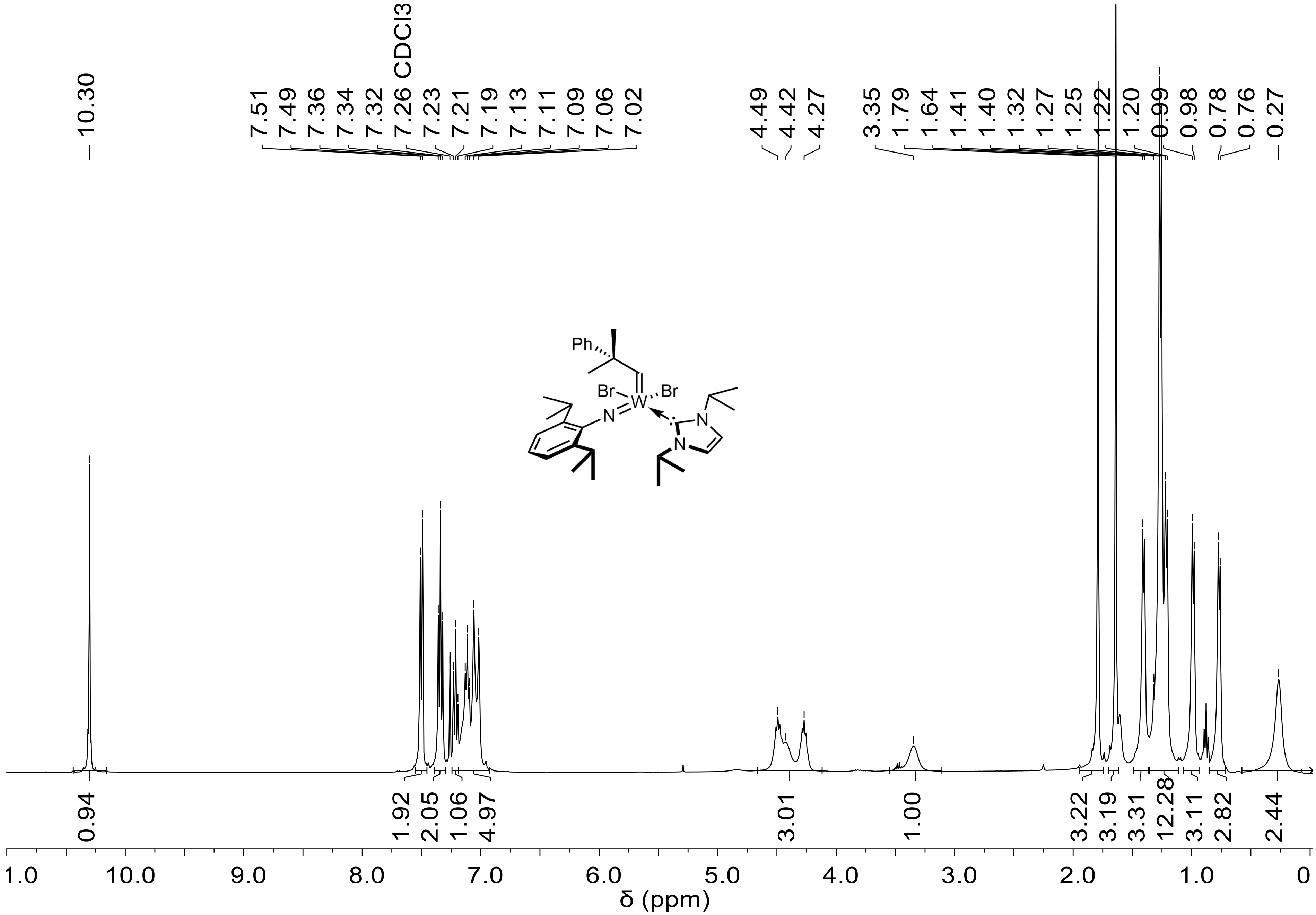

Figure S23: ¹H-NMR (400 MHz, 25 °C, $CDCl_3$) of W-03.

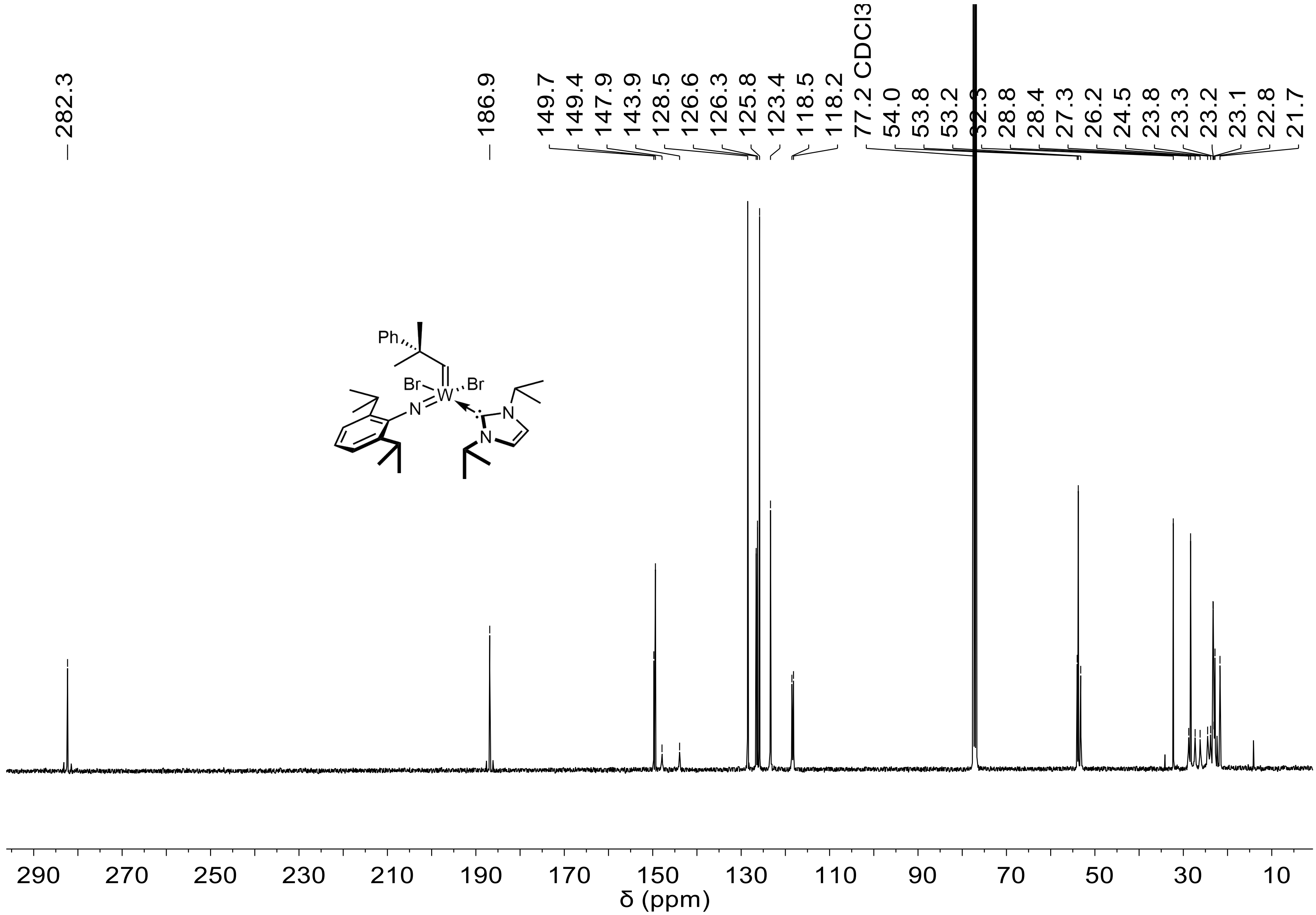

Figure S24: ^{13}C-NMR (101 MHz, 25 °C, $CDCl_3$) of W-03.

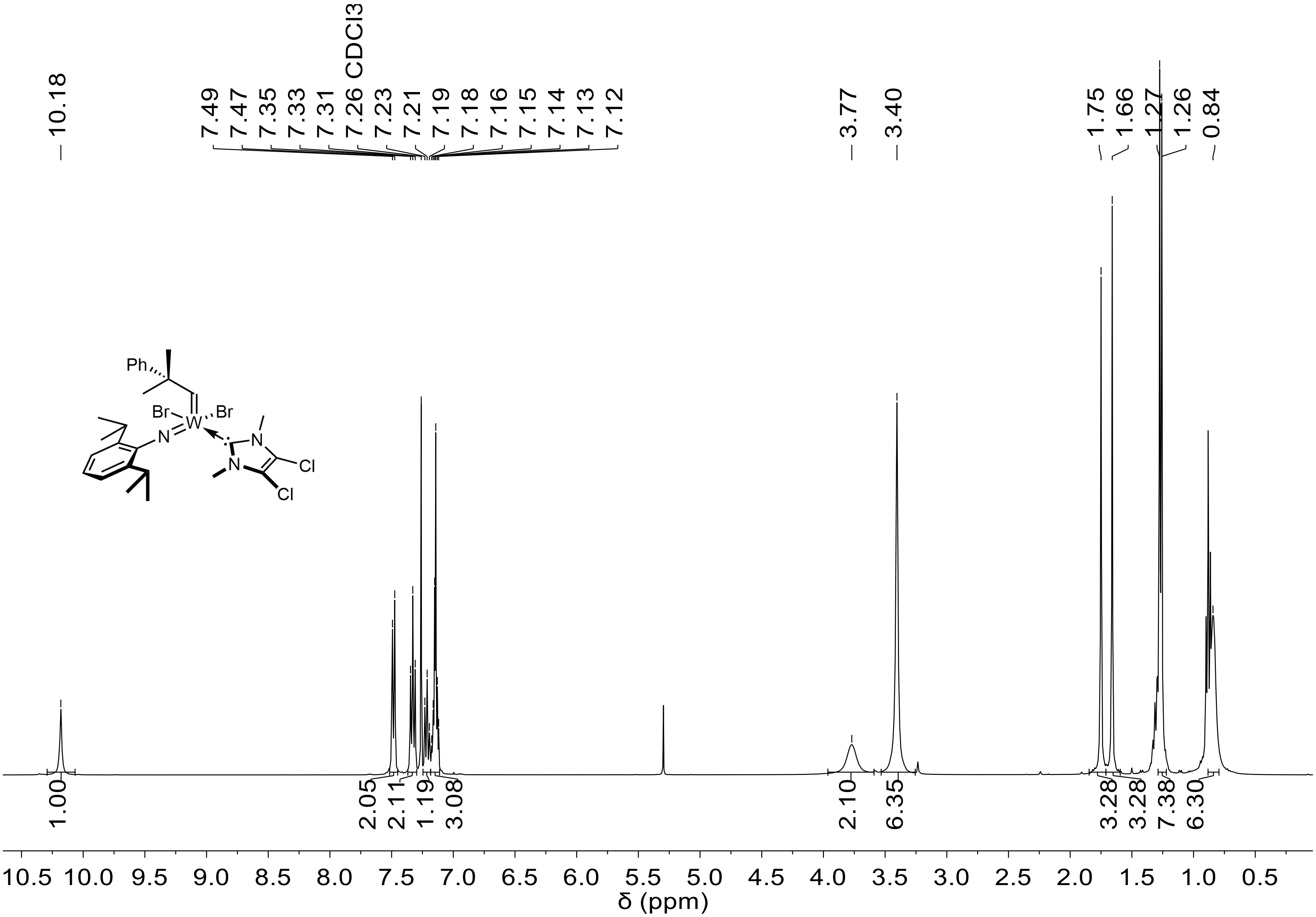

Figure S25: ^{1}H-NMR (400 MHz, 25 °C, $CDCl_3$) of W-04.

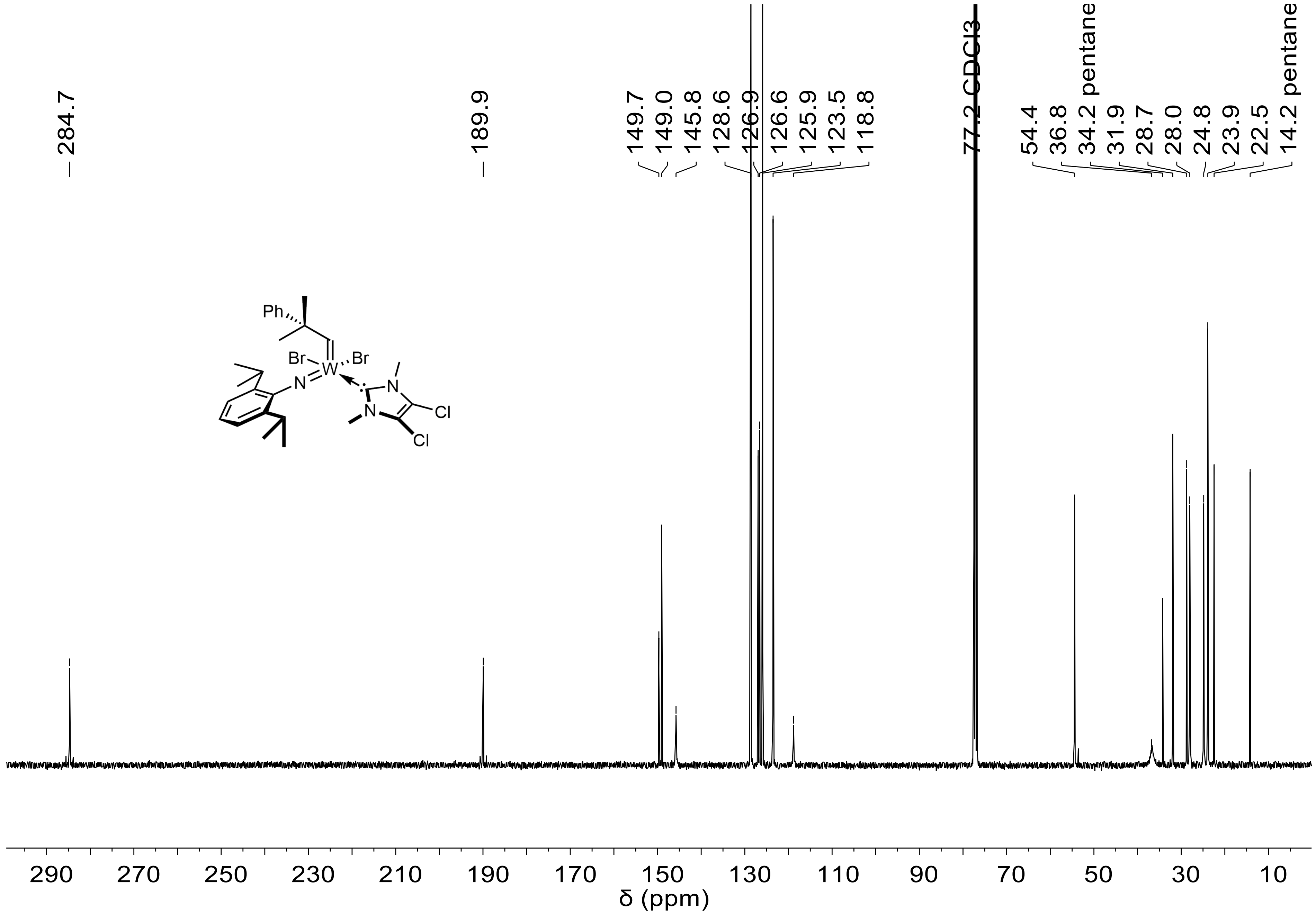

Figure S26: ^{13}C-NMR (101 MHz, 25 °C, $CDCl_3$) of W-04.

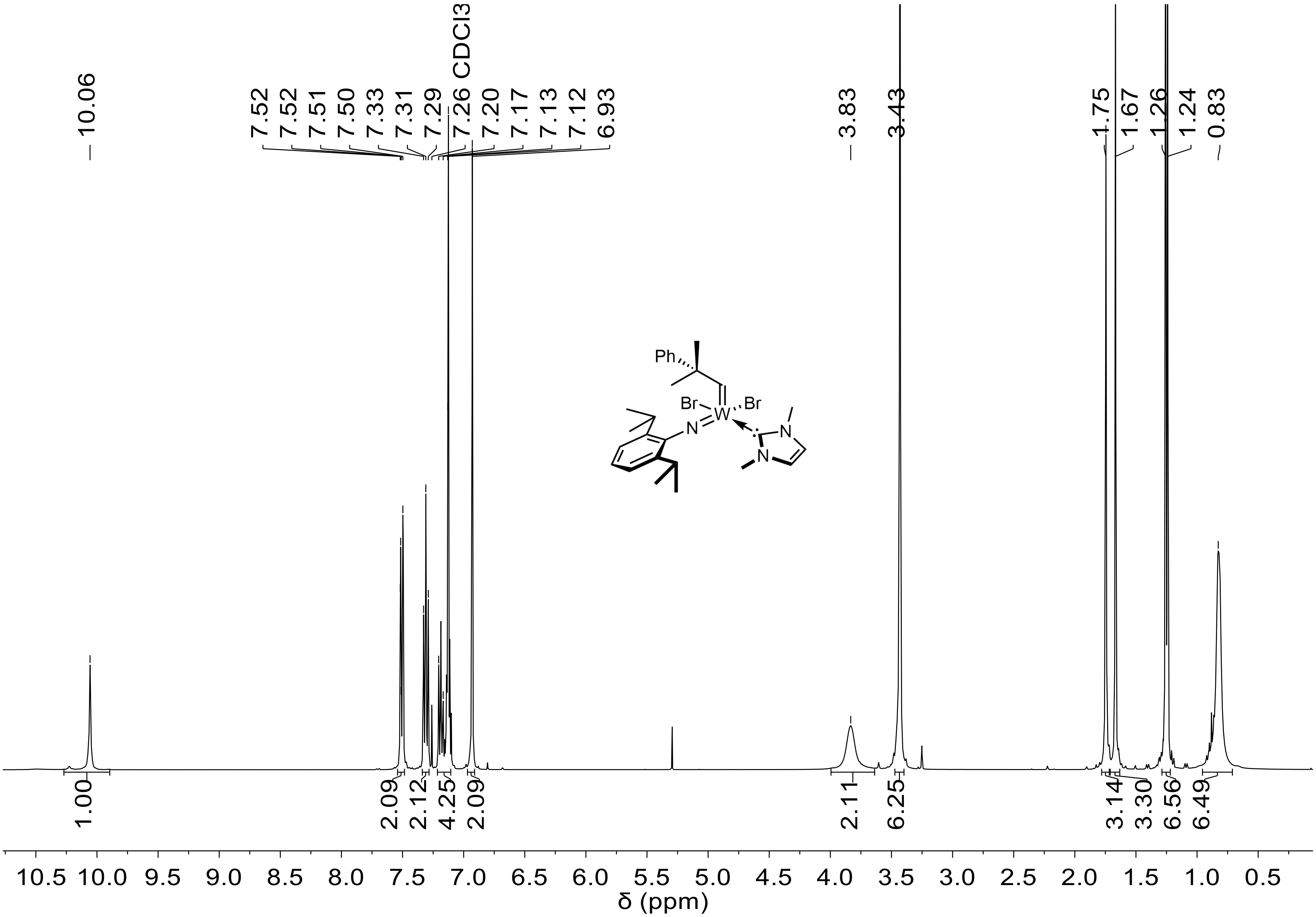

Figure S27: ^{1}H-NMR (400 MHz, 25 °C, $CDCl_3$) of W-05.

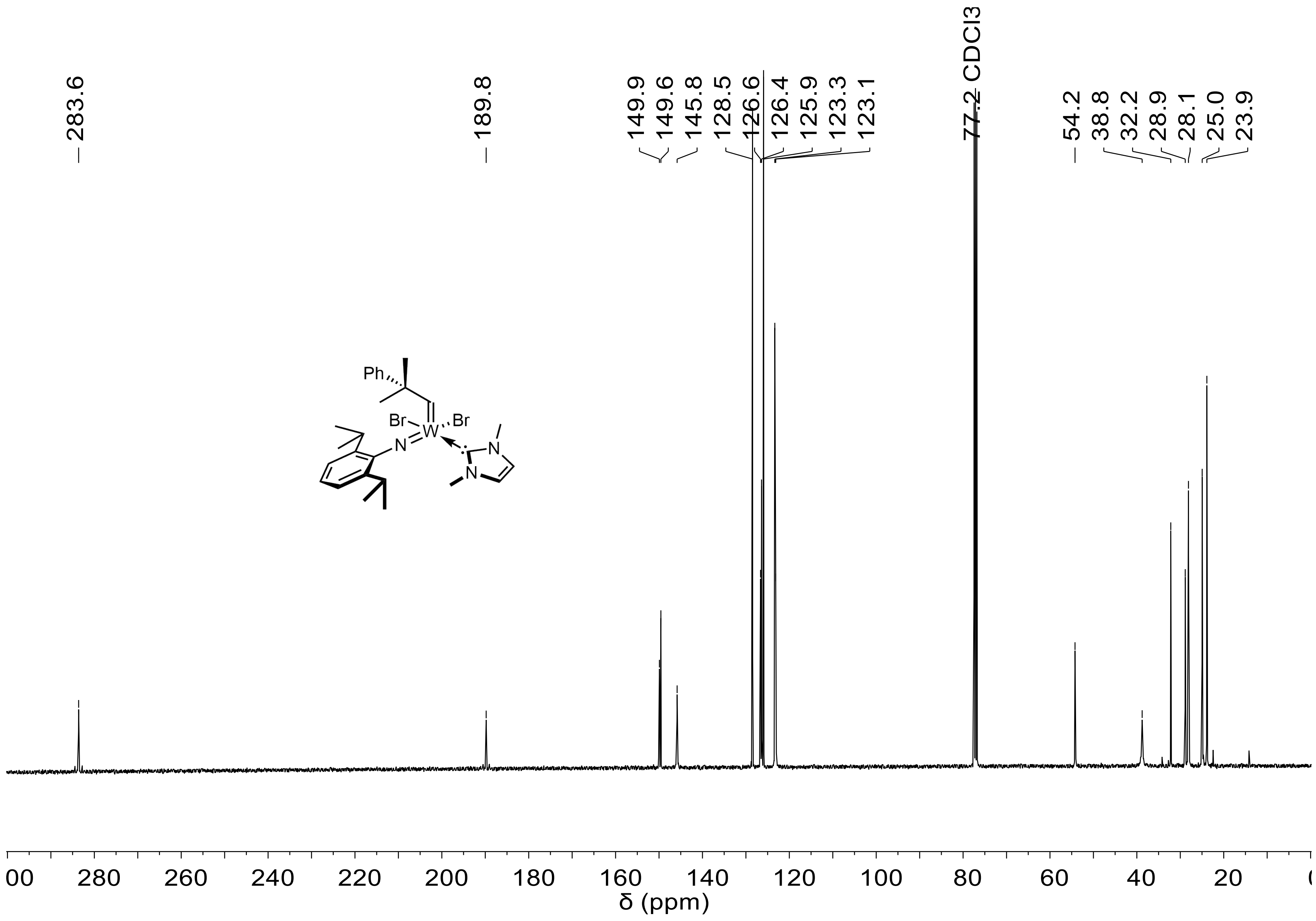

Figure S28: ^{13}C-NMR (101 MHz, 25 °C, $CDCl_3$) of W-05.

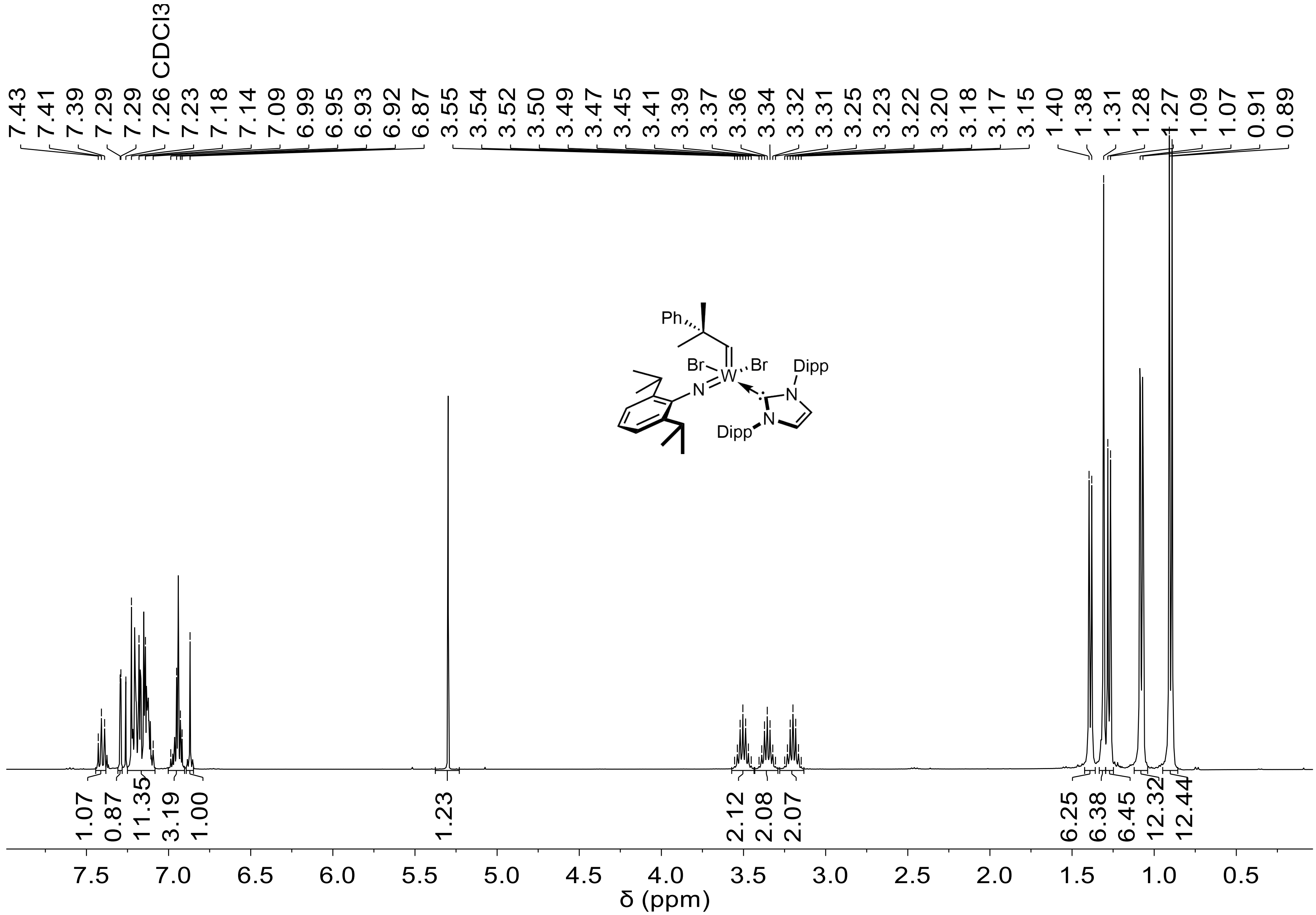

Figure S29: ^{1}H-NMR (400 MHz, 25 °C, $CDCl_3$) of W-06.

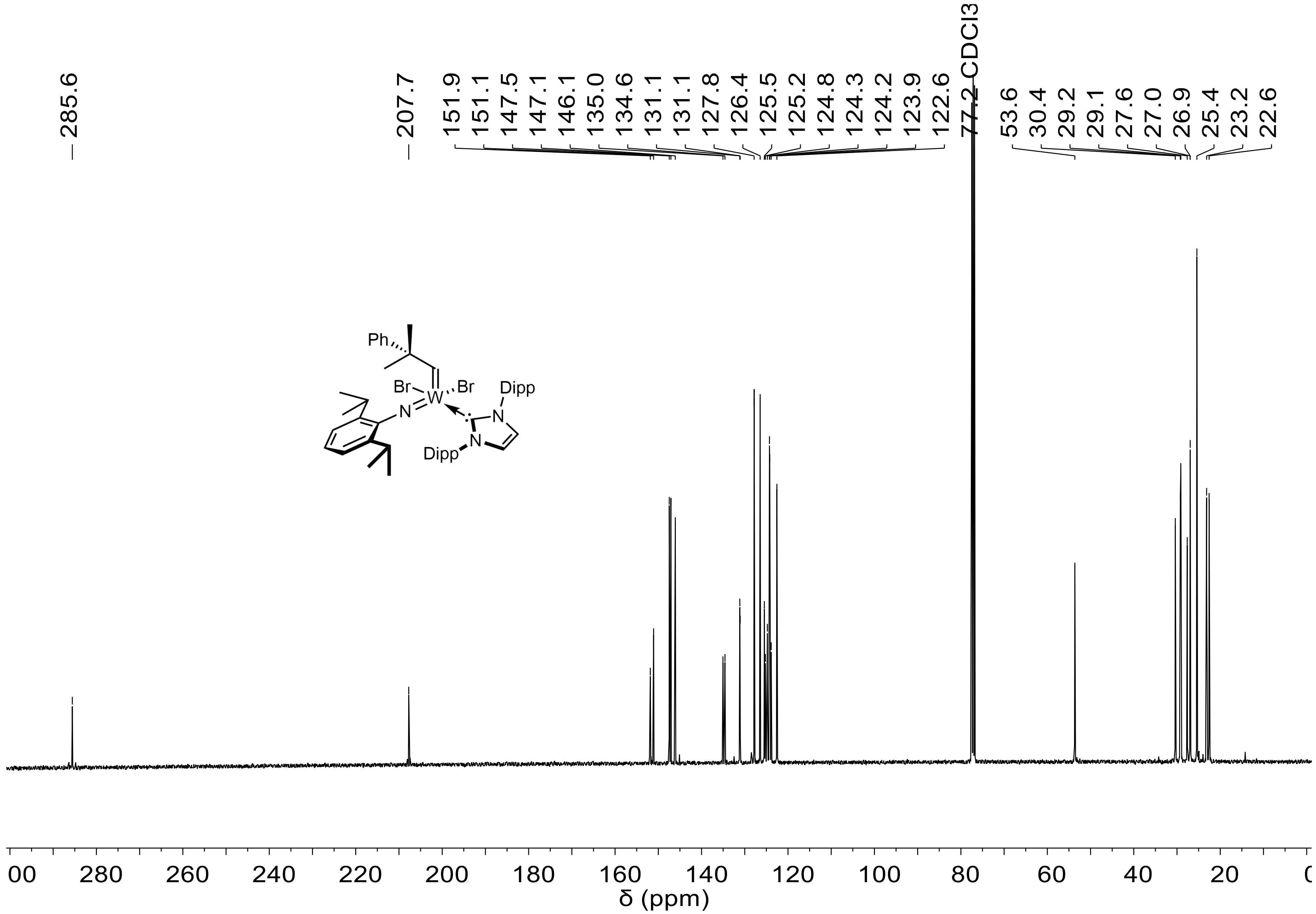

Figure S30: ^{13}C-NMR (101 MHz, 25 °C, $CDCl_3$) of W-06.

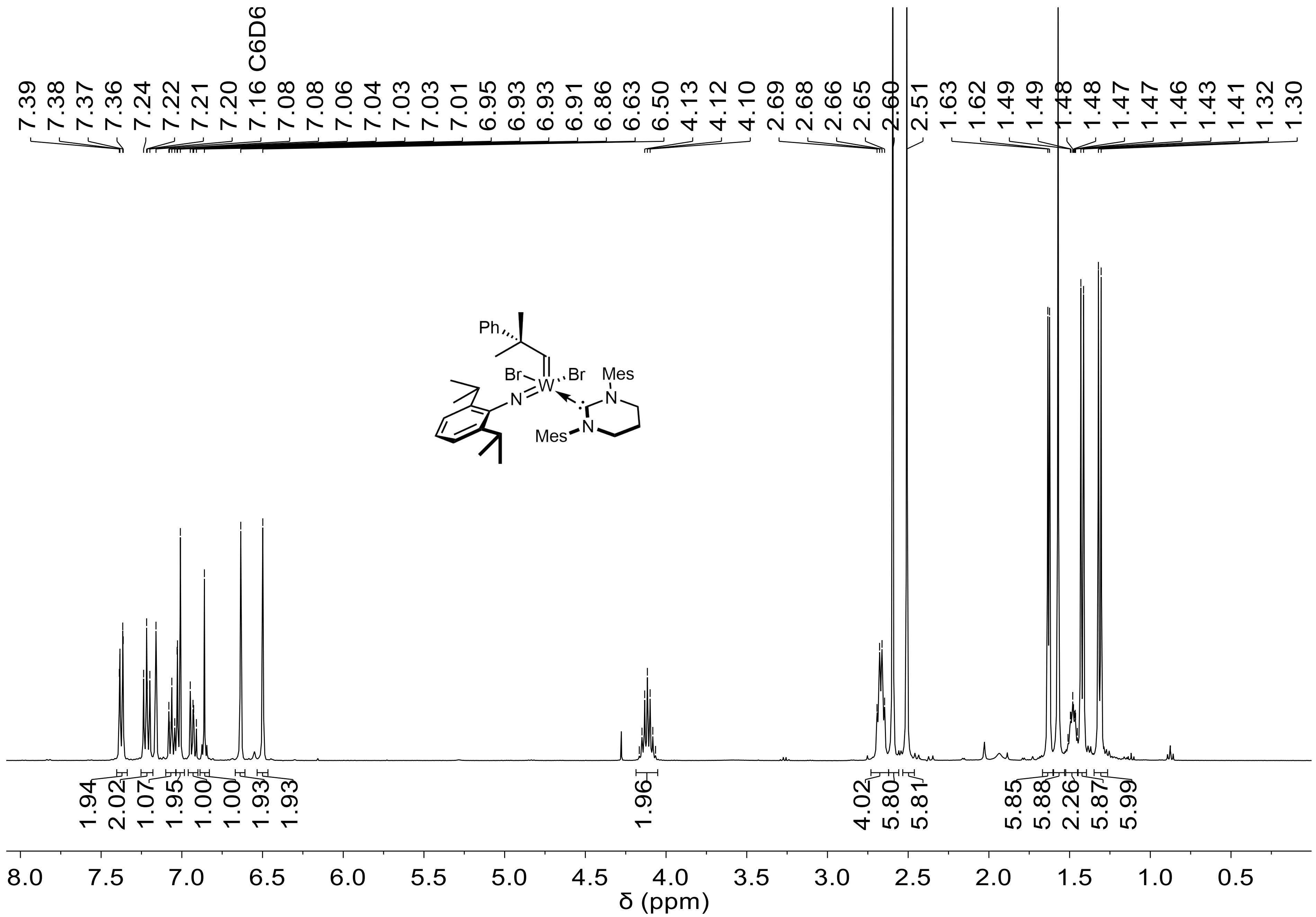

Figure S31: ^{1}H-NMR (400 MHz, 25 °C, C_6D_6) spectrum of W-07.

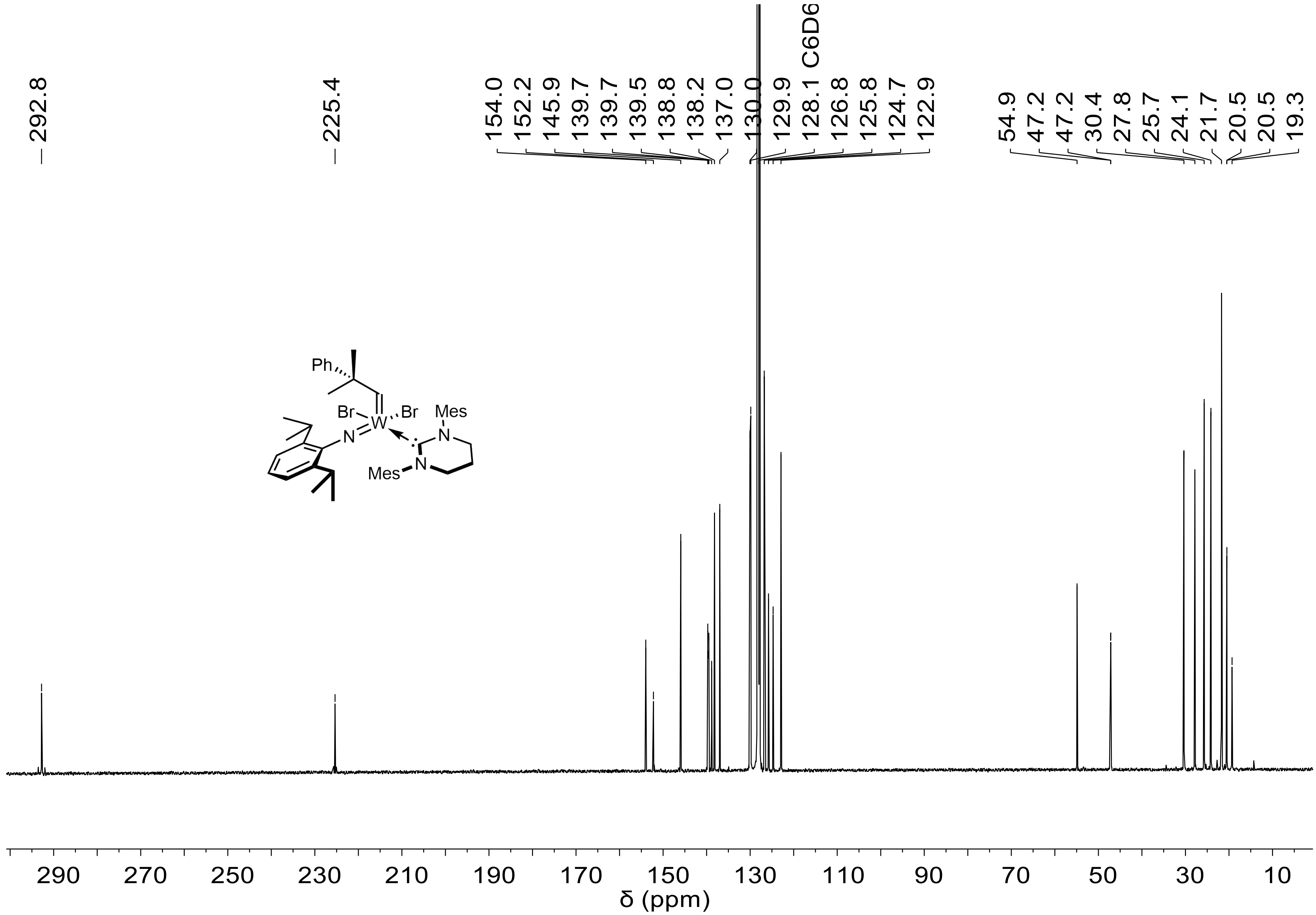

Figure S32: ^{13}C-NMR (101 MHz, 25 °C, C_6D_6) of W-07.

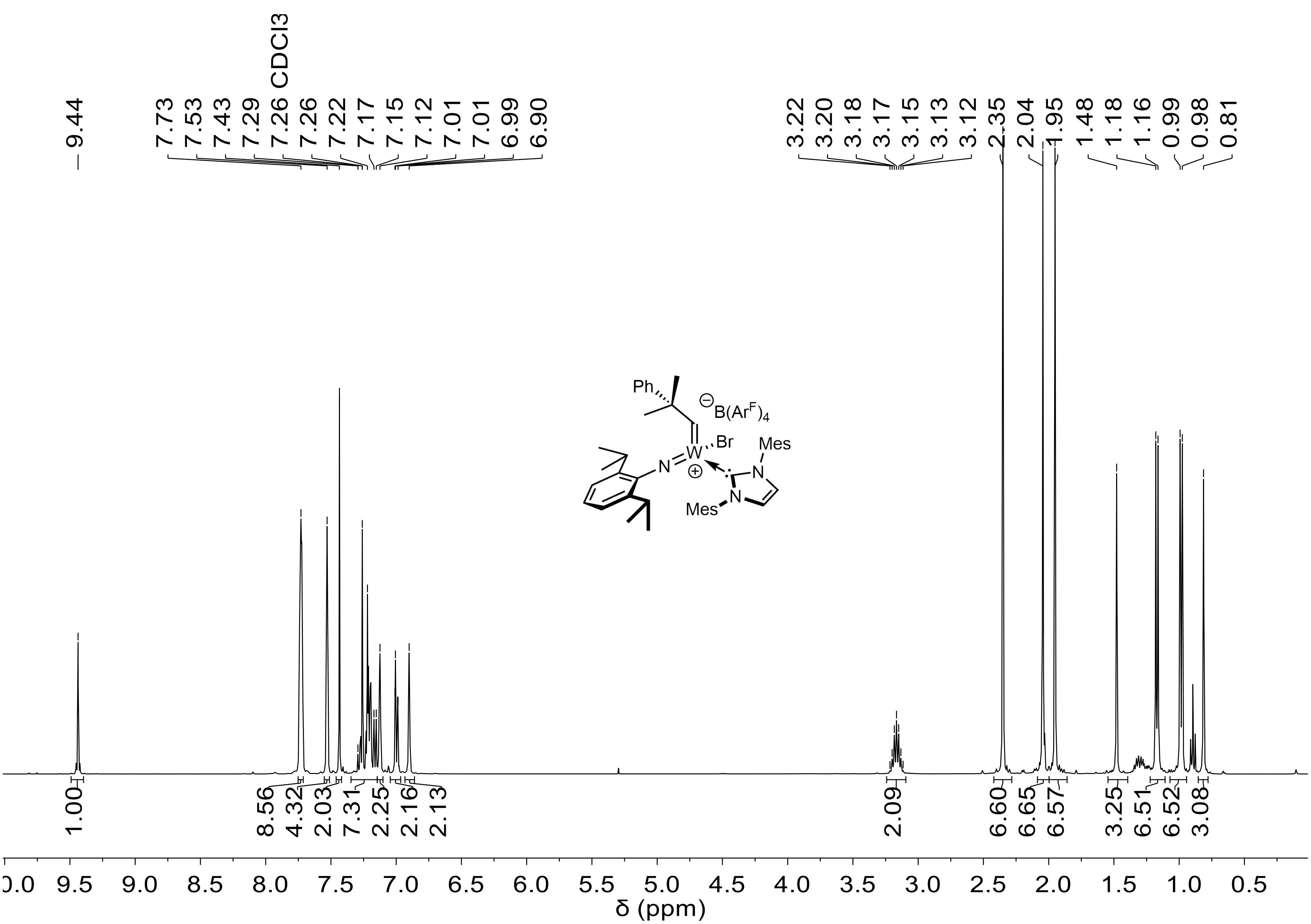

Figure S33: ^{1}H-NMR (400 MHz, 25 °C, CDCl$_3$) of W-08.

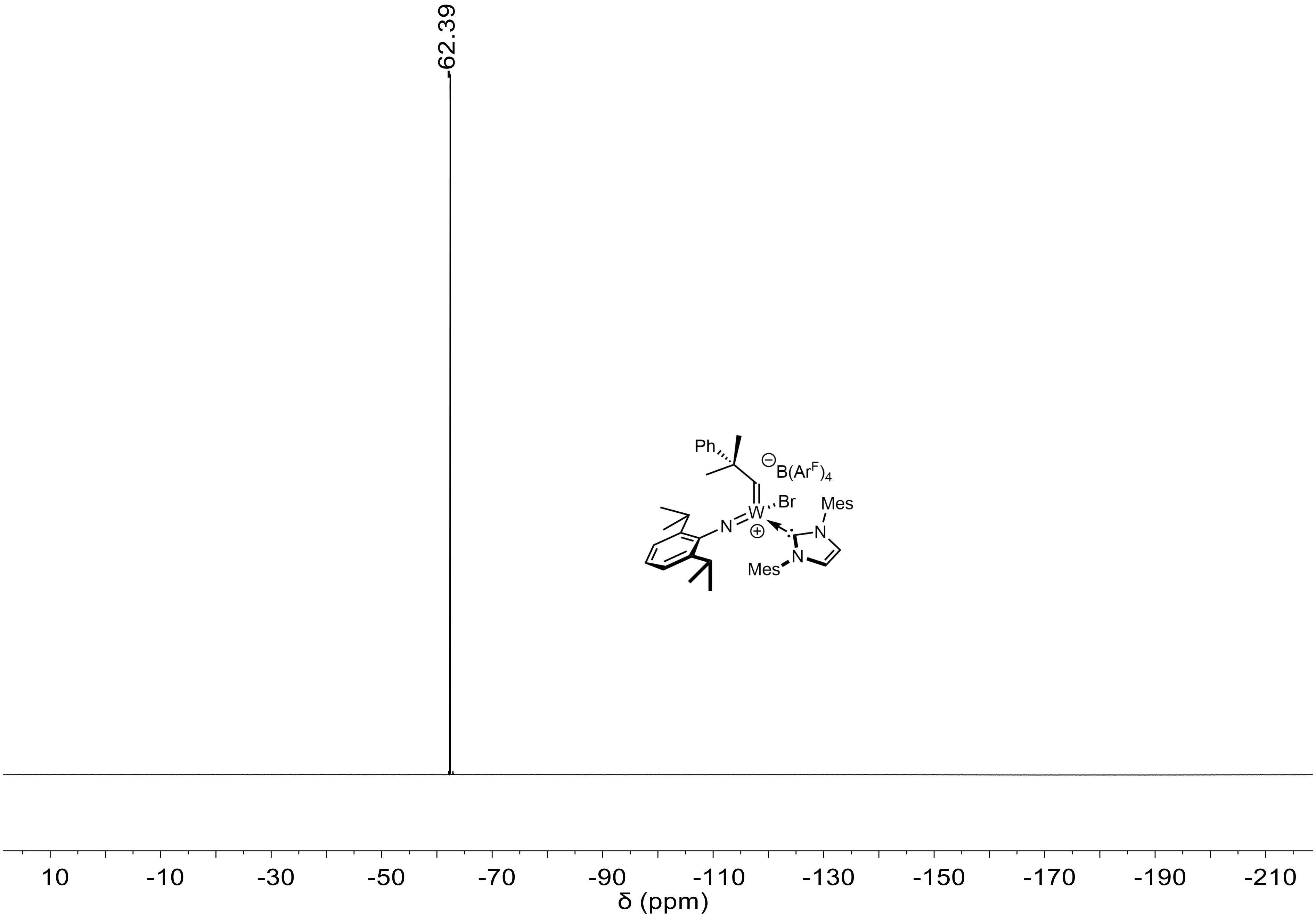

Figure S34: ^{19}F-NMR (376 MHz, 25 °C, $CDCl_3$) of W-08.

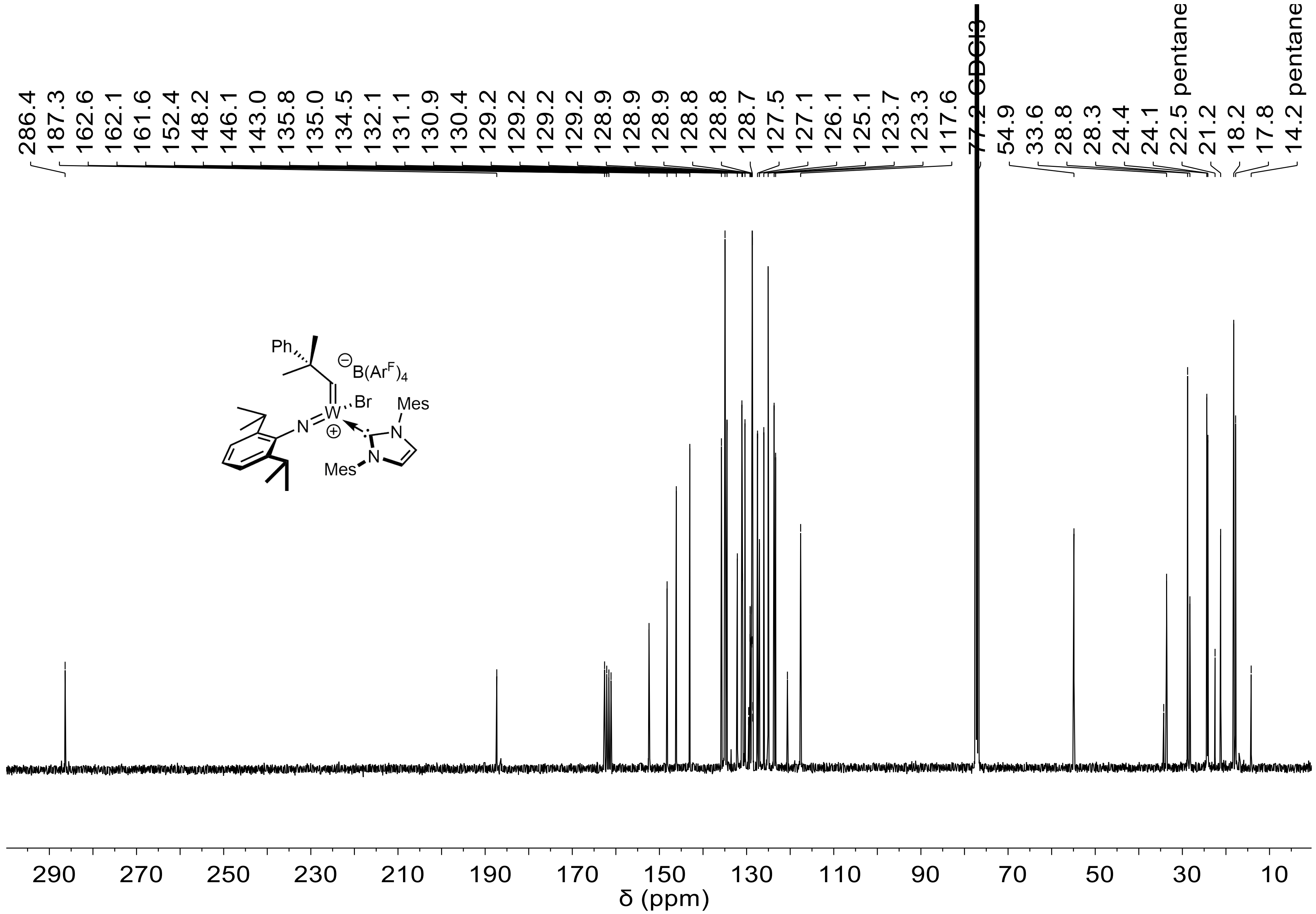

Figure S35: ^{13}C-NMR (101 MHz, 25 °C, $CDCl_3$) of W-08.

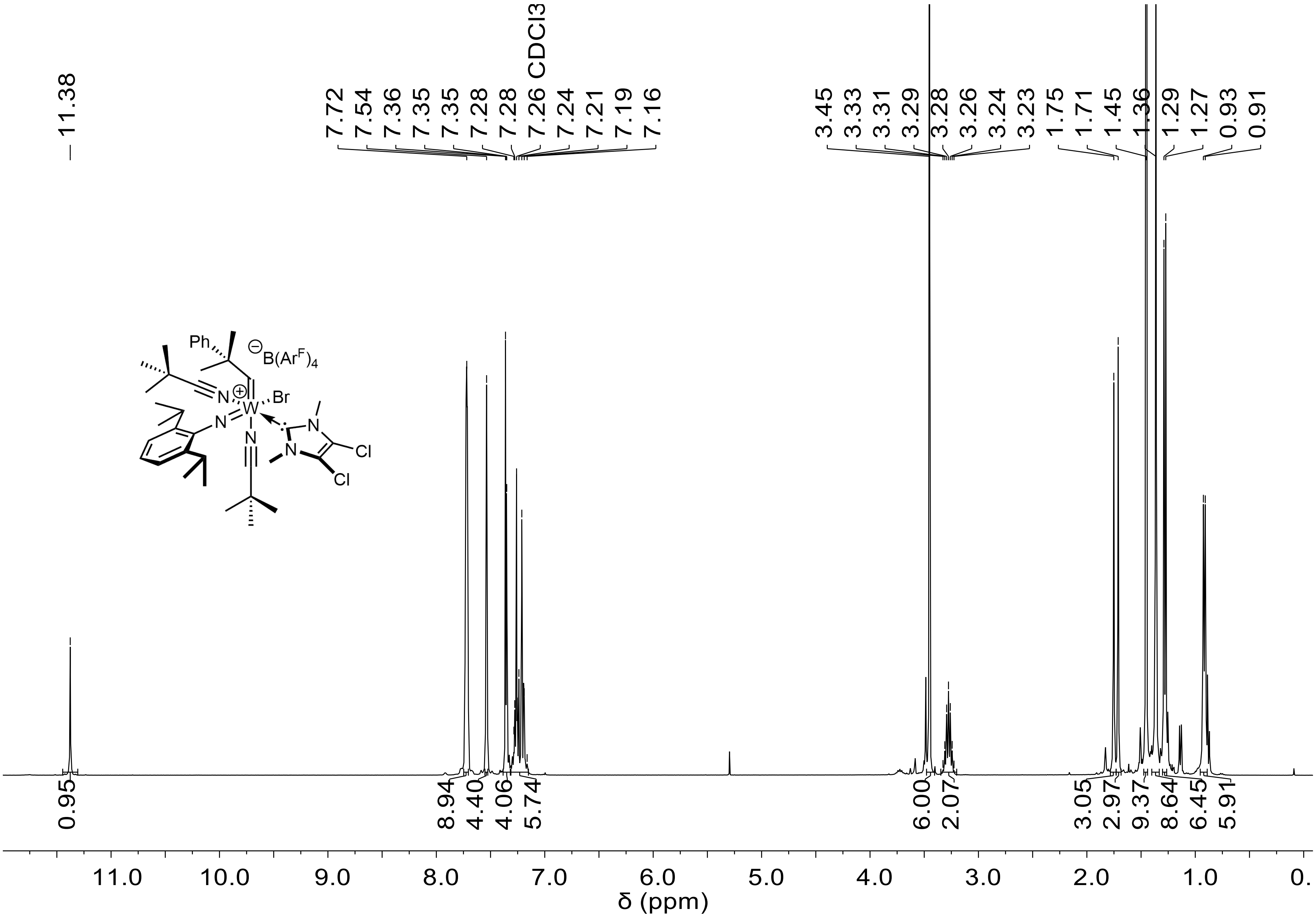

Figure S36: ^{1}H-NMR (400 MHz, 25 °C, $CDCl_3$) of W-09.

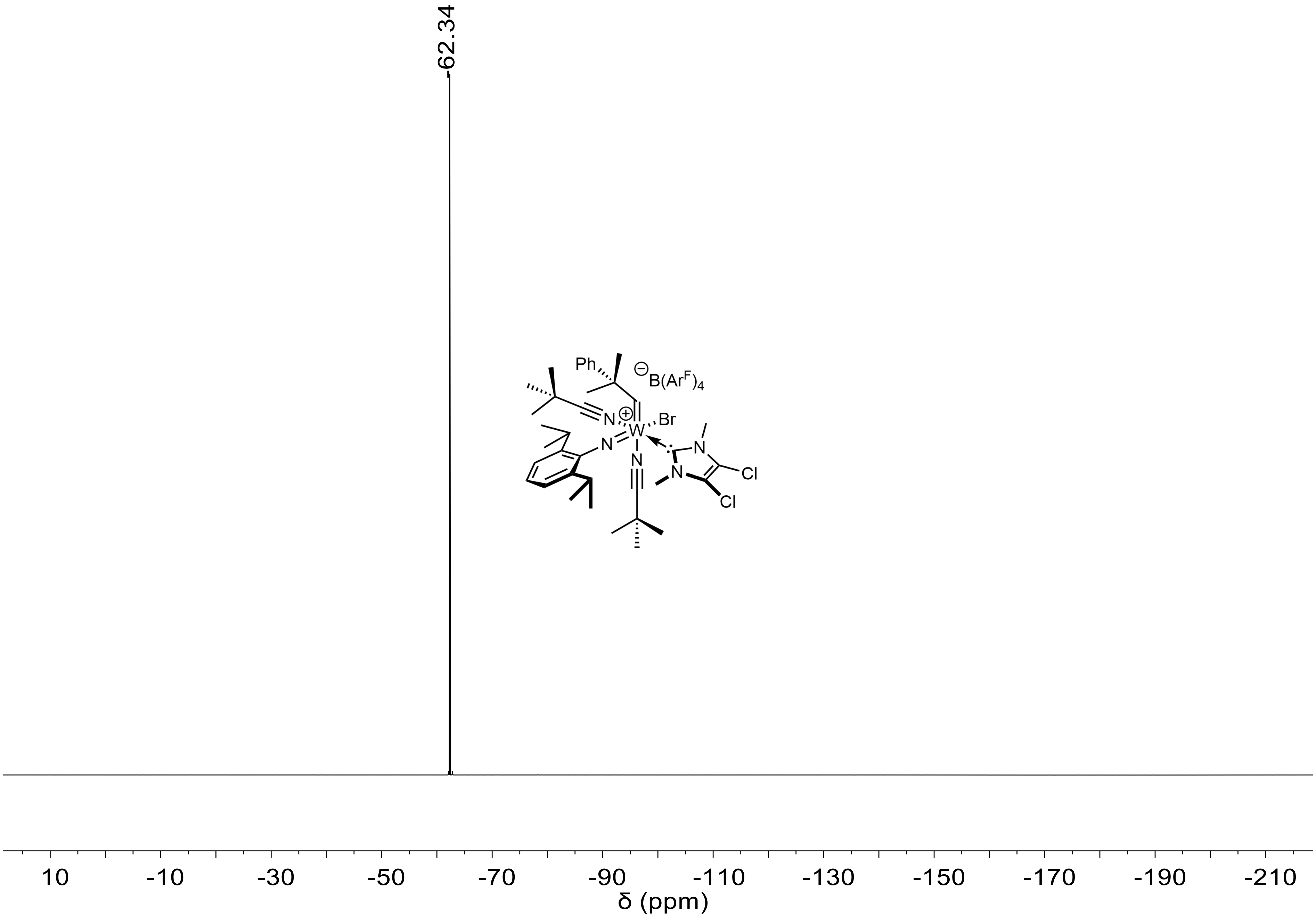

Figure S37: ^{19}F-NMR (376 MHz, 25 °C, $CDCl_3$) of W-09.

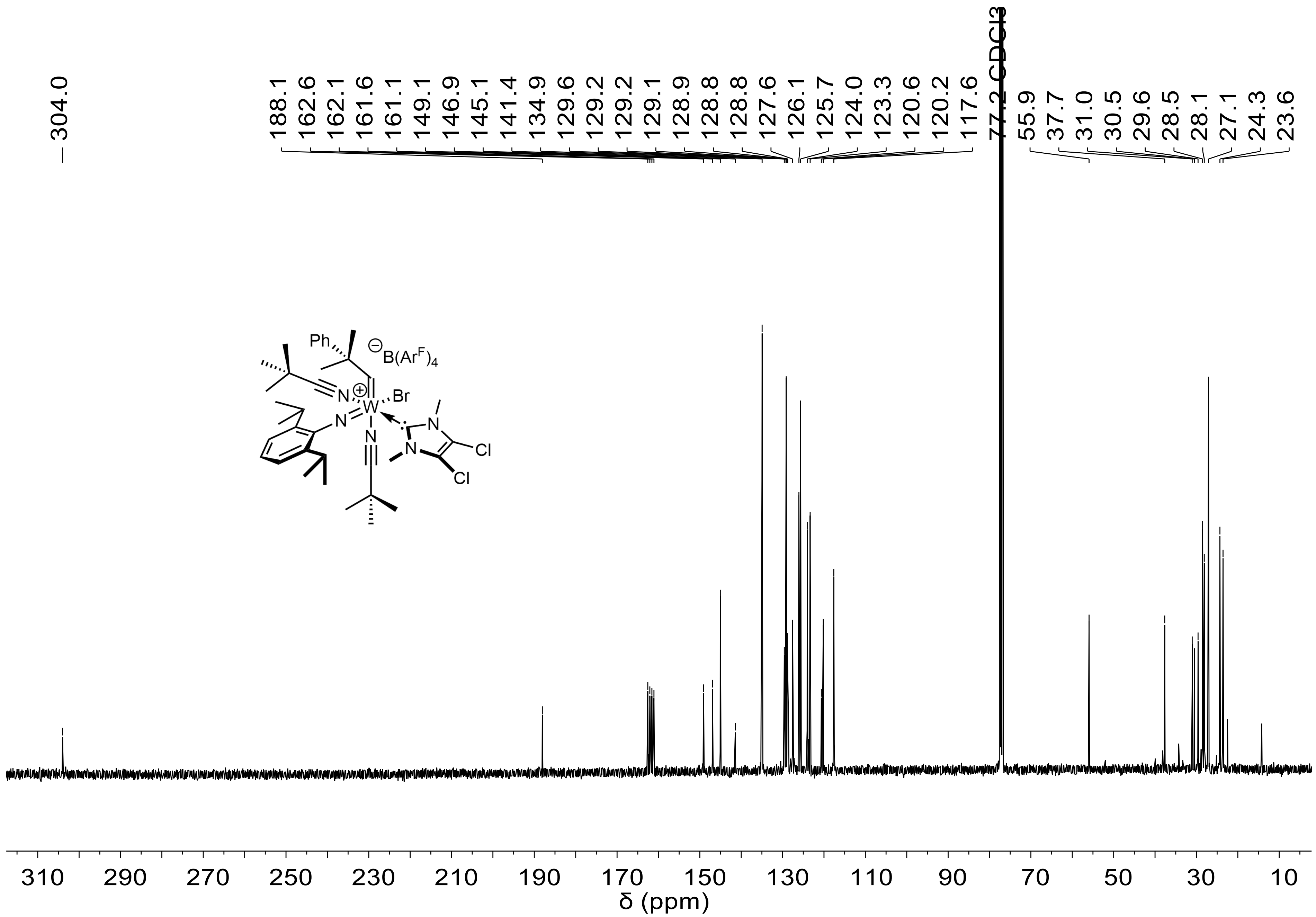

Figure S38: ^{13}C-NMR (101 MHz, 25 °C, CDCl$_3$) of W-09.

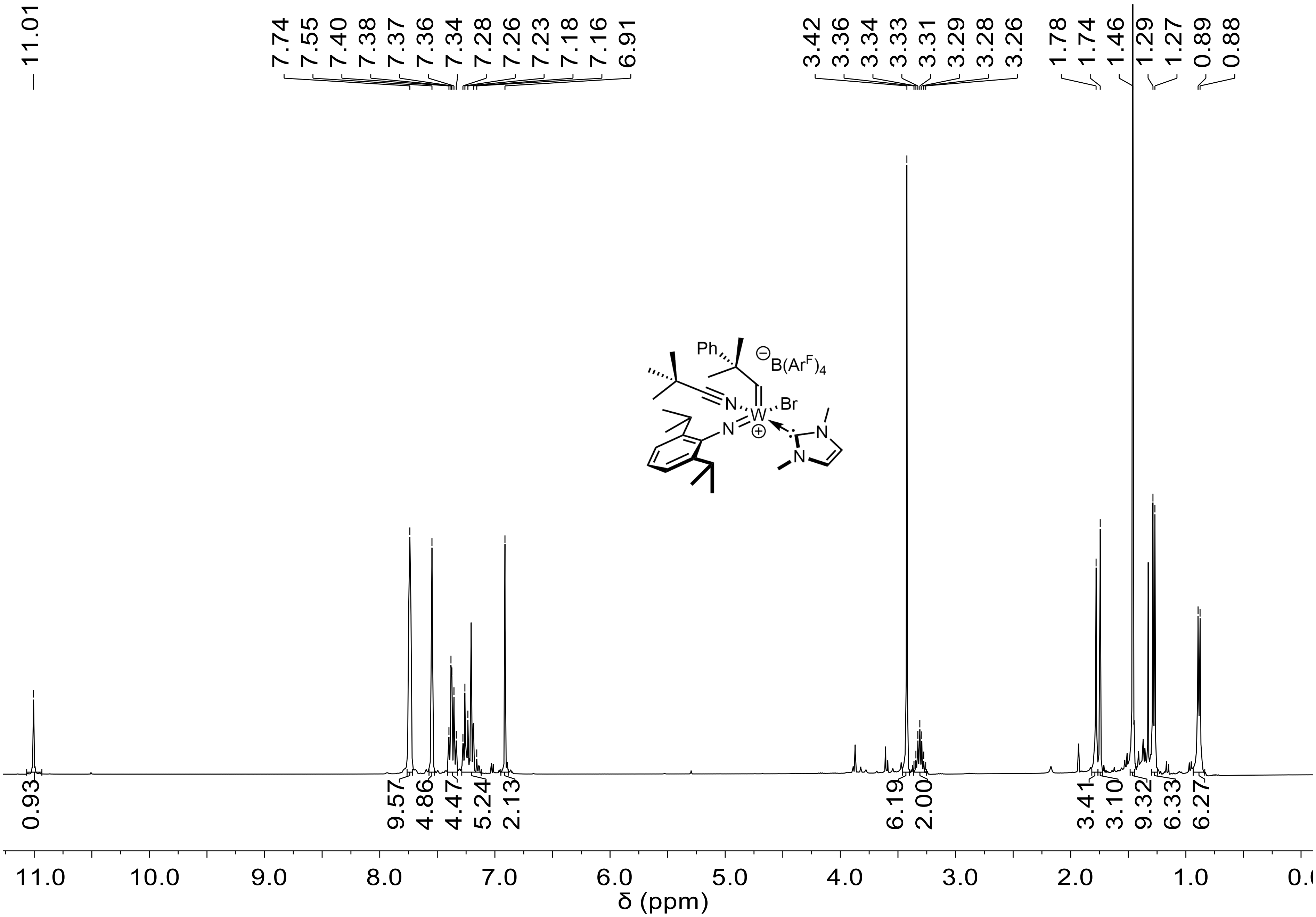

Figure S39: ^{1}H-NMR (400 MHz, 25 °C, $CDCl_3$) of W-10.

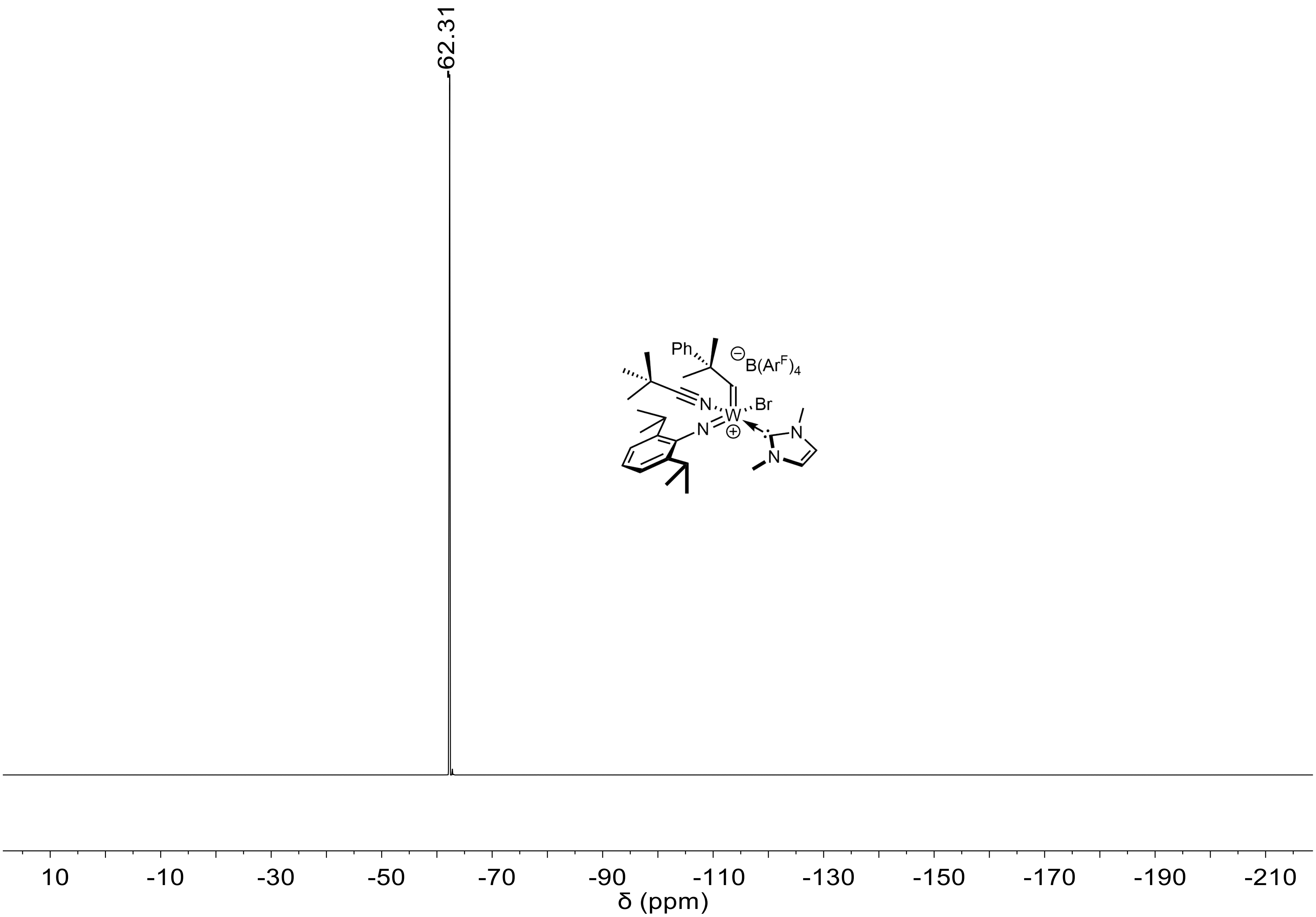

Figure S40: ^{19}F-NMR (376 MHz, 25 °C, $CDCl_3$) of W-10.

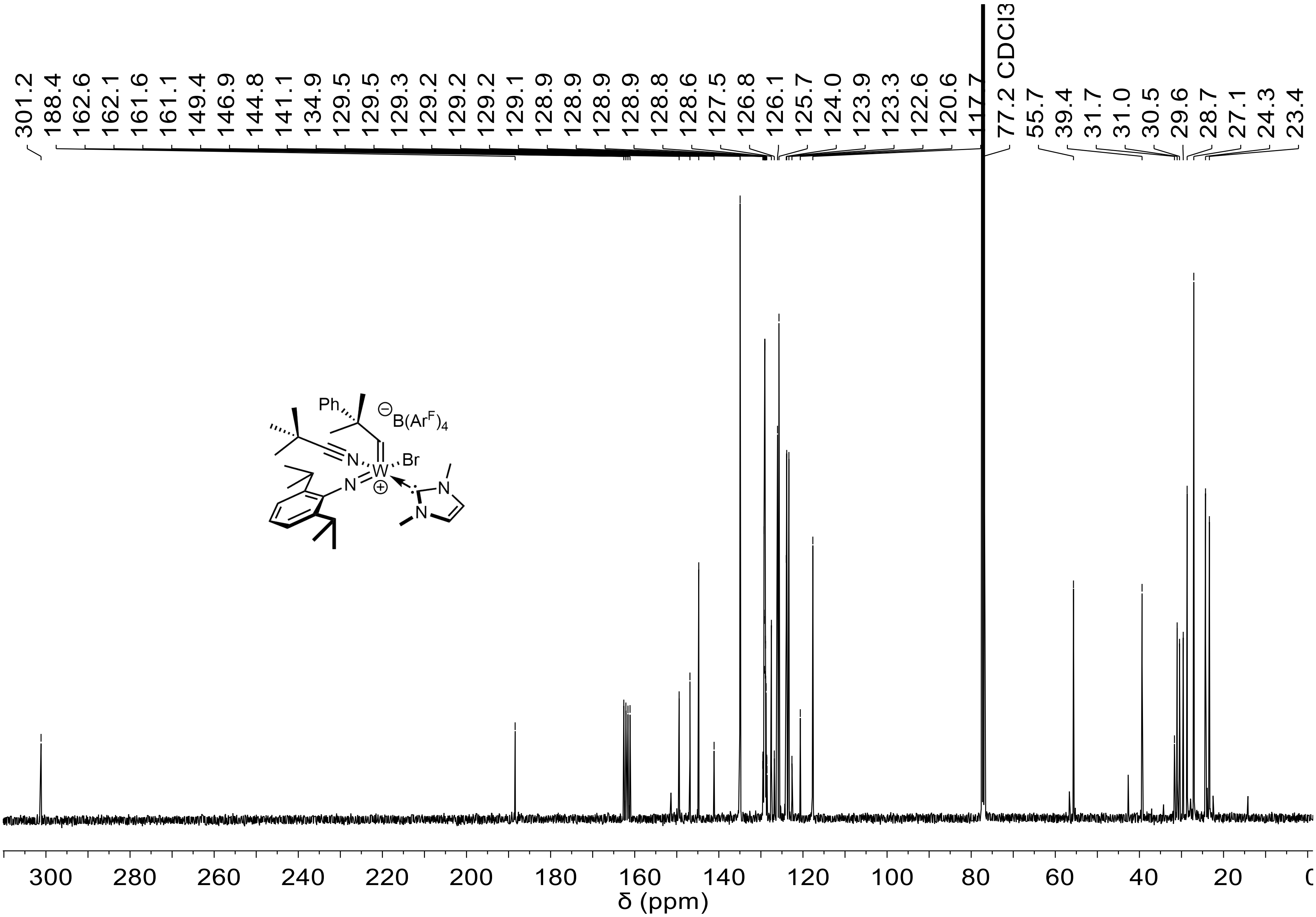

Figure S41: ^{13}C-NMR (101 MHz, 25 °C, $CDCl_3$) of W-10.

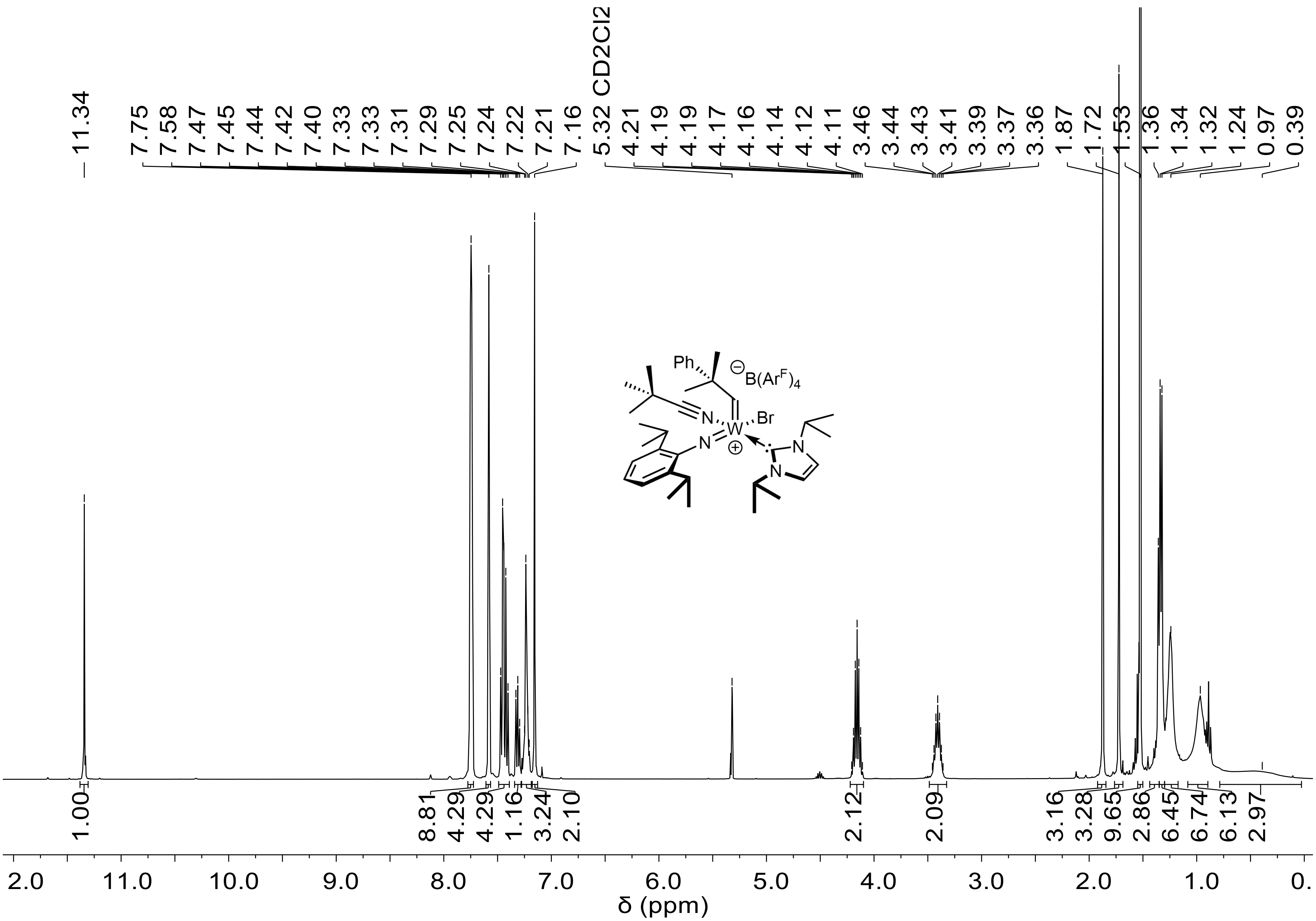

Figure S42: ^{1}H-NMR (400 MHz, 25 °C, CD_2Cl_2) of W-11.

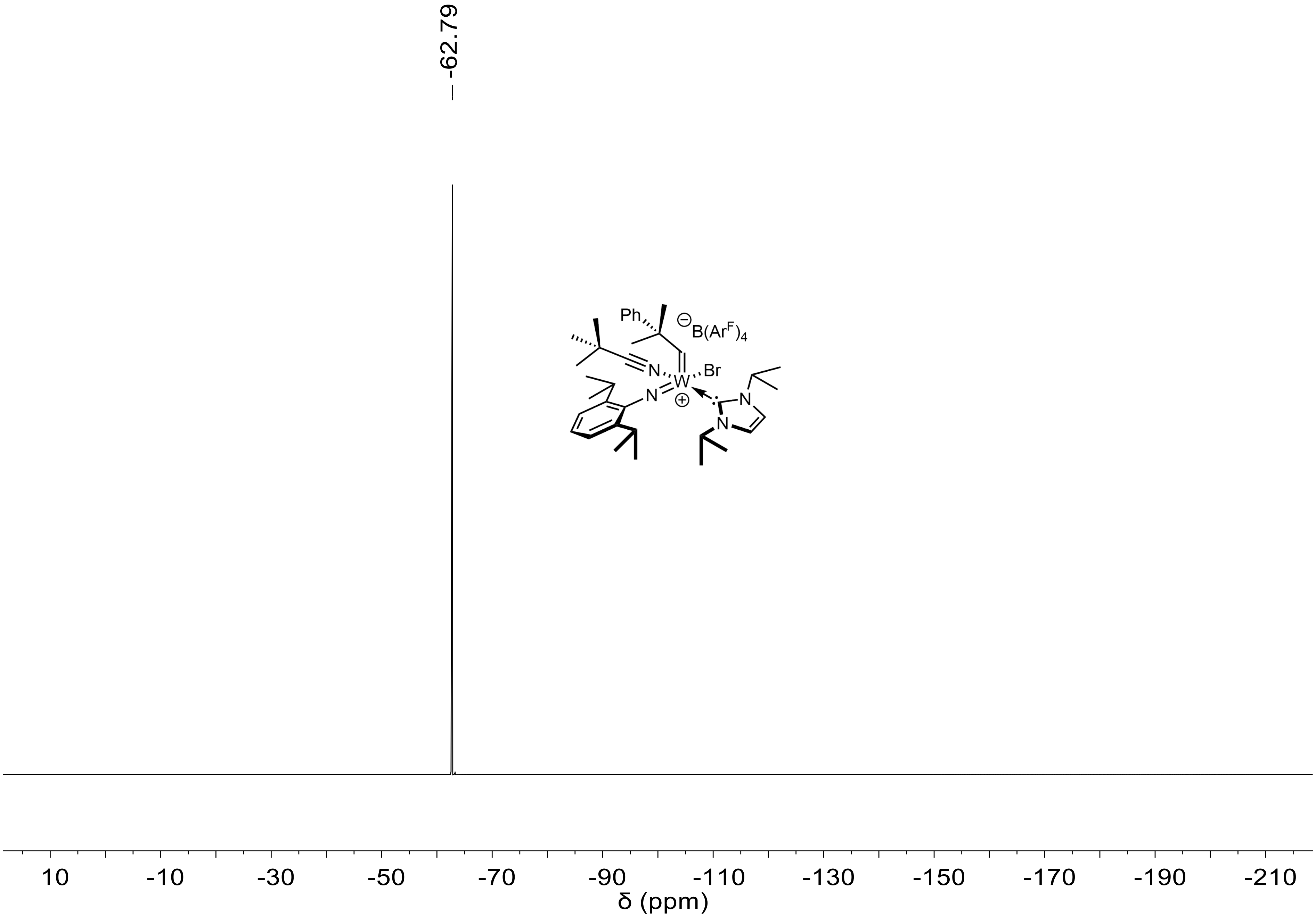

Figure S43: ^{19}F-NMR (376 MHz, 25 °C, CD_2Cl_2) of W-11.

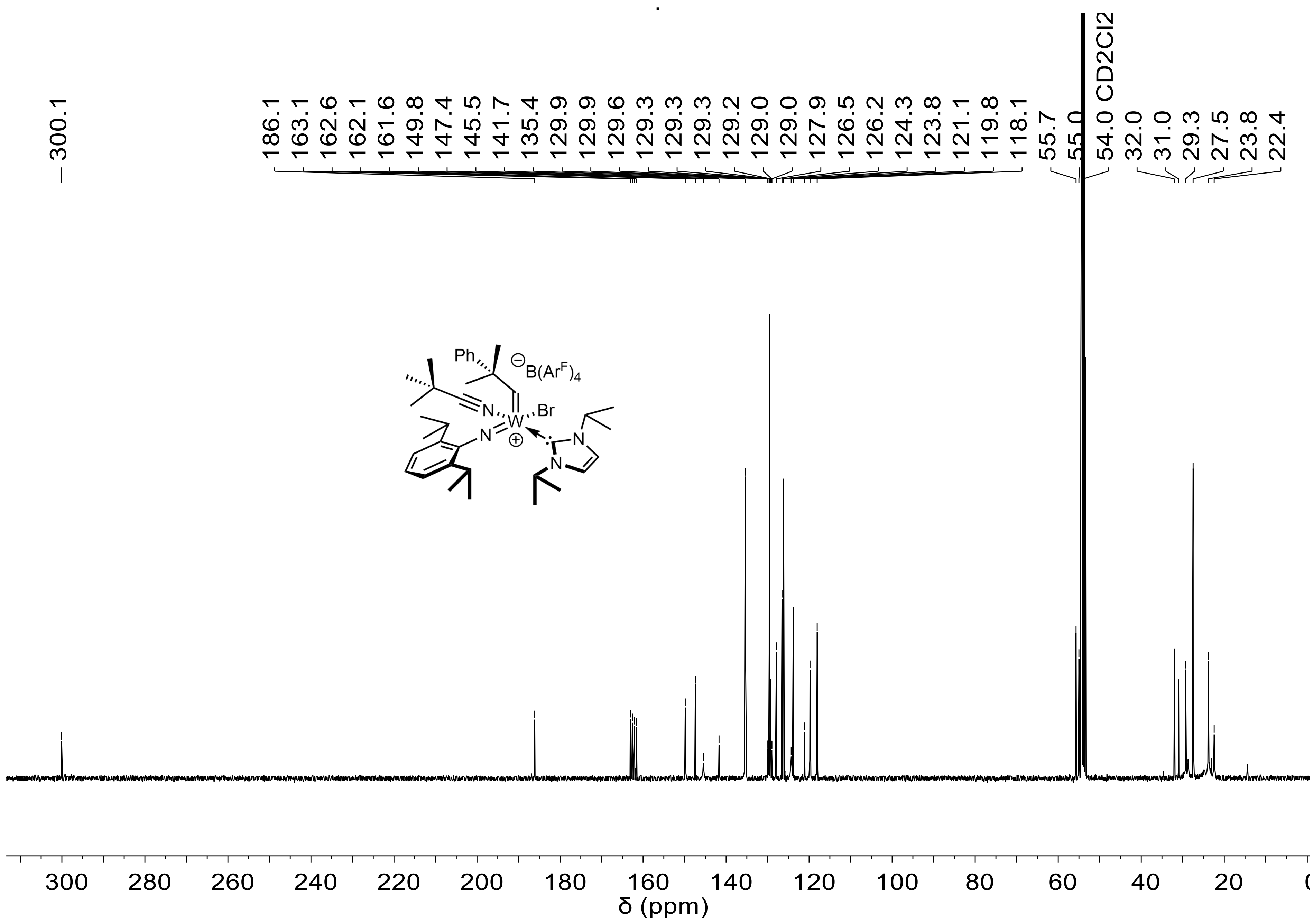

Figure S44: ^{13}C-NMR (101 MHz, 25 °C, CD_2Cl_2) of W-11.

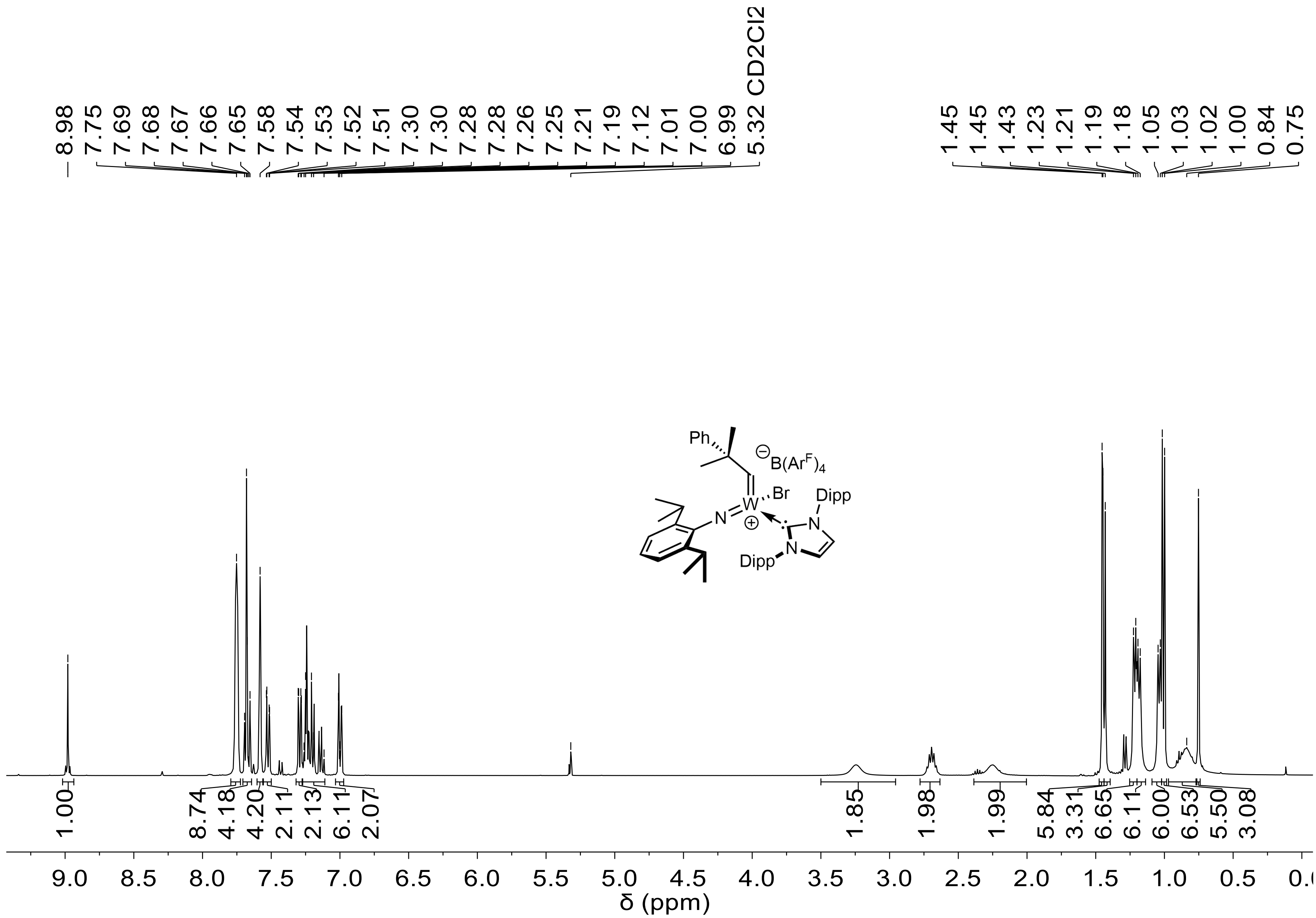

Figure S45: ^{1}H-NMR (400 MHz, 25 °C, CD_2Cl_2) of W-12.

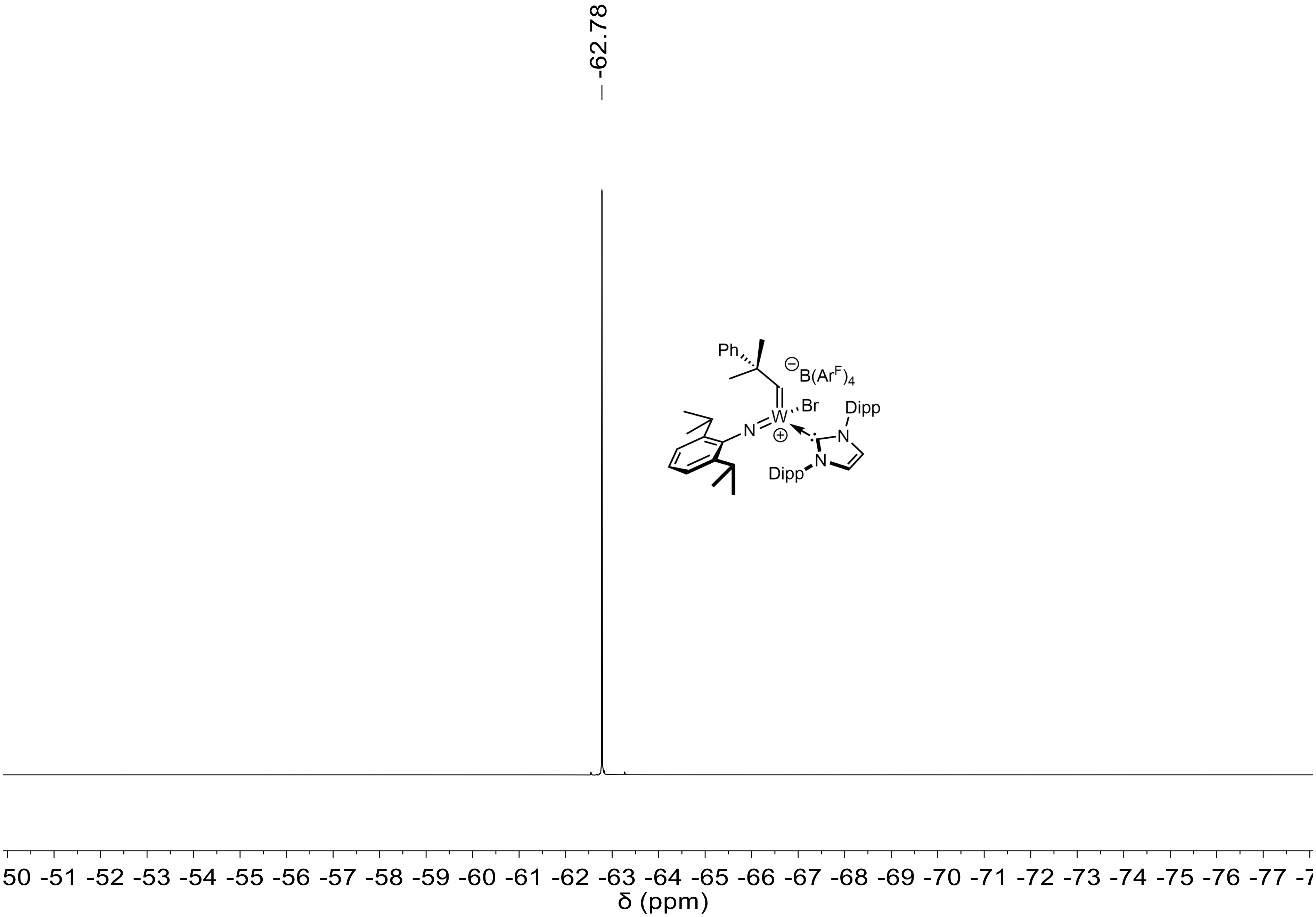

Figure S46: ^{19}F-NMR (376 MHz, 25 °C, CD_2Cl_2) of W-12.

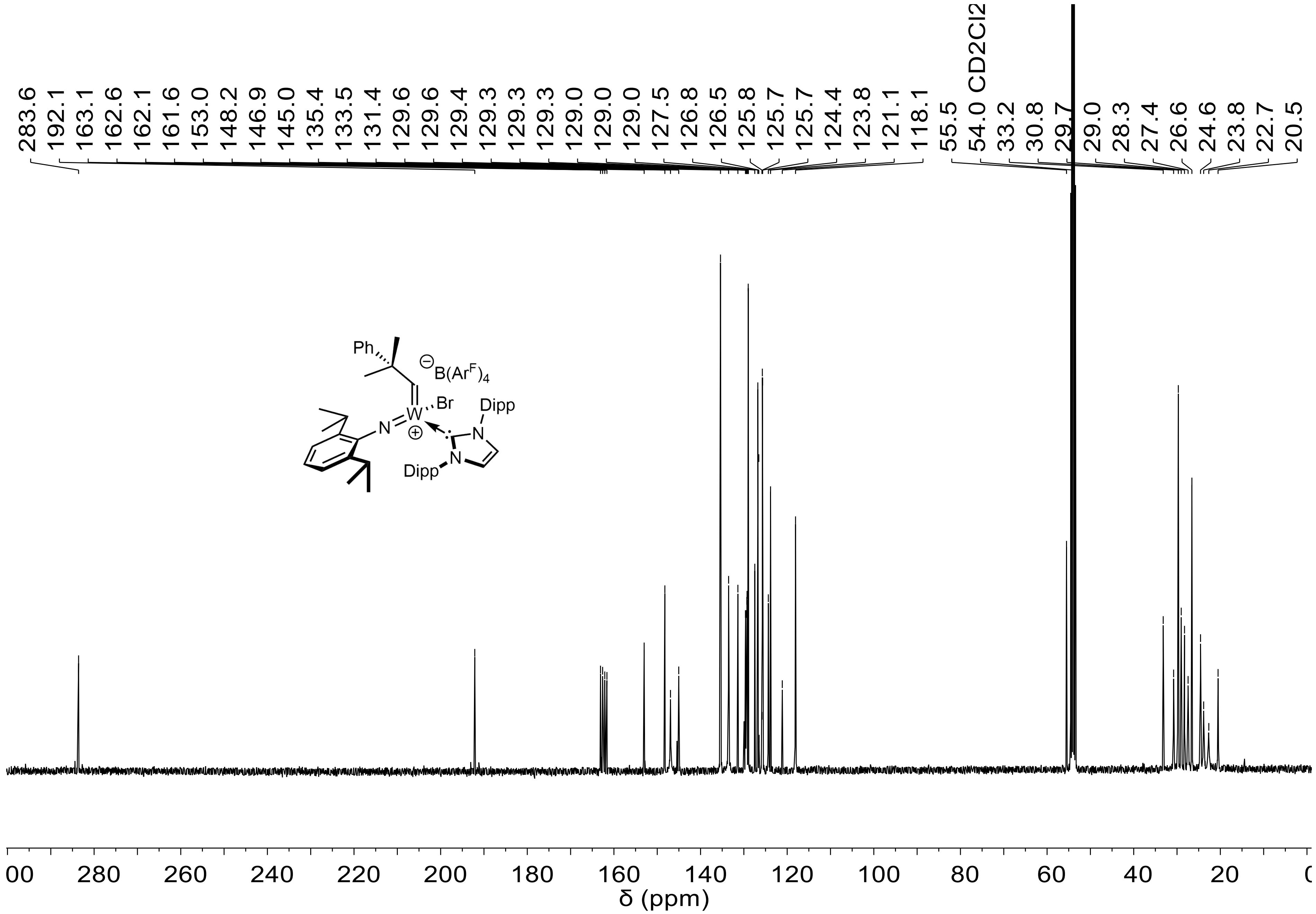

Figure S47: ^{13}C-NMR (101 MHz, 25 °C, CD_2Cl_2) of W-12.

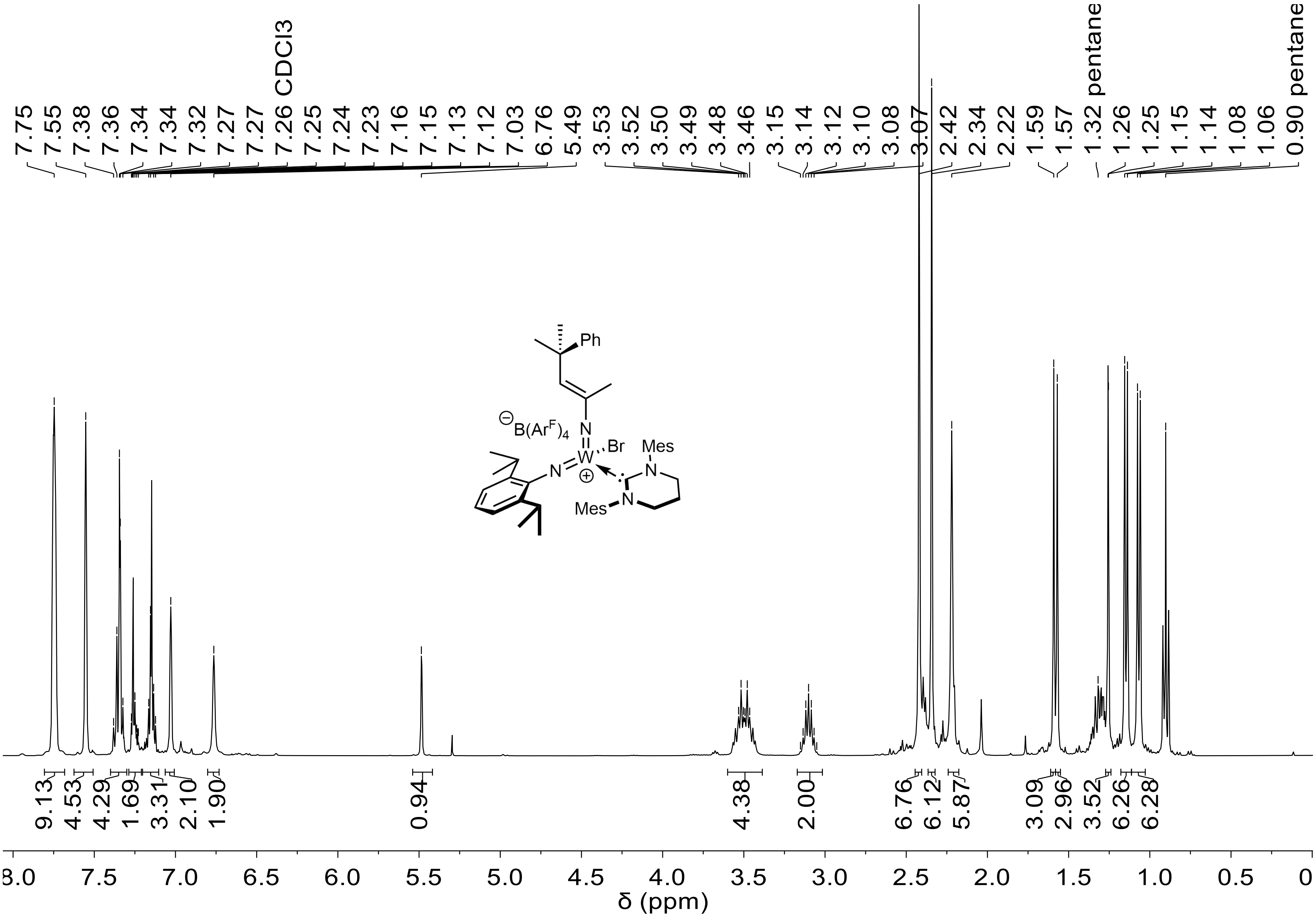

Figure S48: ^{1}H-NMR (400 MHz, 25 °C, $CDCl_3$) of W-13.

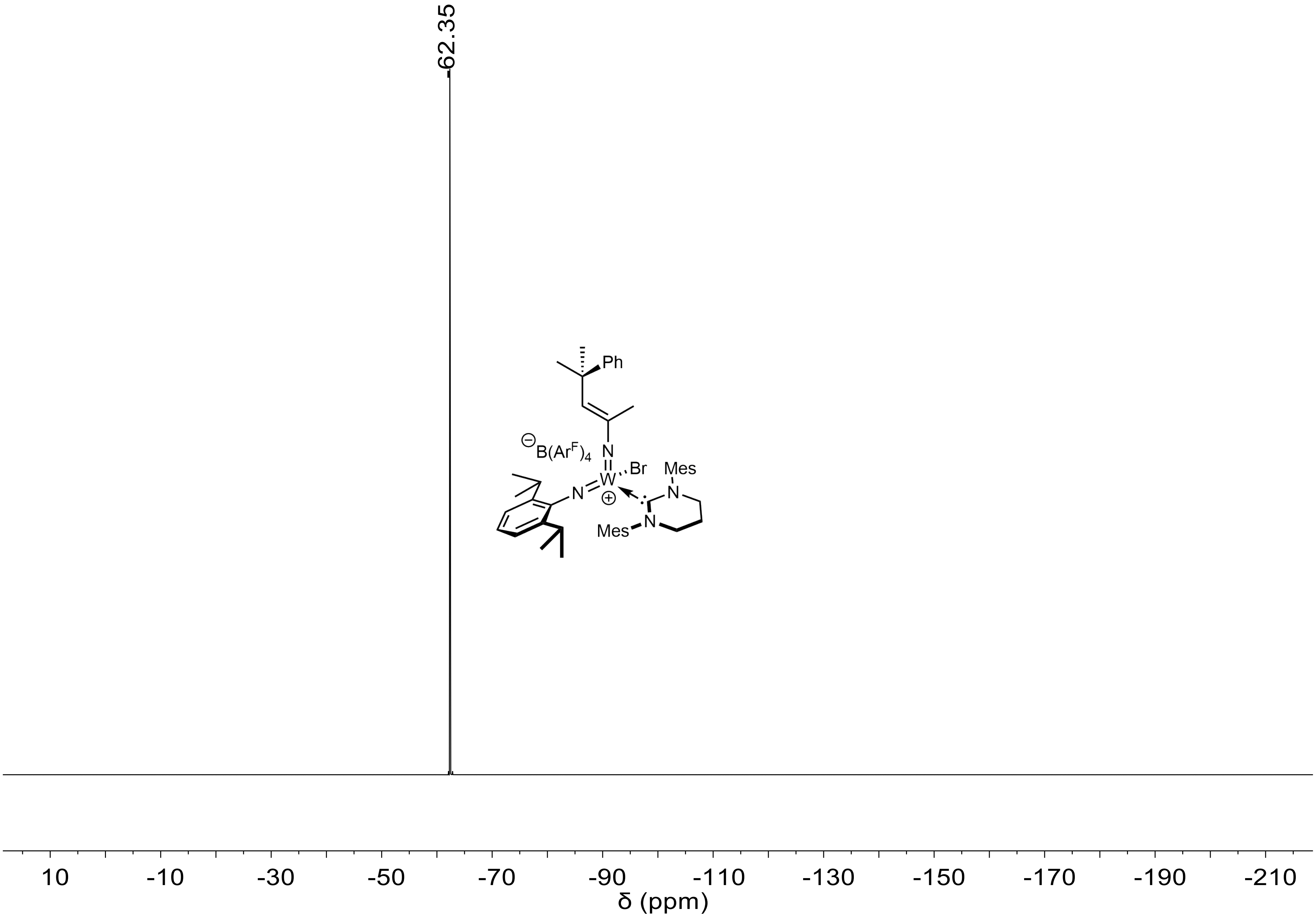

Figure S49: ^{19}F-NMR (376 MHz, 25 °C, CDCl$_3$) of W-13.

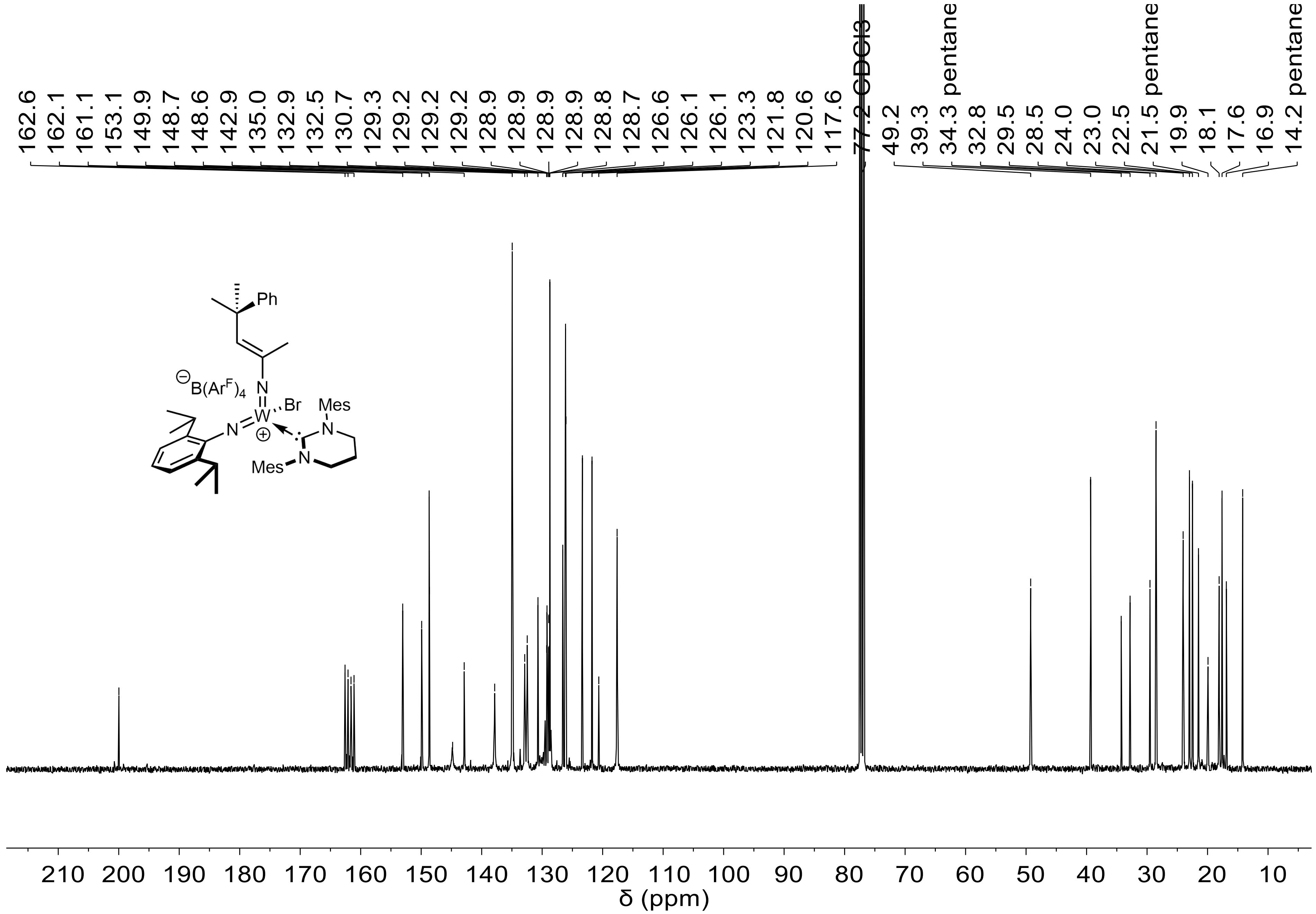

Figure S50: ^{13}C-NMR (101 MHz, 25 °C, $CDCl_3$) of W-13.

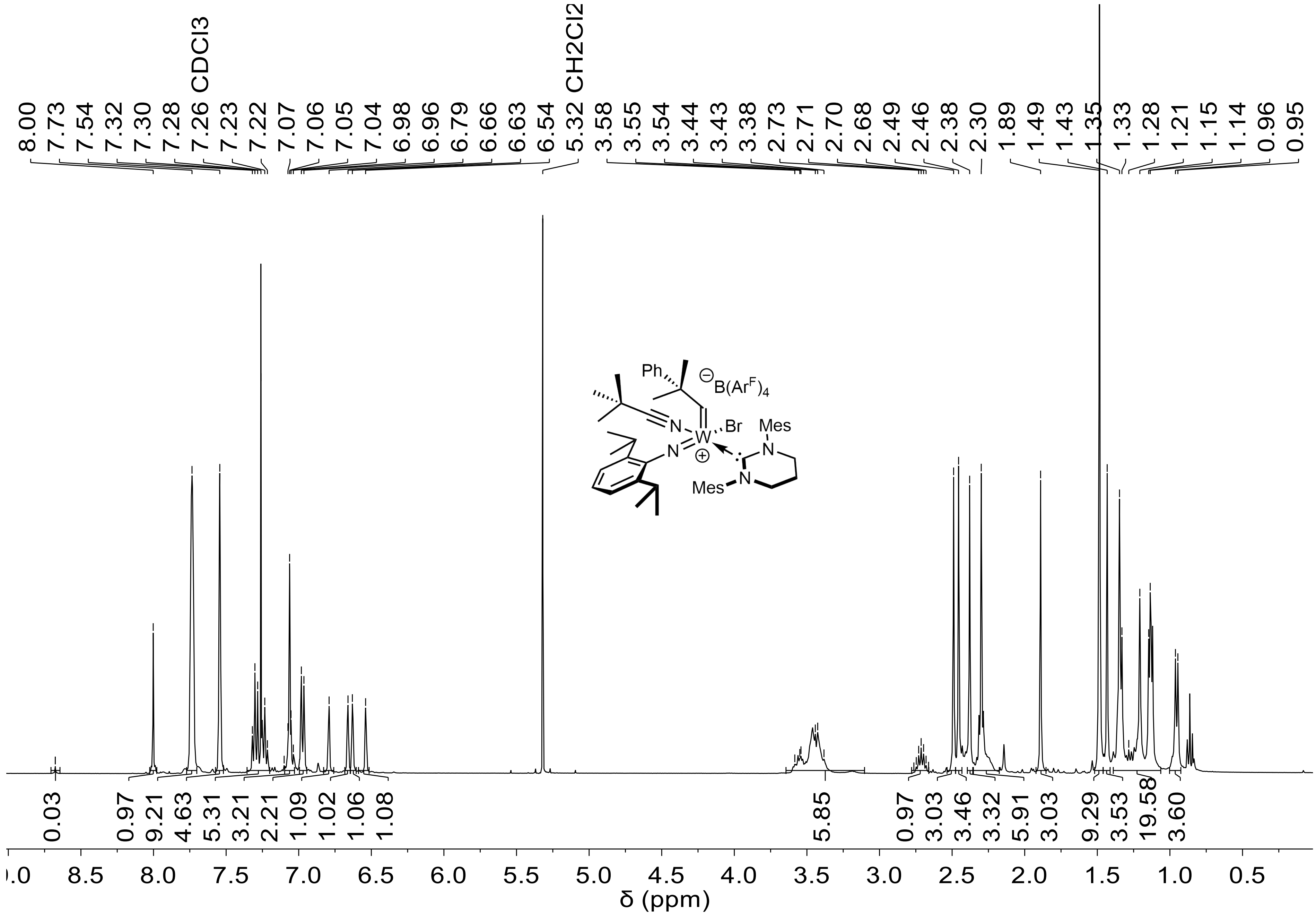

Figure S51: ¹H-NMR (400 MHz, -40 °C, $CDCl_3$) of W-14.

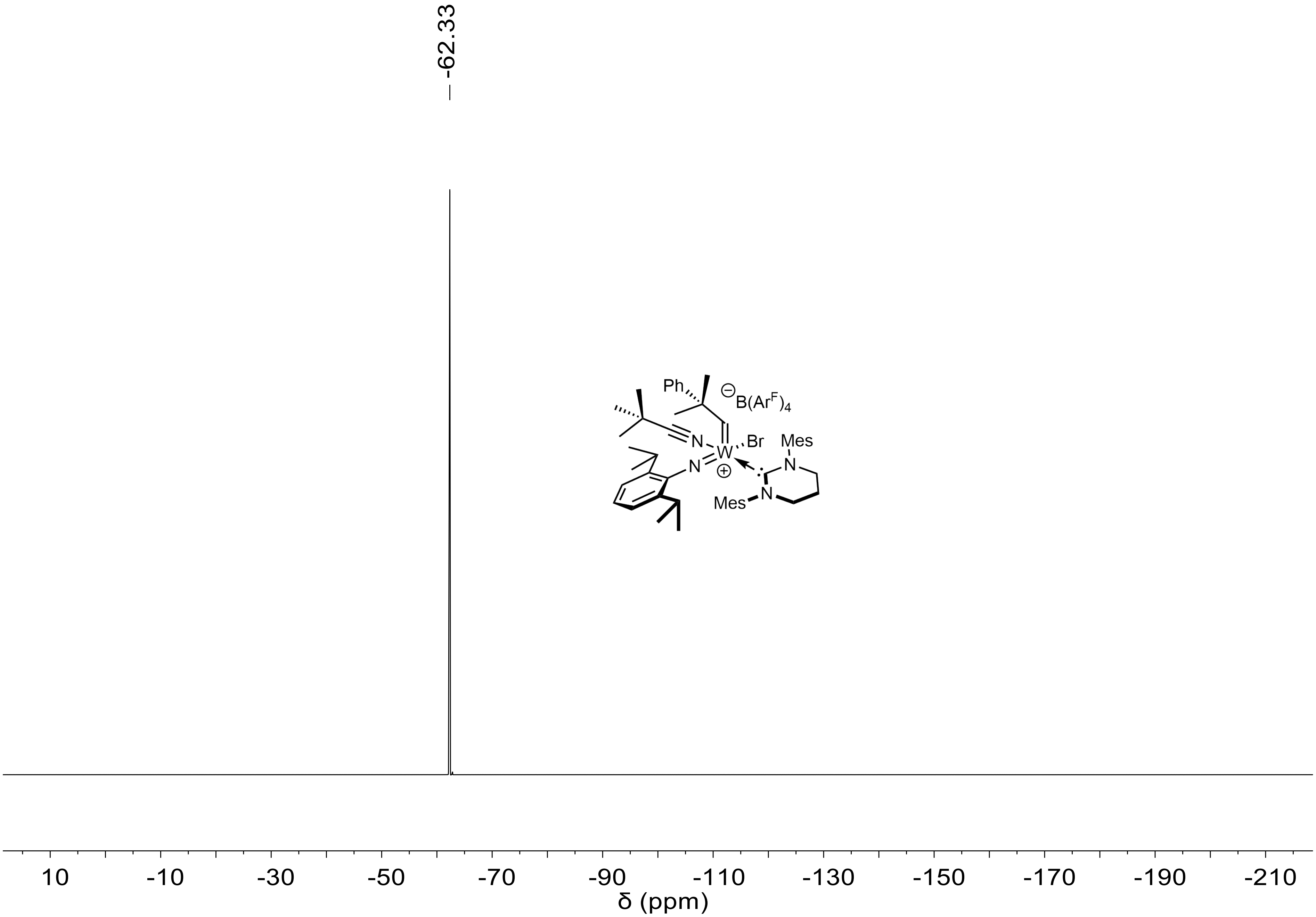

Figure S52: ^{19}F-NMR (376 MHz, 25 °C, $CDCl_3$) of W-14.

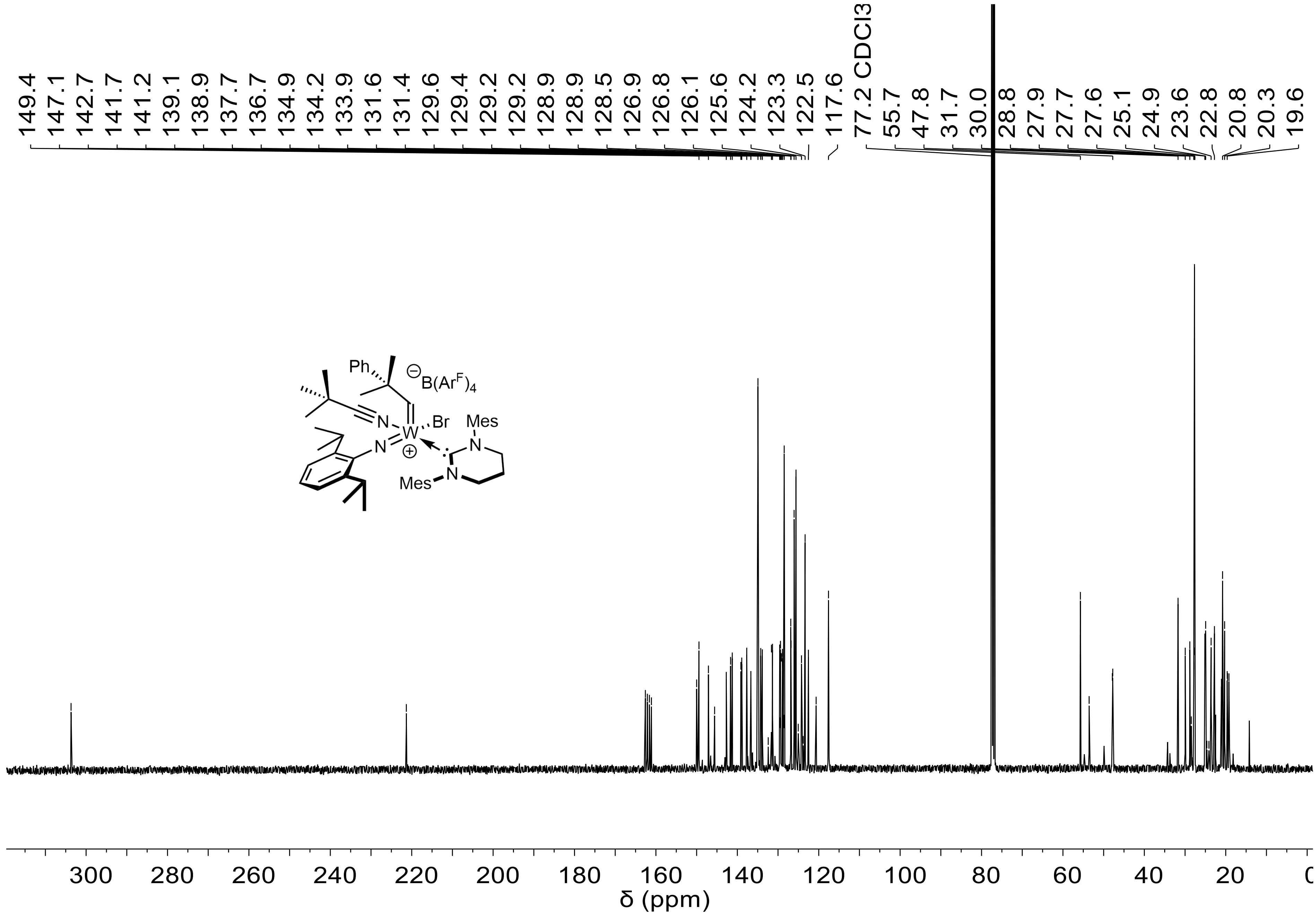

Figure S53: ^{13}C-NMR (101 MHz, 25 °C, CDCl$_3$) of W-14.

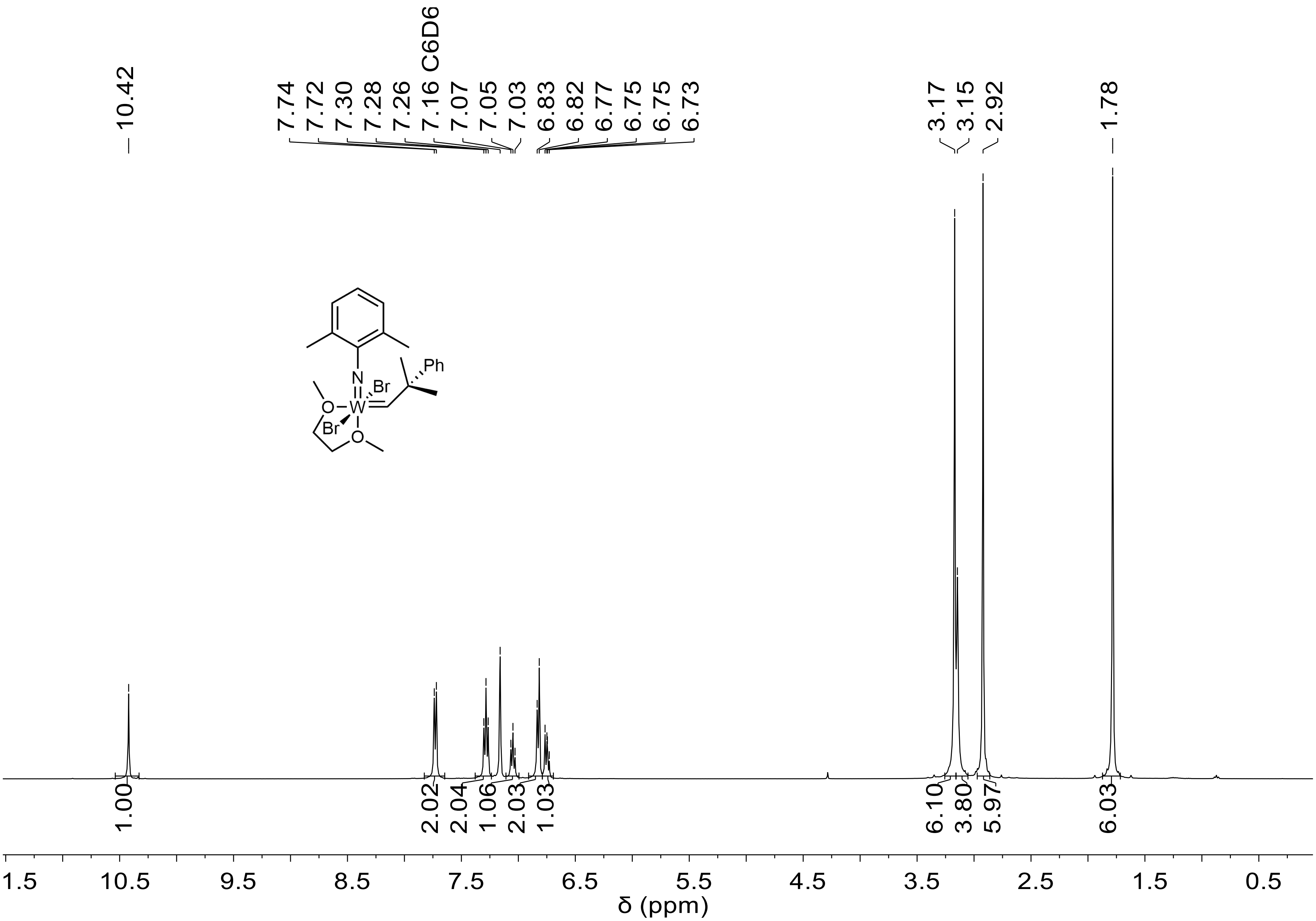

Figure S54: ^{1}H-NMR (400 MHz, 25 °C, C_6D_6) of W-15.

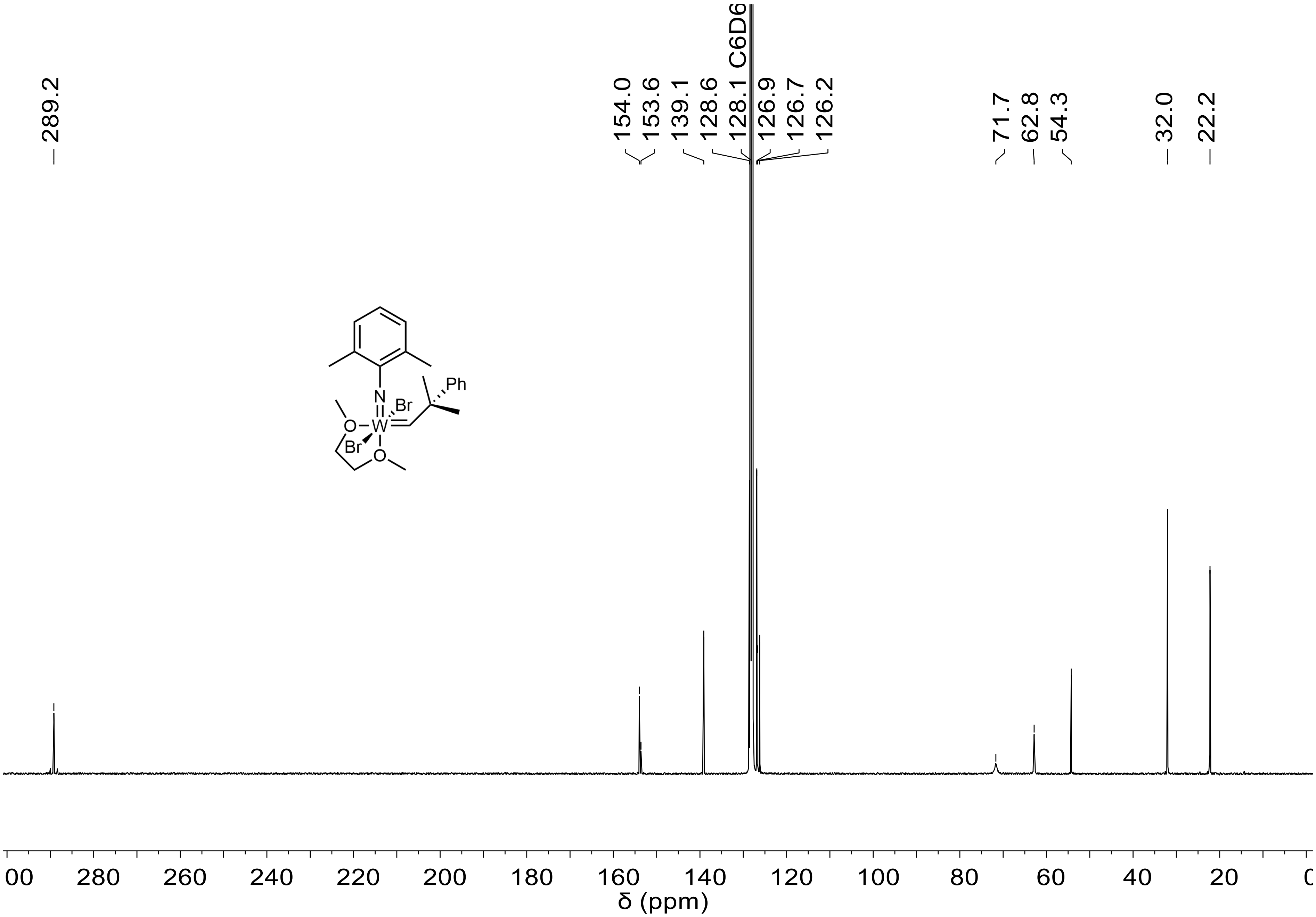

Figure S55: ^{13}C-NMR (101 MHz, 25 °C, C_6D_6) of W-15.

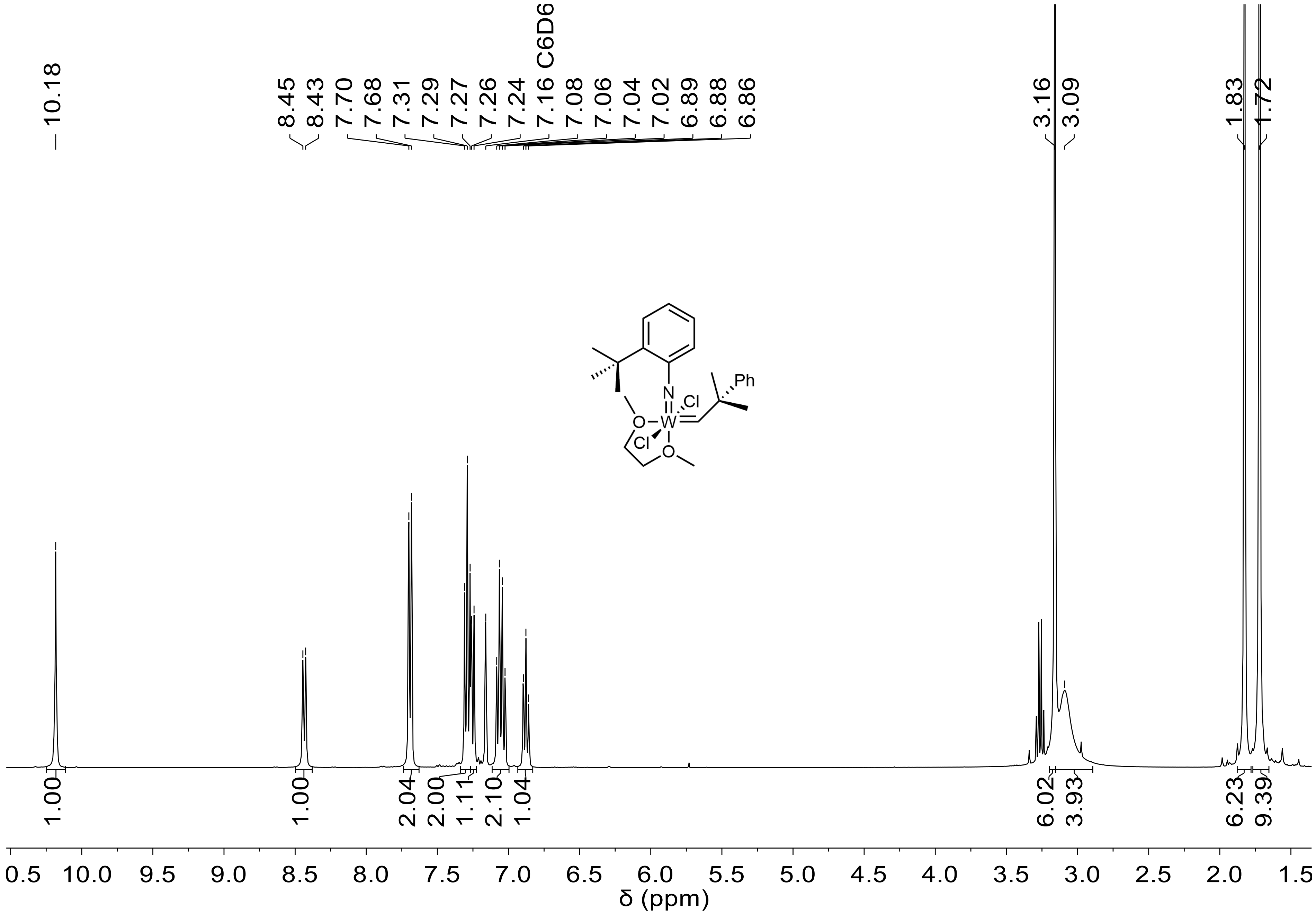

Figure S56: ^{1}H-NMR (400 MHz, 25 °C, C_6D_6) of W-19.

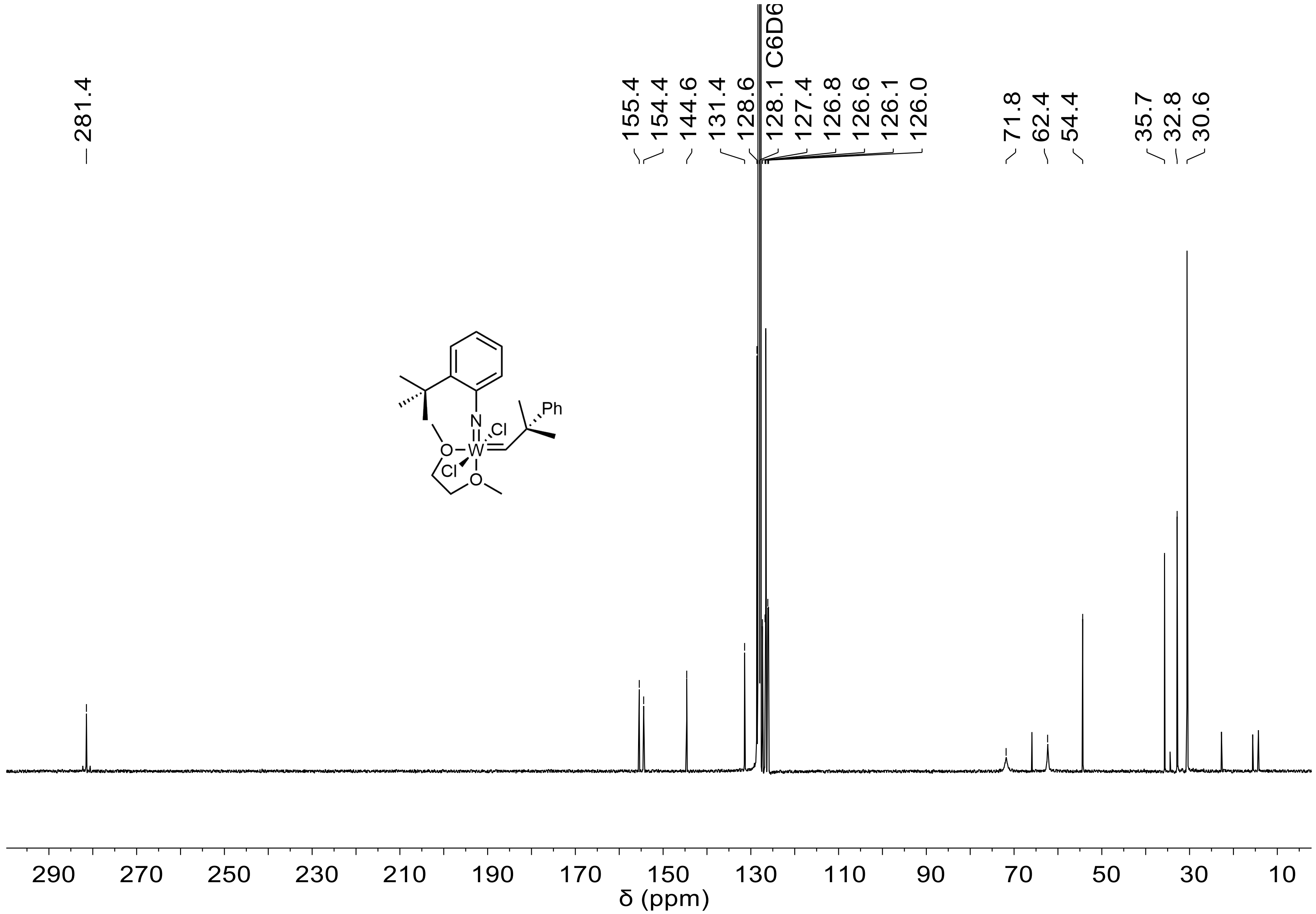

Figure S57: ^{13}C-NMR (101 MHz, 25 °C, C_6D_6) of W-19.

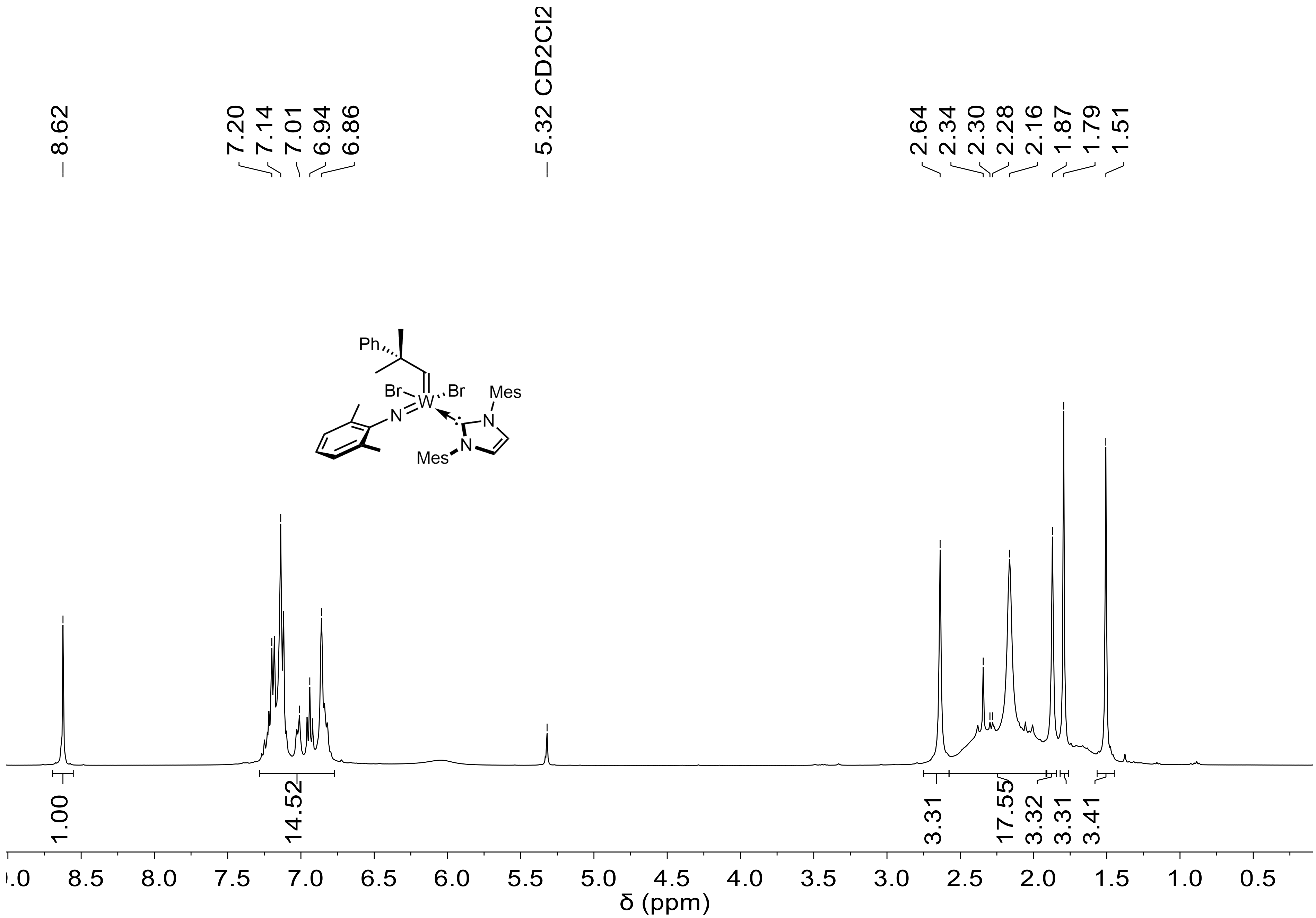

Figure S58: ^{1}H-NMR (400 MHz, 25 °C, CD_2Cl_2) of W-20.

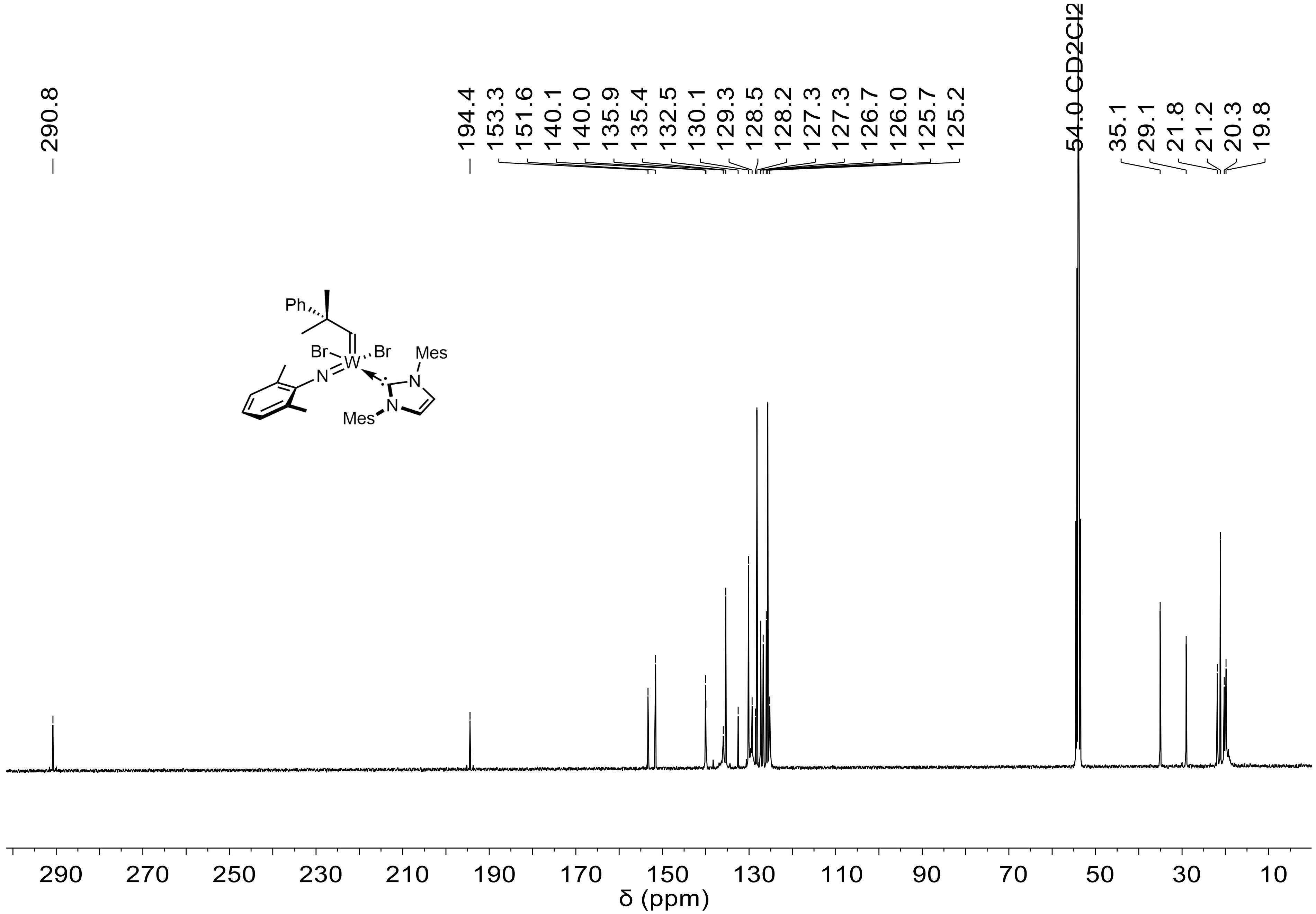

Figure S59: ^{13}C-NMR (101 MHz, 25 °C, CD_2Cl_2) of W-20.

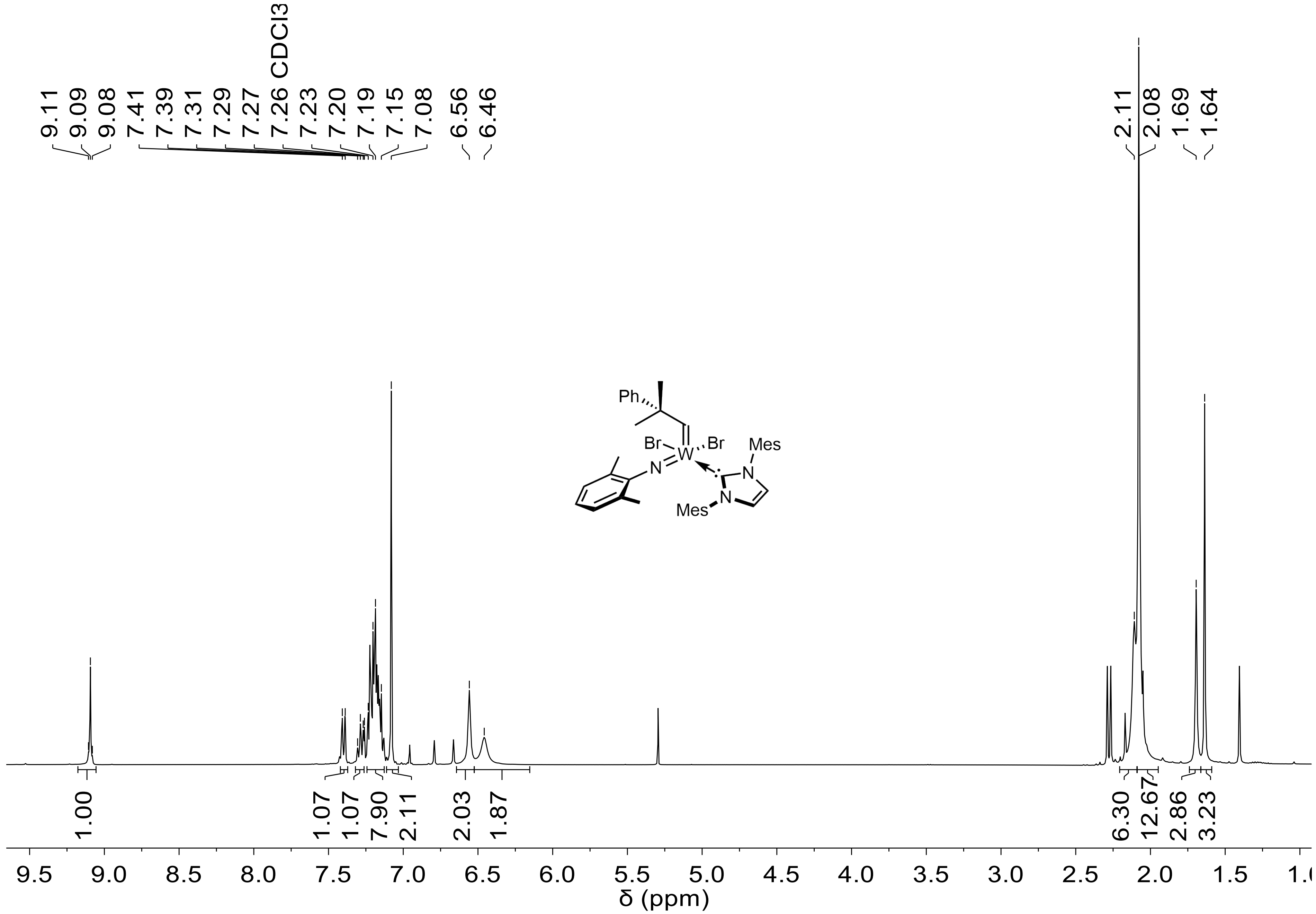

Figure S60: ^{1}H-NMR (400 MHz, 25 °C, $CDCl_3$) of W-21.

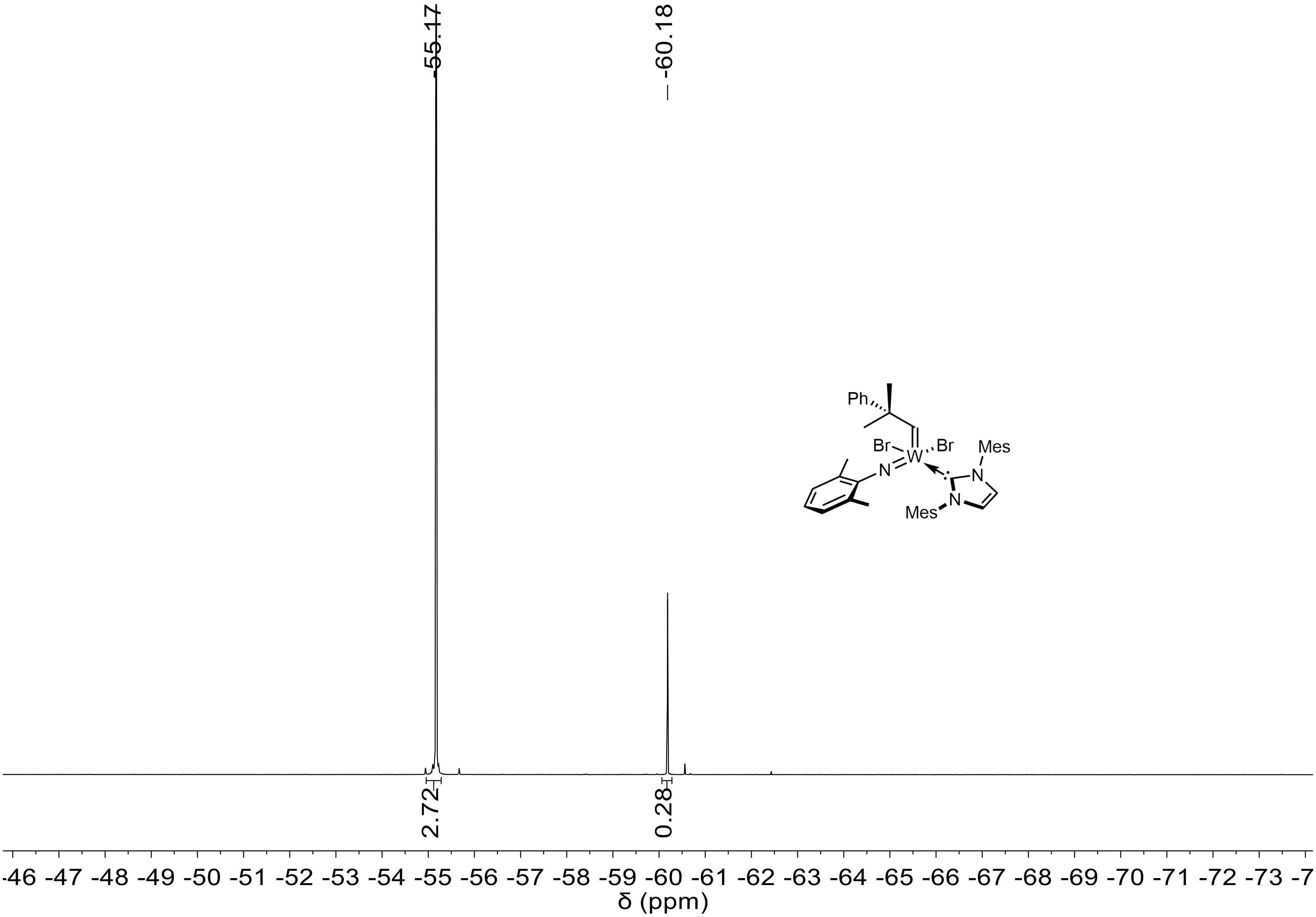

Figure S61: ^{19}F-NMR (376 MHz, 25 °C, $CDCl_3$) of W-21.

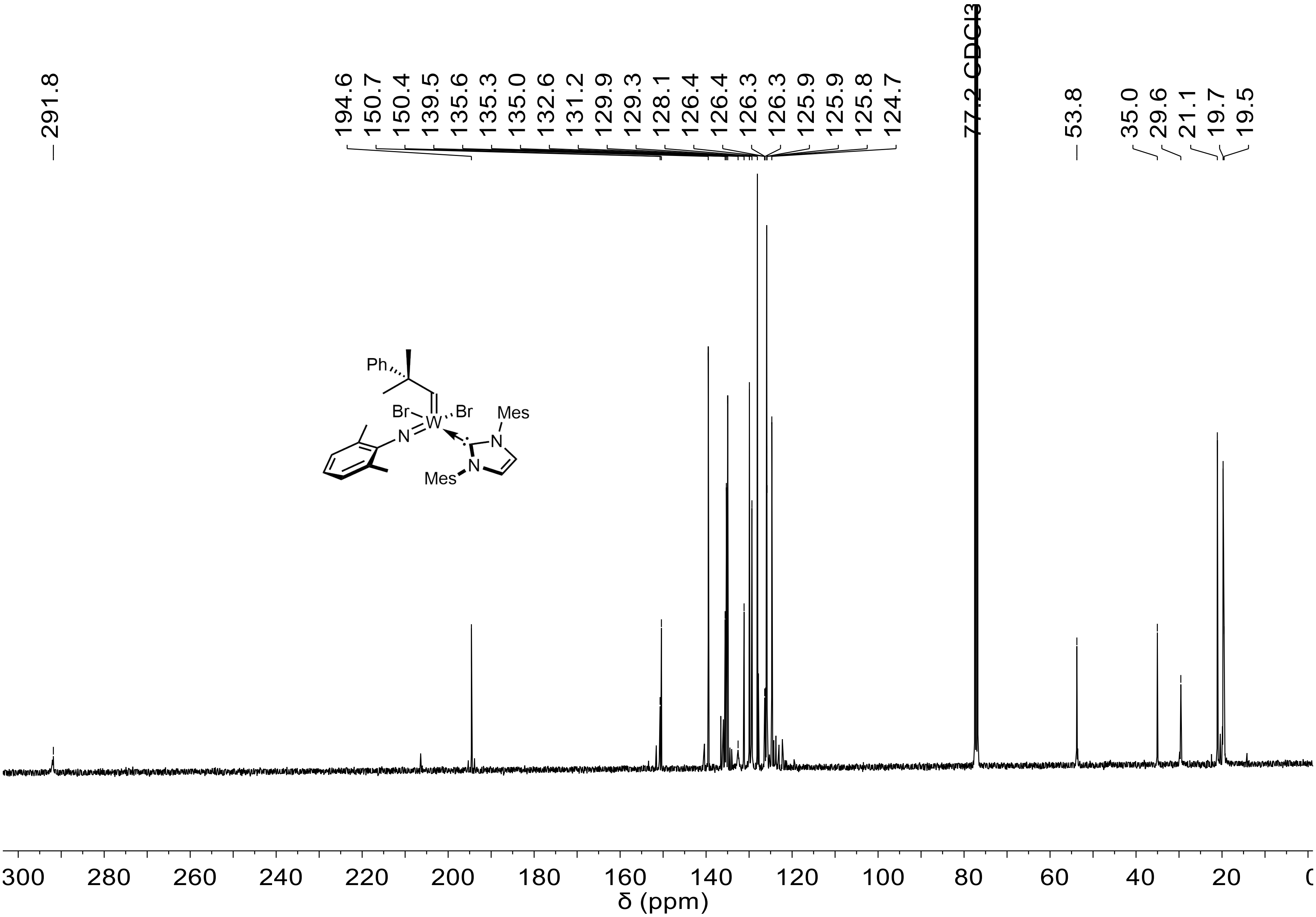

Figure S62: ^{13}C-NMR (101 MHz, 25 °C, CDCl$_3$) of W-21.

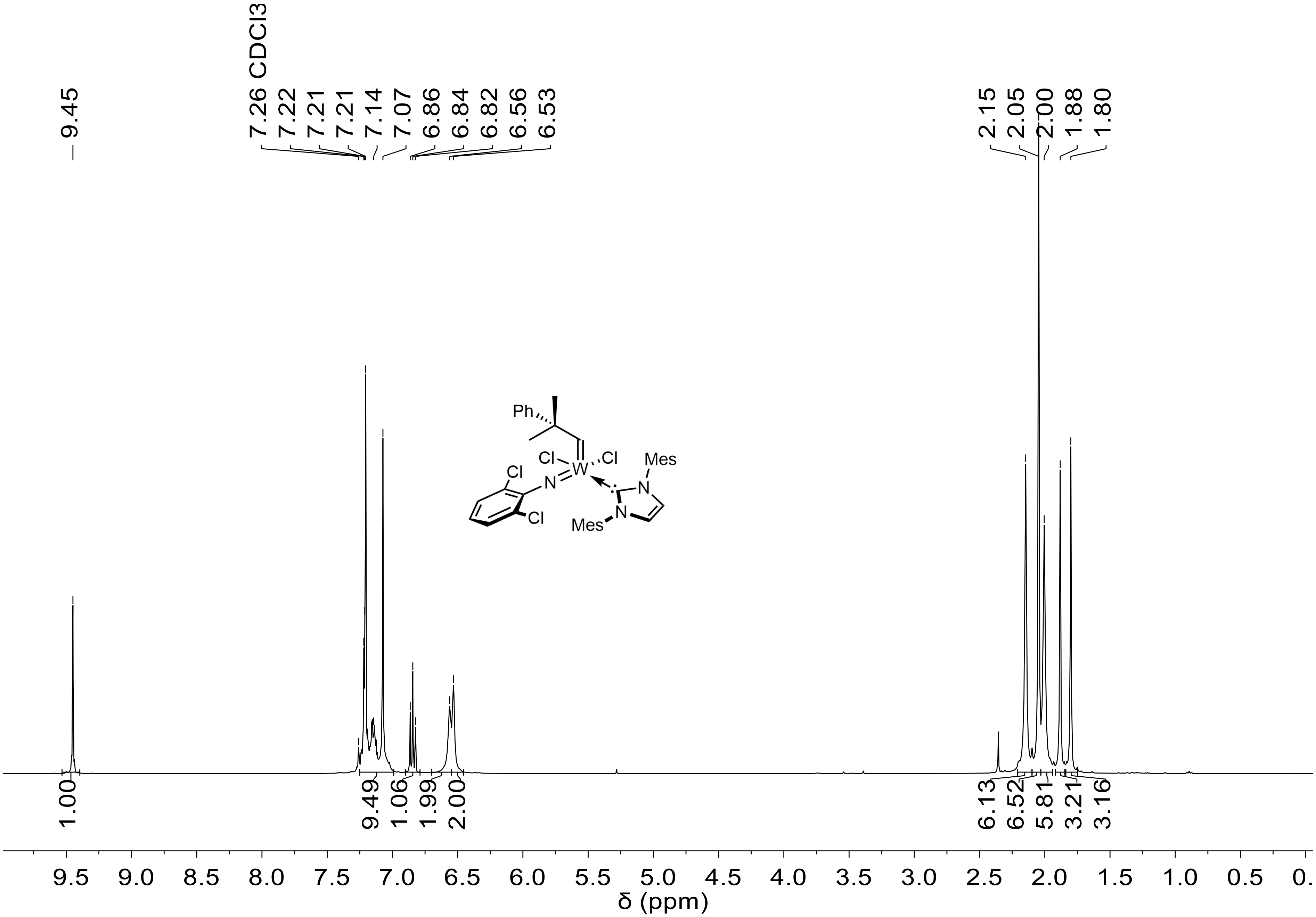

Figure S63: ^{1}H-NMR (400 MHz, 25 °C, $CDCl_3$) of W-22.

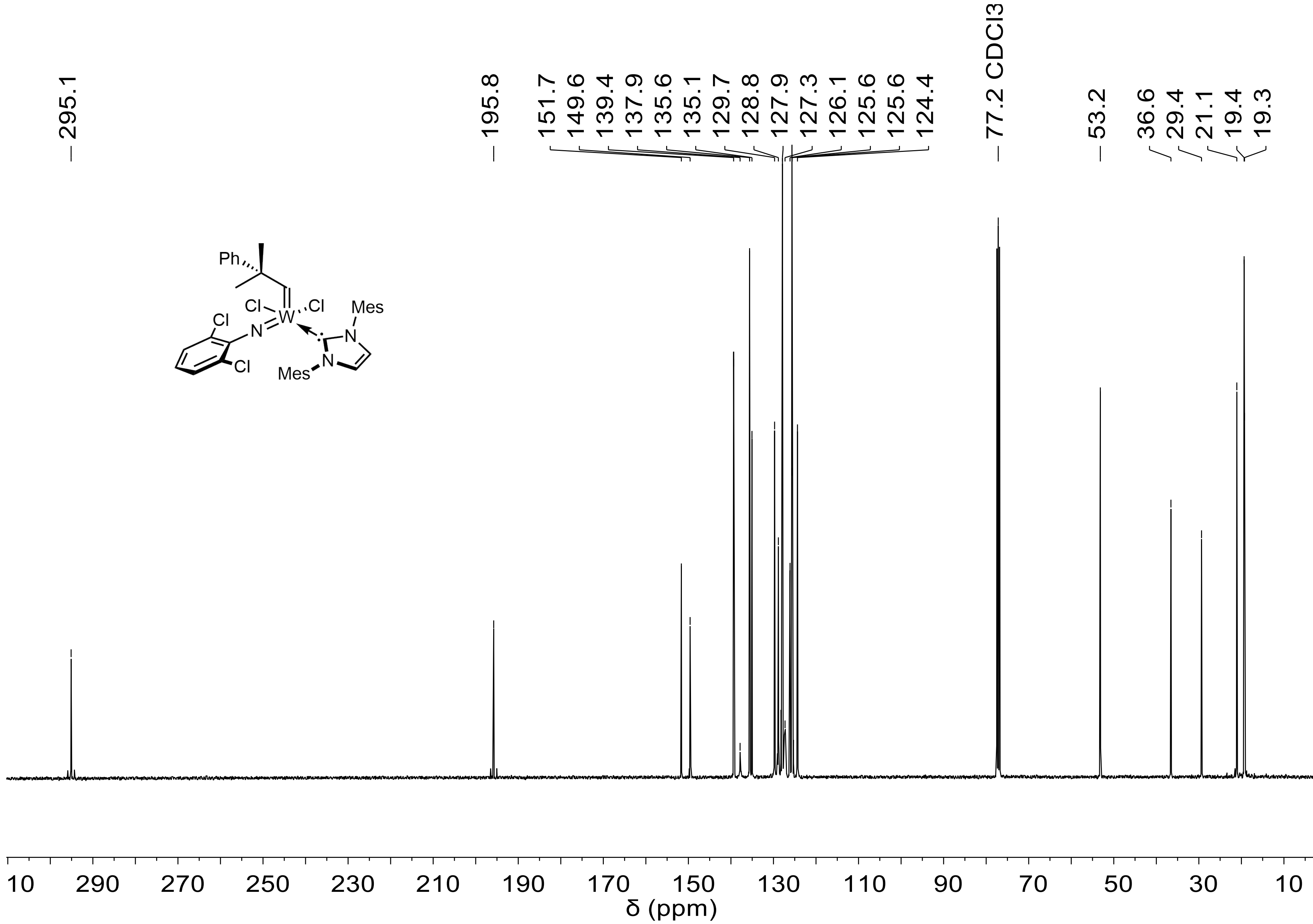

Figure S64: ^{13}C-NMR (101 MHz, 25 °C, $CDCl_3$) of W-22.

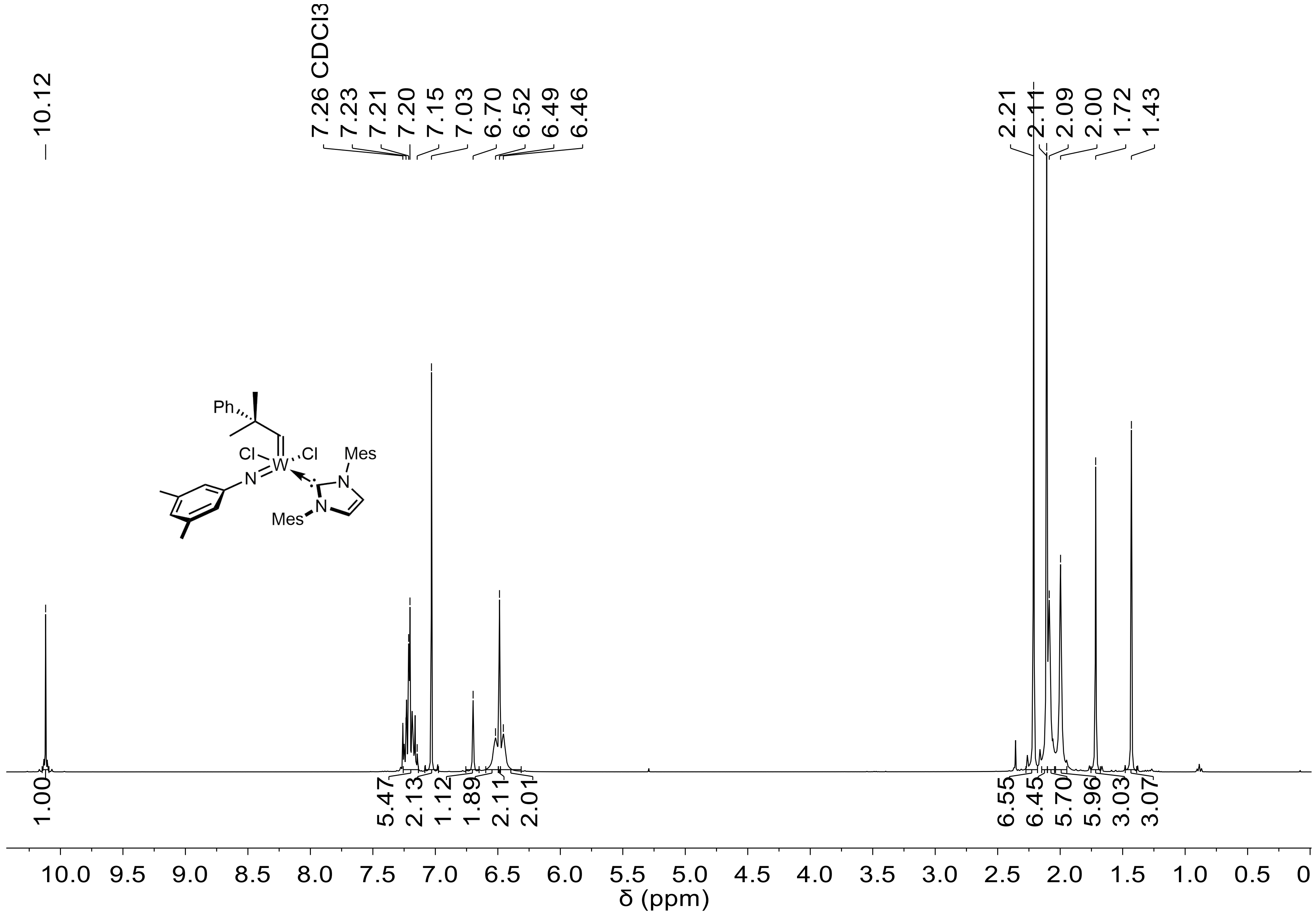

Figure S65: ^{1}H-NMR (400 MHz, 25 °C, $CDCl_3$) of W-23.

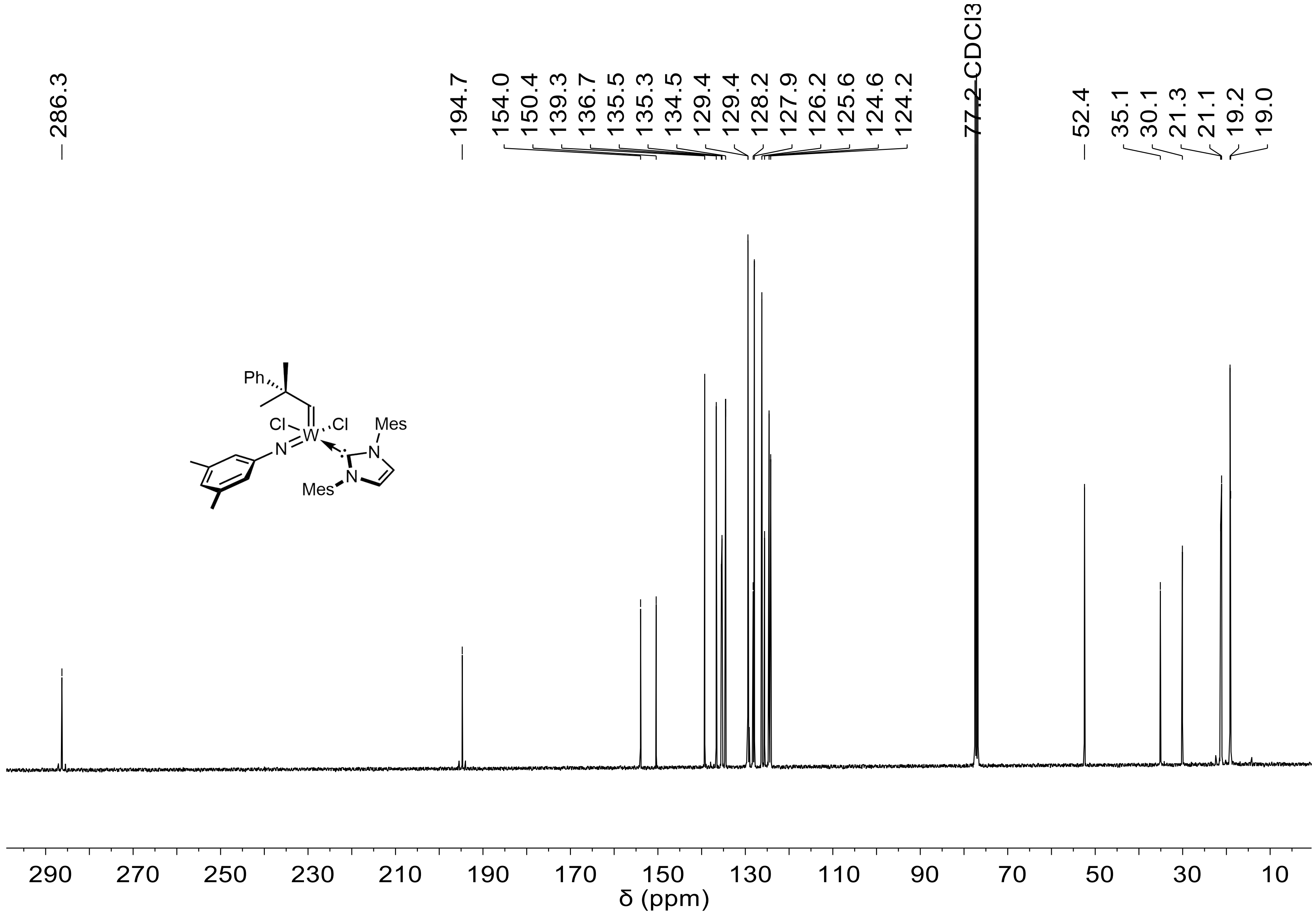

Figure S66: ^{13}C-NMR (101 MHz, 25 °C, $CDCl_3$) of W-23.

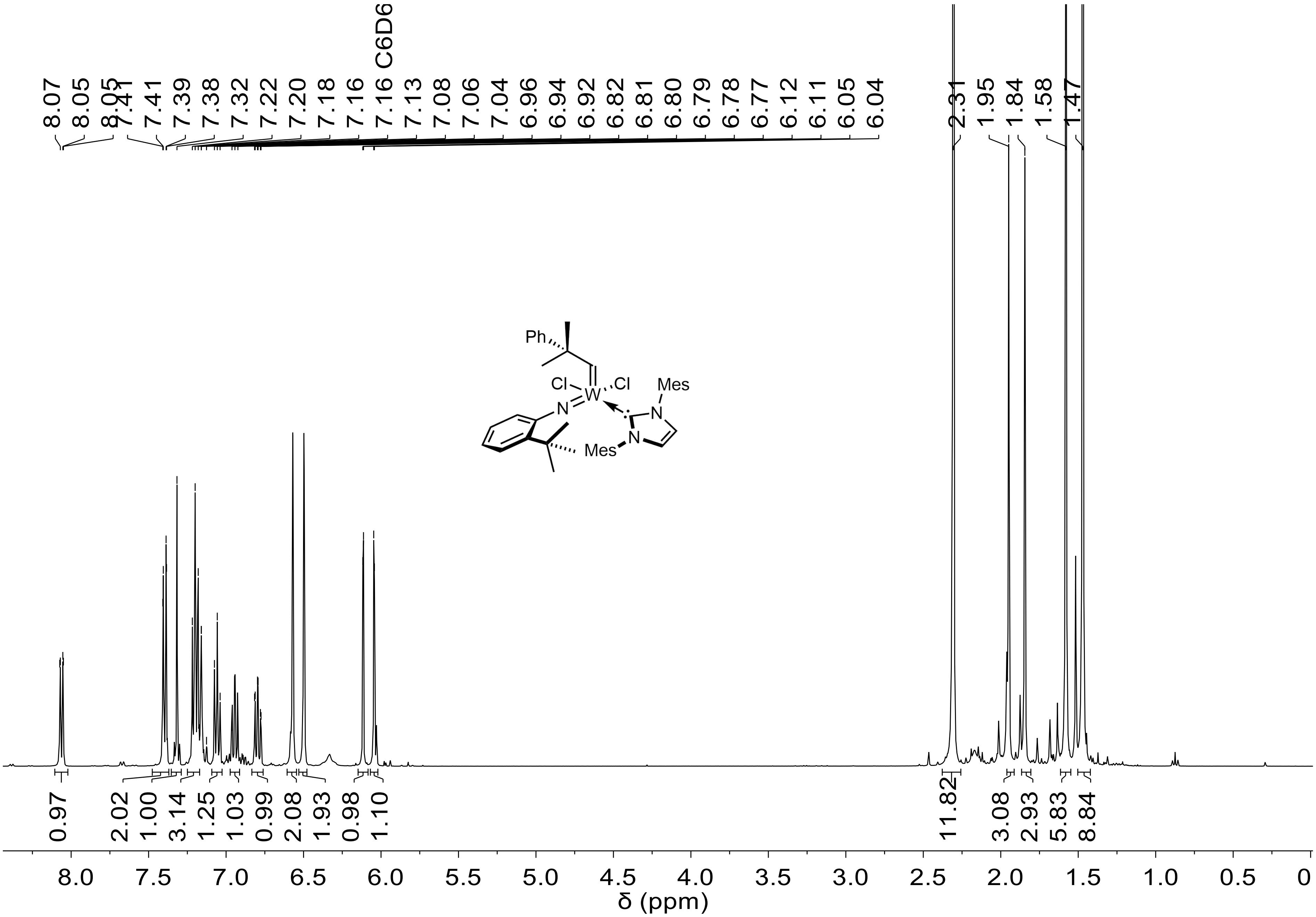

Figure S67: ^{1}H-NMR (400 MHz, 25 °C, C_6D_6) of W-24.

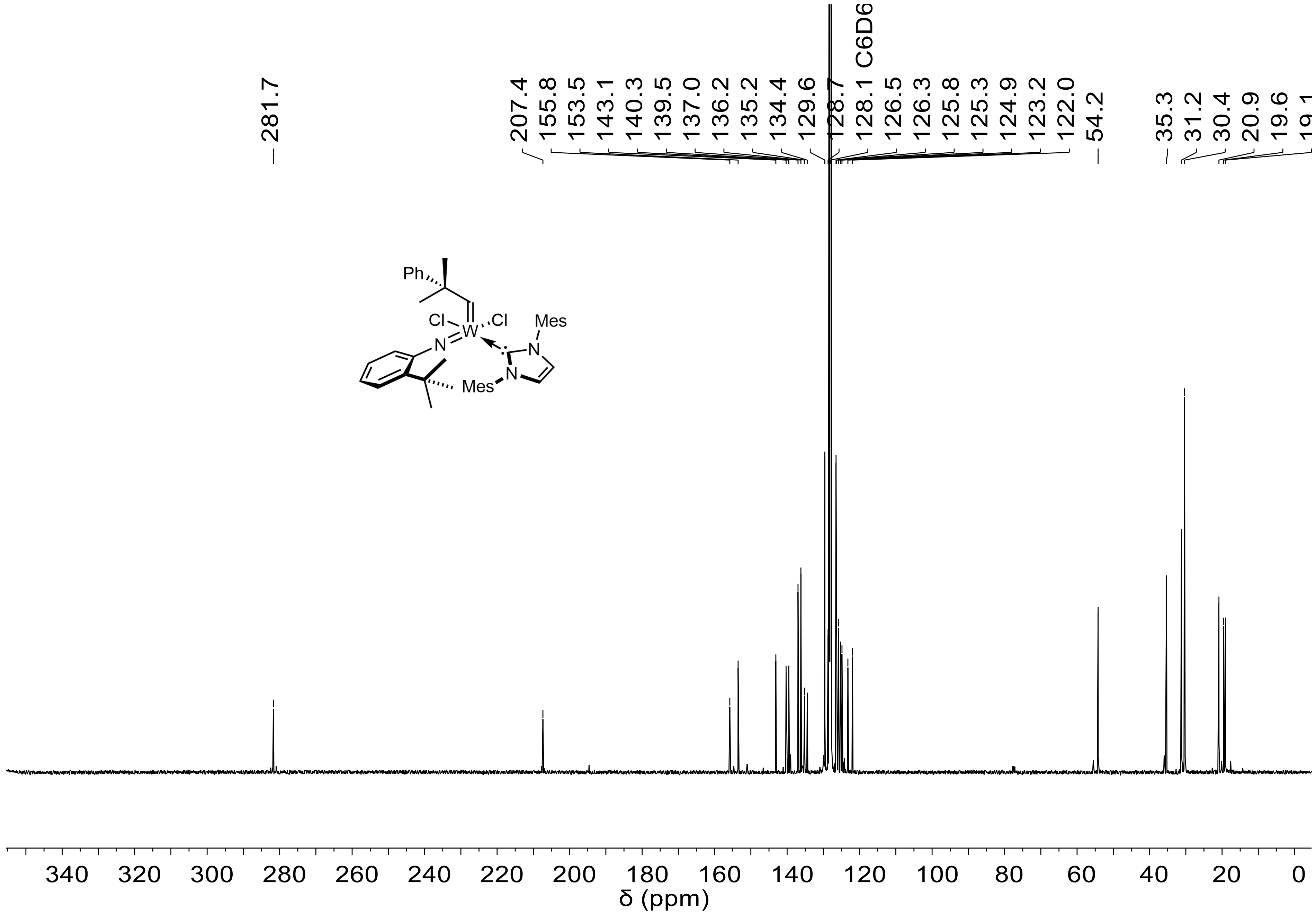

Figure S68: ^{13}C-NMR (101 MHz, 25 °C, C_6D_6) of W-24.

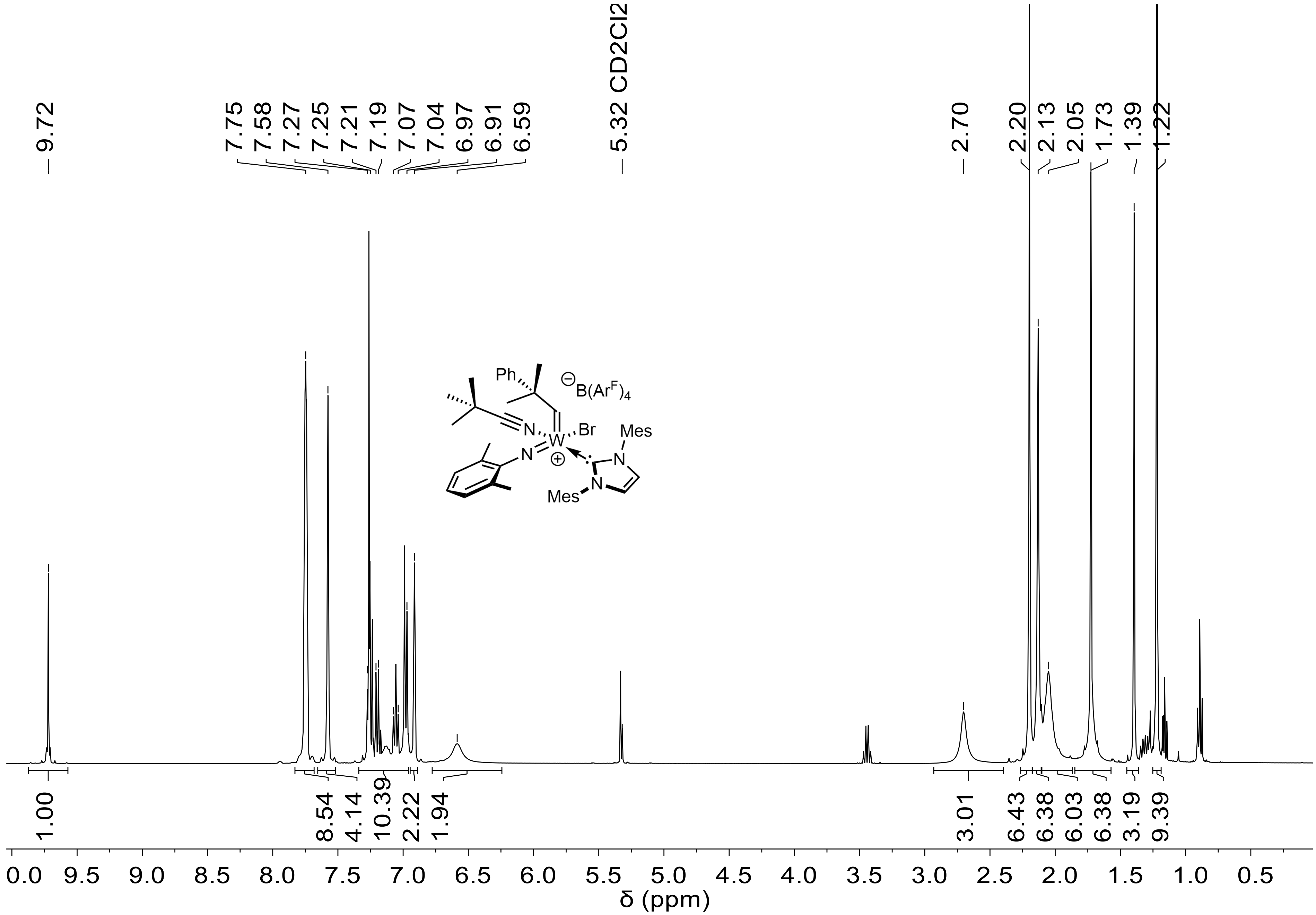

Figure S69: ^{1}H-NMR (400 MHz, 25 °C, CD_2Cl_2) of W-25.

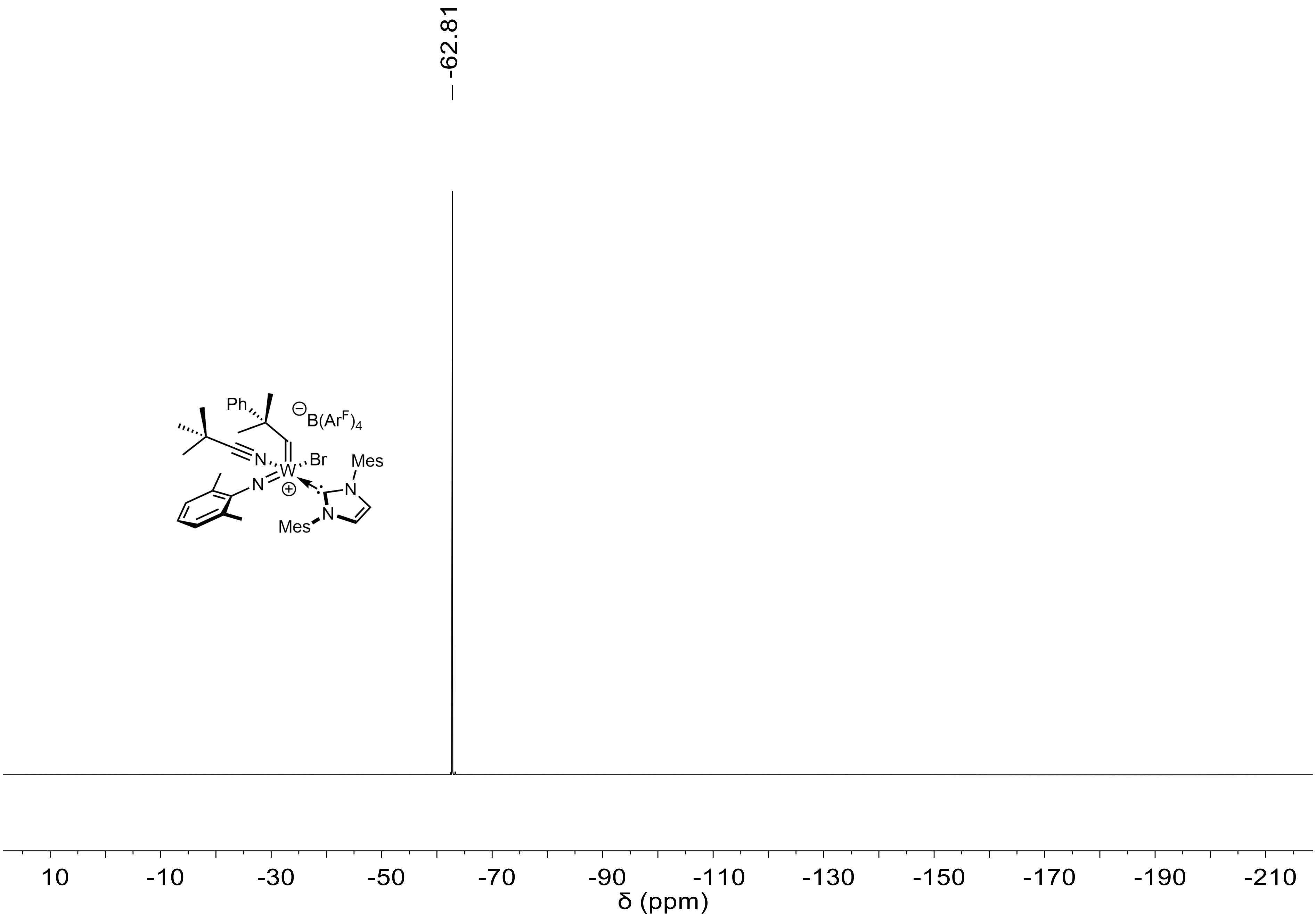

Figure S70: ^{19}F-NMR (376 MHz, 25 °C, CD_2Cl_2) of W-25.

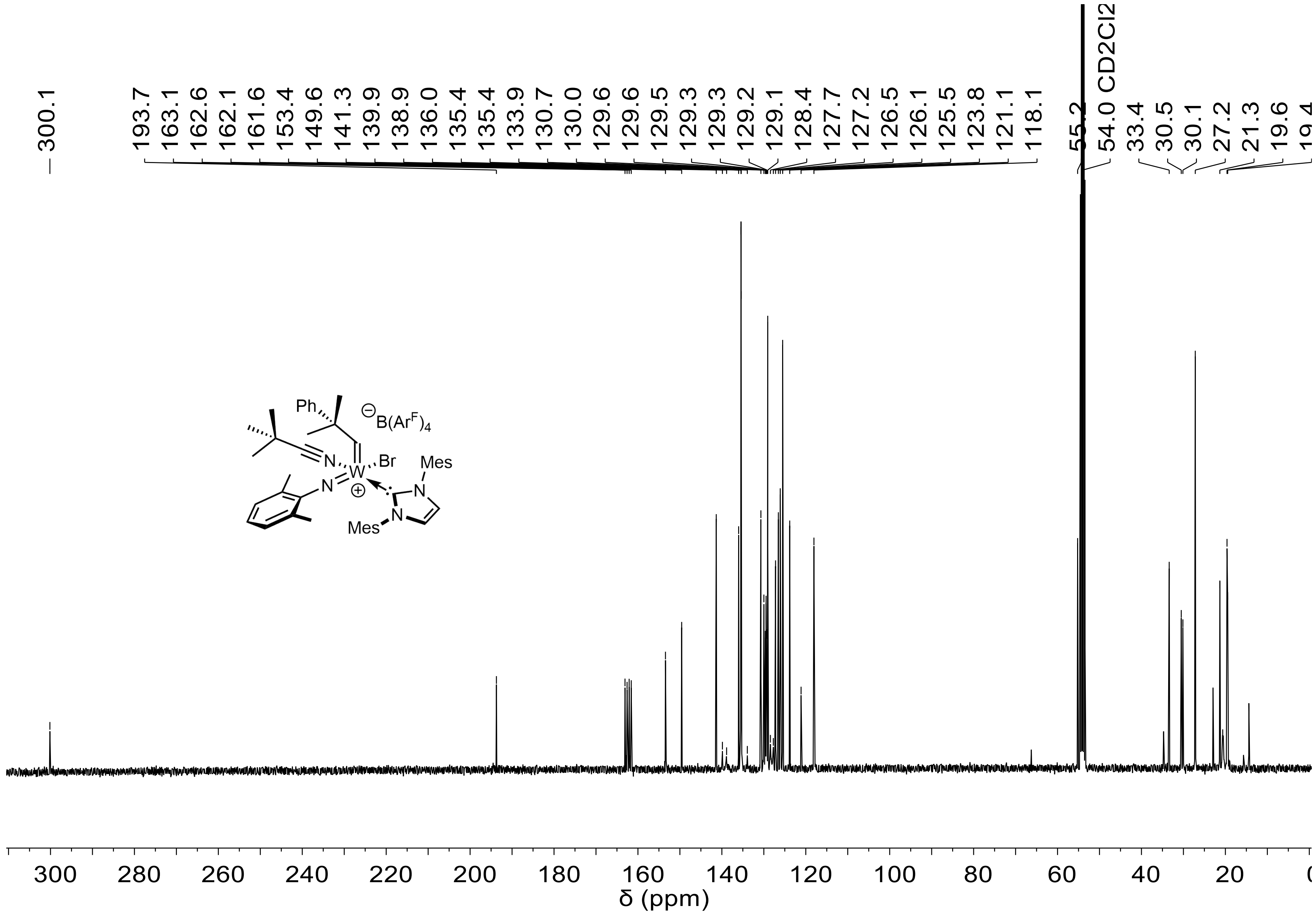

Figure S71: ^{13}C-NMR (101 MHz, 25 °C, CD_2Cl_2) of W-25.

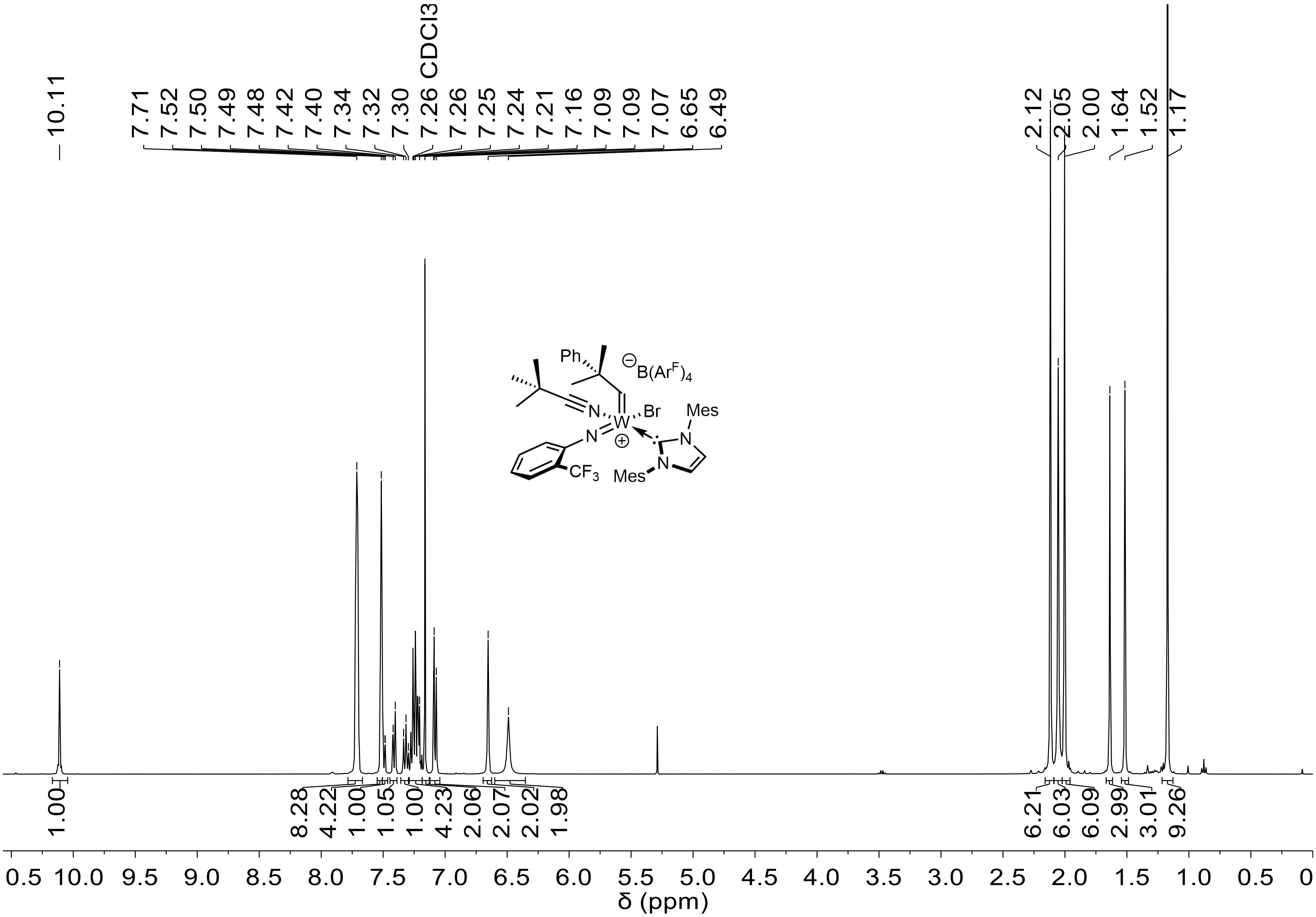

Figure S72: ^{1}H-NMR (400 MHz, 25 °C, $CDCl_3$) of W-26.

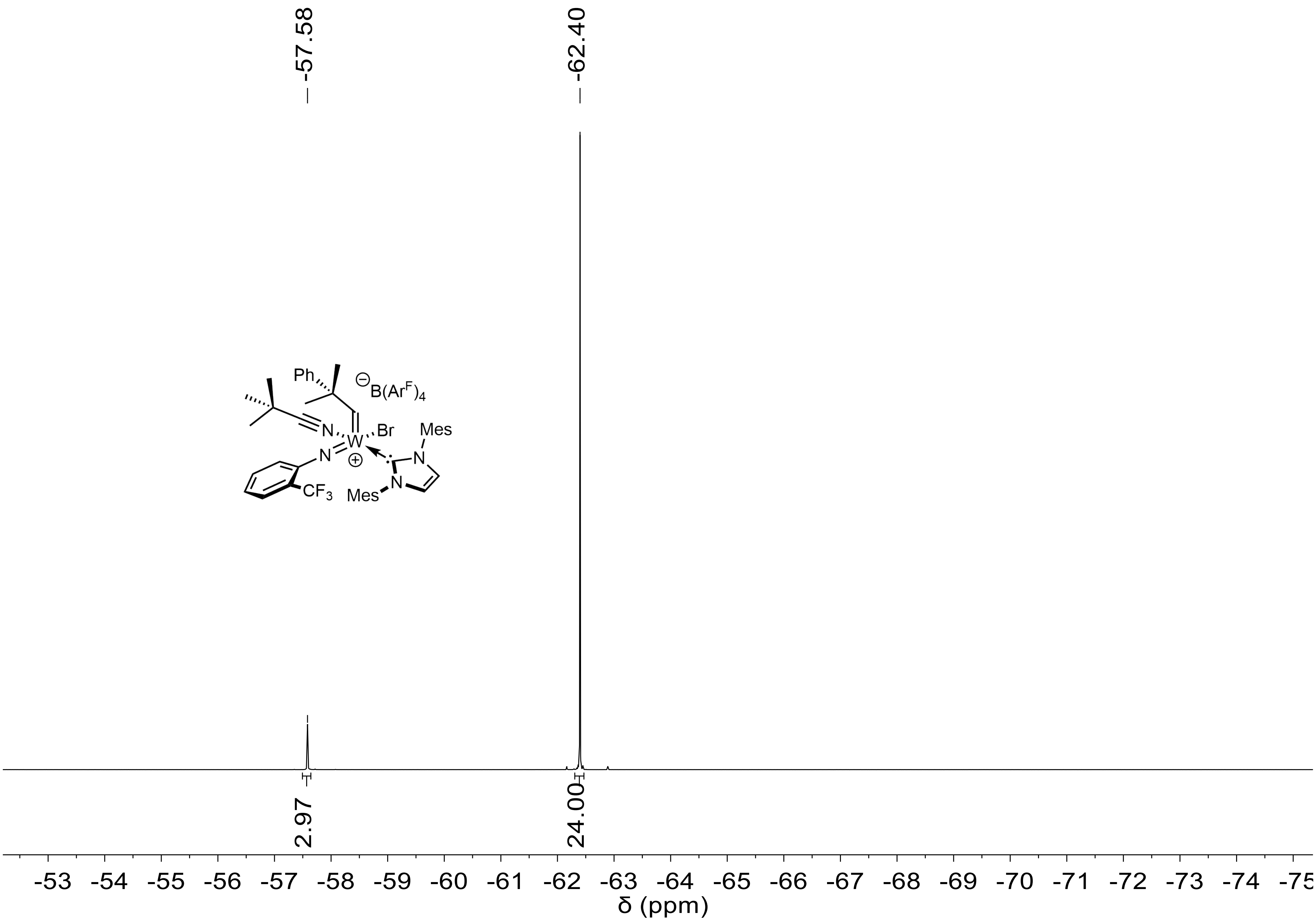

Figure S73: ^{19}F-NMR (376 MHz, 25 °C, $CDCl_3$) of W-26.

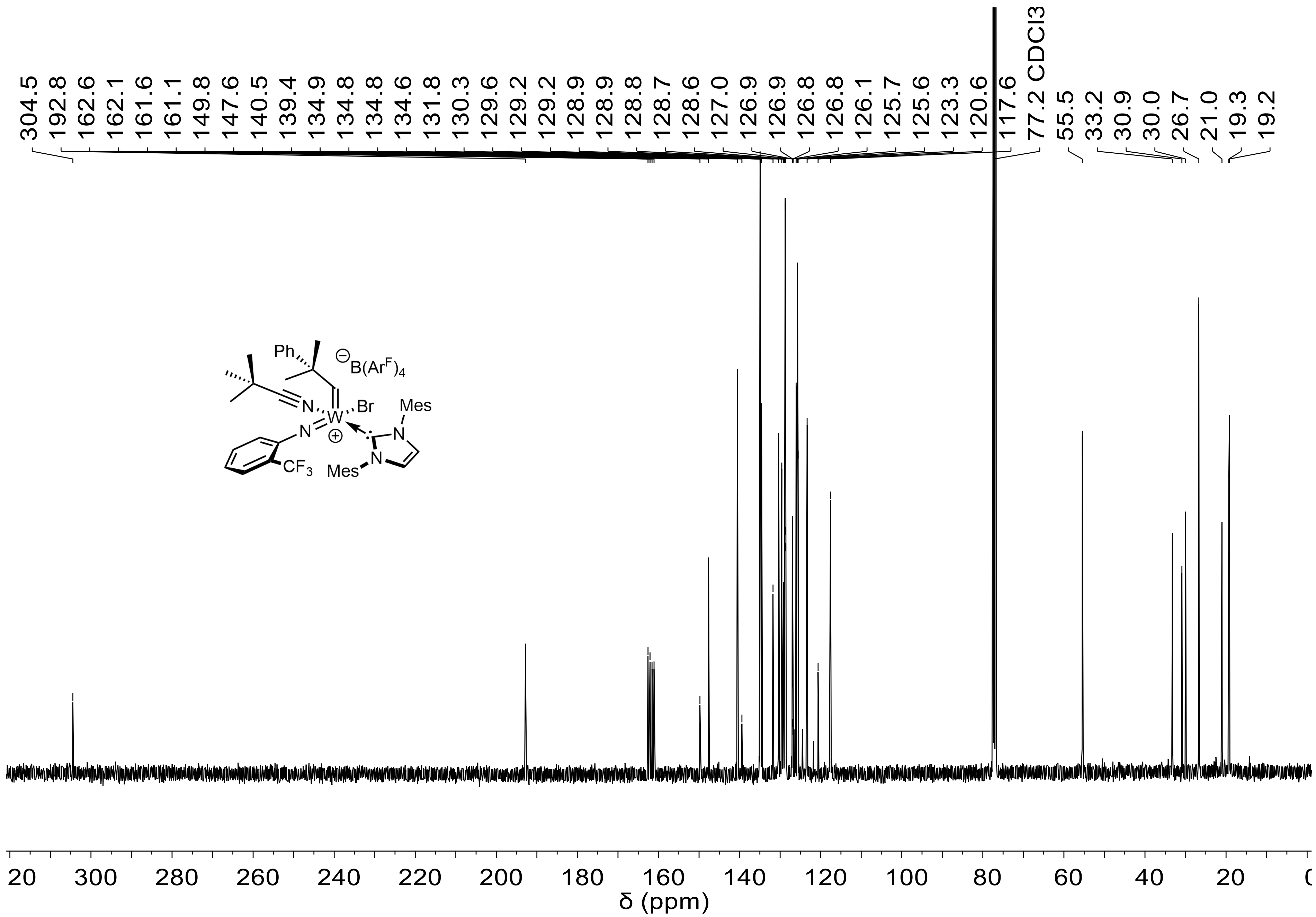

Figure S74: ^{13}C-NMR (101 MHz, 25 °C, $CDCl_3$) of W-26.

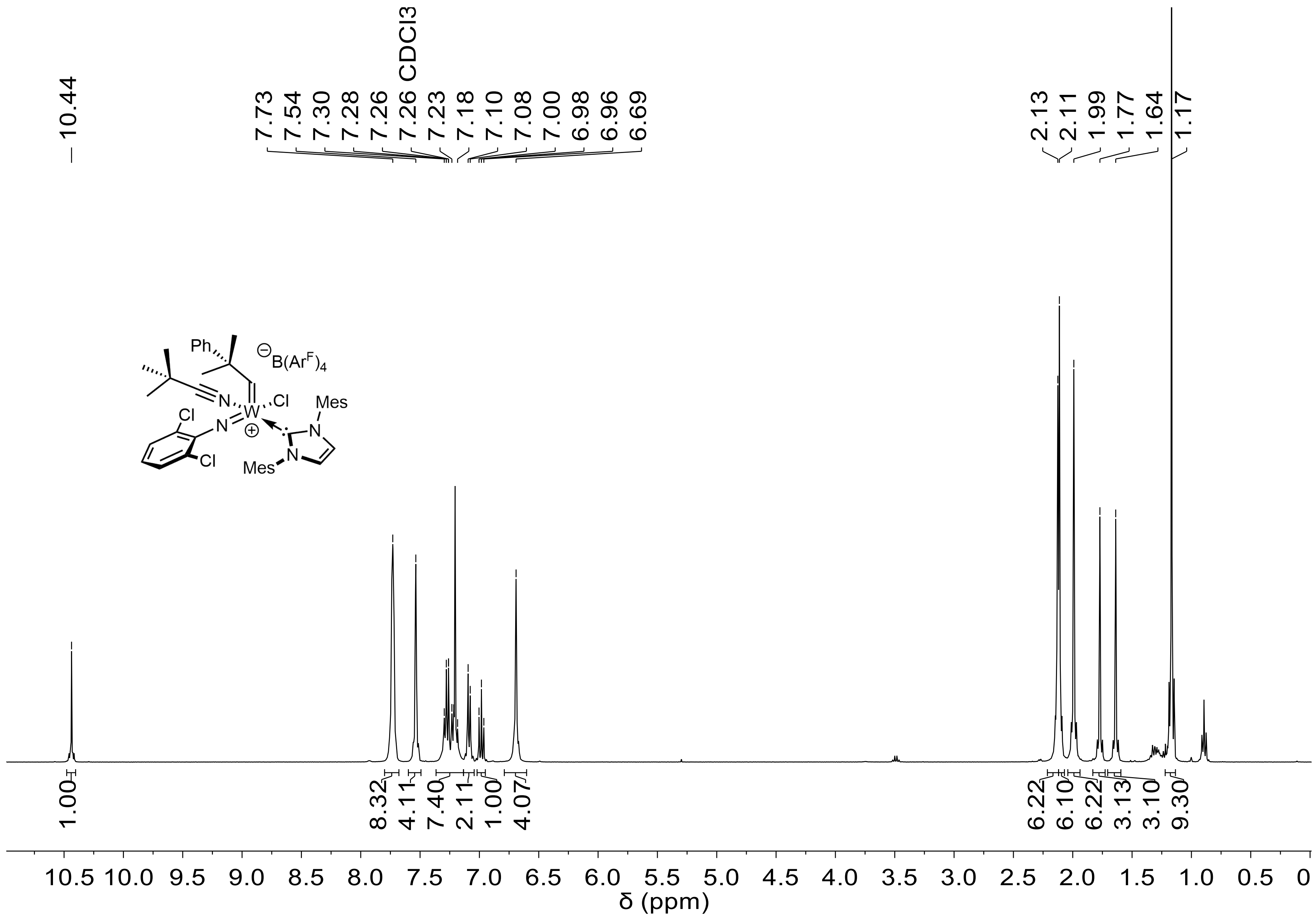

Figure S75: ^{1}H-NMR (400 MHz, 25 °C, $CDCl_3$) of W-27.

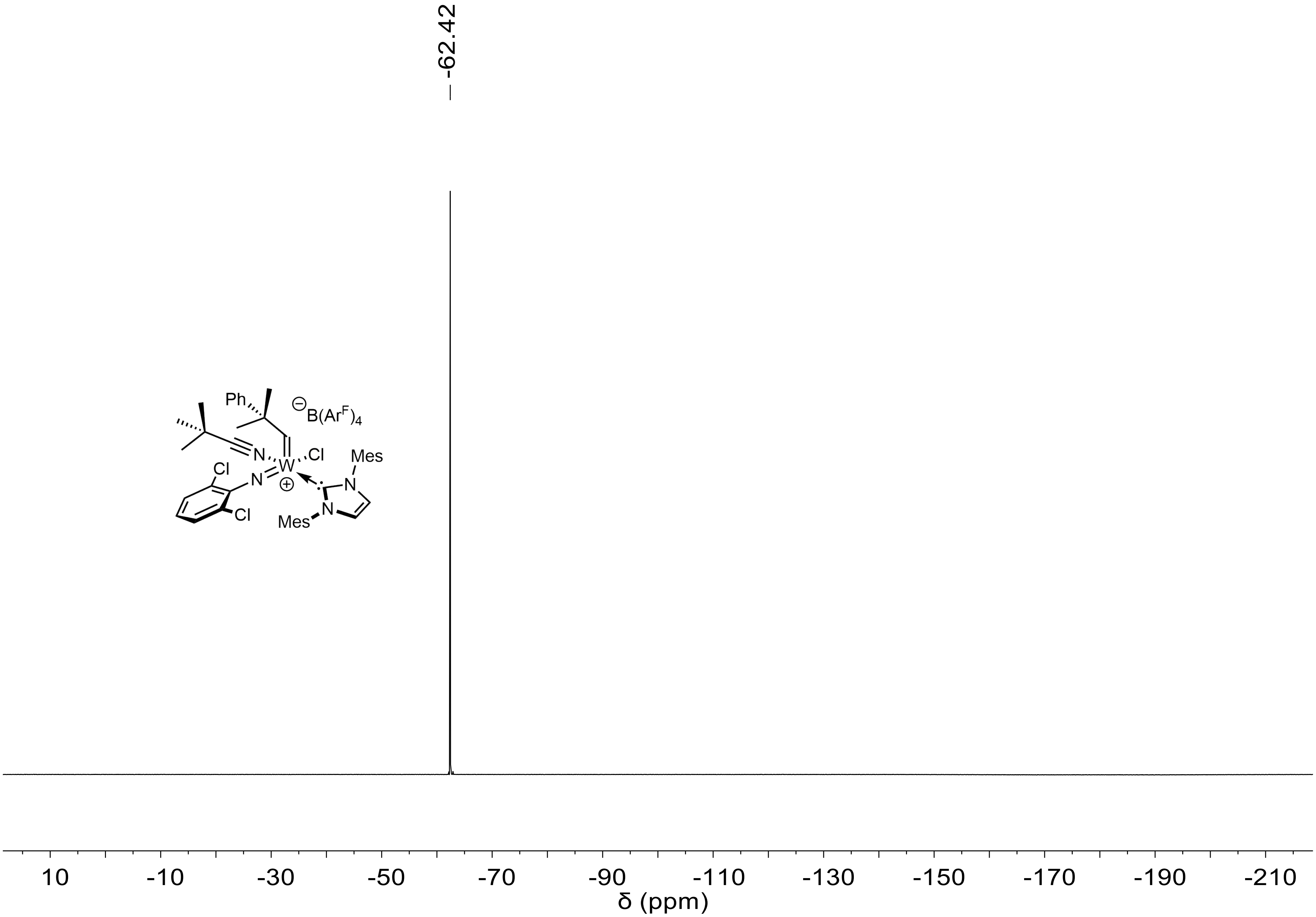

Figure S76: ^{19}F-NMR (376 MHz, 25 °C, $CDCl_3$) of W-27.

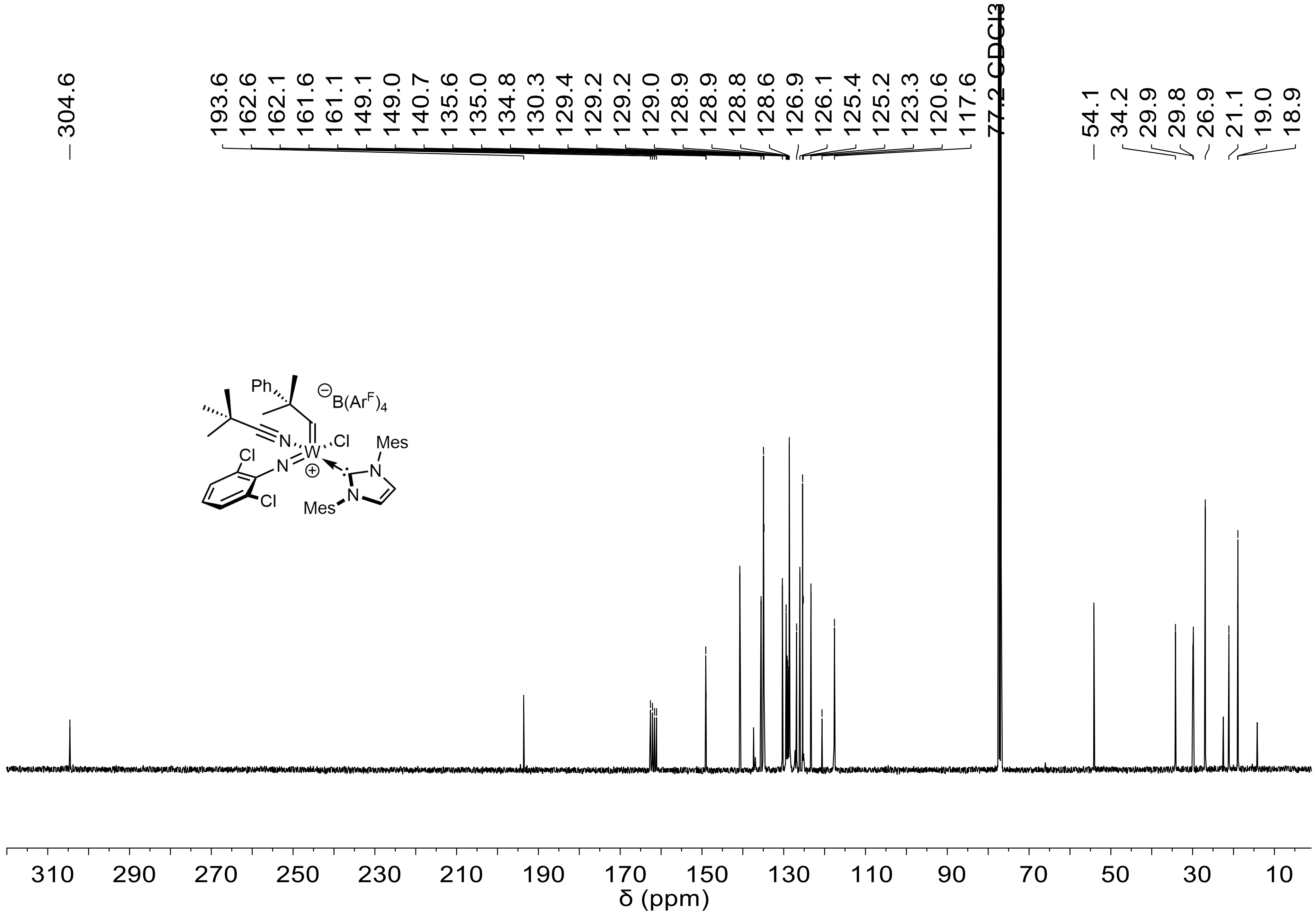

Figure S77: ^{13}C-NMR (101 MHz, 25 °C, $CDCl_3$) of W-27.

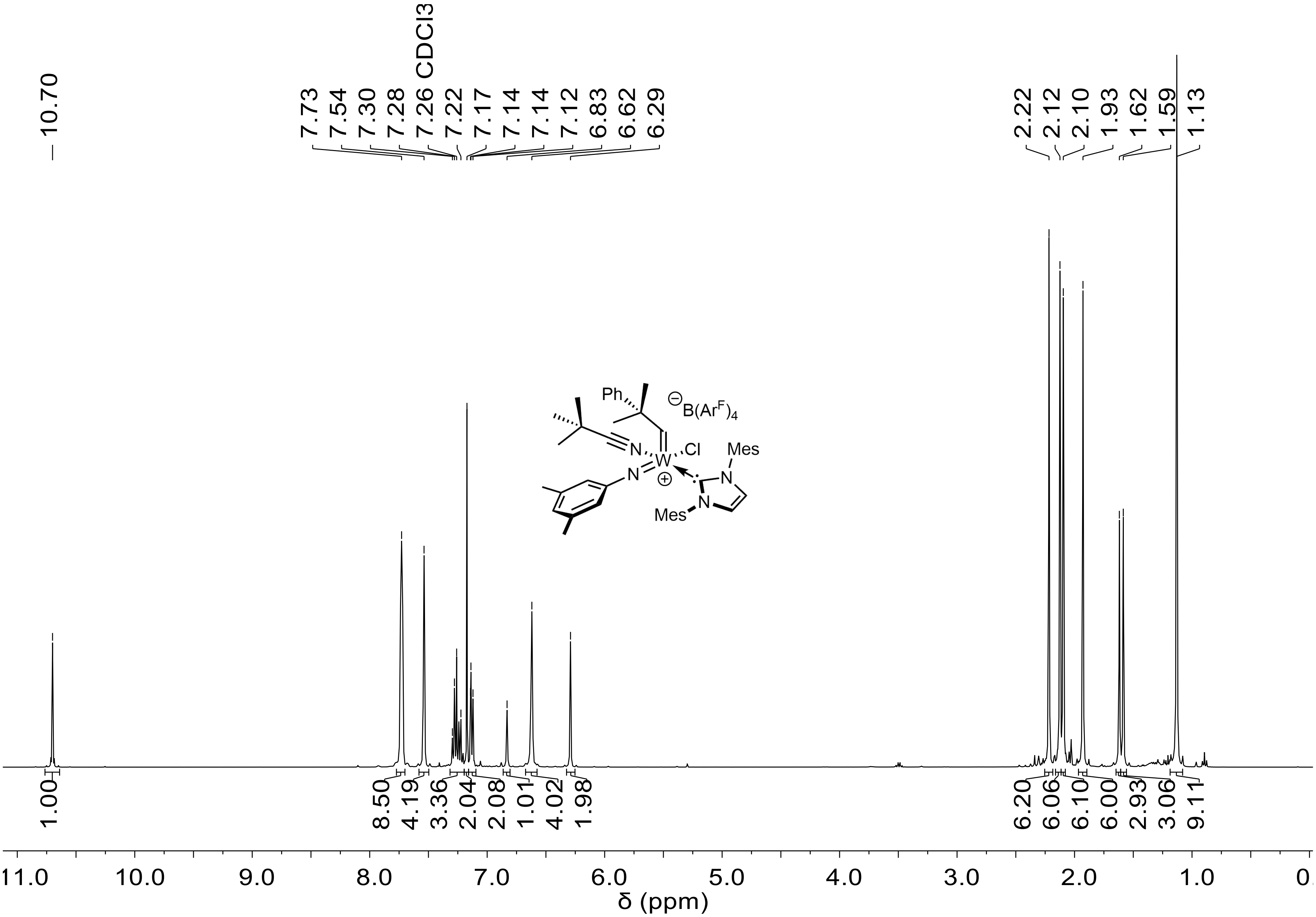

Figure S78: ^{1}H-NMR (400 MHz, 25 °C, $CDCl_3$) of W-28.

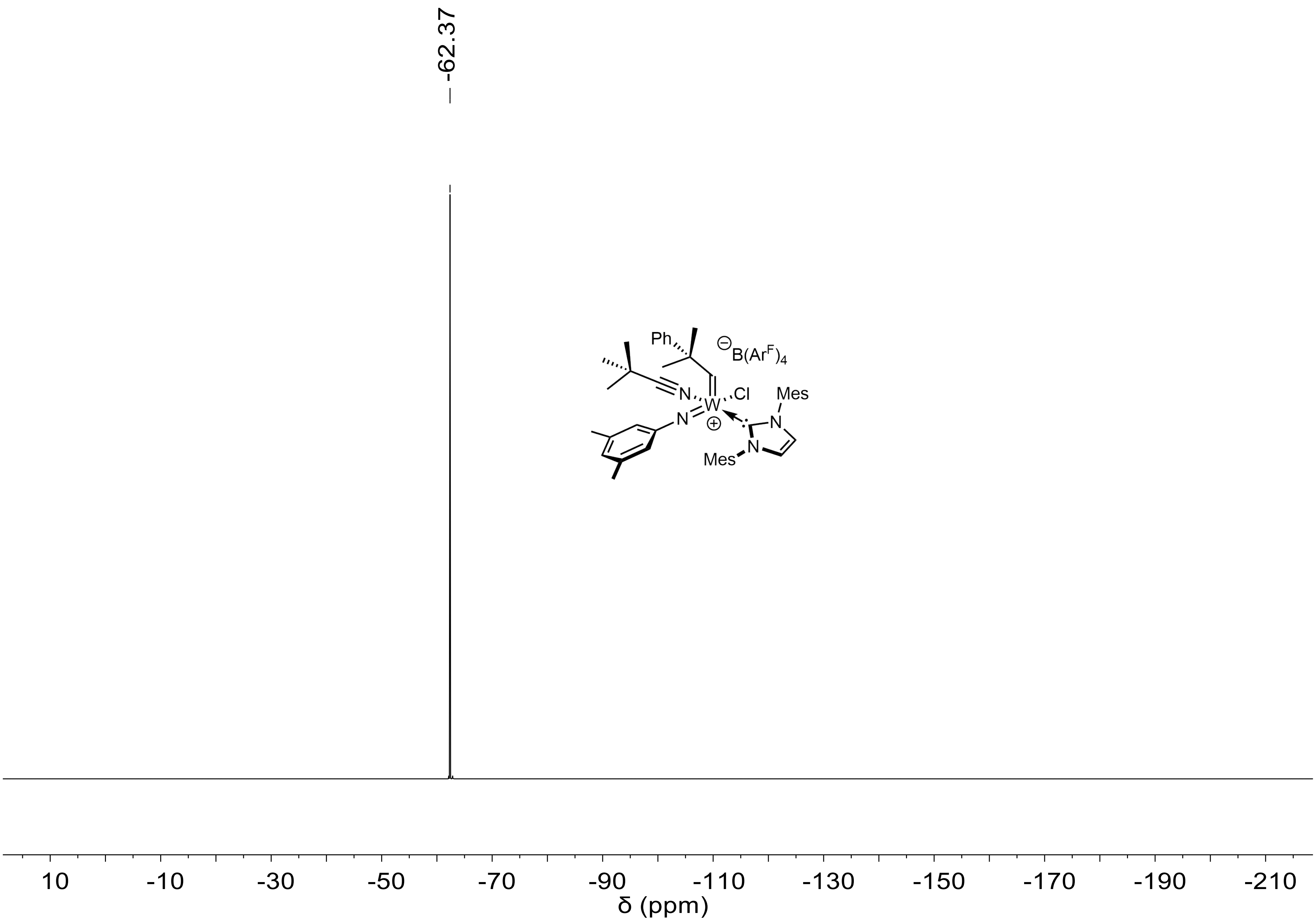

Figure S79: ^{19}F-NMR (376 MHz, 25 °C, $CDCl_3$) of W-28.

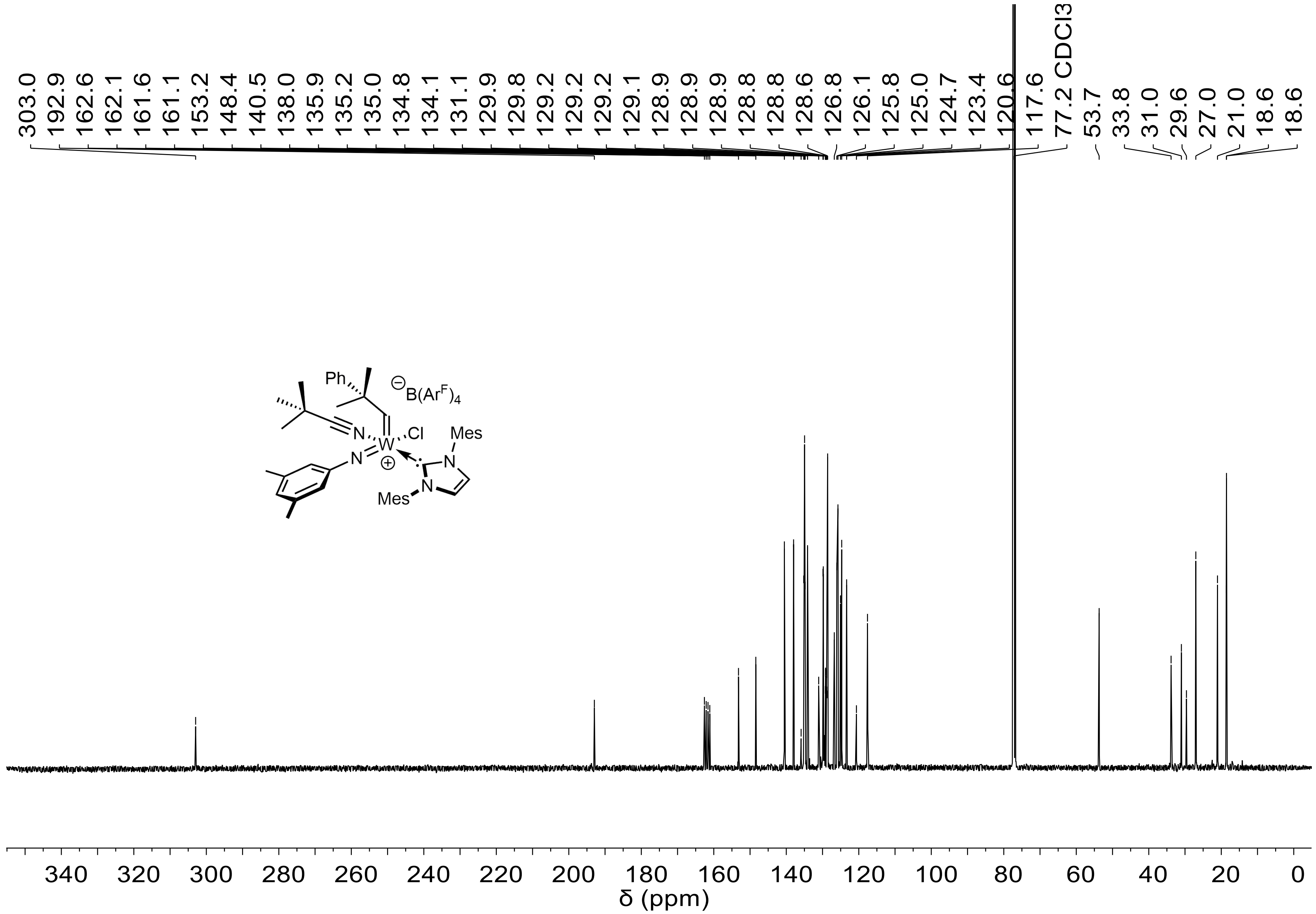

Figure S80: ^{13}C-NMR (101 MHz, 25 °C, CDCl$_3$) of W-28.

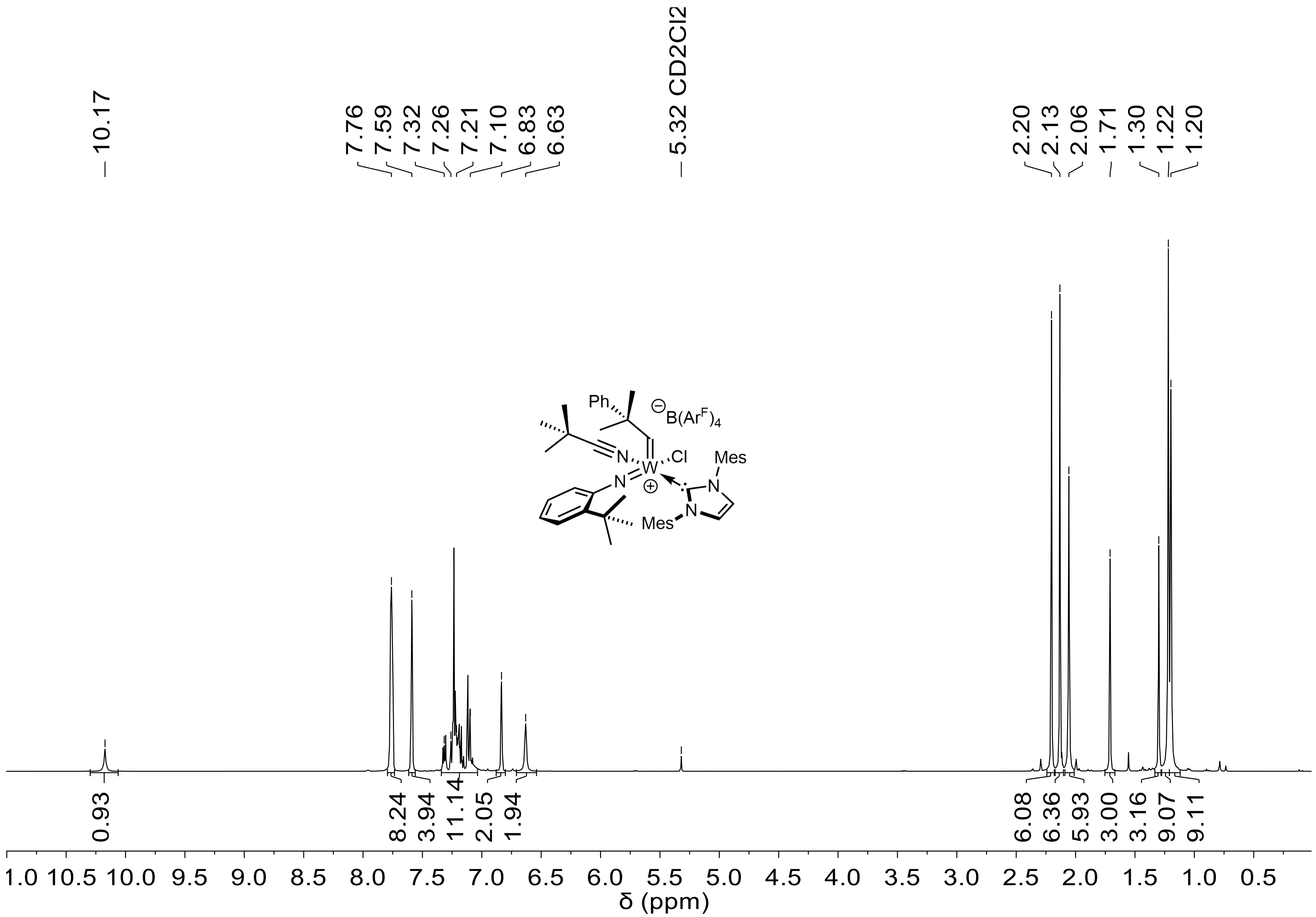

Figure S81: ^{1}H-NMR (400 MHz, 25 °C, CD_2Cl_2) of W-29.

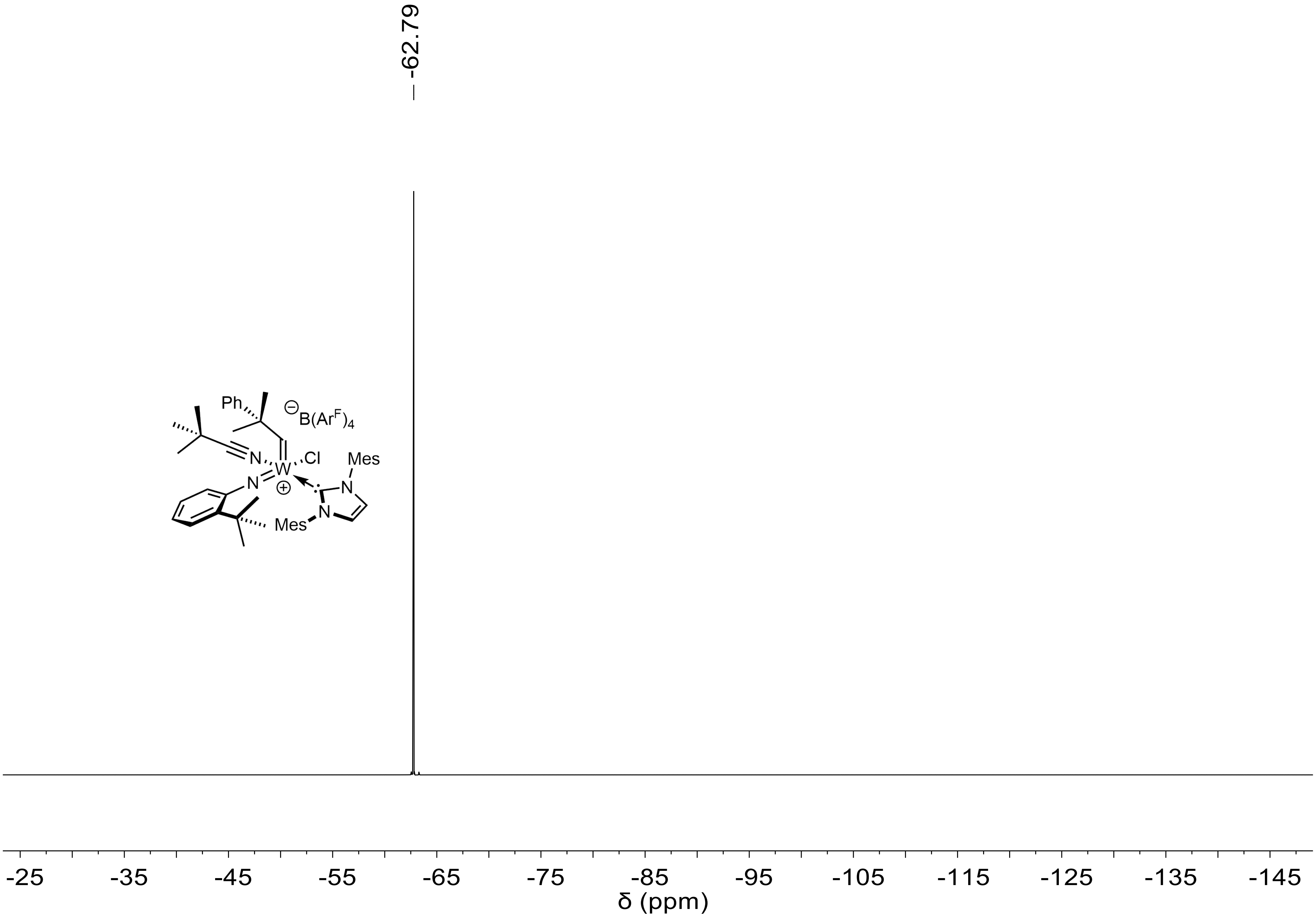

Figure S82: ^{19}F-NMR (376 MHz, 25 °C, CD_2Cl_2) of W-29.

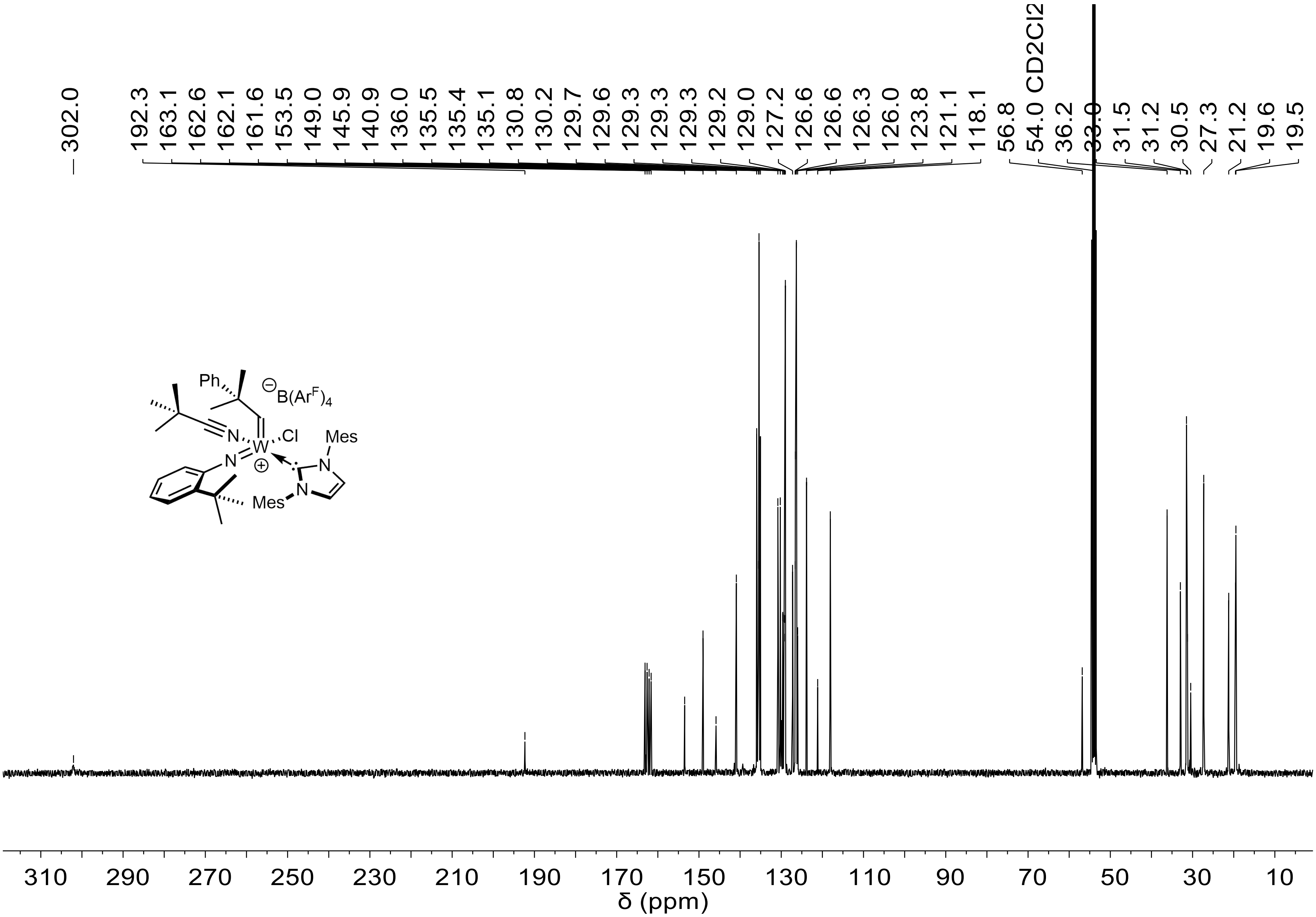

Figure S83: ^{13}C-NMR (101 MHz, 25 °C, CD_2Cl_2) of W-29.

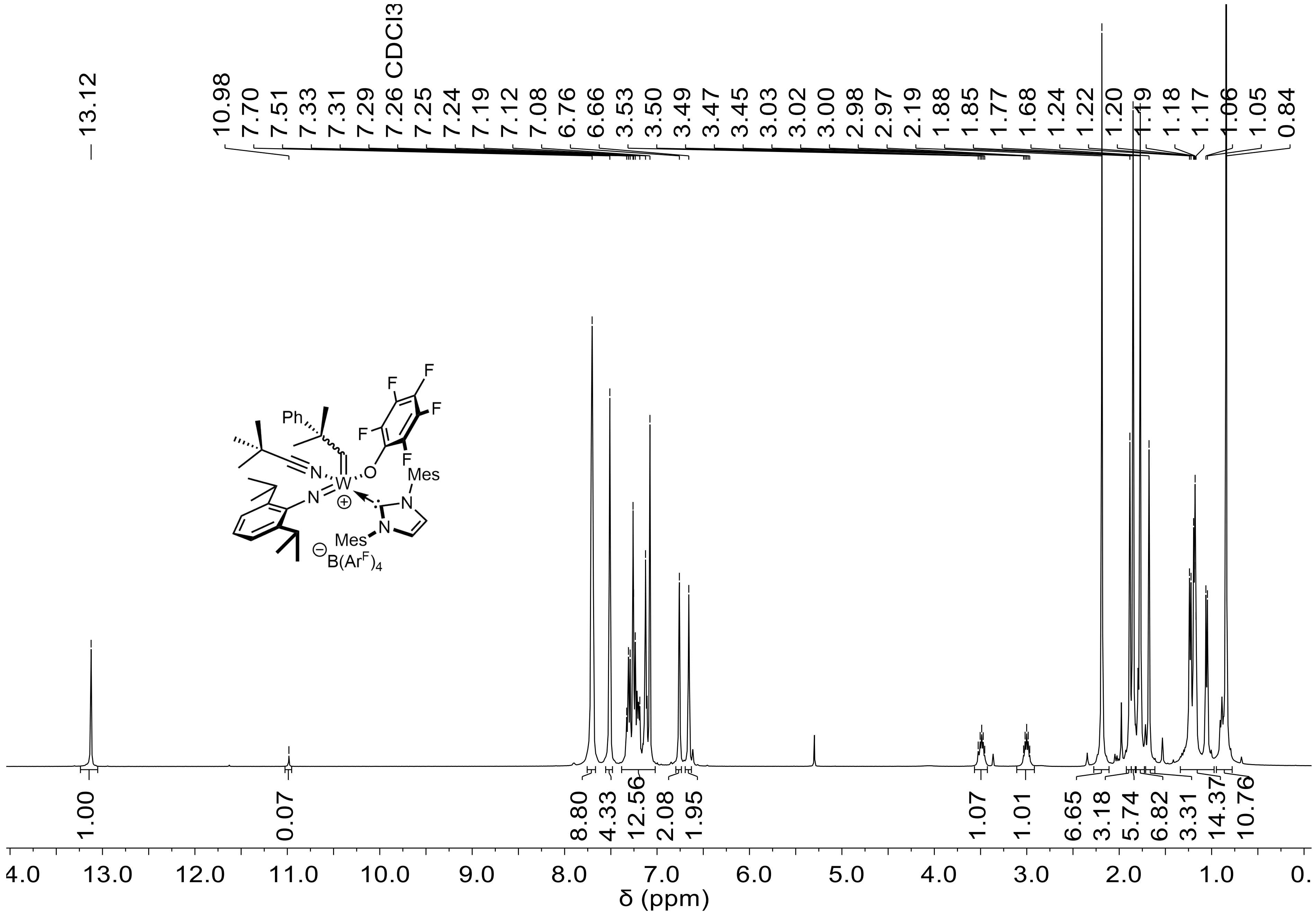

Figure S84: ¹H-NMR (400 MHz, 25 °C, CDCl$_3$) of W-30.

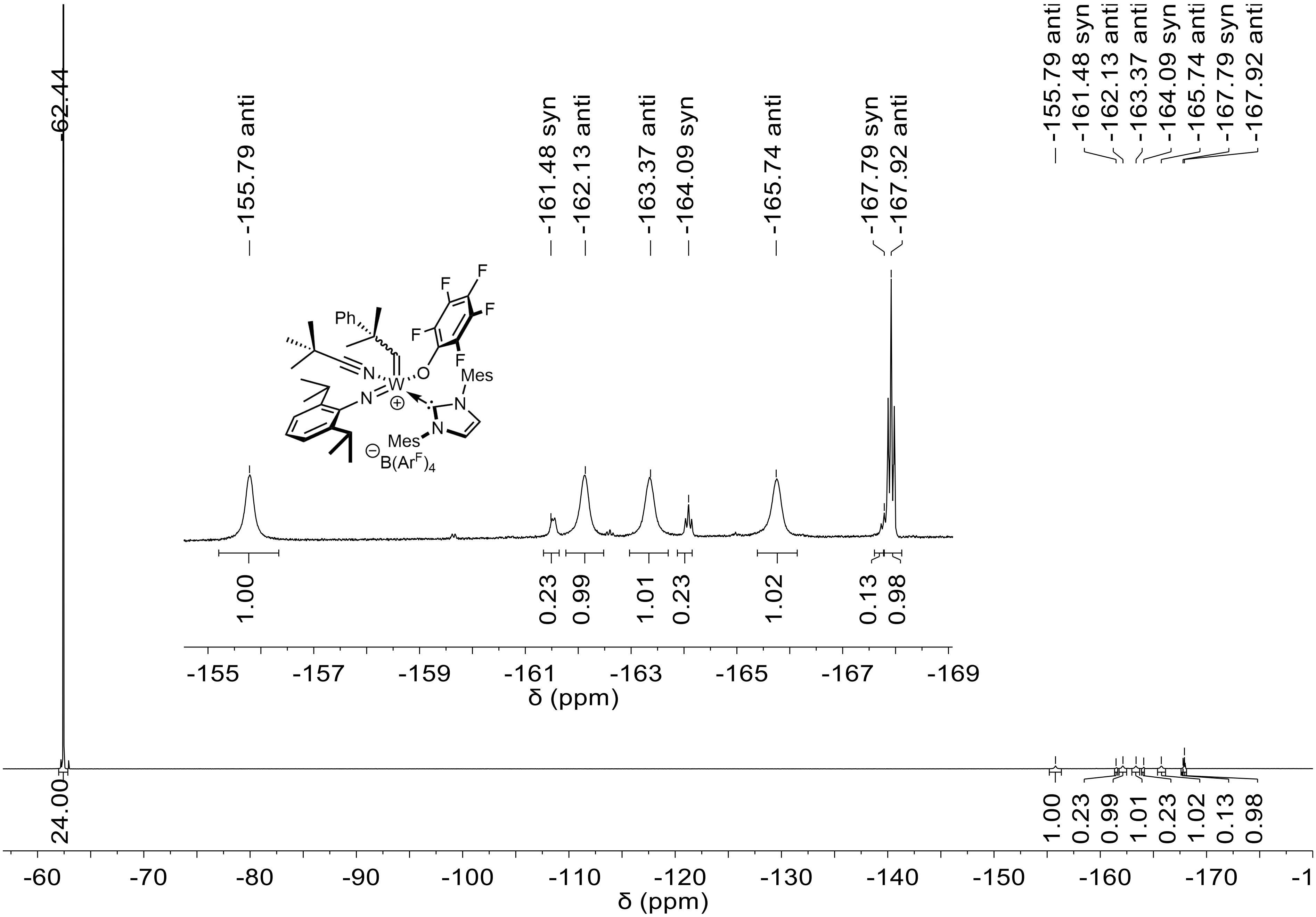

Figure S85: ^{19}F-NMR (376 MHz, 25 °C, $CDCl_3$) of W-30.

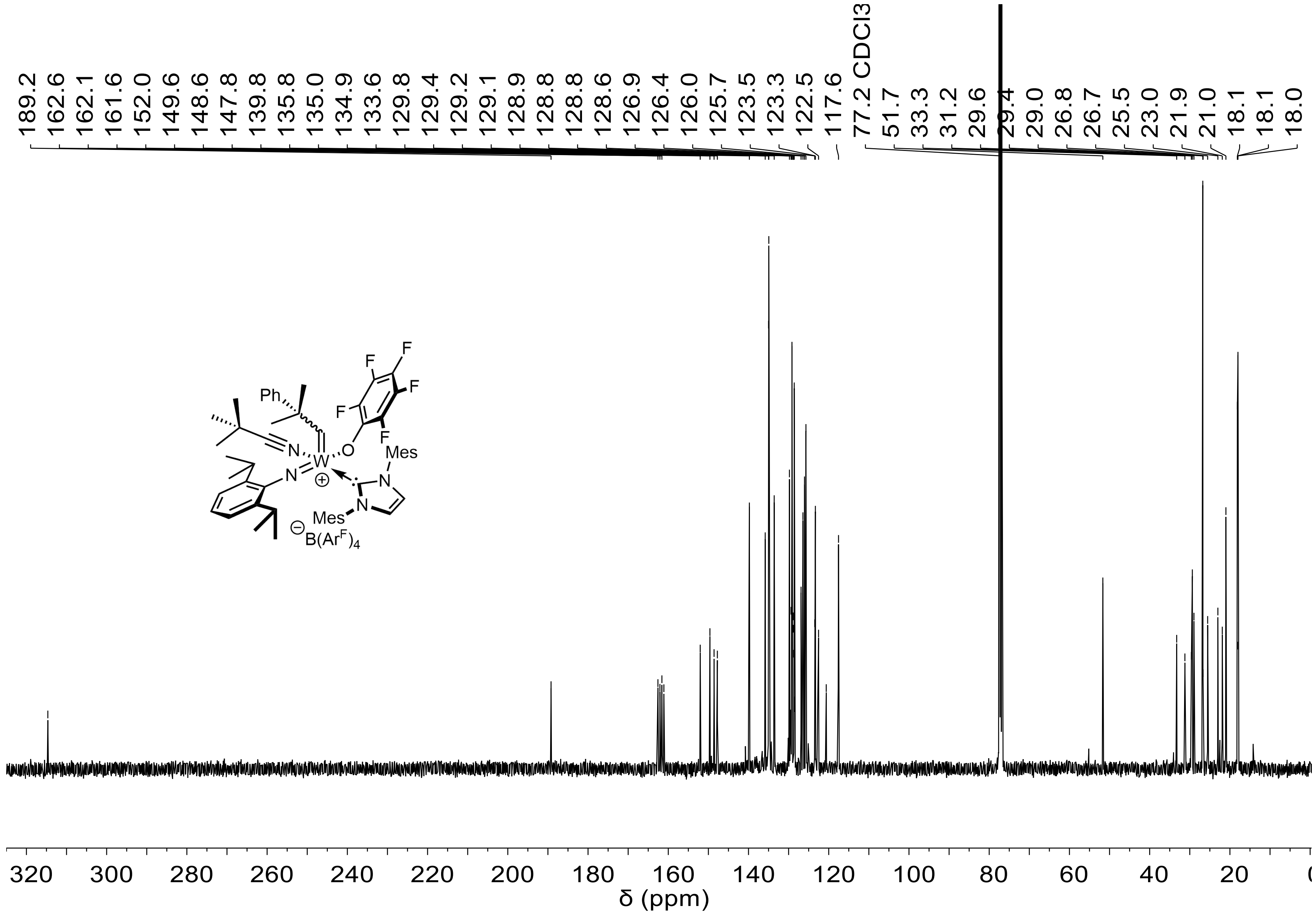

Figure S86: ^{13}C-NMR (101 MHz, 25 °C, $CDCl_3$) of W-30.

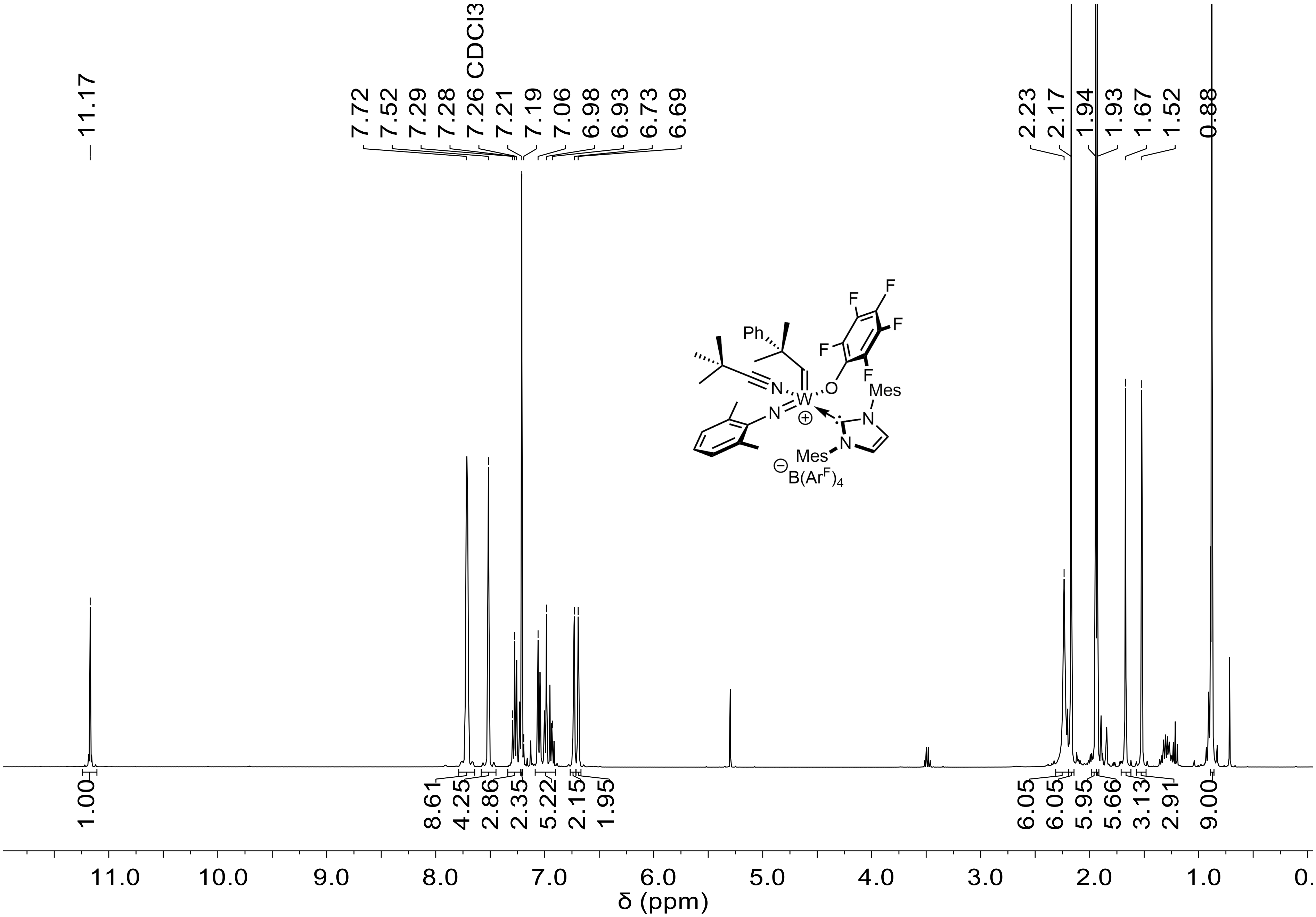

Figure S87: ^{1}H-NMR (400 MHz, 25 °C, $CDCl_3$) of W-31.

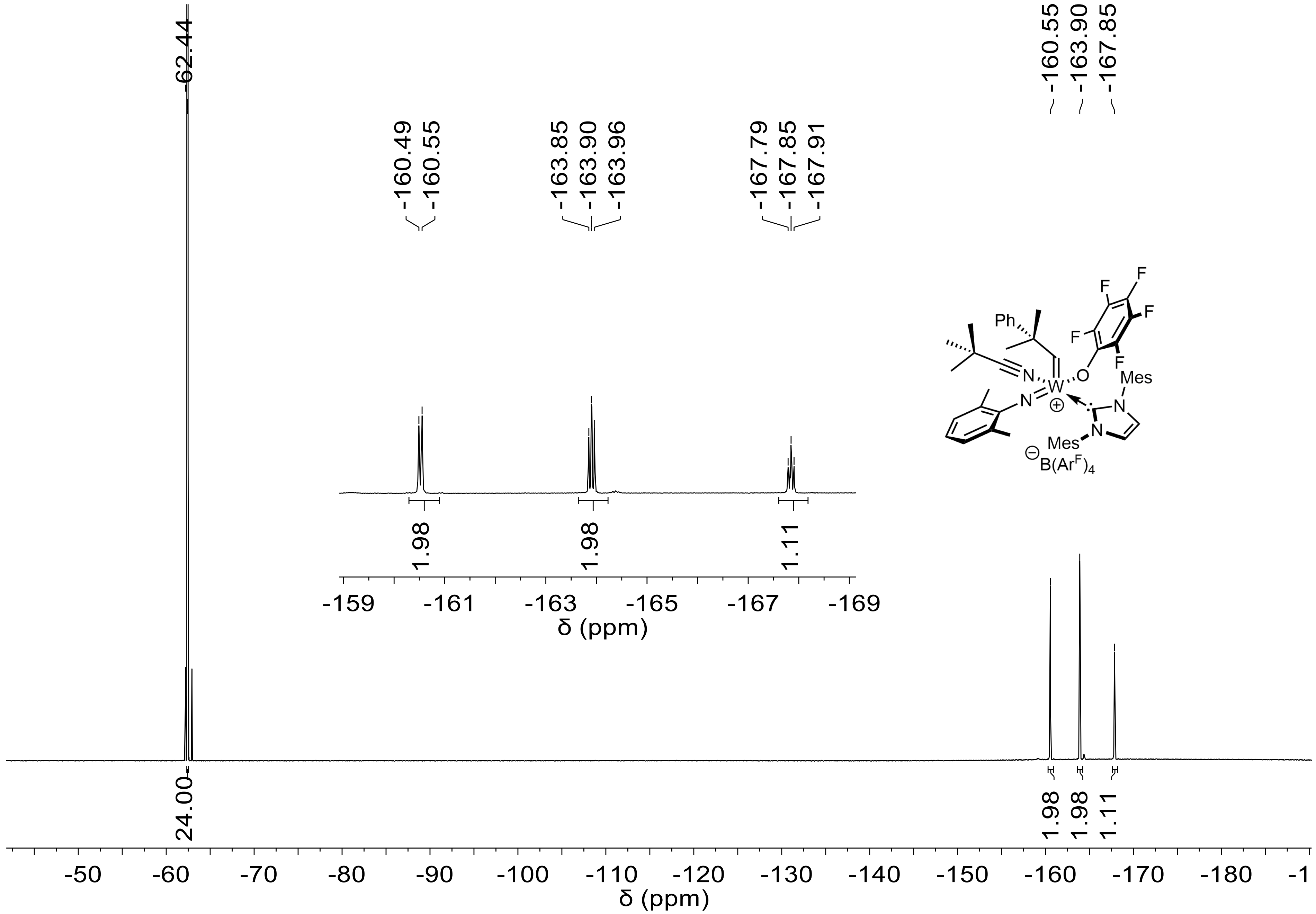

Figure S88: ^{19}F-NMR (376 MHz, 25 °C, CDCl$_3$) of W-31.

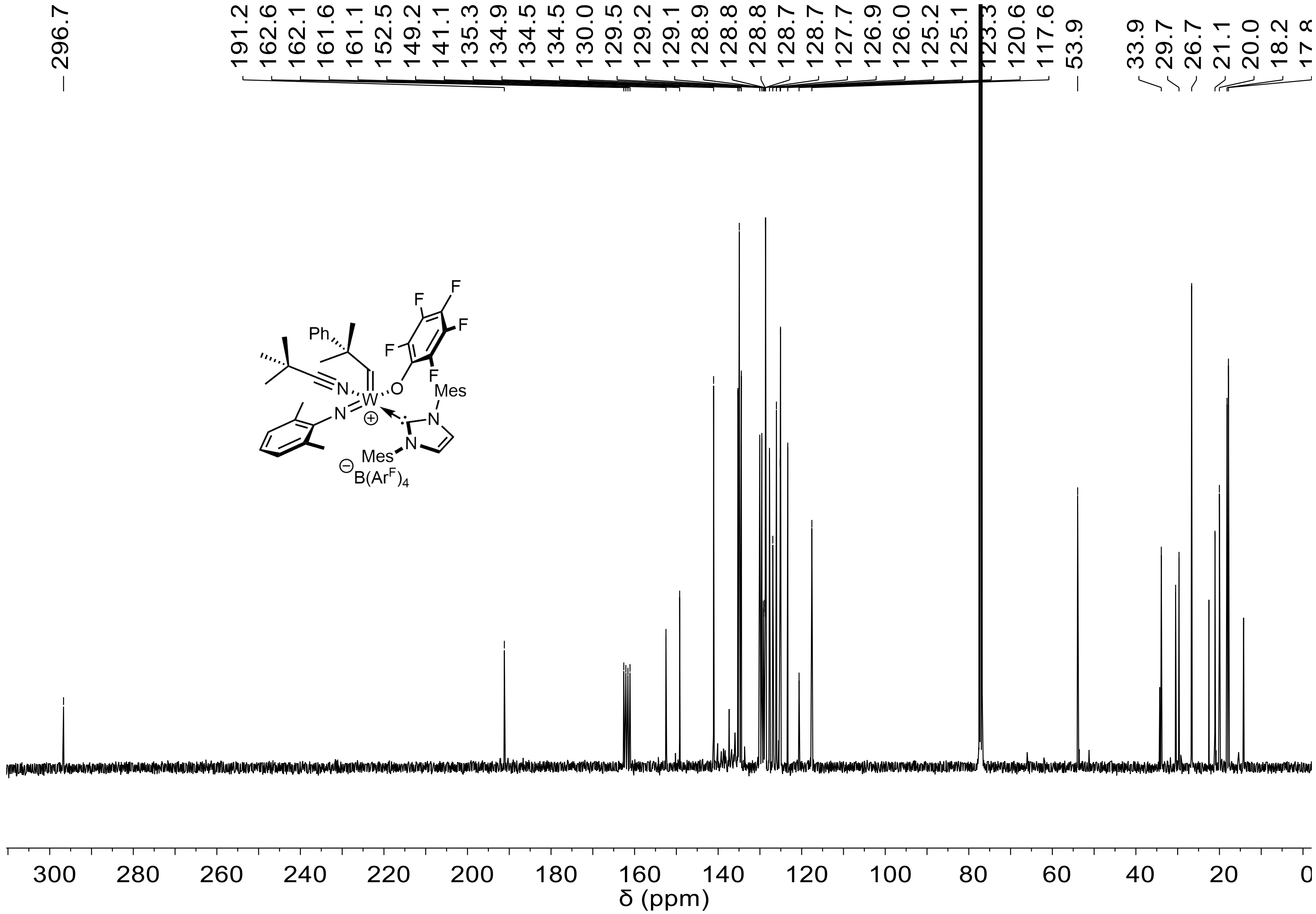

Figure S89: ^{13}C-NMR (101 MHz, 25 °C, CDCl$_3$) of W-31.

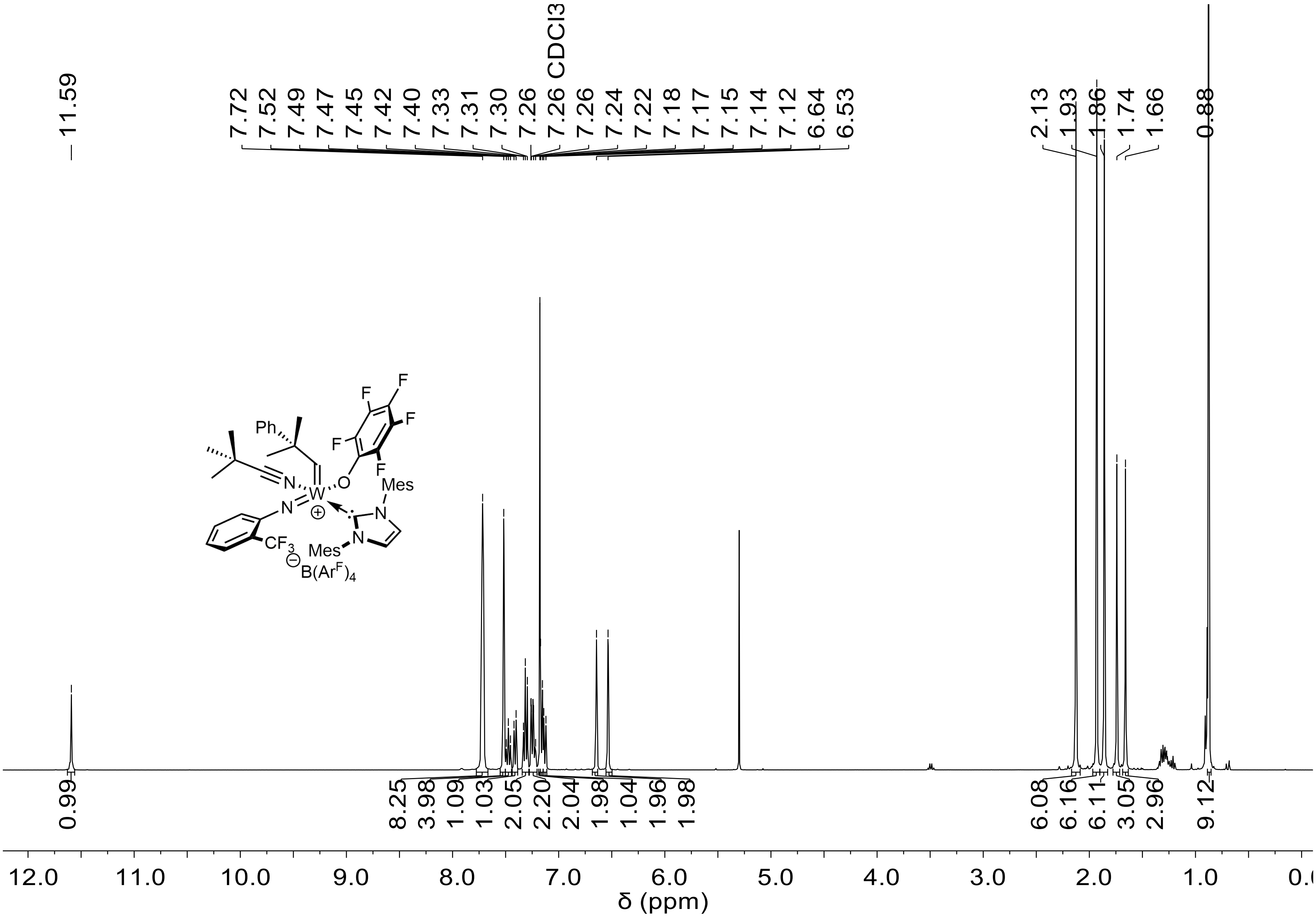

Figure S90: ^{1}H-NMR (400 MHz, 25 °C, $CDCl_3$) of W-32.

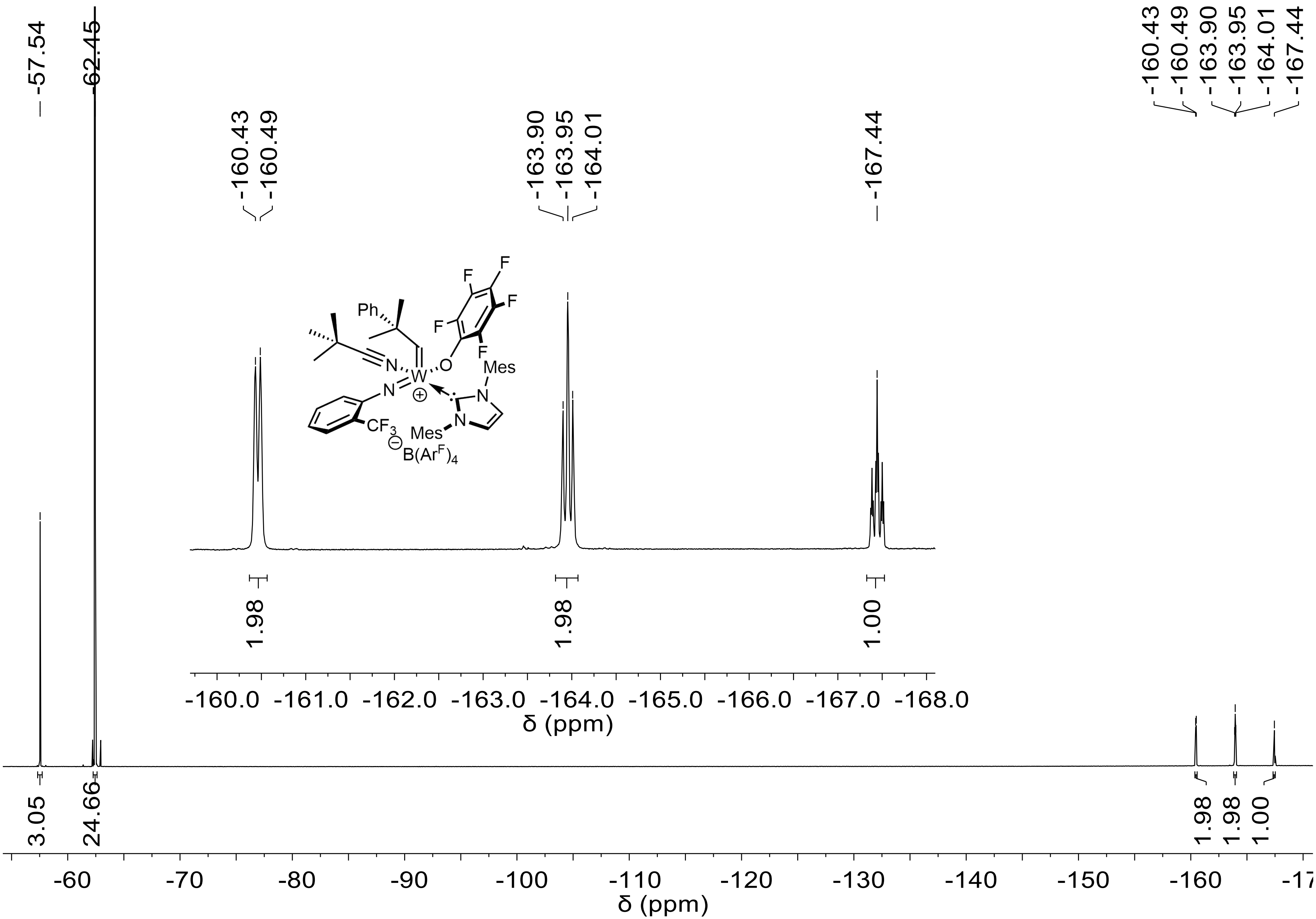

Figure S91: ^{19}F-NMR (376 MHz, 25 °C, $CDCl_3$) of W-32.

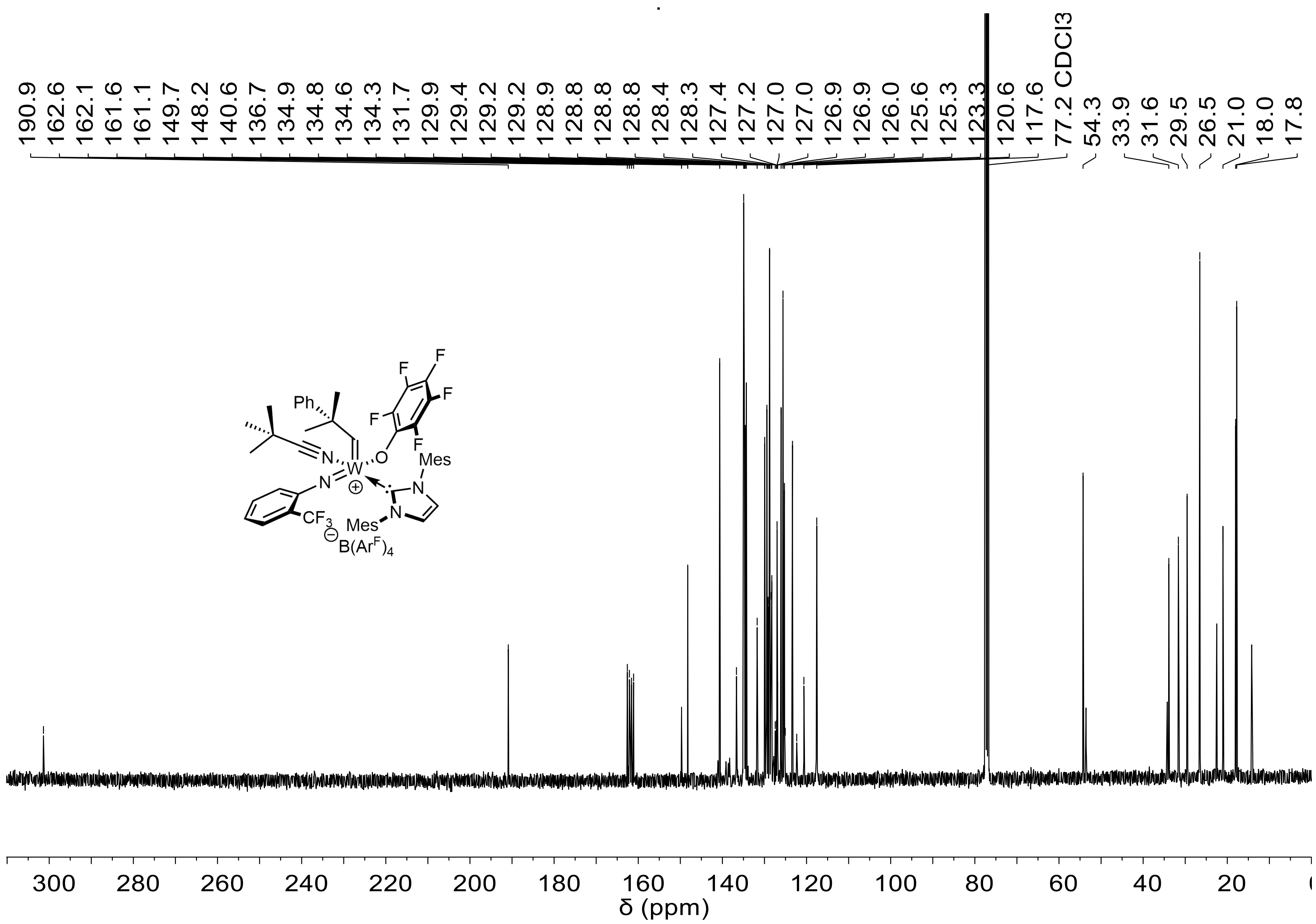

Figure S92: ^{13}C-NMR (101 MHz, 25 °C, $CDCl_3$) of W-32.

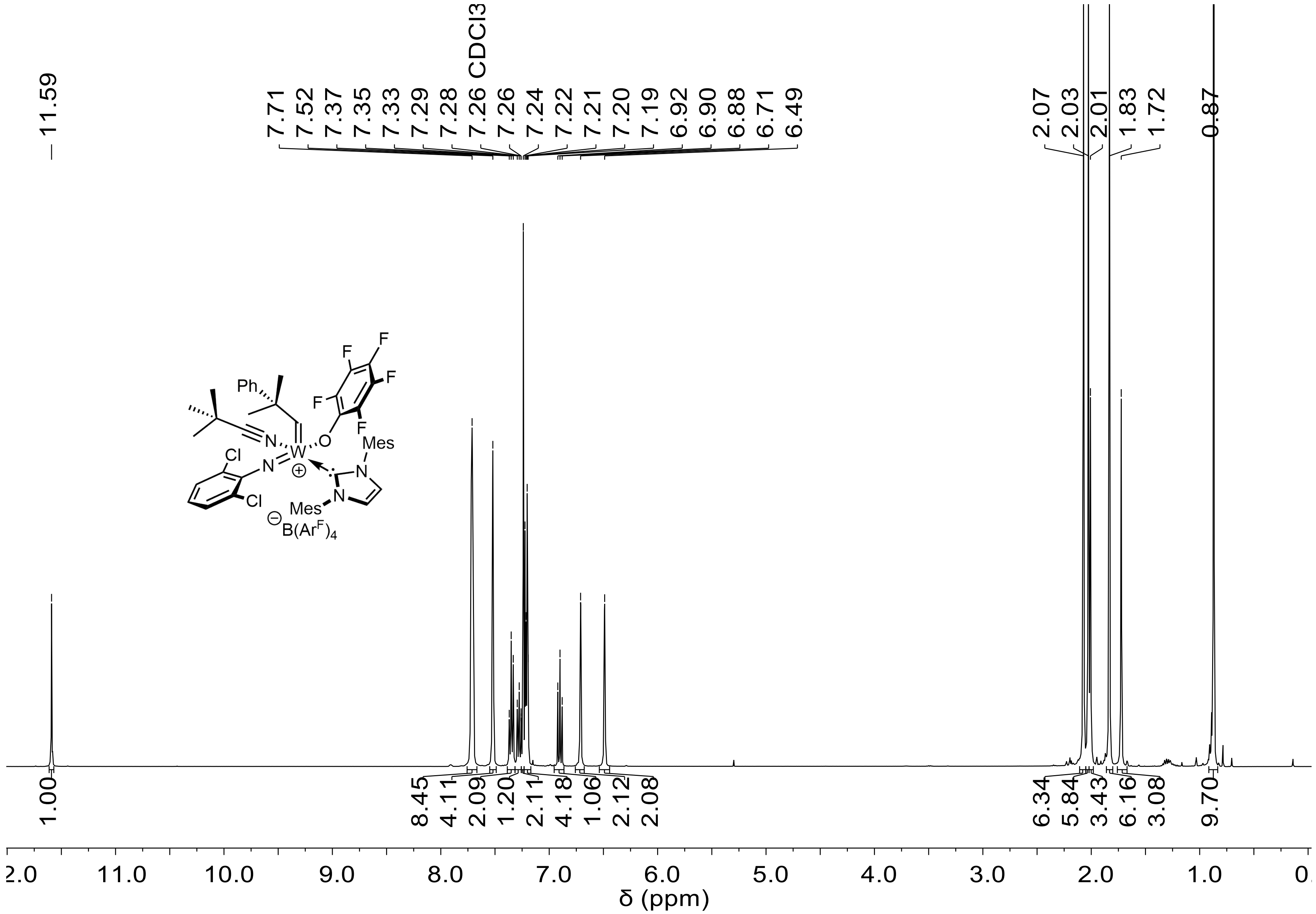

Figure S93: ^{1}H-NMR (400 MHz, 25 °C, $CDCl_3$) of W-33.

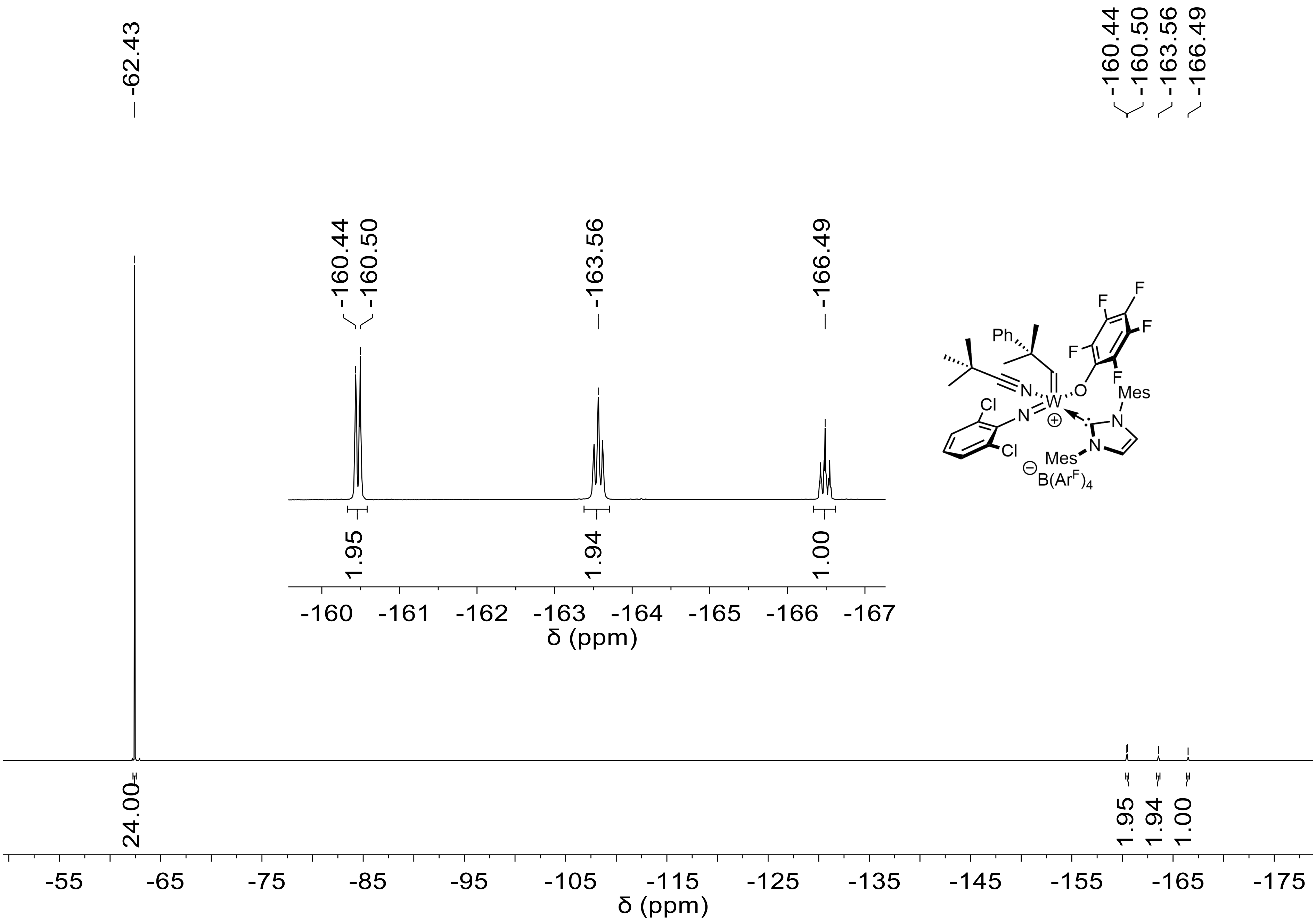

Figure S94: ^{19}F-NMR (376 MHz, 25 °C, CDCl$_3$) of W-33.

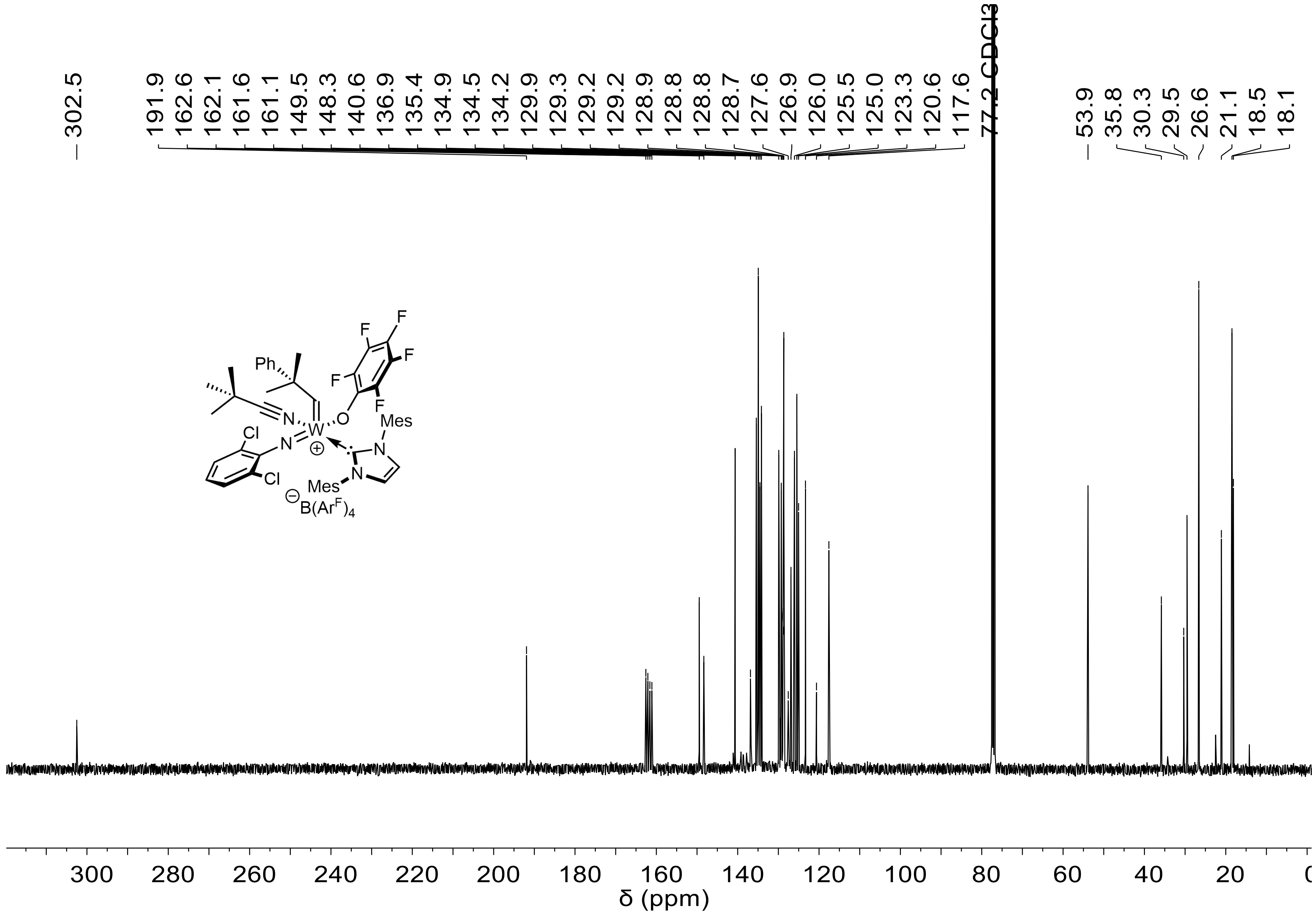

Figure S95: ^{13}C-NMR (101 MHz, 25 °C, CDCl$_3$) of W-33.

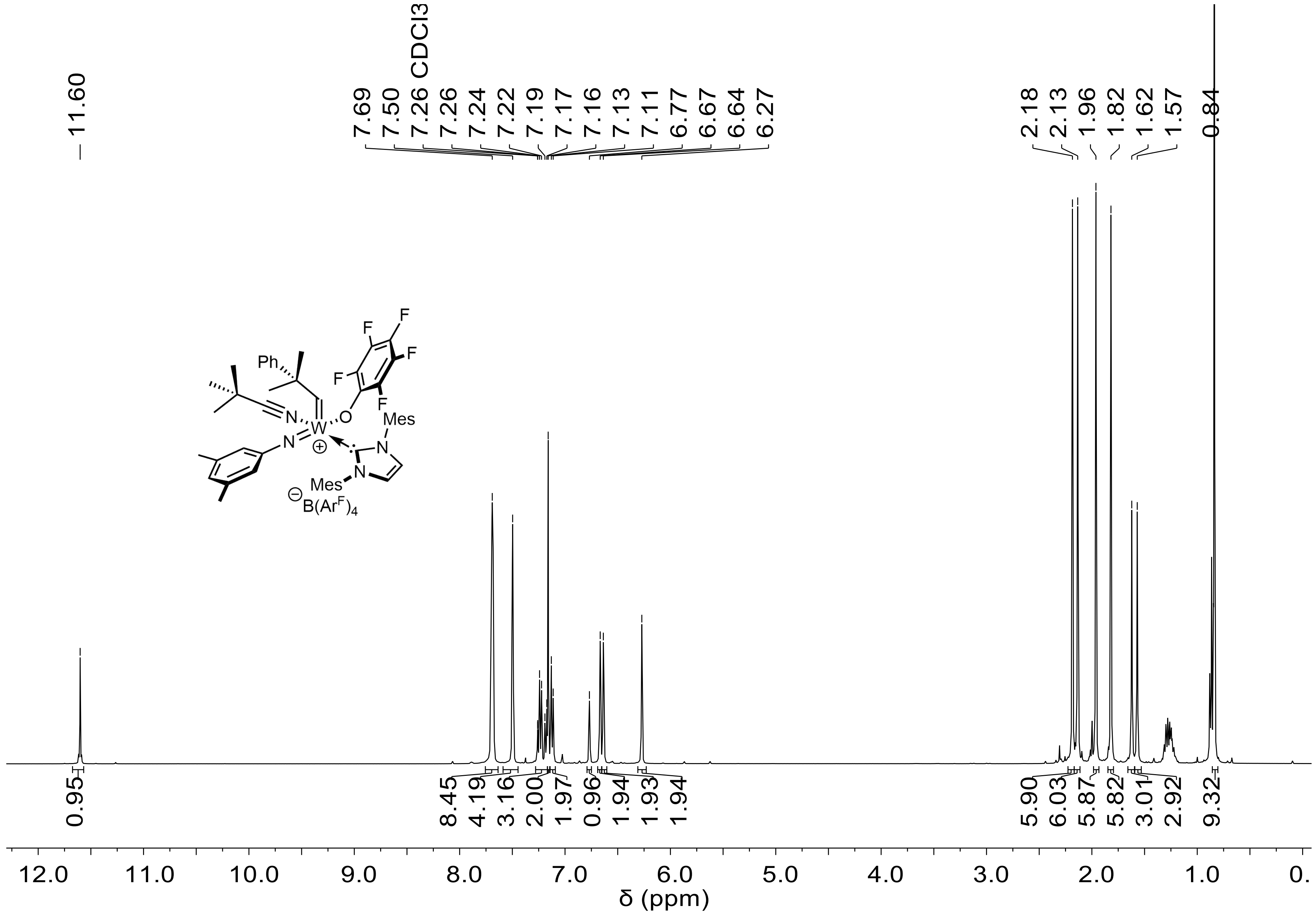

Figure S96: ^{1}H-NMR (400 MHz, 25 °C, $CDCl_3$) of W-34.

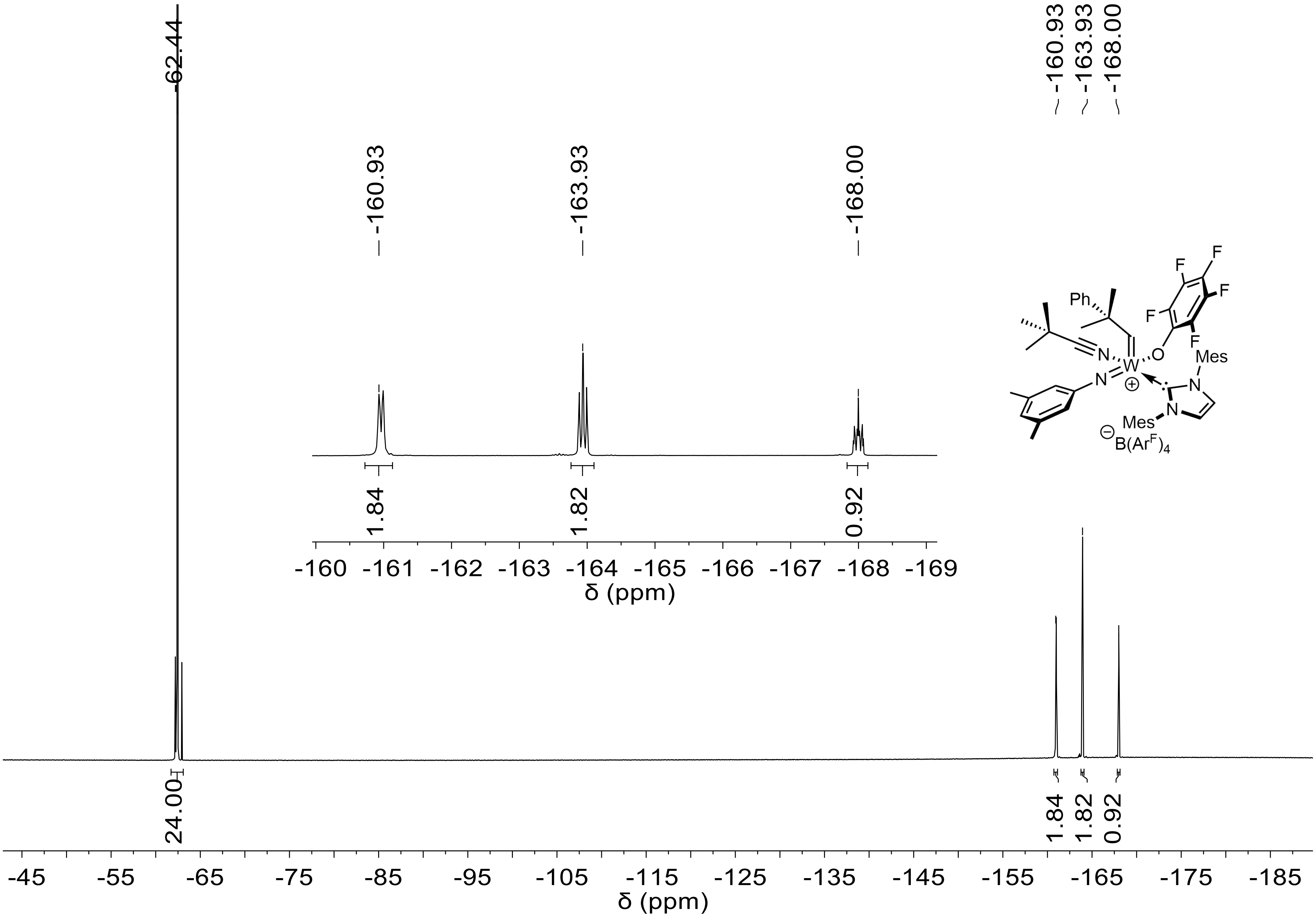

Figure S97: ^{19}F-NMR (376 MHz, 25 °C, CDCl$_3$) of W-34.

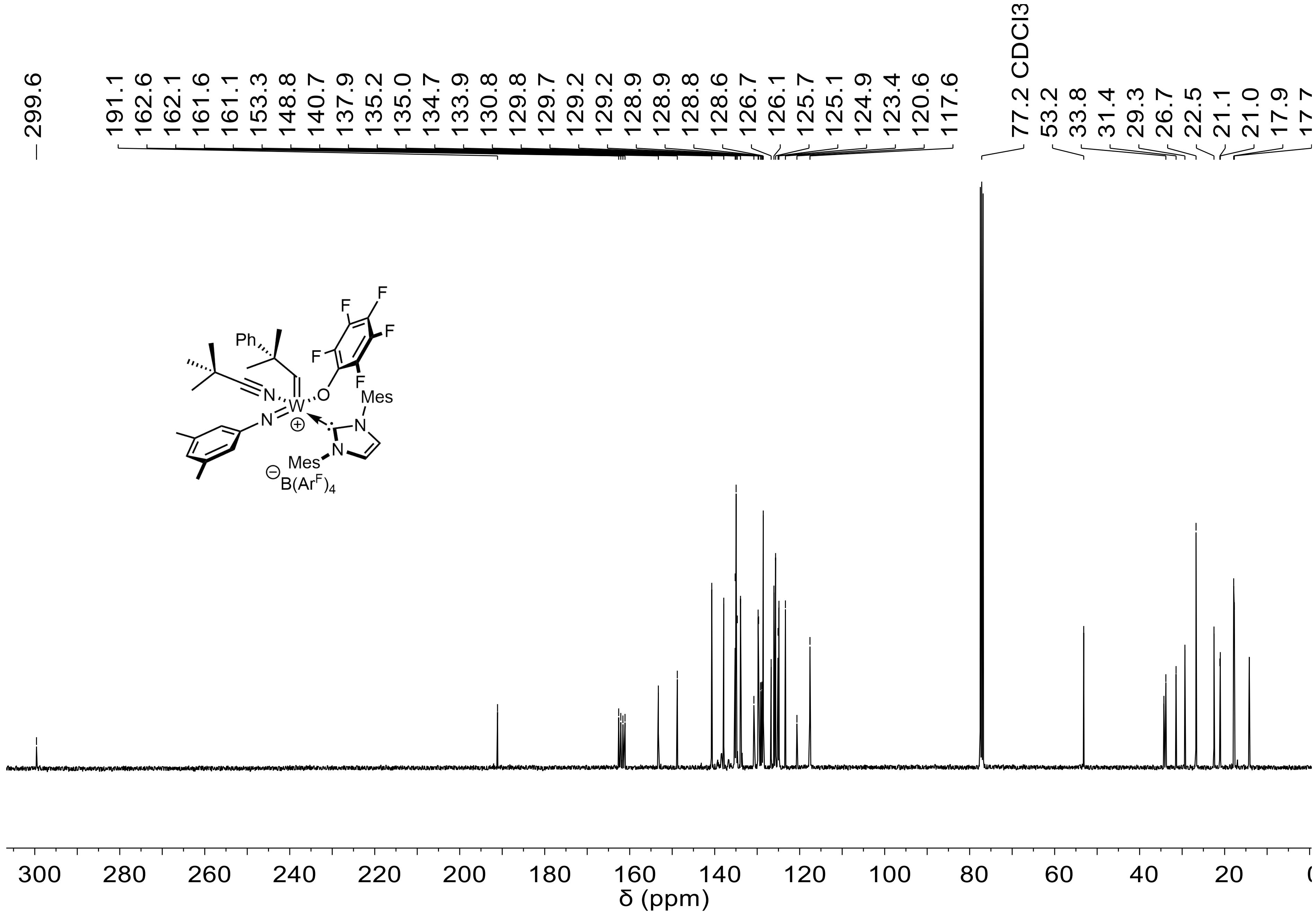

Figure S98: ^{13}C-NMR (101 MHz, 25 °C, $CDCl_3$) of W-34.

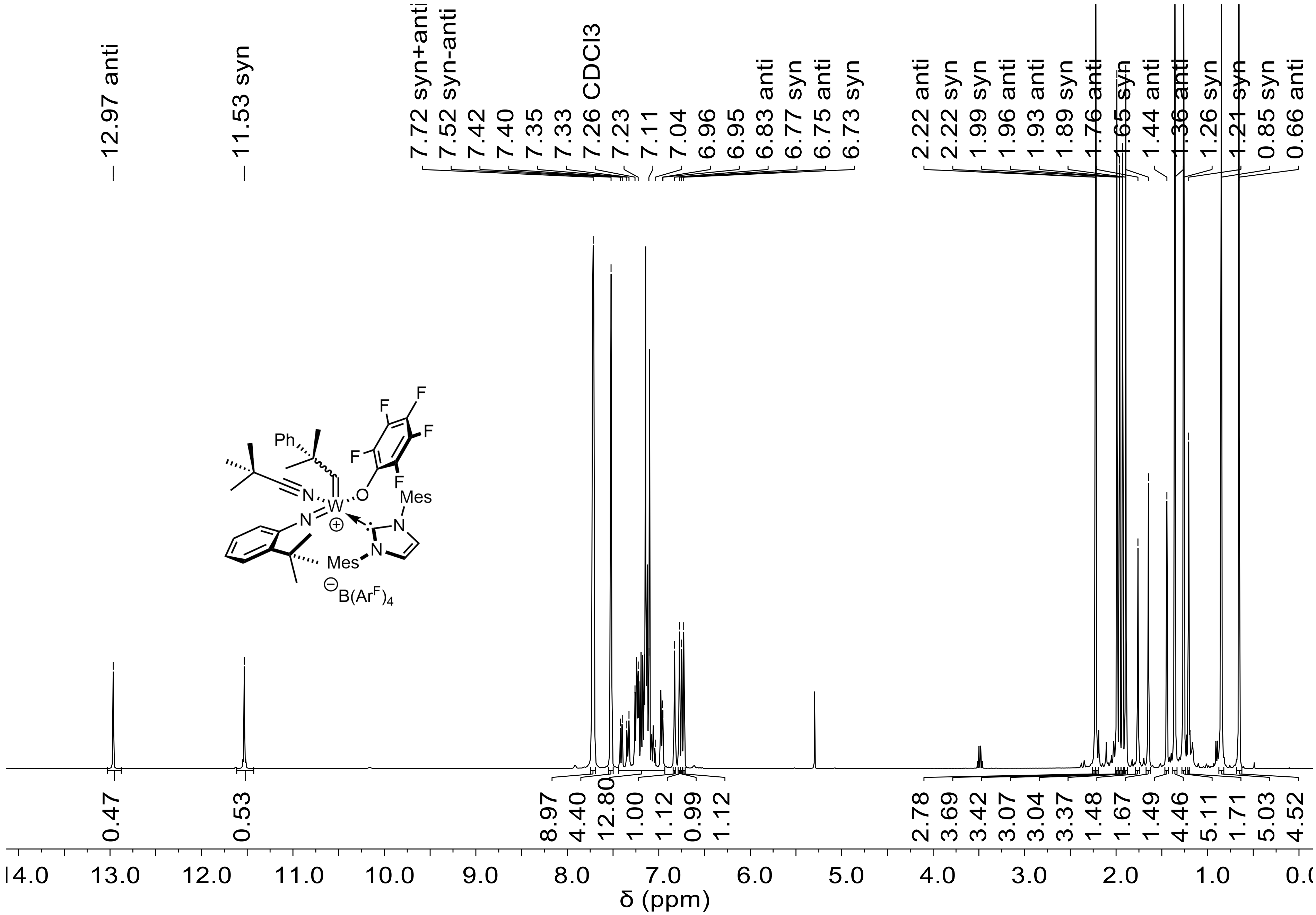

Figure S99: ^{1}H-NMR (400 MHz, 25 °C, $CDCl_3$) of W-35.

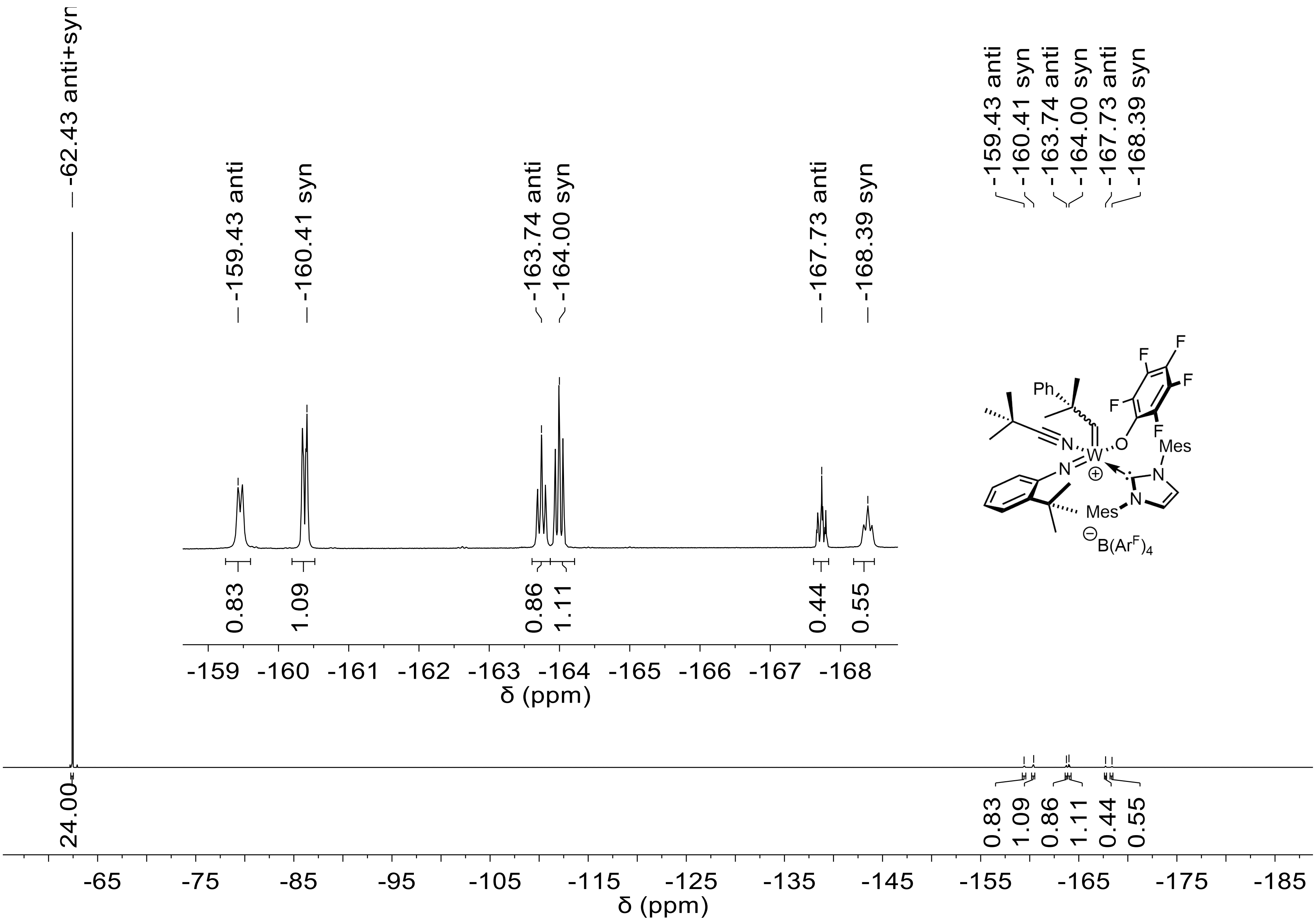

Figure S100: ^{19}F-NMR (376 MHz, 25 °C, $CDCl_3$) of W-35.

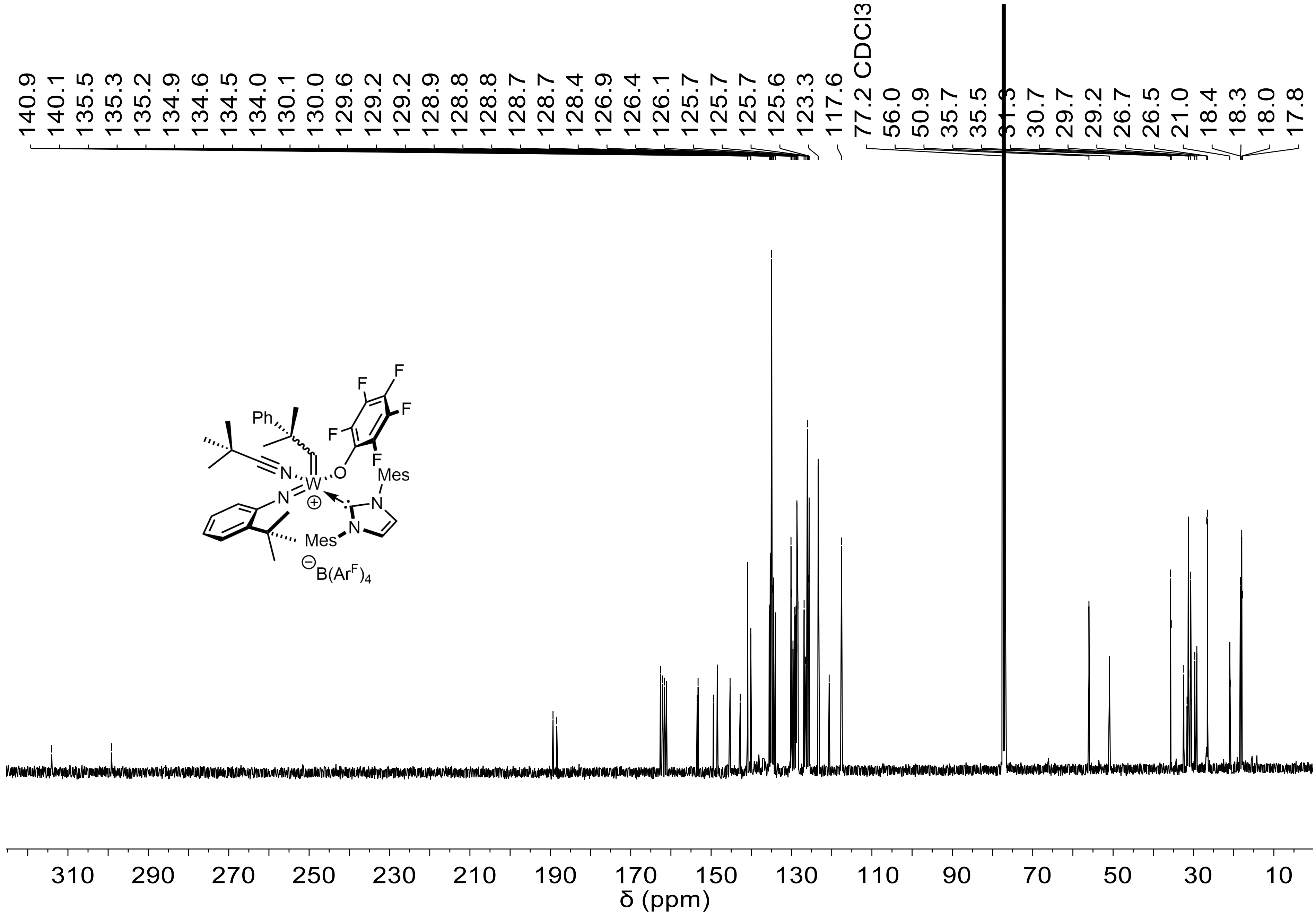

Figure S101: ^{13}C-NMR (101 MHz, 25 °C, $CDCl_3$) of W-35.

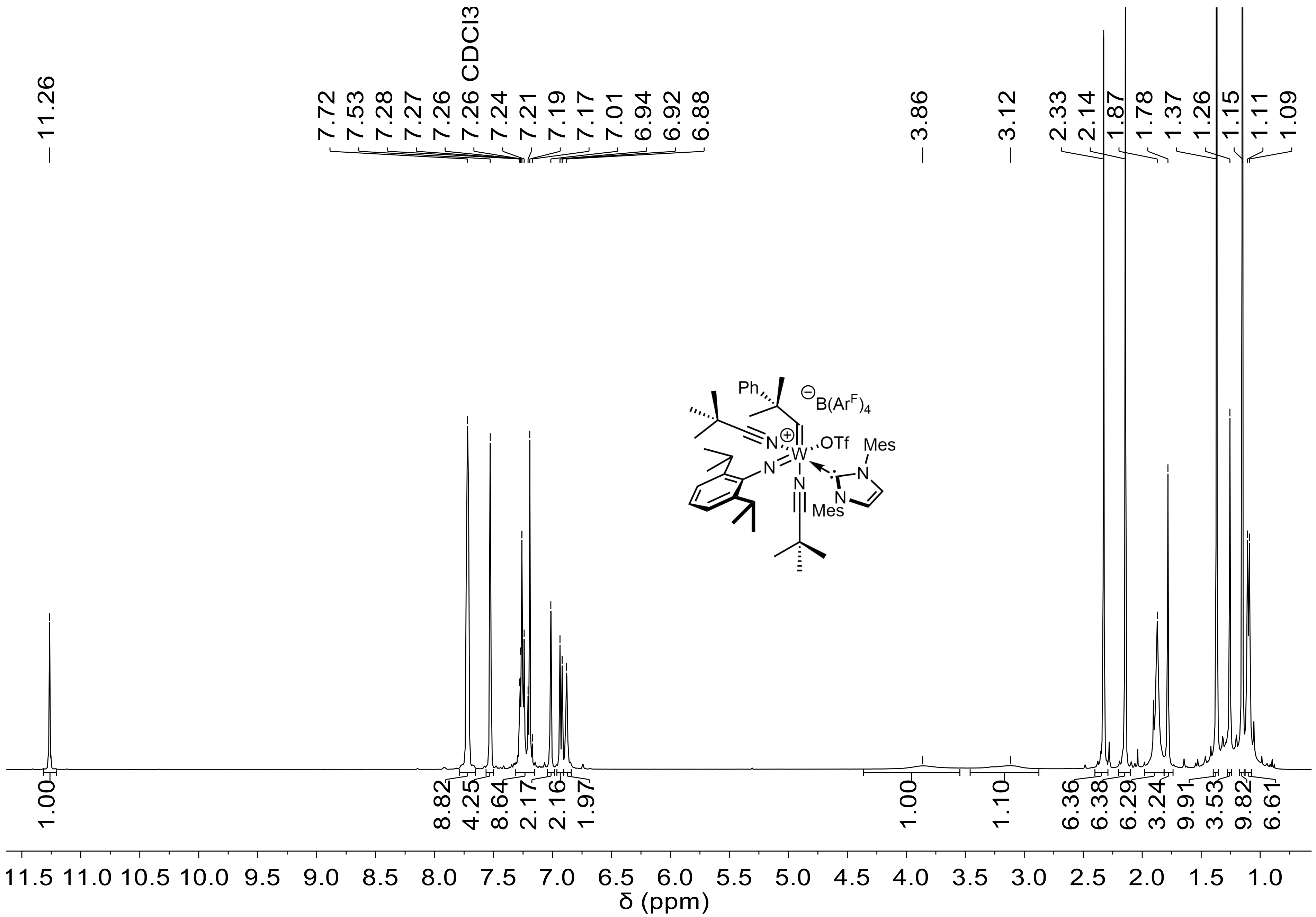

Figure S102: ^{1}H-NMR (400 MHz, 25 °C, $CDCl_3$) of W-36.

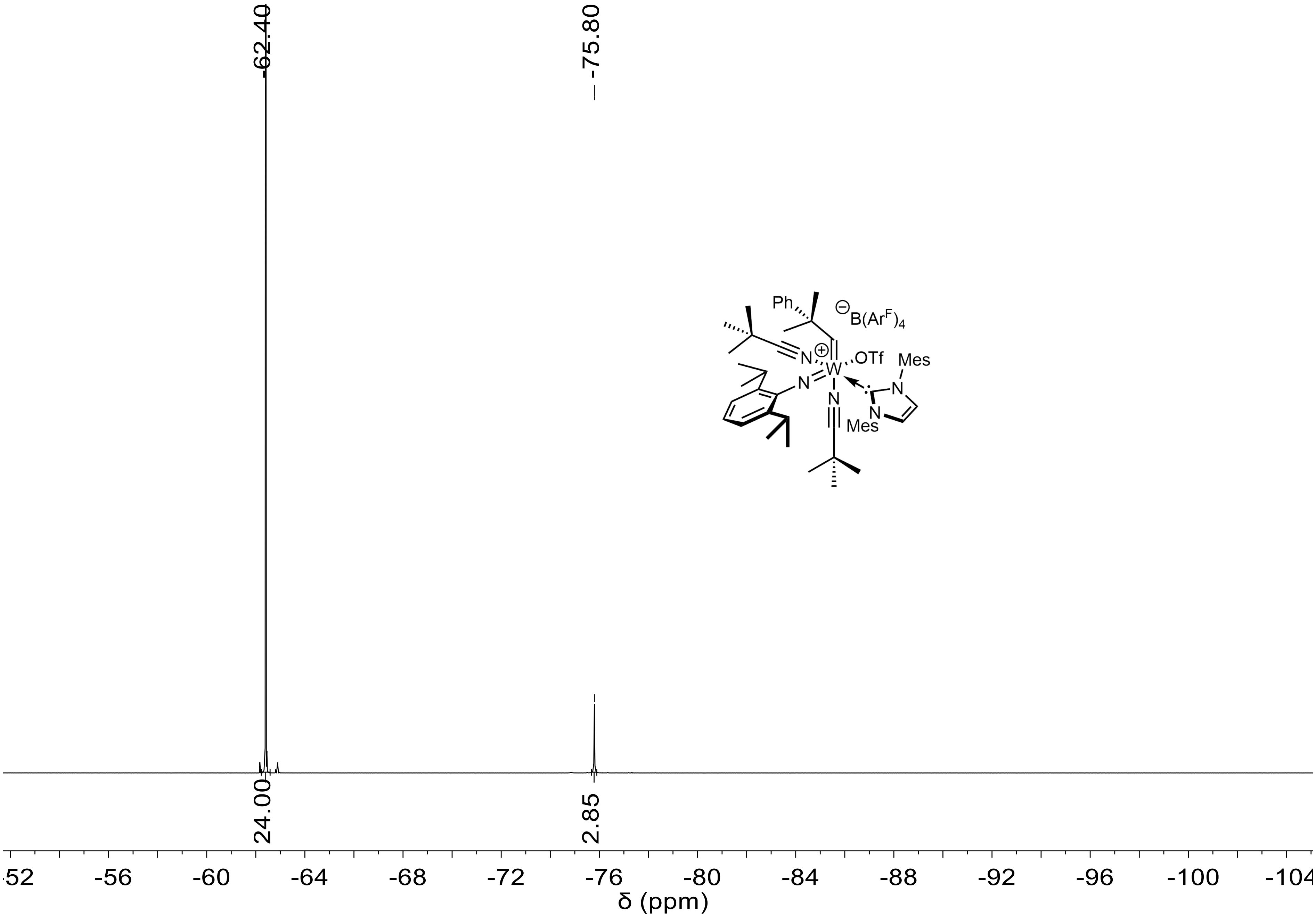

Figure S103: ^{19}F-NMR (376 MHz, 25 °C, $CDCl_3$) of W-36.

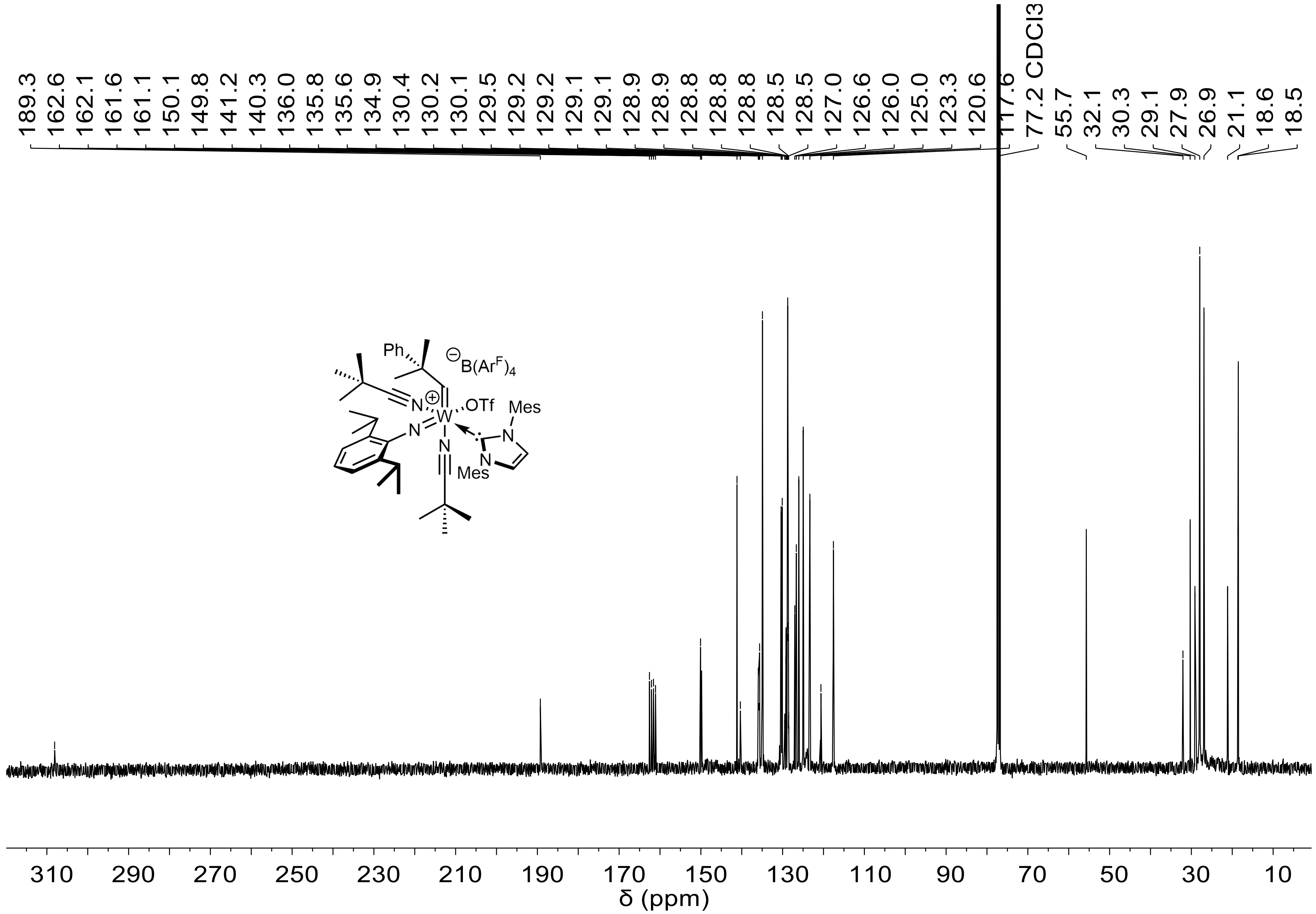

Figure S104: ^{13}C-NMR (101 MHz, 25 °C, $CDCl_3$) of W-36.

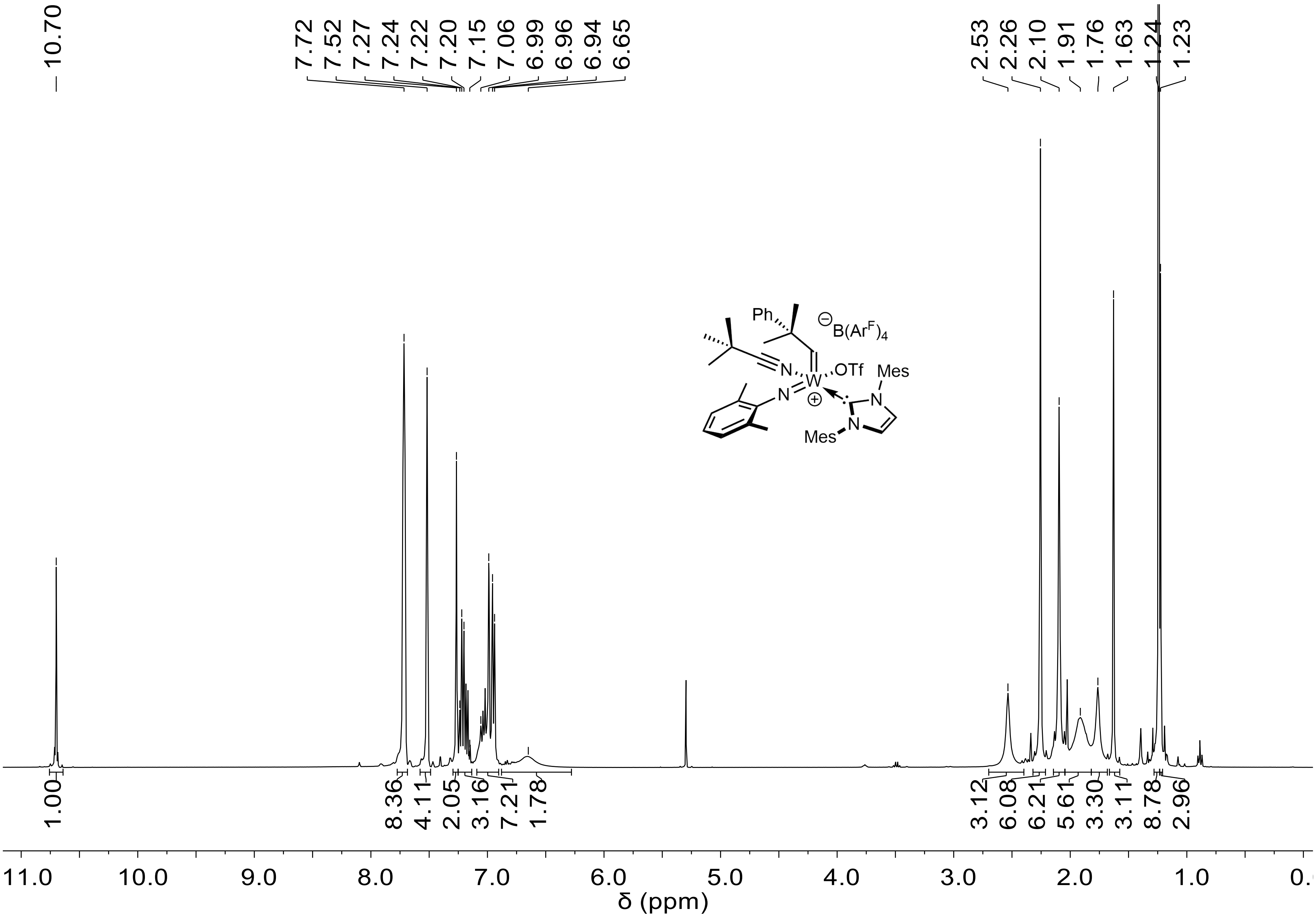

Figure S105: ^{1}H-NMR (400 MHz, 25 °C, $CDCl_3$) of W-37.

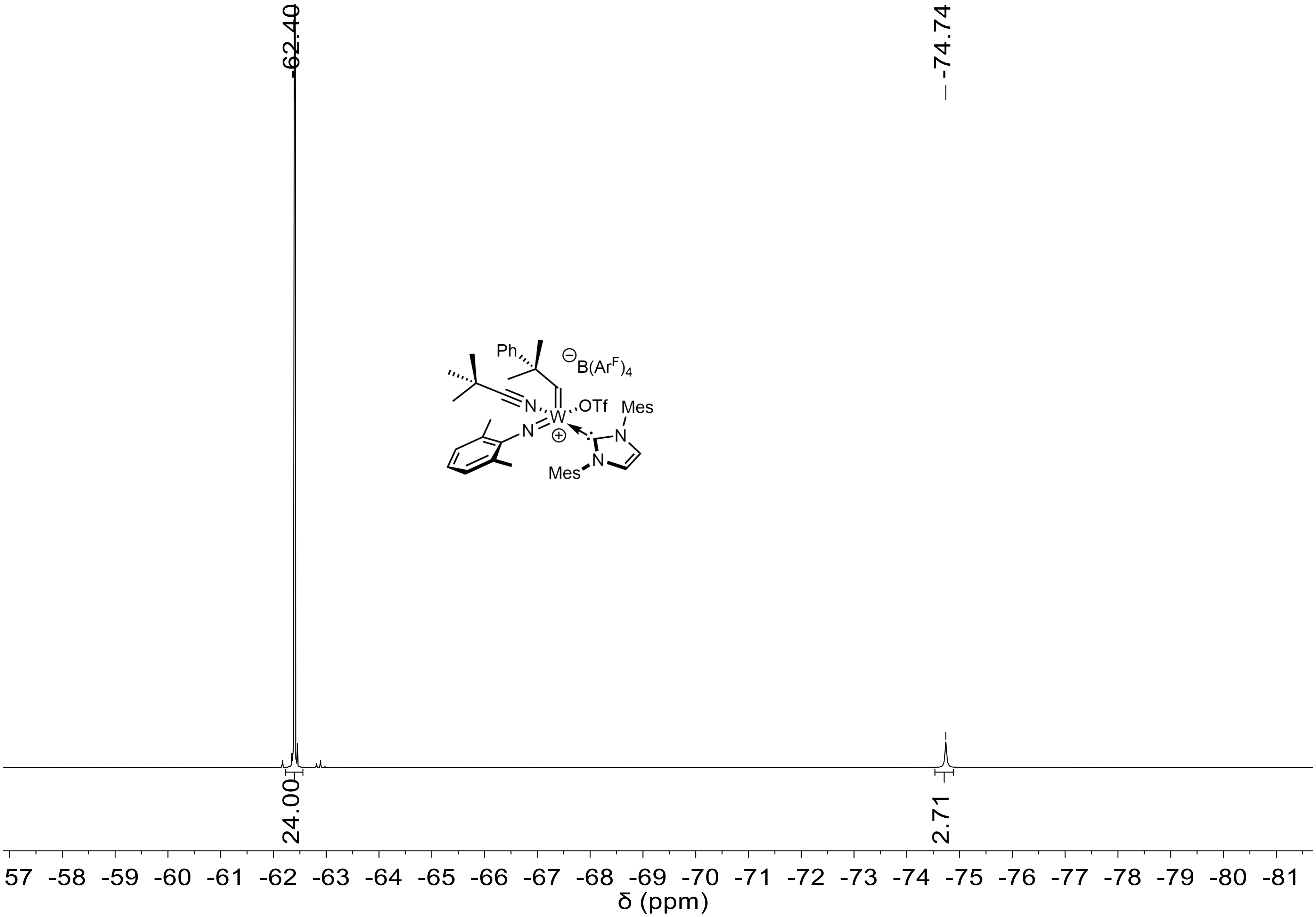

Figure S106: ^{19}F-NMR (376 MHz, 25 °C, $CDCl_3$) of W-37.

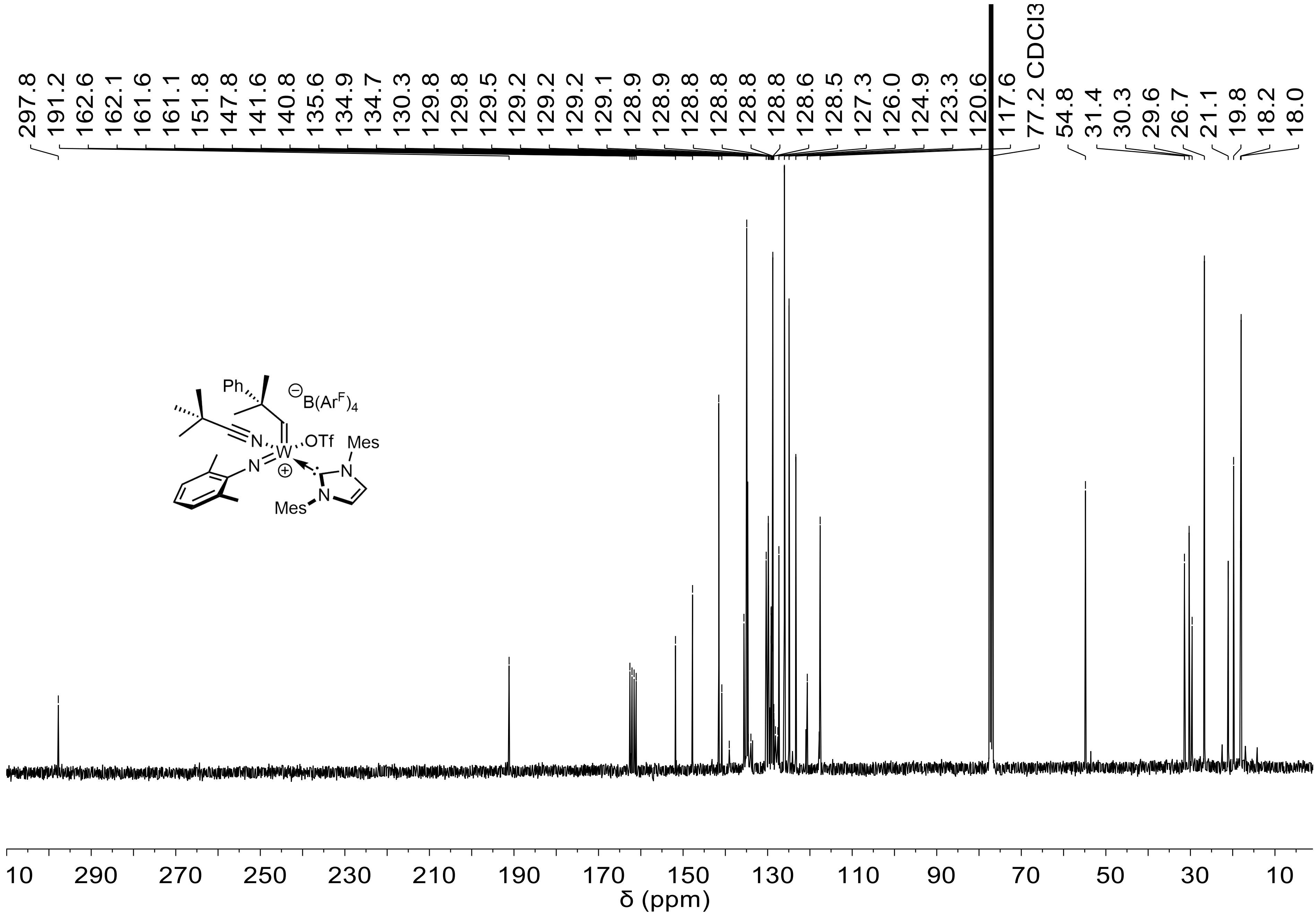

Figure S107: ^{13}C-NMR (101 MHz, 25 °C, $CDCl_3$) of W-37.

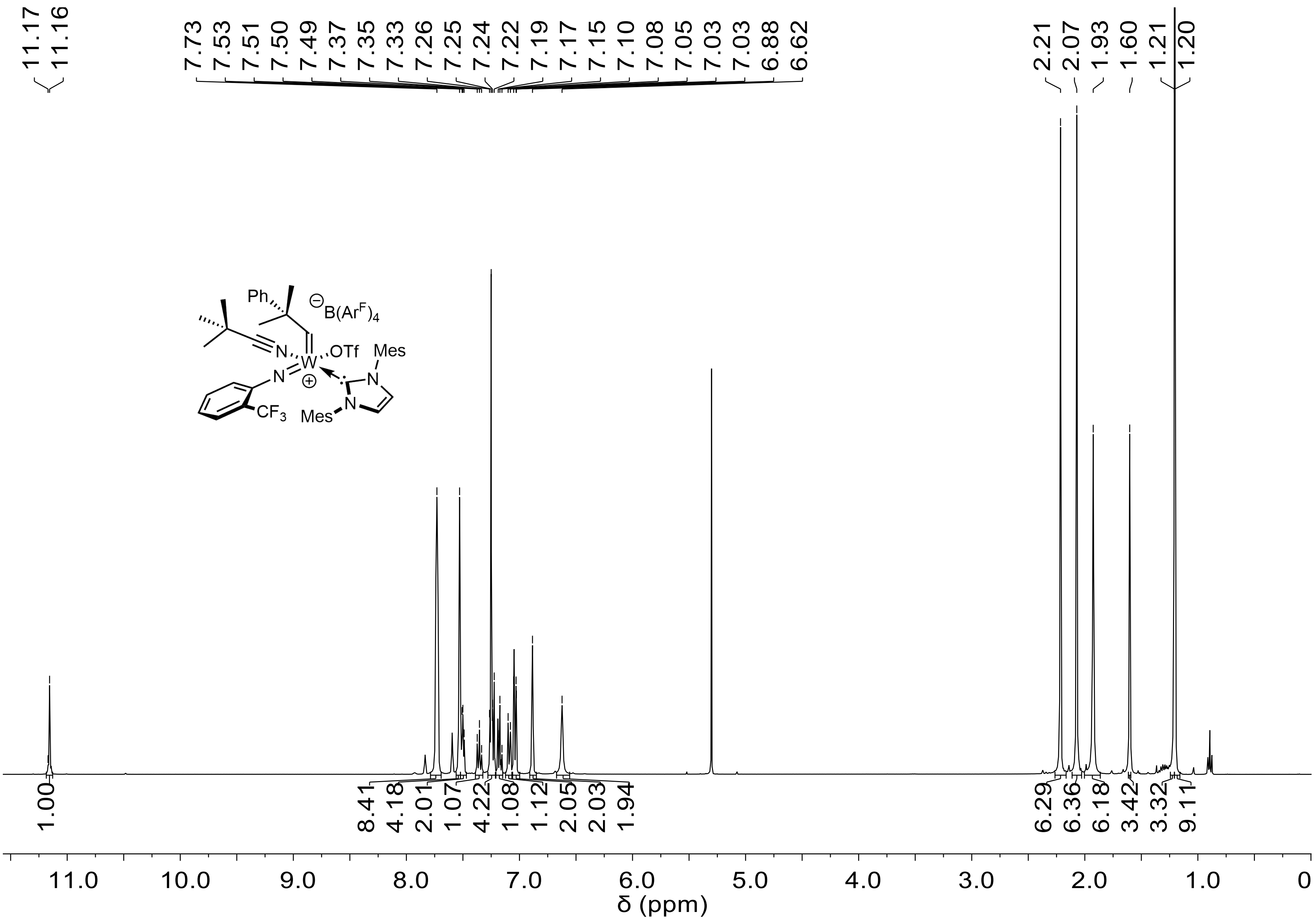

Figure S108: ^{1}H-NMR (400 MHz, 25 °C, $CDCl_3$) of W-38.

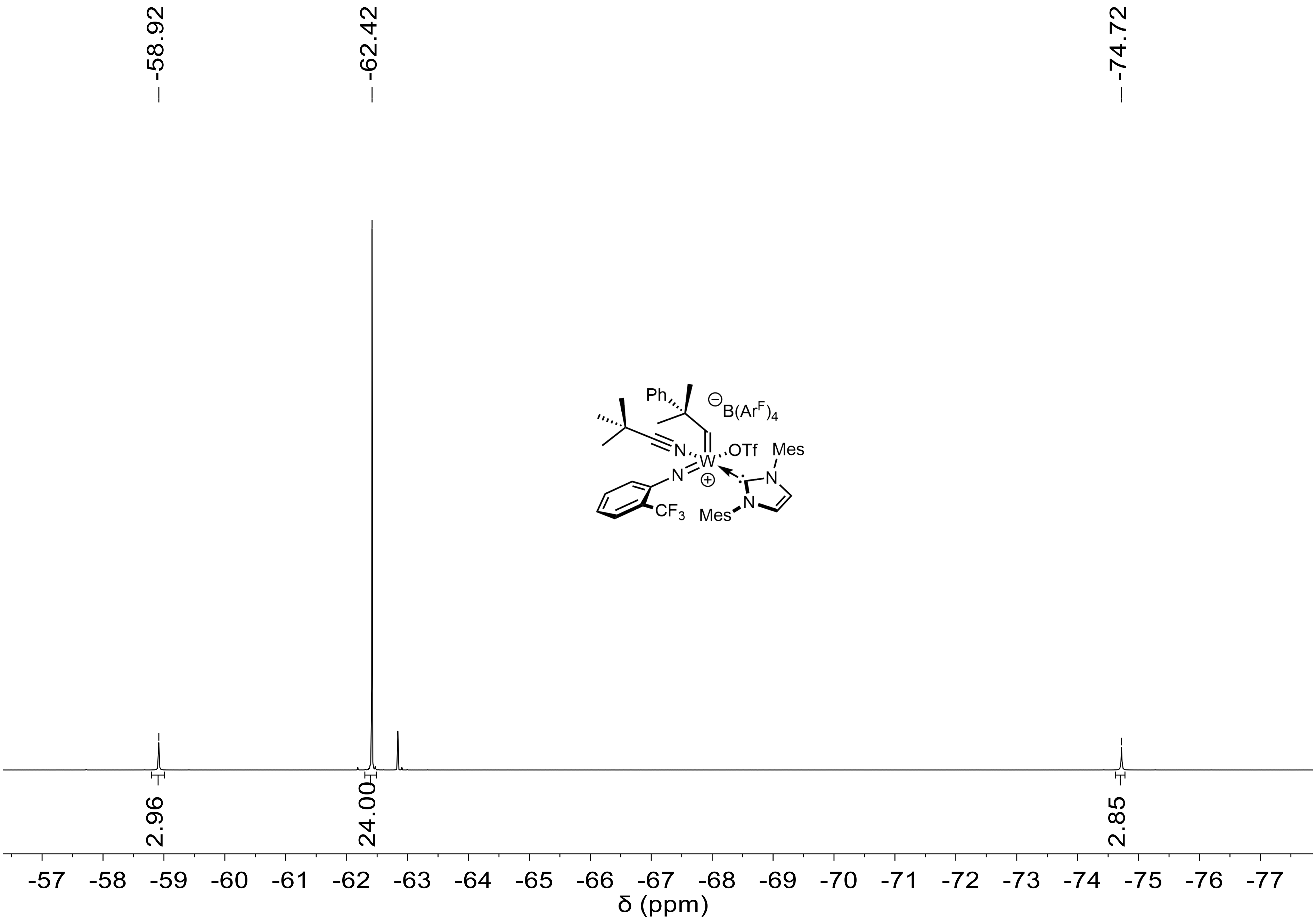

Figure S109: ^{19}F-NMR (376 MHz, 25 °C, $CDCl_3$) of W-38.

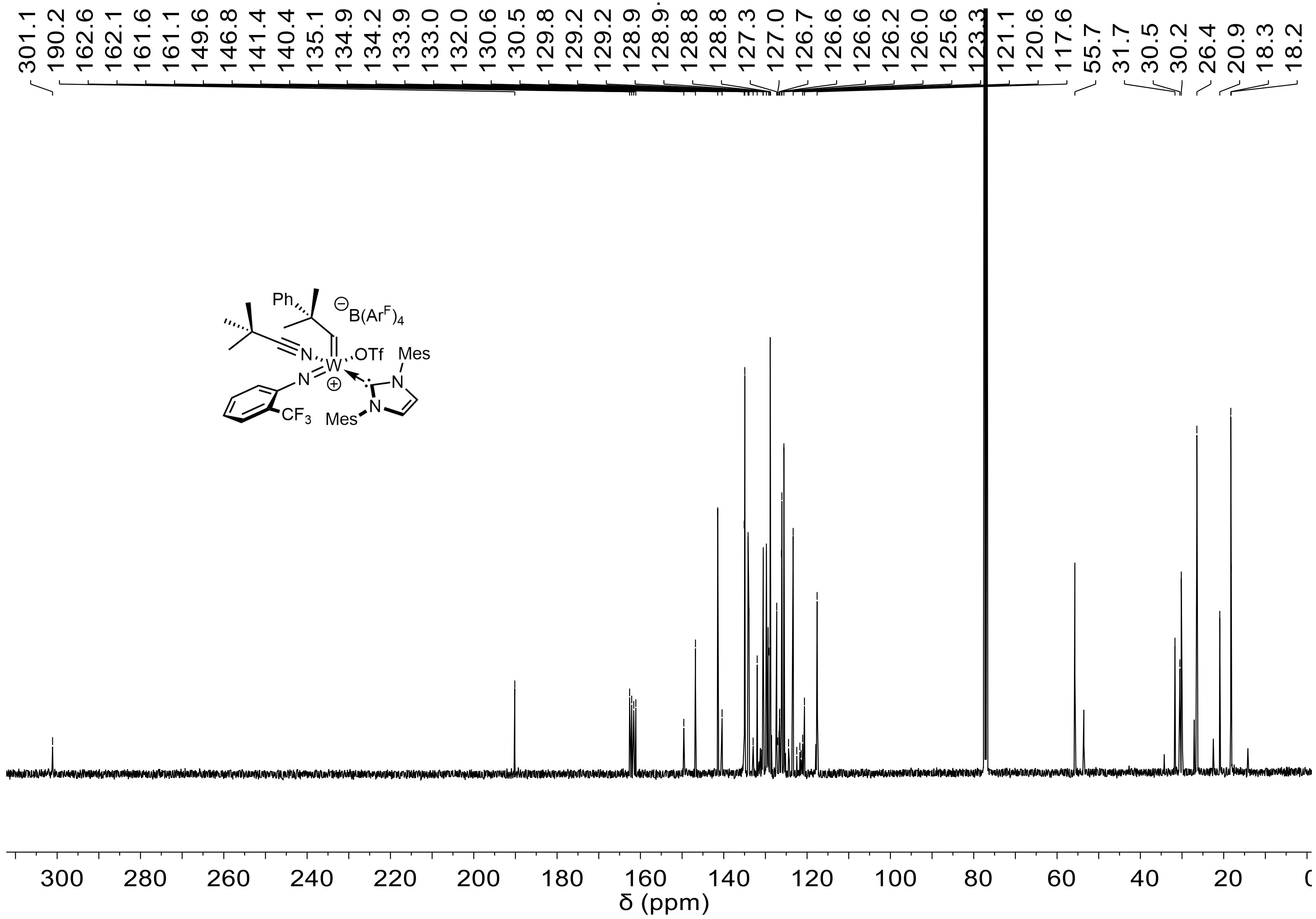

Figure S110: ^{13}C-NMR (101 MHz, 25 °C, $CDCl_3$) of W-38

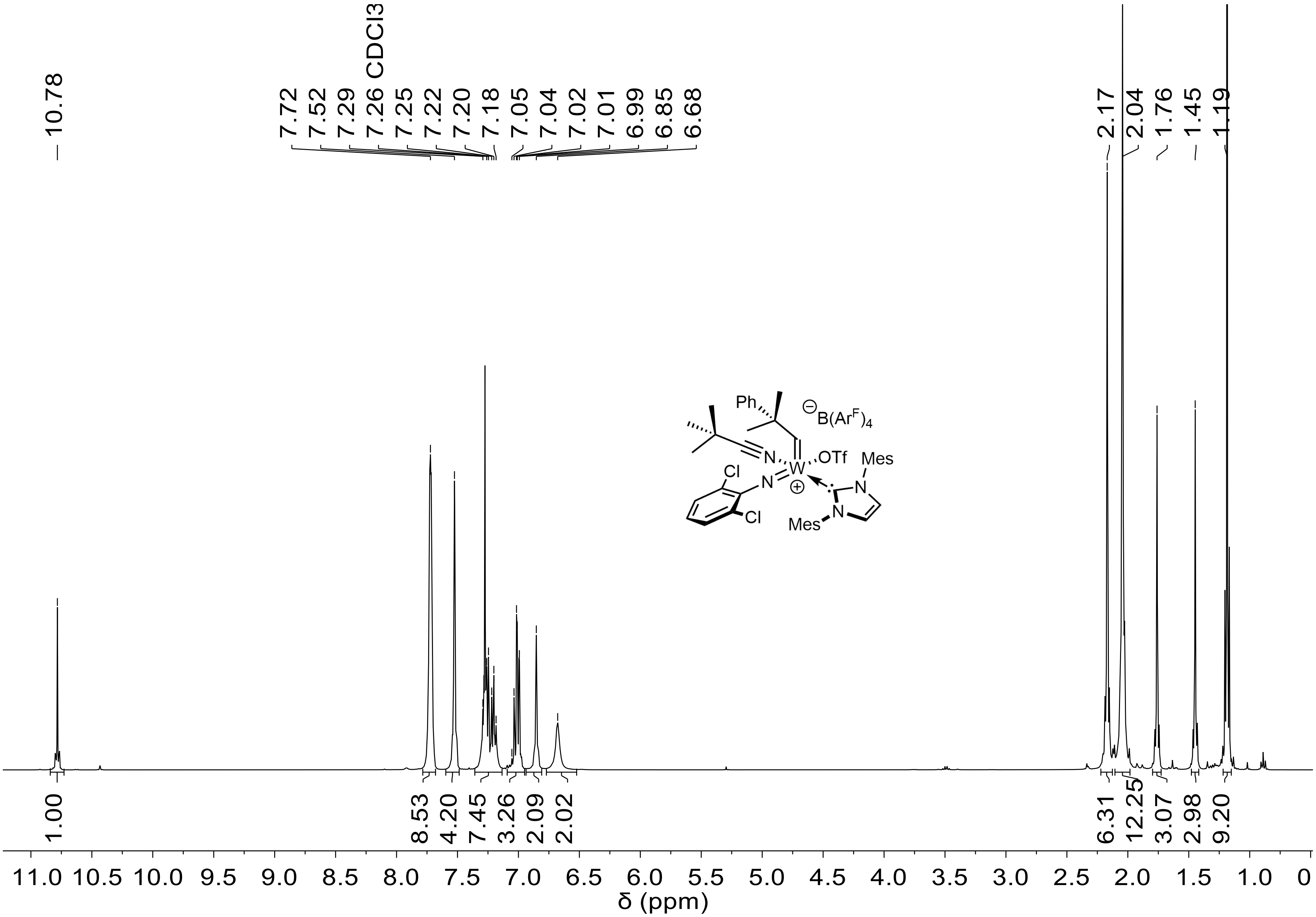

Figure S111: ^{1}H-NMR (400 MHz, 25 °C, $CDCl_3$) of W-39.

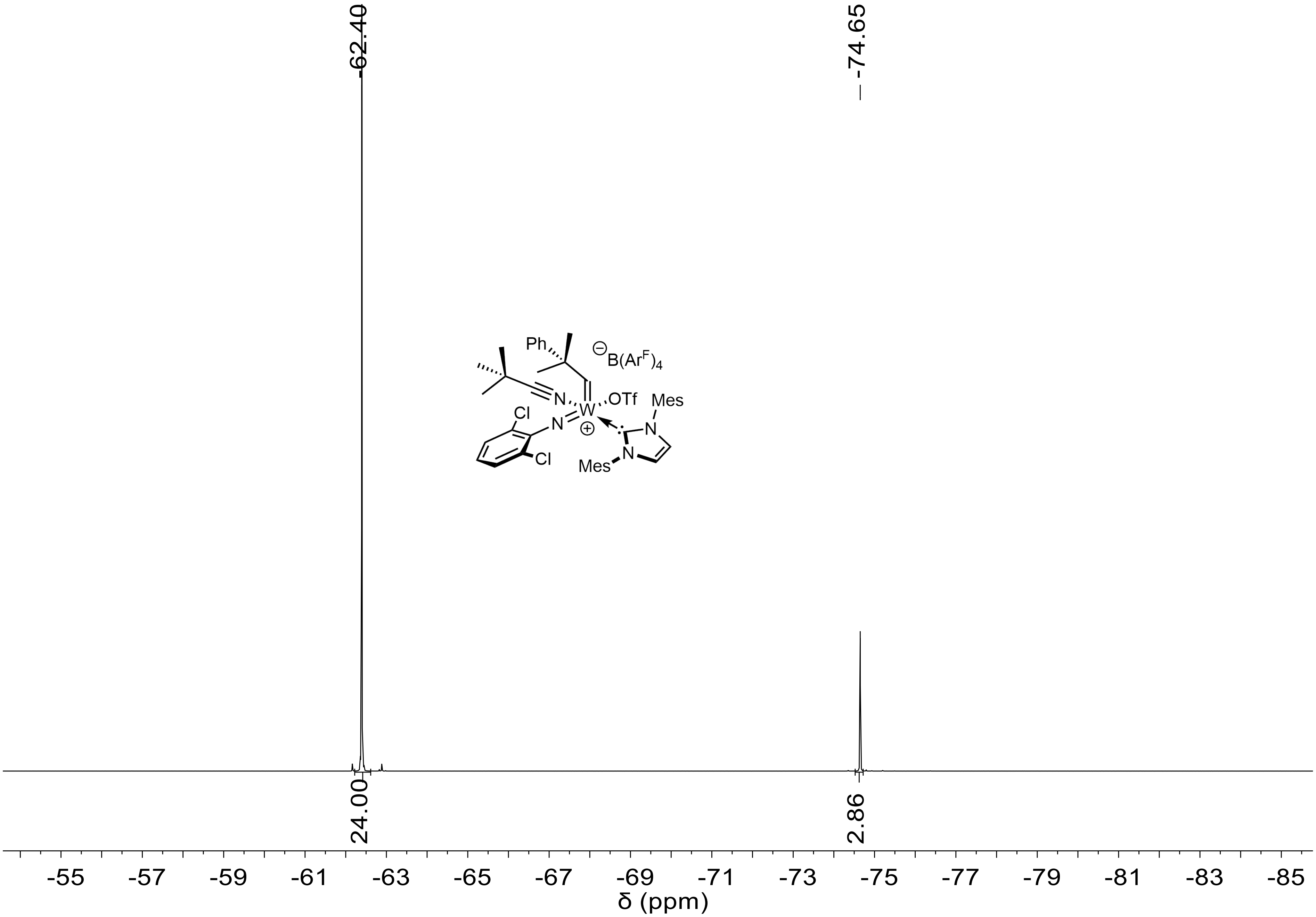

Figure S112: ^{19}F-NMR (376 MHz, 25 °C, $CDCl_3$) of W-39.

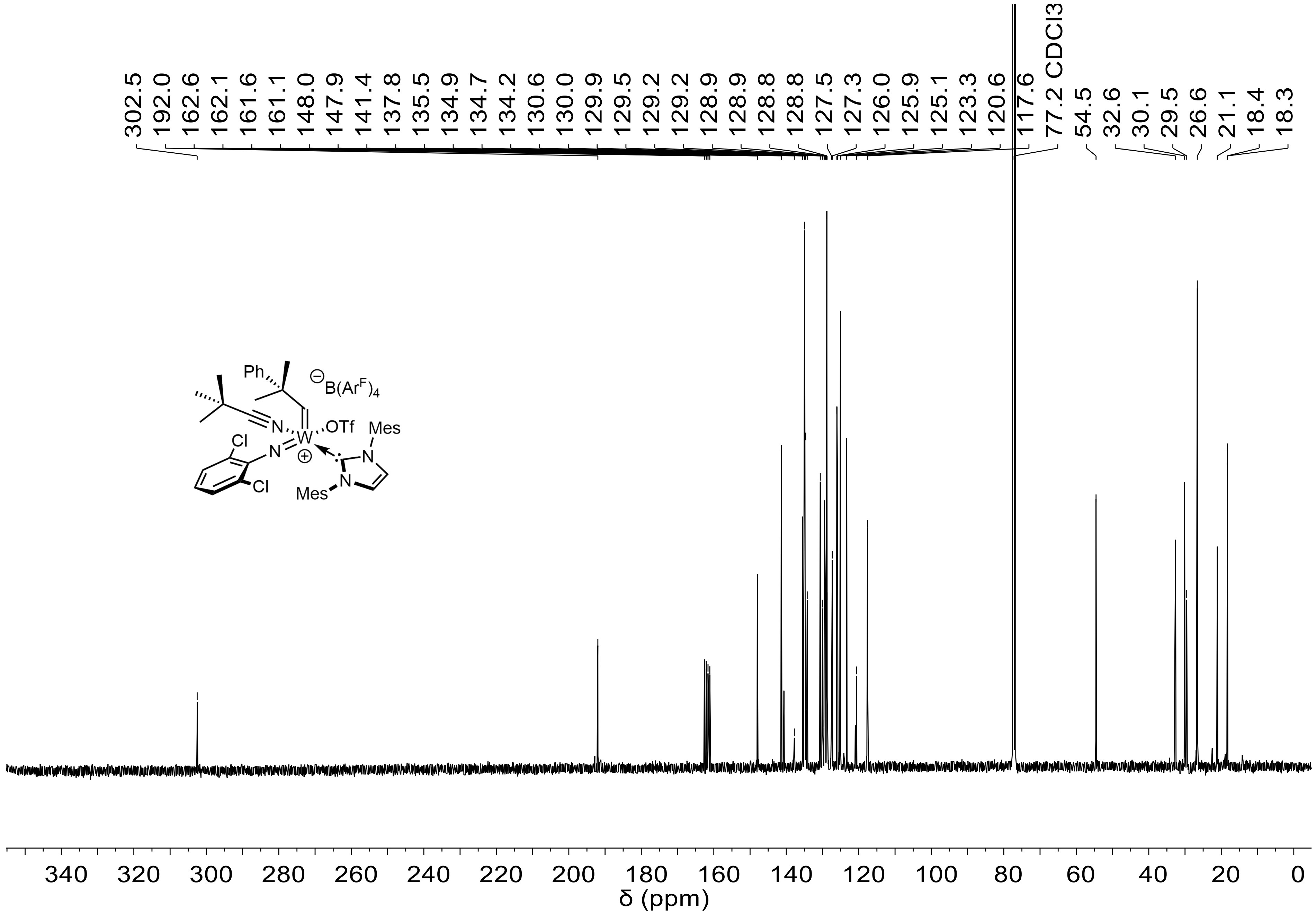

Figure S113: ^{13}C-NMR (101 MHz, 25 °C, $CDCl_3$) of W-39.

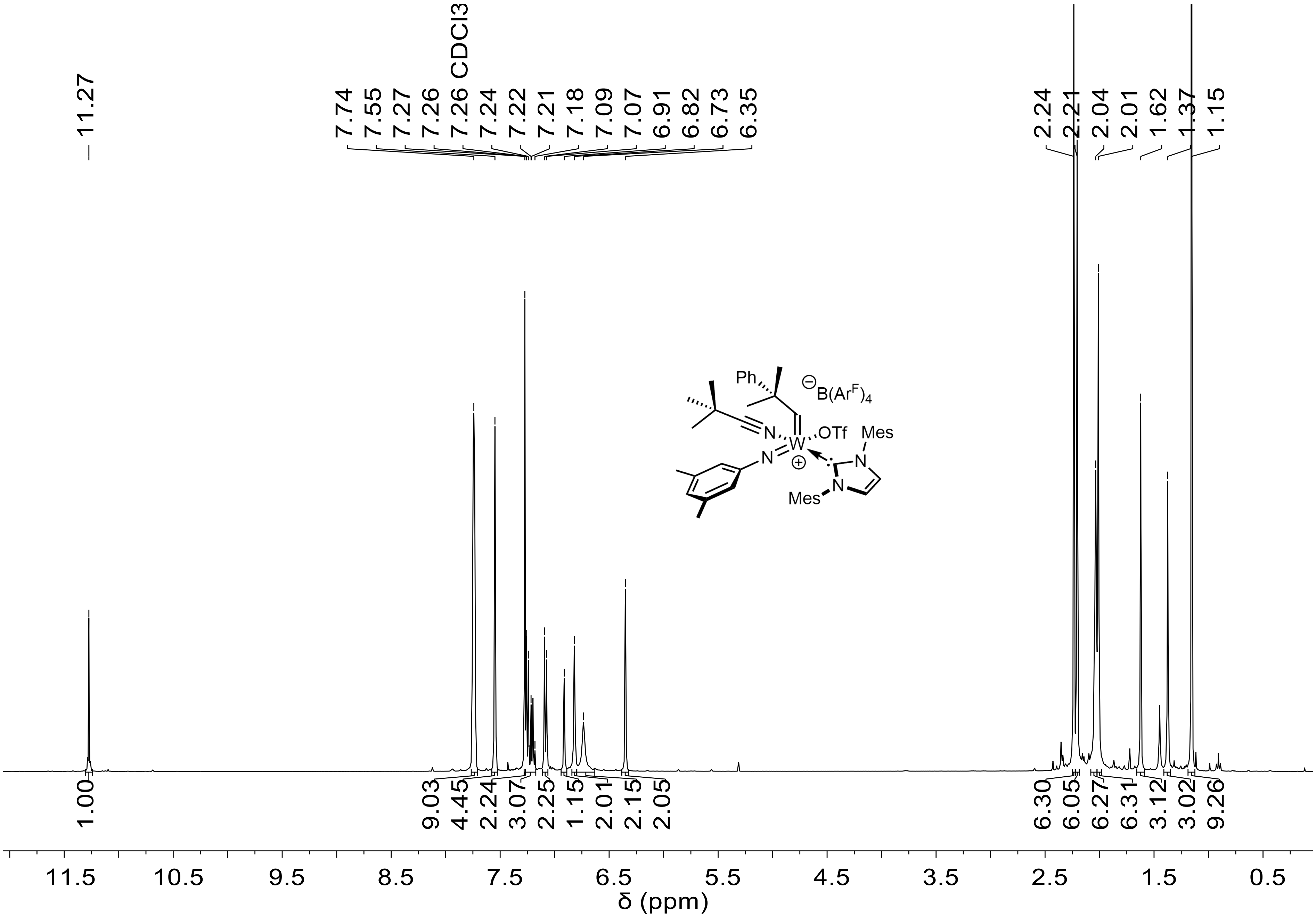

Figure S114: ^{1}H-NMR (400 MHz, 25 °C, CDCl$_3$) of W-40.

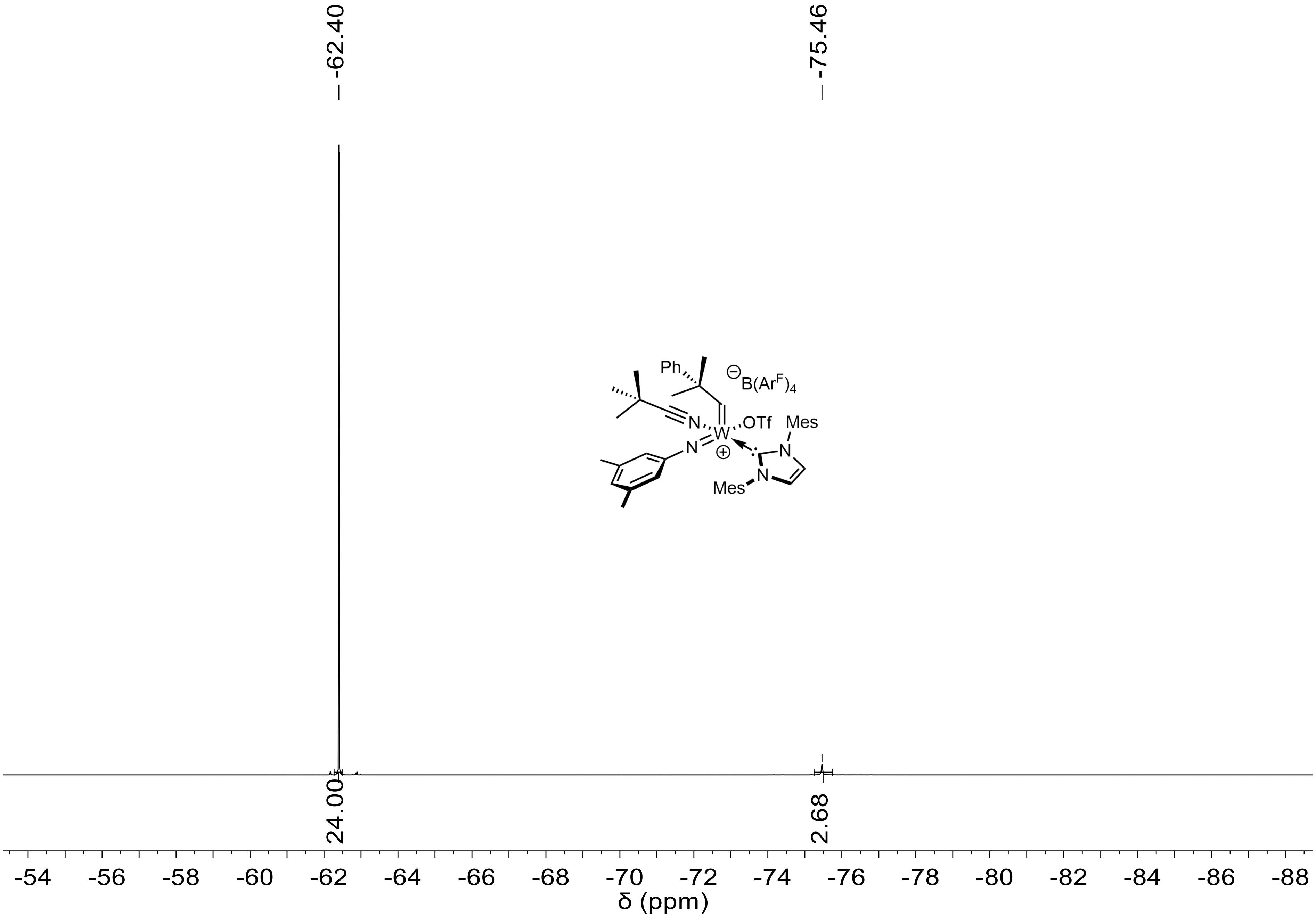

Figure S115: ^{19}F-NMR (376 MHz, 25 °C, $CDCl_3$) of W-40.

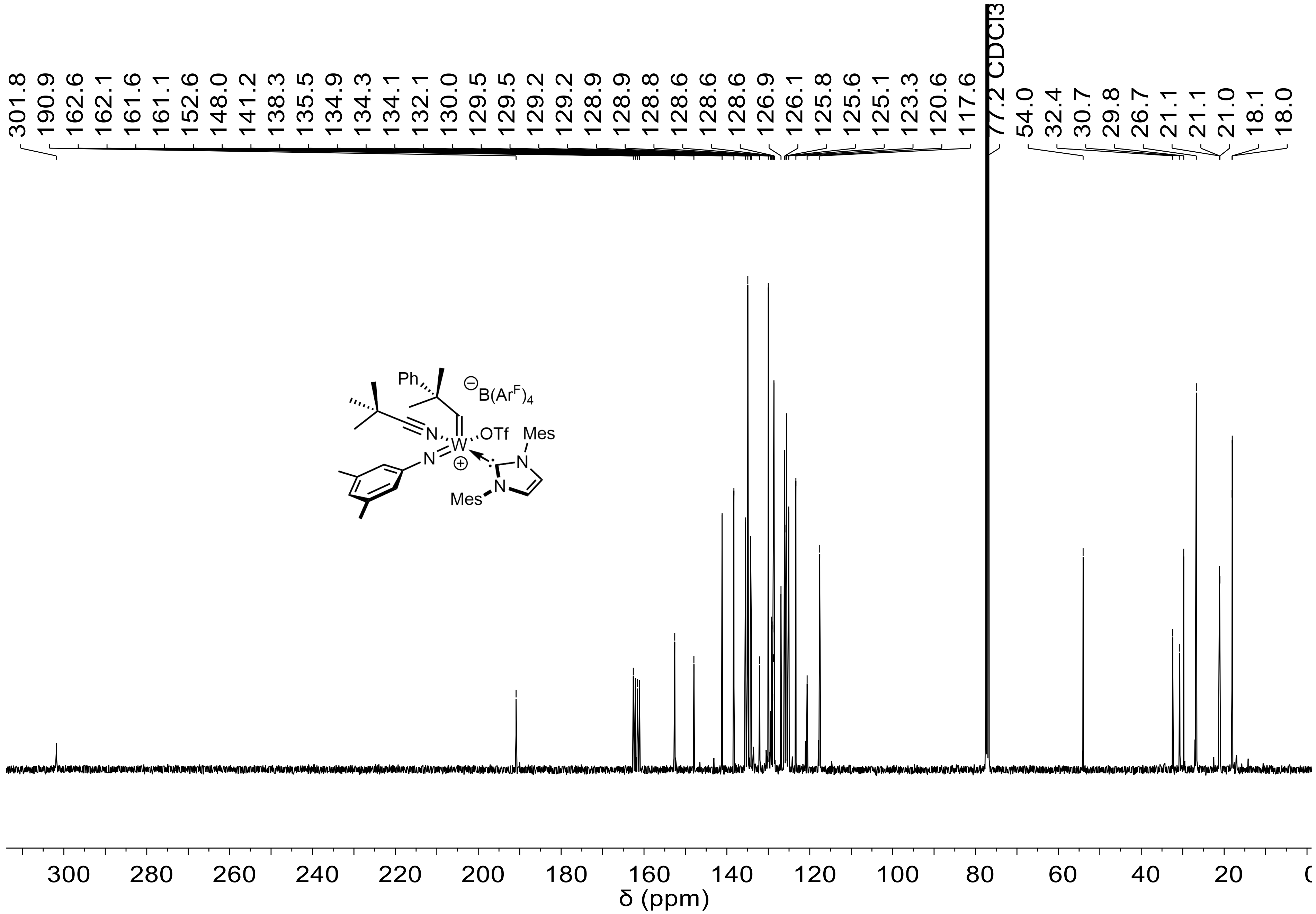

Figure S116: ^{13}C-NMR (101 MHz, 25 °C, CDCl$_3$) of W-40.

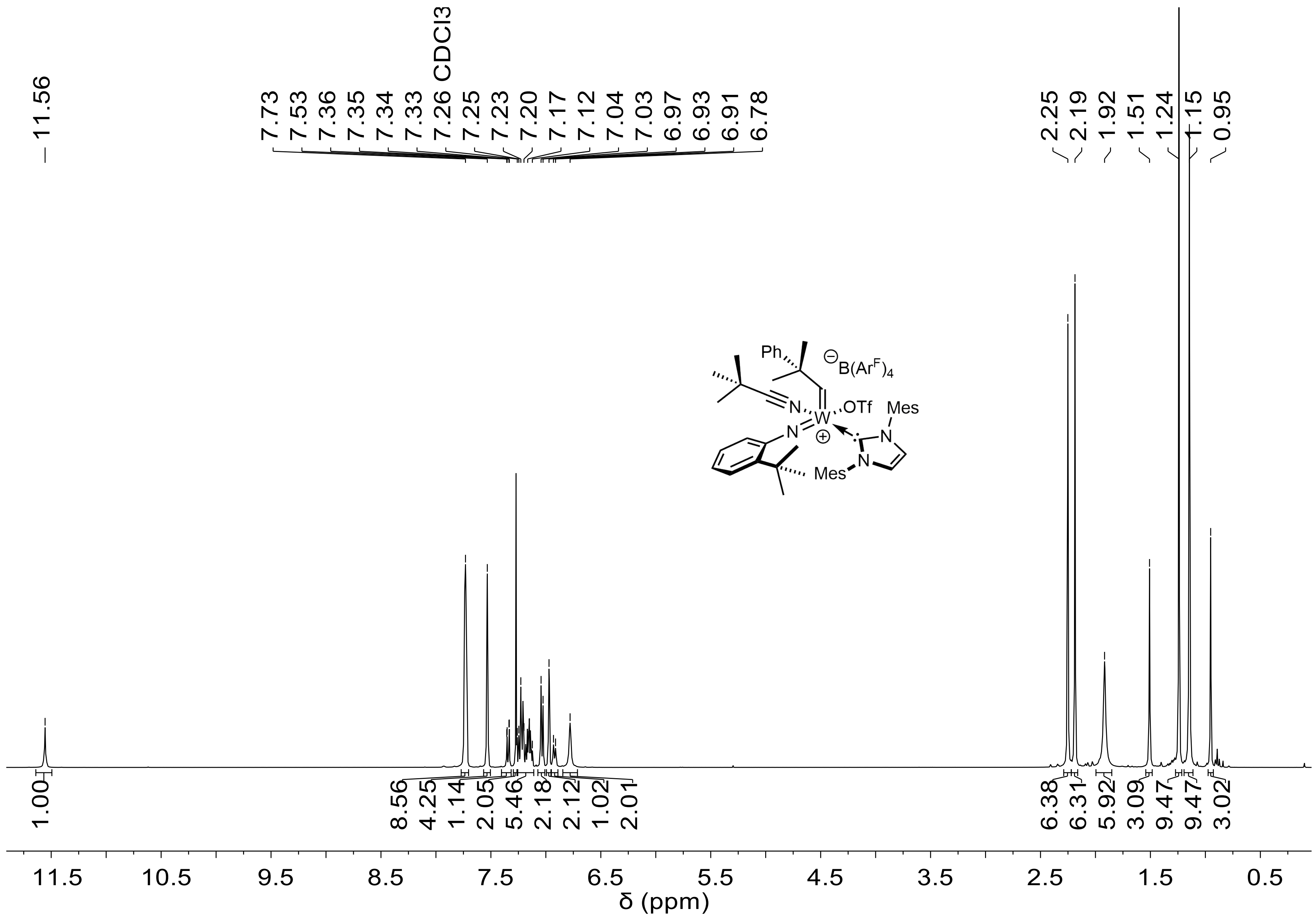

Figure S117: ¹H-NMR (400 MHz, 25 °C, CDCl$_3$) of W-41.

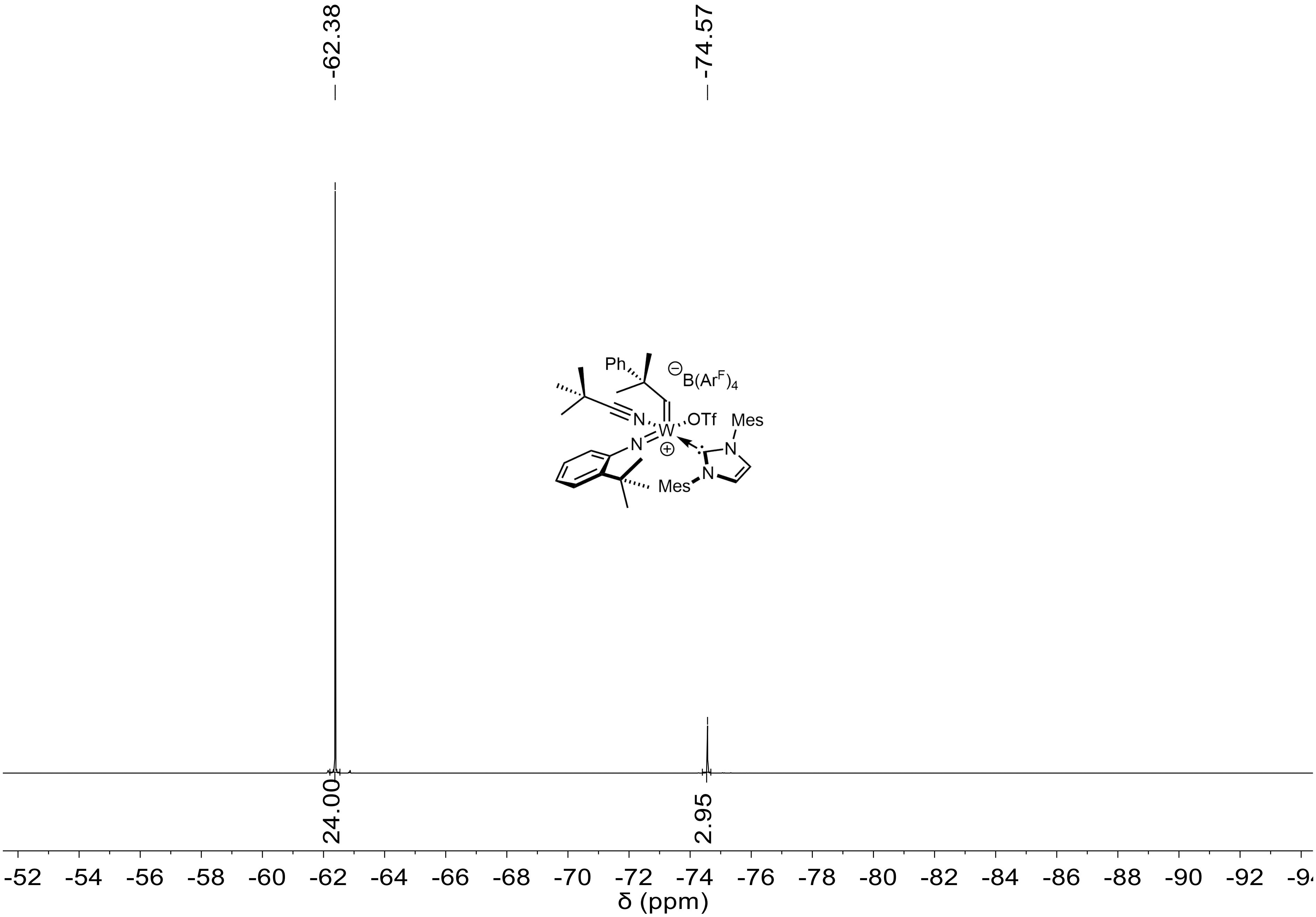

Figure S118: ^{19}F-NMR (376 MHz, 25 °C, $CDCl_3$) of W-41.

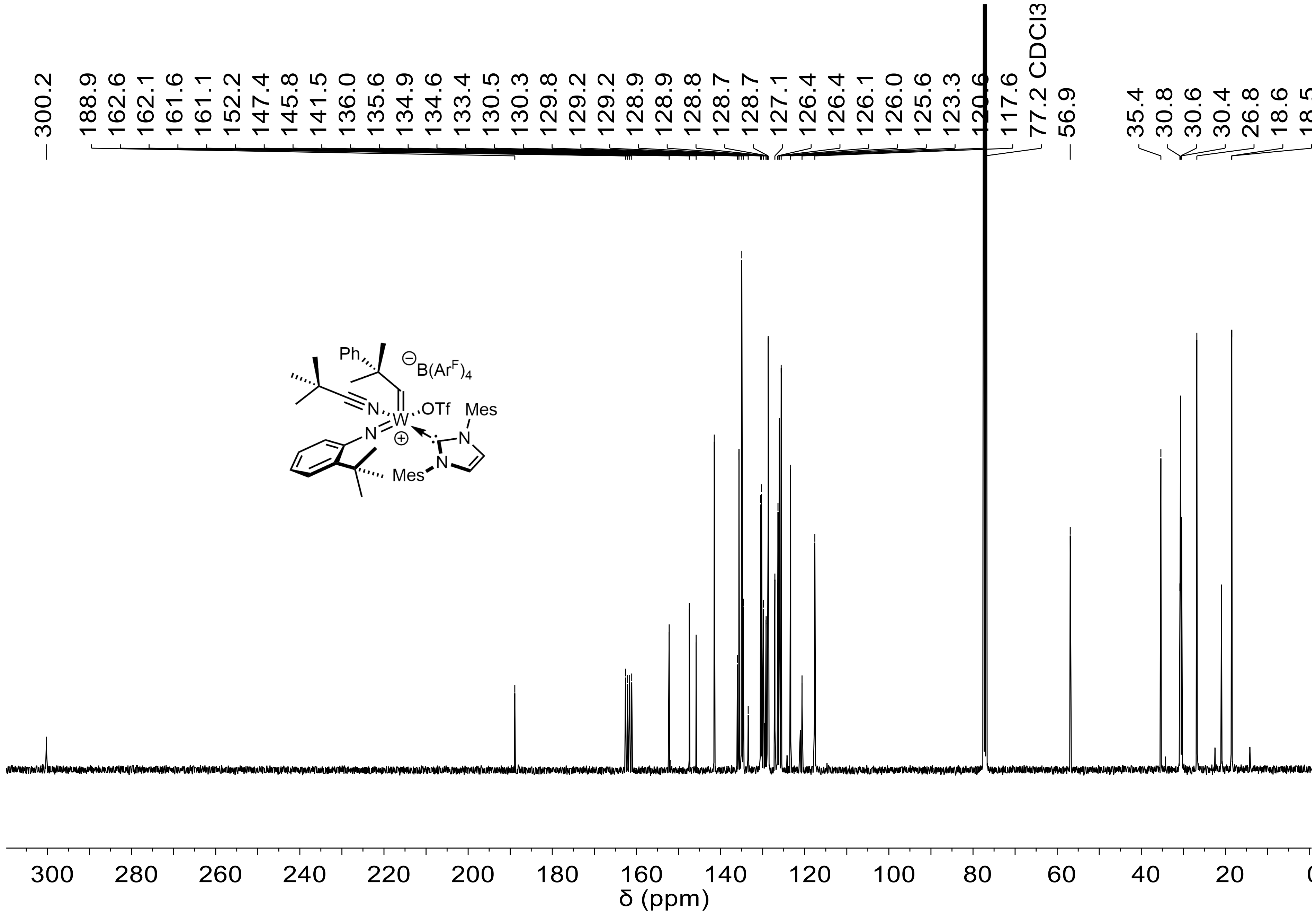

Figure S119: ^{13}C-NMR (101 MHz, 25 °C, CDCl$_3$) of W-41.

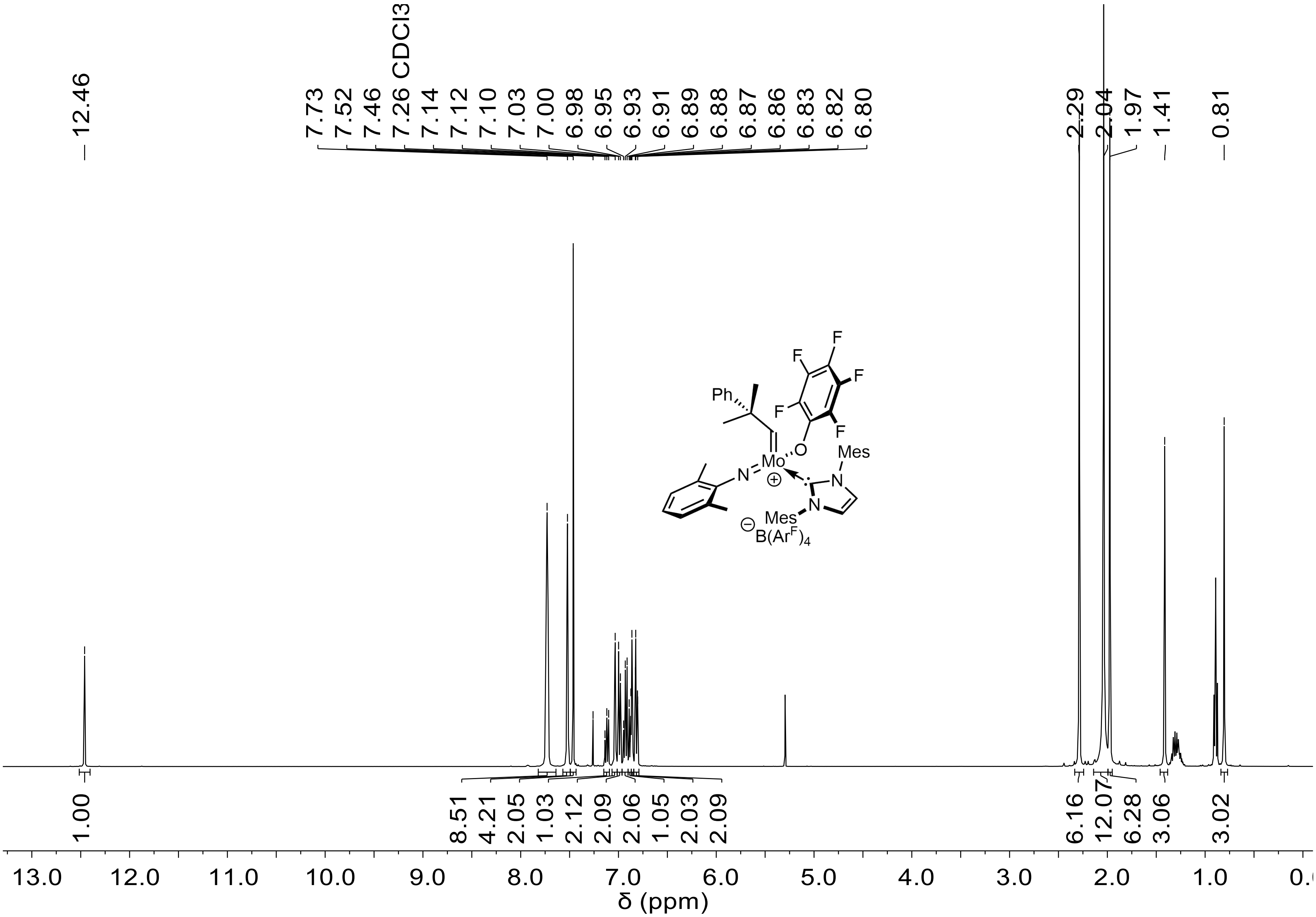

Figure S120: ^{1}H-NMR (400 MHz, 25 °C, $CDCl_3$) of Mo-02.

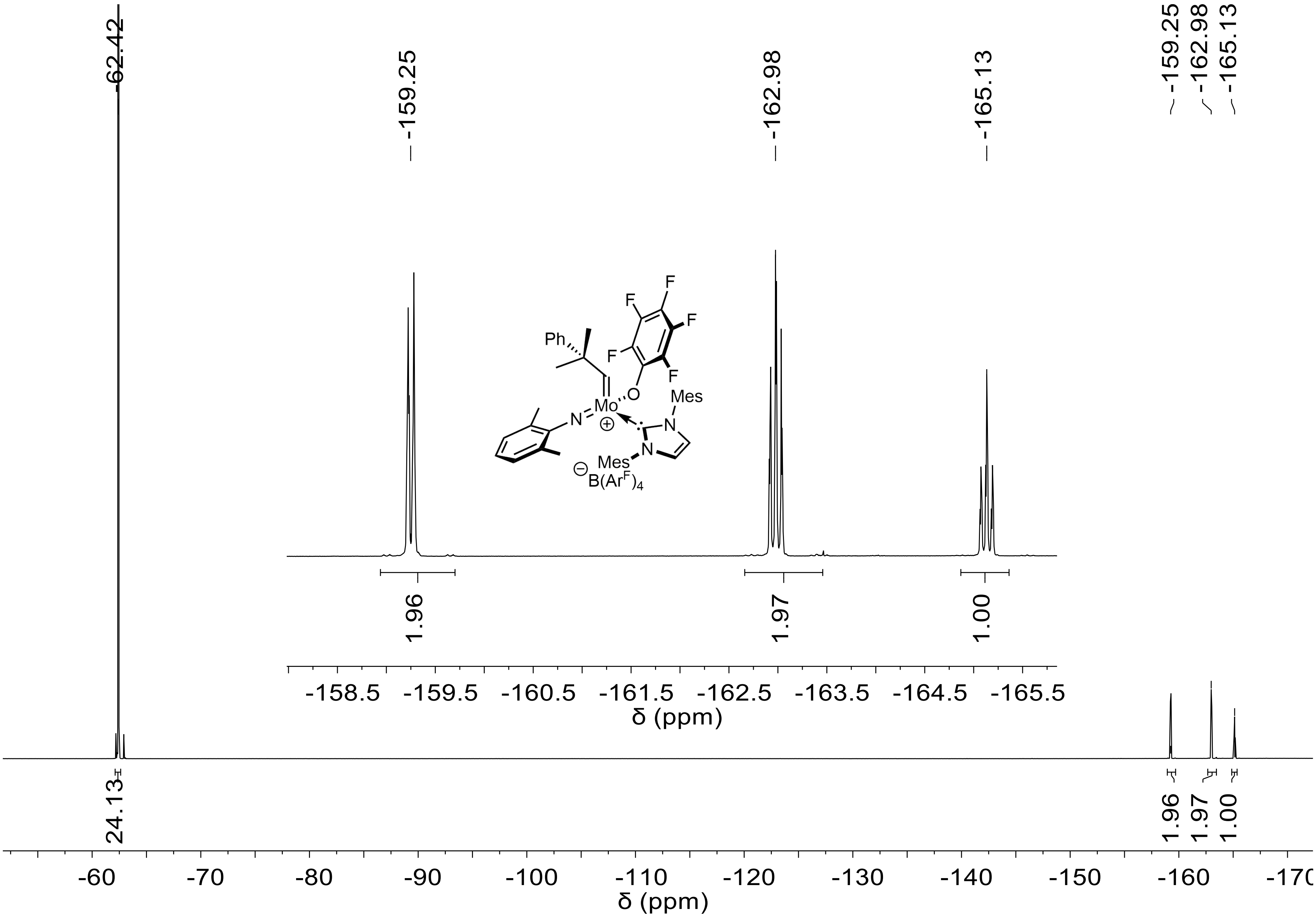

Figure S121: ^{19}F-NMR (376 MHz, 25 °C, $CDCl_3$) of Mo-02.

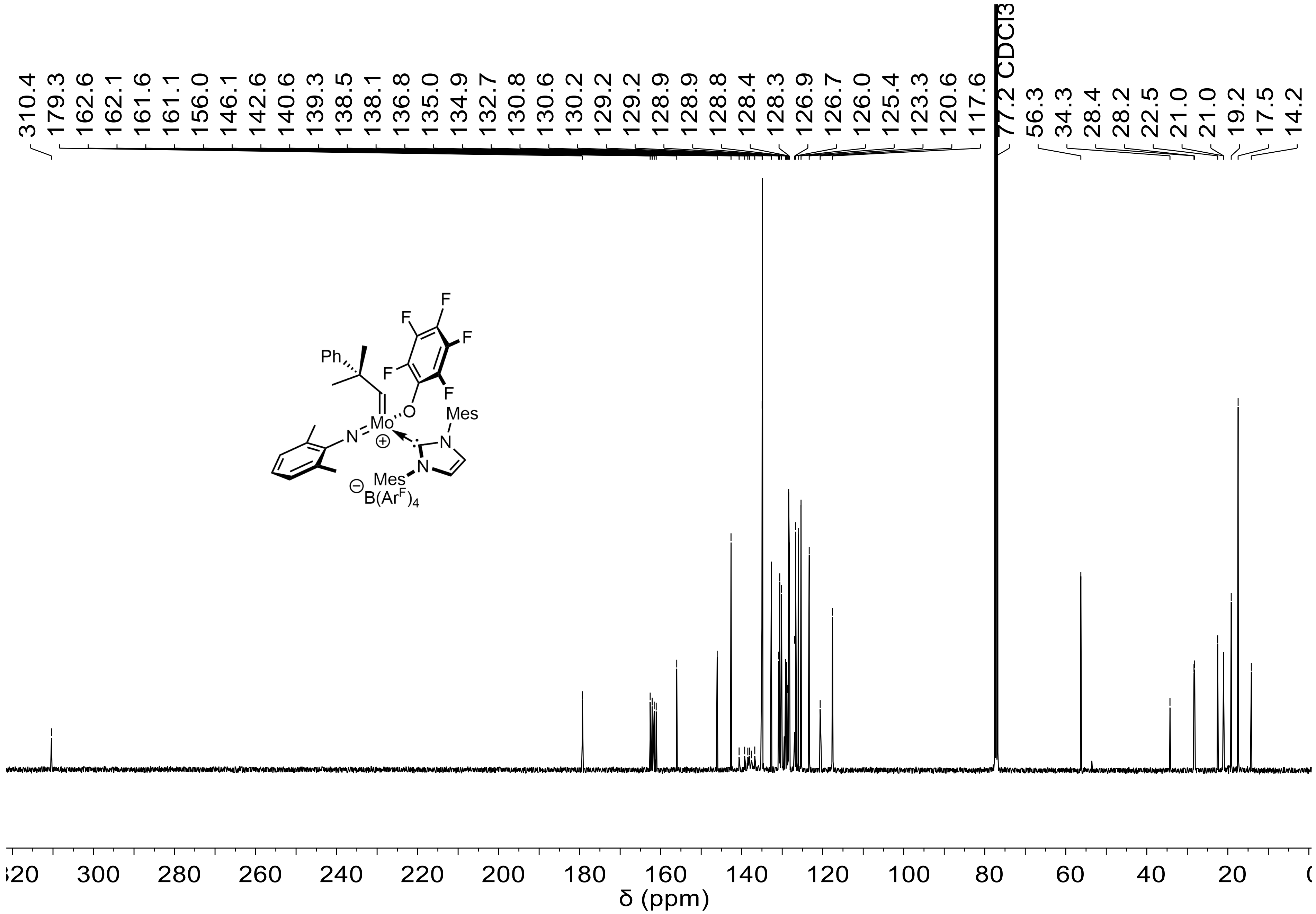

Figure S122: ^{13}C-NMR (101 MHz, 25 °C, $CDCl_3$) of Mo-02.

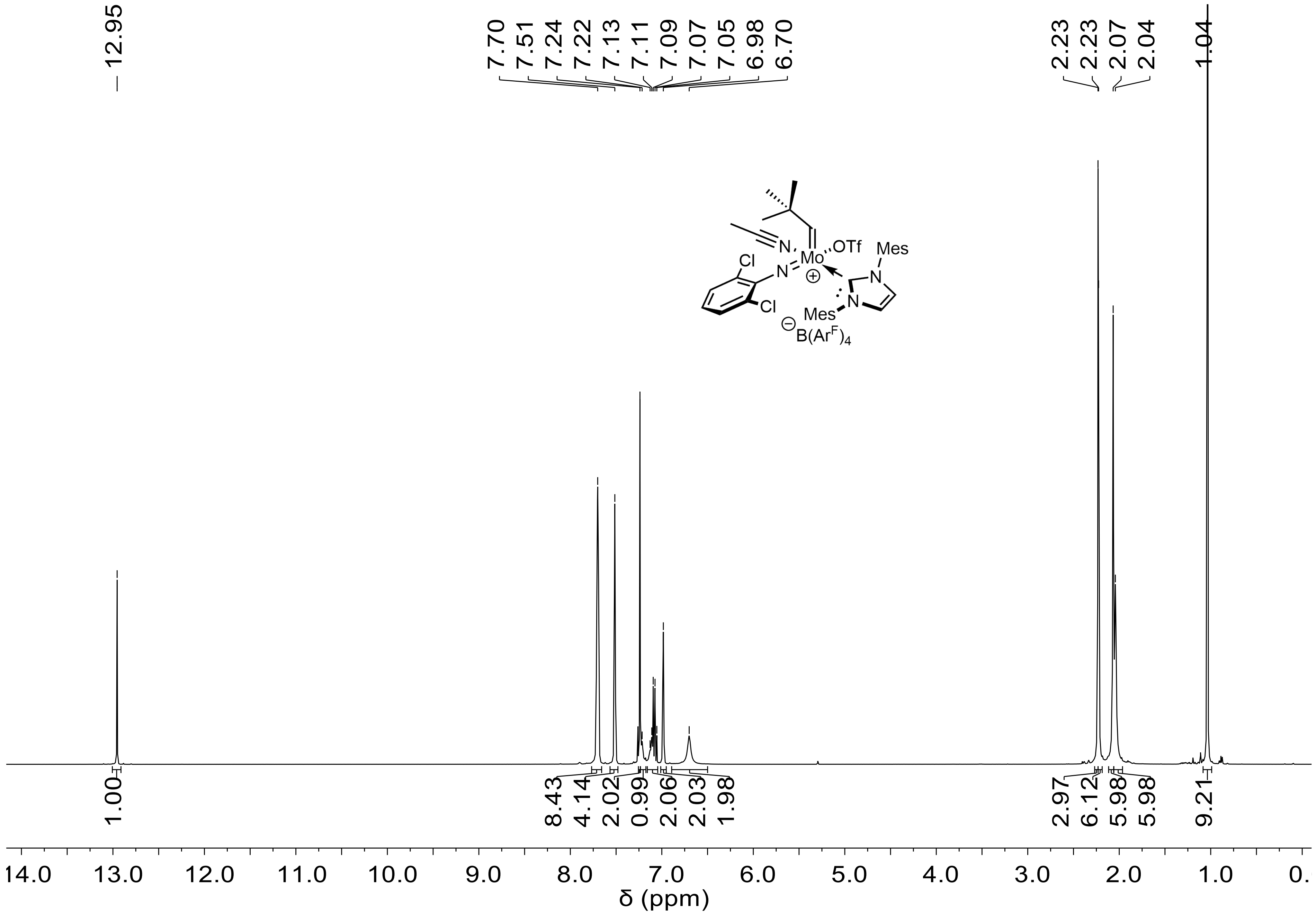

Figure S123: ^{1}H-NMR (400 MHz, 25 °C, $CDCl_3$) of Mo-03-MeCN.

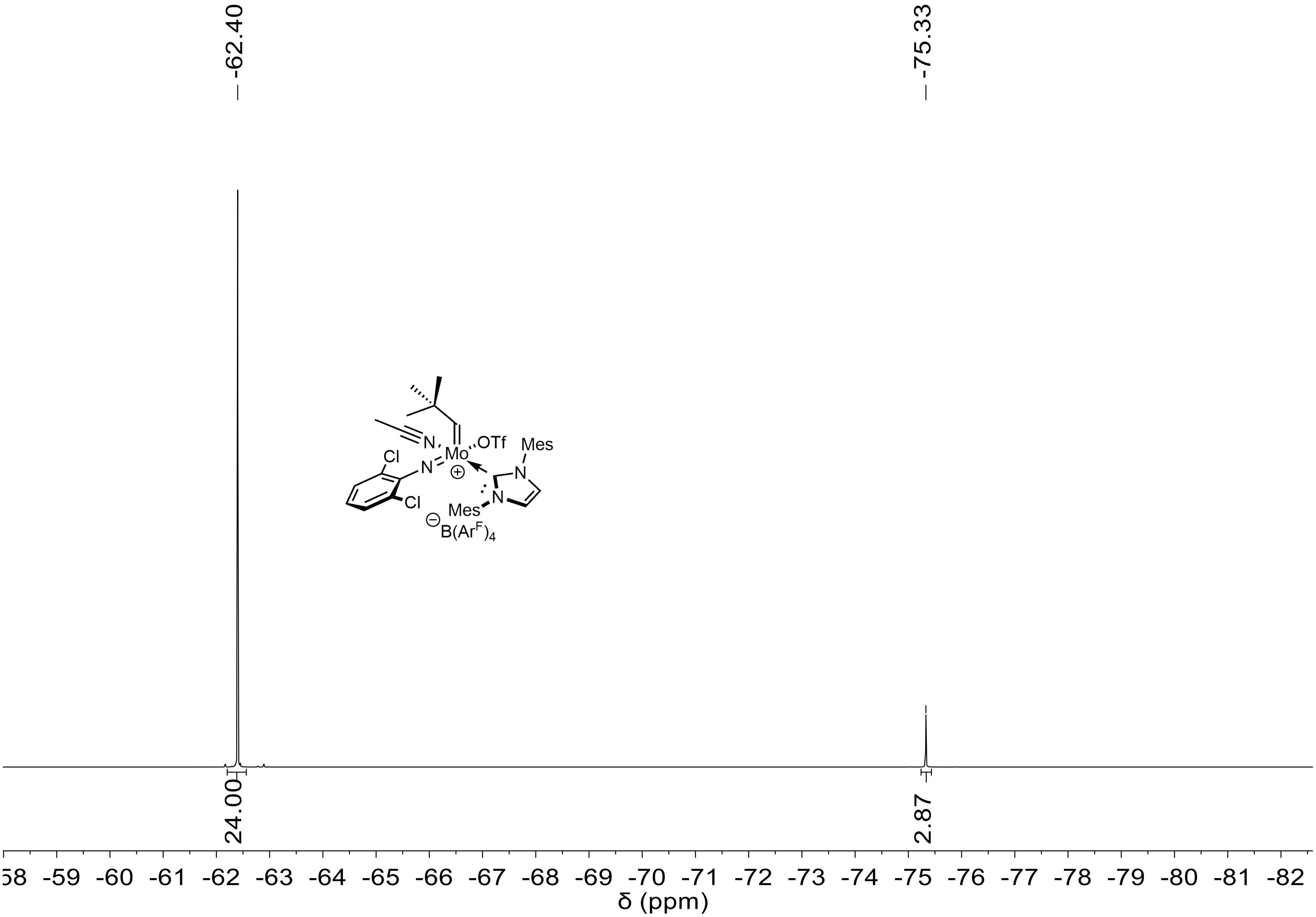

Figure S124: ^{19}F-NMR (376 MHz, 25 °C, $CDCl_3$) of Mo-03-MeCN.

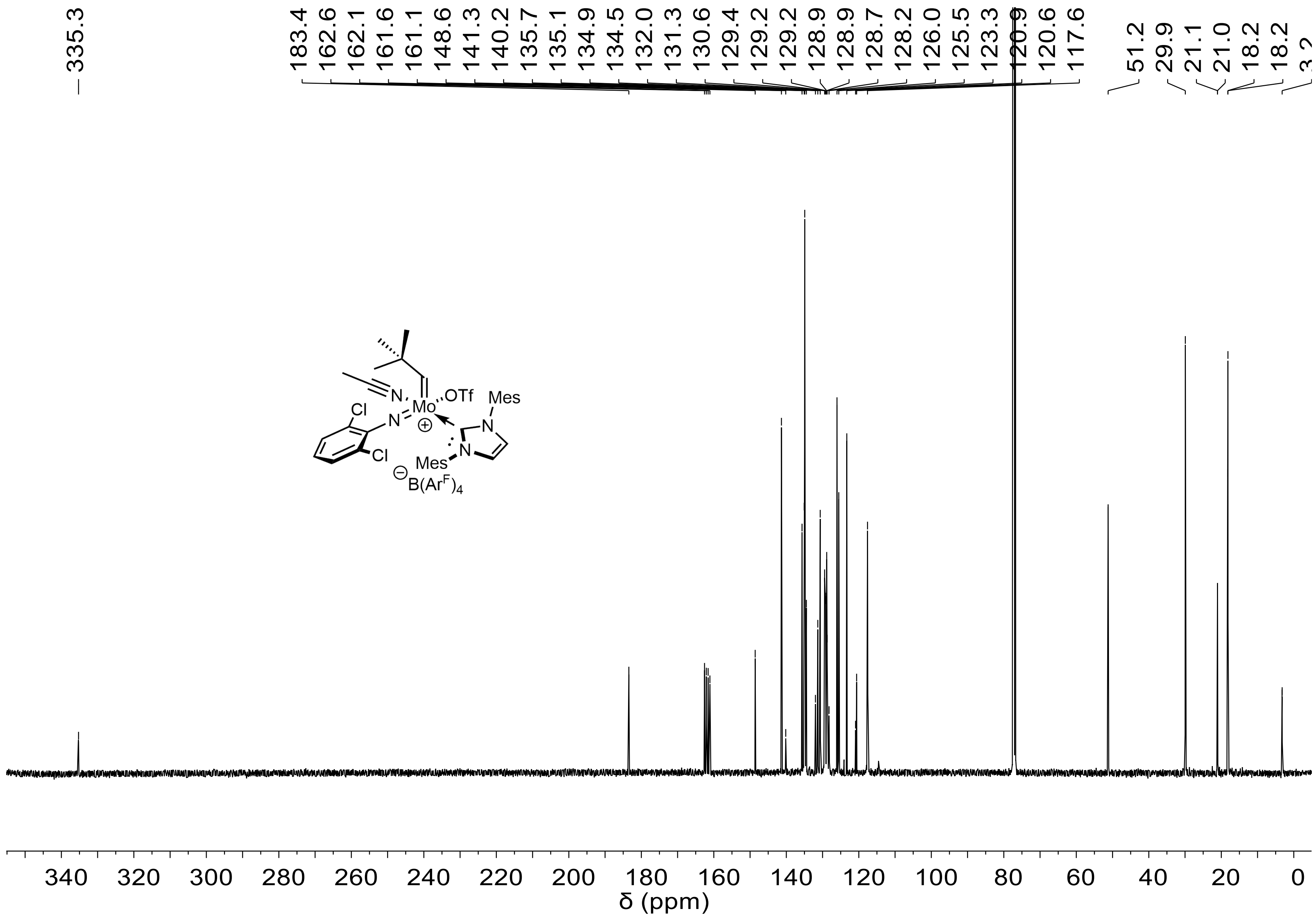

Figure S125: ^{13}C-NMR (101 MHz, 25 °C, $CDCl_3$) of Mo-03-MeCN.

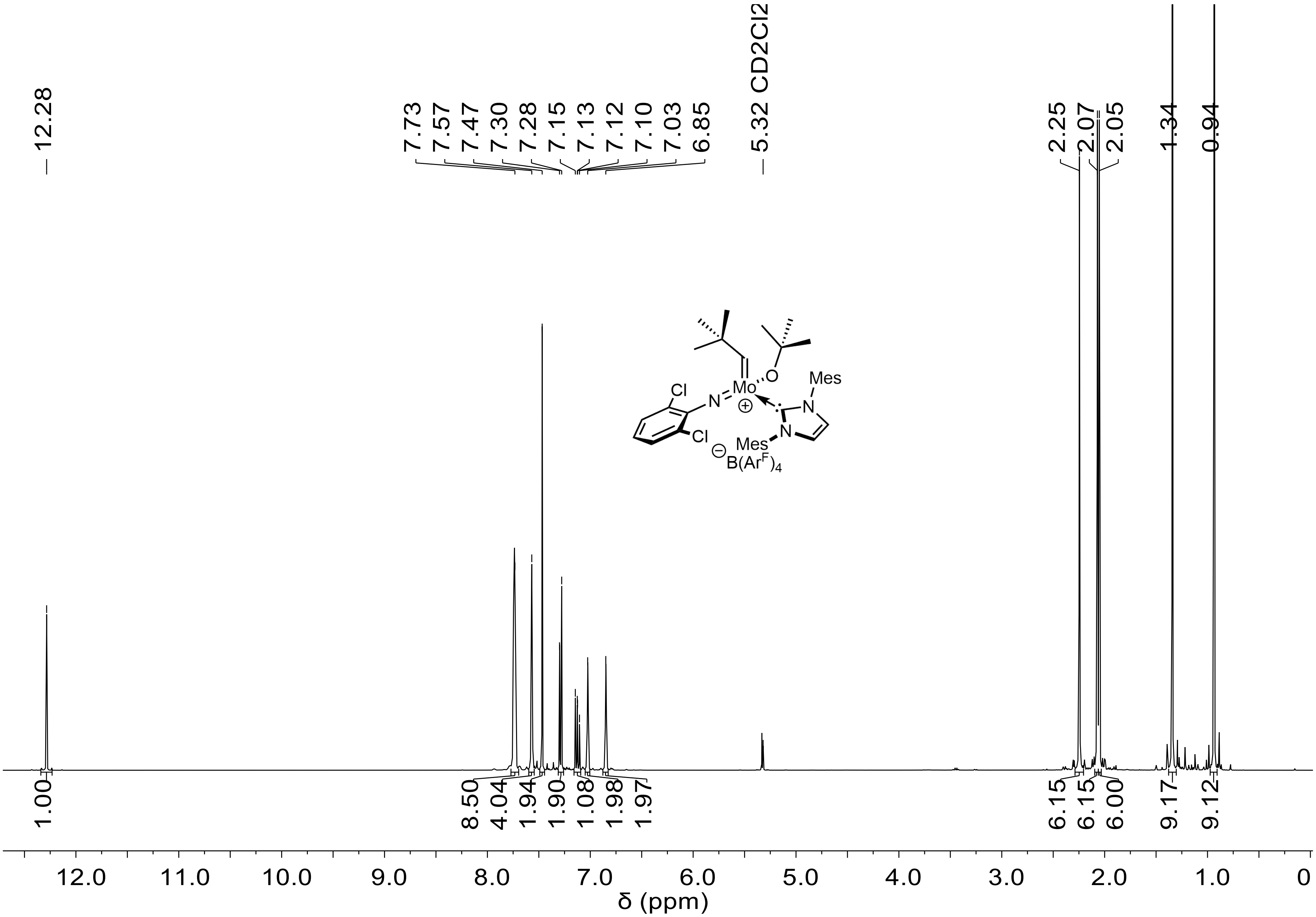

Figure S126: ^{1}H-NMR (400 MHz, 25 °C, CD_2Cl_2) of Mo-05.

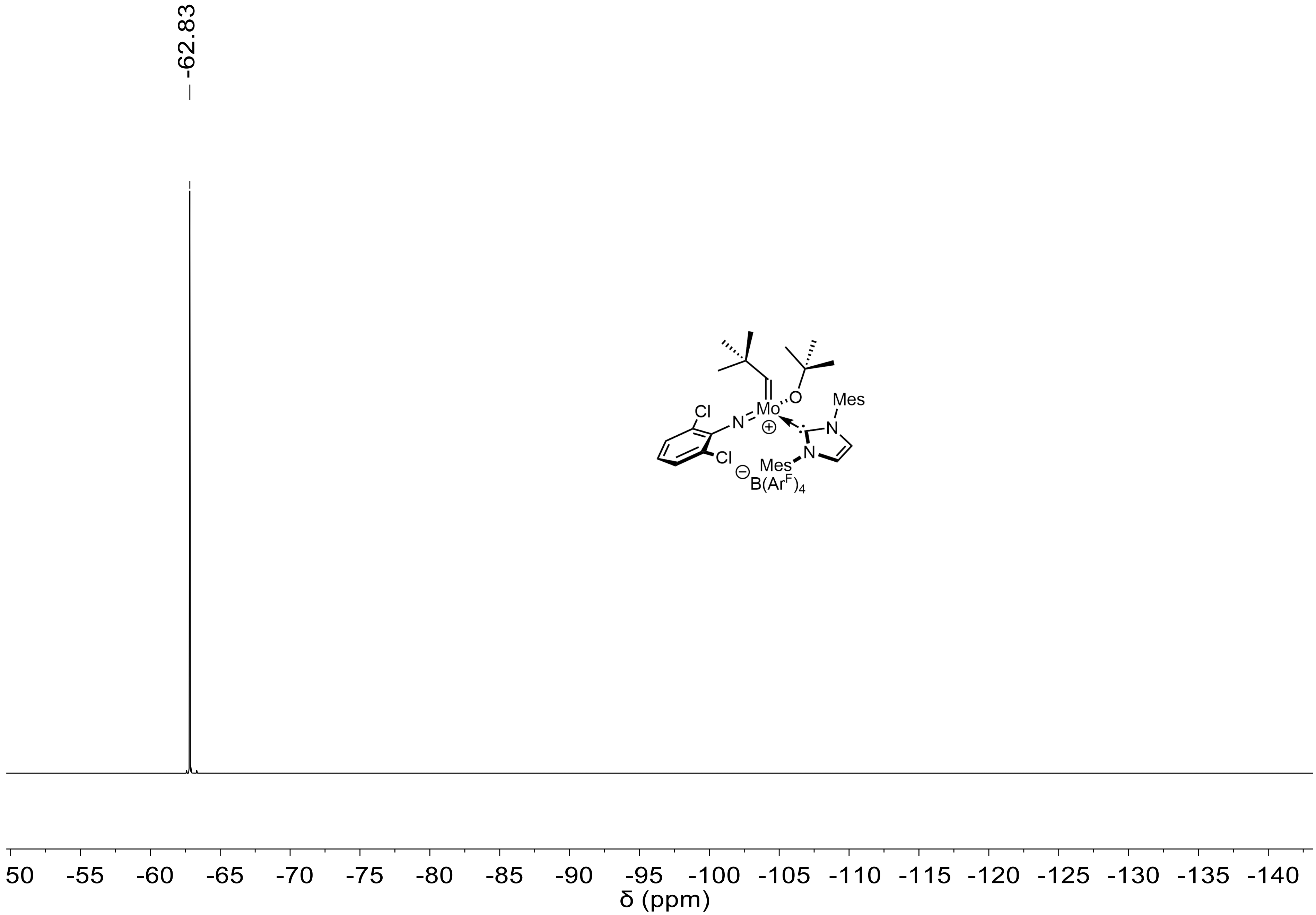

Figure S127: ^{19}F-NMR (376 MHz, 25 °C, CD_2Cl_2) of Mo-05.

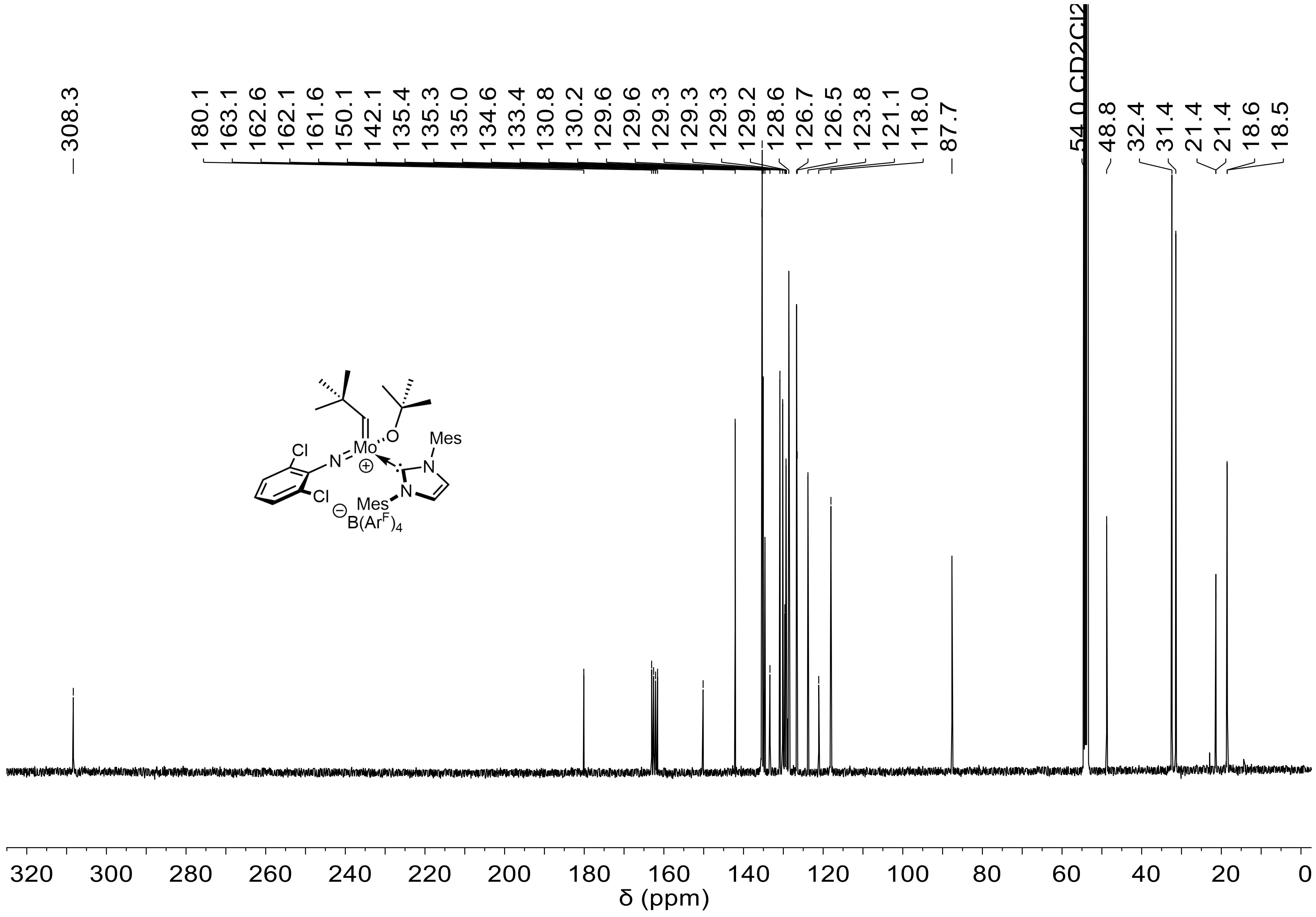

Figure S128: ^{13}C-NMR (101 MHz, 25 °C, CD_2Cl_2) of Mo-05.

5. NMR spectra of Air-Stability Experiments

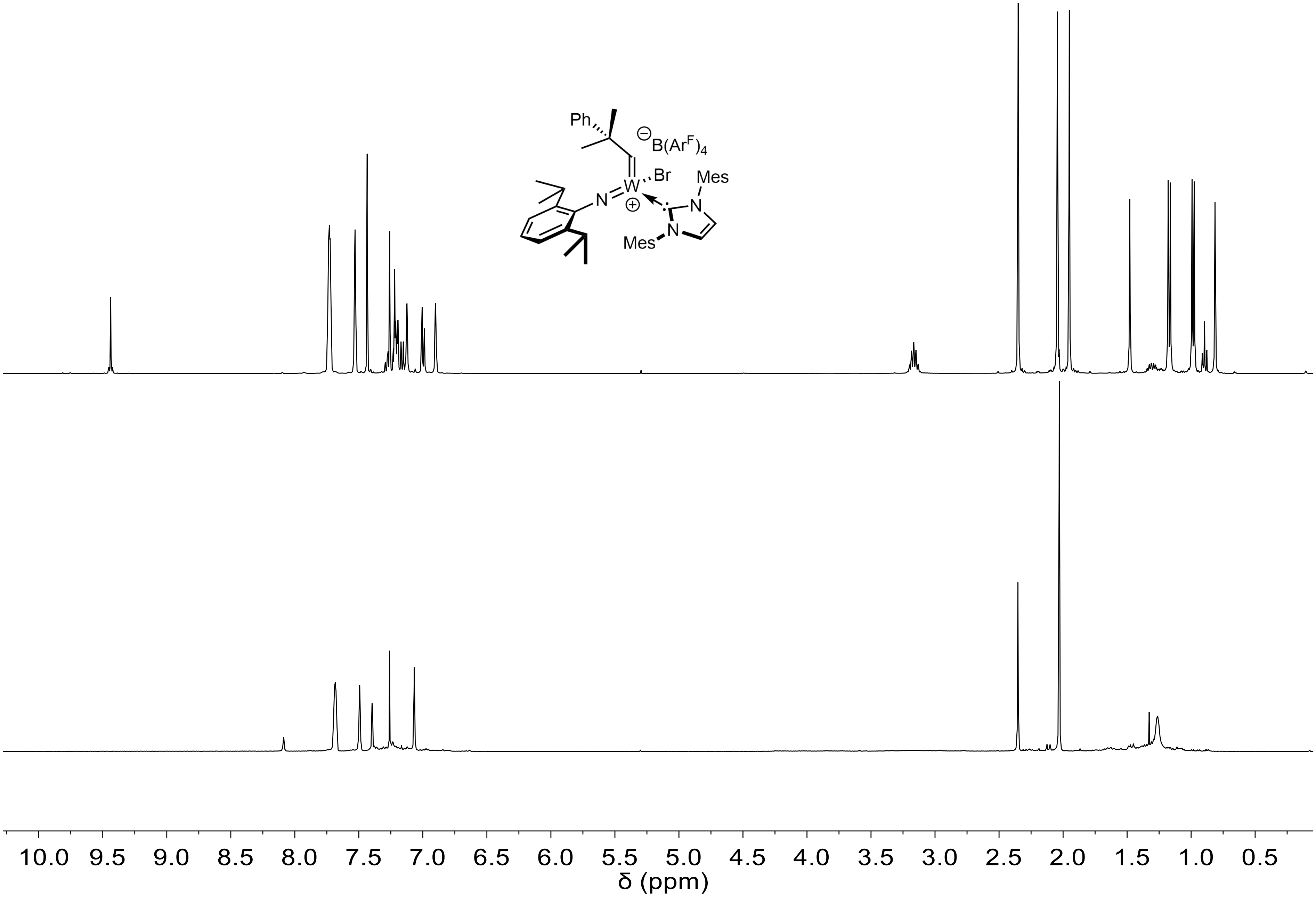

Figure S 129: ^{1}H-NMR (400 MHz, 25 °C, $CDCl_3$) of W-8 (upper) and W-8 after exposure to air overnight (lower).

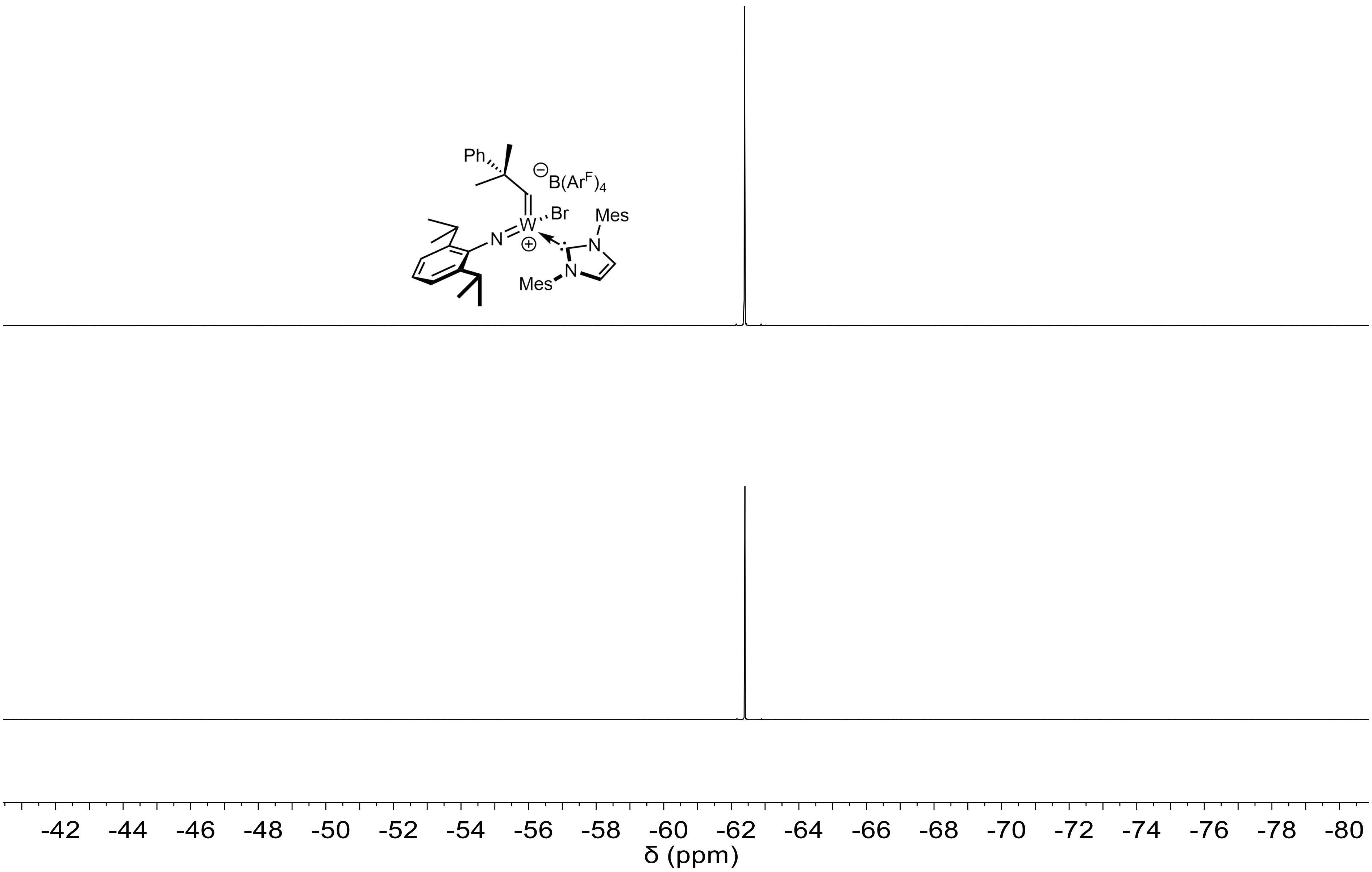

Figure S 130: ^{19}F-NMR (376 MHz, 25 °C, $CDCl_3$) of W-8 (upper) and W-8 after exposure to air overnight (lower).

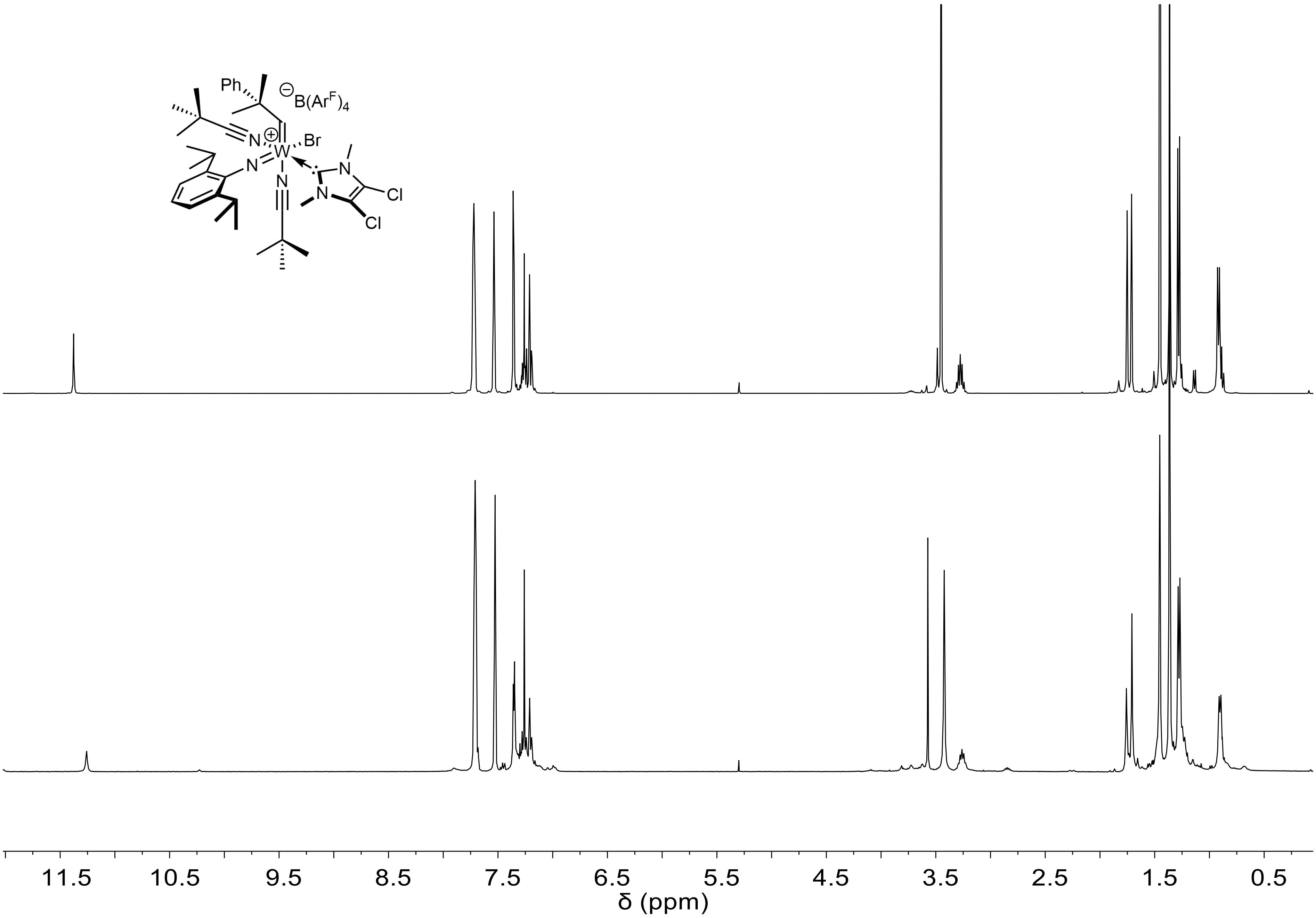

Figure S 131: ¹H-NMR (400 MHz, 25 °C, $CDCl_3$) of W-9 (upper) and W-9 after exposure to air overnight (lower).

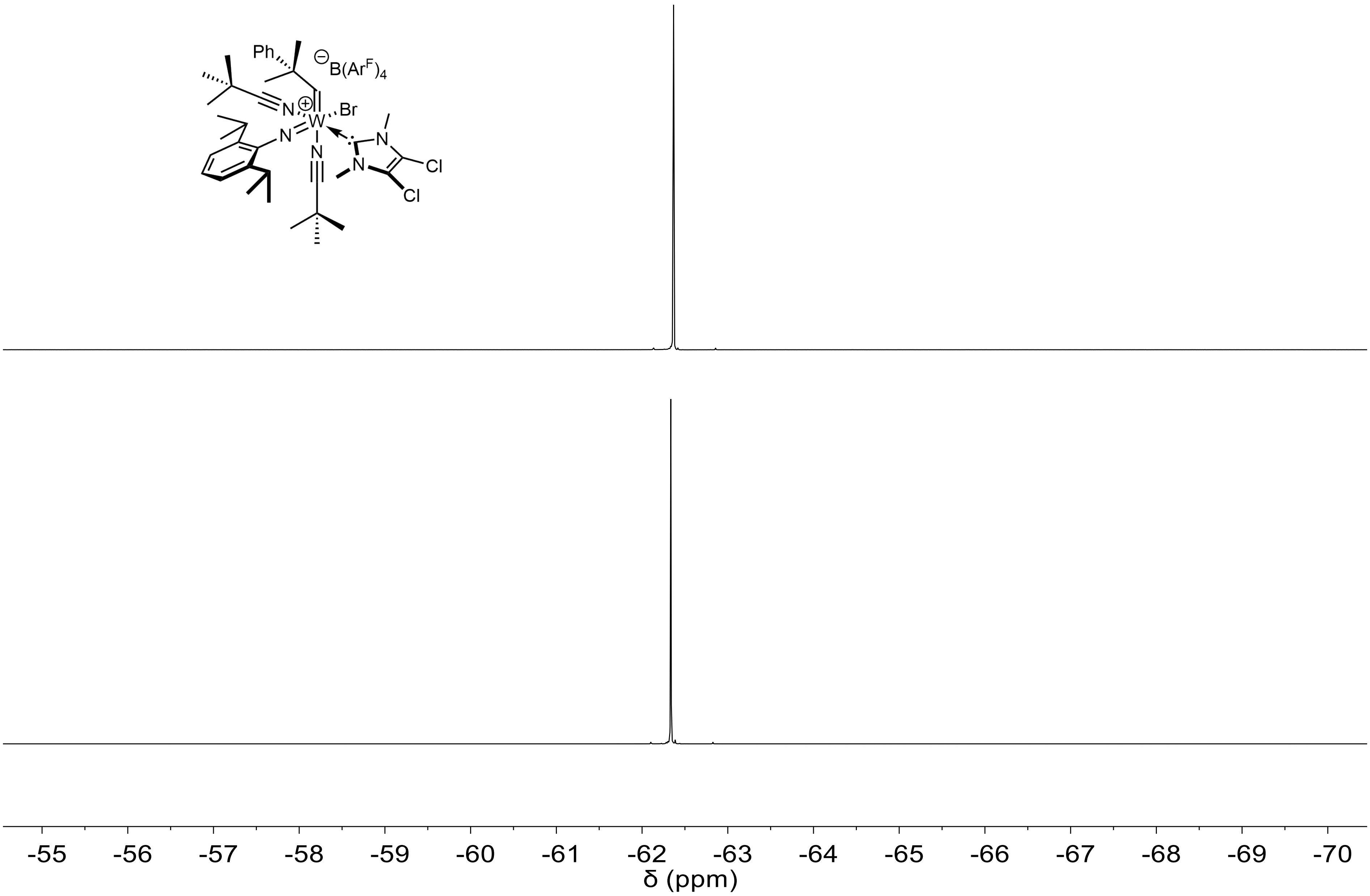

Figure S 132: ^{19}F-NMR (376 MHz, 25 °C, $CDCl_3$) of W-9 (upper) and W-9 after exposure to air overnight (lower).

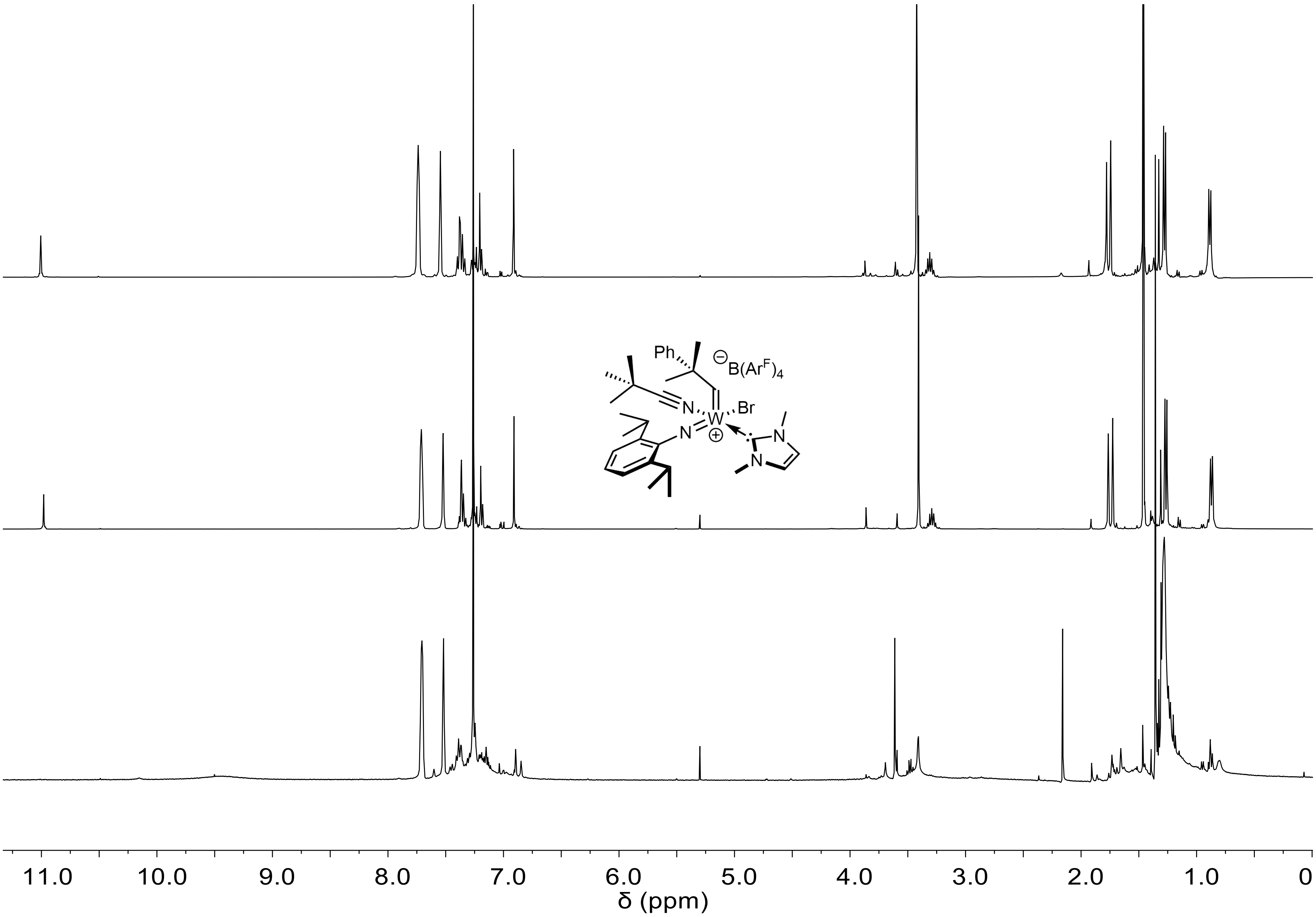

Figure S 133: ^{1}H-NMR (400 MHz, 25 °C, $CDCl_3$) of W-10 (upper), W-10 after exposure to air overnight (middle) and W-10 after exposure to air for two weeks (lower).

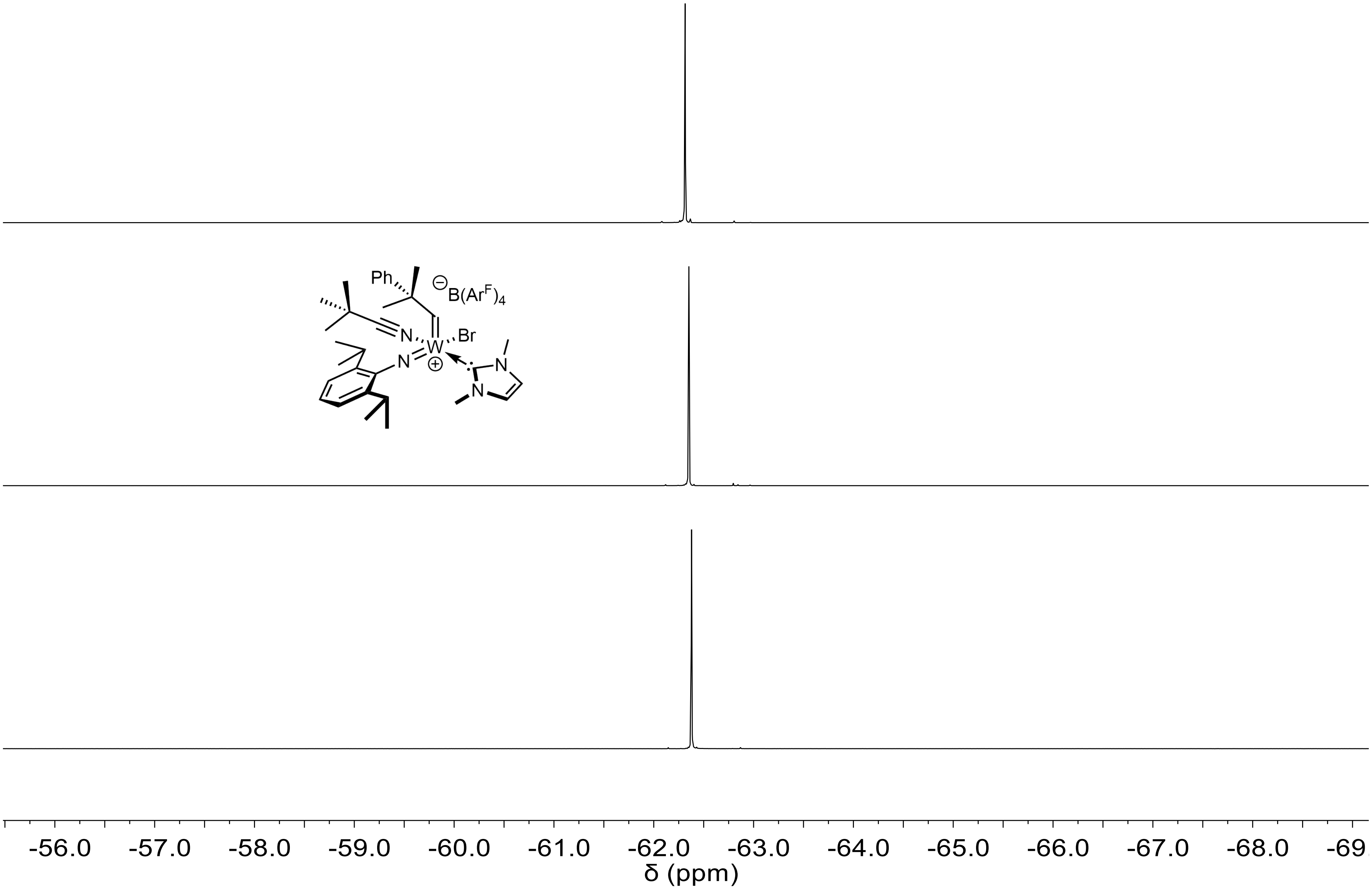

Figure S 134. ^{19}F-NMR (376 MHz, 25 °C, $CDCl_3$) of W-10 (upper), W-10 after exposure to air overnight (middle) and W-10 after exposure to air for two weeks (lower).

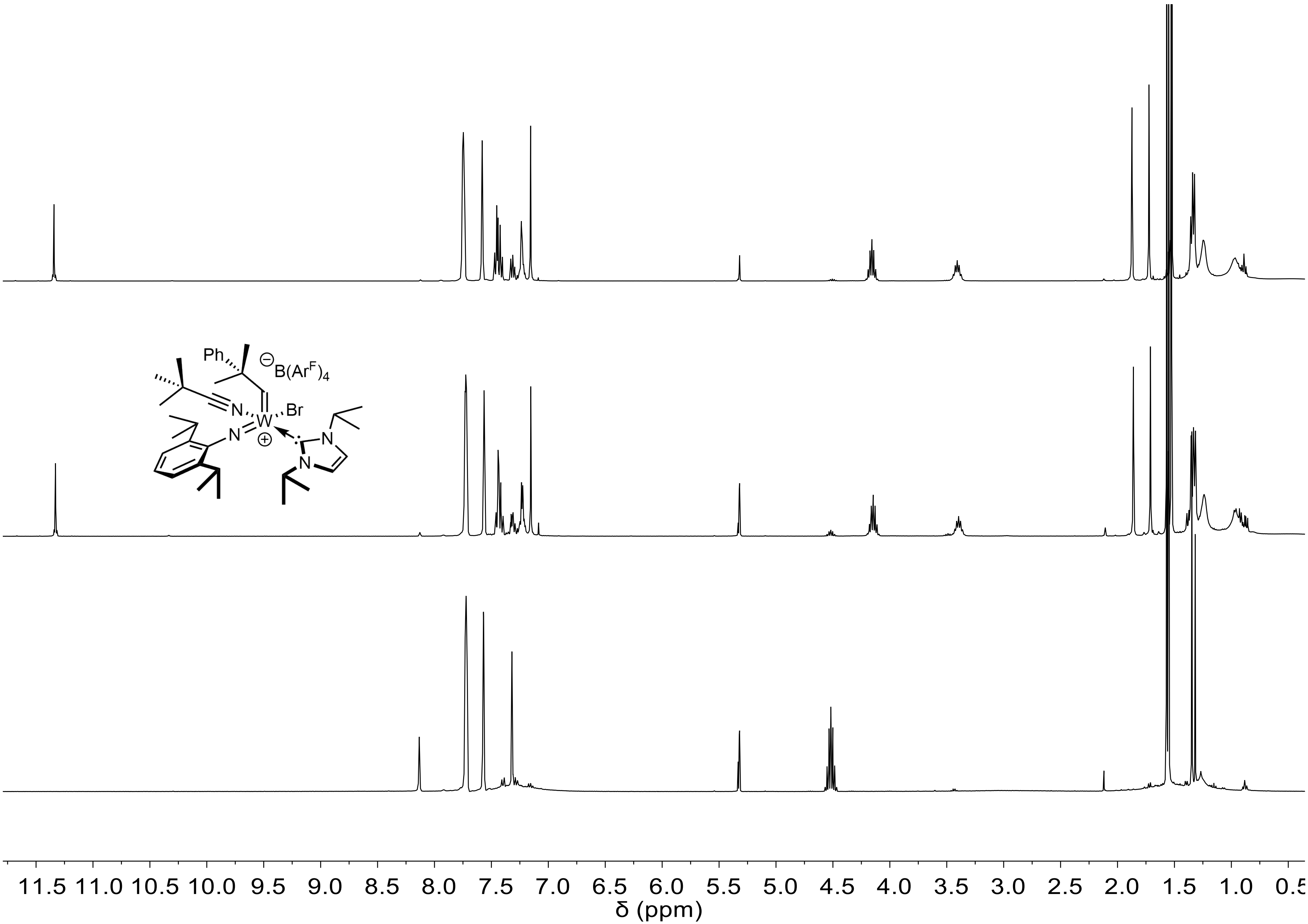

Figure S 135: ^{1}H-NMR (400 MHz, 25 °C, CD_2Cl_2) of W-11 (upper), W-11 after exposure to air overnight (middle) and W-11 after exposure to air for two weeks (lower).

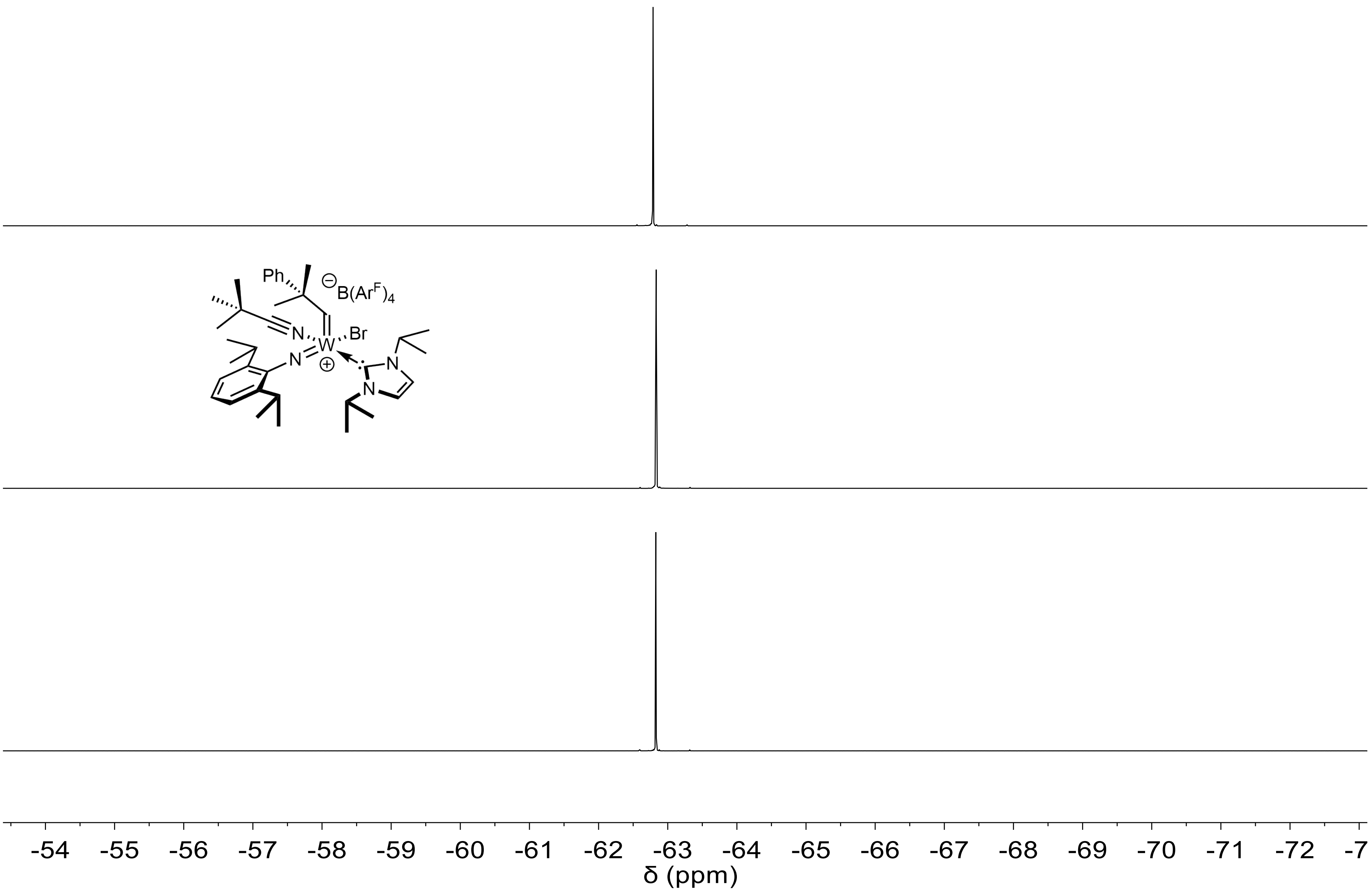

Figure S 136: ^{19}F-NMR (376 MHz, 25 °C, CD_2Cl_2) of W-11 (upper), W-11 after exposure to air overnight (middle) and W-11 after exposure to air for two weeks (lower).

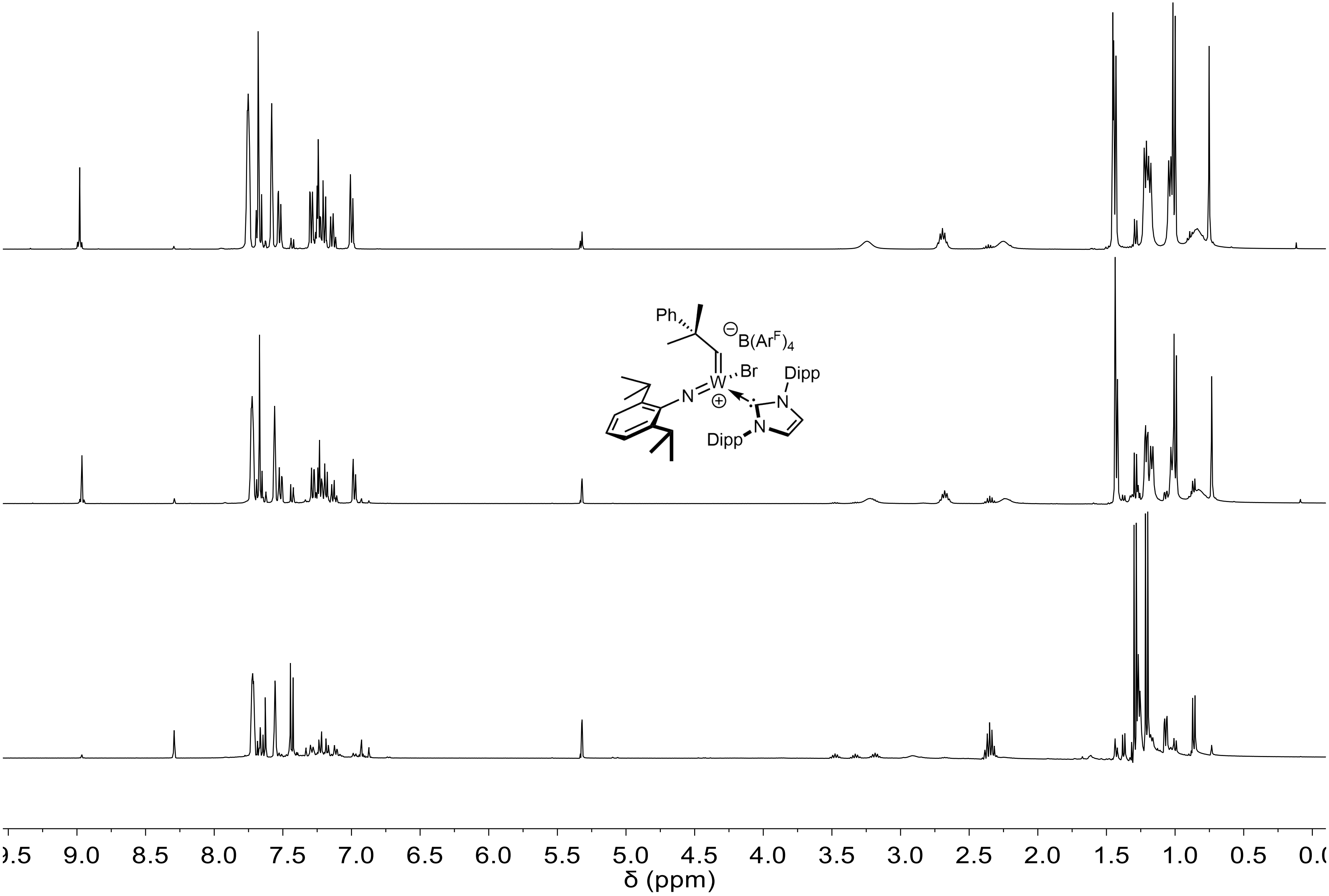

Figure S 137: ^{1}H-NMR (400 MHz, 25 °C, CD_2Cl_2) of W-12 (upper), W-12 after exposure to air overnight (middle) and W-12 after exposure to air for two weeks (lower).

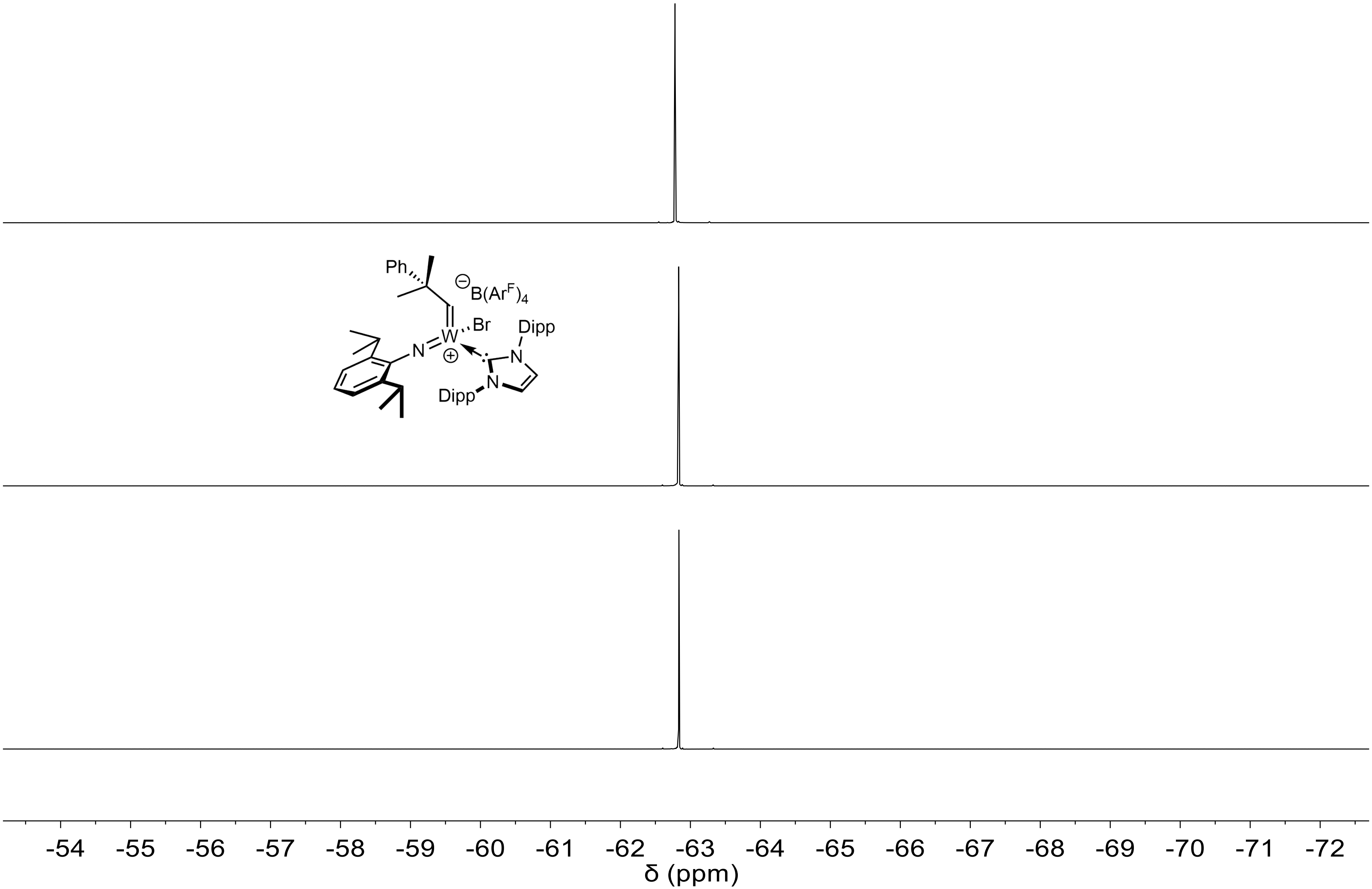

Figure S 138: ^{19}F-NMR (376 MHz, 25 °C, CD_2Cl_2) of W-12 (upper), W-12 after exposure to air overnight (middle) and W-12 after exposure to air for two weeks (lower).

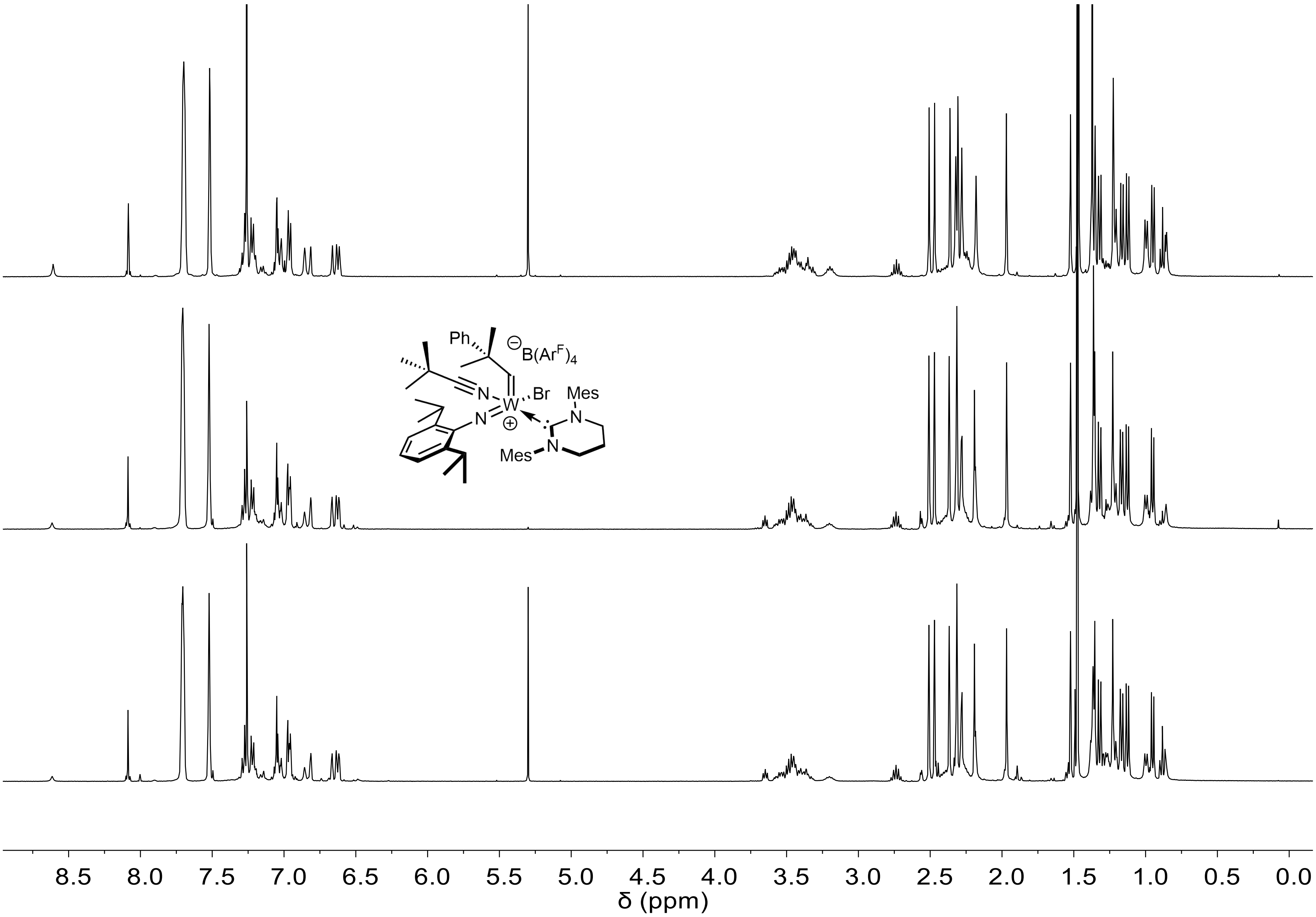

Figure S 139: ^{1}H-NMR (400 MHz, 25 °C, $CDCl_3$) of W-14 (upper), W-14 after exposure to air overnight (middle) and W-14 after exposure to air for two weeks (lower).

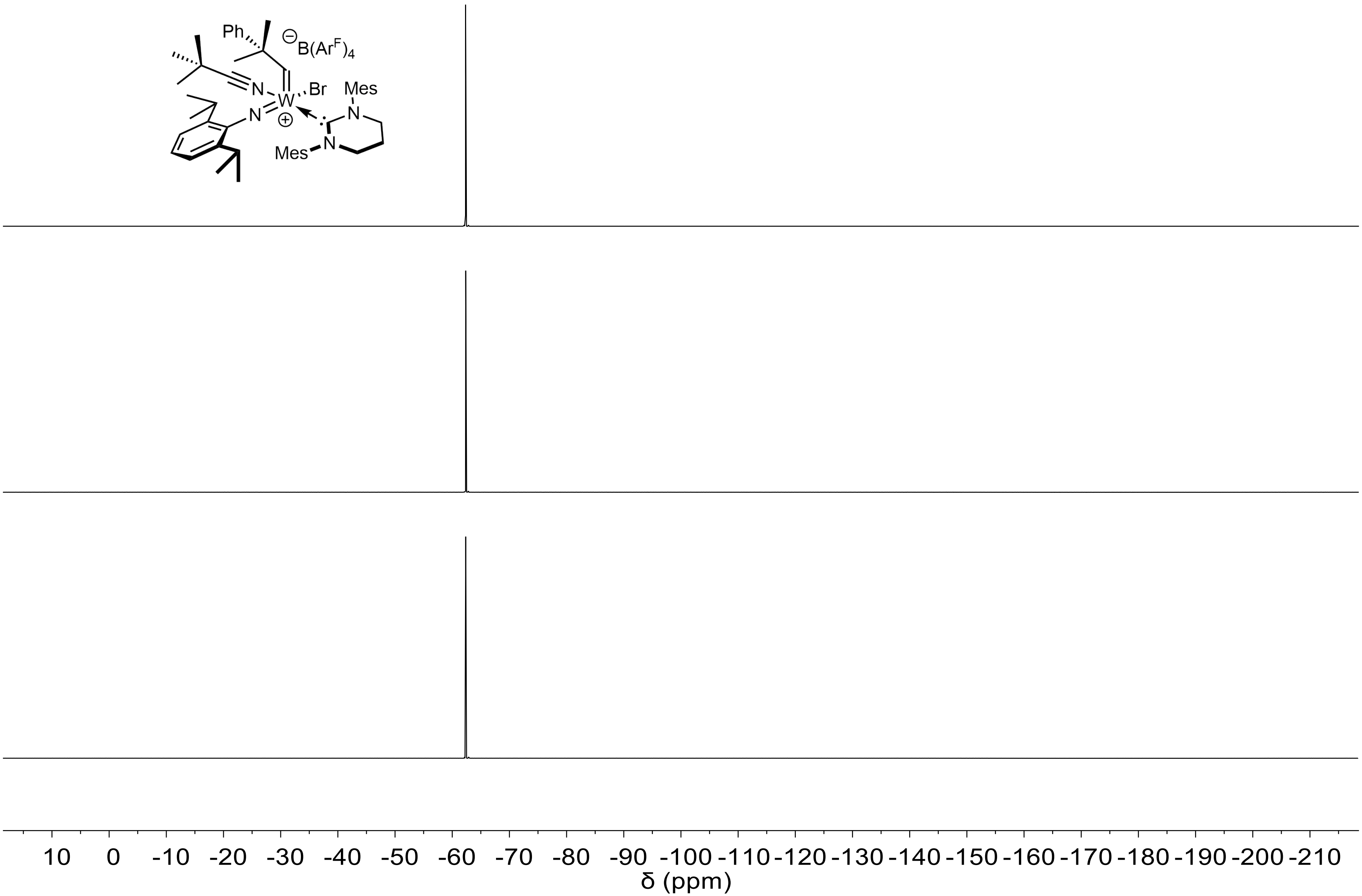

Figure S 140: ^{19}F-NMR (376 MHz, 25 °C, $CDCl_3$) of W-14 (upper), W-14 after exposure to air overnight (middle) and W-14 after exposure to air for two weeks (lower).

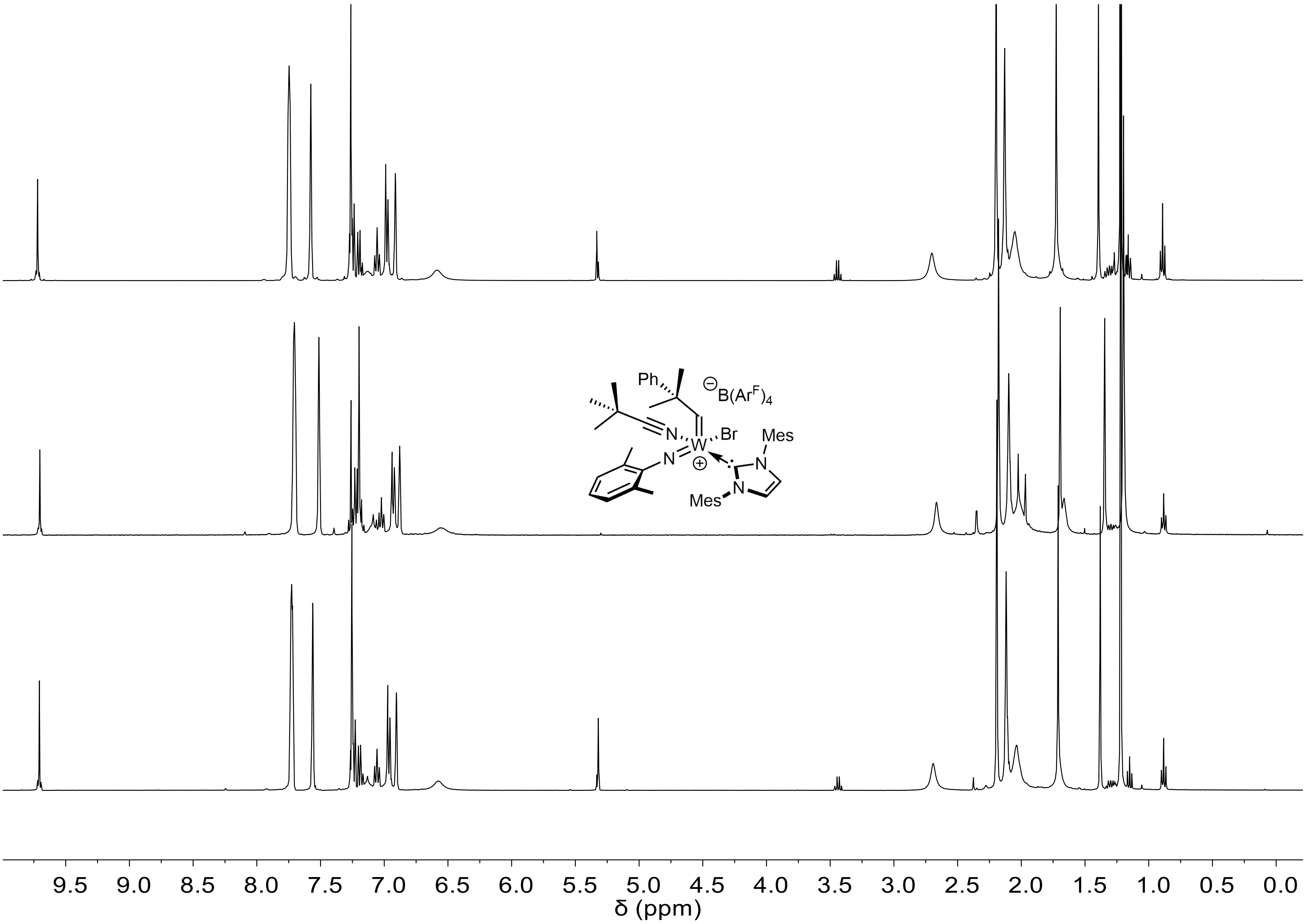

Figure S 141: ^{1}H-NMR (400 MHz, 25 °C, CD_2Cl_2) of W-25 (upper), W-25 after exposure to air overnight (middle) and W-25 after exposure to air for two weeks (lower).

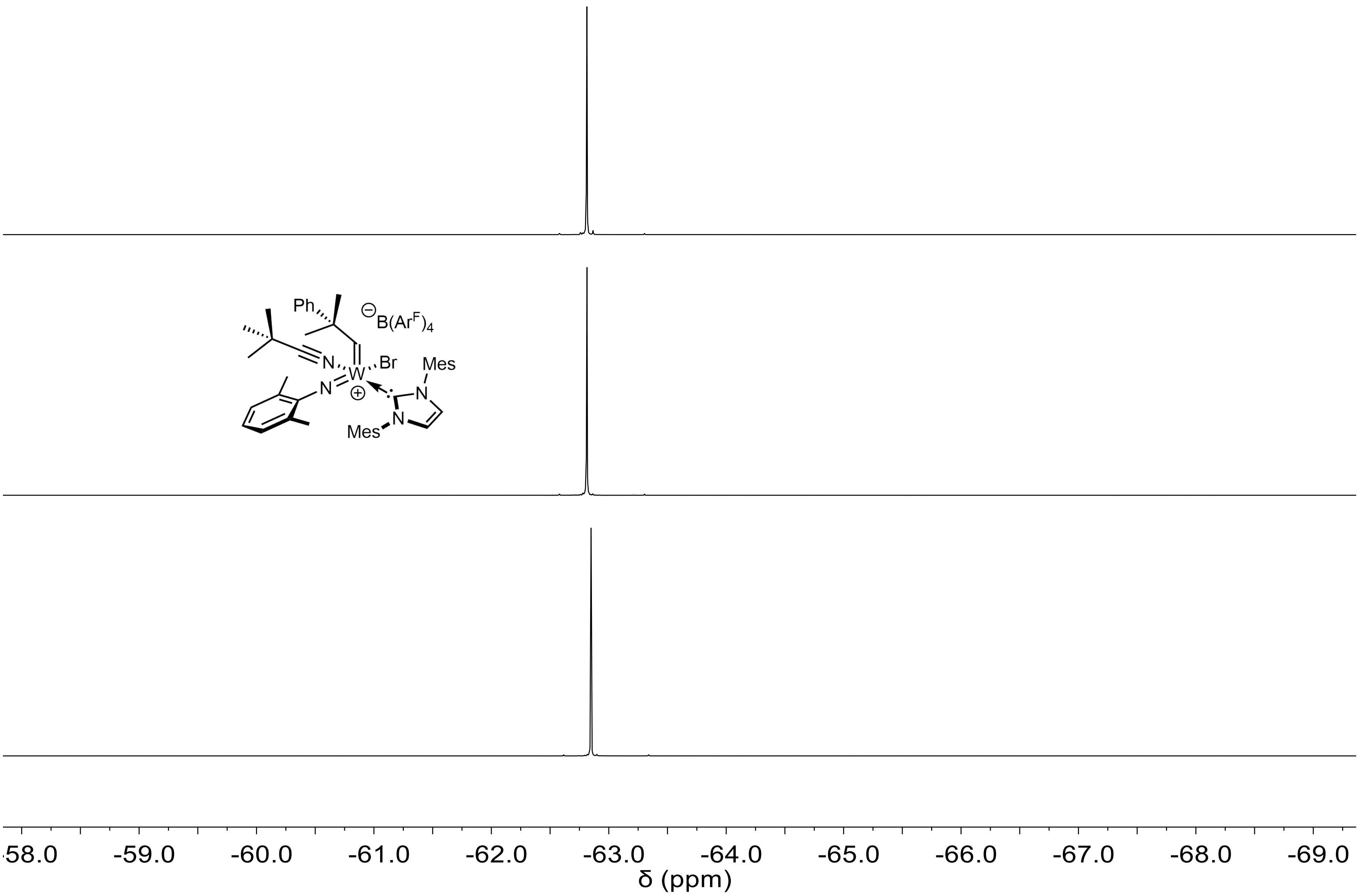

Figure S 142: ^{19}F-NMR (376 MHz, 25 °C, CD_2Cl_2) of W-25 (upper), W-25 after exposure to air overnight (middle) and W-25 after exposure to air for two weeks (lower).

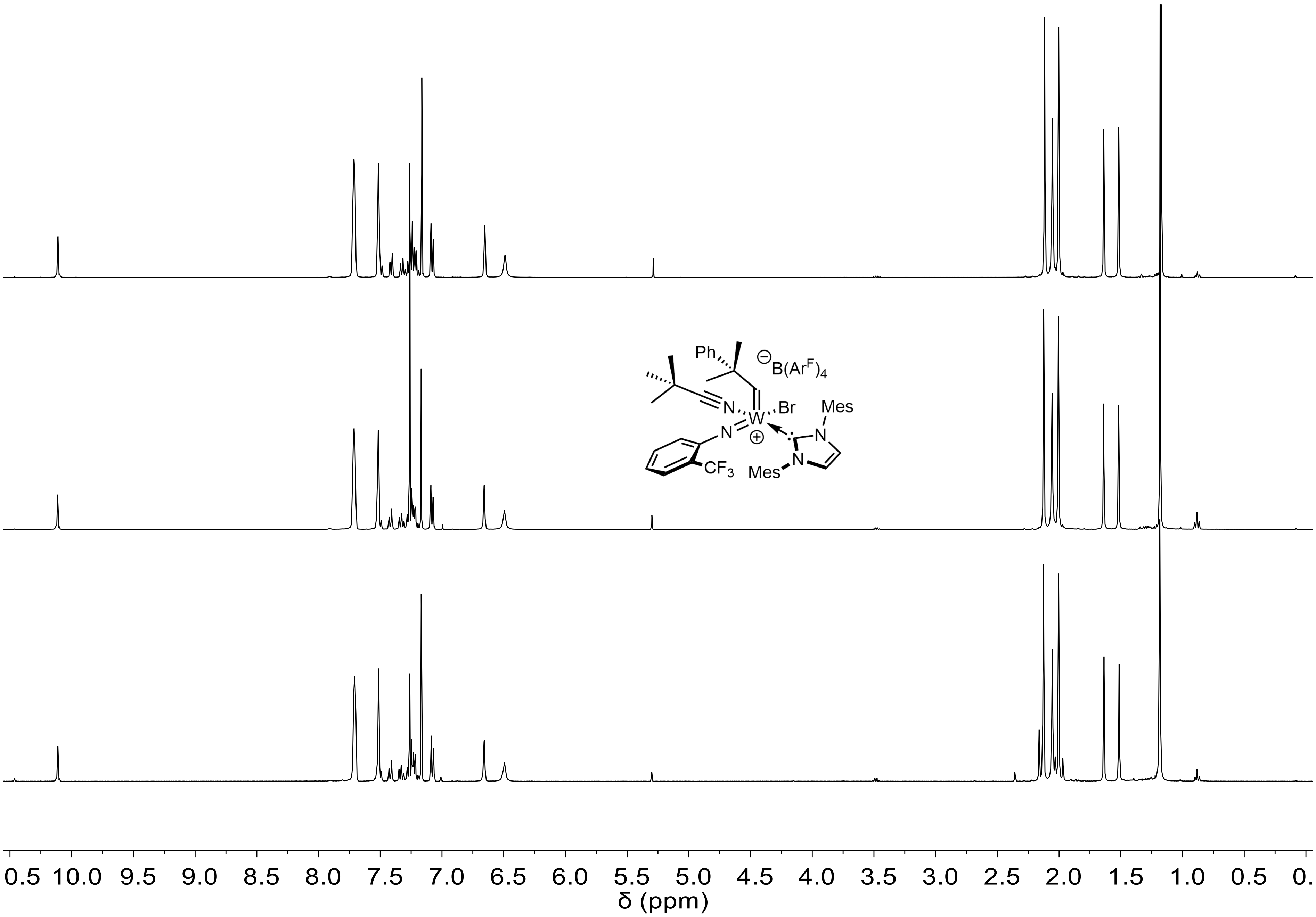

Figure S 143: ^{1}H-NMR (400 MHz, 25 °C, $CDCl_3$) of W-26 (upper), W-26 after exposure to air overnight (middle) and W-26 after exposure to air for two weeks (lower).

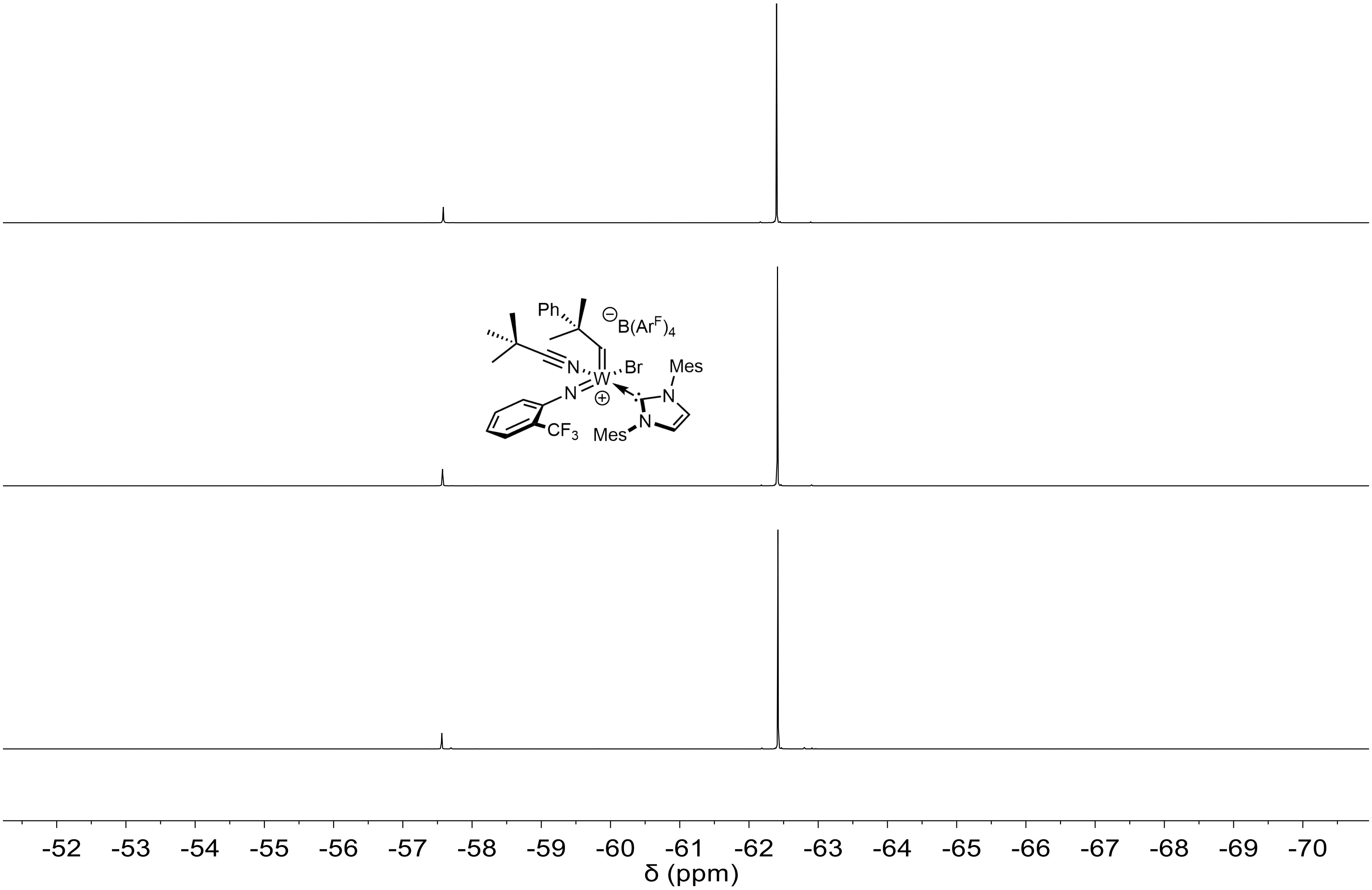

Figure S 144: ^{19}F-NMR (376 MHz, 25 °C, $CDCl_3$) of W-26 (upper), W-26 after exposure to air overnight (middle) and W-26 after exposure to air for two weeks (lower).

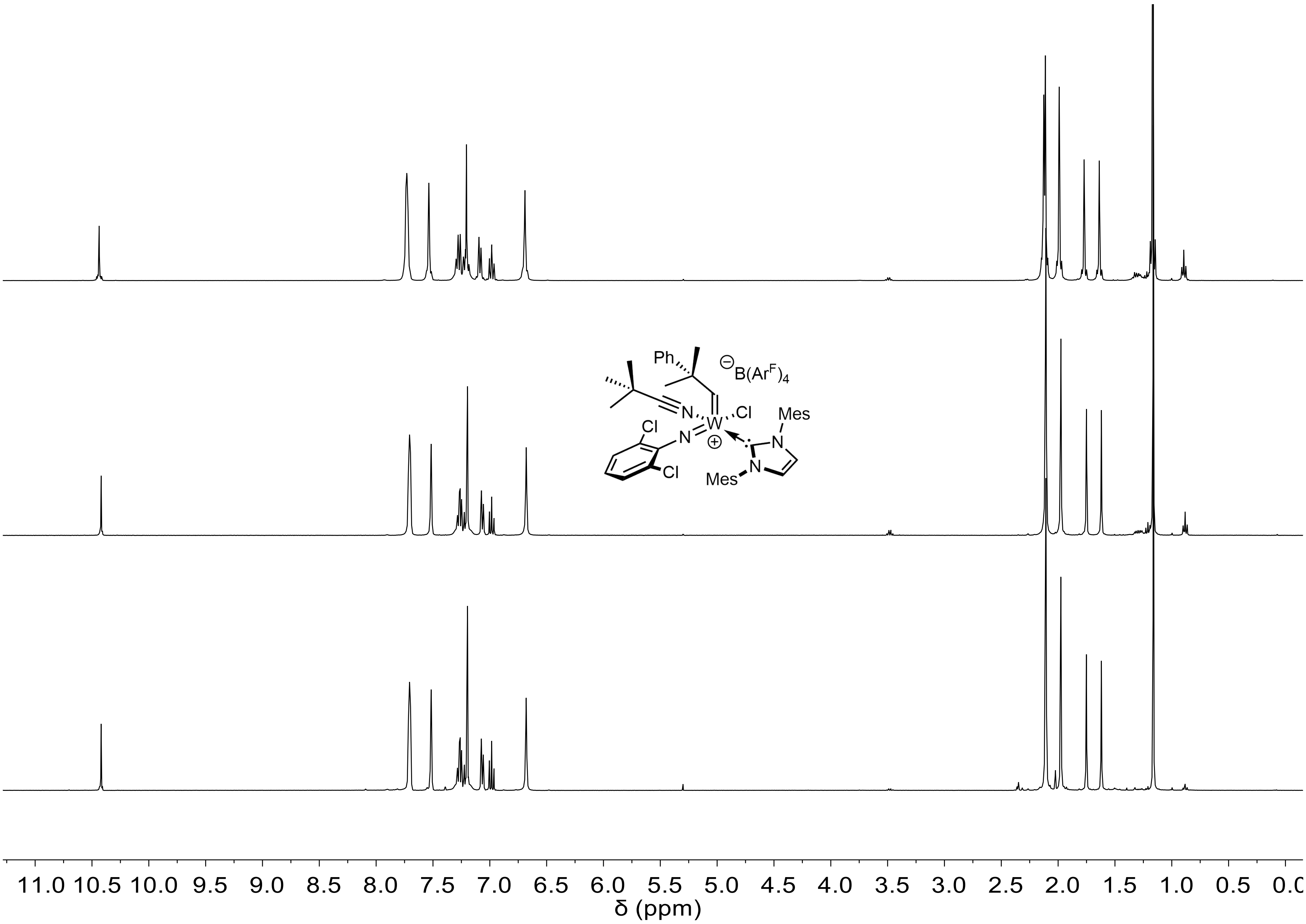

Figure S 145: ^{1}H-NMR (400 MHz, 25 °C, $CDCl_3$) of W-27 (upper), W-27 after exposure to air overnight (middle) and W-27 after exposure to air for two weeks (lower).

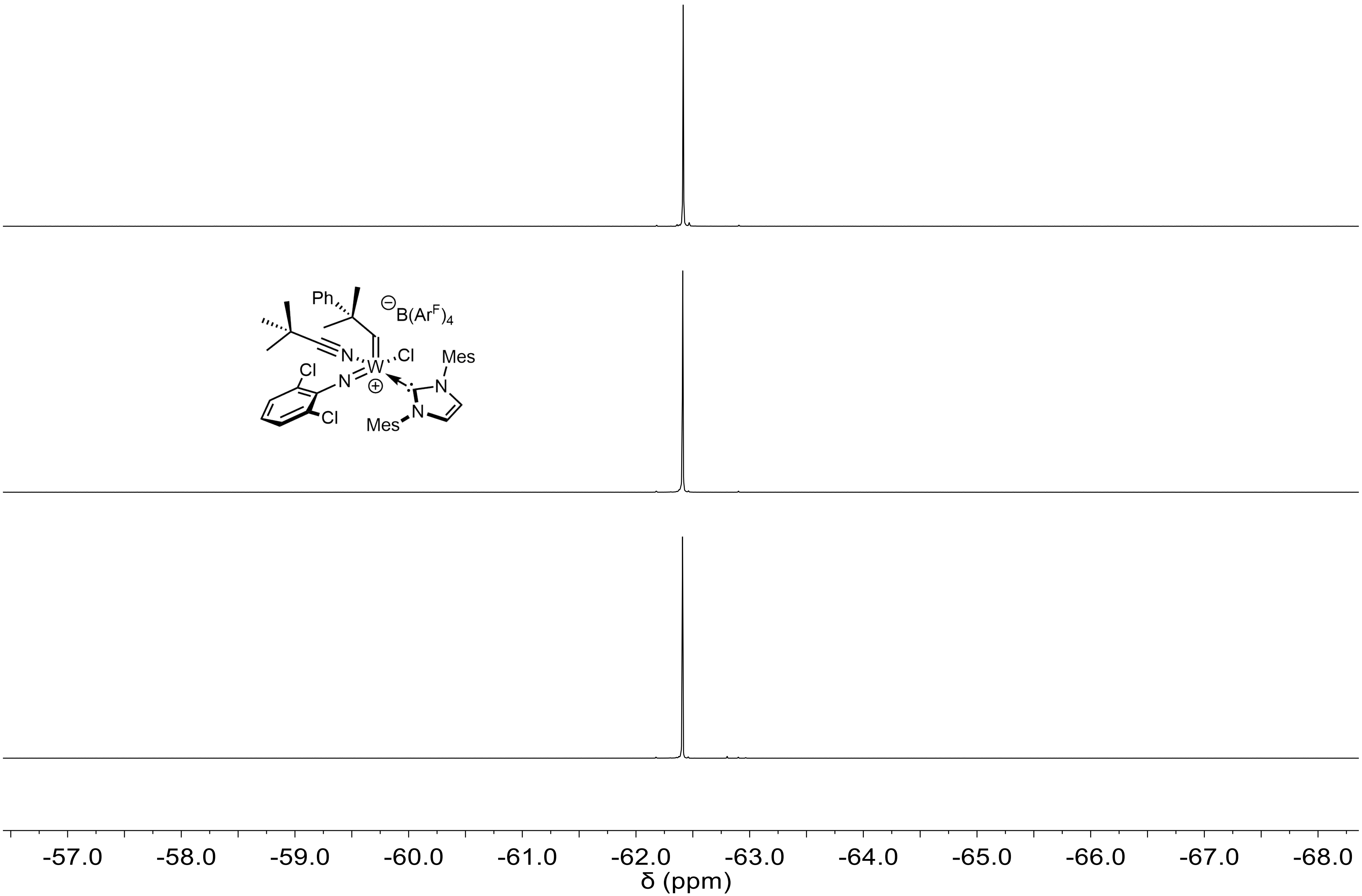

Figure S 146: ^{19}F-NMR (376 MHz, 25 °C, $CDCl_3$) of W-27 (upper), W-27 after exposure to air overnight (middle) and W-27 after exposure to air for two weeks (lower).

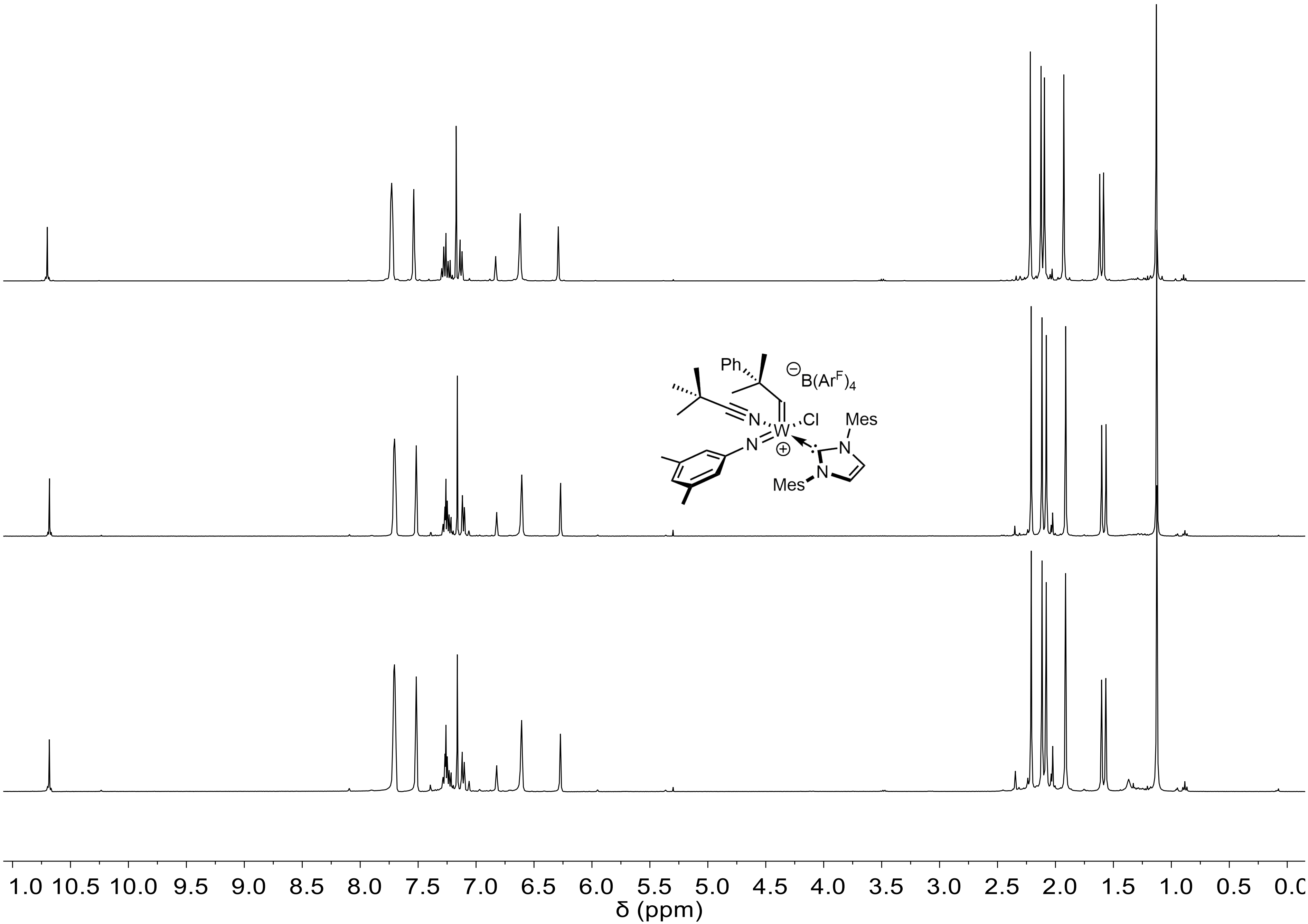

Figure S 147: ^{1}H-NMR (400 MHz, 25 °C, $CDCl_3$) of W-28 (upper), W-28 after exposure to air overnight (middle) and W-28 after exposure to air for two weeks (lower).

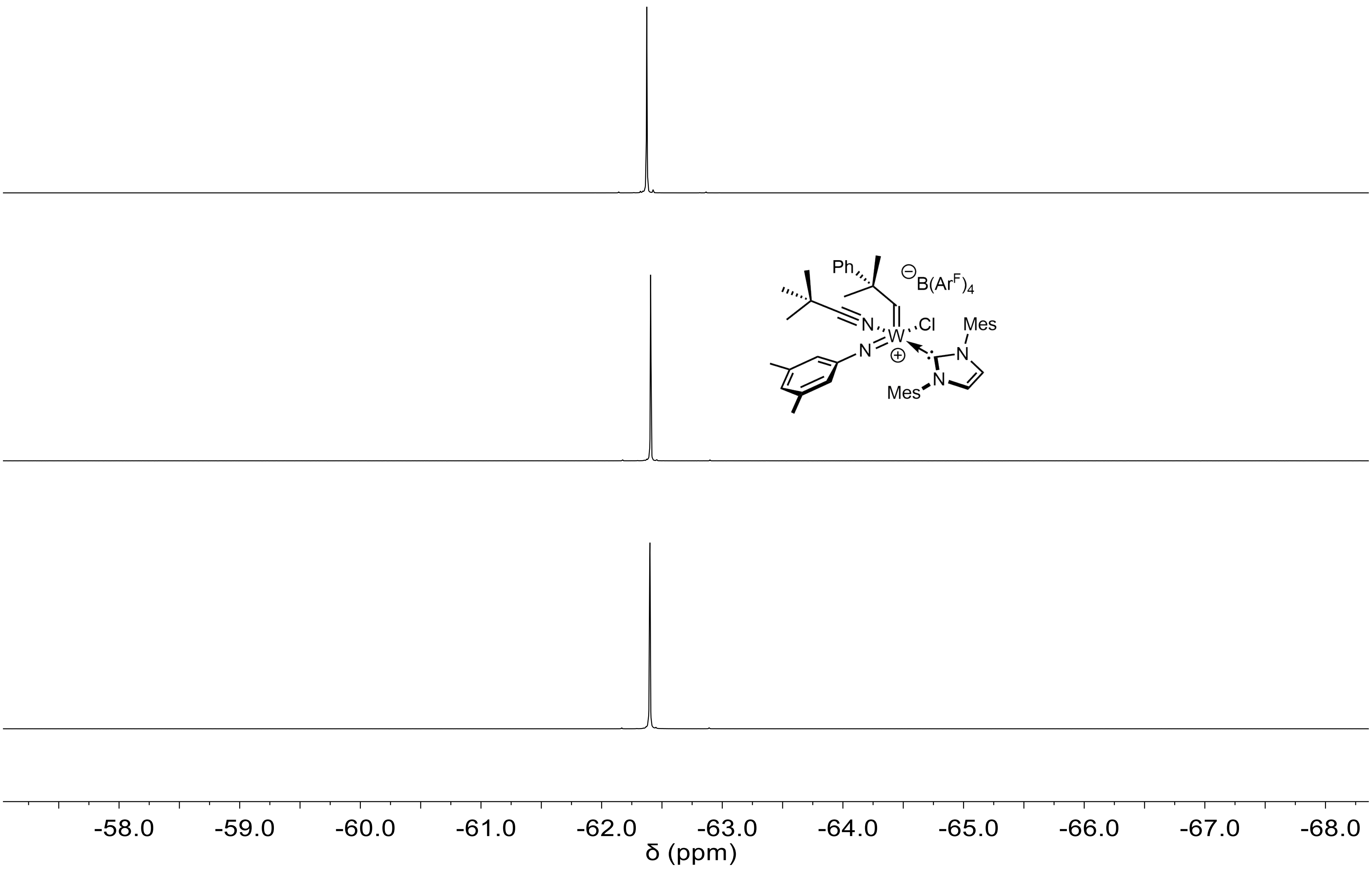

Figure S 148: ^{19}F-NMR (376 MHz, 25 °C, $CDCl_3$) of W-28 (upper), W-28 after exposure to air overnight (middle) and W-28 after exposure to air for two weeks (lower).

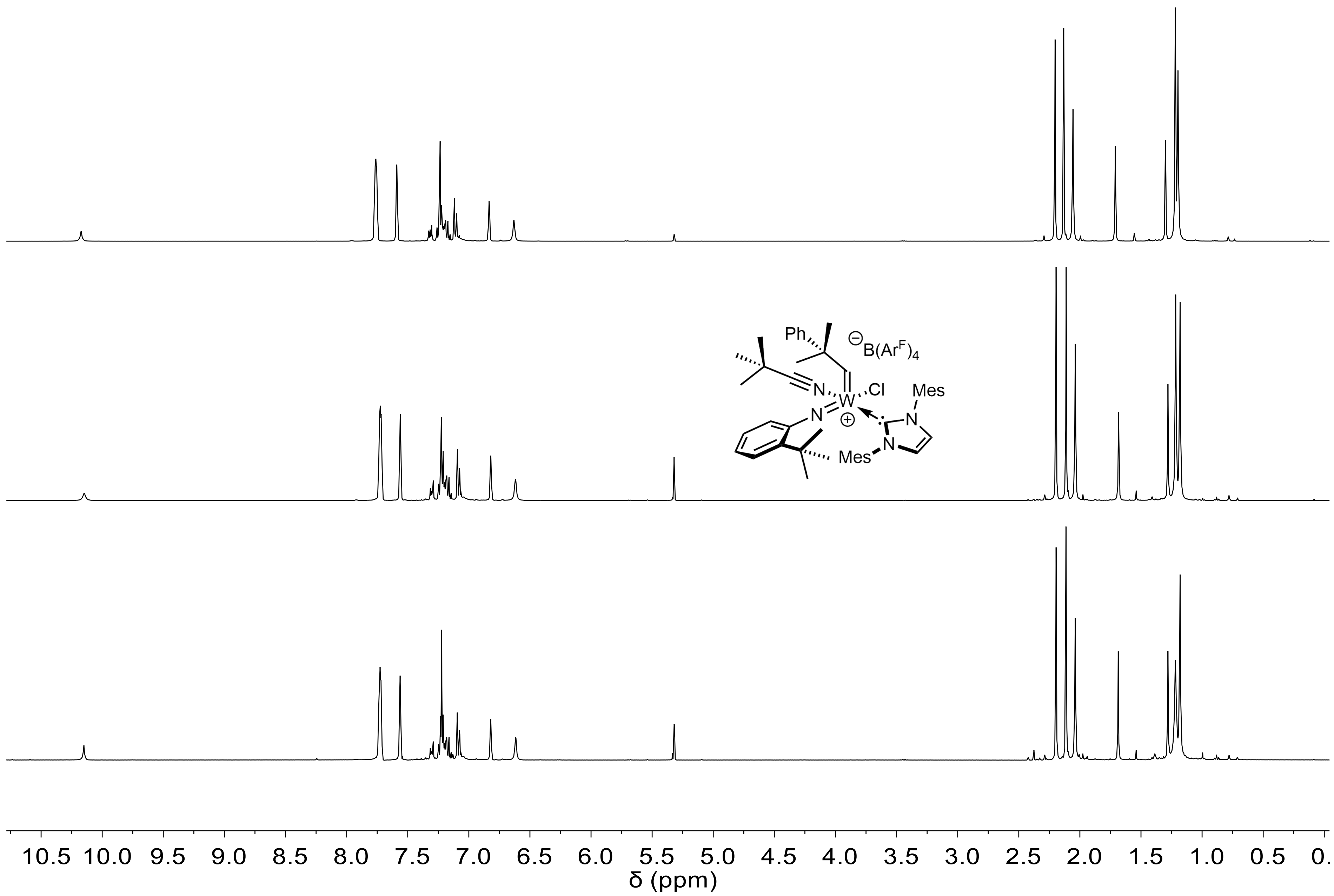

Figure S 149: ^{1}H-NMR (400 MHz, 25 °C, CD_2Cl_2) of W-29 (upper), W-29 after exposure to air overnight (middle) and W-29 after exposure to air for two weeks (lower).

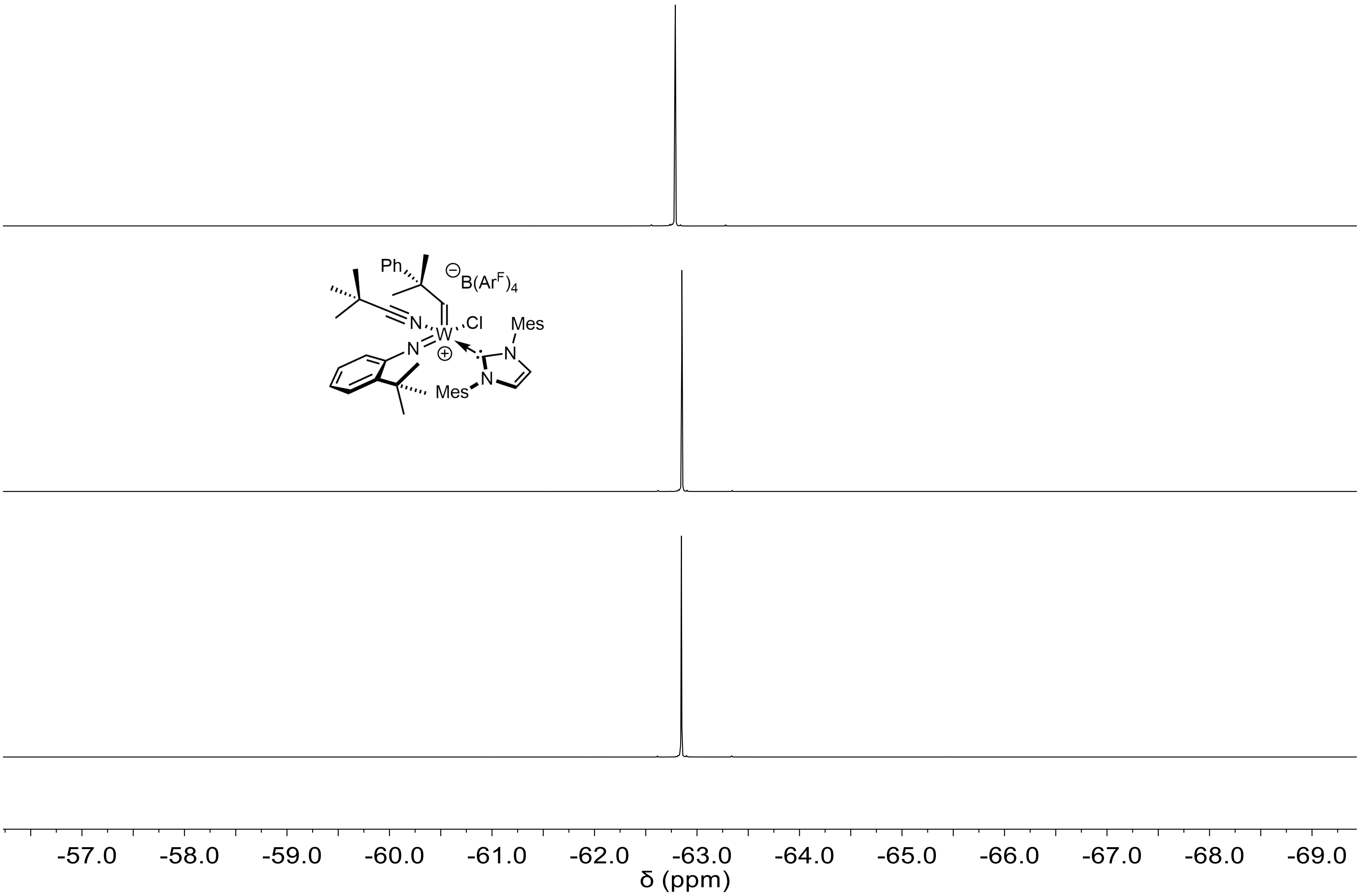

Figure S 150: ^{19}F-NMR (376 MHz, 25 °C, CD_2Cl_2) of W-29 (upper), W-29 after exposure to air overnight (middle) and W-29 after exposure to air for two weeks (lower).

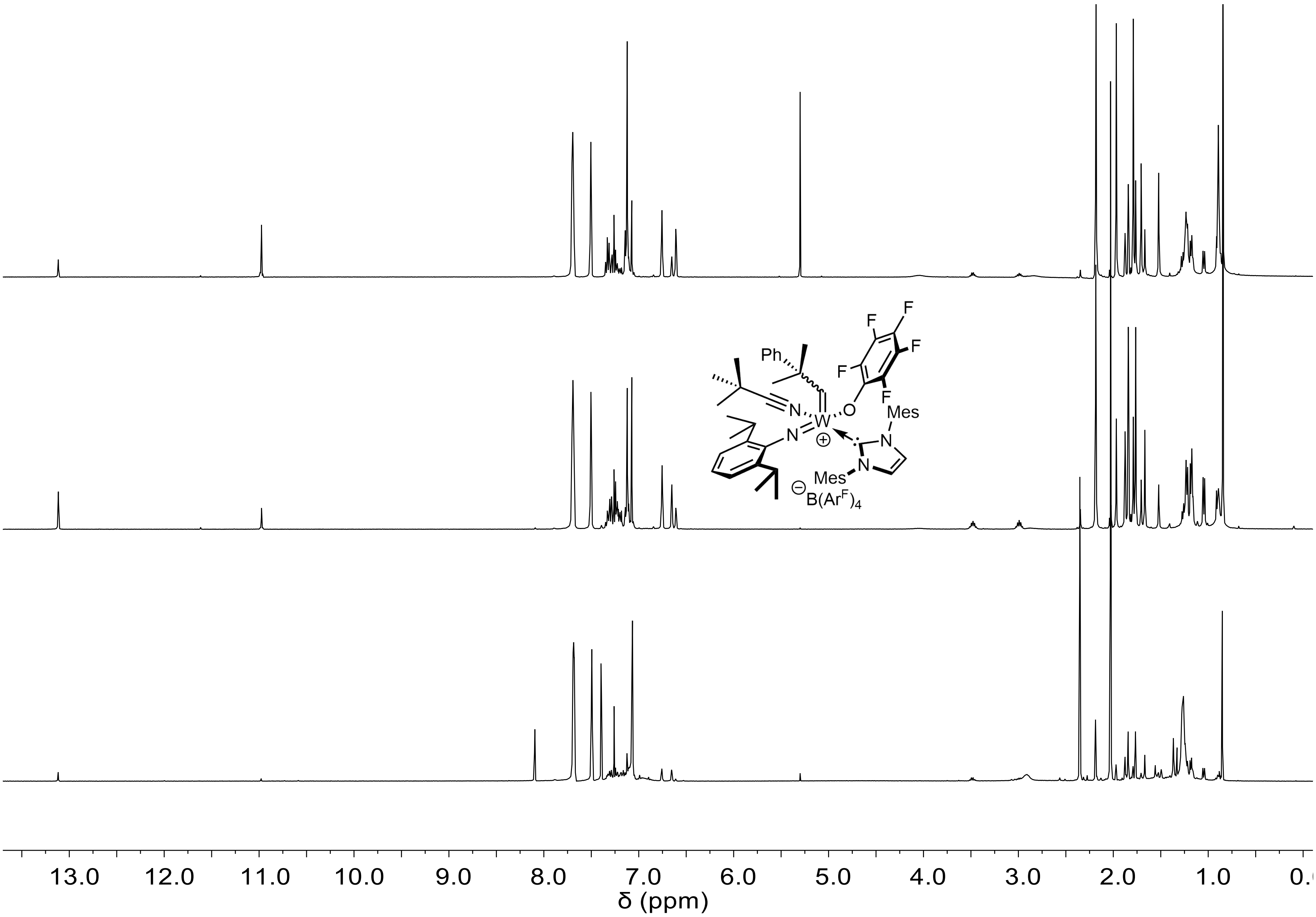

Figure S 151: ^{1}H-NMR (400 MHz, 25 °C, $CDCl_3$) of W-30 (upper), W-30 after exposure to air overnight (middle) and W-30 after exposure to air for two weeks (lower).

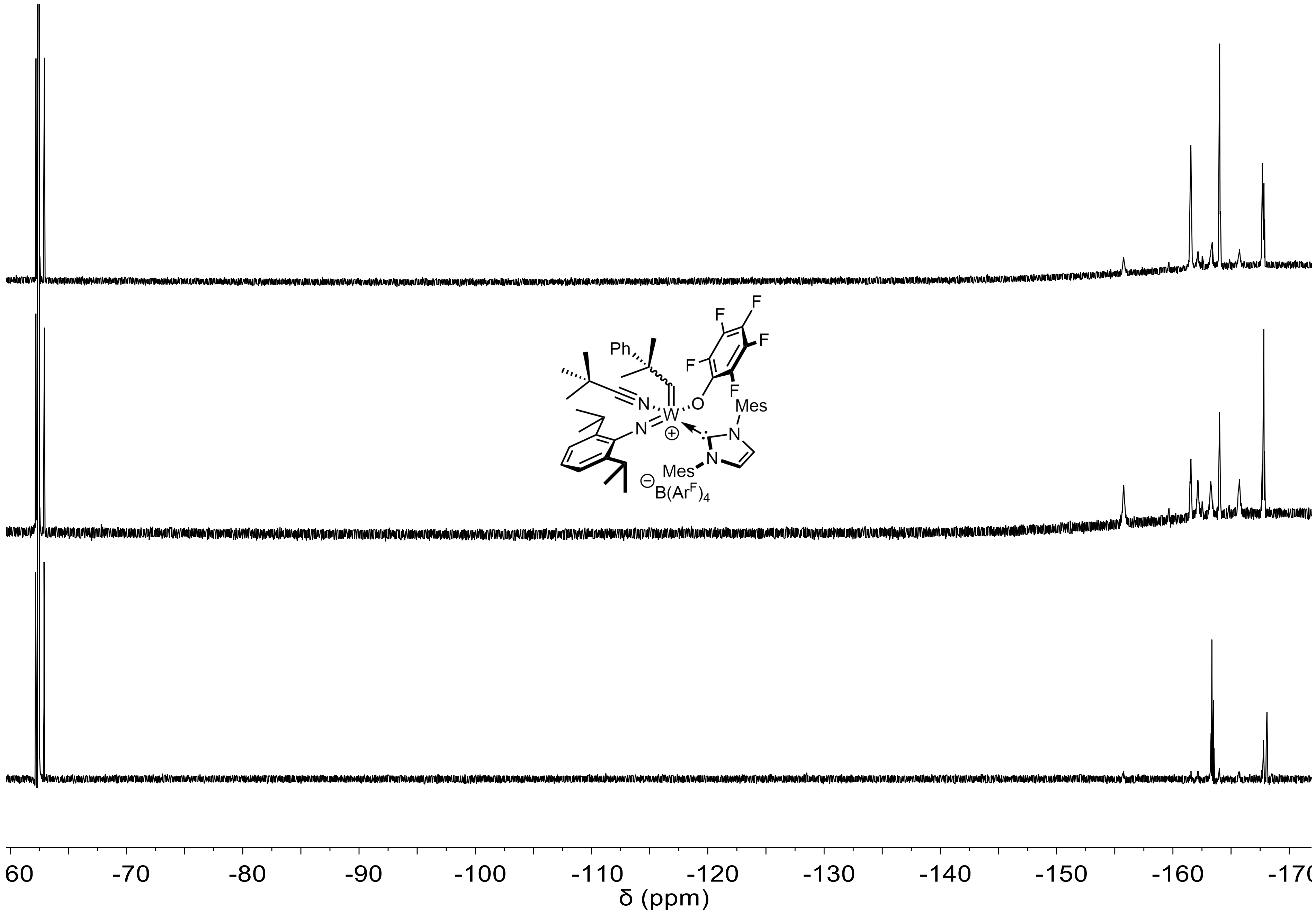

Figure S 152: ^{19}F-NMR (376 MHz, 25 °C, $CDCl_3$) of W-30 (upper), W-30 after exposure to air overnight (middle) and W-30 after exposure to air for two weeks (lower).

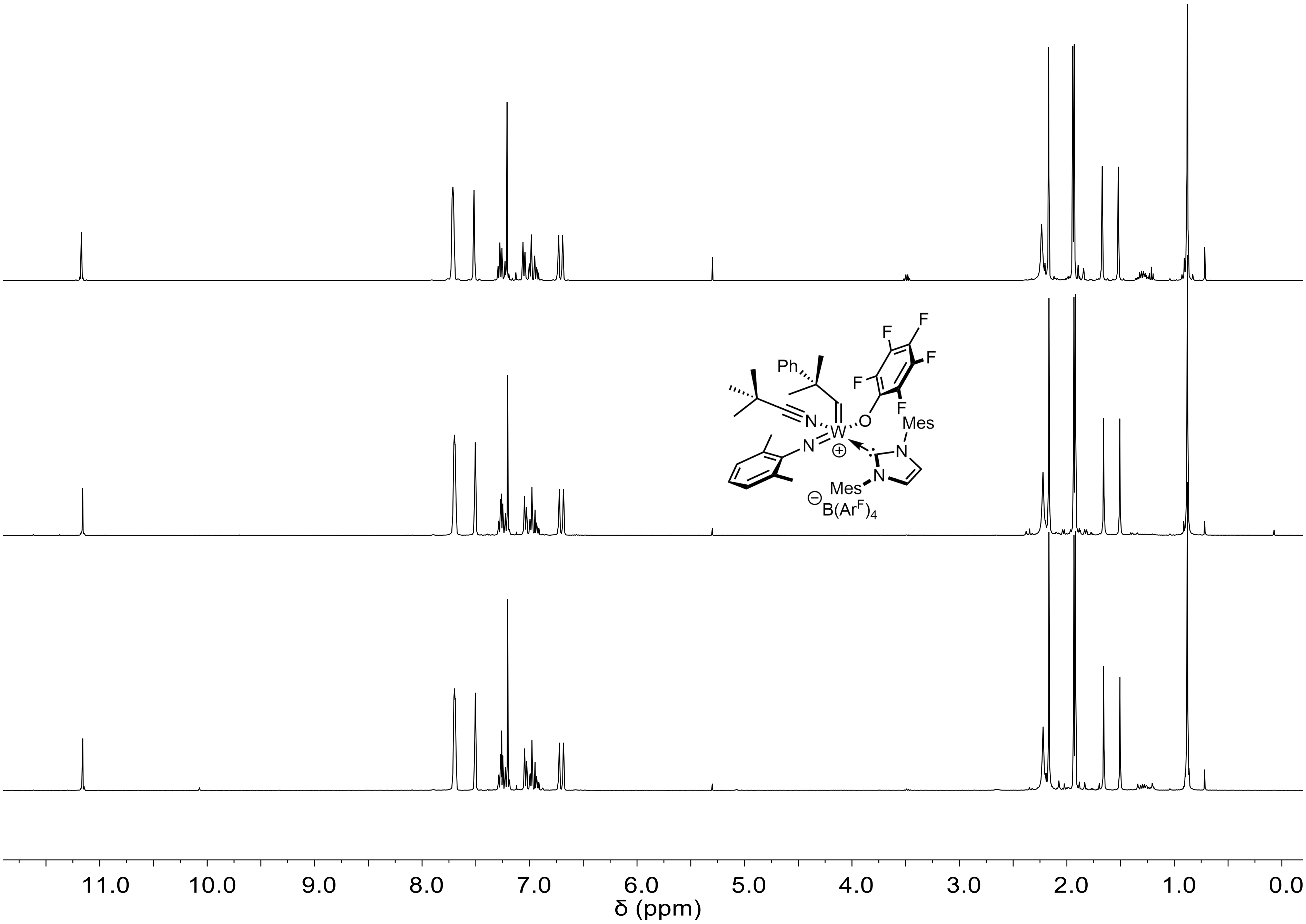

Figure S 153: ^{1}H-NMR (400 MHz, 25 °C, $CDCl_3$) of W-31 (upper), W-31 after exposure to air overnight (middle) and W-31 after exposure to air for two weeks (lower).

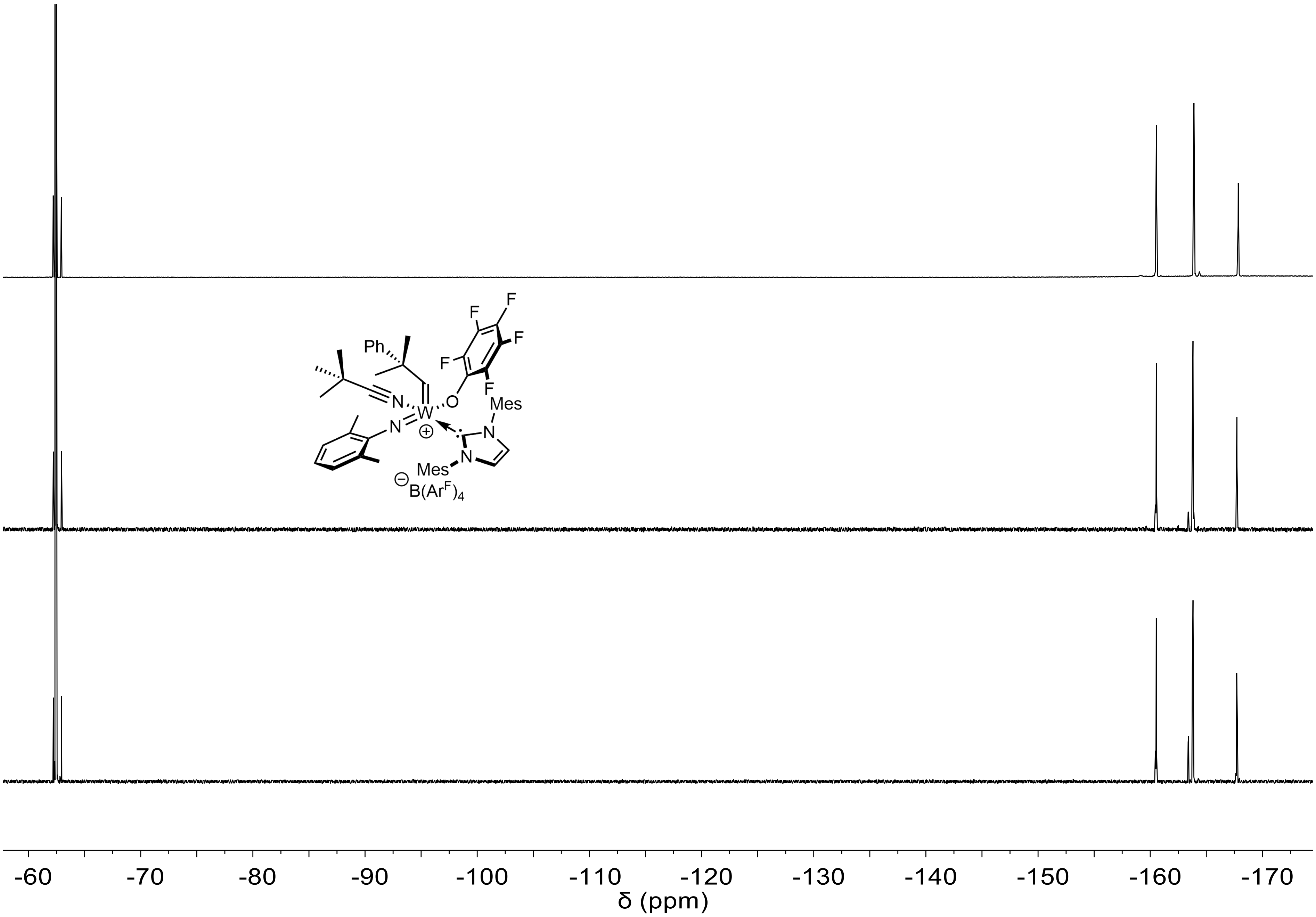

Figure S 154: ^{19}F-NMR (376 MHz, 25 °C, $CDCl_3$) of W-31 (upper), W-31 after exposure to air overnight (middle) and W-31 after exposure to air for two weeks (lower).

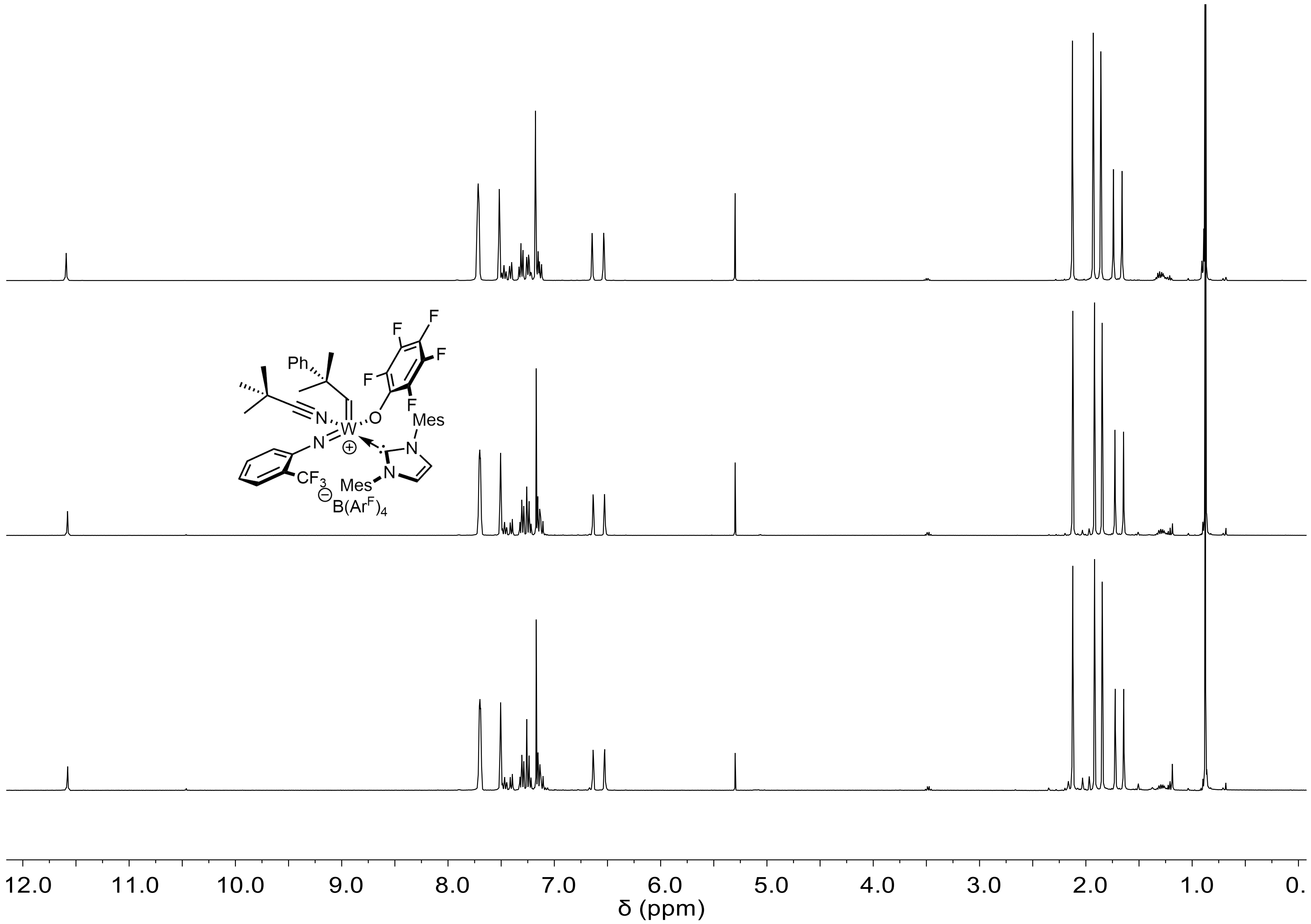

Figure S 155: ^{1}H-NMR (400 MHz, 25 °C, $CDCl_3$) of W-32 (upper), W-32 after exposure to air overnight (middle) and W-32 after exposure to air for two weeks (lower).

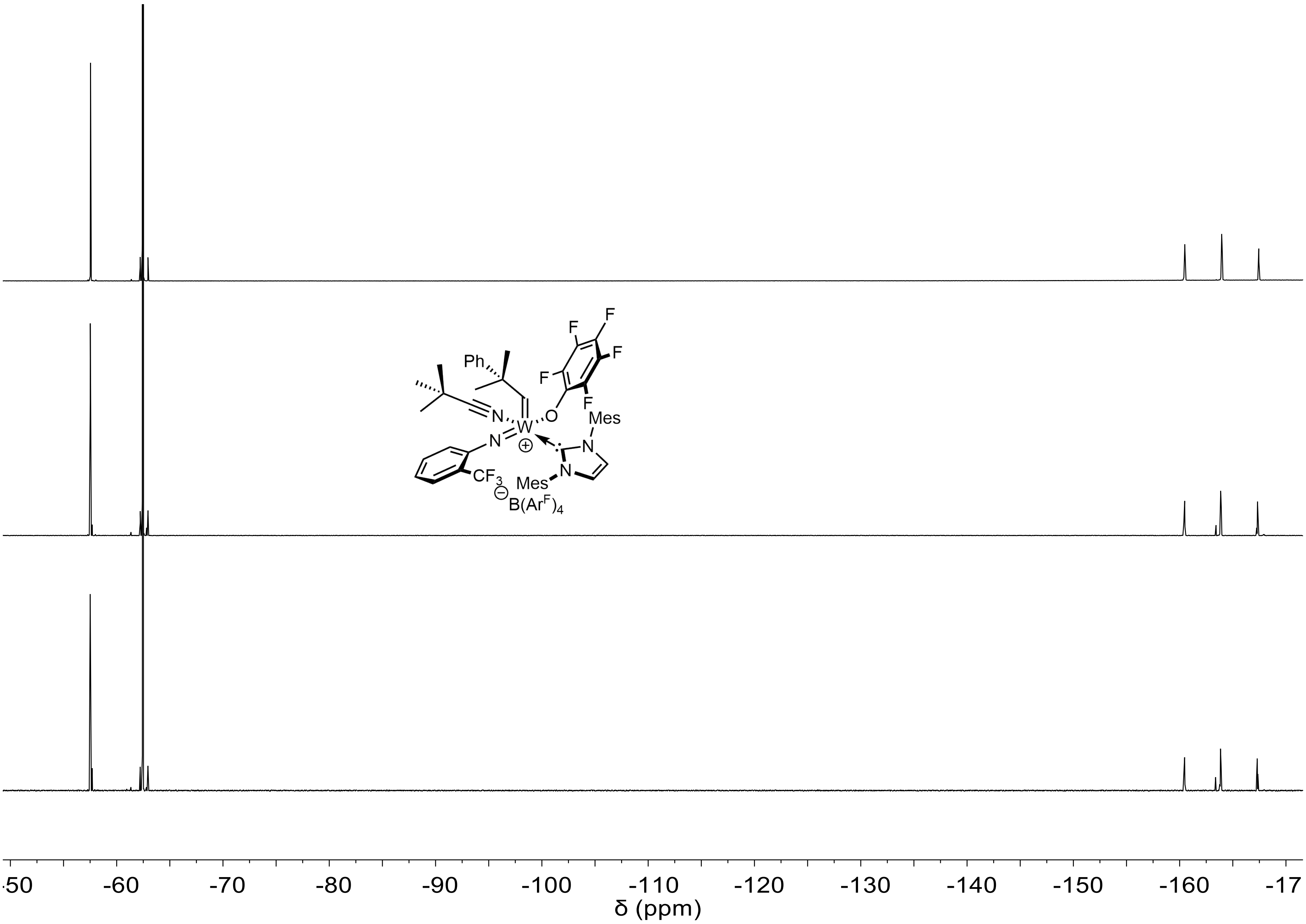

Figure S 156: ^{19}F-NMR (376 MHz, 25 °C, $CDCl_3$) of W-32 (upper), W-32 after exposure to air overnight (middle) and W-32 after exposure to air for two weeks (lower).

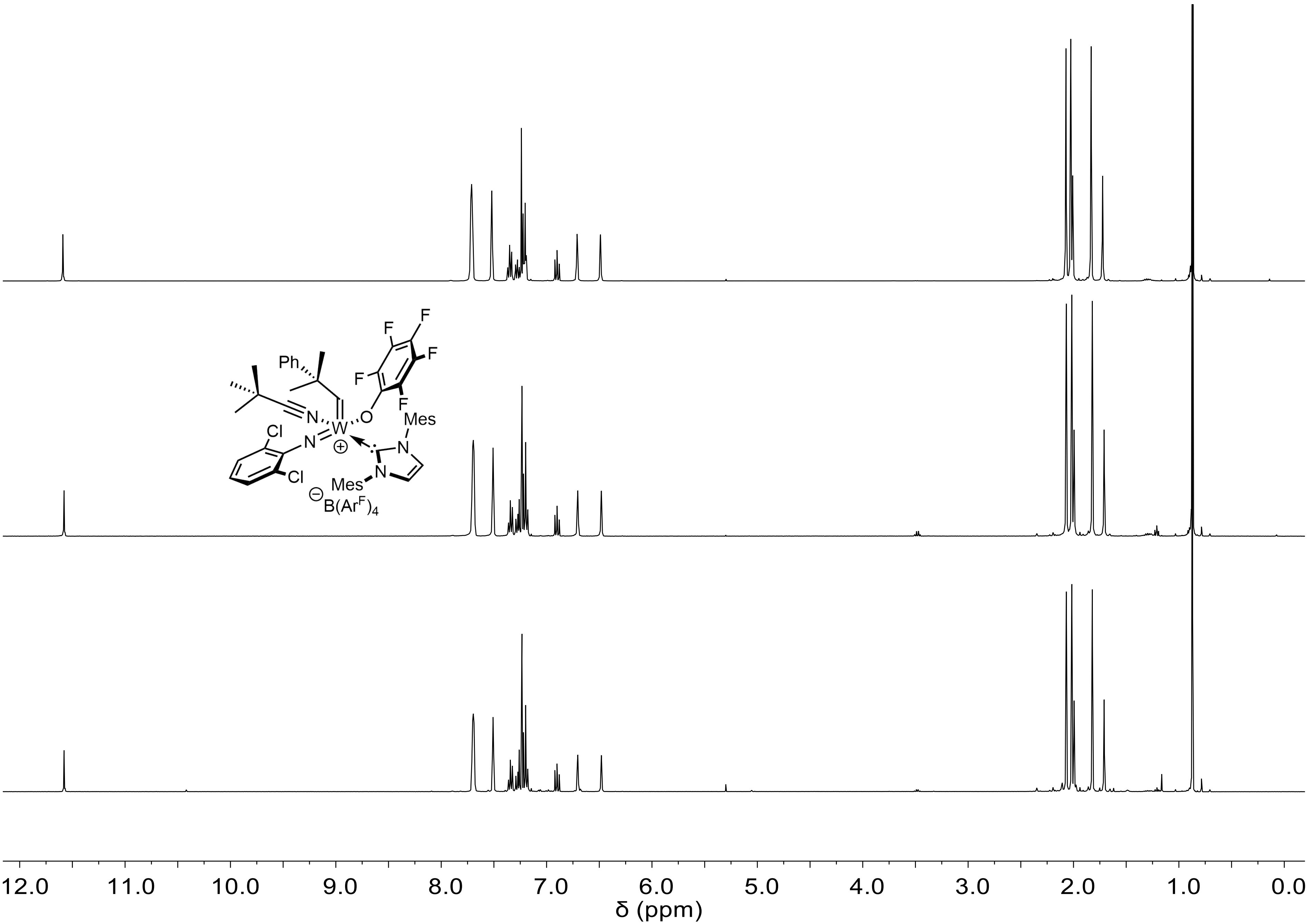

Figure S 157: ^{1}H-NMR (400 MHz, 25 °C, CDCl$_3$) of W-33 (upper), W-33 after exposure to air overnight (middle) and W-33 after exposure to air for two weeks (lower).

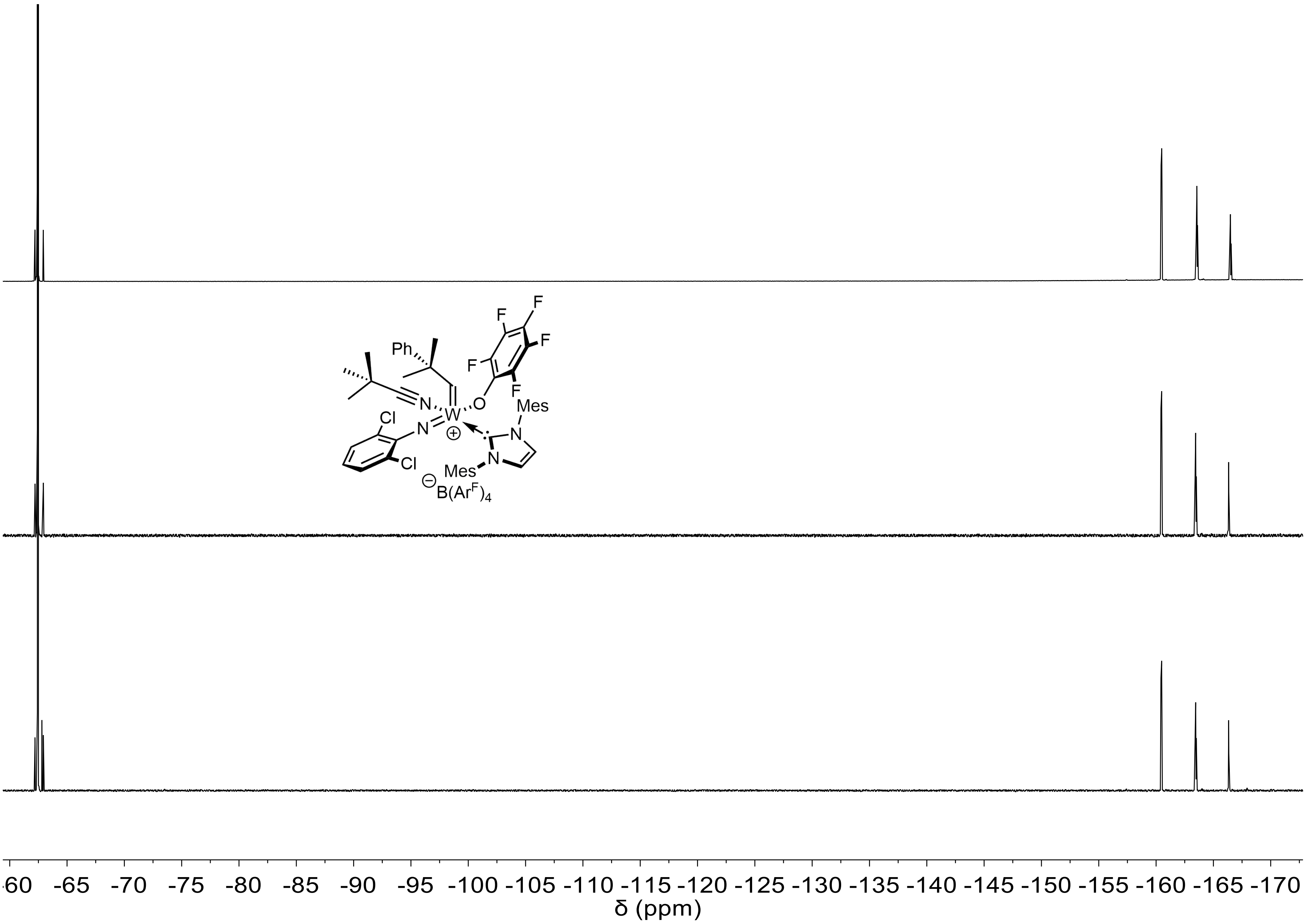

Figure S 158: ^{19}F-NMR (376 MHz, 25 °C, $CDCl_3$) of W-33 (upper), W-33 after exposure to air overnight (middle) and W-33 after exposure to air for two weeks (lower).

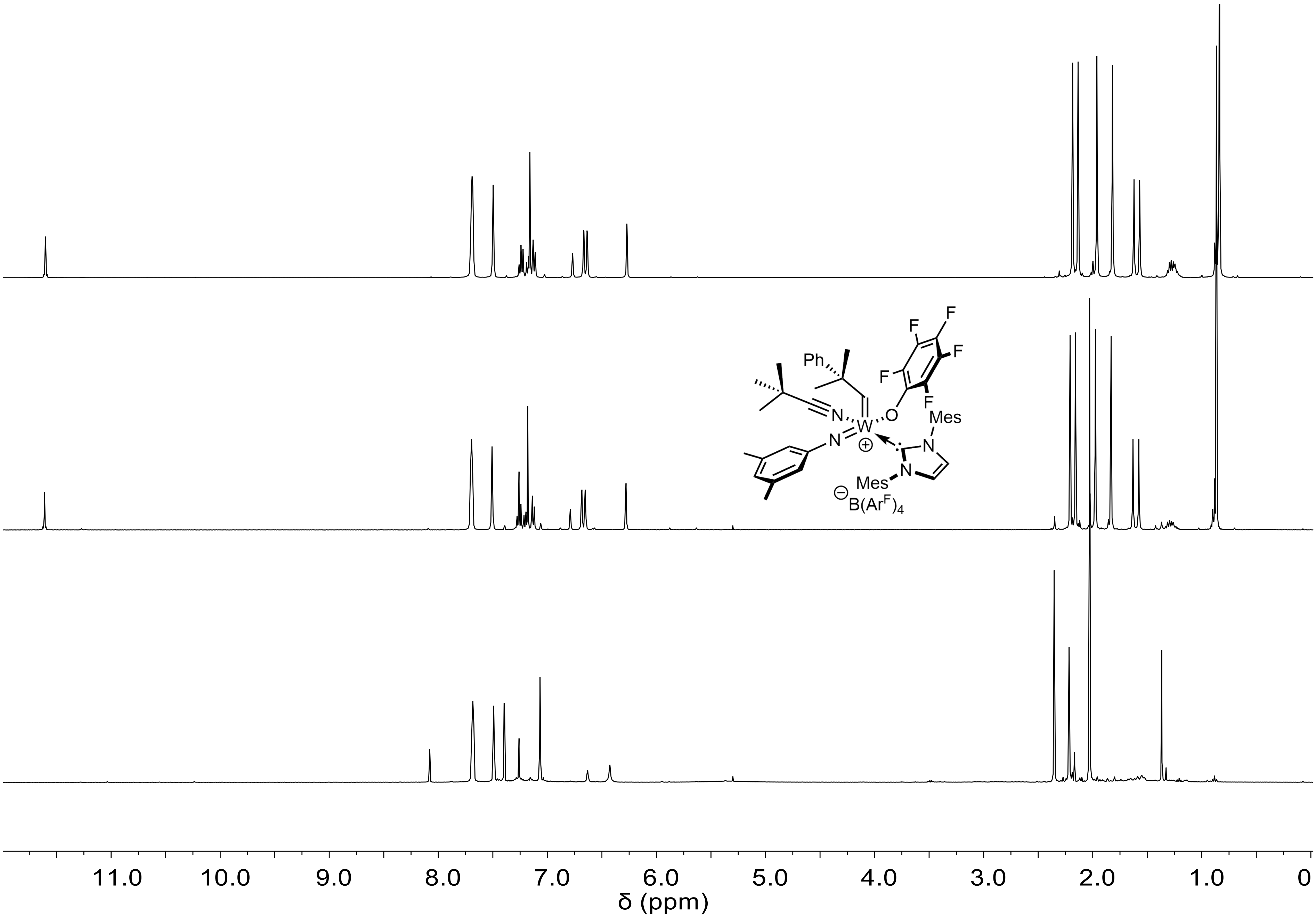

Figure S 159: ¹H-NMR (400 MHz, 25 °C, $CDCl_3$) of W-34 (upper), W-34 after exposure to air overnight (middle) and W-34 after exposure to air for two weeks (lower).

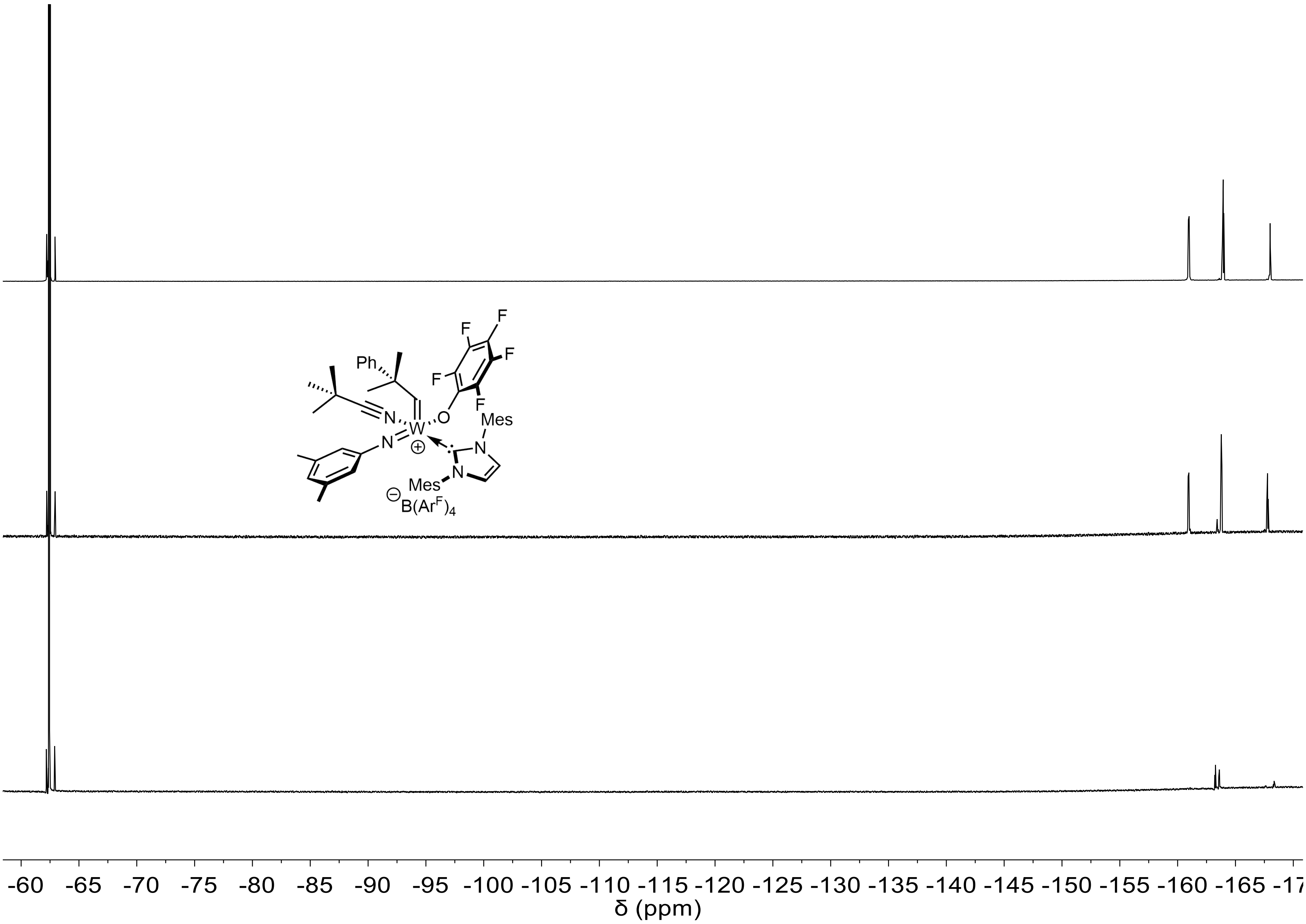

Figure S 160: ^{19}F-NMR (376 MHz, 25 °C, $CDCl_3$) of W-34 (upper), W-34 after exposure to air overnight (middle) and W-34 after exposure to air for two weeks (lower).

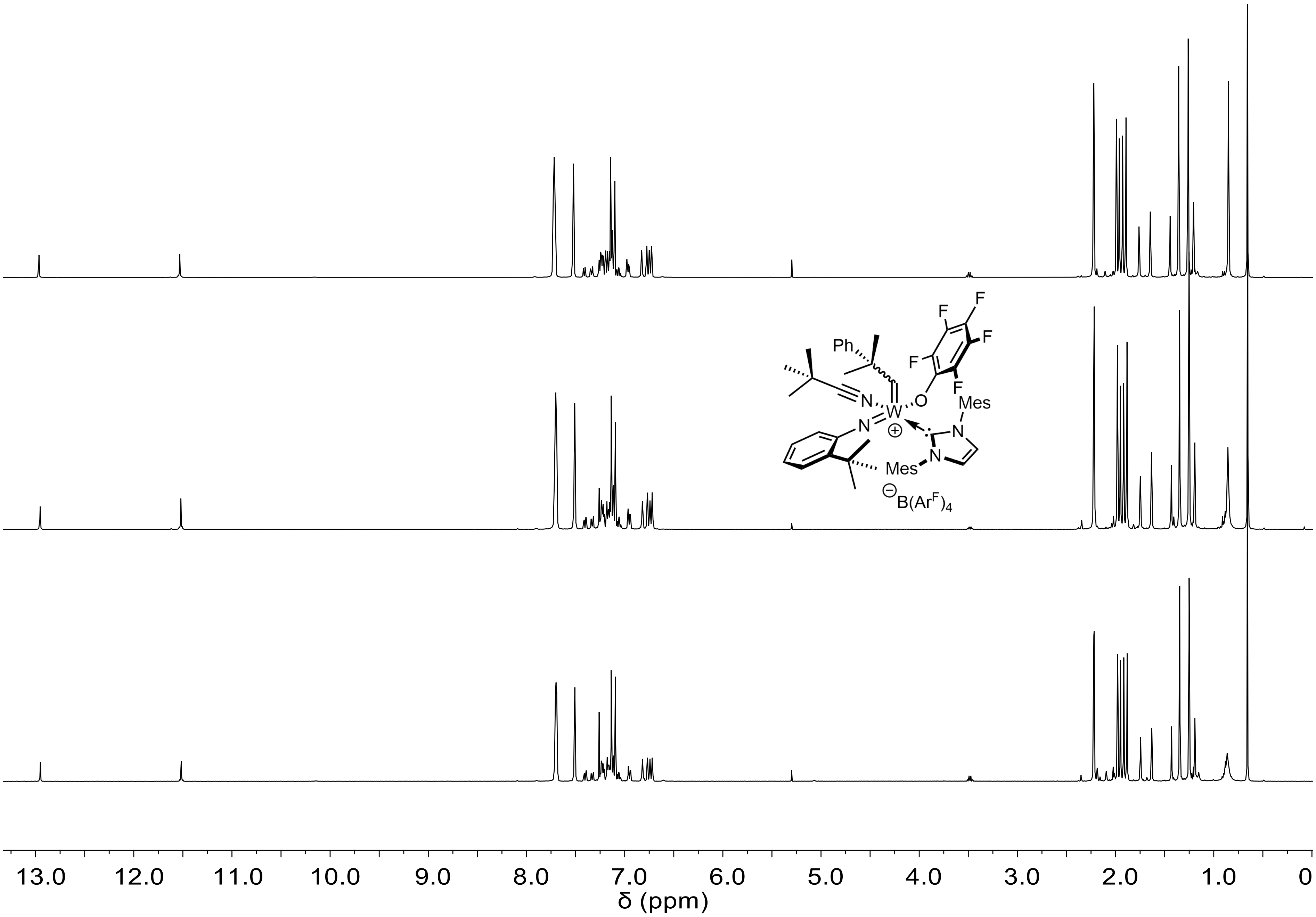

Figure S 161: ^{1}H-NMR (400 MHz, 25 °C, $CDCl_3$) of W-35 (upper), W-35 after exposure to air overnight (middle) and W-35 after exposure to air for two weeks (lower).

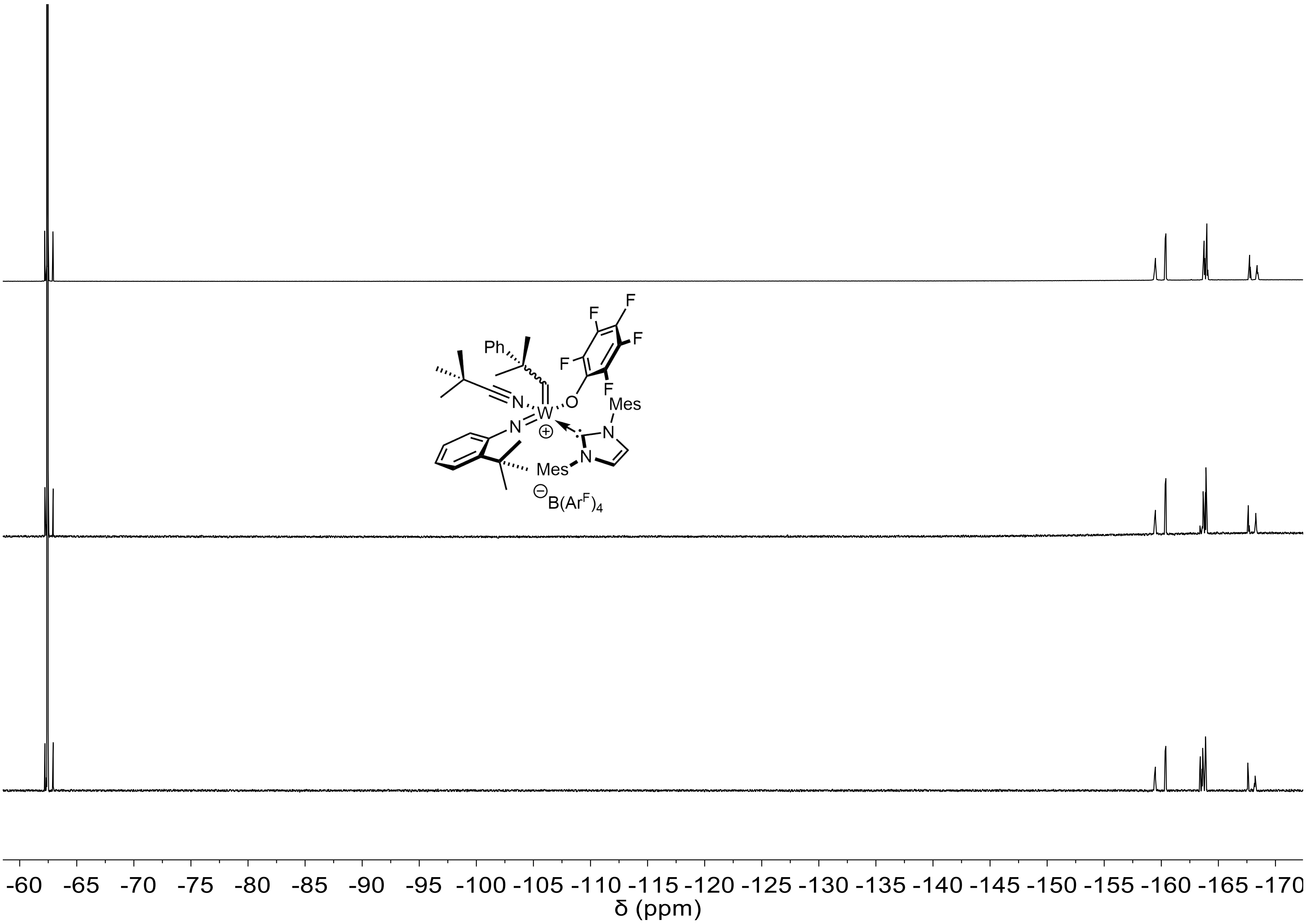

Figure S 162: ^{19}F-NMR (376 MHz, 25 °C, $CDCl_3$) of W-35 (upper), W-35 after exposure to air overnight (middle) and W-35 after exposure to air for two weeks (lower).

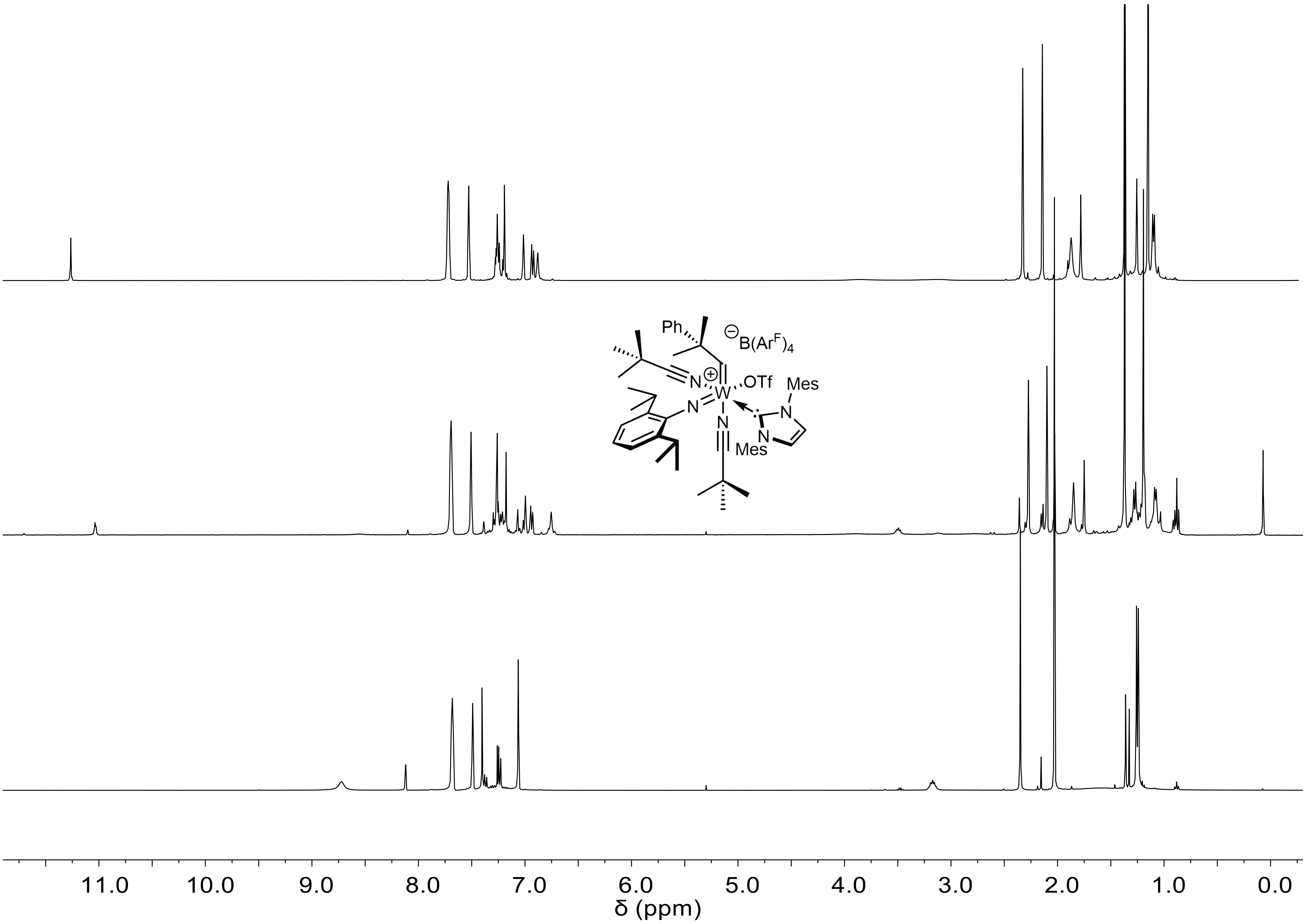

Figure S 163: ^{1}H-NMR (400 MHz, 25 °C, $CDCl_3$) of W-36 (upper), W-36 after exposure to air overnight (middle) and W-36 after exposure to air for two weeks (lower).

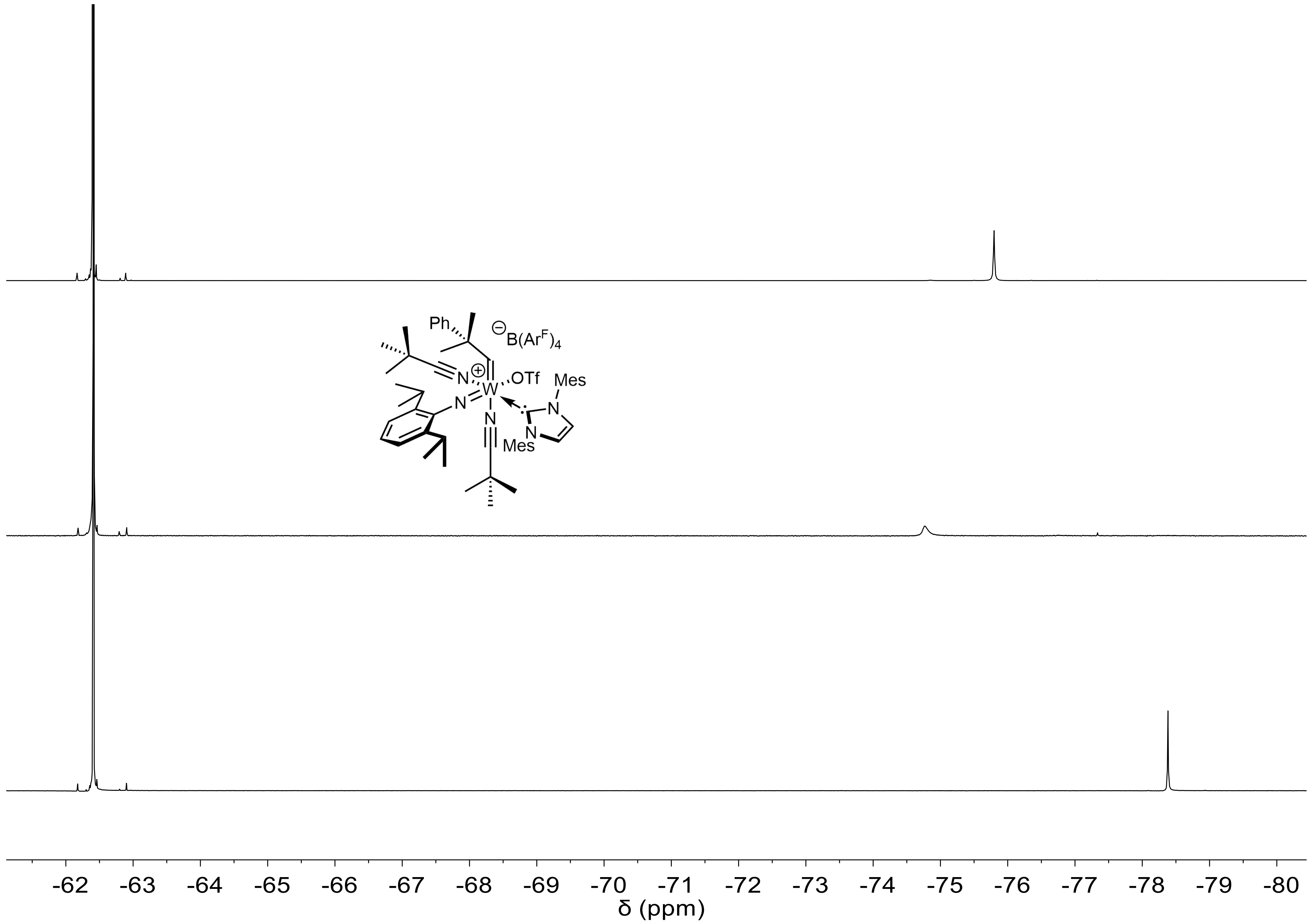

Figure S 164: ^{19}F-NMR (376 MHz, 25 °C, $CDCl_3$) of W-36 (upper), W-36 after exposure to air overnight (middle) and W-36 after exposure to air for two weeks (lower).

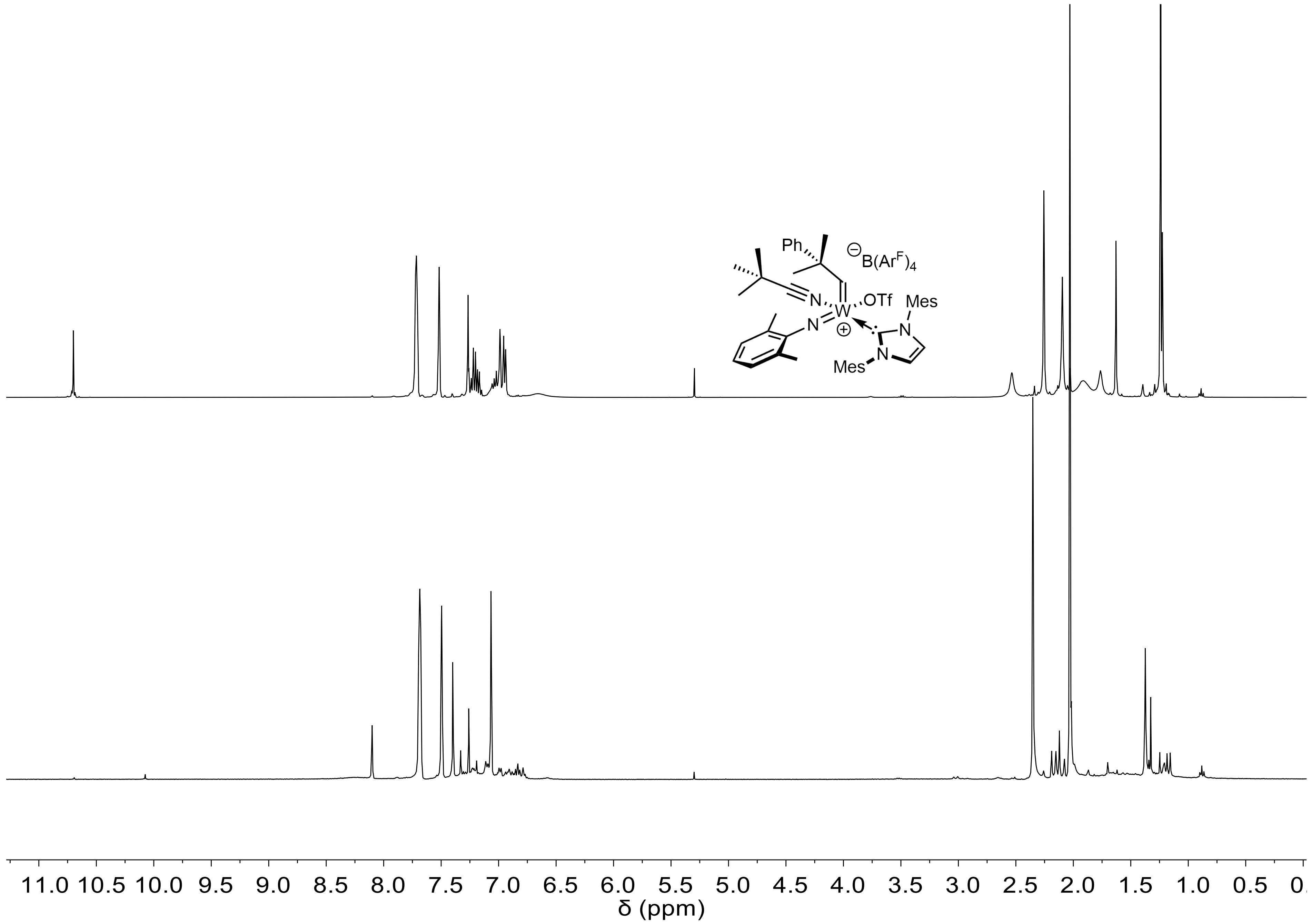

Figure S 165: ^{1}H-NMR (400 MHz, 25 °C, $CDCl_3$) of W-37 (upper) and W-37 after exposure to air for two weeks (lower).

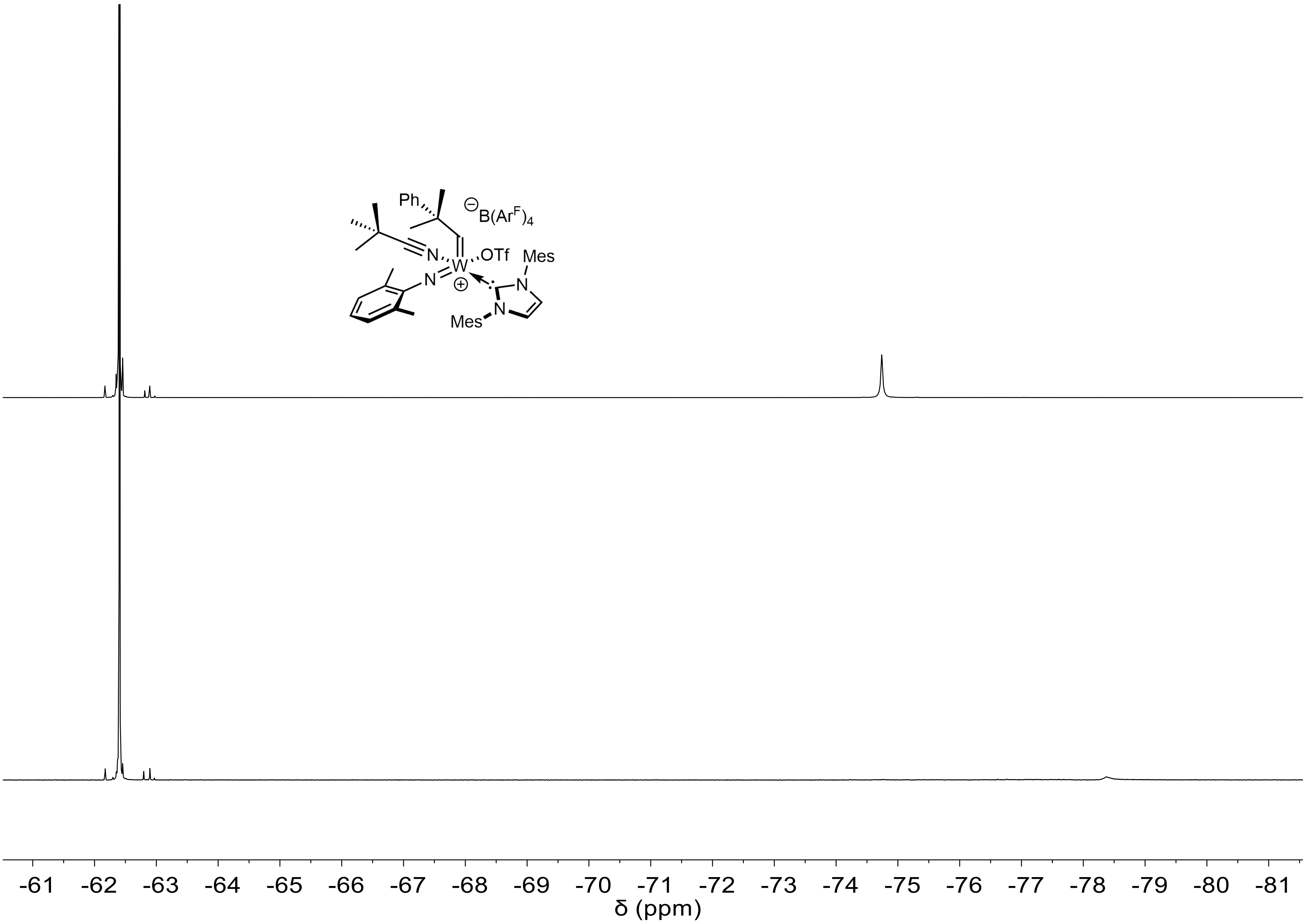

Figure S 166: ^{19}F-NMR (376 MHz, 25 °C, CDCl$_3$) of W-37 (upper) and W-37 after exposure to air for two weeks (lower).

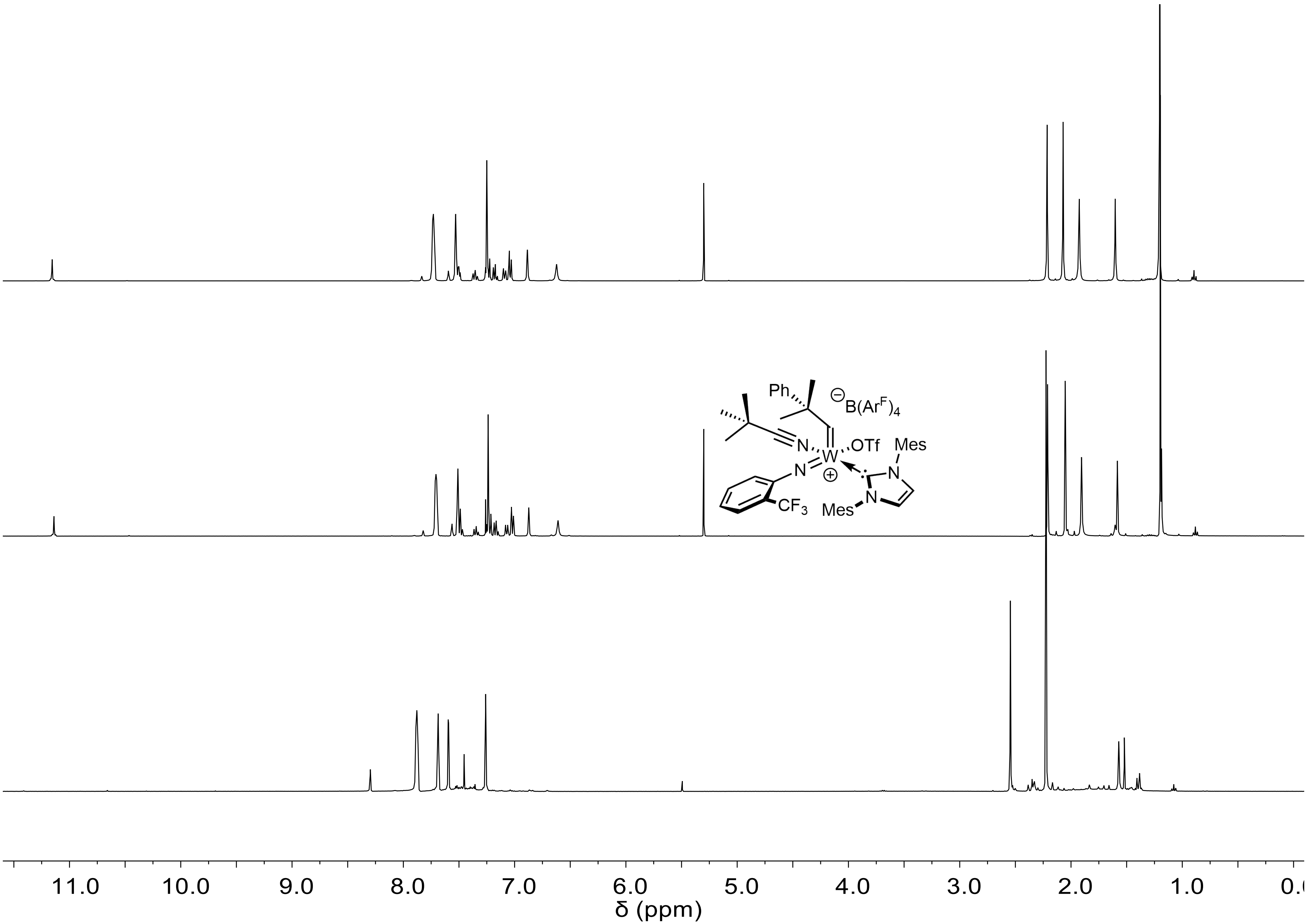

Figure S 167: ^{1}H-NMR (400 MHz, 25 °C, $CDCl_3$) of W-38 (upper), W-38 after exposure to air overnight (middle) and W-38 after exposure to air for two weeks (lower).

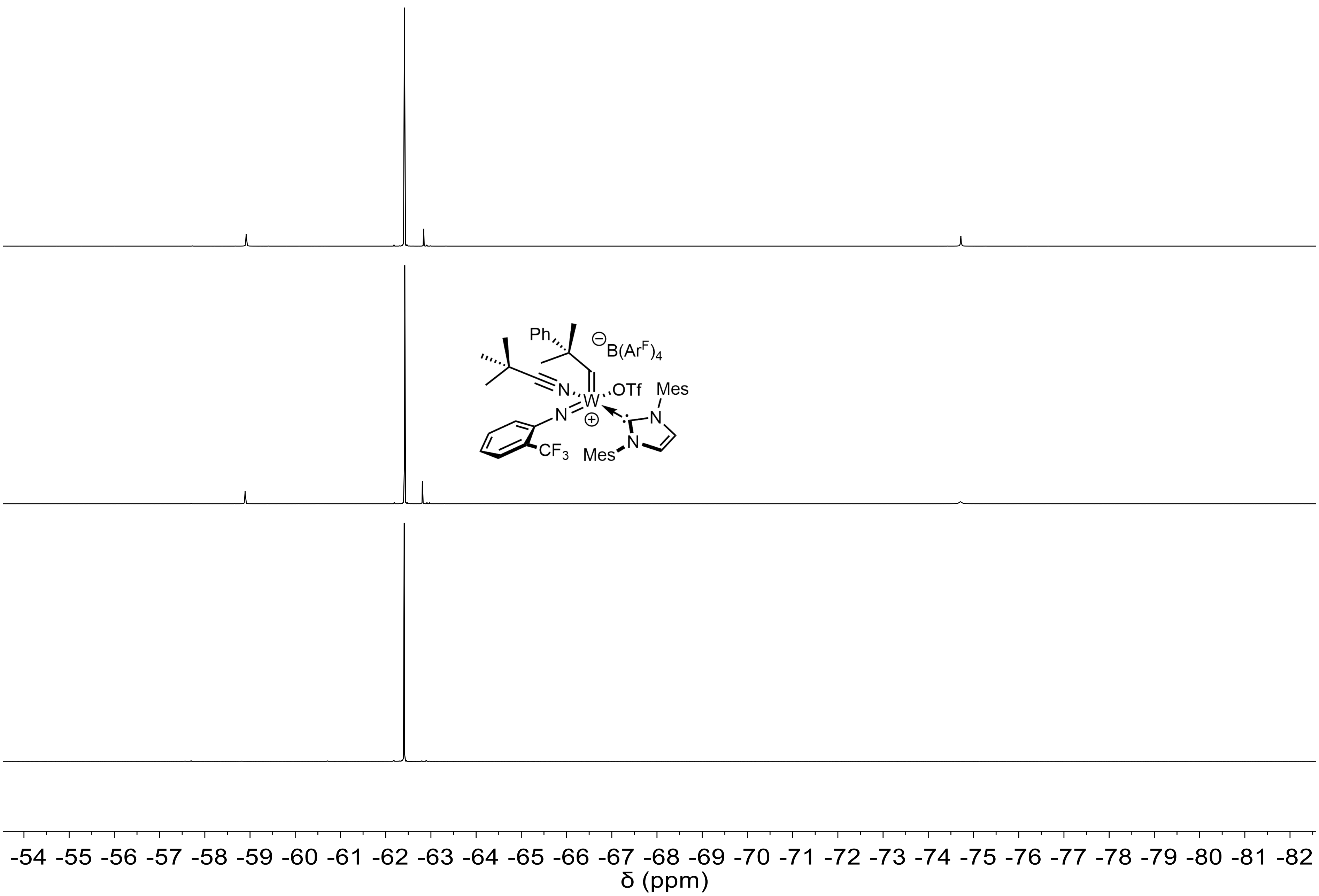

Figure S 168: ^{19}F-NMR (376 MHz, 25 °C, $CDCl_3$) of W-38 (upper), W-38 after exposure to air overnight (middle) and W-38 after exposure to air for two weeks (lower).

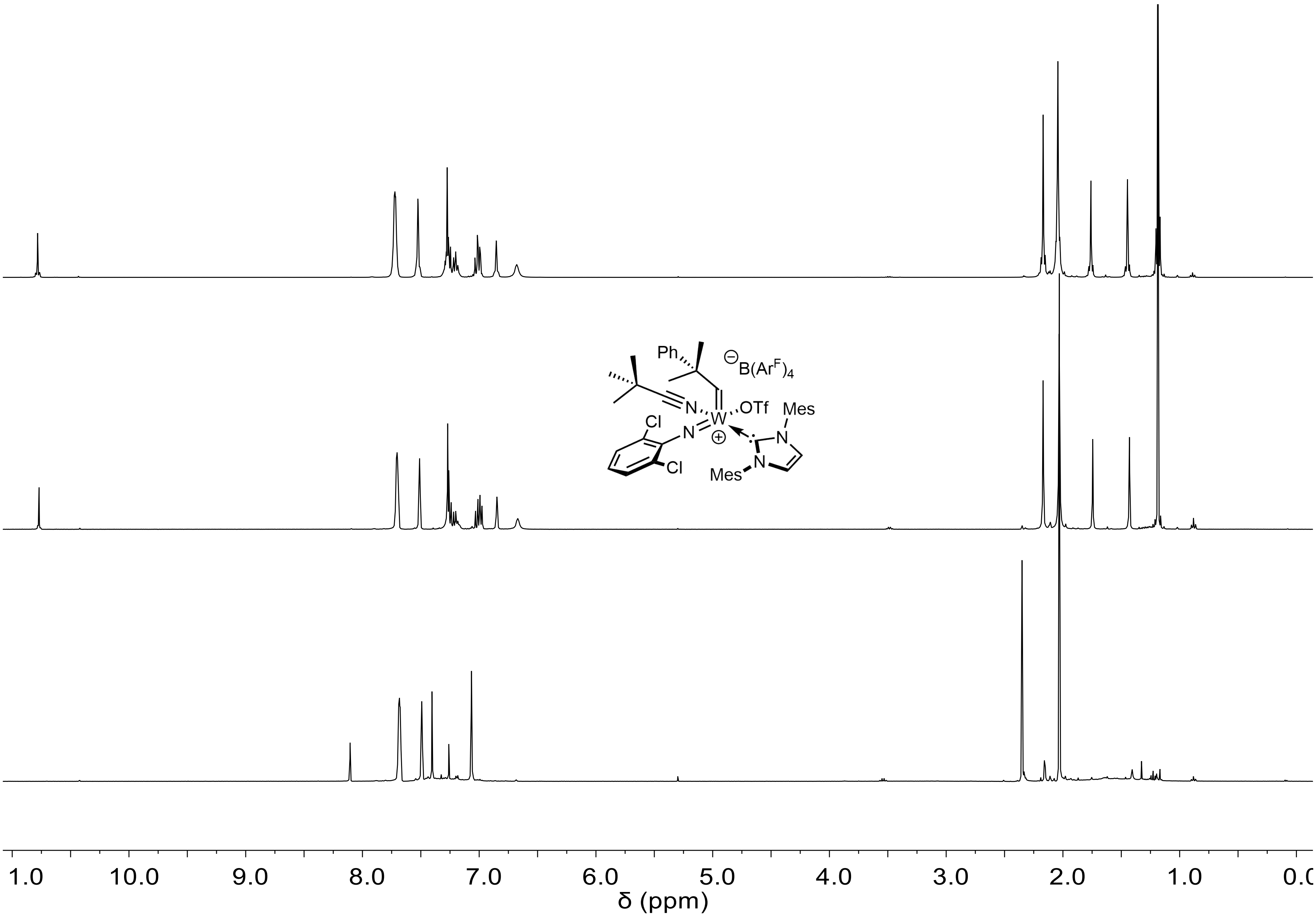

Figure S 169: ^{1}H-NMR (400 MHz, 25 °C, $CDCl_3$) of W-39 (upper), W-39 after exposure to air overnight (middle) and W-39 after exposure to air for two weeks (lower).

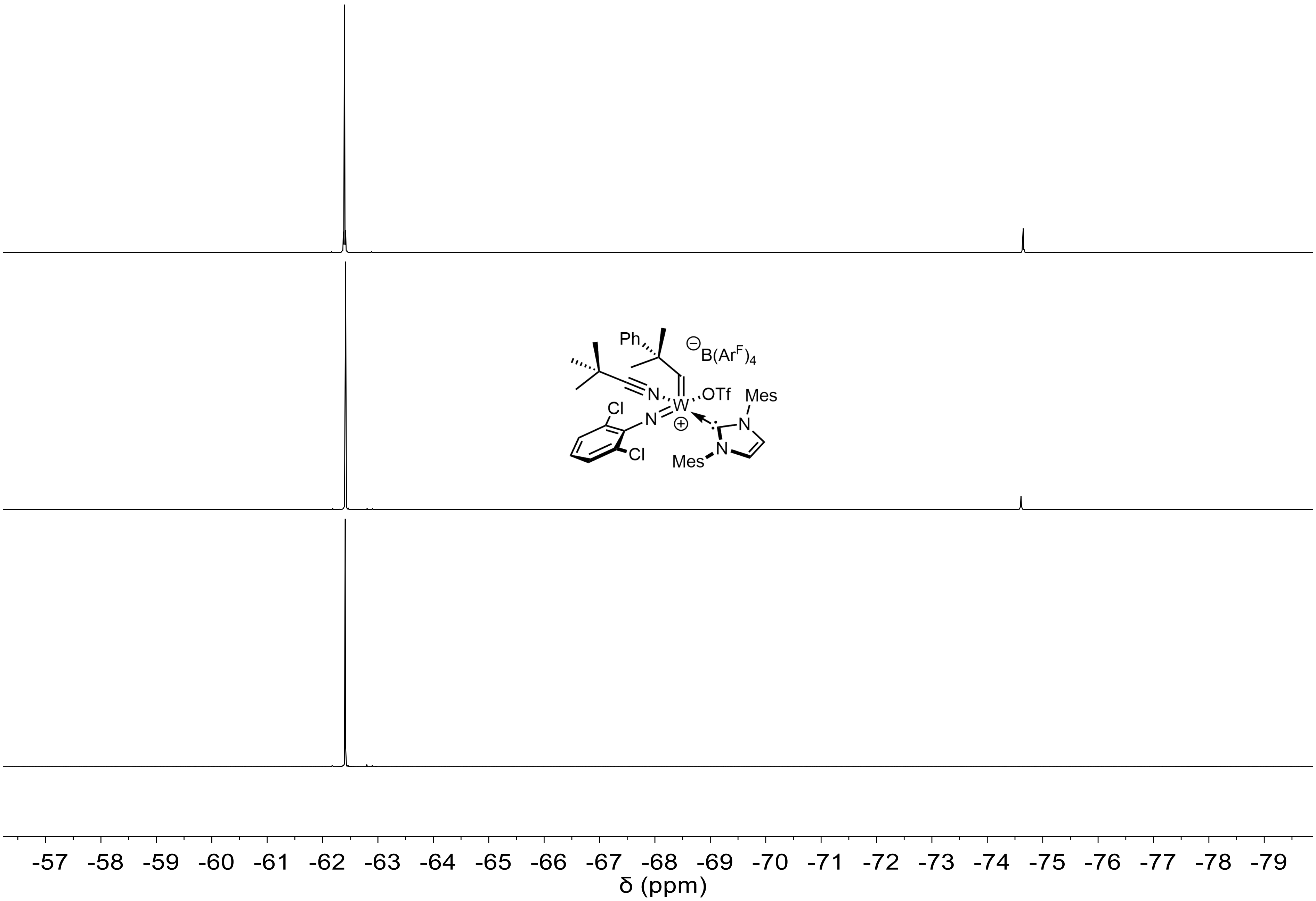

Figure S 170: ^{19}F-NMR (376 MHz, 25 °C, $CDCl_3$) of W-39 (upper), W-39 after exposure to air overnight (middle) and W-39 after exposure to air for two weeks (lower).

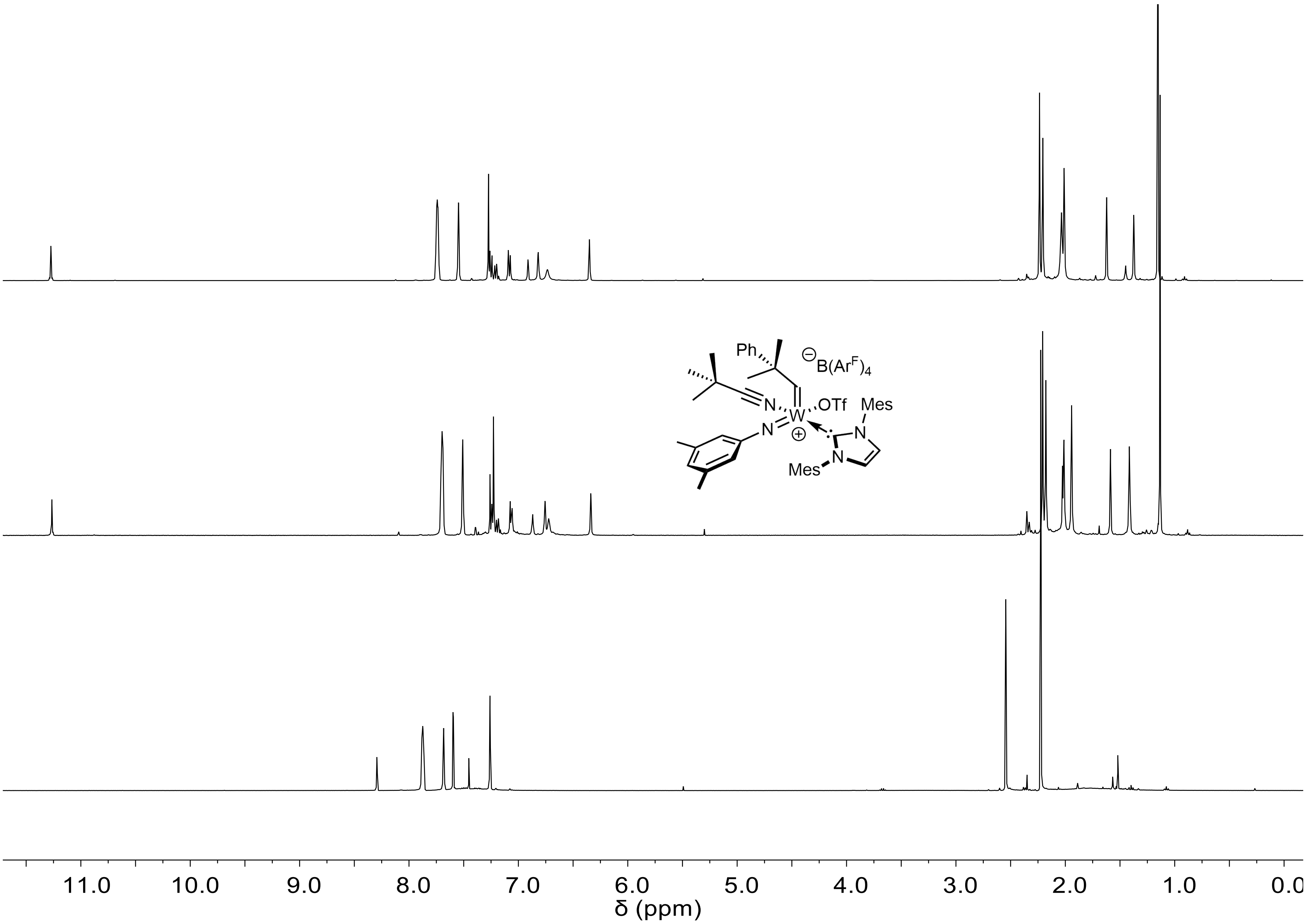

Figure S 171: ^{1}H-NMR (400 MHz, 25 °C, $CDCl_3$) of W-40 (upper), W-40 after exposure to air overnight (middle) and W-40 after exposure to air for two weeks (lower).

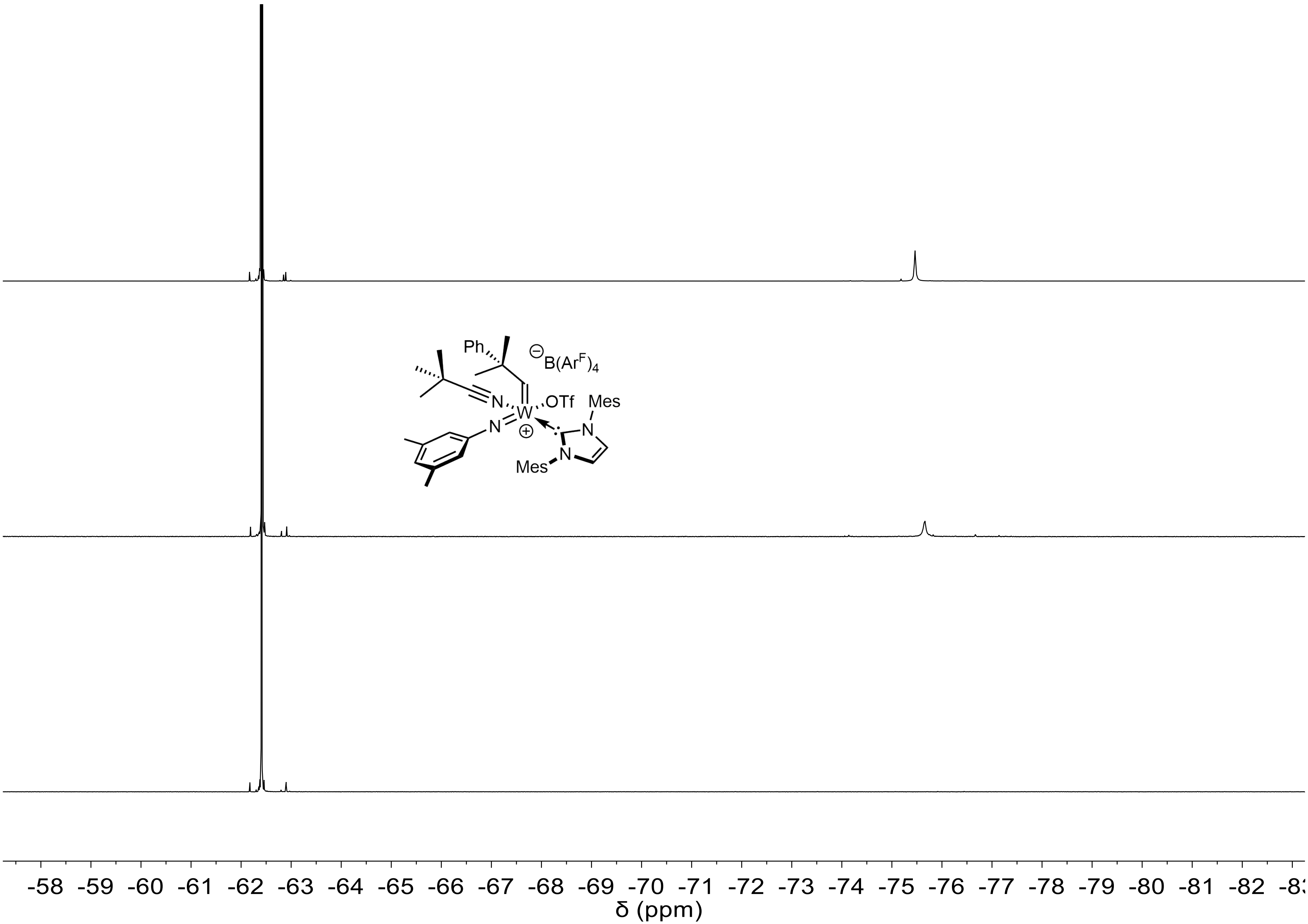

Figure S 172: ^{19}F-NMR (376 MHz, 25 °C, $CDCl_3$) of W-40 (upper), W-40 after exposure to air overnight (middle) and W-40 after exposure to air for two weeks (lower).

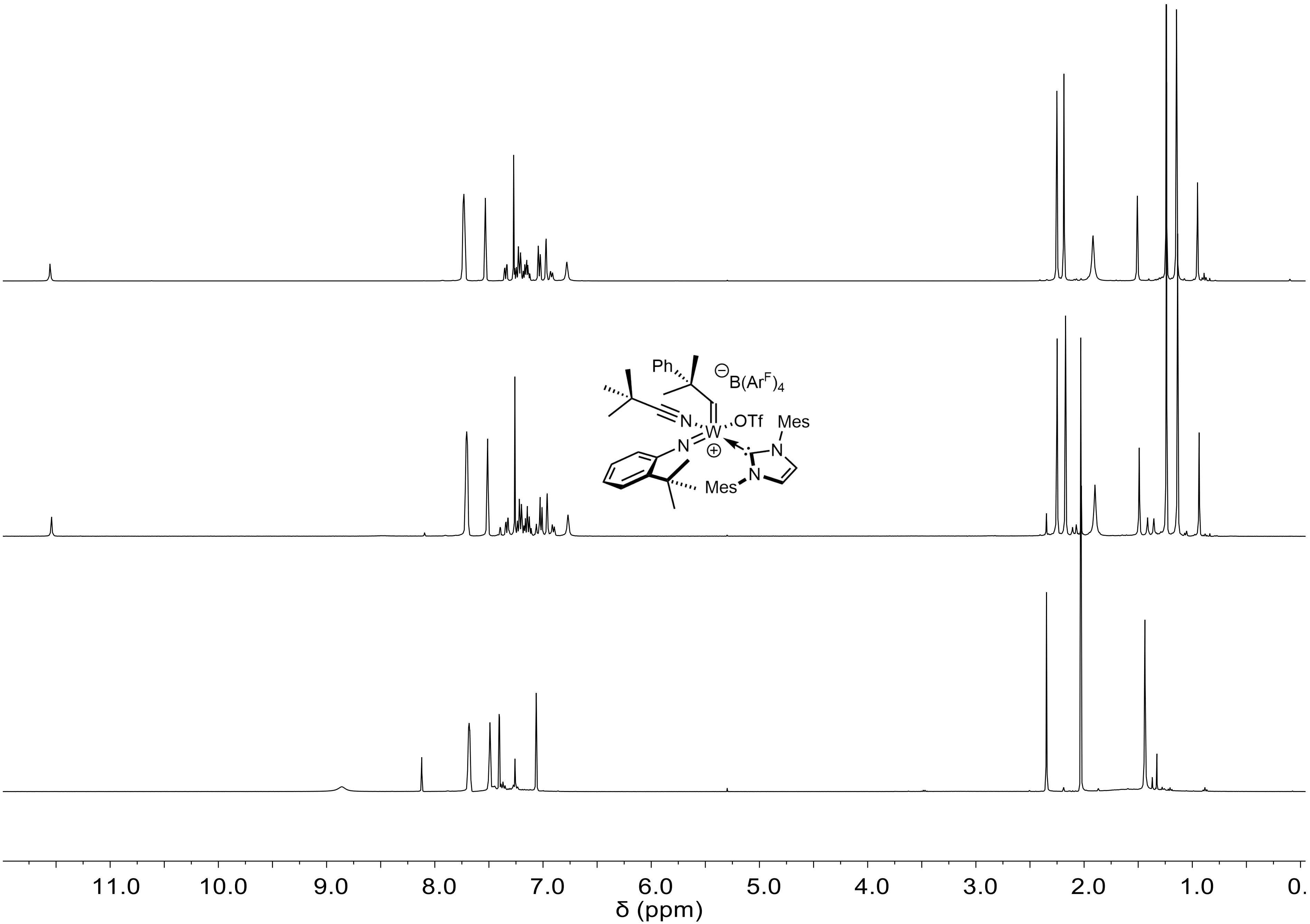

Figure S 173: ^{1}H-NMR (400 MHz, 25 °C, $CDCl_3$) of W-41 (upper), W-41 after exposure to air overnight (middle) and W-41 after exposure to air for two weeks (lower).

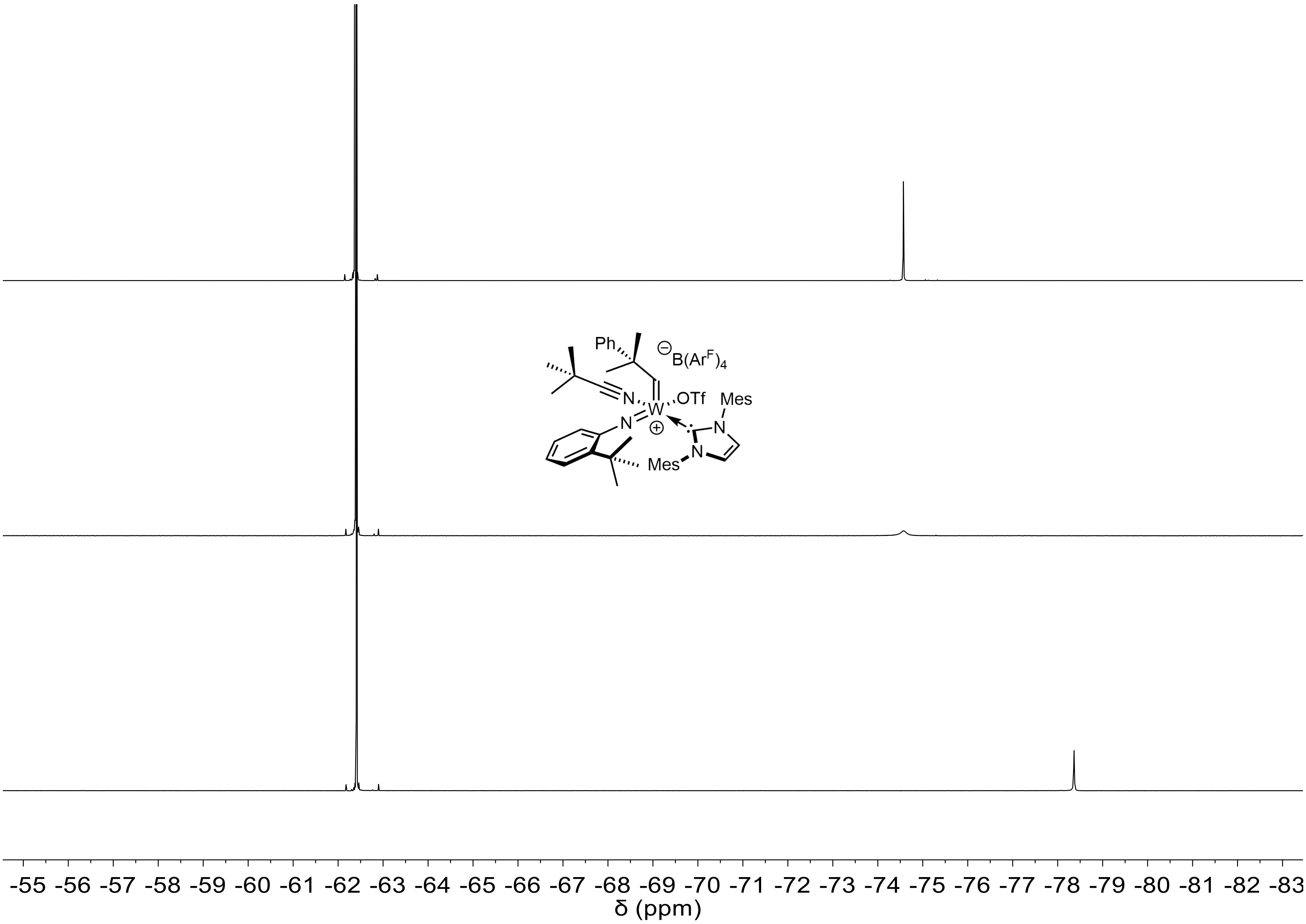

Figure S 174: ^{19}F-NMR (376 MHz, 25 °C, $CDCl_3$) of W-41 (upper), W-41 after exposure to air overnight (middle) and W-41 after exposure to air for two weeks (lower).

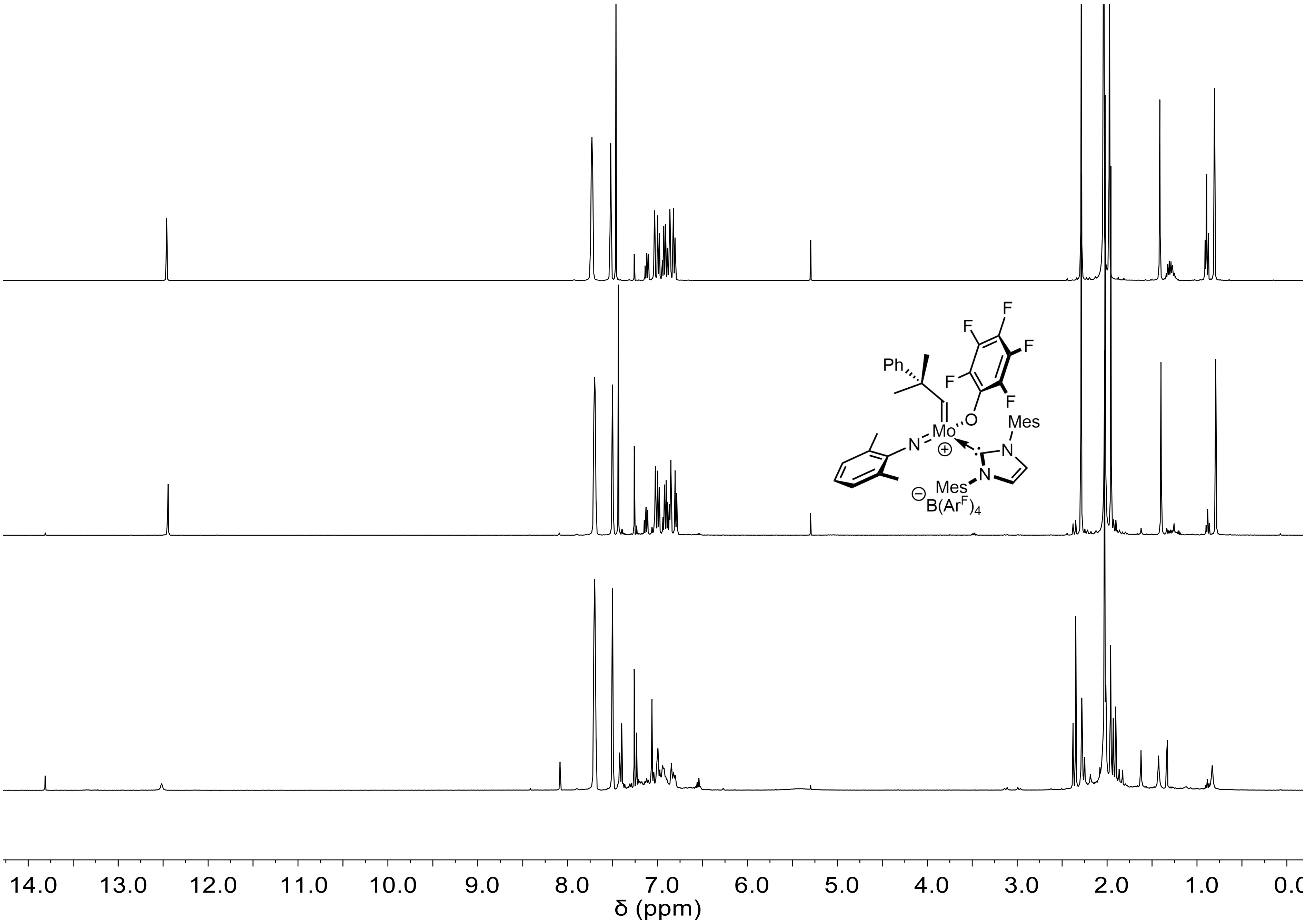

Figure S 175: ^{1}H-NMR (400 MHz, 25 °C, $CDCl_3$) of Mo-02 (upper), Mo-02 after exposure to air overnight (middle) and Mo-02 after exposure to air for nine days (lower).

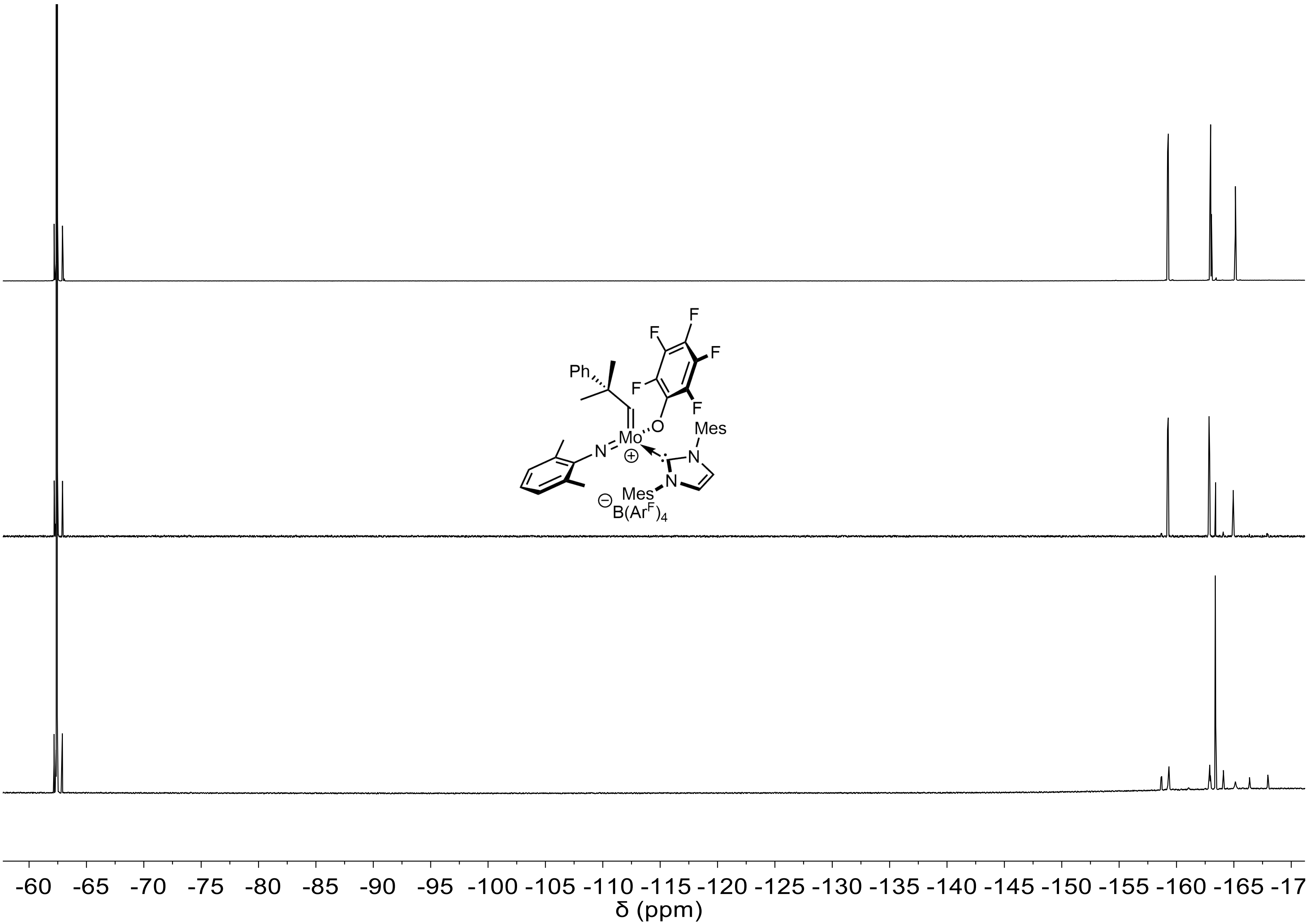

Figure S 176: ^{19}F-NMR (376 MHz, 25 °C, $CDCl_3$) of Mo-02 (upper), Mo-02 after exposure to air overnight (middle) and Mo-02 after exposure to air for nine days (lower).

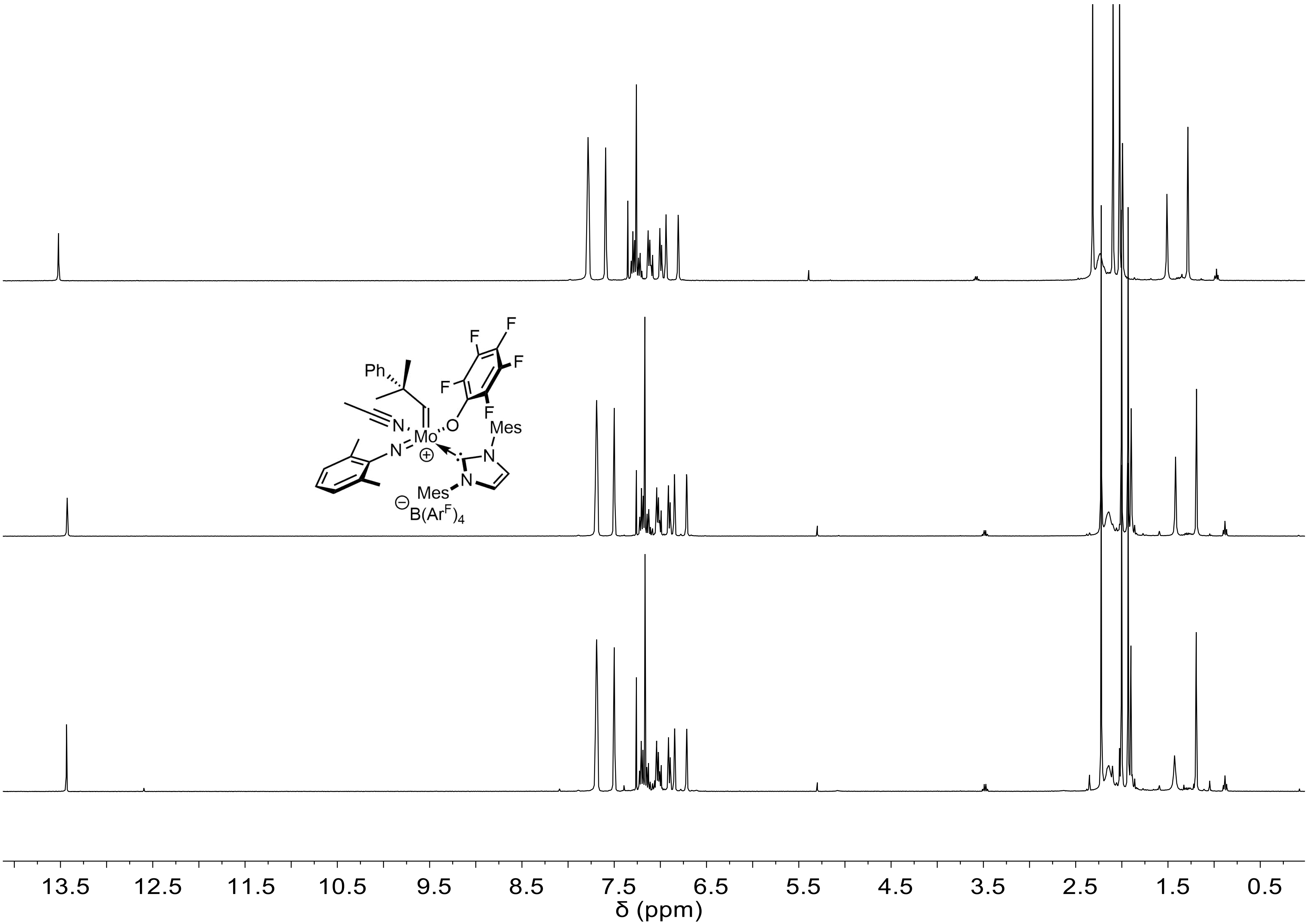

Figure S 177: ^{1}H-NMR (400 MHz, 25 °C, $CDCl_3$) of Mo-02-MeCN (upper), Mo-02-MeCN after exposure to air overnight (middle) and Mo-02-MeCN after exposure to air for two weeks (lower).

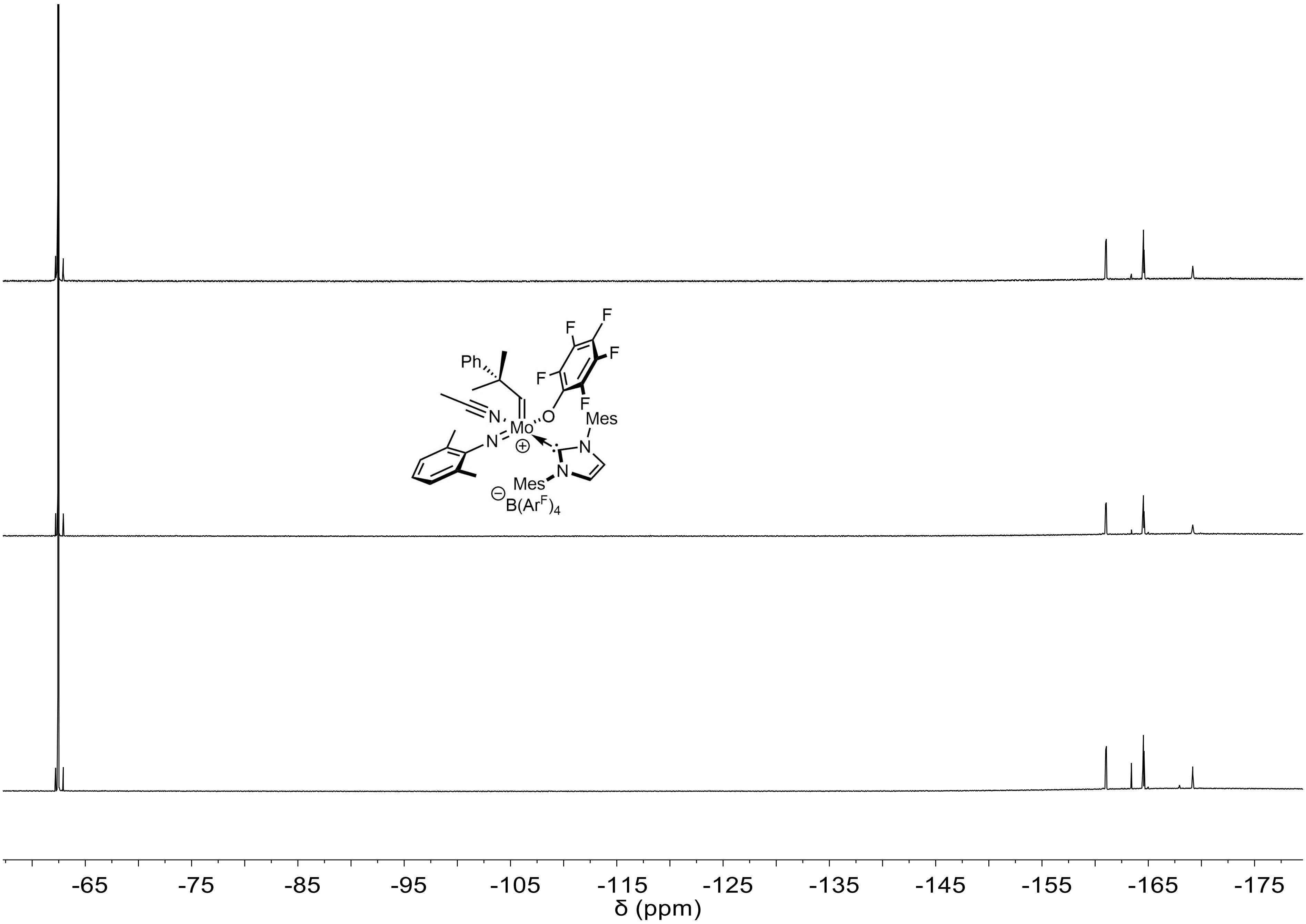

Figure S 178: ^{19}F-NMR (376 MHz, 25 °C, $CDCl_3$) of Mo-02-MeCN (upper), Mo-02-MeCN after exposure to air overnight (middle) and Mo-02-MeCN after exposure to air for two weeks (lower).

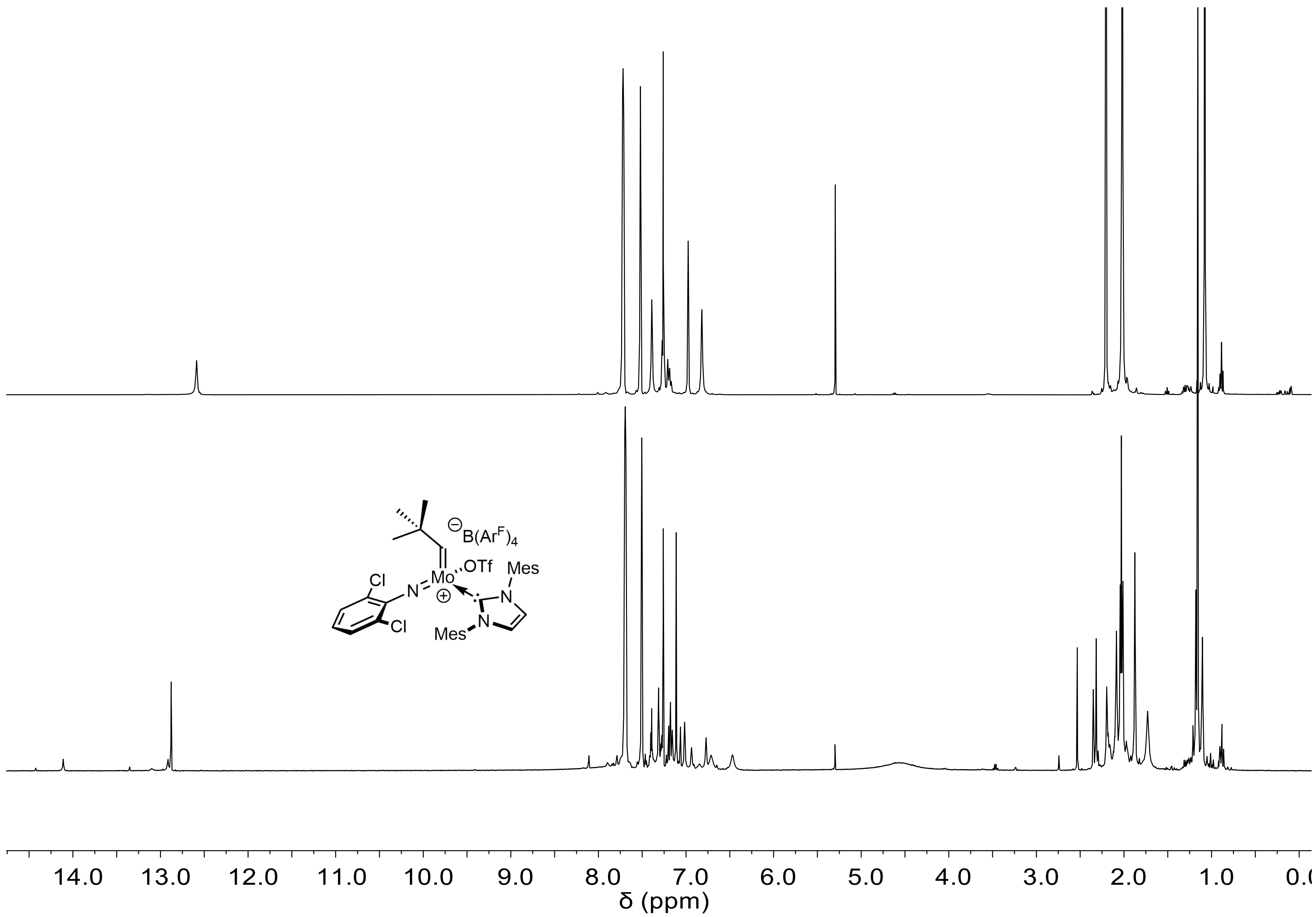

Figure S 179: ^{1}H-NMR (400 MHz, 25 °C, $CDCl_3$) of Mo-03 (upper) and Mo-03 after exposure to air overnight (lower).

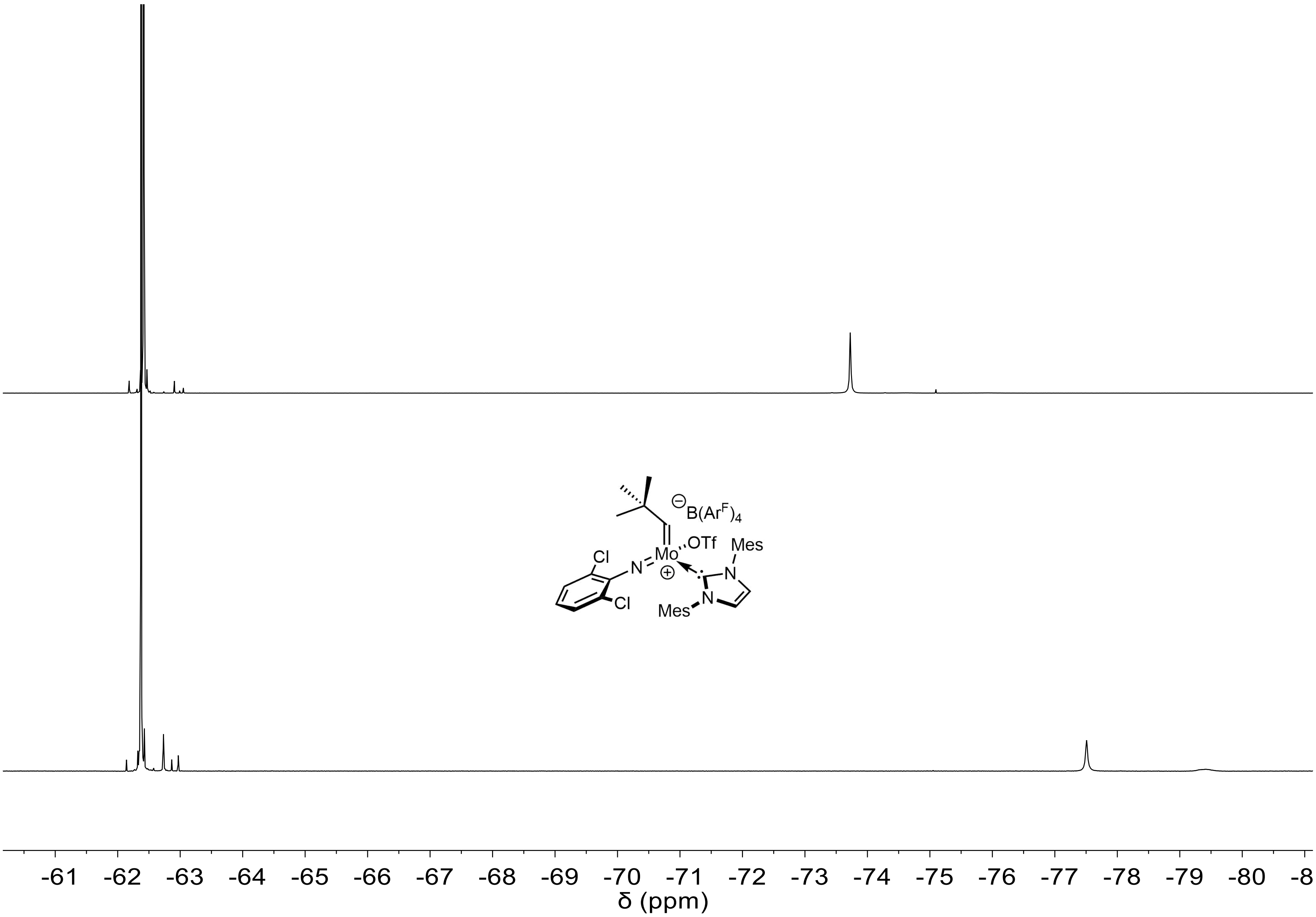

Figure S 180: ^{19}F-NMR (376 MHz, 25 °C, $CDCl_3$) of Mo-03 (upper) and Mo-03 after exposure to air overnight (lower).

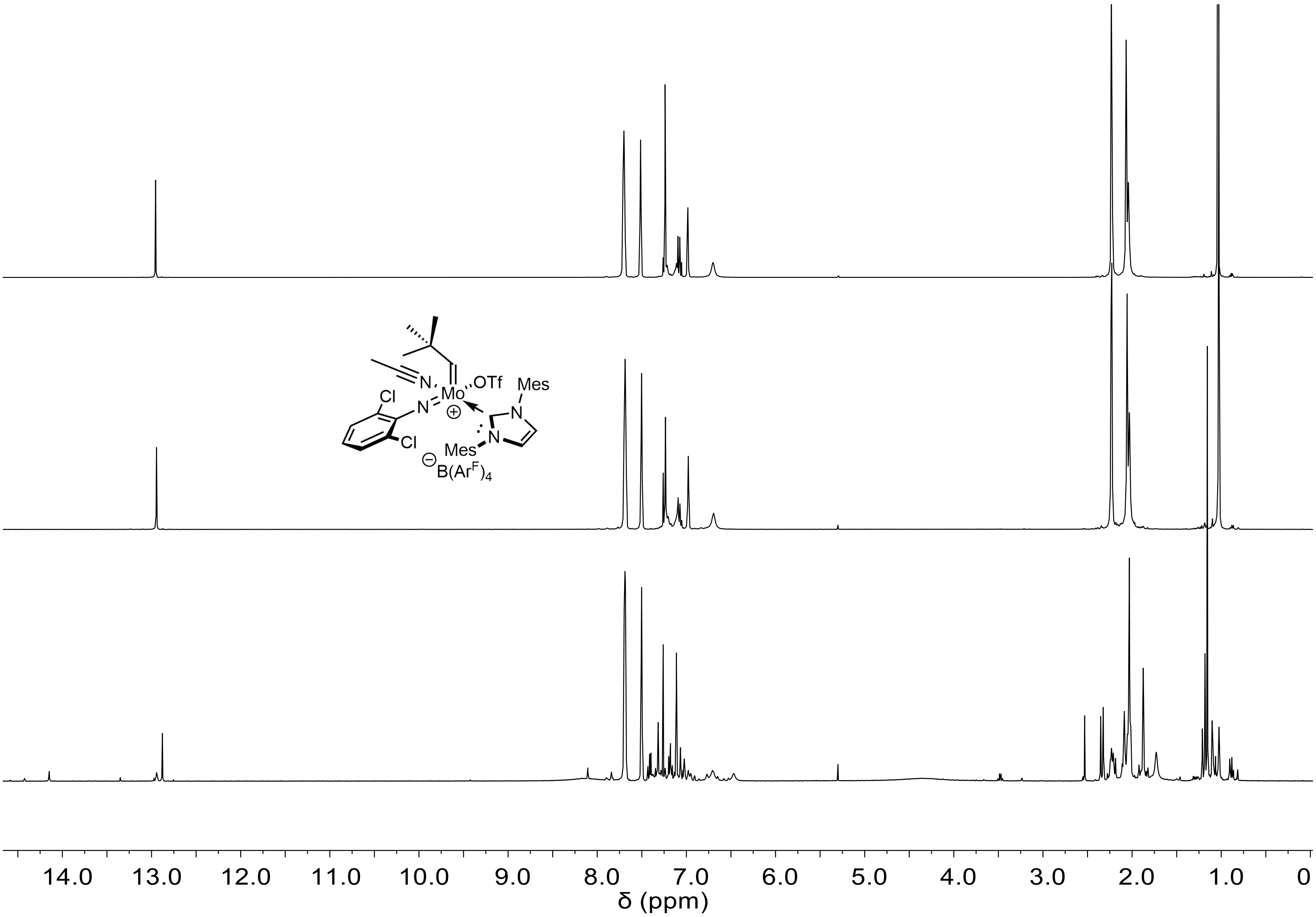

Figure S 181: ^{1}H-NMR (400 MHz, 25 °C, $CDCl_3$) of Mo-03-MeCN (upper), Mo-03-MeCN after exposure to air overnight (middle) and Mo-03-MeCN after exposure to air for two weeks (lower).

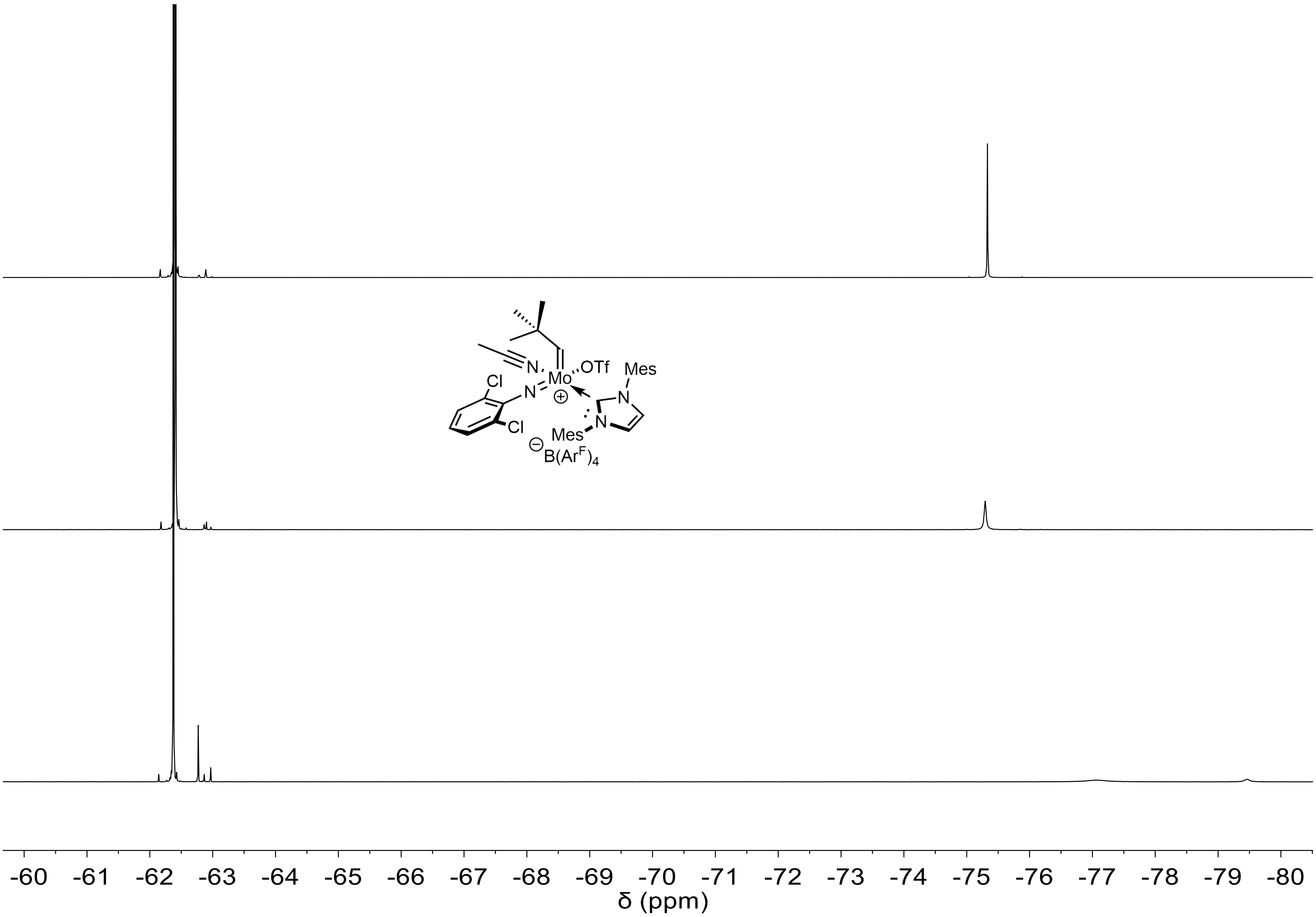

Figure S 182: ^{19}F-NMR (376 MHz, 25 °C, $CDCl_3$) of Mo-03-MeCN (upper), Mo-03-MeCN after exposure to air overnight (middle) and Mo-03-MeCN after exposure to air for two weeks (lower).

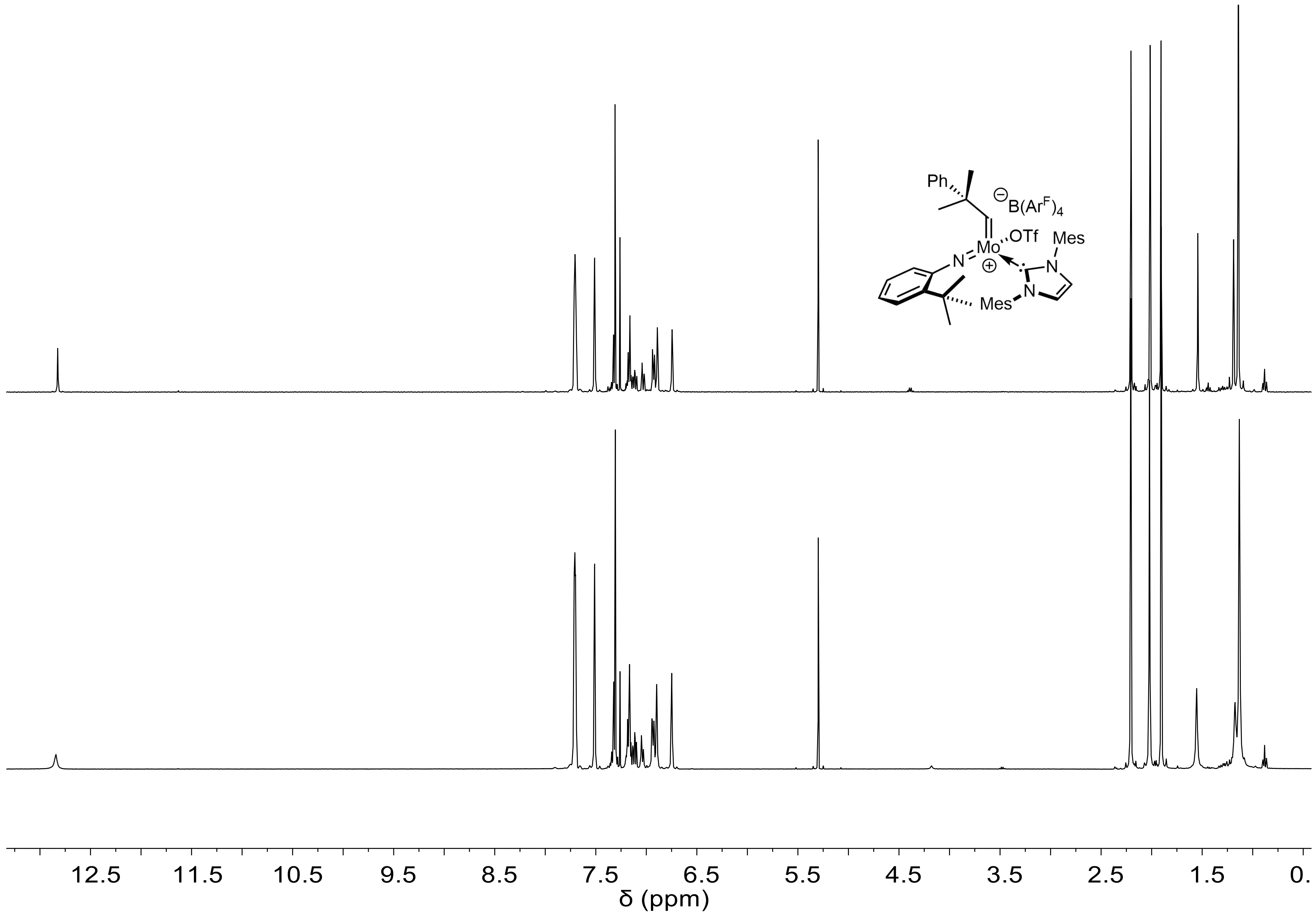

Figure S 183: ^{1}H-NMR (400 MHz, 25 °C, $CDCl_3$) of Mo-04 (upper) and Mo-04 after exposure to air overnight (lower).

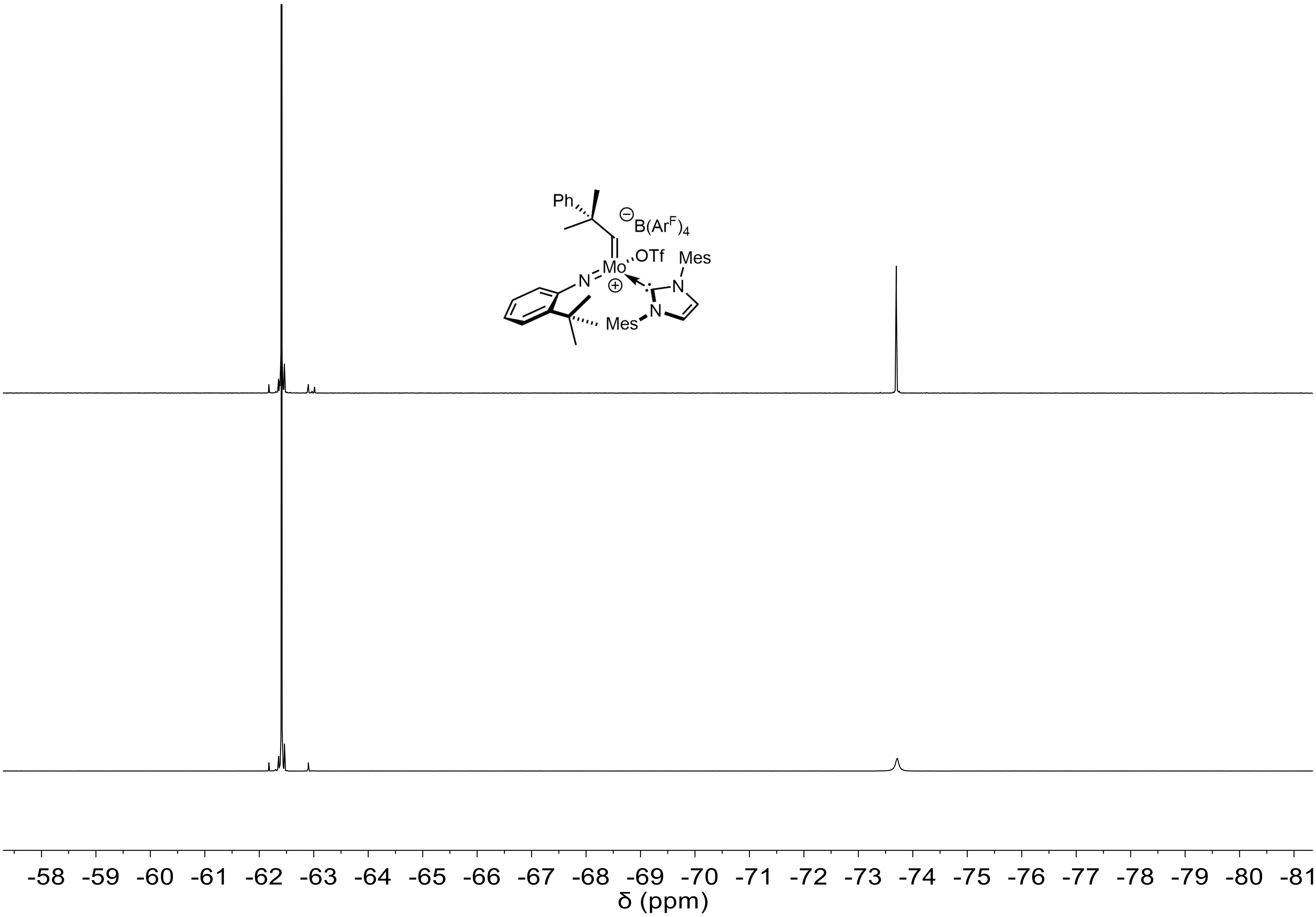

Figure S 184: ^{19}F-NMR (376 MHz, 25 °C, $CDCl_3$) of Mo-04 (upper) and Mo-04 after exposure to air overnight (lower).

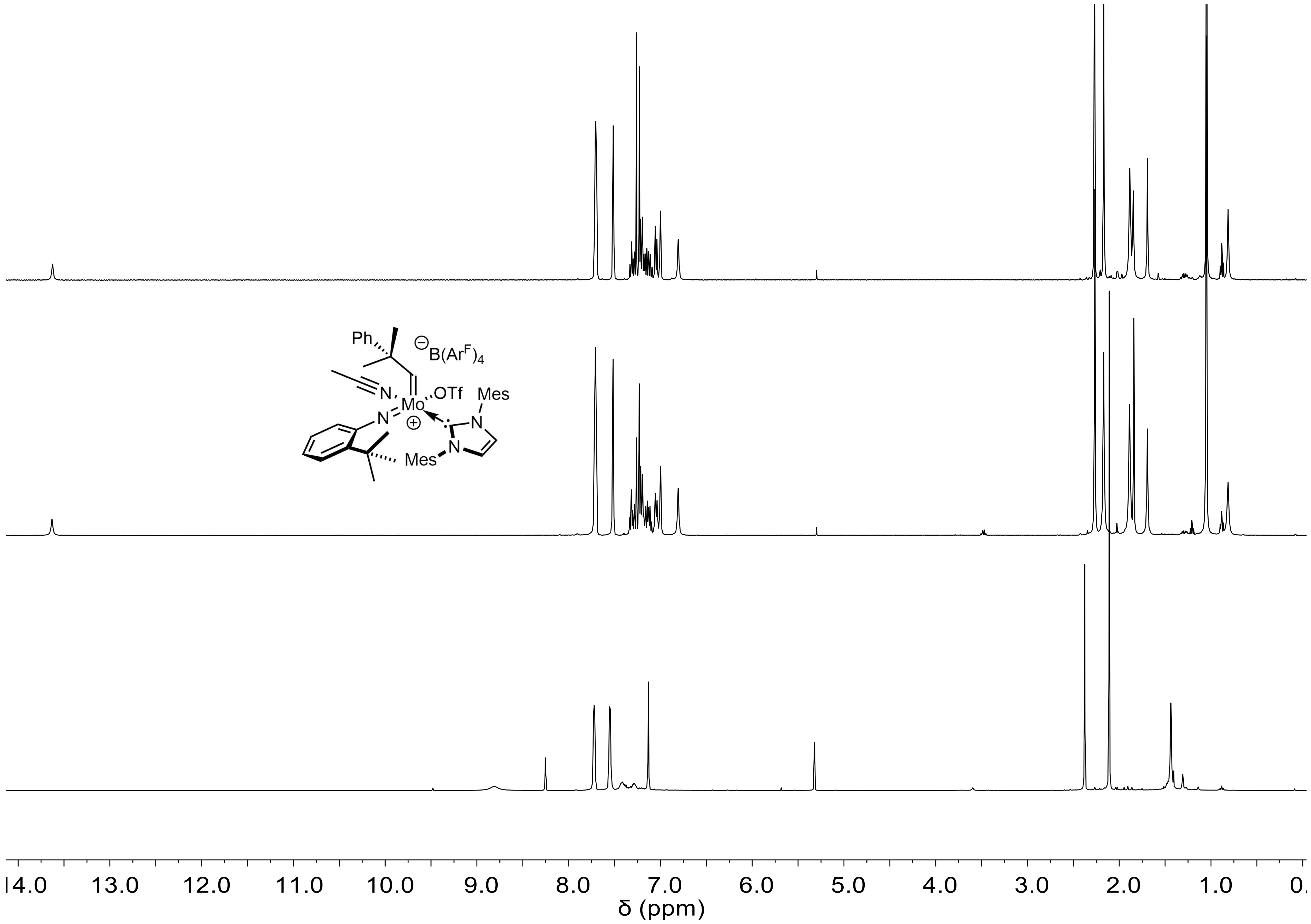

Figure S 185: ^{1}H-NMR (400 MHz, 25 °C) of Mo-04-MeCN (upper, $CDCl_3$), Mo-04-MeCN after exposure to air overnight (middle, $CDCl_3$) and Mo-04-MeCN after exposure to air for two weeks (lower, CD_2Cl_2).

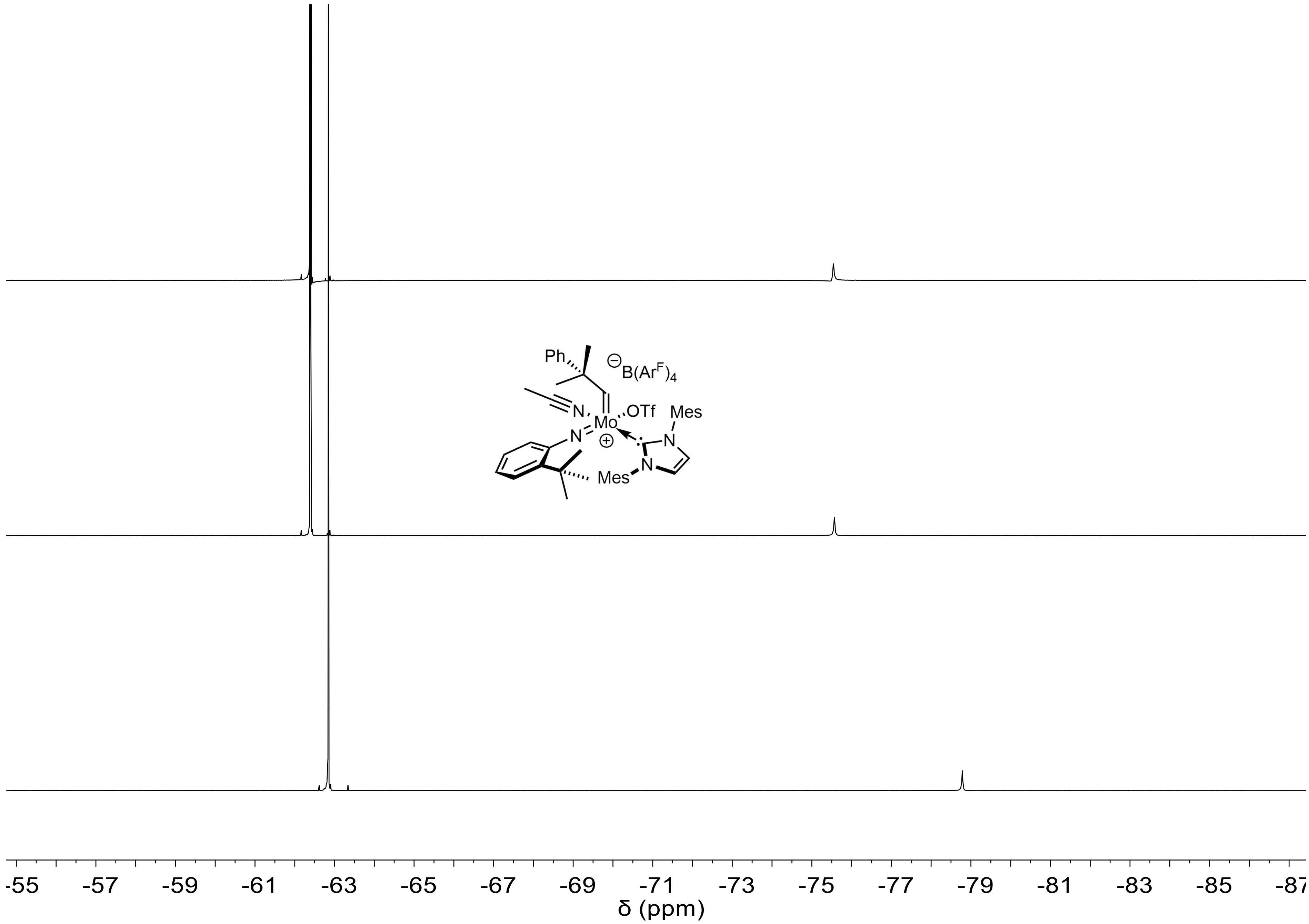

Figure S 186: 1H-NMR (400 MHz, 25 °C) of Mo-04-MeCN (upper, $CDCl_3$), Mo-04-MeCN after exposure to air overnight (middle, $CDCl_3$) and Mo-04-MeCN after exposure to air for two weeks (lower, CD_2Cl_2).

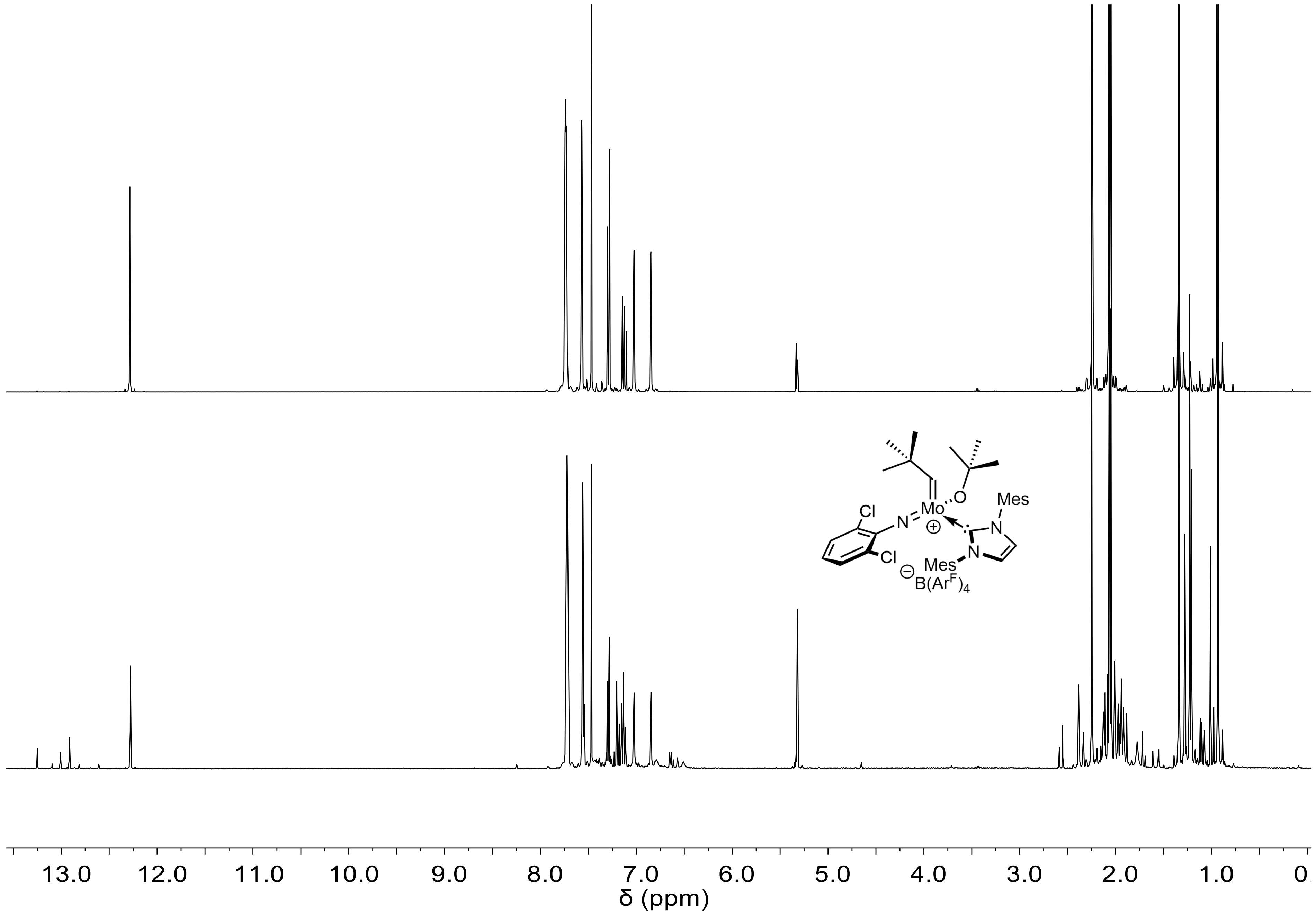

Figure S 187: ^{1}H-NMR (400 MHz, 25 °C, CD_2Cl_2) of Mo-05 (upper) and Mo-05 after exposure to air overnight (lower).

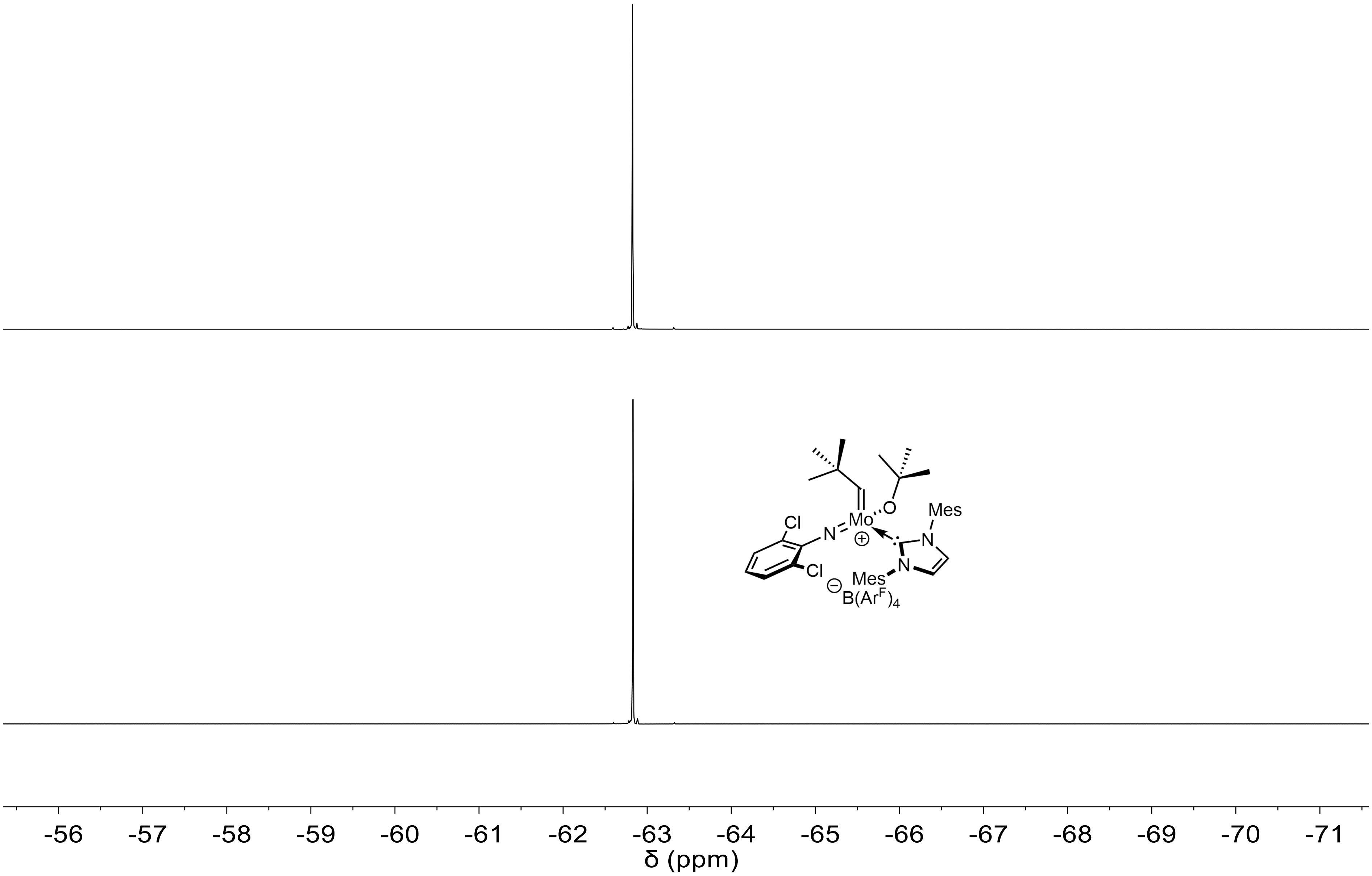

Figure S 188: ^{19}F-NMR (376 MHz, 25 °C, CD_2Cl_2) of Mo-05 (upper) and Mo-05 after exposure to air overnight (lower).

6. NMR Spectra of Polymerizations

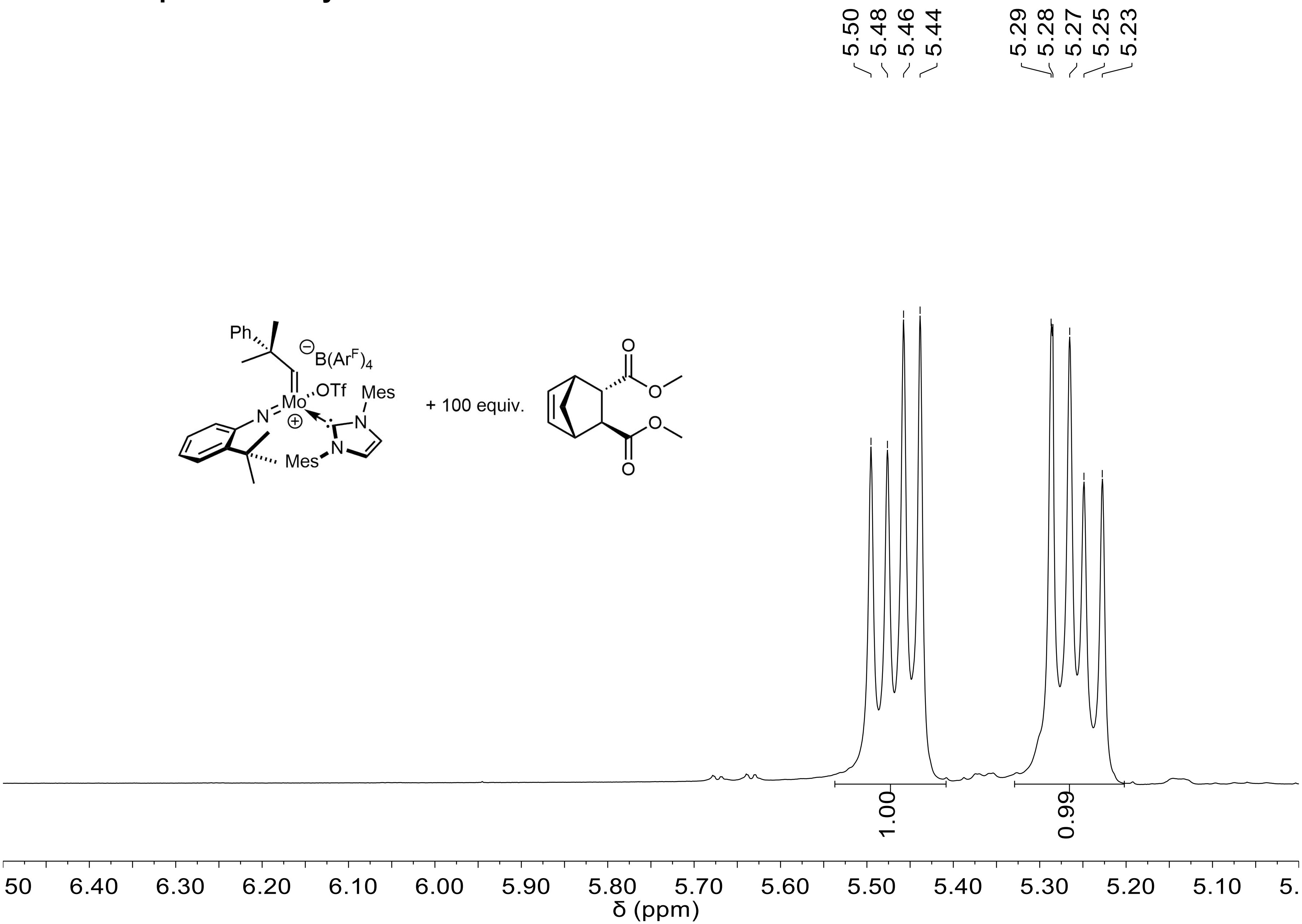

Figure S 189: Olefinic region of the ^{1}H-NMR (400 MHz, 25 °C, $CDCl_3$) spectrum of the polymerization of 100 equiv. (+)-DCMNBE by the action of Mo-04.

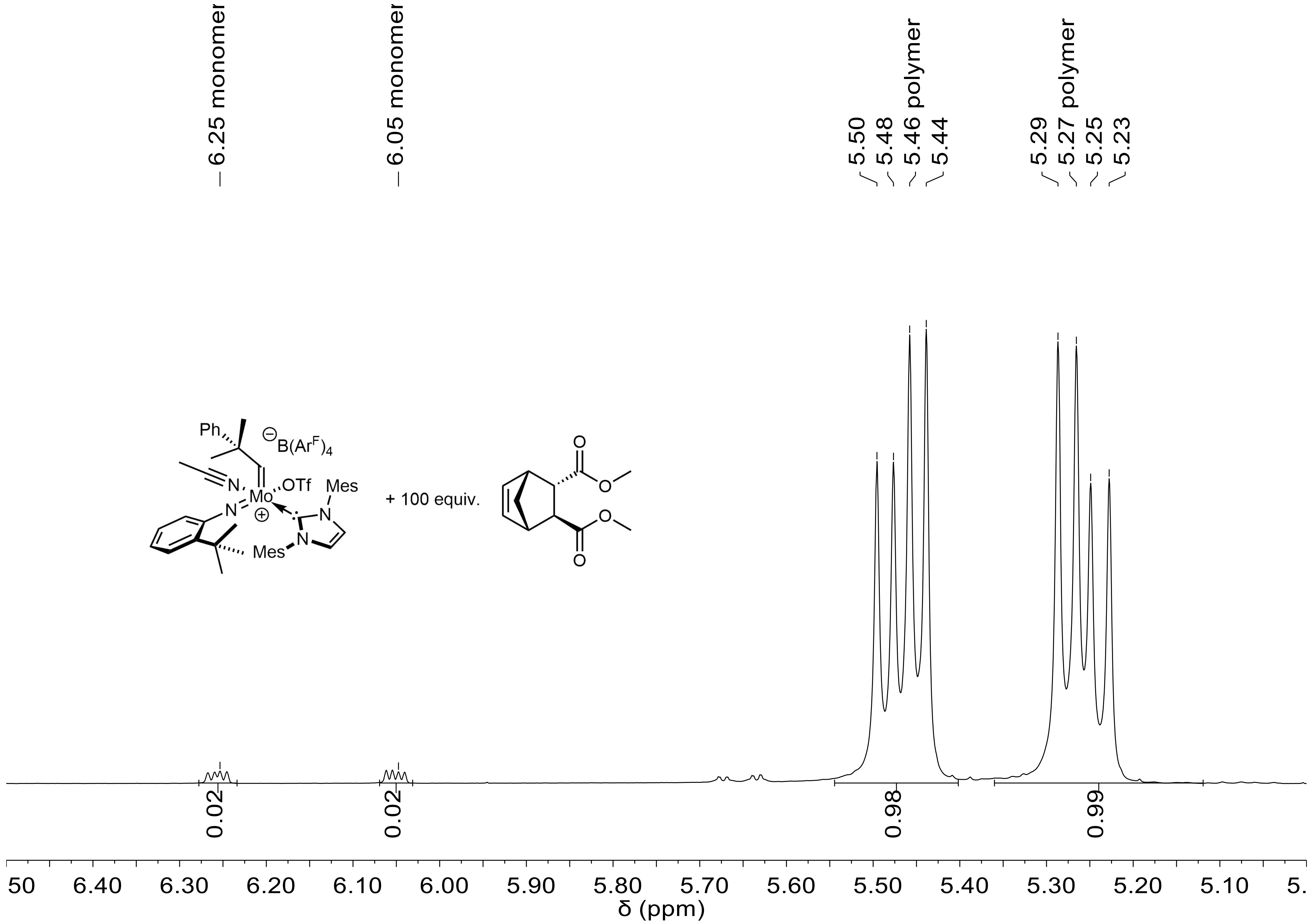

Figure S 190: Olefinic region of the ^{1}H-NMR (400 MHz, 25 °C, $CDCl_3$) spectrum of the polymerization of 100 equiv. (+)-DCMNBE by the action of Mo-04-MeCN.

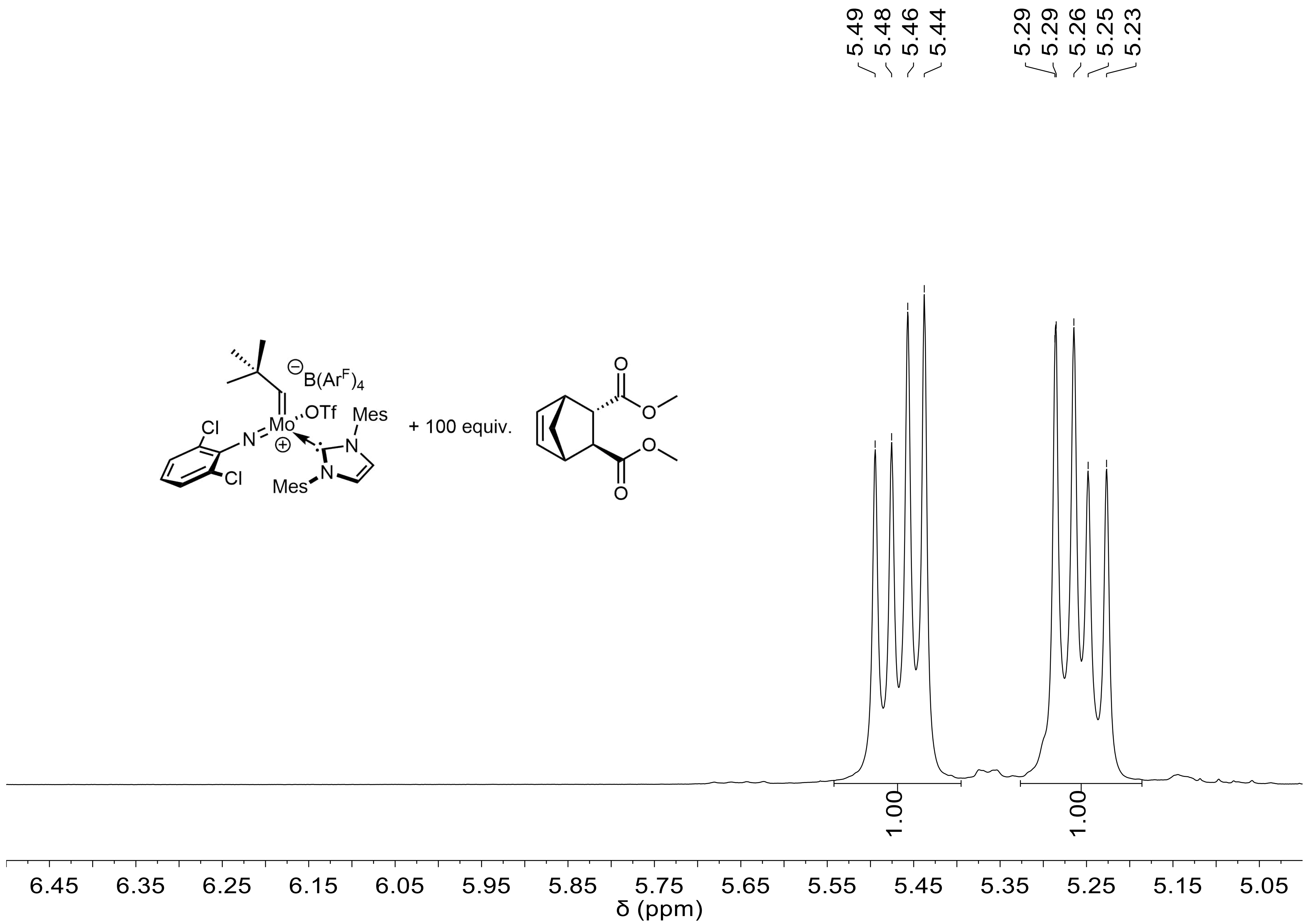

Figure S 191: Olefinic region of the ^{1}H-NMR (400 MHz, 25 °C, $CDCl_3$) spectrum of the polymerization of 100 equiv. (+)-DCMNBE by the action of Mo-03.

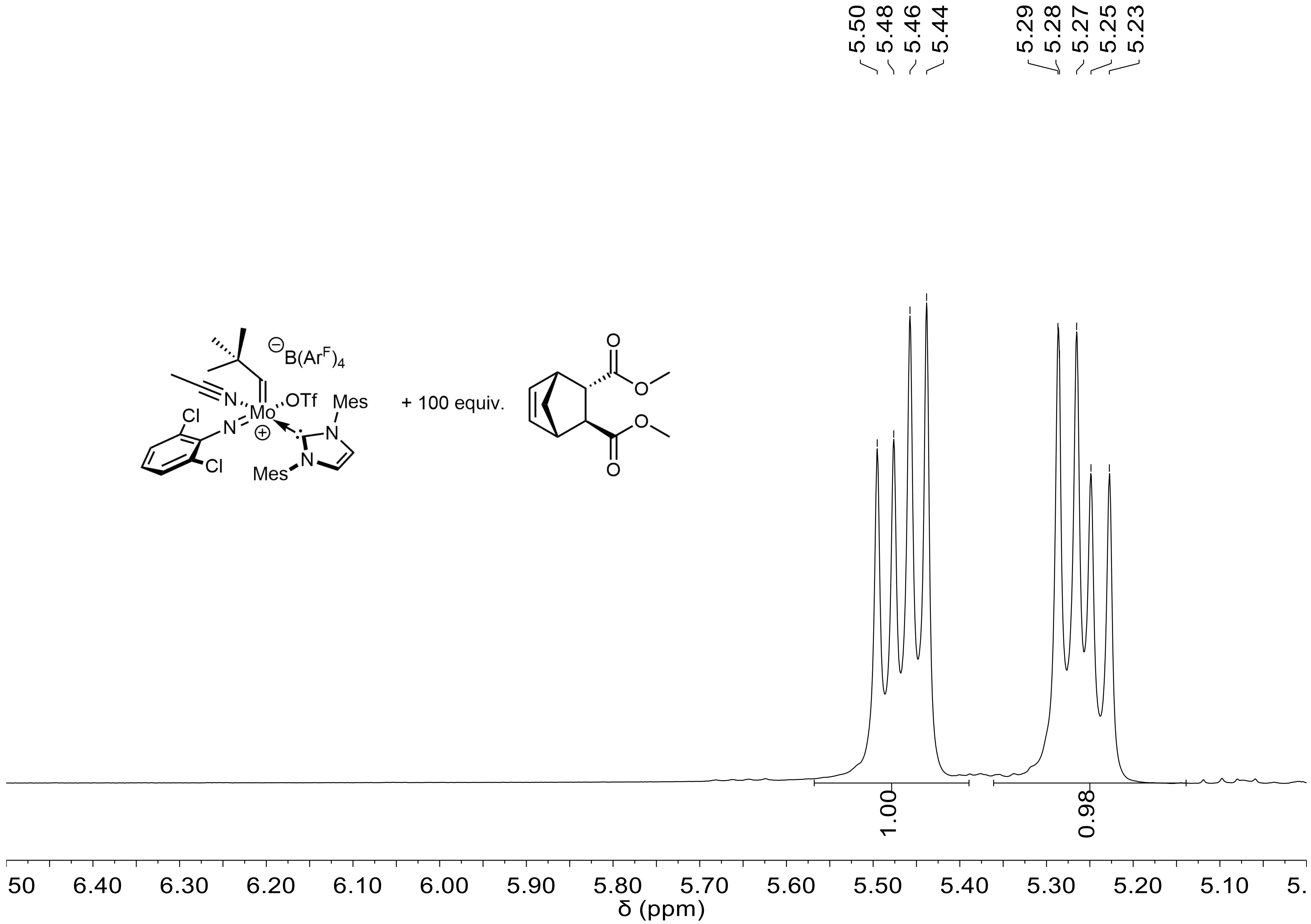

Figure S 192: Olefinic region of the ^{1}H-NMR (400 MHz, 25 °C, $CDCl_3$) spectrum of the polymerization of 100 equiv. (+)-DCMNBE by the action of Mo-03-MeCN.

7. NMR Experiments

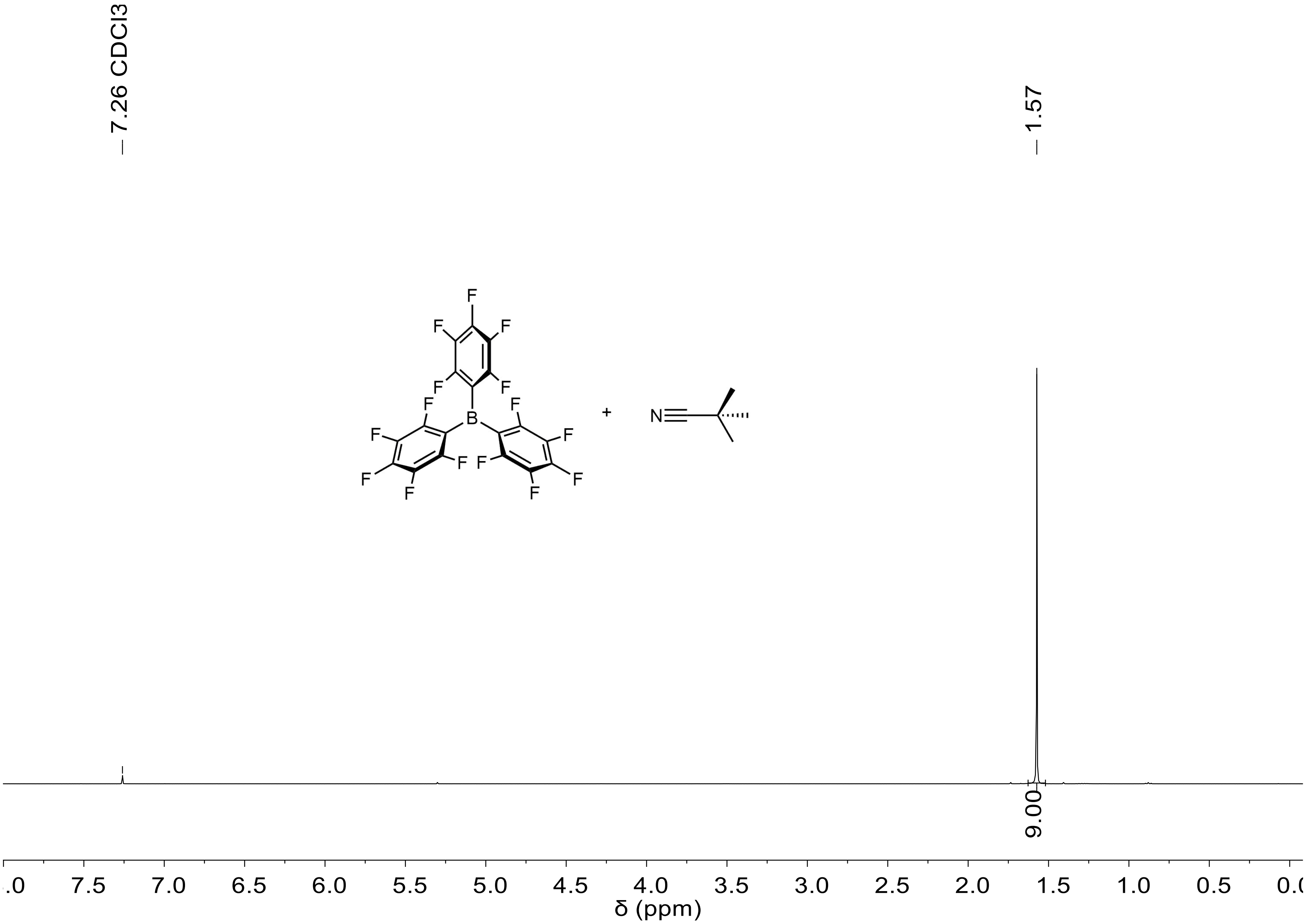

Figure S 193: ^{1}H-NMR (400 MHz, 25 °C, $CDCl_3$) spectrum of the reaction between pivalonitrile and BCF (1.2 equiv.).

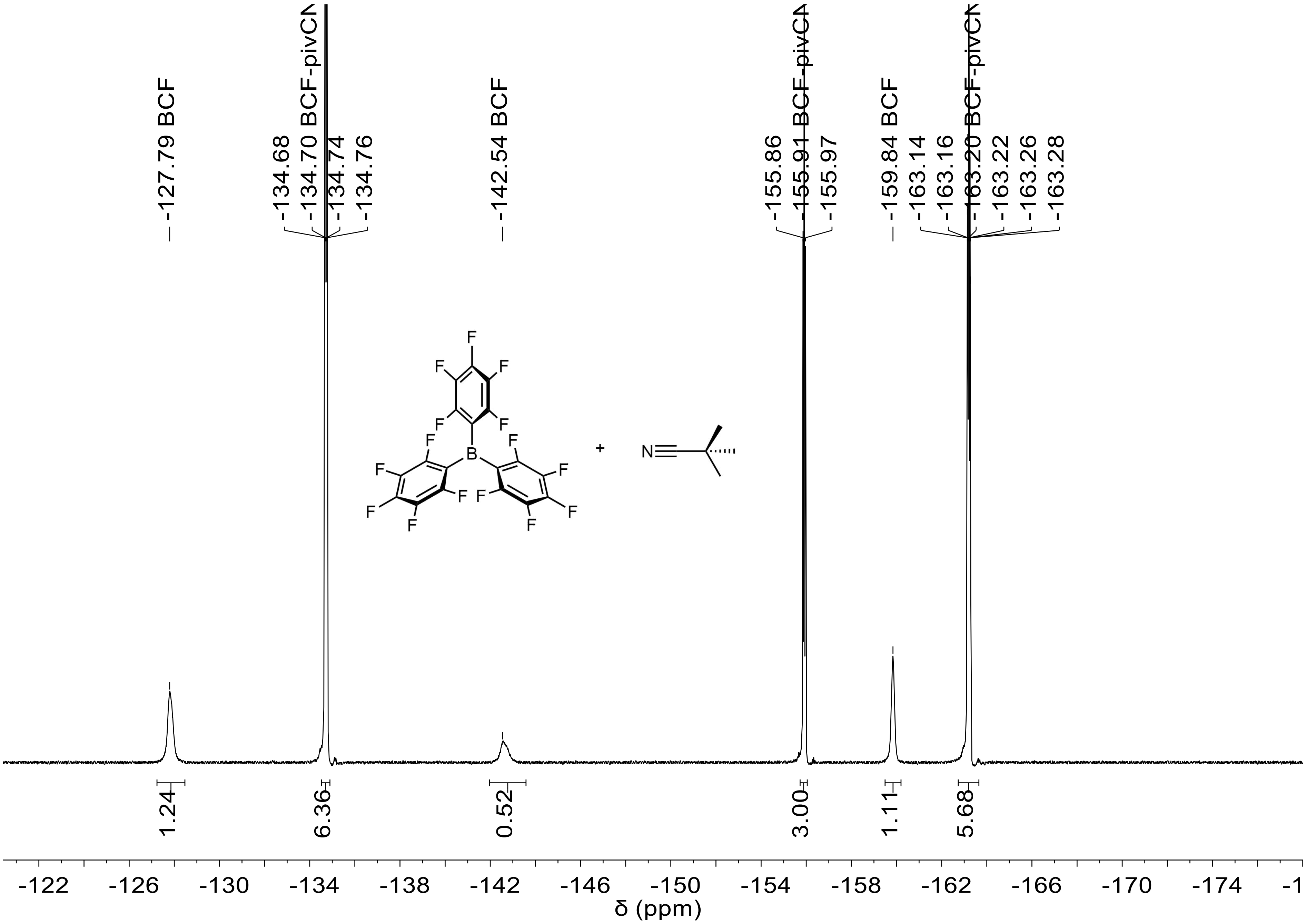

Figure S 194: ^{19}F-NMR (376 MHz, 25 °C, $CDCl_3$) spectrum of the reaction between pivalonitrile and BCF (1.2 equiv.).

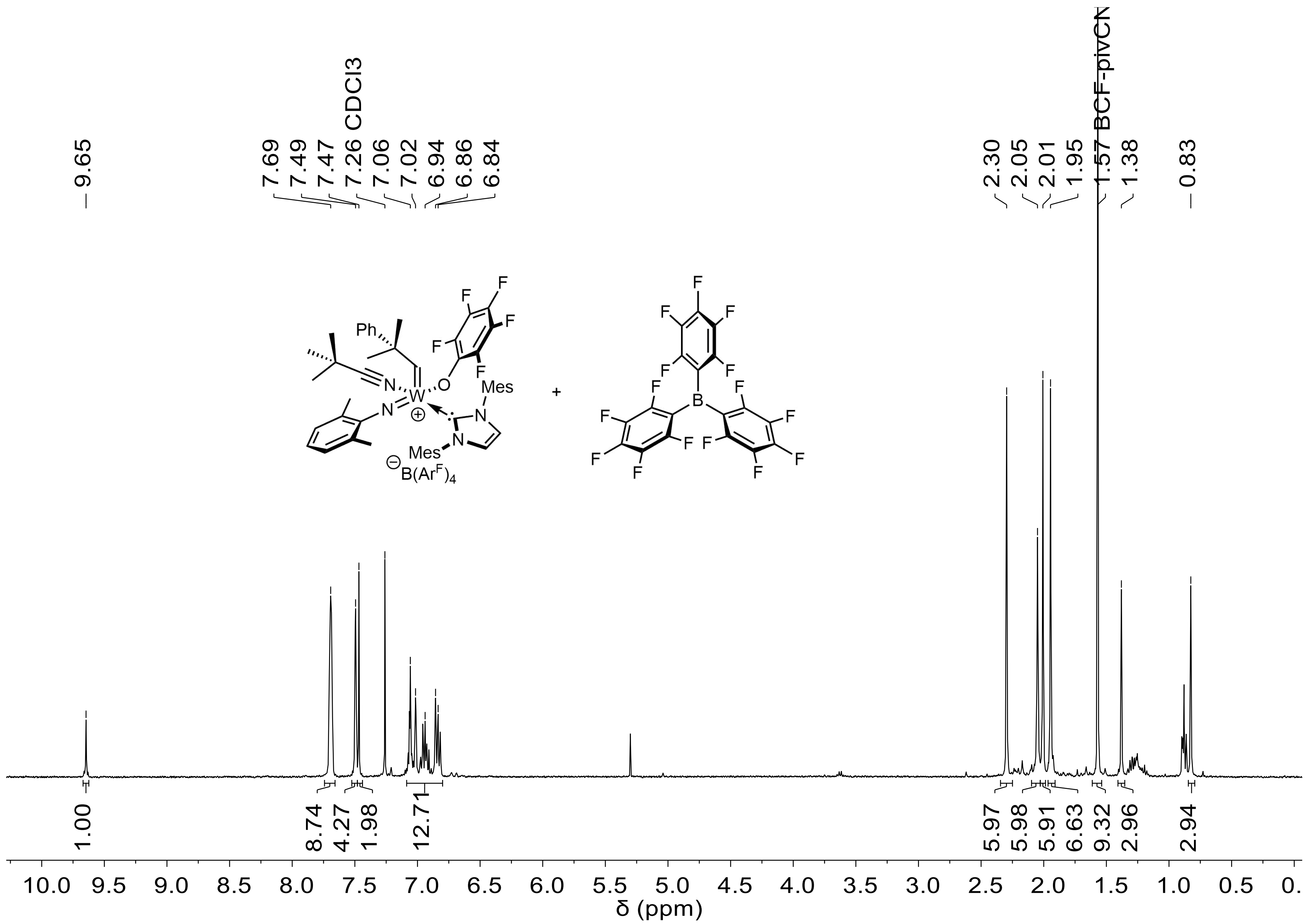

Figure S 195: **^{1}H-NMR (400 MHz, 25 °C, $CDCl_3$) spectrum of W-31 in the presence of BCF (6 equiv.).**

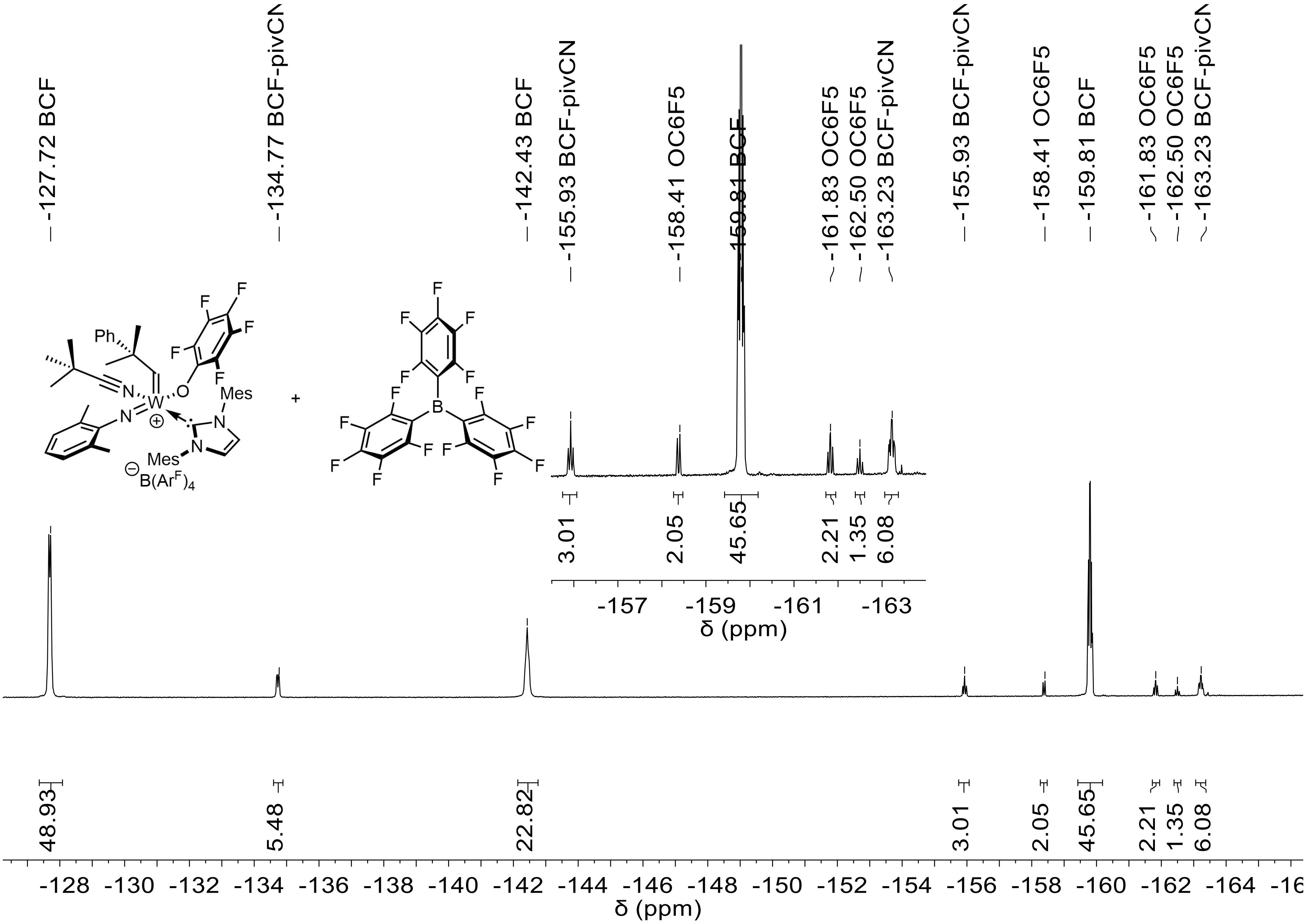

Figure S 196: ^{19}F-NMR (376 MHz, 25 °C, $CDCl_3$) spectrum of W-31 in the presence of BCF (6 equiv.).

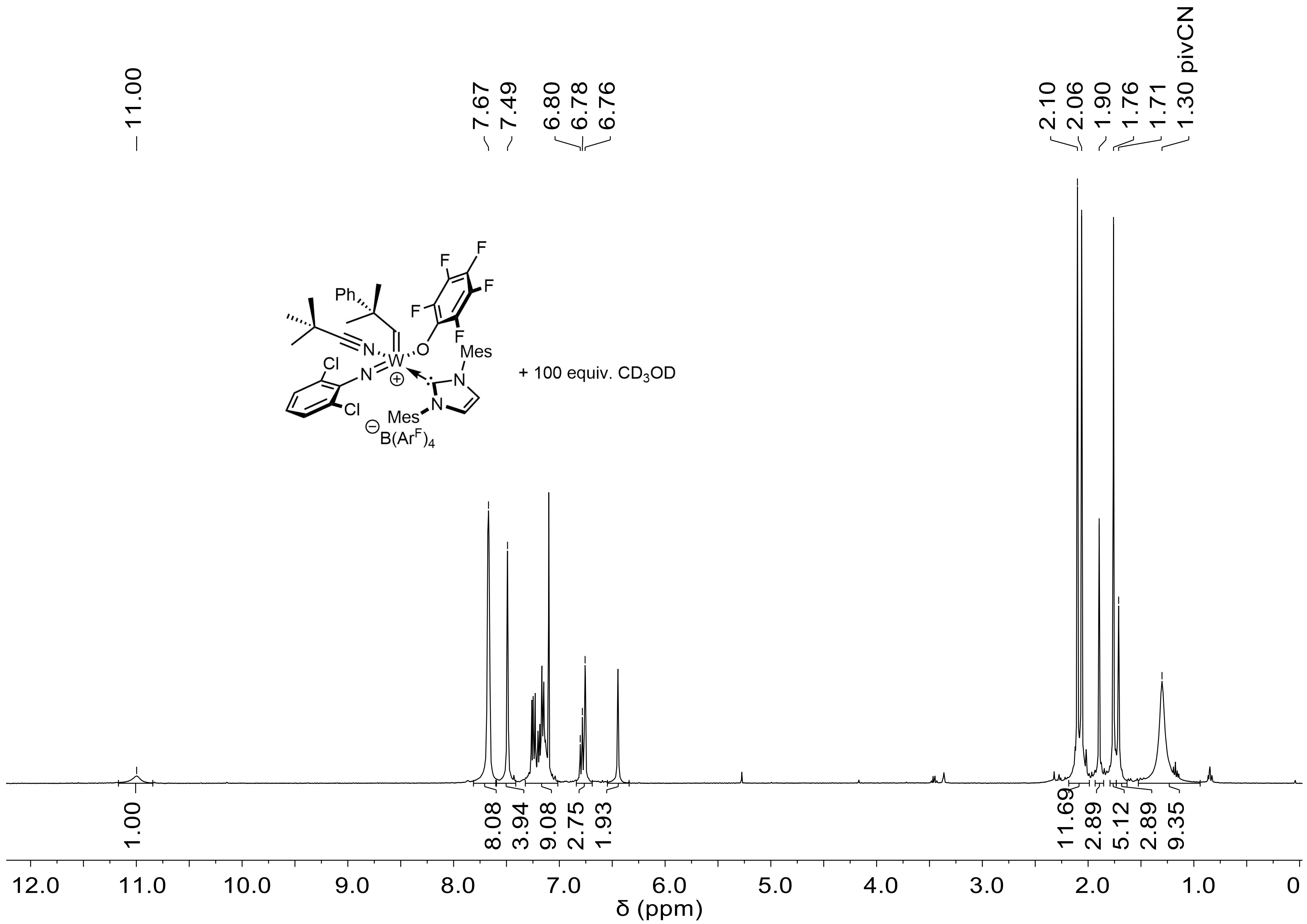

Figure S 197: ^{1}H-NMR (400 MHz, 25 °C, $CDCl_3$) spectrum of W-33 in the presence of CD_3OD (100 equiv.).

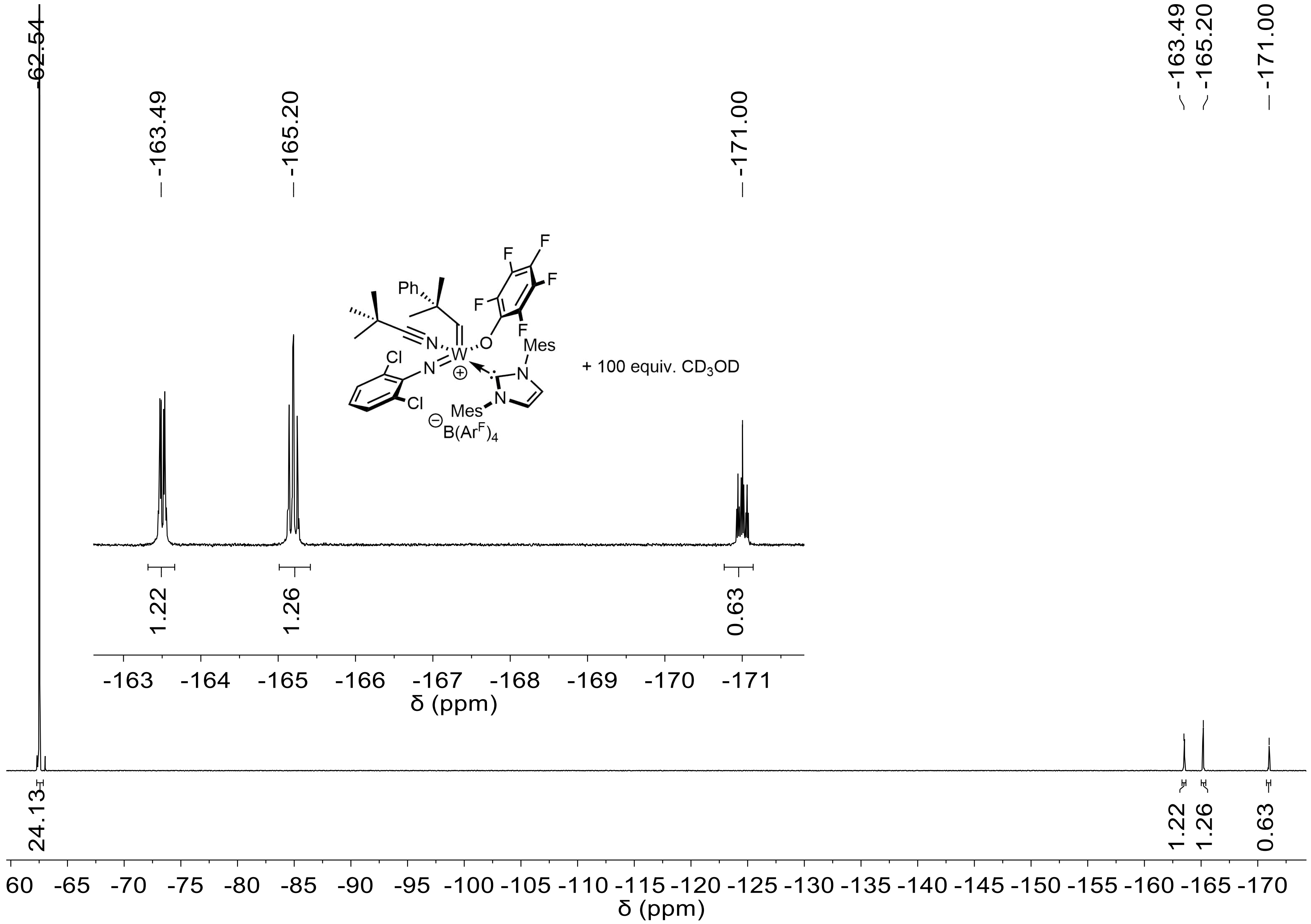

Figure S 198: ^{19}F-NMR (376 MHz, 25 °C, $CDCl_3$) spectrum of W-33 in the presence of CD_3OD (100 equiv.).

8. Single-Crystal X-Ray Data

Table S 2: Crystal data and structure refinement for W-13.

Empirical formula	$C_{79.50}H_{75}BBrCl_{1.50}F_{24}N_4O_{0.25}W$
Formula weight	1874.18
Temperature	135(2) K
Wavelength	1.54178 Å
Crystal system, space group	Monoclinic, Pc
Unit cell dimensions	a = 27.5725(10) Å, α = 90 °
	b = 12.4705(4) Å, β = 91.523(2) °
	c = 23.7591(7) Å, γ = 90 °
Volume	8166.5(5) $Å^3$
Z, Calculated density	4, 1.524 mg/m^3
Absorption coefficient	4.527 mm^{-1}
F(000)	3750
Crystal size	0.112 x 0.101 x 0.030 mm
Θ range for data collection	3.544 to 65.986 °
Limiting indices	-31<=h<=32, -12<=k<=14, -28<=l<=25
Reflections collected / unique	54200 / 22445 [R(int) = 0.0439]
Completeness to Θ = 65.986	97.2 %
Absorption correction	Numerical
Max. and min. transmission	0.9230 and 0.6861
Refinement method	Full-matrix least-squares on F^2
Data / restraints / parameters	22445 / 35 / 2071
Goodness-of-fit on F^2	1.023
Final R indices [I>2σ(I)]	R1 = 0.0437, wR2 = 0.0945
R indices (all data)	R1 = 0.0560, wR2 = 0.0991
Absolute structure parameter	0.308(8)
Extinction coefficient	n/a
Largest diff. peak and hole	1.034 and -0.533 e. $Å^{-3}$

Table S 3: Atomic coordinates (x 10^4) and equivalent isotropic displacement parameters ($Å^2$ x 10^3) for W-13. U(eq) is defined as one third of the trace of the orthogonalized U_{ij} tensor.

	x	y	z	U(eq)
W(1A)	-172(1)	5989(1)	5017(1)	21(1)
BR1A	-764(1)	6198(1)	4232(1)	33(1)
N(1A)	-117(3)	3865(6)	5535(3)	29(2)
C(1A)	134(4)	4769(7)	5598(4)	26(2)
N(2A)	509(3)	4822(6)	5955(3)	27(2)
C(2A)	-4(4)	2873(8)	5849(5)	39(3)
N(3A)	368(3)	6300(6)	4673(3)	26(2)
C(3A)	522(5)	2846(9)	5982(6)	55(4)

N(4A)	-364(3)	6970(6)	5496(3)	24(2)
C(4A)	678(5)	3867(8)	6273(5)	41(3)
C(5A)	-547(4)	3940(7)	5179(4)	27(2)
C(6A)	-563(4)	3468(8)	4648(4)	30(2)
C(7A)	-1001(4)	3494(8)	4346(4)	36(3)
C(8A)	-1409(4)	3983(8)	4543(4)	34(2)
C(9A)	-1383(4)	4416(8)	5075(5)	33(2)
C(10A)	-975(4)	4388(7)	5418(4)	27(2)
C(11A)	-124(4)	2881(10)	4428(5)	50(3)
C(12A)	-1866(4)	4072(10)	4182(5)	54(3)
C(13A)	-987(4)	4742(8)	6032(4)	32(2)
C(14A)	764(4)	5814(7)	6065(4)	28(3)
C(15A)	1179(4)	6059(8)	5782(4)	30(3)
C(16A)	1414(4)	7027(8)	5908(4)	33(2)
C(17A)	1252(4)	7693(8)	6335(4)	37(3)
C(18A)	854(4)	7386(8)	6640(4)	32(2)
C(19A)	598(4)	6449(8)	6509(4)	31(2)
C(20A)	1388(4)	5306(8)	5364(4)	35(3)
C(21A)	1514(5)	8724(9)	6478(5)	48(3)
C(22A)	163(4)	6158(8)	6843(4)	37(3)
C(23A)	762(4)	6722(8)	4401(4)	33(3)
C(24A)	1018(5)	6032(9)	4037(5)	38(3)
C(25A)	1437(4)	6435(11)	3785(5)	52(3)
C(26A)	1581(5)	7492(13)	3892(6)	65(4)
C(27A)	1327(4)	8185(11)	4232(5)	50(3)
C(28A)	915(4)	7787(9)	4496(4)	37(3)
C(29A)	851(4)	4910(9)	3930(4)	40(3)
C(30A)	1264(6)	4116(11)	3837(6)	65(4)
C(31A)	479(5)	4907(10)	3438(5)	60(4)
C(32A)	609(5)	8522(8)	4848(4)	43(3)
C(33A)	152(5)	8895(9)	4500(5)	48(3)
C(34A)	891(6)	9511(11)	5077(5)	76(5)
C(35A)	-534(4)	7869(7)	5776(4)	23(2)
C(36A)	-1075(4)	7962(8)	5737(4)	34(3)
C(37A)	-227(4)	8523(7)	6050(4)	25(2)
C(38A)	-349(4)	9457(8)	6431(4)	33(3)
C(39A)	126(4)	10062(8)	6572(5)	45(3)
C(40A)	-541(5)	8979(8)	6983(4)	45(3)
C(41A)	-715(4)	10266(7)	6178(4)	27(2)
C(42A)	-996(4)	10915(8)	6512(4)	34(2)
C(43A)	-1302(4)	11676(8)	6282(4)	37(2)
C(44A)	-1349(4)	11811(8)	5713(4)	39(3)
C(45A)	-1064(4)	11188(8)	5364(5)	40(3)
C(46A)	-743(4)	10437(8)	5603(4)	37(3)

B(1A)	7791(4)	7460(9)	2866(4)	28(2)
C(47A)	7794(3)	6479(7)	2404(4)	27(2)
C(48A)	7581(3)	6566(8)	1870(4)	26(2)
C(49A)	7593(3)	5692(8)	1490(4)	33(2)
C(50A)	7805(4)	4720(8)	1630(4)	33(2)
C(51A)	8015(3)	4639(7)	2160(4)	32(2)
C(52A)	8007(4)	5489(8)	2533(4)	28(2)
C(53A)	7362(4)	5895(9)	921(4)	42(3)
F(53A)	7609(3)	6573(7)	616(3)	69(2)
F(53B)	7338(3)	4999(6)	605(3)	80(3)
F(53C)	6917(2)	6253(6)	936(3)	60(2)
C(54A)	8262(4)	3635(9)	2332(5)	44(3)
F(54A)	7955(4)	2969(7)	2580(4)	101(3)
F(54B)	8410(4)	3064(7)	1906(3)	108(4)
F(54C)	8602(4)	3721(7)	2705(5)	122(4)
C(55A)	7549(3)	6972(7)	3441(4)	29(2)
C(56A)	7181(4)	6225(7)	3414(3)	29(2)
C(57A)	6965(4)	5826(8)	3880(4)	33(3)
C(58A)	7102(4)	6169(8)	4415(4)	31(2)
C(59A)	7465(4)	6907(8)	4466(4)	28(2)
C(60A)	7699(4)	7295(8)	3984(3)	28(2)
C(61A)	6580(4)	4961(9)	3815(4)	42(3)
F(61A)	6278(2)	5134(5)	3380(2)	52(2)
F(61B)	6311(3)	4818(6)	4266(2)	68(2)
F(61C)	6782(3)	4000(5)	3707(3)	65(2)
C(62A)	7636(4)	7269(8)	5038(4)	34(2)
F(62A)	8002(2)	6658(5)	5238(2)	43(2)
F(62B)	7808(3)	8269(5)	5037(2)	59(2)
F(62C)	7288(2)	7236(5)	5408(2)	51(2)
C(63A)	8343(4)	7918(7)	2984(3)	27(2)
C(64A)	8764(4)	7399(7)	2835(3)	29(2)
C(65A)	9225(4)	7817(8)	2954(4)	31(2)
C(66A)	9277(4)	8781(7)	3222(4)	30(2)
C(67A)	8868(4)	9334(8)	3379(4)	31(2)
C(68A)	8403(4)	8915(8)	3251(3)	31(2)
C(69A)	9665(4)	7222(9)	2755(4)	41(3)
F(69A)	9810(3)	7550(7)	2266(3)	100(3)
F(69B)	10048(3)	7360(6)	3109(4)	77(2)
F(69C)	9603(2)	6181(5)	2720(3)	55(2)
C(70A)	8912(5)	10415(10)	3649(5)	48(3)
F(70A)	8566(3)	10651(7)	3976(4)	111(4)
F(70B)	9317(3)	10555(8)	3932(4)	111(4)
F(70C)	8912(6)	11190(7)	3295(4)	151(6)
C(71A)	7453(3)	8452(7)	2622(3)	24(2)

C(72A)	6971(4)	8666(8)	2778(4)	34(2)
C(73A)	6699(4)	9497(9)	2554(4)	37(2)
C(74A)	6901(4)	10167(8)	2149(4)	37(2)
C(75A)	7367(4)	9982(8)	1987(4)	35(2)
C(76A)	7634(4)	9157(7)	2218(4)	31(2)
C(77A)	6202(5)	9679(11)	2744(6)	59(3)
F(77A)	5985(4)	10493(9)	2534(5)	133(5)
F(77B)	5910(3)	8880(9)	2613(6)	138(5)
F(77C)	6155(3)	9656(11)	3282(4)	119(4)
C(78A)	7579(4)	10679(8)	1551(4)	36(3)
F(78A)	8054(2)	10782(6)	1605(3)	67(2)
F(78B)	7397(2)	11642(5)	1512(2)	49(2)
F(78C)	7507(3)	10233(6)	1027(2)	74(2)
W(1B)	5070(1)	4233(1)	6410(1)	23(1)
BR1B	5695(1)	4111(1)	7164(1)	37(1)
N(1B)	4337(3)	5351(6)	5511(3)	29(2)
C(1B)	4741(4)	5417(8)	5834(4)	28(2)
N(2B)	4983(3)	6326(6)	5863(3)	30(2)
C(2B)	4136(4)	6285(8)	5195(5)	36(3)
N(3B)	4556(3)	3843(6)	6784(3)	30(2)
C(3B)	4321(5)	7301(9)	5419(6)	51(3)
N(4B)	5270(3)	3269(6)	5934(3)	27(2)
C(4B)	4871(5)	7272(8)	5510(5)	47(3)
C(5B)	4109(5)	4327(8)	5390(4)	30(3)
C(6B)	3706(4)	4026(7)	5698(4)	26(2)
C(7B)	3480(4)	3056(8)	5564(4)	33(2)
C(8B)	3634(4)	2423(8)	5124(4)	34(3)
C(9B)	4030(4)	2779(8)	4813(4)	35(3)
C(10B)	4273(4)	3705(8)	4935(4)	33(2)
C(11B)	3494(4)	4760(8)	6136(4)	34(2)
C(12B)	3384(5)	1367(9)	4974(5)	51(3)
C(13B)	4701(5)	4073(9)	4608(5)	42(3)
C(14B)	5410(4)	6319(8)	6230(4)	27(2)
C(15B)	5845(5)	6001(8)	6041(4)	33(3)
C(16B)	6261(4)	6082(8)	6391(5)	34(3)
C(17B)	6232(4)	6510(8)	6927(4)	34(2)
C(18B)	5788(4)	6870(8)	7101(4)	37(3)
C(19B)	5365(4)	6807(7)	6767(5)	33(2)
C(20B)	5911(5)	5624(9)	5439(4)	43(3)
C(21B)	6683(4)	6598(10)	7298(5)	53(3)
C(22B)	4903(5)	7289(10)	6956(5)	51(3)
C(23B)	4171(4)	3343(8)	7056(4)	30(2)
C(24B)	3913(5)	3983(9)	7434(5)	39(3)
C(25B)	3536(5)	3512(11)	7702(5)	50(3)

C(26B)	3418(5)	2477(12)	7618(5)	59(4)
C(27B)	3680(5)	1859(11)	7243(5)	58(4)
C(28B)	4060(4)	2280(9)	6955(4)	37(3)
C(29B)	4058(4)	5140(9)	7554(4)	41(3)
C(30B)	4444(5)	5226(11)	8011(5)	59(4)
C(31B)	3644(6)	5867(10)	7700(6)	61(4)
C(32B)	4375(4)	1596(8)	6589(4)	39(3)
C(33B)	4837(5)	1281(9)	6902(5)	43(3)
C(34B)	4128(5)	593(10)	6347(5)	54(3)
C(35B)	5448(4)	2365(8)	5654(4)	33(2)
C(36B)	5984(4)	2263(10)	5759(6)	55(4)
C(37B)	5155(4)	1747(7)	5333(4)	29(2)
C(38B)	5289(4)	825(8)	4949(5)	34(3)
C(39B)	4838(4)	153(9)	4821(5)	47(3)
C(40B)	5461(4)	1333(9)	4391(4)	41(3)
C(41B)	5663(4)	65(8)	5212(4)	35(2)
C(42B)	5612(4)	-305(8)	5752(5)	40(3)
C(43B)	5937(5)	-1030(9)	5999(5)	45(3)
C(44B)	6332(4)	-1356(9)	5722(5)	45(3)
C(45B)	6398(4)	-1020(9)	5177(5)	49(3)
C(46B)	6073(4)	-280(9)	4921(4)	44(3)
B(1B)	2423(5)	8519(8)	8245(5)	23(2)
C(47B)	2384(3)	7773(7)	8804(4)	23(2)
C(48B)	2618(4)	6789(7)	8853(4)	25(2)
C(49B)	2622(4)	6188(7)	9351(4)	26(2)
C(50B)	2389(4)	6548(8)	9817(4)	30(2)
C(51B)	2161(4)	7535(7)	9783(4)	26(2)
C(52B)	2156(3)	8126(7)	9295(4)	26(2)
C(53B)	2859(4)	5108(8)	9339(4)	33(3)
F(53D)	2843(3)	4600(5)	9838(3)	61(2)
F(53E)	2668(3)	4460(5)	8954(3)	63(2)
F(53F)	3335(2)	5161(5)	9235(3)	52(2)
C(54B)	1909(5)	7973(9)	10292(5)	43(3)
F(54D)	1946(4)	7367(7)	10725(3)	106(4)
F(54E)	2019(5)	8956(7)	10402(4)	119(5)
F(54F)	1434(4)	8019(10)	10202(4)	114(4)
C(55B)	2479(4)	7795(7)	7659(4)	24(2)
C(56B)	2239(4)	6812(7)	7596(4)	24(2)
C(57B)	2264(4)	6198(8)	7118(4)	29(2)
C(58B)	2507(4)	6596(8)	6657(4)	34(3)
C(59B)	2728(4)	7585(9)	6701(4)	38(3)
C(60B)	2716(4)	8185(8)	7196(4)	27(2)
C(61B)	2028(5)	5118(8)	7094(4)	39(3)
F(61D)	2215(3)	4476(6)	7481(3)	79(3)

F(61E)	1561(3)	5160(6)	7221(4)	71(2)
F(61F)	2049(3)	4644(5)	6606(3)	68(2)
C(62B)	2988(6)	8041(11)	6213(5)	55(4)
F(62D)	2992(4)	7422(7)	5762(3)	108(4)
F(62E)	2871(6)	8980(7)	6071(5)	151(7)
F(62F)	3468(4)	8121(12)	6329(4)	134(5)
C(63B)	2899(4)	9260(7)	8378(4)	21(2)
C(64B)	3371(4)	8972(7)	8235(4)	28(2)
C(65B)	3770(3)	9569(7)	8405(4)	24(2)
C(66B)	3726(4)	10466(7)	8738(4)	29(2)
C(67B)	3260(4)	10762(7)	8894(4)	24(2)
C(68B)	2866(4)	10182(8)	8719(4)	26(2)
C(69B)	4260(5)	9200(7)	8221(4)	30(3)
F(69D)	4266(3)	9031(5)	7655(3)	46(2)
F(69E)	4623(2)	9885(5)	8341(3)	48(2)
F(69F)	4391(2)	8252(5)	8449(3)	45(2)
C(70B)	3207(4)	11709(8)	9283(5)	39(3)
F(70D)	2767(3)	11965(7)	9376(4)	87(3)
F(70E)	3405(3)	11495(7)	9797(3)	83(3)
F(70F)	3449(4)	12558(6)	9128(3)	86(3)
C(71B)	1941(4)	9241(7)	8114(4)	27(2)
C(72B)	1478(4)	8868(7)	8241(4)	21(2)
C(73B)	1050(4)	9418(8)	8095(4)	30(2)
C(74B)	1080(4)	10375(8)	7800(4)	35(3)
C(75B)	1520(4)	10753(7)	7657(4)	30(2)
C(76B)	1943(4)	10211(7)	7812(4)	29(2)
C(77B)	586(4)	8966(8)	8264(5)	35(3)
F(77D)	568(3)	8763(6)	8818(3)	47(2)
F(77E)	205(2)	9589(6)	8136(3)	58(2)
F(77F)	486(2)	8015(5)	8007(3)	49(2)
C(78B)	1550(5)	11743(9)	7304(5)	49(3)
F(78D)	1217(4)	12468(6)	7425(3)	91(3)
F(78E)	1488(4)	11552(6)	6767(3)	90(3)
F(78F)	1963(3)	12255(6)	7373(4)	105(4)
C(1X)	7222(6)	8682(13)	9985(6)	35(5)
C(2X)	7557(7)	9177(14)	9599(6)	37(5)
CL1X	6912(2)	7727(4)	9624(2)	27(1)
CL2X	7303(2)	9914(5)	9054(2)	49(1)
O(1Z)	7443(4)	8756(8)	8477(4)	18(2)
C(1Y)	5528(5)	3018(11)	8559(4)	58(3)
C(2Y)	5077(5)	2469(10)	8385(5)	57(4)
CL1Y	5784(1)	2450(3)	9176(1)	71(1)
CL2Y	4604(1)	2674(3)	8875(1)	66(1)

Table S 4: Bond lengths [Å] and angles [°] for W-13.

W(1A)-N(4A)	1.761(8)
W(1A)-N(3A)	1.762(8)
W(1A)-C(1A)	2.207(9)
W(1A)-BR1A	2.4594(11)
N(1A)-C(1A)	1.330(12)
N(1A)-C(5A)	1.442(13)
N(1A)-C(2A)	1.473(12)
C(1A)-N(2A)	1.320(13)
N(2A)-C(14A)	1.443(12)
N(2A)-C(4A)	1.480(12)
C(2A)-C(3A)	1.475(16)
C(2A)-H(2A1)	0.9900
C(2A)-H(2A2)	0.9900
N(3A)-C(23A)	1.383(13)
C(3A)-C(4A)	1.508(16)
C(3A)-H(3A1)	0.9900
C(3A)-H(3A2)	0.9900
N(4A)-C(35A)	1.392(11)
C(4A)-H(4A1)	0.9900
C(4A)-H(4A2)	0.9900
C(5A)-C(6A)	1.391(13)
C(5A)-C(10A)	1.435(14)
C(6A)-C(7A)	1.389(15)
C(6A)-C(11A)	1.519(14)
C(7A)-C(8A)	1.373(15)
C(7A)-H(7A)	0.9500
C(8A)-C(9A)	1.377(15)
C(8A)-C(12A)	1.508(14)
C(9A)-C(10A)	1.372(15)
C(9A)-H(9A)	0.9500
C(10A)-C(13A)	1.526(13)
C(11A)-H(11A)	0.9800
C(11A)-H(11B)	0.9800
C(11A)-H(11C)	0.9800
C(12A)-H(12A)	0.9800
C(12A)-H(12B)	0.9800
C(12A)-H(12C)	0.9800
C(13A)-H(13A)	0.9800
C(13A)-H(13B)	0.9800
C(13A)-H(13C)	0.9800
C(14A)-C(15A)	1.377(16)
C(14A)-C(19A)	1.405(14)

C(15A)-C(16A)	1.399(14)
C(15A)-C(20A)	1.493(13)
C(16A)-C(17A)	1.392(15)
C(16A)-H(16A)	0.9500
C(17A)-C(18A)	1.386(16)
C(17A)-C(21A)	1.509(14)
C(18A)-C(19A)	1.396(14)
C(18A)-H(18A)	0.9500
C(19A)-C(22A)	1.500(15)
C(20A)-H(20A)	0.9800
C(20A)-H(20B)	0.9800
C(20A)-H(20C)	0.9800
C(21A)-H(21A)	0.9800
C(21A)-H(21B)	0.9800
C(21A)-H(21C)	0.9800
C(22A)-H(22A)	0.9800
C(22A)-H(22B)	0.9800
C(22A)-H(22C)	0.9800
C(23A)-C(28A)	1.409(15)
C(23A)-C(24A)	1.420(15)
C(24A)-C(25A)	1.408(17)
C(24A)-C(29A)	1.492(16)
C(25A)-C(26A)	1.398(19)
C(25A)-H(25A)	0.9500
C(26A)-C(27A)	1.386(19)
C(26A)-H(26A)	0.9500
C(27A)-C(28A)	1.402(15)
C(27A)-H(27A)	0.9500
C(28A)-C(32A)	1.513(16)
C(29A)-C(30A)	1.530(16)
C(29A)-C(31A)	1.535(16)
C(29A)-H(29A)	1.000
C(30A)-H(30A)	0.9800
C(30A)-H(30B)	0.9800
C(30A)-H(30C)	0.9800
C(31A)-H(31A)	0.9800
C(31A)-H(31B)	0.9800
C(31A)-H(31C)	0.9800
C(32A)-C(34A)	1.548(15)
C(32A)-C(33A)	1.558(17)
C(32A)-H(32A)	1.000
C(33A)-H(33A)	0.9800
C(33A)-H(33B)	0.9800
C(33A)-H(33C)	0.9800

C(34A)-H(34A)	0.9800
C(34A)-H(34B)	0.9800
C(34A)-H(34C)	0.9800
C(35A)-C(37A)	1.333(13)
C(35A)-C(36A)	1.497(14)
C(36A)-H(36A)	0.9800
C(36A)-H(36B)	0.9800
C(36A)-H(36C)	0.9800
C(37A)-C(38A)	1.517(13)
C(37A)-H(37A)	0.9500
C(38A)-C(41A)	1.539(13)
C(38A)-C(39A)	1.541(15)
C(38A)-C(40A)	1.545(15)
C(39A)-H(39A)	0.9800
C(39A)-H(39B)	0.9800
C(39A)-H(39C)	0.9800
C(40A)-H(40A)	0.9800
C(40A)-H(40B)	0.9800
C(40A)-H(40C)	0.9800
C(41A)-C(46A)	1.383(13)
C(41A)-C(42A)	1.386(14)
C(42A)-C(43A)	1.374(14)
C(42A)-H(42A)	0.9500
C(43A)-C(44A)	1.365(14)
C(43A)-H(43A)	0.9500
C(44A)-C(45A)	1.395(16)
C(44A)-H(44A)	0.9500
C(45A)-C(46A)	1.399(16)
C(45A)-H(45A)	0.9500
C(46A)-H(46A)	0.9500
B(1A)-C(47A)	1.642(13)
B(1A)-C(63A)	1.643(15)
B(1A)-C(71A)	1.645(14)
B(1A)-C(55A)	1.654(13)
C(47A)-C(48A)	1.390(13)
C(47A)-C(52A)	1.397(14)
C(48A)-C(49A)	1.415(13)
C(48A)-H(48A)	0.9500
C(49A)-C(50A)	1.384(14)
C(49A)-C(53A)	1.501(14)
C(50A)-C(51A)	1.374(13)
C(50A)-H(50A)	0.9500
C(51A)-C(52A)	1.383(13)
C(51A)-C(54A)	1.478(14)

C(52A)-H(52A)	0.9500
C(53A)-F(53C)	1.305(12)
C(53A)-F(53A)	1.316(13)
C(53A)-F(53B)	1.347(12)
C(54A)-F(54C)	1.278(13)
C(54A)-F(54B)	1.313(12)
C(54A)-F(54A)	1.333(14)
C(55A)-C(56A)	1.379(13)
C(55A)-C(60A)	1.403(12)
C(56A)-C(57A)	1.365(14)
C(56A)-H(56A)	0.9500
C(57A)-C(58A)	1.386(14)
C(57A)-C(61A)	1.519(15)
C(58A)-C(59A)	1.364(14)
C(58A)-H(58A)	0.9500
C(59A)-C(60A)	1.414(13)
C(59A)-C(62A)	1.496(12)
C(60A)-H(60A)	0.9500
C(61A)-F(61A)	1.328(12)
C(61A)-F(61B)	1.330(11)
C(61A)-F(61C)	1.349(13)
C(62A)-F(62C)	1.320(11)
C(62A)-F(62B)	1.335(12)
C(62A)-F(62A)	1.342(11)
C(63A)-C(64A)	1.383(13)
C(63A)-C(68A)	1.402(13)
C(64A)-C(65A)	1.395(13)
C(64A)-H(64A)	0.9500
C(65A)-C(66A)	1.366(13)
C(65A)-C(69A)	1.509(14)
C(66A)-C(67A)	1.382(14)
C(66A)-H(66A)	0.9500
C(67A)-C(68A)	1.411(14)
C(67A)-C(70A)	1.495(14)
C(68A)-H(68A)	0.9500
C(69A)-F(69A)	1.306(11)
C(69A)-F(69C)	1.312(12)
C(69A)-F(69B)	1.343(13)
C(70A)-F(70A)	1.280(13)
C(70A)-F(70C)	1.282(15)
C(70A)-F(70B)	1.301(15)
C(71A)-C(76A)	1.403(12)
C(71A)-C(72A)	1.415(14)
C(72A)-C(73A)	1.378(14)

C(72A)-H(72A)	0.9500
C(73A)-C(74A)	1.401(13)
C(73A)-C(77A)	1.474(16)
C(74A)-C(75A)	1.371(14)
C(74A)-H(74A)	0.9500
C(75A)-C(76A)	1.370(13)
C(75A)-C(78A)	1.483(14)
C(76A)-H(76A)	0.9500
C(77A)-F(77A)	1.274(14)
C(77A)-F(77C)	1.288(14)
C(77A)-F(77B)	1.313(16)
C(78A)-F(78B)	1.303(11)
C(78A)-F(78A)	1.319(13)
C(78A)-F(78C)	1.373(12)
W(1B)-N(4B)	1.749(8)
W(1B)-N(3B)	1.762(9)
W(1B)-C(1B)	2.193(10)
W(1B)-BR1B	2.4560(12)
N(1B)-C(1B)	1.339(13)
N(1B)-C(5B)	1.448(13)
N(1B)-C(2B)	1.485(12)
C(1B)-N(2B)	1.317(13)
N(2B)-C(14B)	1.447(13)
N(2B)-C(4B)	1.476(12)
C(2B)-C(3B)	1.462(16)
C(2B)-H(2B1)	0.9900
C(2B)-H(2B2)	0.9900
N(3B)-C(23B)	1.402(13)
C(3B)-C(4B)	1.525(16)
C(3B)-H(3B1)	0.9900
C(3B)-H(3B2)	0.9900
N(4B)-C(35B)	1.404(12)
C(4B)-H(4B1)	0.9900
C(4B)-H(4B2)	0.9900
C(5B)-C(6B)	1.399(15)
C(5B)-C(10B)	1.414(14)
C(6B)-C(7B)	1.393(13)
C(6B)-C(11B)	1.515(14)
C(7B)-C(8B)	1.387(14)
C(7B)-H(7B)	0.9500
C(8B)-C(9B)	1.407(16)
C(8B)-C(12B)	1.525(14)
C(9B)-C(10B)	1.361(15)
C(9B)-H(9B)	0.9500

C(10B)-C(13B)	1.503(16)
C(11B)-H(11D)	0.9800
C(11B)-H(11E)	0.9800
C(11B)-H(11F)	0.9800
C(12B)-H(12D)	0.9800
C(12B)-H(12E)	0.9800
C(12B)-H(12F)	0.9800
C(13B)-H(13D)	0.9800
C(13B)-H(13E)	0.9800
C(13B)-H(13F)	0.9800
C(14B)-C(15B)	1.350(15)
C(14B)-C(19B)	1.422(14)
C(15B)-C(16B)	1.402(16)
C(15B)-C(20B)	1.522(14)
C(16B)-C(17B)	1.384(14)
C(16B)-H(16B)	0.9500
C(17B)-C(18B)	1.380(15)
C(17B)-C(21B)	1.508(15)
C(18B)-C(19B)	1.394(15)
C(18B)-H(18B)	0.9500
C(19B)-C(22B)	1.488(16)
C(20B)-H(20D)	0.9800
C(20B)-H(20E)	0.9800
C(20B)-H(20F)	0.9800
C(21B)-H(21D)	0.9800
C(21B)-H(21E)	0.9800
C(21B)-H(21F)	0.9800
C(22B)-H(22D)	0.9800
C(22B)-H(22E)	0.9800
C(22B)-H(22F)	0.9800
C(23B)-C(28B)	1.381(15)
C(23B)-C(24B)	1.409(15)
C(24B)-C(25B)	1.366(16)
C(24B)-C(29B)	1.521(16)
C(25B)-C(26B)	1.344(18)
C(25B)-H(25B)	0.9500
C(26B)-C(27B)	1.396(19)
C(26B)-H(26B)	0.9500
C(27B)-C(28B)	1.370(17)
C(27B)-H(27B)	0.9500
C(28B)-C(32B)	1.508(16)
C(29B)-C(31B)	1.505(16)
C(29B)-C(30B)	1.505(16)
C(29B)-H(29B)	1.000

C(30B)-H(30D)	0.9800
C(30B)-H(30E)	0.9800
C(30B)-H(30F)	0.9800
C(31B)-H(31D)	0.9800
C(31B)-H(31E)	0.9800
C(31B)-H(31F)	0.9800
C(32B)-C(33B)	1.510(16)
C(32B)-C(34B)	1.528(15)
C(32B)-H(32B)	1.000
C(33B)-H(33D)	0.9800
C(33B)-H(33E)	0.9800
C(33B)-H(33F)	0.9800
C(34B)-H(34D)	0.9800
C(34B)-H(34E)	0.9800
C(34B)-H(34F)	0.9800
C(35B)-C(37B)	1.339(13)
C(35B)-C(36B)	1.499(15)
C(36B)-H(36D)	0.9800
C(36B)-H(36E)	0.9800
C(36B)-H(36F)	0.9800
C(37B)-C(38B)	1.519(13)
C(37B)-H(37B)	0.9500
C(38B)-C(41B)	1.522(14)
C(38B)-C(39B)	1.526(15)
C(38B)-C(40B)	1.554(15)
C(39B)-H(39D)	0.9800
C(39B)-H(39E)	0.9800
C(39B)-H(39F)	0.9800
C(40B)-H(40D)	0.9800
C(40B)-H(40E)	0.9800
C(40B)-H(40F)	0.9800
C(41B)-C(42B)	1.373(15)
C(41B)-C(46B)	1.410(15)
C(42B)-C(43B)	1.391(16)
C(42B)-H(42B)	0.9500
C(43B)-C(44B)	1.350(17)
C(43B)-H(43B)	0.9500
C(44B)-C(45B)	1.377(15)
C(44B)-H(44B)	0.9500
C(45B)-C(46B)	1.413(15)
C(45B)-H(45B)	0.9500
C(46B)-H(46B)	0.9500
B(1B)-C(71B)	1.628(16)
B(1B)-C(47B)	1.628(14)

B(1B)-C(63B)	1.630(15)
B(1B)-C(55B)	1.669(14)
C(47B)-C(48B)	1.389(13)
C(47B)-C(52B)	1.412(13)
C(48B)-C(49B)	1.401(13)
C(48B)-H(48B)	0.9500
C(49B)-C(50B)	1.370(13)
C(49B)-C(53B)	1.497(14)
C(50B)-C(51B)	1.383(13)
C(50B)-H(50B)	0.9500
C(51B)-C(52B)	1.373(13)
C(51B)-C(54B)	1.513(14)
C(52B)-H(52B)	0.9500
C(53B)-F(53E)	1.318(12)
C(53B)-F(53F)	1.342(13)
C(53B)-F(53D)	1.347(11)
C(54B)-F(54D)	1.280(13)
C(54B)-F(54E)	1.287(14)
C(54B)-F(54F)	1.323(16)
C(55B)-C(60B)	1.382(13)
C(55B)-C(56B)	1.401(13)
C(56B)-C(57B)	1.373(13)
C(56B)-H(56B)	0.9500
C(57B)-C(58B)	1.392(14)
C(57B)-C(61B)	1.496(14)
C(58B)-C(59B)	1.378(15)
C(58B)-H(58B)	0.9500
C(59B)-C(60B)	1.394(13)
C(59B)-C(62B)	1.492(15)
C(60B)-H(60B)	0.9500
C(61B)-F(61F)	1.304(12)
C(61B)-F(61D)	1.314(12)
C(61B)-F(61E)	1.331(14)
C(62B)-F(62E)	1.258(16)
C(62B)-F(62D)	1.320(14)
C(62B)-F(62F)	1.350(17)
C(63B)-C(64B)	1.398(15)
C(63B)-C(68B)	1.411(13)
C(64B)-C(65B)	1.380(14)
C(64B)-H(64B)	0.9500
C(65B)-C(66B)	1.376(13)
C(65B)-C(69B)	1.503(15)
C(66B)-C(67B)	1.395(14)
C(66B)-H(66B)	0.9500

C(67B)-C(68B)	1.362(14)
C(67B)-C(70B)	1.510(13)
C(68B)-H(68B)	0.9500
C(69B)-F(69E)	1.341(12)
C(69B)-F(69F)	1.346(11)
C(69B)-F(69D)	1.361(12)
C(70B)-F(70D)	1.281(13)
C(70B)-F(70F)	1.309(13)
C(70B)-F(70E)	1.351(13)
C(71B)-C(72B)	1.402(14)
C(71B)-C(76B)	1.407(13)
C(72B)-C(73B)	1.400(14)
C(72B)-H(72B)	0.9500
C(73B)-C(74B)	1.387(14)
C(73B)-C(77B)	1.464(15)
C(74B)-C(75B)	1.353(15)
C(74B)-H(74B)	0.9500
C(75B)-C(76B)	1.391(14)
C(75B)-C(78B)	1.495(14)
C(76B)-H(76B)	0.9500
C(77B)-F(77E)	1.335(13)
C(77B)-F(77D)	1.343(13)
C(77B)-F(77F)	1.359(12)
C(78B)-F(78E)	1.306(13)
C(78B)-F(78F)	1.310(14)
C(78B)-F(78D)	1.326(14)
C(1X)-C(2X)	1.458(12)
C(1X)-CL1X	1.686(11)
C(1X)-H(1X1)	0.9900
C(1X)-H(1X2)	0.9900
C(2X)-CL2X	1.721(11)
C(2X)-H(2X1)	0.9900
C(2X)-H(2X2)	0.9900
C(1Y)-C(2Y)	1.467(17)
C(1Y)-CL1Y	1.760(11)
C(1Y)-H(1Y1)	0.9900
C(1Y)-H(1Y2)	0.9900
C(2Y)-CL2Y	1.790(13)
C(2Y)-H(2Y1)	0.9900
C(2Y)-H(2Y2)	0.9900
N(4A)-W(1A)-N(3A)	114.8(4)
N(4A)-W(1A)-C(1A)	101.0(3)
N(3A)-W(1A)-C(1A)	97.3(4)

N(4A)-W(1A)-BR1A	102.2(2)
N(3A)-W(1A)-BR1A	100.2(3)
C(1A)-W(1A)-BR1A	141.5(3)
C(1A)-N(1A)-C(5A)	115.4(8)
C(1A)-N(1A)-C(2A)	123.6(9)
C(5A)-N(1A)-C(2A)	120.7(8)
N(2A)-C(1A)-N(1A)	120.7(8)
N(2A)-C(1A)-W(1A)	130.3(7)
N(1A)-C(1A)-W(1A)	109.0(7)
C(1A)-N(2A)-C(14A)	121.9(8)
C(1A)-N(2A)-C(4A)	121.3(8)
C(14A)-N(2A)-C(4A)	116.8(8)
N(1A)-C(2A)-C(3A)	108.7(9)
N(1A)-C(2A)-H(2A1)	109.9
C(3A)-C(2A)-H(2A1)	109.9
N(1A)-C(2A)-H(2A2)	109.9
C(3A)-C(2A)-H(2A2)	109.9
H(2A1)-C(2A)-H(2A2)	108.3
C(23A)-N(3A)-W(1A)	170.2(7)
C(2A)-C(3A)-C(4A)	110.3(11)
C(2A)-C(3A)-H(3A1)	109.6
C(4A)-C(3A)-H(3A1)	109.6
C(2A)-C(3A)-H(3A2)	109.6
C(4A)-C(3A)-H(3A2)	109.6
H(3A1)-C(3A)-H(3A2)	108.1
C(35A)-N(4A)-W(1A)	168.3(6)
N(2A)-C(4A)-C(3A)	111.2(9)
N(2A)-C(4A)-H(4A1)	109.4
C(3A)-C(4A)-H(4A1)	109.4
N(2A)-C(4A)-H(4A2)	109.4
C(3A)-C(4A)-H(4A2)	109.4
H(4A1)-C(4A)-H(4A2)	108.0
C(6A)-C(5A)-C(10A)	121.2(9)
C(6A)-C(5A)-N(1A)	120.7(9)
C(10A)-C(5A)-N(1A)	117.6(9)
C(7A)-C(6A)-C(5A)	117.7(9)
C(7A)-C(6A)-C(11A)	121.5(10)
C(5A)-C(6A)-C(11A)	120.6(10)
C(8A)-C(7A)-C(6A)	122.9(10)
C(8A)-C(7A)-H(7A)	118.5
C(6A)-C(7A)-H(7A)	118.5
C(7A)-C(8A)-C(9A)	117.6(9)
C(7A)-C(8A)-C(12A)	121.2(10)
C(9A)-C(8A)-C(12A)	121.1(10)

C(10A)-C(9A)-C(8A)	123.9(10)
C(10A)-C(9A)-H(9A)	118.1
C(8A)-C(9A)-H(9A)	118.1
C(9A)-C(10A)-C(5A)	116.4(9)
C(9A)-C(10A)-C(13A)	121.4(10)
C(5A)-C(10A)-C(13A)	122.0(9)
C(6A)-C(11A)-H(11A)	109.5
C(6A)-C(11A)-H(11B)	109.5
H(11A)-C(11A)-H(11B)	109.5
C(6A)-C(11A)-H(11C)	109.5
H(11A)-C(11A)-H(11C)	109.5
H(11B)-C(11A)-H(11C)	109.5
C(8A)-C(12A)-H(12A)	109.5
C(8A)-C(12A)-H(12B)	109.5
H(12A)-C(12A)-H(12B)	109.5
C(8A)-C(12A)-H(12C)	109.5
H(12A)-C(12A)-H(12C)	109.5
H(12B)-C(12A)-H(12C)	109.5
C(10A)-C(13A)-H(13A)	109.5
C(10A)-C(13A)-H(13B)	109.5
H(13A)-C(13A)-H(13B)	109.5
C(10A)-C(13A)-H(13C)	109.5
H(13A)-C(13A)-H(13C)	109.5
H(13B)-C(13A)-H(13C)	109.5
C(15A)-C(14A)-C(19A)	122.2(9)
C(15A)-C(14A)-N(2A)	120.5(9)
C(19A)-C(14A)-N(2A)	117.0(10)
C(14A)-C(15A)-C(16A)	118.3(9)
C(14A)-C(15A)-C(20A)	121.7(9)
C(16A)-C(15A)-C(20A)	120.0(10)
C(17A)-C(16A)-C(15A)	121.0(10)
C(17A)-C(16A)-H(16A)	119.5
C(15A)-C(16A)-H(16A)	119.5
C(18A)-C(17A)-C(16A)	119.3(10)
C(18A)-C(17A)-C(21A)	119.9(10)
C(16A)-C(17A)-C(21A)	120.8(11)
C(17A)-C(18A)-C(19A)	121.2(9)
C(17A)-C(18A)-H(18A)	119.4
C(19A)-C(18A)-H(18A)	119.4
C(18A)-C(19A)-C(14A)	117.8(10)
C(18A)-C(19A)-C(22A)	119.5(9)
C(14A)-C(19A)-C(22A)	122.7(9)
C(15A)-C(20A)-H(20A)	109.5
C(15A)-C(20A)-H(20B)	109.5

H(20A)-C(20A)-H(20B)	109.5
C(15A)-C(20A)-H(20C)	109.5
H(20A)-C(20A)-H(20C)	109.5
H(20B)-C(20A)-H(20C)	109.5
C(17A)-C(21A)-H(21A)	109.5
C(17A)-C(21A)-H(21B)	109.5
H(21A)-C(21A)-H(21B)	109.5
C(17A)-C(21A)-H(21C)	109.5
H(21A)-C(21A)-H(21C)	109.5
H(21B)-C(21A)-H(21C)	109.5
C(19A)-C(22A)-H(22A)	109.5
C(19A)-C(22A)-H(22B)	109.5
H(22A)-C(22A)-H(22B)	109.5
C(19A)-C(22A)-H(22C)	109.5
H(22A)-C(22A)-H(22C)	109.5
H(22B)-C(22A)-H(22C)	109.5
N(3A)-C(23A)-C(28A)	121.3(9)
N(3A)-C(23A)-C(24A)	117.5(9)
C(28A)-C(23A)-C(24A)	121.2(10)
C(25A)-C(24A)-C(23A)	117.9(11)
C(25A)-C(24A)-C(29A)	121.0(11)
C(23A)-C(24A)-C(29A)	121.1(11)
C(26A)-C(25A)-C(24A)	119.5(12)
C(26A)-C(25A)-H(25A)	120.3
C(24A)-C(25A)-H(25A)	120.3
C(27A)-C(26A)-C(25A)	123.2(11)
C(27A)-C(26A)-H(26A)	118.4
C(25A)-C(26A)-H(26A)	118.4
C(26A)-C(27A)-C(28A)	117.9(11)
C(26A)-C(27A)-H(27A)	121.0
C(28A)-C(27A)-H(27A)	121.0
C(27A)-C(28A)-C(23A)	120.2(11)
C(27A)-C(28A)-C(32A)	120.2(10)
C(23A)-C(28A)-C(32A)	119.4(10)
C(24A)-C(29A)-C(30A)	113.9(11)
C(24A)-C(29A)-C(31A)	109.2(9)
C(30A)-C(29A)-C(31A)	111.9(10)
C(24A)-C(29A)-H(29A)	107.2
C(30A)-C(29A)-H(29A)	107.2
C(31A)-C(29A)-H(29A)	107.2
C(29A)-C(30A)-H(30A)	109.5
C(29A)-C(30A)-H(30B)	109.5
H(30A)-C(30A)-H(30B)	109.5
C(29A)-C(30A)-H(30C)	109.5

H(30A)-C(30A)-H(30C)	109.5
H(30B)-C(30A)-H(30C)	109.5
C(29A)-C(31A)-H(31A)	109.5
C(29A)-C(31A)-H(31B)	109.5
H(31A)-C(31A)-H(31B)	109.5
C(29A)-C(31A)-H(31C)	109.5
H(31A)-C(31A)-H(31C)	109.5
H(31B)-C(31A)-H(31C)	109.5
C(28A)-C(32A)-C(34A)	113.3(12)
C(28A)-C(32A)-C(33A)	110.1(9)
C(34A)-C(32A)-C(33A)	109.9(10)
C(28A)-C(32A)-H(32A)	107.8
C(34A)-C(32A)-H(32A)	107.8
C(33A)-C(32A)-H(32A)	107.8
C(32A)-C(33A)-H(33A)	109.5
C(32A)-C(33A)-H(33B)	109.5
H(33A)-C(33A)-H(33B)	109.5
C(32A)-C(33A)-H(33C)	109.5
H(33A)-C(33A)-H(33C)	109.5
H(33B)-C(33A)-H(33C)	109.5
C(32A)-C(34A)-H(34A)	109.5
C(32A)-C(34A)-H(34B)	109.5
H(34A)-C(34A)-H(34B)	109.5
C(32A)-C(34A)-H(34C)	109.5
H(34A)-C(34A)-H(34C)	109.5
H(34B)-C(34A)-H(34C)	109.5
C(37A)-C(35A)-N(4A)	120.5(9)
C(37A)-C(35A)-C(36A)	127.0(8)
N(4A)-C(35A)-C(36A)	112.5(8)
C(35A)-C(36A)-H(36A)	109.5
C(35A)-C(36A)-H(36B)	109.5
H(36A)-C(36A)-H(36B)	109.5
C(35A)-C(36A)-H(36C)	109.5
H(36A)-C(36A)-H(36C)	109.5
H(36B)-C(36A)-H(36C)	109.5
C(35A)-C(37A)-C(38A)	127.9(10)
C(35A)-C(37A)-H(37A)	116.1
C(38A)-C(37A)-H(37A)	116.1
C(37A)-C(38A)-C(41A)	115.0(8)
C(37A)-C(38A)-C(39A)	107.8(9)
C(41A)-C(38A)-C(39A)	108.0(8)
C(37A)-C(38A)-C(40A)	107.2(8)
C(41A)-C(38A)-C(40A)	110.4(9)
C(39A)-C(38A)-C(40A)	108.2(9)

C(38A)-C(39A)-H(39A)	109.5
C(38A)-C(39A)-H(39B)	109.5
H(39A)-C(39A)-H(39B)	109.5
C(38A)-C(39A)-H(39C)	109.5
H(39A)-C(39A)-H(39C)	109.5
H(39B)-C(39A)-H(39C)	109.5
C(38A)-C(40A)-H(40A)	109.5
C(38A)-C(40A)-H(40B)	109.5
H(40A)-C(40A)-H(40B)	109.5
C(38A)-C(40A)-H(40C)	109.5
H(40A)-C(40A)-H(40C)	109.5
H(40B)-C(40A)-H(40C)	109.5
C(46A)-C(41A)-C(42A)	117.3(9)
C(46A)-C(41A)-C(38A)	120.5(9)
C(42A)-C(41A)-C(38A)	122.0(9)
C(43A)-C(42A)-C(41A)	121.5(9)
C(43A)-C(42A)-H(42A)	119.3
C(41A)-C(42A)-H(42A)	119.3
C(44A)-C(43A)-C(42A)	121.4(10)
C(44A)-C(43A)-H(43A)	119.3
C(42A)-C(43A)-H(43A)	119.3
C(43A)-C(44A)-C(45A)	118.7(10)
C(43A)-C(44A)-H(44A)	120.6
C(45A)-C(44A)-H(44A)	120.6
C(44A)-C(45A)-C(46A)	119.4(11)
C(44A)-C(45A)-H(45A)	120.3
C(46A)-C(45A)-H(45A)	120.3
C(41A)-C(46A)-C(45A)	121.5(10)
C(41A)-C(46A)-H(46A)	119.2
C(45A)-C(46A)-H(46A)	119.2
C(47A)-B(1A)-C(63A)	110.6(8)
C(47A)-B(1A)-C(71A)	109.8(7)
C(63A)-B(1A)-C(71A)	108.2(8)
C(47A)-B(1A)-C(55A)	106.7(8)
C(63A)-B(1A)-C(55A)	112.2(7)
C(71A)-B(1A)-C(55A)	109.3(7)
C(48A)-C(47A)-C(52A)	115.7(8)
C(48A)-C(47A)-B(1A)	122.8(8)
C(52A)-C(47A)-B(1A)	121.4(8)
C(47A)-C(48A)-C(49A)	120.5(9)
C(47A)-C(48A)-H(48A)	119.8
C(49A)-C(48A)-H(48A)	119.8
C(50A)-C(49A)-C(48A)	122.5(9)
C(50A)-C(49A)-C(53A)	122.1(8)

C(48A)-C(49A)-C(53A)	115.4(9)
C(51A)-C(50A)-C(49A)	116.7(9)
C(51A)-C(50A)-H(50A)	121.6
C(49A)-C(50A)-H(50A)	121.6
C(50A)-C(51A)-C(52A)	121.2(9)
C(50A)-C(51A)-C(54A)	119.7(9)
C(52A)-C(51A)-C(54A)	119.1(9)
C(51A)-C(52A)-C(47A)	123.4(9)
C(51A)-C(52A)-H(52A)	118.3
C(47A)-C(52A)-H(52A)	118.3
F(53C)-C(53A)-F(53A)	107.3(10)
F(53C)-C(53A)-F(53B)	105.6(9)
F(53A)-C(53A)-F(53B)	104.2(9)
F(53C)-C(53A)-C(49A)	114.1(8)
F(53A)-C(53A)-C(49A)	113.0(9)
F(53B)-C(53A)-C(49A)	112.0(9)
F(54C)-C(54A)-F(54B)	109.9(11)
F(54C)-C(54A)-F(54A)	102.1(11)
F(54B)-C(54A)-F(54A)	102.5(10)
F(54C)-C(54A)-C(51A)	116.5(9)
F(54B)-C(54A)-C(51A)	113.4(9)
F(54A)-C(54A)-C(51A)	110.9(10)
C(56A)-C(55A)-C(60A)	115.9(8)
C(56A)-C(55A)-B(1A)	121.5(8)
C(60A)-C(55A)-B(1A)	122.6(8)
C(57A)-C(56A)-C(55A)	123.0(9)
C(57A)-C(56A)-H(56A)	118.5
C(55A)-C(56A)-H(56A)	118.5
C(56A)-C(57A)-C(58A)	121.2(10)
C(56A)-C(57A)-C(61A)	119.7(9)
C(58A)-C(57A)-C(61A)	119.0(9)
C(59A)-C(58A)-C(57A)	118.1(9)
C(59A)-C(58A)-H(58A)	120.9
C(57A)-C(58A)-H(58A)	120.9
C(58A)-C(59A)-C(60A)	120.6(8)
C(58A)-C(59A)-C(62A)	119.8(9)
C(60A)-C(59A)-C(62A)	119.6(9)
C(55A)-C(60A)-C(59A)	121.1(9)
C(55A)-C(60A)-H(60A)	119.5
C(59A)-C(60A)-H(60A)	119.5
F(61A)-C(61A)-F(61B)	107.4(9)
F(61A)-C(61A)-F(61C)	104.5(8)
F(61B)-C(61A)-F(61C)	106.0(9)
F(61A)-C(61A)-C(57A)	112.7(9)

F(61B)-C(61A)-C(57A)	114.6(9)
F(61C)-C(61A)-C(57A)	111.0(9)
F(62C)-C(62A)-F(62B)	107.2(8)
F(62C)-C(62A)-F(62A)	107.5(7)
F(62B)-C(62A)-F(62A)	105.5(8)
F(62C)-C(62A)-C(59A)	112.3(9)
F(62B)-C(62A)-C(59A)	112.6(8)
F(62A)-C(62A)-C(59A)	111.5(8)
C(64A)-C(63A)-C(68A)	116.2(9)
C(64A)-C(63A)-B(1A)	124.9(8)
C(68A)-C(63A)-B(1A)	118.9(9)
C(63A)-C(64A)-C(65A)	122.6(9)
C(63A)-C(64A)-H(64A)	118.7
C(65A)-C(64A)-H(64A)	118.7
C(66A)-C(65A)-C(64A)	120.5(9)
C(66A)-C(65A)-C(69A)	120.1(9)
C(64A)-C(65A)-C(69A)	119.3(9)
C(65A)-C(66A)-C(67A)	119.2(9)
C(65A)-C(66A)-H(66A)	120.4
C(67A)-C(66A)-H(66A)	120.4
C(66A)-C(67A)-C(68A)	120.0(9)
C(66A)-C(67A)-C(70A)	120.6(10)
C(68A)-C(67A)-C(70A)	119.3(10)
C(63A)-C(68A)-C(67A)	121.4(10)
C(63A)-C(68A)-H(68A)	119.3
C(67A)-C(68A)-H(68A)	119.3
F(69A)-C(69A)-F(69C)	107.2(9)
F(69A)-C(69A)-F(69B)	105.2(10)
F(69C)-C(69A)-F(69B)	105.4(9)
F(69A)-C(69A)-C(65A)	113.1(9)
F(69C)-C(69A)-C(65A)	113.7(9)
F(69B)-C(69A)-C(65A)	111.6(9)
F(70A)-C(70A)-F(70C)	103.8(13)
F(70A)-C(70A)-F(70B)	107.3(10)
F(70C)-C(70A)-F(70B)	102.8(12)
F(70A)-C(70A)-C(67A)	114.5(10)
F(70C)-C(70A)-C(67A)	113.6(9)
F(70B)-C(70A)-C(67A)	113.7(11)
C(76A)-C(71A)-C(72A)	114.5(8)
C(76A)-C(71A)-B(1A)	120.3(8)
C(72A)-C(71A)-B(1A)	125.2(8)
C(73A)-C(72A)-C(71A)	123.1(9)
C(73A)-C(72A)-H(72A)	118.5
C(71A)-C(72A)-H(72A)	118.5

C(72A)-C(73A)-C(74A)	119.5(9)
C(72A)-C(73A)-C(77A)	119.9(9)
C(74A)-C(73A)-C(77A)	120.6(9)
C(75A)-C(74A)-C(73A)	119.0(9)
C(75A)-C(74A)-H(74A)	120.5
C(73A)-C(74A)-H(74A)	120.5
C(74A)-C(75A)-C(76A)	120.7(9)
C(74A)-C(75A)-C(78A)	119.1(9)
C(76A)-C(75A)-C(78A)	120.2(9)
C(75A)-C(76A)-C(71A)	123.2(9)
C(75A)-C(76A)-H(76A)	118.4
C(71A)-C(76A)-H(76A)	118.4
F(77A)-C(77A)-F(77C)	110.5(13)
F(77A)-C(77A)-F(77B)	103.5(13)
F(77C)-C(77A)-F(77B)	98.2(11)
F(77A)-C(77A)-C(73A)	115.7(10)
F(77C)-C(77A)-C(73A)	114.7(11)
F(77B)-C(77A)-C(73A)	112.4(12)
F(78B)-C(78A)-F(78A)	107.3(9)
F(78B)-C(78A)-F(78C)	105.2(8)
F(78A)-C(78A)-F(78C)	104.3(9)
F(78B)-C(78A)-C(75A)	115.6(9)
F(78A)-C(78A)-C(75A)	113.4(8)
F(78C)-C(78A)-C(75A)	110.2(8)
N(4B)-W(1B)-N(3B)	114.1(4)
N(4B)-W(1B)-C(1B)	101.1(3)
N(3B)-W(1B)-C(1B)	100.1(4)
N(4B)-W(1B)-BR1B	101.6(3)
N(3B)-W(1B)-BR1B	100.0(3)
C(1B)-W(1B)-BR1B	140.1(3)
C(1B)-N(1B)-C(5B)	121.2(8)
C(1B)-N(1B)-C(2B)	122.3(8)
C(5B)-N(1B)-C(2B)	116.0(8)
N(2B)-C(1B)-N(1B)	119.8(9)
N(2B)-C(1B)-W(1B)	110.2(7)
N(1B)-C(1B)-W(1B)	129.9(7)
C(1B)-N(2B)-C(14B)	115.5(8)
C(1B)-N(2B)-C(4B)	124.0(9)
C(14B)-N(2B)-C(4B)	120.2(8)
C(3B)-C(2B)-N(1B)	111.9(9)
C(3B)-C(2B)-H(2B1)	109.2
N(1B)-C(2B)-H(2B1)	109.2
C(3B)-C(2B)-H(2B2)	109.2
N(1B)-C(2B)-H(2B2)	109.2

H(2B1)-C(2B)-H(2B2)	107.9
C(23B)-N(3B)-W(1B)	169.7(7)
C(2B)-C(3B)-C(4B)	111.5(10)
C(2B)-C(3B)-H(3B1)	109.3
C(4B)-C(3B)-H(3B1)	109.3
C(2B)-C(3B)-H(3B2)	109.3
C(4B)-C(3B)-H(3B2)	109.3
H(3B1)-C(3B)-H(3B2)	108.0
C(35B)-N(4B)-W(1B)	168.0(7)
N(2B)-C(4B)-C(3B)	107.0(9)
N(2B)-C(4B)-H(4B1)	110.3
C(3B)-C(4B)-H(4B1)	110.3
N(2B)-C(4B)-H(4B2)	110.3
C(3B)-C(4B)-H(4B2)	110.3
H(4B1)-C(4B)-H(4B2)	108.6
C(6B)-C(5B)-C(10B)	121.8(10)
C(6B)-C(5B)-N(1B)	118.6(9)
C(10B)-C(5B)-N(1B)	119.4(10)
C(7B)-C(6B)-C(5B)	118.1(10)
C(7B)-C(6B)-C(11B)	120.2(10)
C(5B)-C(6B)-C(11B)	121.6(9)
C(8B)-C(7B)-C(6B)	121.4(10)
C(8B)-C(7B)-H(7B)	119.3
C(6B)-C(7B)-H(7B)	119.3
C(7B)-C(8B)-C(9B)	118.3(9)
C(7B)-C(8B)-C(12B)	121.5(10)
C(9B)-C(8B)-C(12B)	120.2(10)
C(10B)-C(9B)-C(8B)	122.7(10)
C(10B)-C(9B)-H(9B)	118.7
C(8B)-C(9B)-H(9B)	118.7
C(9B)-C(10B)-C(5B)	117.6(10)
C(9B)-C(10B)-C(13B)	122.5(10)
C(5B)-C(10B)-C(13B)	119.9(10)
C(6B)-C(11B)-H(11D)	109.5
C(6B)-C(11B)-H(11E)	109.5
H(11D)-C(11B)-H(11E)	109.5
C(6B)-C(11B)-H(11F)	109.5
H(11D)-C(11B)-H(11F)	109.5
H(11E)-C(11B)-H(11F)	109.5
C(8B)-C(12B)-H(12D)	109.5
C(8B)-C(12B)-H(12E)	109.5
H(12D)-C(12B)-H(12E)	109.5
C(8B)-C(12B)-H(12F)	109.5
H(12D)-C(12B)-H(12F)	109.5

H(12E)-C(12B)-H(12F)	109.5
C(10B)-C(13B)-H(13D)	109.5
C(10B)-C(13B)-H(13E)	109.5
H(13D)-C(13B)-H(13E)	109.5
C(10B)-C(13B)-H(13F)	109.5
H(13D)-C(13B)-H(13F)	109.5
H(13E)-C(13B)-H(13F)	109.5
C(15B)-C(14B)-C(19B)	121.6(10)
C(15B)-C(14B)-N(2B)	121.1(9)
C(19B)-C(14B)-N(2B)	116.7(9)
C(14B)-C(15B)-C(16B)	120.1(10)
C(14B)-C(15B)-C(20B)	122.2(10)
C(16B)-C(15B)-C(20B)	117.5(11)
C(17B)-C(16B)-C(15B)	120.5(11)
C(17B)-C(16B)-H(16B)	119.8
C(15B)-C(16B)-H(16B)	119.8
C(18B)-C(17B)-C(16B)	118.3(10)
C(18B)-C(17B)-C(21B)	121.7(10)
C(16B)-C(17B)-C(21B)	120.0(10)
C(17B)-C(18B)-C(19B)	123.1(10)
C(17B)-C(18B)-H(18B)	118.5
C(19B)-C(18B)-H(18B)	118.5
C(18B)-C(19B)-C(14B)	116.3(10)
C(18B)-C(19B)-C(22B)	121.0(10)
C(14B)-C(19B)-C(22B)	122.6(10)
C(15B)-C(20B)-H(20D)	109.5
C(15B)-C(20B)-H(20E)	109.5
H(20D)-C(20B)-H(20E)	109.5
C(15B)-C(20B)-H(20F)	109.5
H(20D)-C(20B)-H(20F)	109.5
H(20E)-C(20B)-H(20F)	109.5
C(17B)-C(21B)-H(21D)	109.5
C(17B)-C(21B)-H(21E)	109.5
H(21D)-C(21B)-H(21E)	109.5
C(17B)-C(21B)-H(21F)	109.5
H(21D)-C(21B)-H(21F)	109.5
H(21E)-C(21B)-H(21F)	109.5
C(19B)-C(22B)-H(22D)	109.5
C(19B)-C(22B)-H(22E)	109.5
H(22D)-C(22B)-H(22E)	109.5
C(19B)-C(22B)-H(22F)	109.5
H(22D)-C(22B)-H(22F)	109.5
H(22E)-C(22B)-H(22F)	109.5
C(28B)-C(23B)-N(3B)	120.9(9)

C(28B)-C(23B)-C(24B)	122.8(10)
N(3B)-C(23B)-C(24B)	116.3(9)
C(25B)-C(24B)-C(23B)	117.1(11)
C(25B)-C(24B)-C(29B)	121.3(11)
C(23B)-C(24B)-C(29B)	121.5(10)
C(26B)-C(25B)-C(24B)	122.0(12)
C(26B)-C(25B)-H(25B)	119.0
C(24B)-C(25B)-H(25B)	119.0
C(25B)-C(26B)-C(27B)	119.8(12)
C(25B)-C(26B)-H(26B)	120.1
C(27B)-C(26B)-H(26B)	120.1
C(28B)-C(27B)-C(26B)	121.6(12)
C(28B)-C(27B)-H(27B)	119.2
C(26B)-C(27B)-H(27B)	119.2
C(27B)-C(28B)-C(23B)	116.7(11)
C(27B)-C(28B)-C(32B)	122.0(11)
C(23B)-C(28B)-C(32B)	121.0(10)
C(31B)-C(29B)-C(30B)	108.6(10)
C(31B)-C(29B)-C(24B)	114.7(11)
C(30B)-C(29B)-C(24B)	112.3(10)
C(31B)-C(29B)-H(29B)	107.0
C(30B)-C(29B)-H(29B)	107.0
C(24B)-C(29B)-H(29B)	107.0
C(29B)-C(30B)-H(30D)	109.5
C(29B)-C(30B)-H(30E)	109.5
H(30D)-C(30B)-H(30E)	109.5
C(29B)-C(30B)-H(30F)	109.5
H(30D)-C(30B)-H(30F)	109.5
H(30E)-C(30B)-H(30F)	109.5
C(29B)-C(31B)-H(31D)	109.5
C(29B)-C(31B)-H(31E)	109.5
H(31D)-C(31B)-H(31E)	109.5
C(29B)-C(31B)-H(31F)	109.5
H(31D)-C(31B)-H(31F)	109.5
H(31E)-C(31B)-H(31F)	109.5
C(28B)-C(32B)-C(33B)	110.7(9)
C(28B)-C(32B)-C(34B)	115.0(10)
C(33B)-C(32B)-C(34B)	109.5(10)
C(28B)-C(32B)-H(32B)	107.0
C(33B)-C(32B)-H(32B)	107.0
C(34B)-C(32B)-H(32B)	107.0
C(32B)-C(33B)-H(33D)	109.5
C(32B)-C(33B)-H(33E)	109.5
H(33D)-C(33B)-H(33E)	109.5

C(32B)-C(33B)-H(33F)	109.5
H(33D)-C(33B)-H(33F)	109.5
H(33E)-C(33B)-H(33F)	109.5
C(32B)-C(34B)-H(34D)	109.5
C(32B)-C(34B)-H(34E)	109.5
H(34D)-C(34B)-H(34E)	109.5
C(32B)-C(34B)-H(34F)	109.5
H(34D)-C(34B)-H(34F)	109.5
H(34E)-C(34B)-H(34F)	109.5
C(37B)-C(35B)-N(4B)	121.4(10)
C(37B)-C(35B)-C(36B)	128.5(9)
N(4B)-C(35B)-C(36B)	110.1(9)
C(35B)-C(36B)-H(36D)	109.5
C(35B)-C(36B)-H(36E)	109.5
H(36D)-C(36B)-H(36E)	109.5
C(35B)-C(36B)-H(36F)	109.5
H(36D)-C(36B)-H(36F)	109.5
H(36E)-C(36B)-H(36F)	109.5
C(35B)-C(37B)-C(38B)	128.7(10)
C(35B)-C(37B)-H(37B)	115.6
C(38B)-C(37B)-H(37B)	115.6
C(37B)-C(38B)-C(41B)	113.4(9)
C(37B)-C(38B)-C(39B)	109.0(9)
C(41B)-C(38B)-C(39B)	106.2(9)
C(37B)-C(38B)-C(40B)	106.7(8)
C(41B)-C(38B)-C(40B)	112.8(9)
C(39B)-C(38B)-C(40B)	108.6(9)
C(38B)-C(39B)-H(39D)	109.5
C(38B)-C(39B)-H(39E)	109.5
H(39D)-C(39B)-H(39E)	109.5
C(38B)-C(39B)-H(39F)	109.5
H(39D)-C(39B)-H(39F)	109.5
H(39E)-C(39B)-H(39F)	109.5
C(38B)-C(40B)-H(40D)	109.5
C(38B)-C(40B)-H(40E)	109.5
H(40D)-C(40B)-H(40E)	109.5
C(38B)-C(40B)-H(40F)	109.5
H(40D)-C(40B)-H(40F)	109.5
H(40E)-C(40B)-H(40F)	109.5
C(42B)-C(41B)-C(46B)	117.3(10)
C(42B)-C(41B)-C(38B)	120.6(10)
C(46B)-C(41B)-C(38B)	122.1(9)
C(41B)-C(42B)-C(43B)	122.2(11)
C(41B)-C(42B)-H(42B)	118.9

C(43B)-C(42B)-H(42B)	118.9
C(44B)-C(43B)-C(42B)	120.5(11)
C(44B)-C(43B)-H(43B)	119.7
C(42B)-C(43B)-H(43B)	119.7
C(43B)-C(44B)-C(45B)	119.7(11)
C(43B)-C(44B)-H(44B)	120.2
C(45B)-C(44B)-H(44B)	120.2
C(44B)-C(45B)-C(46B)	120.5(11)
C(44B)-C(45B)-H(45B)	119.8
C(46B)-C(45B)-H(45B)	119.8
C(41B)-C(46B)-C(45B)	119.6(10)
C(41B)-C(46B)-H(46B)	120.2
C(45B)-C(46B)-H(46B)	120.2
C(71B)-B(1B)-C(47B)	113.7(9)
C(71B)-B(1B)-C(63B)	111.8(7)
C(47B)-B(1B)-C(63B)	103.6(8)
C(71B)-B(1B)-C(55B)	103.5(8)
C(47B)-B(1B)-C(55B)	112.4(7)
C(63B)-B(1B)-C(55B)	112.0(9)
C(48B)-C(47B)-C(52B)	115.0(8)
C(48B)-C(47B)-B(1B)	122.2(8)
C(52B)-C(47B)-B(1B)	122.5(8)
C(47B)-C(48B)-C(49B)	122.4(9)
C(47B)-C(48B)-H(48B)	118.8
C(49B)-C(48B)-H(48B)	118.8
C(50B)-C(49B)-C(48B)	120.9(9)
C(50B)-C(49B)-C(53B)	121.7(8)
C(48B)-C(49B)-C(53B)	117.3(8)
C(49B)-C(50B)-C(51B)	117.9(9)
C(49B)-C(50B)-H(50B)	121.0
C(51B)-C(50B)-H(50B)	121.0
C(52B)-C(51B)-C(50B)	121.4(9)
C(52B)-C(51B)-C(54B)	119.0(9)
C(50B)-C(51B)-C(54B)	119.6(9)
C(51B)-C(52B)-C(47B)	122.3(9)
C(51B)-C(52B)-H(52B)	118.8
C(47B)-C(52B)-H(52B)	118.8
F(53E)-C(53B)-F(53F)	106.1(9)
F(53E)-C(53B)-F(53D)	107.5(8)
F(53F)-C(53B)-F(53D)	103.8(8)
F(53E)-C(53B)-C(49B)	113.5(9)
F(53F)-C(53B)-C(49B)	112.8(8)
F(53D)-C(53B)-C(49B)	112.4(9)
F(54D)-C(54B)-F(54E)	112.6(12)

F(54D)-C(54B)-F(54F)	102.3(11)
F(54E)-C(54B)-F(54F)	102.6(12)
F(54D)-C(54B)-C(51B)	113.6(10)
F(54E)-C(54B)-C(51B)	113.3(10)
F(54F)-C(54B)-C(51B)	111.2(10)
C(60B)-C(55B)-C(56B)	117.0(8)
C(60B)-C(55B)-B(1B)	122.0(8)
C(56B)-C(55B)-B(1B)	120.5(8)
C(57B)-C(56B)-C(55B)	122.9(9)
C(57B)-C(56B)-H(56B)	118.6
C(55B)-C(56B)-H(56B)	118.6
C(56B)-C(57B)-C(58B)	119.2(9)
C(56B)-C(57B)-C(61B)	120.2(9)
C(58B)-C(57B)-C(61B)	120.6(9)
C(59B)-C(58B)-C(57B)	118.6(9)
C(59B)-C(58B)-H(58B)	120.7
C(57B)-C(58B)-H(58B)	120.7
C(58B)-C(59B)-C(60B)	121.7(9)
C(58B)-C(59B)-C(62B)	120.1(10)
C(60B)-C(59B)-C(62B)	118.3(10)
C(55B)-C(60B)-C(59B)	120.3(9)
C(55B)-C(60B)-H(60B)	119.8
C(59B)-C(60B)-H(60B)	119.8
F(61F)-C(61B)-F(61D)	108.7(9)
F(61F)-C(61B)-F(61E)	106.6(10)
F(61D)-C(61B)-F(61E)	103.3(10)
F(61F)-C(61B)-C(57B)	114.4(9)
F(61D)-C(61B)-C(57B)	111.0(9)
F(61E)-C(61B)-C(57B)	112.2(9)
F(62E)-C(62B)-F(62D)	109.5(12)
F(62E)-C(62B)-F(62F)	103.3(13)
F(62D)-C(62B)-F(62F)	100.3(12)
F(62E)-C(62B)-C(59B)	116.0(12)
F(62D)-C(62B)-C(59B)	115.0(11)
F(62F)-C(62B)-C(59B)	111.0(11)
C(64B)-C(63B)-C(68B)	115.1(9)
C(64B)-C(63B)-B(1B)	123.8(8)
C(68B)-C(63B)-B(1B)	120.6(9)
C(65B)-C(64B)-C(63B)	122.0(9)
C(65B)-C(64B)-H(64B)	119.0
C(63B)-C(64B)-H(64B)	119.0
C(66B)-C(65B)-C(64B)	121.6(9)
C(66B)-C(65B)-C(69B)	120.6(9)
C(64B)-C(65B)-C(69B)	117.7(9)

C(65B)-C(66B)-C(67B)	117.6(9)
C(65B)-C(66B)-H(66B)	121.2
C(67B)-C(66B)-H(66B)	121.2
C(68B)-C(67B)-C(66B)	120.7(9)
C(68B)-C(67B)-C(70B)	120.9(9)
C(66B)-C(67B)-C(70B)	118.3(9)
C(67B)-C(68B)-C(63B)	122.9(10)
C(67B)-C(68B)-H(68B)	118.5
C(63B)-C(68B)-H(68B)	118.5
F(69E)-C(69B)-F(69F)	106.4(9)
F(69E)-C(69B)-F(69D)	106.2(9)
F(69F)-C(69B)-F(69D)	104.5(7)
F(69E)-C(69B)-C(65B)	114.5(8)
F(69F)-C(69B)-C(65B)	112.7(9)
F(69D)-C(69B)-C(65B)	111.7(9)
F(70D)-C(70B)-F(70F)	109.8(10)
F(70D)-C(70B)-F(70E)	104.8(10)
F(70F)-C(70B)-F(70E)	102.6(9)
F(70D)-C(70B)-C(67B)	114.0(9)
F(70F)-C(70B)-C(67B)	113.7(9)
F(70E)-C(70B)-C(67B)	110.8(9)
C(72B)-C(71B)-C(76B)	114.3(10)
C(72B)-C(71B)-B(1B)	121.3(8)
C(76B)-C(71B)-B(1B)	124.0(10)
C(73B)-C(72B)-C(71B)	123.4(9)
C(73B)-C(72B)-H(72B)	118.3
C(71B)-C(72B)-H(72B)	118.3
C(74B)-C(73B)-C(72B)	119.1(10)
C(74B)-C(73B)-C(77B)	122.2(10)
C(72B)-C(73B)-C(77B)	118.7(9)
C(75B)-C(74B)-C(73B)	119.5(10)
C(75B)-C(74B)-H(74B)	120.2
C(73B)-C(74B)-H(74B)	120.2
C(74B)-C(75B)-C(76B)	121.1(9)
C(74B)-C(75B)-C(78B)	119.6(10)
C(76B)-C(75B)-C(78B)	119.3(10)
C(75B)-C(76B)-C(71B)	122.6(10)
C(75B)-C(76B)-H(76B)	118.7
C(71B)-C(76B)-H(76B)	118.7
F(77E)-C(77B)-F(77D)	106.5(9)
F(77E)-C(77B)-F(77F)	104.9(10)
F(77D)-C(77B)-F(77F)	105.3(9)
F(77E)-C(77B)-C(73B)	113.7(9)
F(77D)-C(77B)-C(73B)	113.3(10)

F(77F)-C(77B)-C(73B)	112.4(9)
F(78E)-C(78B)-F(78F)	107.6(12)
F(78E)-C(78B)-F(78D)	105.2(10)
F(78F)-C(78B)-F(78D)	104.2(10)
F(78E)-C(78B)-C(75B)	112.9(10)
F(78F)-C(78B)-C(75B)	113.2(9)
F(78D)-C(78B)-C(75B)	113.0(11)
C(2X)-C(1X)-CL1X	107.5(12)
C(2X)-C(1X)-H(1X1)	110.2
CL1X-C(1X)-H(1X1)	110.2
C(2X)-C(1X)-H(1X2)	110.2
CL1X-C(1X)-H(1X2)	110.2
H(1X1)-C(1X)-H(1X2)	108.5
C(1X)-C(2X)-CL2X	116.5(13)
C(1X)-C(2X)-H(2X1)	108.2
CL2X-C(2X)-H(2X1)	108.2
C(1X)-C(2X)-H(2X2)	108.2
CL2X-C(2X)-H(2X2)	108.2
H(2X1)-C(2X)-H(2X2)	107.3
C(2Y)-C(1Y)-CL1Y	111.5(9)
C(2Y)-C(1Y)-H(1Y1)	109.3
CL1Y-C(1Y)-H(1Y1)	109.3
C(2Y)-C(1Y)-H(1Y2)	109.3
CL1Y-C(1Y)-H(1Y2)	109.3
H(1Y1)-C(1Y)-H(1Y2)	108.0
C(1Y)-C(2Y)-CL2Y	112.1(9)
C(1Y)-C(2Y)-H(2Y1)	109.2
CL2Y-C(2Y)-H(2Y1)	109.2
C(1Y)-C(2Y)-H(2Y2)	109.2
CL2Y-C(2Y)-H(2Y2)	109.2
H(2Y1)-C(2Y)-H(2Y2)	107.9

Table S 5: Anisotropic displacement parameters (Å^2 x 10^3) for W-13. The anisotropic displacement factor exponent takes the form: $-2\pi^2$ [h^2 a^{*2} U_{11} + ... + 2 h k a* b* U_{12}].

	U11	U22	U33	U23	U13	U12
W(1A)	26(1)	19(1)	19(1)	0(1)	0(1)	0(1)
BR1A	38(1)	33(1)	26(1)	6(1)	-8(1)	-4(1)
N(1A)	29(5)	20(4)	37(5)	-1(3)	-6(4)	6(4)
C(1A)	34(6)	24(5)	19(5)	7(4)	-3(4)	-2(4)
N(2A)	31(5)	22(4)	28(4)	1(3)	-4(4)	4(3)
C(2A)	34(7)	26(6)	56(7)	13(5)	-11(5)	-1(5)
N(3A)	25(5)	28(4)	23(4)	1(3)	4(3)	2(4)
C(3A)	50(8)	38(7)	75(9)	15(6)	-29(7)	-3(6)

N(4A)	33(5)	25(4)	15(4)	8(3)	-6(3)	0(3)
C(4A)	47(7)	34(6)	42(6)	3(5)	-24(5)	10(5)
C(5A)	30(6)	15(5)	35(5)	10(4)	-4(4)	-3(4)
C(6A)	32(7)	29(6)	30(5)	0(4)	9(5)	-5(4)
C(7A)	46(7)	29(6)	32(5)	-1(4)	-17(5)	-15(5)
C(8A)	25(5)	33(6)	43(6)	6(5)	-6(4)	-7(5)
C(9A)	31(6)	23(5)	46(6)	7(5)	9(5)	0(5)
C(10A)	28(6)	21(5)	33(6)	0(4)	8(5)	0(4)
C(11A)	45(8)	47(7)	59(8)	-22(6)	11(6)	-8(6)
C(12A)	41(7)	69(9)	51(7)	6(6)	-21(6)	-9(6)
C(13A)	35(6)	34(6)	26(5)	3(4)	0(4)	-6(5)
C(14A)	28(6)	27(6)	28(6)	-2(4)	-16(5)	3(4)
C(15A)	35(7)	31(6)	23(5)	-3(4)	-9(5)	6(5)
C(16A)	30(7)	37(6)	30(5)	1(4)	-6(5)	0(5)
C(17A)	45(8)	27(6)	37(6)	6(4)	-15(5)	2(5)
C(18A)	37(7)	37(6)	22(5)	-5(4)	-5(5)	3(5)
C(19A)	38(7)	25(5)	29(5)	5(4)	-13(5)	1(4)
C(20A)	41(7)	38(6)	26(5)	-11(4)	-5(5)	3(5)
C(21A)	52(8)	43(7)	46(7)	-5(5)	-21(6)	-5(6)
C(22A)	50(8)	31(6)	30(6)	-4(4)	-3(5)	1(5)
C(23A)	40(7)	30(6)	30(6)	7(4)	-1(5)	6(5)
C(24A)	32(7)	54(7)	28(6)	12(5)	-7(5)	5(5)
C(25A)	29(7)	85(10)	43(7)	10(6)	11(5)	0(7)
C(26A)	25(7)	115(13)	54(8)	34(8)	3(6)	-24(7)
C(27A)	31(7)	69(9)	48(7)	17(6)	1(5)	-32(6)
C(28A)	47(7)	48(7)	17(5)	6(4)	2(5)	-14(5)
C(29A)	36(7)	53(7)	30(6)	2(5)	-1(5)	6(5)
C(30A)	64(11)	74(10)	57(9)	-17(7)	-6(8)	25(7)
C(31A)	71(10)	58(8)	51(8)	1(6)	-19(7)	7(7)
C(32A)	77(9)	30(6)	22(5)	2(4)	-11(5)	-10(6)
C(33A)	64(9)	39(7)	43(7)	0(5)	15(6)	-8(6)
C(34A)	126(14)	49(8)	51(8)	3(6)	-10(8)	-47(9)
C(35A)	34(6)	15(5)	19(5)	-2(3)	4(4)	10(4)
C(36A)	44(7)	22(5)	37(6)	-15(4)	1(5)	-1(5)
C(37A)	32(6)	12(5)	32(5)	3(4)	-2(4)	3(4)
C(38A)	48(7)	20(5)	30(5)	-13(4)	-14(5)	2(5)
C(39A)	39(7)	35(6)	58(8)	-18(5)	-19(6)	12(5)
C(40A)	73(9)	32(6)	28(6)	0(4)	-5(6)	17(6)
C(41A)	34(6)	15(5)	32(5)	-2(4)	-6(4)	2(4)
C(42A)	44(7)	27(6)	30(5)	0(4)	3(5)	-3(5)
C(43A)	33(6)	28(6)	50(6)	3(5)	10(5)	2(5)
C(44A)	38(7)	29(6)	50(7)	12(5)	-9(5)	-2(5)
C(45A)	44(8)	27(6)	49(7)	4(5)	-7(6)	-1(5)
C(46A)	56(8)	33(6)	23(5)	-7(4)	2(5)	-4(5)

B(1A)	38(7)	28(6)	20(5)	-3(4)	1(5)	4(5)
C(47A)	31(5)	27(5)	22(5)	-4(4)	3(4)	-6(4)
C(48A)	26(6)	25(5)	27(5)	1(4)	-4(4)	1(4)
C(49A)	27(5)	38(6)	33(5)	-8(4)	8(4)	-8(4)
C(50A)	32(6)	28(6)	40(5)	-11(4)	4(4)	-4(4)
C(51A)	31(6)	23(5)	43(6)	-3(4)	6(4)	-7(4)
C(52A)	27(6)	29(6)	28(5)	-1(4)	6(4)	0(4)
C(53A)	35(6)	55(7)	35(6)	-19(5)	-3(5)	2(5)
F(53A)	60(5)	105(6)	42(4)	19(4)	-8(3)	-22(4)
F(53B)	112(7)	69(5)	56(5)	-42(4)	-38(4)	21(5)
F(53C)	36(4)	99(6)	44(4)	-10(4)	-5(3)	22(4)
C(54A)	57(8)	28(6)	47(6)	-11(5)	-8(6)	4(5)
F(54A)	101(7)	74(6)	130(8)	47(5)	29(6)	23(5)
F(54B)	187(11)	73(6)	65(5)	4(4)	33(6)	70(6)
F(54C)	125(9)	60(5)	176(10)	-38(6)	-96(8)	37(6)
C(55A)	34(6)	23(5)	29(5)	1(4)	-1(4)	3(4)
C(56A)	43(6)	31(6)	13(4)	2(4)	-7(4)	11(5)
C(57A)	29(6)	41(7)	29(6)	-2(4)	-1(5)	4(5)
C(58A)	36(6)	44(6)	15(4)	3(4)	-4(4)	6(5)
C(59A)	34(6)	29(5)	22(5)	-9(4)	-8(4)	11(4)
C(60A)	34(6)	30(6)	21(4)	-3(4)	3(4)	6(4)
C(61A)	52(7)	49(7)	24(5)	0(5)	1(5)	-2(6)
F(61A)	45(4)	67(5)	43(3)	1(3)	-14(3)	-11(3)
F(61B)	76(5)	91(6)	39(4)	-8(3)	24(3)	-42(4)
F(61C)	79(5)	40(4)	77(5)	1(3)	-13(4)	-8(4)
C(62A)	44(6)	36(6)	20(5)	-3(4)	-6(4)	1(5)
F(62A)	45(4)	53(4)	32(3)	-5(3)	-10(3)	4(3)
F(62B)	104(6)	40(4)	32(3)	-3(3)	-9(3)	-8(4)
F(62C)	57(4)	73(5)	23(3)	-11(3)	1(3)	15(3)
C(63A)	38(6)	25(5)	19(4)	5(4)	3(4)	-4(4)
C(64A)	38(6)	28(5)	20(4)	2(4)	-2(4)	1(4)
C(65A)	30(5)	35(6)	28(5)	7(4)	6(4)	1(4)
C(66A)	31(5)	33(6)	25(5)	-2(4)	3(4)	-10(4)
C(67A)	36(6)	40(6)	18(5)	-5(4)	-5(4)	-6(5)
C(68A)	43(6)	30(5)	19(4)	-1(4)	2(4)	1(5)
C(69A)	44(7)	44(7)	36(6)	6(5)	3(5)	5(5)
F(69A)	103(7)	114(7)	85(5)	61(5)	71(5)	64(6)
F(69B)	43(5)	75(5)	111(6)	-28(5)	-17(4)	9(4)
F(69C)	45(4)	41(4)	80(5)	-15(3)	-3(3)	8(3)
C(70A)	58(8)	42(7)	46(6)	-16(5)	22(6)	-14(6)
F(70A)	72(6)	113(7)	152(8)	-102(6)	49(6)	-34(5)
F(70B)	52(5)	113(7)	167(9)	-97(7)	-15(5)	-10(5)
F(70C)	338(19)	41(5)	73(6)	-21(4)	8(8)	-9(8)
C(71A)	32(5)	22(5)	19(4)	-7(3)	-1(4)	-2(4)

C(72A)	45(6)	32(6)	26(5)	7(4)	-2(4)	-3(5)
C(73A)	30(6)	49(7)	32(5)	6(5)	-2(4)	10(5)
C(74A)	42(6)	32(6)	38(5)	12(4)	-7(5)	-4(5)
C(75A)	35(6)	36(6)	33(5)	-4(4)	3(5)	4(5)
C(76A)	39(6)	29(6)	25(5)	-3(4)	-2(4)	0(4)
C(77A)	45(8)	64(9)	68(8)	35(7)	10(6)	5(7)
F(77A)	77(7)	141(9)	184(11)	110(8)	79(7)	71(7)
F(77B)	40(5)	131(9)	246(15)	-27(9)	25(7)	-22(6)
F(77C)	70(6)	226(13)	63(6)	33(7)	28(5)	63(7)
C(78A)	45(7)	27(6)	38(6)	7(4)	2(5)	2(5)
F(78A)	47(4)	67(5)	87(5)	42(4)	17(4)	0(4)
F(78B)	48(4)	41(4)	58(4)	19(3)	7(3)	4(3)
F(78C)	130(7)	61(5)	33(3)	9(3)	21(4)	-16(5)
W(1B)	28(1)	23(1)	20(1)	-1(1)	-1(1)	0(1)
BR1B	42(1)	36(1)	32(1)	7(1)	-13(1)	-7(1)
N(1B)	31(5)	27(5)	29(4)	2(3)	-2(4)	2(4)
C(1B)	31(6)	35(6)	20(5)	2(4)	6(5)	-4(5)
N(2B)	30(5)	20(4)	41(5)	7(4)	-6(4)	-3(4)
C(2B)	33(6)	27(6)	48(7)	7(5)	-3(5)	0(5)
N(3B)	47(6)	22(4)	20(4)	-1(3)	-10(4)	1(4)
C(3B)	39(8)	39(7)	74(9)	17(6)	-19(6)	3(5)
N(4B)	25(5)	26(4)	30(4)	6(3)	-4(4)	-5(3)
C(4B)	59(9)	27(6)	53(7)	11(5)	-13(6)	-5(5)
C(5B)	50(8)	24(5)	17(5)	5(4)	-4(5)	2(5)
C(6B)	21(6)	28(5)	28(5)	4(4)	-10(5)	-1(4)
C(7B)	28(6)	35(6)	35(6)	0(4)	-10(5)	-3(5)
C(8B)	26(6)	32(6)	43(6)	-1(5)	-18(5)	3(4)
C(9B)	44(7)	32(6)	28(5)	-14(4)	-17(5)	9(5)
C(10B)	37(7)	36(6)	25(5)	-1(4)	-9(5)	11(5)
C(11B)	34(7)	36(6)	32(5)	-6(4)	-2(5)	2(5)
C(12B)	51(9)	39(7)	63(8)	-20(6)	-17(6)	-4(6)
C(13B)	49(8)	50(7)	27(6)	-1(4)	6(6)	12(5)
C(14B)	34(6)	24(5)	24(5)	6(4)	2(4)	-7(4)
C(15B)	47(8)	25(6)	27(6)	6(4)	-1(5)	-2(5)
C(16B)	28(6)	36(6)	38(6)	7(5)	0(5)	0(5)
C(17B)	35(6)	30(6)	35(5)	8(4)	-5(5)	-6(5)
C(18B)	57(8)	29(6)	24(5)	0(4)	7(5)	-11(5)
C(19B)	30(6)	25(5)	43(6)	5(5)	-1(5)	1(5)
C(20B)	57(8)	44(7)	28(5)	-2(5)	13(5)	-1(6)
C(21B)	58(8)	55(8)	45(7)	9(5)	-8(6)	-14(6)
C(22B)	62(9)	44(7)	48(7)	-3(5)	10(6)	4(6)
C(23B)	20(6)	48(6)	23(5)	6(4)	-2(4)	-4(4)
C(24B)	28(7)	59(8)	29(6)	4(5)	11(5)	6(5)
C(25B)	50(8)	64(9)	36(6)	9(5)	10(6)	6(6)

C(26B)	54(9)	84(11)	39(7)	13(6)	16(6)	-9(8)
C(27B)	71(10)	54(8)	47(7)	11(6)	-11(7)	-14(7)
C(28B)	40(7)	50(7)	21(5)	12(4)	-2(5)	-9(5)
C(29B)	51(8)	46(7)	25(5)	-5(5)	-1(5)	14(6)
C(30B)	69(10)	74(9)	34(7)	-8(6)	-20(7)	5(7)
C(31B)	62(11)	66(9)	56(8)	-12(6)	5(7)	21(7)
C(32B)	50(7)	37(6)	30(6)	2(4)	-2(5)	-6(5)
C(33B)	51(8)	35(6)	43(7)	4(5)	-2(6)	-4(6)
C(34B)	74(9)	49(7)	37(7)	2(5)	-11(6)	-22(7)
C(35B)	34(6)	24(5)	43(6)	-6(4)	6(5)	3(4)
C(36B)	24(7)	49(8)	93(10)	-35(7)	5(6)	1(5)
C(37B)	42(7)	21(5)	22(5)	-3(4)	-5(5)	1(4)
C(38B)	35(6)	33(6)	36(6)	-7(4)	-5(5)	11(5)
C(39B)	40(7)	47(7)	52(7)	-17(5)	-14(6)	2(6)
C(40B)	57(8)	38(6)	28(5)	-3(5)	-5(5)	12(6)
C(41B)	39(7)	31(6)	35(6)	-8(4)	-9(5)	1(5)
C(42B)	38(7)	31(6)	50(7)	-8(5)	-3(5)	-6(5)
C(43B)	53(9)	43(7)	38(7)	6(5)	-13(6)	-14(6)
C(44B)	28(6)	34(6)	72(8)	2(6)	-3(6)	10(5)
C(45B)	50(7)	53(8)	45(7)	5(6)	1(5)	9(6)
C(46B)	54(8)	45(7)	32(6)	-6(5)	-8(5)	16(6)
B(1B)	25(5)	20(5)	22(4)	1(4)	-2(4)	-1(5)
C(47B)	16(5)	21(5)	30(5)	-1(4)	-5(4)	2(4)
C(48B)	26(6)	23(5)	26(5)	1(4)	1(4)	-1(4)
C(49B)	33(6)	20(5)	23(5)	3(4)	-3(4)	1(4)
C(50B)	35(6)	29(5)	24(5)	7(4)	-3(5)	-6(4)
C(51B)	22(6)	28(5)	26(5)	-2(4)	0(4)	2(4)
C(52B)	20(5)	25(5)	33(5)	-2(4)	-6(4)	1(4)
C(53B)	37(7)	32(6)	31(6)	9(4)	-12(5)	-6(5)
F(53D)	89(6)	46(4)	48(4)	22(3)	5(4)	28(4)
F(53E)	73(6)	36(4)	78(5)	-12(4)	-34(4)	13(4)
F(53F)	40(4)	39(4)	76(5)	-3(3)	-7(4)	14(3)
C(54B)	53(9)	41(7)	37(6)	2(5)	14(6)	2(6)
F(54D)	194(11)	95(6)	30(4)	18(4)	38(5)	71(7)
F(54E)	196(13)	76(7)	89(7)	-56(5)	85(8)	-37(7)
F(54F)	79(8)	177(11)	90(7)	-22(7)	49(6)	26(7)
C(55B)	29(6)	24(5)	19(5)	0(4)	-1(4)	4(4)
C(56B)	26(6)	22(5)	24(5)	4(4)	-2(4)	-1(4)
C(57B)	29(6)	29(6)	28(5)	5(4)	-6(5)	-1(5)
C(58B)	39(7)	28(6)	34(6)	-12(4)	-7(5)	4(5)
C(59B)	49(8)	46(7)	18(5)	0(4)	5(5)	-6(5)
C(60B)	35(6)	25(5)	22(5)	0(4)	-1(4)	-9(4)
C(61B)	59(9)	16(5)	42(7)	1(4)	-7(6)	-7(5)
F(61D)	116(8)	33(4)	84(6)	19(4)	-55(5)	-23(4)

F(61E)	61(6)	53(5)	99(6)	-16(4)	12(5)	-29(4)
F(61F)	108(7)	42(4)	55(4)	-23(3)	3(4)	-24(4)
C(62B)	68(10)	70(9)	27(6)	-2(6)	15(6)	-22(7)
F(62D)	189(11)	90(6)	48(5)	-12(4)	58(6)	-39(7)
F(62E)	245(16)	65(6)	152(11)	61(6)	152(11)	53(8)
F(62F)	90(9)	226(14)	88(7)	5(8)	33(6)	-61(9)
C(63B)	31(6)	14(4)	17(5)	4(3)	-5(4)	2(4)
C(64B)	38(7)	16(5)	29(5)	3(4)	-3(5)	2(4)
C(65B)	21(5)	25(5)	26(5)	5(4)	1(4)	5(4)
C(66B)	40(7)	26(5)	20(5)	5(4)	-5(4)	-8(5)
C(67B)	29(6)	23(5)	20(5)	-1(3)	0(4)	3(4)
C(68B)	22(5)	32(5)	23(5)	4(4)	-3(4)	0(4)
C(69B)	42(8)	26(6)	23(5)	-3(4)	1(5)	-5(5)
F(69D)	40(4)	67(5)	32(3)	-8(3)	7(3)	6(3)
F(69E)	28(4)	52(4)	65(4)	-18(3)	3(3)	-12(3)
F(69F)	37(4)	41(4)	56(4)	13(3)	8(3)	11(3)
C(70B)	29(6)	31(6)	57(7)	-22(5)	-10(5)	2(5)
F(70D)	44(5)	90(6)	125(7)	-81(5)	-2(4)	7(4)
F(70E)	109(7)	87(6)	53(5)	-33(4)	-29(5)	29(5)
F(70F)	125(8)	39(4)	98(6)	-32(4)	48(5)	-26(5)
C(71B)	32(6)	22(5)	27(5)	-5(4)	-3(5)	1(4)
C(72B)	21(5)	24(5)	19(5)	-2(4)	-4(4)	1(4)
C(73B)	31(6)	29(5)	29(5)	-5(4)	-2(4)	5(4)
C(74B)	35(6)	39(6)	31(5)	-10(4)	-12(5)	11(5)
C(75B)	26(6)	23(5)	39(6)	1(4)	-10(5)	1(4)
C(76B)	40(7)	22(5)	26(5)	1(4)	-1(4)	-6(4)
C(77B)	20(6)	41(7)	45(7)	-7(5)	5(5)	9(5)
F(77D)	47(4)	63(4)	31(3)	-3(3)	7(3)	-6(3)
F(77E)	35(4)	71(5)	67(4)	6(4)	1(3)	18(4)
F(77F)	38(4)	52(4)	59(4)	-25(3)	9(3)	-14(3)
C(78B)	60(9)	30(6)	55(8)	15(5)	-23(6)	6(6)
F(78D)	135(8)	43(5)	93(6)	27(4)	10(5)	40(5)
F(78E)	170(10)	56(5)	42(4)	24(4)	-8(5)	-13(5)
F(78F)	86(7)	62(5)	163(9)	75(6)	-54(6)	-40(5)
C(1X)	34(9)	38(9)	32(9)	9(8)	-5(8)	8(8)
C(2X)	55(10)	27(9)	28(8)	-7(7)	7(8)	4(8)
CL1X	25(2)	35(3)	22(2)	6(2)	3(2)	-5(2)
CL2X	53(4)	51(3)	42(3)	15(2)	5(3)	-8(3)
O(1Z)	28(6)	19(6)	9(4)	2(4)	10(4)	-4(5)
C(1Y)	67(9)	74(9)	31(6)	9(6)	-6(6)	-12(7)
C(2Y)	84(11)	47(7)	41(7)	10(5)	6(7)	-5(7)
CL1Y	88(3)	81(2)	45(2)	3(2)	-5(2)	5(2)
CL2Y	70(2)	57(2)	74(2)	-7(2)	18(2)	-13(2)

Table S 6: Hydrogen coordinates (x 10^4) and isotropic displacement parameters (Å^2 x 10^3) for W-13.

	x	y	z	U(eq)
H(2A1)	-98	2240	5618	46
H(2A2)	-188	2852	6201	46
H(3A1)	702	2759	5630	66
H(3A2)	597	2225	6228	66
H(4A1)	545	3885	6656	50
H(4A2)	1037	3882	6312	50
H(7A)	-1019	3158	3987	43
H(9A)	-1664	4755	5214	40
H(11A)	173	3248	4558	75
H(11B)	-140	2875	4016	75
H(11C)	-121	2143	4569	75
H(12A)	-2145	3854	4401	81
H(12B)	-1840	3603	3852	81
H(12C)	-1908	4816	4057	81
H(13A)	-1188	5387	6062	48
H(13B)	-657	4898	6170	48
H(13C)	-1126	4167	6259	48
H(16A)	1687	7234	5699	39
H(18A)	754	7821	6944	39
H(20A)	1614	5696	5127	53
H(20B)	1126	5001	5128	53
H(20C)	1561	4728	5563	53
H(21A)	1737	8902	6177	71
H(21B)	1698	8636	6834	71
H(21C)	1277	9304	6517	71
H(22A)	-133	6327	6623	55
H(22B)	167	6567	7195	55
H(22C)	170	5389	6928	55
H(25A)	1620	5993	3544	63
H(26A)	1868	7747	3724	78
H(27A)	1428	8906	4285	60
H(29A)	678	4668	4272	48
H(30A)	1413	4266	3475	98
H(30B)	1135	3383	3834	98
H(30C)	1509	4189	4141	98
H(31A)	216	5408	3519	91
H(31B)	346	4184	3389	91
H(31C)	638	5129	3093	91
H(32A)	495	8102	5177	52
H(33A)	253	9328	4180	73
H(33B)	-56	9326	4740	73

H(33C)	-28	8266	4363	73
H(34A)	1201	9278	5246	113
H(34B)	699	9870	5363	113
H(34C)	950	10009	4768	113
H(36A)	-1180	8568	5967	51
H(36B)	-1222	7299	5874	51
H(36C)	-1178	8081	5343	51
H(37A)	108	8389	6001	30
H(39A)	358	9574	6759	67
H(39B)	59	10664	6824	67
H(39C)	264	10334	6224	67
H(40A)	-314	8432	7126	67
H(40B)	-859	8652	6909	67
H(40C)	-570	9549	7263	67
H(42A)	-976	10833	6910	40
H(43A)	-1485	12117	6524	44
H(44A)	-1572	12320	5558	47
H(45A)	-1088	11274	4966	48
H(46A)	-540	10035	5365	45
H(48A)	7427	7216	1758	32
H(50A)	7806	4136	1373	40
H(52A)	8155	5396	2896	34
H(56A)	7072	5977	3054	35
H(58A)	6947	5898	4738	38
H(60A)	7962	7784	4029	34
H(64A)	8738	6731	2644	35
H(66A)	9591	9067	3300	35
H(68A)	8124	9316	3346	37
H(72A)	6828	8215	3050	41
H(74A)	6718	10741	1989	45
H(76A)	7957	9058	2099	37
H(2B1)	4222	6222	4794	44
H(2B2)	3778	6279	5214	44
H(3B1)	4167	7455	5782	61
H(3B2)	4234	7887	5154	61
H(4B1)	5033	7214	5145	56
H(4B2)	4985	7935	5701	56
H(7B)	3214	2823	5780	40
H(9B)	4133	2357	4505	42
H(11D)	3757	5154	6330	51
H(11E)	3318	4331	6410	51
H(11F)	3271	5269	5952	51
H(12D)	3333	1322	4564	77
H(12E)	3070	1333	5157	77

H(12F)	3587	768	5104	77
H(13D)	4998	3983	4838	63
H(13E)	4661	4831	4508	63
H(13F)	4723	3646	4263	63
H(16B)	6565	5841	6260	41
H(18B)	5768	7176	7466	44
H(20D)	5615	5263	5303	64
H(20E)	6185	5123	5429	64
H(20F)	5977	6243	5199	64
H(21D)	6783	5882	7424	79
H(21E)	6615	7045	7626	79
H(21F)	6944	6926	7084	79
H(22D)	4898	7285	7368	77
H(22E)	4629	6872	6804	77
H(22F)	4879	8030	6819	77
H(25B)	3352	3927	7954	60
H(26B)	3157	2167	7814	70
H(27B)	3594	1129	7185	69
H(29B)	4200	5429	7202	49
H(30D)	4335	4864	8351	89
H(30E)	4507	5983	8095	89
H(30F)	4743	4886	7885	89
H(31D)	3568	5777	8098	92
H(31E)	3358	5683	7465	92
H(31F)	3735	6614	7630	92
H(32B)	4470	2049	6263	47
H(33D)	5002	1927	7043	65
H(33E)	5049	897	6647	65
H(33F)	4759	816	7220	65
H(34D)	4336	270	6064	81
H(34E)	3816	791	6170	81
H(34F)	4075	77	6650	81
H(36D)	6107	1655	5544	83
H(36E)	6050	2145	6162	83
H(36F)	6145	2923	5640	83
H(37B)	4819	1907	5347	34
H(39D)	4715	-141	5172	70
H(39E)	4920	-436	4568	70
H(39F)	4588	604	4640	70
H(40D)	5208	1812	4239	62
H(40E)	5524	764	4118	62
H(40F)	5759	1743	4464	62
H(42B)	5347	-57	5964	48
H(43B)	5880	-1298	6366	54

H(44B)	6563	-1814	5902	54
H(45B)	6663	-1288	4973	59
H(46B)	6131	-16	4553	53
H(48B)	2782	6515	8537	30
H(50B)	2384	6133	10152	36
H(52B)	1992	8797	9288	31
H(56B)	2050	6559	7897	29
H(58B)	2521	6193	6318	40
H(60B)	2871	8865	7214	33
H(64B)	3417	8347	8015	33
H(66B)	4002	10868	8856	34
H(68B)	2554	10408	8831	31
H(72B)	1452	8207	8437	26
H(74B)	794	10762	7700	42
H(76B)	2246	10508	7710	35
H(1X1)	6995	9226	10130	41
H(1X2)	7402	8359	10309	41
H(2X1)	7758	8604	9436	44
H(2X2)	7778	9653	9820	44
H(1Y1)	5458	3786	8625	69
H(1Y2)	5763	2970	8253	69
H(2Y1)	5141	1691	8350	69
H(2Y2)	4969	2738	8010	69

Table S 7: Torsion angles [°] for W-13.

C(5A)-N(1A)-C(1A)-N(2A)	173.7(9)
C(2A)-N(1A)-C(1A)-N(2A)	-0.1(15)
C(5A)-N(1A)-C(1A)-W(1A)	-7.7(10)
C(2A)-N(1A)-C(1A)-W(1A)	178.4(8)
N(1A)-C(1A)-N(2A)-C(14A)	-174.8(9)
W(1A)-C(1A)-N(2A)-C(14A)	7.1(14)
N(1A)-C(1A)-N(2A)-C(4A)	3.4(15)
W(1A)-C(1A)-N(2A)-C(4A)	-174.8(8)
C(1A)-N(1A)-C(2A)-C(3A)	-29.2(15)
C(5A)-N(1A)-C(2A)-C(3A)	157.3(10)
N(1A)-C(2A)-C(3A)-C(4A)	53.0(13)
N(3A)-W(1A)-N(4A)-C(35A)	70(4)
C(1A)-W(1A)-N(4A)-C(35A)	173(4)
BR1A-W(1A)-N(4A)-C(35A)	-38(4)
C(1A)-N(2A)-C(4A)-C(3A)	22.7(15)
C(14A)-N(2A)-C(4A)-C(3A)	-159.0(11)
C(2A)-C(3A)-C(4A)-N(2A)	-51.2(14)

C(1A)-N(1A)-C(5A)-C(6A)	108.3(10)
C(2A)-N(1A)-C(5A)-C(6A)	-77.6(12)
C(1A)-N(1A)-C(5A)-C(10A)	-79.8(11)
C(2A)-N(1A)-C(5A)-C(10A)	94.2(11)
C(10A)-C(5A)-C(6A)-C(7A)	3.2(13)
N(1A)-C(5A)-C(6A)-C(7A)	174.8(9)
C(10A)-C(5A)-C(6A)-C(11A)	-172.8(9)
N(1A)-C(5A)-C(6A)-C(11A)	-1.2(14)
C(5A)-C(6A)-C(7A)-C(8A)	1.6(15)
C(11A)-C(6A)-C(7A)-C(8A)	177.5(10)
C(6A)-C(7A)-C(8A)-C(9A)	-3.2(15)
C(6A)-C(7A)-C(8A)-C(12A)	174.9(10)
C(7A)-C(8A)-C(9A)-C(10A)	0.1(15)
C(12A)-C(8A)-C(9A)-C(10A)	-178.1(10)
C(8A)-C(9A)-C(10A)-C(5A)	4.4(15)
C(8A)-C(9A)-C(10A)-C(13A)	-171.4(9)
C(6A)-C(5A)-C(10A)-C(9A)	-6.0(13)
N(1A)-C(5A)-C(10A)-C(9A)	-177.9(8)
C(6A)-C(5A)-C(10A)-C(13A)	169.7(9)
N(1A)-C(5A)-C(10A)-C(13A)	-2.1(13)
C(1A)-N(2A)-C(14A)-C(15A)	-95.9(12)
C(4A)-N(2A)-C(14A)-C(15A)	85.8(12)
C(1A)-N(2A)-C(14A)-C(19A)	90.5(12)
C(4A)-N(2A)-C(14A)-C(19A)	-87.8(12)
C(19A)-C(14A)-C(15A)-C(16A)	-6.3(15)
N(2A)-C(14A)-C(15A)-C(16A)	-179.6(9)
C(19A)-C(14A)-C(15A)-C(20A)	171.7(9)
N(2A)-C(14A)-C(15A)-C(20A)	-1.5(15)
C(14A)-C(15A)-C(16A)-C(17A)	4.4(15)
C(20A)-C(15A)-C(16A)-C(17A)	-173.7(9)
C(15A)-C(16A)-C(17A)-C(18A)	0.3(15)
C(15A)-C(16A)-C(17A)-C(21A)	178.5(10)
C(16A)-C(17A)-C(18A)-C(19A)	-3.2(16)
C(21A)-C(17A)-C(18A)-C(19A)	178.5(9)
C(17A)-C(18A)-C(19A)-C(14A)	1.4(15)
C(17A)-C(18A)-C(19A)-C(22A)	-178.7(9)
C(15A)-C(14A)-C(19A)-C(18A)	3.5(15)
N(2A)-C(14A)-C(19A)-C(18A)	177.0(9)
C(15A)-C(14A)-C(19A)-C(22A)	-176.4(10)
N(2A)-C(14A)-C(19A)-C(22A)	-2.9(14)
N(3A)-C(23A)-C(24A)-C(25A)	-176.3(10)
C(28A)-C(23A)-C(24A)-C(25A)	1.8(17)
N(3A)-C(23A)-C(24A)-C(29A)	2.9(16)
C(28A)-C(23A)-C(24A)-C(29A)	-179.0(10)

C(23A)-C(24A)-C(25A)-C(26A)	-1.1(17)
C(29A)-C(24A)-C(25A)-C(26A)	179.7(11)
C(24A)-C(25A)-C(26A)-C(27A)	-1(2)
C(25A)-C(26A)-C(27A)-C(28A)	2(2)
C(26A)-C(27A)-C(28A)-C(23A)	-1.6(18)
C(26A)-C(27A)-C(28A)-C(32A)	-176.9(10)
N(3A)-C(23A)-C(28A)-C(27A)	177.6(10)
C(24A)-C(23A)-C(28A)-C(27A)	-0.4(17)
N(3A)-C(23A)-C(28A)-C(32A)	-7.1(16)
C(24A)-C(23A)-C(28A)-C(32A)	174.9(10)
C(25A)-C(24A)-C(29A)-C(30A)	32.5(16)
C(23A)-C(24A)-C(29A)-C(30A)	-146.7(11)
C(25A)-C(24A)-C(29A)-C(31A)	-93.4(13)
C(23A)-C(24A)-C(29A)-C(31A)	87.4(13)
C(27A)-C(28A)-C(32A)-C(34A)	-23.6(15)
C(23A)-C(28A)-C(32A)-C(34A)	161.1(10)
C(27A)-C(28A)-C(32A)-C(33A)	99.8(13)
C(23A)-C(28A)-C(32A)-C(33A)	-75.5(12)
W(1A)-N(4A)-C(35A)-C(37A)	-105(4)
W(1A)-N(4A)-C(35A)-C(36A)	76(4)
N(4A)-C(35A)-C(37A)-C(38A)	-172.3(9)
C(36A)-C(35A)-C(37A)-C(38A)	6.1(16)
C(35A)-C(37A)-C(38A)-C(41A)	-50.9(14)
C(35A)-C(37A)-C(38A)-C(39A)	-171.4(9)
C(35A)-C(37A)-C(38A)-C(40A)	72.2(12)
C(37A)-C(38A)-C(41A)-C(46A)	-29.9(14)
C(39A)-C(38A)-C(41A)-C(46A)	90.5(11)
C(40A)-C(38A)-C(41A)-C(46A)	-151.3(10)
C(37A)-C(38A)-C(41A)-C(42A)	156.0(9)
C(39A)-C(38A)-C(41A)-C(42A)	-83.6(12)
C(40A)-C(38A)-C(41A)-C(42A)	34.6(13)
C(46A)-C(41A)-C(42A)-C(43A)	2.1(15)
C(38A)-C(41A)-C(42A)-C(43A)	176.4(10)
C(41A)-C(42A)-C(43A)-C(44A)	1.1(16)
C(42A)-C(43A)-C(44A)-C(45A)	-2.6(16)
C(43A)-C(44A)-C(45A)-C(46A)	0.7(16)
C(42A)-C(41A)-C(46A)-C(45A)	-3.9(16)
C(38A)-C(41A)-C(46A)-C(45A)	-178.3(10)
C(44A)-C(45A)-C(46A)-C(41A)	2.6(17)
C(63A)-B(1A)-C(47A)-C(48A)	-112.8(10)
C(71A)-B(1A)-C(47A)-C(48A)	6.5(13)
C(55A)-B(1A)-C(47A)-C(48A)	124.8(9)
C(63A)-B(1A)-C(47A)-C(52A)	68.2(11)
C(71A)-B(1A)-C(47A)-C(52A)	-172.5(8)

C(55A)-B(1A)-C(47A)-C(52A)	-54.1(12)
C(52A)-C(47A)-C(48A)-C(49A)	-0.7(13)
B(1A)-C(47A)-C(48A)-C(49A)	-179.7(9)
C(47A)-C(48A)-C(49A)-C(50A)	1.0(14)
C(47A)-C(48A)-C(49A)-C(53A)	-178.5(9)
C(48A)-C(49A)-C(50A)-C(51A)	-0.9(14)
C(53A)-C(49A)-C(50A)-C(51A)	178.5(9)
C(49A)-C(50A)-C(51A)-C(52A)	0.6(14)
C(49A)-C(50A)-C(51A)-C(54A)	-178.1(9)
C(50A)-C(51A)-C(52A)-C(47A)	-0.3(15)
C(54A)-C(51A)-C(52A)-C(47A)	178.3(9)
C(48A)-C(47A)-C(52A)-C(51A)	0.4(14)
B(1A)-C(47A)-C(52A)-C(51A)	179.4(9)
C(50A)-C(49A)-C(53A)-F(53C)	127.2(10)
C(48A)-C(49A)-C(53A)-F(53C)	-53.3(13)
C(50A)-C(49A)-C(53A)-F(53A)	-109.9(11)
C(48A)-C(49A)-C(53A)-F(53A)	69.6(11)
C(50A)-C(49A)-C(53A)-F(53B)	7.4(14)
C(48A)-C(49A)-C(53A)-F(53B)	-173.2(9)
C(50A)-C(51A)-C(54A)-F(54C)	152.6(11)
C(52A)-C(51A)-C(54A)-F(54C)	-26.1(16)
C(50A)-C(51A)-C(54A)-F(54B)	23.5(15)
C(52A)-C(51A)-C(54A)-F(54B)	-155.2(10)
C(50A)-C(51A)-C(54A)-F(54A)	-91.2(12)
C(52A)-C(51A)-C(54A)-F(54A)	90.1(12)
C(47A)-B(1A)-C(55A)-C(56A)	-35.1(12)
C(63A)-B(1A)-C(55A)-C(56A)	-156.5(9)
C(71A)-B(1A)-C(55A)-C(56A)	83.5(10)
C(47A)-B(1A)-C(55A)-C(60A)	145.6(9)
C(63A)-B(1A)-C(55A)-C(60A)	24.3(12)
C(71A)-B(1A)-C(55A)-C(60A)	-95.7(10)
C(60A)-C(55A)-C(56A)-C(57A)	0.9(14)
B(1A)-C(55A)-C(56A)-C(57A)	-178.3(9)
C(55A)-C(56A)-C(57A)-C(58A)	1.1(16)
C(55A)-C(56A)-C(57A)-C(61A)	-176.7(9)
C(56A)-C(57A)-C(58A)-C(59A)	-1.1(15)
C(61A)-C(57A)-C(58A)-C(59A)	176.7(9)
C(57A)-C(58A)-C(59A)-C(60A)	-0.9(14)
C(57A)-C(58A)-C(59A)-C(62A)	-177.8(9)
C(56A)-C(55A)-C(60A)-C(59A)	-2.9(13)
B(1A)-C(55A)-C(60A)-C(59A)	176.4(8)
C(58A)-C(59A)-C(60A)-C(55A)	2.9(14)
C(62A)-C(59A)-C(60A)-C(55A)	179.9(9)
C(56A)-C(57A)-C(61A)-F(61A)	-41.1(14)

C(58A)-C(57A)-C(61A)-F(61A)	141.1(10)
C(56A)-C(57A)-C(61A)-F(61B)	-164.2(10)
C(58A)-C(57A)-C(61A)-F(61B)	18.0(15)
C(56A)-C(57A)-C(61A)-F(61C)	75.7(12)
C(58A)-C(57A)-C(61A)-F(61C)	-102.1(11)
C(58A)-C(59A)-C(62A)-F(62C)	-29.9(13)
C(60A)-C(59A)-C(62A)-F(62C)	153.2(9)
C(58A)-C(59A)-C(62A)-F(62B)	-150.9(9)
C(60A)-C(59A)-C(62A)-F(62B)	32.1(13)
C(58A)-C(59A)-C(62A)-F(62A)	90.8(11)
C(60A)-C(59A)-C(62A)-F(62A)	-86.2(11)
C(47A)-B(1A)-C(63A)-C(64A)	-15.1(12)
C(71A)-B(1A)-C(63A)-C(64A)	-135.4(8)
C(55A)-B(1A)-C(63A)-C(64A)	104.0(10)
C(47A)-B(1A)-C(63A)-C(68A)	164.1(8)
C(71A)-B(1A)-C(63A)-C(68A)	43.8(10)
C(55A)-B(1A)-C(63A)-C(68A)	-76.8(10)
C(68A)-C(63A)-C(64A)-C(65A)	1.8(12)
B(1A)-C(63A)-C(64A)-C(65A)	-179.0(8)
C(63A)-C(64A)-C(65A)-C(66A)	-0.8(13)
C(63A)-C(64A)-C(65A)-C(69A)	-177.5(8)
C(64A)-C(65A)-C(66A)-C(67A)	0.5(13)
C(69A)-C(65A)-C(66A)-C(67A)	177.2(9)
C(65A)-C(66A)-C(67A)-C(68A)	-1.4(13)
C(65A)-C(66A)-C(67A)-C(70A)	-177.1(9)
C(64A)-C(63A)-C(68A)-C(67A)	-2.7(12)
B(1A)-C(63A)-C(68A)-C(67A)	178.0(8)
C(66A)-C(67A)-C(68A)-C(63A)	2.6(14)
C(70A)-C(67A)-C(68A)-C(63A)	178.3(9)
C(66A)-C(65A)-C(69A)-F(69A)	-83.6(12)
C(64A)-C(65A)-C(69A)-F(69A)	93.1(12)
C(66A)-C(65A)-C(69A)-F(69C)	153.8(9)
C(64A)-C(65A)-C(69A)-F(69C)	-29.5(13)
C(66A)-C(65A)-C(69A)-F(69B)	34.8(13)
C(64A)-C(65A)-C(69A)-F(69B)	-148.5(9)
C(66A)-C(67A)-C(70A)-F(70A)	-152.3(11)
C(68A)-C(67A)-C(70A)-F(70A)	32.0(16)
C(66A)-C(67A)-C(70A)-F(70C)	88.6(15)
C(68A)-C(67A)-C(70A)-F(70C)	-87.0(14)
C(66A)-C(67A)-C(70A)-F(70B)	-28.5(15)
C(68A)-C(67A)-C(70A)-F(70B)	155.8(10)
C(47A)-B(1A)-C(71A)-C(76A)	-78.1(10)
C(63A)-B(1A)-C(71A)-C(76A)	42.8(10)
C(55A)-B(1A)-C(71A)-C(76A)	165.3(8)

C(47A)-B(1A)-C(71A)-C(72A)	100.6(10)
C(63A)-B(1A)-C(71A)-C(72A)	-138.5(9)
C(55A)-B(1A)-C(71A)-C(72A)	-16.1(12)
C(76A)-C(71A)-C(72A)-C(73A)	-0.1(14)
B(1A)-C(71A)-C(72A)-C(73A)	-178.8(9)
C(71A)-C(72A)-C(73A)-C(74A)	0.5(15)
C(71A)-C(72A)-C(73A)-C(77A)	-179.1(10)
C(72A)-C(73A)-C(74A)-C(75A)	-0.4(15)
C(77A)-C(73A)-C(74A)-C(75A)	179.2(11)
C(73A)-C(74A)-C(75A)-C(76A)	-0.1(15)
C(73A)-C(74A)-C(75A)-C(78A)	179.0(9)
C(74A)-C(75A)-C(76A)-C(71A)	0.7(15)
C(78A)-C(75A)-C(76A)-C(71A)	-178.5(9)
C(72A)-C(71A)-C(76A)-C(75A)	-0.6(13)
B(1A)-C(71A)-C(76A)-C(75A)	178.3(8)
C(72A)-C(73A)-C(77A)-F(77A)	177.0(12)
C(74A)-C(73A)-C(77A)-F(77A)	-3(2)
C(72A)-C(73A)-C(77A)-F(77C)	46.6(18)
C(74A)-C(73A)-C(77A)-F(77C)	-133.0(12)
C(72A)-C(73A)-C(77A)-F(77B)	-64.5(16)
C(74A)-C(73A)-C(77A)-F(77B)	115.9(13)
C(74A)-C(75A)-C(78A)-F(78B)	26.2(14)
C(76A)-C(75A)-C(78A)-F(78B)	-154.6(9)
C(74A)-C(75A)-C(78A)-F(78A)	150.7(10)
C(76A)-C(75A)-C(78A)-F(78A)	-30.1(14)
C(74A)-C(75A)-C(78A)-F(78C)	-92.8(11)
C(76A)-C(75A)-C(78A)-F(78C)	86.4(12)
C(5B)-N(1B)-C(1B)-N(2B)	169.0(9)
C(2B)-N(1B)-C(1B)-N(2B)	-2.4(14)
C(5B)-N(1B)-C(1B)-W(1B)	-15.9(14)
C(2B)-N(1B)-C(1B)-W(1B)	172.7(7)
N(1B)-C(1B)-N(2B)-C(14B)	179.1(8)
W(1B)-C(1B)-N(2B)-C(14B)	3.1(10)
N(1B)-C(1B)-N(2B)-C(4B)	-7.0(15)
W(1B)-C(1B)-N(2B)-C(4B)	177.0(8)
C(1B)-N(1B)-C(2B)-C(3B)	-19.2(14)
C(5B)-N(1B)-C(2B)-C(3B)	169.0(10)
N(4B)-W(1B)-N(3B)-C(23B)	21(4)
C(1B)-W(1B)-N(3B)-C(23B)	128(4)
BR1B-W(1B)-N(3B)-C(23B)	-86(4)
N(1B)-C(2B)-C(3B)-C(4B)	47.5(13)
N(3B)-W(1B)-N(4B)-C(35B)	-63(4)
C(1B)-W(1B)-N(4B)-C(35B)	-169(3)
BR1B-W(1B)-N(4B)-C(35B)	44(4)

C(1B)-N(2B)-C(4B)-C(3B)	34.6(14)
C(14B)-N(2B)-C(4B)-C(3B)	-151.7(10)
C(2B)-C(3B)-C(4B)-N(2B)	-53.7(13)
C(1B)-N(1B)-C(5B)-C(6B)	100.0(12)
C(2B)-N(1B)-C(5B)-C(6B)	-88.1(11)
C(1B)-N(1B)-C(5B)-C(10B)	-85.6(12)
C(2B)-N(1B)-C(5B)-C(10B)	86.4(12)
C(10B)-C(5B)-C(6B)-C(7B)	3.5(15)
N(1B)-C(5B)-C(6B)-C(7B)	177.8(9)
C(10B)-C(5B)-C(6B)-C(11B)	-172.3(9)
N(1B)-C(5B)-C(6B)-C(11B)	2.0(15)
C(5B)-C(6B)-C(7B)-C(8B)	-2.9(15)
C(11B)-C(6B)-C(7B)-C(8B)	173.0(9)
C(6B)-C(7B)-C(8B)-C(9B)	0.5(15)
C(6B)-C(7B)-C(8B)-C(12B)	-179.4(10)
C(7B)-C(8B)-C(9B)-C(10B)	1.3(16)
C(12B)-C(8B)-C(9B)-C(10B)	-178.7(10)
C(8B)-C(9B)-C(10B)-C(5B)	-0.7(15)
C(8B)-C(9B)-C(10B)-C(13B)	179.0(10)
C(6B)-C(5B)-C(10B)-C(9B)	-1.7(15)
N(1B)-C(5B)-C(10B)-C(9B)	-176.0(9)
C(6B)-C(5B)-C(10B)-C(13B)	178.5(10)
N(1B)-C(5B)-C(10B)-C(13B)	4.3(15)
C(1B)-N(2B)-C(14B)-C(15B)	87.1(12)
C(4B)-N(2B)-C(14B)-C(15B)	-87.0(13)
C(1B)-N(2B)-C(14B)-C(19B)	-101.8(11)
C(4B)-N(2B)-C(14B)-C(19B)	84.1(12)
C(19B)-C(14B)-C(15B)-C(16B)	4.3(15)
N(2B)-C(14B)-C(15B)-C(16B)	174.9(9)
C(19B)-C(14B)-C(15B)-C(20B)	-171.2(9)
N(2B)-C(14B)-C(15B)-C(20B)	-0.6(15)
C(14B)-C(15B)-C(16B)-C(17B)	-1.5(15)
C(20B)-C(15B)-C(16B)-C(17B)	174.2(9)
C(15B)-C(16B)-C(17B)-C(18B)	-1.2(14)
C(15B)-C(16B)-C(17B)-C(21B)	-179.6(10)
C(16B)-C(17B)-C(18B)-C(19B)	1.1(15)
C(21B)-C(17B)-C(18B)-C(19B)	179.5(9)
C(17B)-C(18B)-C(19B)-C(14B)	1.5(14)
C(17B)-C(18B)-C(19B)-C(22B)	-174.8(10)
C(15B)-C(14B)-C(19B)-C(18B)	-4.3(14)
N(2B)-C(14B)-C(19B)-C(18B)	-175.3(8)
C(15B)-C(14B)-C(19B)-C(22B)	172.0(10)
N(2B)-C(14B)-C(19B)-C(22B)	1.0(14)
W(1B)-N(3B)-C(23B)-C(28B)	-15(4)

W(1B)-N(3B)-C(23B)-C(24B)	165(3)
C(28B)-C(23B)-C(24B)-C(25B)	-0.6(17)
N(3B)-C(23B)-C(24B)-C(25B)	179.7(10)
C(28B)-C(23B)-C(24B)-C(29B)	177.6(10)
N(3B)-C(23B)-C(24B)-C(29B)	-2.2(16)
C(23B)-C(24B)-C(25B)-C(26B)	1.2(19)
C(29B)-C(24B)-C(25B)-C(26B)	-177.0(12)
C(24B)-C(25B)-C(26B)-C(27B)	-1(2)
C(25B)-C(26B)-C(27B)-C(28B)	0(2)
C(26B)-C(27B)-C(28B)-C(23B)	0.3(18)
C(26B)-C(27B)-C(28B)-C(32B)	174.3(11)
N(3B)-C(23B)-C(28B)-C(27B)	179.6(10)
C(24B)-C(23B)-C(28B)-C(27B)	-0.1(17)
N(3B)-C(23B)-C(28B)-C(32B)	5.6(15)
C(24B)-C(23B)-C(28B)-C(32B)	-174.1(10)
C(25B)-C(24B)-C(29B)-C(31B)	-32.3(16)
C(23B)-C(24B)-C(29B)-C(31B)	149.6(11)
C(25B)-C(24B)-C(29B)-C(30B)	92.3(14)
C(23B)-C(24B)-C(29B)-C(30B)	-85.8(13)
C(27B)-C(28B)-C(32B)-C(33B)	-98.2(13)
C(23B)-C(28B)-C(32B)-C(33B)	75.5(13)
C(27B)-C(28B)-C(32B)-C(34B)	26.6(16)
C(23B)-C(28B)-C(32B)-C(34B)	-159.6(10)
W(1B)-N(4B)-C(35B)-C(37B)	110(3)
W(1B)-N(4B)-C(35B)-C(36B)	-72(4)
N(4B)-C(35B)-C(37B)-C(38B)	172.3(9)
C(36B)-C(35B)-C(37B)-C(38B)	-5.4(18)
C(35B)-C(37B)-C(38B)-C(41B)	44.1(15)
C(35B)-C(37B)-C(38B)-C(39B)	162.2(10)
C(35B)-C(37B)-C(38B)-C(40B)	-80.7(13)
C(37B)-C(38B)-C(41B)-C(42B)	46.7(14)
C(39B)-C(38B)-C(41B)-C(42B)	-73.0(12)
C(40B)-C(38B)-C(41B)-C(42B)	168.2(9)
C(37B)-C(38B)-C(41B)-C(46B)	-132.5(11)
C(39B)-C(38B)-C(41B)-C(46B)	107.7(12)
C(40B)-C(38B)-C(41B)-C(46B)	-11.1(14)
C(46B)-C(41B)-C(42B)-C(43B)	-3.0(16)
C(38B)-C(41B)-C(42B)-C(43B)	177.7(10)
C(41B)-C(42B)-C(43B)-C(44B)	3.5(18)
C(42B)-C(43B)-C(44B)-C(45B)	-4.1(18)
C(43B)-C(44B)-C(45B)-C(46B)	4.4(18)
C(42B)-C(41B)-C(46B)-C(45B)	3.2(16)
C(38B)-C(41B)-C(46B)-C(45B)	-177.5(10)
C(44B)-C(45B)-C(46B)-C(41B)	-4.0(18)

C(71B)-B(1B)-C(47B)-C(48B)	149.2(9)
C(63B)-B(1B)-C(47B)-C(48B)	-89.2(10)
C(55B)-B(1B)-C(47B)-C(48B)	32.0(15)
C(71B)-B(1B)-C(47B)-C(52B)	-37.8(13)
C(63B)-B(1B)-C(47B)-C(52B)	83.8(11)
C(55B)-B(1B)-C(47B)-C(52B)	-155.0(8)
C(52B)-C(47B)-C(48B)-C(49B)	0.7(14)
B(1B)-C(47B)-C(48B)-C(49B)	174.3(10)
C(47B)-C(48B)-C(49B)-C(50B)	0.4(16)
C(47B)-C(48B)-C(49B)-C(53B)	176.0(9)
C(48B)-C(49B)-C(50B)-C(51B)	-1.7(15)
C(53B)-C(49B)-C(50B)-C(51B)	-177.1(9)
C(49B)-C(50B)-C(51B)-C(52B)	1.9(15)
C(49B)-C(50B)-C(51B)-C(54B)	-178.7(10)
C(50B)-C(51B)-C(52B)-C(47B)	-0.8(15)
C(54B)-C(51B)-C(52B)-C(47B)	179.8(10)
C(48B)-C(47B)-C(52B)-C(51B)	-0.5(14)
B(1B)-C(47B)-C(52B)-C(51B)	-174.0(9)
C(50B)-C(49B)-C(53B)-F(53E)	117.8(11)
C(48B)-C(49B)-C(53B)-F(53E)	-57.9(13)
C(50B)-C(49B)-C(53B)-F(53F)	-121.5(11)
C(48B)-C(49B)-C(53B)-F(53F)	62.9(12)
C(50B)-C(49B)-C(53B)-F(53D)	-4.5(15)
C(48B)-C(49B)-C(53B)-F(53D)	179.8(9)
C(52B)-C(51B)-C(54B)-F(54D)	-178.6(11)
C(50B)-C(51B)-C(54B)-F(54D)	2.0(17)
C(52B)-C(51B)-C(54B)-F(54E)	-48.4(16)
C(50B)-C(51B)-C(54B)-F(54E)	132.2(12)
C(52B)-C(51B)-C(54B)-F(54F)	66.6(14)
C(50B)-C(51B)-C(54B)-F(54F)	-112.8(12)
C(71B)-B(1B)-C(55B)-C(60B)	85.1(11)
C(47B)-B(1B)-C(55B)-C(60B)	-151.9(9)
C(63B)-B(1B)-C(55B)-C(60B)	-35.6(13)
C(71B)-B(1B)-C(55B)-C(56B)	-87.5(10)
C(47B)-B(1B)-C(55B)-C(56B)	35.6(14)
C(63B)-B(1B)-C(55B)-C(56B)	151.8(9)
C(60B)-C(55B)-C(56B)-C(57B)	5.6(15)
B(1B)-C(55B)-C(56B)-C(57B)	178.5(10)
C(55B)-C(56B)-C(57B)-C(58B)	-5.1(16)
C(55B)-C(56B)-C(57B)-C(61B)	175.5(10)
C(56B)-C(57B)-C(58B)-C(59B)	1.8(16)
C(61B)-C(57B)-C(58B)-C(59B)	-178.7(11)
C(57B)-C(58B)-C(59B)-C(60B)	0.6(17)
C(57B)-C(58B)-C(59B)-C(62B)	-179.4(11)

C(56B)-C(55B)-C(60B)-C(59B)	-3.0(15)
B(1B)-C(55B)-C(60B)-C(59B)	-175.8(10)
C(58B)-C(59B)-C(60B)-C(55B)	0.1(18)
C(62B)-C(59B)-C(60B)-C(55B)	180.0(11)
C(56B)-C(57B)-C(61B)-F(61F)	175.6(10)
C(58B)-C(57B)-C(61B)-F(61F)	-3.9(16)
C(56B)-C(57B)-C(61B)-F(61D)	-60.9(15)
C(58B)-C(57B)-C(61B)-F(61D)	119.6(11)
C(56B)-C(57B)-C(61B)-F(61E)	54.0(14)
C(58B)-C(57B)-C(61B)-F(61E)	-125.4(11)
C(58B)-C(59B)-C(62B)-F(62E)	128.4(15)
C(60B)-C(59B)-C(62B)-F(62E)	-51.5(19)
C(58B)-C(59B)-C(62B)-F(62D)	-1(2)
C(60B)-C(59B)-C(62B)-F(62D)	178.9(12)
C(58B)-C(59B)-C(62B)-F(62F)	-114.2(14)
C(60B)-C(59B)-C(62B)-F(62F)	65.9(16)
C(71B)-B(1B)-C(63B)-C(64B)	-147.1(8)
C(47B)-B(1B)-C(63B)-C(64B)	90.1(11)
C(55B)-B(1B)-C(63B)-C(64B)	-31.4(12)
C(71B)-B(1B)-C(63B)-C(68B)	41.7(13)
C(47B)-B(1B)-C(63B)-C(68B)	-81.1(10)
C(55B)-B(1B)-C(63B)-C(68B)	157.5(8)
C(68B)-C(63B)-C(64B)-C(65B)	-1.7(13)
B(1B)-C(63B)-C(64B)-C(65B)	-173.3(9)
C(63B)-C(64B)-C(65B)-C(66B)	1.8(14)
C(63B)-C(64B)-C(65B)-C(69B)	-179.0(9)
C(64B)-C(65B)-C(66B)-C(67B)	-0.7(13)
C(69B)-C(65B)-C(66B)-C(67B)	-179.9(8)
C(65B)-C(66B)-C(67B)-C(68B)	-0.4(13)
C(65B)-C(66B)-C(67B)-C(70B)	176.9(9)
C(66B)-C(67B)-C(68B)-C(63B)	0.3(14)
C(70B)-C(67B)-C(68B)-C(63B)	-176.8(9)
C(64B)-C(63B)-C(68B)-C(67B)	0.7(13)
B(1B)-C(63B)-C(68B)-C(67B)	172.6(9)
C(66B)-C(65B)-C(69B)-F(69E)	-8.6(13)
C(64B)-C(65B)-C(69B)-F(69E)	172.2(8)
C(66B)-C(65B)-C(69B)-F(69F)	113.2(10)
C(64B)-C(65B)-C(69B)-F(69F)	-66.0(12)
C(66B)-C(65B)-C(69B)-F(69D)	-129.4(9)
C(64B)-C(65B)-C(69B)-F(69D)	51.4(11)
C(68B)-C(67B)-C(70B)-F(70D)	-6.9(16)
C(66B)-C(67B)-C(70B)-F(70D)	175.9(10)
C(68B)-C(67B)-C(70B)-F(70F)	-133.9(11)
C(66B)-C(67B)-C(70B)-F(70F)	48.8(14)

C(68B)-C(67B)-C(70B)-F(70E)	111.1(11)
C(66B)-C(67B)-C(70B)-F(70E)	-66.1(13)
C(47B)-B(1B)-C(71B)-C(72B)	-33.3(13)
C(63B)-B(1B)-C(71B)-C(72B)	-150.2(8)
C(55B)-B(1B)-C(71B)-C(72B)	88.9(10)
C(47B)-B(1B)-C(71B)-C(76B)	154.4(9)
C(63B)-B(1B)-C(71B)-C(76B)	37.4(14)
C(55B)-B(1B)-C(71B)-C(76B)	-83.4(11)
C(76B)-C(71B)-C(72B)-C(73B)	-1.5(14)
B(1B)-C(71B)-C(72B)-C(73B)	-174.5(9)
C(71B)-C(72B)-C(73B)-C(74B)	1.1(14)
C(71B)-C(72B)-C(73B)-C(77B)	-178.3(9)
C(72B)-C(73B)-C(74B)-C(75B)	0.5(14)
C(77B)-C(73B)-C(74B)-C(75B)	179.8(10)
C(73B)-C(74B)-C(75B)-C(76B)	-1.6(15)
C(73B)-C(74B)-C(75B)-C(78B)	175.6(9)
C(74B)-C(75B)-C(76B)-C(71B)	1.2(15)
C(78B)-C(75B)-C(76B)-C(71B)	-176.1(10)
C(72B)-C(71B)-C(76B)-C(75B)	0.4(14)
B(1B)-C(71B)-C(76B)-C(75B)	173.2(9)
C(74B)-C(73B)-C(77B)-F(77E)	-3.6(15)
C(72B)-C(73B)-C(77B)-F(77E)	175.7(9)
C(74B)-C(73B)-C(77B)-F(77D)	-125.4(10)
C(72B)-C(73B)-C(77B)-F(77D)	53.9(12)
C(74B)-C(73B)-C(77B)-F(77F)	115.4(10)
C(72B)-C(73B)-C(77B)-F(77F)	-65.3(13)
C(74B)-C(75B)-C(78B)-F(78E)	-82.5(15)
C(76B)-C(75B)-C(78B)-F(78E)	94.8(13)
C(74B)-C(75B)-C(78B)-F(78F)	155.0(11)
C(76B)-C(75B)-C(78B)-F(78F)	-27.8(16)
C(74B)-C(75B)-C(78B)-F(78D)	36.8(15)
C(76B)-C(75B)-C(78B)-F(78D)	-145.9(11)
CL1X-C(1X)-C(2X)-CL2X	67.2(17)
CL1Y-C(1Y)-C(2Y)-CL2Y	66.9(12)

Table S 8: Crystal data and structure refinement for W-14.

Empirical formula	$C_{83}H_{82}BBrCl_2F_{24}N_4W$
Formula weight	1936.99
Temperature	135(2) K
Wavelength	0.71073 Å
Crystal system, space group	Triclinic, $P\overline{1}$
Unit cell dimensions	a = 12.7749(7) Å, α = 85.646(2) °

	b = 17.2868(9) Å, β = 87.387(2) °
	c = 18.8293(10) Å, γ = 85.634(3) °
Volume	4130.9(4) Å^3
Z, Calculated density	2, 1.557 mg/m^3
Absorption coefficient	2.047 mm^{-1}
F(000)	1944
Crystal size	0.457 x 0.395 x 0.158 mm
Θ range for data collection	1.547 to 30.591 °
Limiting indices	-18<=h<=18, -19<=k<=24, -25<=l<=26
Reflections collected / unique	114654 / 25268 [R(int) = 0.0238]
Completeness to Θ = 25.242	100.0 %
Absorption correction	Numerical
Max. and min. transmission	0.8100 and 0.5596
Refinement method	Full-matrix least-squares on F^2
Data / restraints / parameters	25268 / 78 / 1117
Goodness-of-fit on F^2	1.029
Final R indices [I>2σ(I)]	R1 = 0.0284, wR2 = 0.0725
R indices (all data)	R1 = 0.0357, wR2 = 0.0751
Extinction coefficient	n/a
Largest diff. peak and hole	1.754 and -0.933 e.Å^{-3}

Table S 9: Atomic coordinates (x 10^4) and equivalent isotropic displacement parameters (Å^2 x 10^3) for W-14. U(eq) is defined as one third of the trace of the orthogonalized U_{ij} tensor.

	x	y	z	U(eq)
W(1)	5526(1)	7832(1)	6598(1)	16(1)
BR1	5441(1)	8651(1)	5438(1)	23(1)
N(1)	2965(1)	8261(1)	6599(1)	22(1)
C(1)	3737(2)	7739(1)	6414(1)	20(1)
N(2)	3479(1)	7097(1)	6128(1)	27(1)
N(3)	6694(1)	7270(1)	6439(1)	18(1)
N(4)	5054(1)	7203(1)	7582(1)	21(1)
C(2)	1835(2)	8158(1)	6539(1)	31(1)
C(3)	1668(2)	7283(2)	6539(2)	34(1)
C(3A)	1664(9)	7678(7)	5980(7)	40(3)
C(4)	2392(2)	6926(2)	6005(2)	39(1)
C(5)	3211(2)	9015(1)	6799(1)	22(1)
C(6)	3306(2)	9141(1)	7516(1)	25(1)
C(7)	3583(2)	9870(1)	7683(1)	30(1)
C(8)	3764(2)	10458(1)	7160(1)	33(1)
C(9)	3610(2)	10326(1)	6458(1)	32(1)
C(10)	3298(2)	9620(1)	6264(1)	26(1)
C(11)	3117(2)	8528(1)	8106(1)	32(1)
C(12)	4092(2)	11234(2)	7355(2)	47(1)

C(13)	3013(2)	9544(1)	5509(1)	32(1)
C(14)	4307(2)	6566(1)	5855(1)	25(1)
C(15)	4696(2)	6702(1)	5153(1)	28(1)
C(16)	5520(2)	6203(1)	4914(1)	32(1)
C(17)	5929(2)	5572(1)	5336(1)	34(1)
C(18)	5459(2)	5415(1)	6001(1)	33(1)
C(19)	4637(2)	5895(1)	6272(1)	29(1)
C(20)	4213(2)	7320(1)	4641(1)	37(1)
C(21)	6848(2)	5060(2)	5066(2)	46(1)
C(22)	4096(2)	5654(1)	6973(1)	38(1)
C(23)	7593(2)	6797(1)	6259(1)	21(1)
C(24)	8277(2)	7054(1)	5693(1)	28(1)
C(25)	9165(2)	6573(2)	5525(1)	39(1)
C(26)	9396(2)	5875(2)	5905(2)	42(1)
C(27)	8737(2)	5640(1)	6472(1)	34(1)
C(28)	7834(2)	6087(1)	6659(1)	24(1)
C(29)	8067(2)	7818(1)	5257(1)	32(1)
C(30)	7679(2)	7668(2)	4528(1)	43(1)
C(31)	9031(2)	8292(2)	5184(2)	45(1)
C(32)	7170(2)	5853(1)	7315(1)	23(1)
C(33)	7268(2)	4980(1)	7525(1)	35(1)
C(34)	7477(2)	6298(1)	7933(1)	31(1)
C(35)	5991(2)	8621(1)	7116(1)	19(1)
C(36)	6916(2)	8923(1)	7457(1)	22(1)
C(37)	7966(2)	8547(1)	7174(1)	29(1)
C(38)	6780(2)	8717(1)	8261(1)	34(1)
C(39)	6900(2)	9803(1)	7275(1)	22(1)
C(40)	6725(2)	10095(1)	6579(1)	36(1)
C(41)	6724(3)	10887(1)	6385(1)	41(1)
C(42)	6914(2)	11399(1)	6884(1)	31(1)
C(43)	7091(2)	11117(1)	7573(1)	28(1)
C(44)	7079(2)	10326(1)	7769(1)	26(1)
C(45)	4871(2)	6923(1)	8138(1)	24(1)
C(46)	4646(2)	6575(1)	8873(1)	32(1)
C(47)	4859(2)	5689(1)	8885(1)	40(1)
C(48)	3493(2)	6782(2)	9090(2)	56(1)
C(49)	5379(3)	6919(2)	9374(1)	47(1)
B(1)	1043(2)	7311(1)	1102(1)	18(1)
C(50)	2276(1)	7508(1)	1072(1)	17(1)
C(51)	2634(1)	8138(1)	634(1)	18(1)
C(52)	3675(2)	8316(1)	604(1)	20(1)
C(53)	4419(2)	7869(1)	1003(1)	23(1)
C(54)	4098(2)	7226(1)	1412(1)	21(1)
C(55)	3050(2)	7048(1)	1443(1)	19(1)

C(56)	4015(2)	9019(1)	168(1)	27(1)
F(56A)	4838(1)	8852(1)	-275(1)	49(1)
F(56B)	4331(2)	9548(1)	561(1)	54(1)
F(56C)	3276(1)	9345(1)	-254(1)	68(1)
C(57)	4873(2)	6715(1)	1838(2)	35(1)
F(57A)	4522(2)	6544(2)	2501(1)	91(1)
F(57B)	5770(1)	7014(1)	1897(1)	66(1)
F(57C)	5061(2)	6030(1)	1594(2)	97(1)
C(58)	228(1)	8091(1)	1003(1)	19(1)
C(59)	-796(2)	8013(1)	799(1)	24(1)
C(60)	-1541(2)	8637(1)	732(1)	26(1)
C(61)	-1302(2)	9380(1)	852(1)	28(1)
C(62)	-300(2)	9476(1)	1059(1)	24(1)
C(63)	443(2)	8844(1)	1145(1)	21(1)
C(64)	-2616(2)	8513(2)	502(1)	37(1)
F(64A)	-3369(1)	8935(1)	841(1)	66(1)
F(64B)	-2801(4)	8918(7)	-149(3)	66(2)
F(64C)	-2834(6)	7830(5)	453(9)	87(3)
F(64E)	-2647(7)	8387(14)	-150(3)	99(5)
F(64F)	-2920(6)	7793(5)	813(6)	55(2)
C(65)	-23(2)	10275(1)	1200(1)	31(1)
F(65A)	973(1)	10394(1)	1022(1)	64(1)
F(65B)	-571(2)	10836(1)	828(1)	58(1)
F(65C)	-174(2)	10425(1)	1874(1)	86(1)
C(66)	669(1)	6875(1)	1866(1)	18(1)
C(67)	-204(2)	6425(1)	1922(1)	21(1)
C(68)	-606(2)	6127(1)	2576(1)	22(1)
C(69)	-122(2)	6225(1)	3204(1)	22(1)
C(70)	757(2)	6647(1)	3159(1)	19(1)
C(71)	1126(2)	6981(1)	2508(1)	19(1)
C(72)	-1557(2)	5670(2)	2619(1)	33(1)
F(72A)	-1934(7)	5542(5)	2028(3)	71(3)
F(72B)	-2357(4)	6026(5)	2992(6)	81(3)
F(72C)	-1427(5)	4992(5)	2988(6)	92(4)
F(72D)	-2029(8)	5633(7)	3227(3)	81(3)
F(72E)	-2217(6)	5902(6)	2122(6)	85(3)
F(72F)	-1299(4)	4931(3)	2495(7)	72(3)
C(73)	1348(2)	6732(1)	3810(1)	26(1)
F(73A)	2391(1)	6613(1)	3687(1)	37(1)
F(73B)	1101(1)	6227(1)	4357(1)	35(1)
F(73C)	1198(2)	7439(1)	4056(1)	47(1)
C(74)	974(2)	6760(1)	439(1)	20(1)
C(75)	1316(2)	5968(1)	493(1)	22(1)
C(76)	1356(2)	5510(1)	-84(1)	25(1)

C(77)	1027(2)	5826(1)	-746(1)	30(1)
C(78)	684(2)	6607(1)	-811(1)	29(1)
C(79)	674(2)	7064(1)	-237(1)	25(1)
F(80A)	1507(2)	4323(1)	619(1)	60(1)
C(80)	1794(2)	4687(1)	0(1)	35(1)
F(80B)	1477(2)	4244(1)	-480(1)	75(1)
F(80C)	2828(2)	4629(1)	-10(2)	93(1)
C(81)	316(2)	6992(2)	-1507(1)	42(1)
F(81A)	233(2)	6490(1)	-1998(1)	49(1)
F(81B)	912(2)	7545(1)	-1760(1)	72(1)
F(81C)	-653(2)	7360(1)	-1444(1)	64(1)
C(1X)	9038(2)	9229(2)	3203(2)	48(1)
C(2X)	10158(3)	9279(2)	3369(2)	59(1)
CL1X	8909(1)	8382(1)	2724(1)	60(1)
CL2X	10227(1)	10068(1)	3915(1)	77(1)

Table S 10: Bond lengths [Å] and angles [°] for W-14.

W(1)-N(3)	1.7442(16)
W(1)-C(35)	1.8812(18)
W(1)-N(4)	2.1600(17)
W(1)-C(1)	2.3465(19)
W(1)-BR1	2.5140(2)
N(1)-C(1)	1.337(3)
N(1)-C(5)	1.445(2)
N(1)-C(2)	1.478(3)
C(1)-N(2)	1.340(2)
N(2)-C(14)	1.449(3)
N(2)-C(4)	1.474(3)
N(3)-C(23)	1.402(2)
N(4)-C(45)	1.142(3)
C(2)-C(3A)	1.424(11)
C(2)-C(3)	1.544(4)
C(2)-H(2A)	0.9645
C(2)-H(2B)	0.9657
C(2)-H(2C)	0.9681
C(2)-H(2D)	0.9603
C(3)-C(4)	1.473(4)
C(3)-H(3A)	0.9900
C(3)-H(3B)	0.9900
C(3A)-C(4)	1.539(12)
C(3A)-H(3C)	0.9900
C(3A)-H(3D)	0.9900

C(4)-H(4A)	0.9660
C(4)-H(4B)	0.9640
C(4)-H(4C)	0.9606
C(4)-H(4D)	0.9680
C(5)-C(6)	1.395(3)
C(5)-C(10)	1.403(3)
C(6)-C(7)	1.397(3)
C(6)-C(11)	1.501(3)
C(7)-C(8)	1.385(3)
C(7)-H(7)	0.9500
C(8)-C(9)	1.384(3)
C(8)-C(12)	1.510(3)
C(9)-C(10)	1.391(3)
C(9)-H(9)	0.9500
C(10)-C(13)	1.502(3)
C(11)-H(11A)	0.9800
C(11)-H(11B)	0.9800
C(11)-H(11C)	0.9800
C(12)-H(12A)	0.9800
C(12)-H(12B)	0.9800
C(12)-H(12C)	0.9800
C(13)-H(13A)	0.9800
C(13)-H(13B)	0.9800
C(13)-H(13C)	0.9800
C(14)-C(19)	1.400(3)
C(14)-C(15)	1.399(3)
C(15)-C(16)	1.390(3)
C(15)-C(20)	1.500(3)
C(16)-C(17)	1.384(3)
C(16)-H(16)	0.9500
C(17)-C(18)	1.381(4)
C(17)-C(21)	1.509(3)
C(18)-C(19)	1.391(3)
C(18)-H(18)	0.9500
C(19)-C(22)	1.506(4)
C(20)-H(20A)	0.9800
C(20)-H(20B)	0.9800
C(20)-H(20C)	0.9800
C(21)-H(21A)	0.9800
C(21)-H(21B)	0.9800
C(21)-H(21C)	0.9800
C(22)-H(22A)	0.9800
C(22)-H(22B)	0.9800
C(22)-H(22C)	0.9800

C(23)-C(28)	1.411(3)
C(23)-C(24)	1.414(3)
C(24)-C(25)	1.394(3)
C(24)-C(29)	1.513(3)
C(25)-C(26)	1.375(4)
C(25)-H(25)	0.9500
C(26)-C(27)	1.385(3)
C(26)-H(26)	0.9500
C(27)-C(28)	1.387(3)
C(27)-H(27)	0.9500
C(28)-C(32)	1.513(3)
C(29)-C(31)	1.527(4)
C(29)-C(30)	1.527(4)
C(29)-H(29)	1.000
C(30)-H(30A)	0.9800
C(30)-H(30B)	0.9800
C(30)-H(30C)	0.9800
C(31)-H(31A)	0.9800
C(31)-H(31B)	0.9800
C(31)-H(31C)	0.9800
C(32)-C(34)	1.525(3)
C(32)-C(33)	1.527(3)
C(32)-H(32)	1.000
C(33)-H(33A)	0.9800
C(33)-H(33B)	0.9800
C(33)-H(33C)	0.9800
C(34)-H(34A)	0.9800
C(34)-H(34B)	0.9800
C(34)-H(34C)	0.9800
C(35)-C(36)	1.515(3)
C(35)-H(35)	0.9500
C(36)-C(39)	1.533(3)
C(36)-C(38)	1.534(3)
C(36)-C(37)	1.535(3)
C(37)-H(37A)	0.9800
C(37)-H(37B)	0.9800
C(37)-H(37C)	0.9800
C(38)-H(38A)	0.9800
C(38)-H(38B)	0.9800
C(38)-H(38C)	0.9800
C(39)-C(44)	1.385(3)
C(39)-C(40)	1.391(3)
C(40)-C(41)	1.390(3)
C(40)-H(40)	0.9500

C(41)-C(42)	1.380(3)
C(41)-H(41)	0.9500
C(42)-C(43)	1.372(3)
C(42)-H(42)	0.9500
C(43)-C(44)	1.390(3)
C(43)-H(43)	0.9500
C(44)-H(44)	0.9500
C(45)-C(46)	1.490(3)
C(46)-C(48)	1.534(4)
C(46)-C(47)	1.534(3)
C(46)-C(49)	1.540(4)
C(47)-H(47A)	0.9800
C(47)-H(47B)	0.9800
C(47)-H(47C)	0.9800
C(48)-H(48A)	0.9800
C(48)-H(48B)	0.9800
C(48)-H(48C)	0.9800
C(49)-H(49A)	0.9800
C(49)-H(49B)	0.9800
C(49)-H(49C)	0.9800
B(1)-C(50)	1.635(3)
B(1)-C(74)	1.637(3)
B(1)-C(66)	1.641(3)
B(1)-C(58)	1.644(3)
C(50)-C(55)	1.399(3)
C(50)-C(51)	1.407(3)
C(51)-C(52)	1.385(3)
C(51)-H(51)	0.9500
C(52)-C(53)	1.389(3)
C(52)-C(56)	1.496(3)
C(53)-C(54)	1.382(3)
C(53)-H(53)	0.9500
C(54)-C(55)	1.393(3)
C(54)-C(57)	1.495(3)
C(55)-H(55)	0.9500
C(56)-F(56B)	1.317(3)
C(56)-F(56C)	1.327(3)
C(56)-F(56A)	1.339(3)
C(57)-F(57C)	1.304(3)
C(57)-F(57B)	1.304(3)
C(57)-F(57A)	1.325(3)
C(58)-C(63)	1.398(3)
C(58)-C(59)	1.400(3)
C(59)-C(60)	1.386(3)

C(59)-H(59)	0.9500
C(60)-C(61)	1.380(3)
C(60)-C(64)	1.495(3)
C(61)-C(62)	1.381(3)
C(61)-H(61)	0.9500
C(62)-C(63)	1.396(3)
C(62)-C(65)	1.497(3)
C(63)-H(63)	0.9500
C(64)-F(64C)	1.244(8)
C(64)-F(64E)	1.266(9)
C(64)-F(64A)	1.331(3)
C(64)-F(64B)	1.384(7)
C(64)-F(64F)	1.408(8)
C(65)-F(65C)	1.317(3)
C(65)-F(65A)	1.327(3)
C(65)-F(65B)	1.327(3)
C(66)-C(71)	1.397(3)
C(66)-C(67)	1.403(3)
C(67)-C(68)	1.389(3)
C(67)-H(67)	0.9500
C(68)-C(69)	1.388(3)
C(68)-C(72)	1.494(3)
C(69)-C(70)	1.381(3)
C(69)-H(69)	0.9500
C(70)-C(71)	1.392(3)
C(70)-C(73)	1.491(3)
C(71)-H(71)	0.9500
C(72)-F(72D)	1.270(5)
C(72)-F(72A)	1.273(6)
C(72)-F(72E)	1.309(8)
C(72)-F(72C)	1.320(5)
C(72)-F(72F)	1.329(5)
C(72)-F(72B)	1.350(6)
C(73)-F(73C)	1.338(2)
C(73)-F(73B)	1.341(2)
C(73)-F(73A)	1.344(3)
C(74)-C(79)	1.398(3)
C(74)-C(75)	1.403(3)
C(75)-C(76)	1.390(3)
C(75)-H(75)	0.9500
C(76)-C(77)	1.392(3)
C(76)-C(80)	1.489(3)
C(77)-C(78)	1.386(3)
C(77)-H(77)	0.9500

C(78)-C(79)	1.387(3)
C(78)-C(81)	1.503(3)
C(79)-H(79)	0.9500
F(80A)-C(80)	1.332(3)
C(80)-F(80C)	1.316(3)
C(80)-F(80B)	1.323(3)
C(81)-F(81B)	1.314(3)
C(81)-F(81A)	1.328(3)
C(81)-F(81C)	1.352(4)
C(1X)-C(2X)	1.489(5)
C(1X)-CL1X	1.798(3)
C(1X)-H(1X1)	0.9900
C(1X)-H(1X2)	0.9900
C(2X)-CL2X	1.779(4)
C(2X)-H(2X1)	0.9900
C(2X)-H(2X2)	0.9900
N(3)-W(1)-C(35)	101.70(8)
N(3)-W(1)-N(4)	96.97(7)
C(35)-W(1)-N(4)	90.19(7)
N(3)-W(1)-C(1)	136.38(7)
C(35)-W(1)-C(1)	121.63(8)
N(4)-W(1)-C(1)	79.41(6)
N(3)-W(1)-BR1	99.63(5)
C(35)-W(1)-BR1	94.77(6)
N(4)-W(1)-BR1	161.31(4)
C(1)-W(1)-BR1	82.66(5)
C(1)-N(1)-C(5)	120.09(16)
C(1)-N(1)-C(2)	124.14(17)
C(5)-N(1)-C(2)	115.43(17)
N(1)-C(1)-N(2)	118.29(18)
N(1)-C(1)-W(1)	124.94(13)
N(2)-C(1)-W(1)	116.72(14)
C(1)-N(2)-C(14)	119.08(17)
C(1)-N(2)-C(4)	124.04(18)
C(14)-N(2)-C(4)	116.61(17)
C(23)-N(3)-W(1)	174.99(14)
C(45)-N(4)-W(1)	172.47(16)
C(3A)-C(2)-N(1)	111.4(5)
N(1)-C(2)-C(3)	109.49(19)
N(1)-C(2)-H(2A)	110.0
C(3)-C(2)-H(2A)	110.1
N(1)-C(2)-H(2B)	109.9
C(3)-C(2)-H(2B)	109.9

H(2A)-C(2)-H(2B)	107.5
C(3A)-C(2)-H(2C)	109.2
N(1)-C(2)-H(2C)	109.3
C(3A)-C(2)-H(2D)	109.6
N(1)-C(2)-H(2D)	109.8
H(2C)-C(2)-H(2D)	107.3
C(4)-C(3)-C(2)	109.7(2)
C(4)-C(3)-H(3A)	109.7
C(2)-C(3)-H(3A)	109.7
C(4)-C(3)-H(3B)	109.7
C(2)-C(3)-H(3B)	109.7
H(3A)-C(3)-H(3B)	108.2
C(2)-C(3A)-C(4)	112.6(8)
C(2)-C(3A)-H(3C)	109.1
C(4)-C(3A)-H(3C)	109.1
C(2)-C(3A)-H(3D)	109.1
C(4)-C(3A)-H(3D)	109.1
H(3C)-C(3A)-H(3D)	107.8
C(3)-C(4)-N(2)	109.9(2)
N(2)-C(4)-C(3A)	110.5(5)
C(3)-C(4)-H(4A)	109.8
N(2)-C(4)-H(4A)	109.8
C(3)-C(4)-H(4B)	110.0
N(2)-C(4)-H(4B)	109.9
H(4A)-C(4)-H(4B)	107.4
N(2)-C(4)-H(4C)	110.1
C(3A)-C(4)-H(4C)	110.1
N(2)-C(4)-H(4D)	109.6
C(3A)-C(4)-H(4D)	109.1
H(4C)-C(4)-H(4D)	107.5
C(6)-C(5)-C(10)	121.18(19)
C(6)-C(5)-N(1)	120.04(18)
C(10)-C(5)-N(1)	118.71(18)
C(5)-C(6)-C(7)	118.1(2)
C(5)-C(6)-C(11)	122.52(19)
C(7)-C(6)-C(11)	119.4(2)
C(8)-C(7)-C(6)	121.8(2)
C(8)-C(7)-H(7)	119.1
C(6)-C(7)-H(7)	119.1
C(9)-C(8)-C(7)	118.4(2)
C(9)-C(8)-C(12)	120.8(2)
C(7)-C(8)-C(12)	120.7(2)
C(8)-C(9)-C(10)	122.1(2)
C(8)-C(9)-H(9)	119.0

C(10)-C(9)-H(9)	119.0
C(9)-C(10)-C(5)	117.9(2)
C(9)-C(10)-C(13)	119.6(2)
C(5)-C(10)-C(13)	122.41(19)
C(6)-C(11)-H(11A)	109.5
C(6)-C(11)-H(11B)	109.5
H(11A)-C(11)-H(11B)	109.5
C(6)-C(11)-H(11C)	109.5
H(11A)-C(11)-H(11C)	109.5
H(11B)-C(11)-H(11C)	109.5
C(8)-C(12)-H(12A)	109.5
C(8)-C(12)-H(12B)	109.5
H(12A)-C(12)-H(12B)	109.5
C(8)-C(12)-H(12C)	109.5
H(12A)-C(12)-H(12C)	109.5
H(12B)-C(12)-H(12C)	109.5
C(10)-C(13)-H(13A)	109.5
C(10)-C(13)-H(13B)	109.5
H(13A)-C(13)-H(13B)	109.5
C(10)-C(13)-H(13C)	109.5
H(13A)-C(13)-H(13C)	109.5
H(13B)-C(13)-H(13C)	109.5
C(19)-C(14)-C(15)	121.2(2)
C(19)-C(14)-N(2)	119.6(2)
C(15)-C(14)-N(2)	118.94(19)
C(16)-C(15)-C(14)	117.8(2)
C(16)-C(15)-C(20)	119.1(2)
C(14)-C(15)-C(20)	122.9(2)
C(17)-C(16)-C(15)	122.1(2)
C(17)-C(16)-H(16)	118.9
C(15)-C(16)-H(16)	118.9
C(18)-C(17)-C(16)	118.3(2)
C(18)-C(17)-C(21)	121.1(2)
C(16)-C(17)-C(21)	120.6(3)
C(17)-C(18)-C(19)	122.2(2)
C(17)-C(18)-H(18)	118.9
C(19)-C(18)-H(18)	118.9
C(18)-C(19)-C(14)	117.8(2)
C(18)-C(19)-C(22)	119.5(2)
C(14)-C(19)-C(22)	122.6(2)
C(15)-C(20)-H(20A)	109.5
C(15)-C(20)-H(20B)	109.5
H(20A)-C(20)-H(20B)	109.5
C(15)-C(20)-H(20C)	109.5

H(20A)-C(20)-H(20C)	109.5
H(20B)-C(20)-H(20C)	109.5
C(17)-C(21)-H(21A)	109.5
C(17)-C(21)-H(21B)	109.5
H(21A)-C(21)-H(21B)	109.5
C(17)-C(21)-H(21C)	109.5
H(21A)-C(21)-H(21C)	109.5
H(21B)-C(21)-H(21C)	109.5
C(19)-C(22)-H(22A)	109.5
C(19)-C(22)-H(22B)	109.5
H(22A)-C(22)-H(22B)	109.5
C(19)-C(22)-H(22C)	109.5
H(22A)-C(22)-H(22C)	109.5
H(22B)-C(22)-H(22C)	109.5
N(3)-C(23)-C(28)	120.12(17)
N(3)-C(23)-C(24)	119.34(18)
C(28)-C(23)-C(24)	120.48(18)
C(25)-C(24)-C(23)	118.1(2)
C(25)-C(24)-C(29)	119.2(2)
C(23)-C(24)-C(29)	122.61(19)
C(26)-C(25)-C(24)	121.7(2)
C(26)-C(25)-H(25)	119.1
C(24)-C(25)-H(25)	119.1
C(25)-C(26)-C(27)	119.6(2)
C(25)-C(26)-H(26)	120.2
C(27)-C(26)-H(26)	120.2
C(26)-C(27)-C(28)	121.4(2)
C(26)-C(27)-H(27)	119.3
C(28)-C(27)-H(27)	119.3
C(27)-C(28)-C(23)	118.59(19)
C(27)-C(28)-C(32)	120.61(19)
C(23)-C(28)-C(32)	120.60(18)
C(24)-C(29)-C(31)	111.9(2)
C(24)-C(29)-C(30)	110.0(2)
C(31)-C(29)-C(30)	111.3(2)
C(24)-C(29)-H(29)	107.8
C(31)-C(29)-H(29)	107.8
C(30)-C(29)-H(29)	107.8
C(29)-C(30)-H(30A)	109.5
C(29)-C(30)-H(30B)	109.5
H(30A)-C(30)-H(30B)	109.5
C(29)-C(30)-H(30C)	109.5
H(30A)-C(30)-H(30C)	109.5
H(30B)-C(30)-H(30C)	109.5

C(29)-C(31)-H(31A)	109.5
C(29)-C(31)-H(31B)	109.5
H(31A)-C(31)-H(31B)	109.5
C(29)-C(31)-H(31C)	109.5
H(31A)-C(31)-H(31C)	109.5
H(31B)-C(31)-H(31C)	109.5
C(28)-C(32)-C(34)	108.77(17)
C(28)-C(32)-C(33)	113.61(18)
C(34)-C(32)-C(33)	109.73(18)
C(28)-C(32)-H(32)	108.2
C(34)-C(32)-H(32)	108.2
C(33)-C(32)-H(32)	108.2
C(32)-C(33)-H(33A)	109.5
C(32)-C(33)-H(33B)	109.5
H(33A)-C(33)-H(33B)	109.5
C(32)-C(33)-H(33C)	109.5
H(33A)-C(33)-H(33C)	109.5
H(33B)-C(33)-H(33C)	109.5
C(32)-C(34)-H(34A)	109.5
C(32)-C(34)-H(34B)	109.5
H(34A)-C(34)-H(34B)	109.5
C(32)-C(34)-H(34C)	109.5
H(34A)-C(34)-H(34C)	109.5
H(34B)-C(34)-H(34C)	109.5
C(36)-C(35)-W(1)	145.88(15)
C(36)-C(35)-H(35)	107.1
W(1)-C(35)-H(35)	107.1
C(35)-C(36)-C(39)	108.28(16)
C(35)-C(36)-C(38)	106.51(17)
C(39)-C(36)-C(38)	112.24(16)
C(35)-C(36)-C(37)	111.59(16)
C(39)-C(36)-C(37)	108.39(17)
C(38)-C(36)-C(37)	109.85(19)
C(36)-C(37)-H(37A)	109.5
C(36)-C(37)-H(37B)	109.5
H(37A)-C(37)-H(37B)	109.5
C(36)-C(37)-H(37C)	109.5
H(37A)-C(37)-H(37C)	109.5
H(37B)-C(37)-H(37C)	109.5
C(36)-C(38)-H(38A)	109.5
C(36)-C(38)-H(38B)	109.5
H(38A)-C(38)-H(38B)	109.5
C(36)-C(38)-H(38C)	109.5
H(38A)-C(38)-H(38C)	109.5

H(38B)-C(38)-H(38C)	109.5
C(44)-C(39)-C(40)	117.85(18)
C(44)-C(39)-C(36)	123.11(18)
C(40)-C(39)-C(36)	119.02(17)
C(41)-C(40)-C(39)	121.1(2)
C(41)-C(40)-H(40)	119.4
C(39)-C(40)-H(40)	119.4
C(42)-C(41)-C(40)	120.2(2)
C(42)-C(41)-H(41)	119.9
C(40)-C(41)-H(41)	119.9
C(43)-C(42)-C(41)	119.2(2)
C(43)-C(42)-H(42)	120.4
C(41)-C(42)-H(42)	120.4
C(42)-C(43)-C(44)	120.72(19)
C(42)-C(43)-H(43)	119.6
C(44)-C(43)-H(43)	119.6
C(39)-C(44)-C(43)	120.9(2)
C(39)-C(44)-H(44)	119.5
C(43)-C(44)-H(44)	119.5
N(4)-C(45)-C(46)	178.3(2)
C(45)-C(46)-C(48)	109.3(2)
C(45)-C(46)-C(47)	109.19(19)
C(48)-C(46)-C(47)	109.9(2)
C(45)-C(46)-C(49)	107.5(2)
C(48)-C(46)-C(49)	110.4(2)
C(47)-C(46)-C(49)	110.5(2)
C(46)-C(47)-H(47A)	109.5
C(46)-C(47)-H(47B)	109.5
H(47A)-C(47)-H(47B)	109.5
C(46)-C(47)-H(47C)	109.5
H(47A)-C(47)-H(47C)	109.5
H(47B)-C(47)-H(47C)	109.5
C(46)-C(48)-H(48A)	109.5
C(46)-C(48)-H(48B)	109.5
H(48A)-C(48)-H(48B)	109.5
C(46)-C(48)-H(48C)	109.5
H(48A)-C(48)-H(48C)	109.5
H(48B)-C(48)-H(48C)	109.5
C(46)-C(49)-H(49A)	109.5
C(46)-C(49)-H(49B)	109.5
H(49A)-C(49)-H(49B)	109.5
C(46)-C(49)-H(49C)	109.5
H(49A)-C(49)-H(49C)	109.5
H(49B)-C(49)-H(49C)	109.5

C(50)-B(1)-C(74)	103.57(15)
C(50)-B(1)-C(66)	113.00(15)
C(74)-B(1)-C(66)	112.03(15)
C(50)-B(1)-C(58)	113.14(15)
C(74)-B(1)-C(58)	110.62(15)
C(66)-B(1)-C(58)	104.69(15)
C(55)-C(50)-C(51)	115.63(16)
C(55)-C(50)-B(1)	123.01(16)
C(51)-C(50)-B(1)	121.27(16)
C(52)-C(51)-C(50)	122.10(17)
C(52)-C(51)-H(51)	118.9
C(50)-C(51)-H(51)	118.9
C(51)-C(52)-C(53)	121.02(18)
C(51)-C(52)-C(56)	120.67(17)
C(53)-C(52)-C(56)	118.25(17)
C(54)-C(53)-C(52)	117.99(18)
C(54)-C(53)-H(53)	121.0
C(52)-C(53)-H(53)	121.0
C(53)-C(54)-C(55)	120.90(18)
C(53)-C(54)-C(57)	120.08(18)
C(55)-C(54)-C(57)	119.01(18)
C(54)-C(55)-C(50)	122.22(17)
C(54)-C(55)-H(55)	118.9
C(50)-C(55)-H(55)	118.9
F(56B)-C(56)-F(56C)	109.1(2)
F(56B)-C(56)-F(56A)	104.67(18)
F(56C)-C(56)-F(56A)	104.6(2)
F(56B)-C(56)-C(52)	112.61(18)
F(56C)-C(56)-C(52)	112.82(17)
F(56A)-C(56)-C(52)	112.43(18)
F(57C)-C(57)-F(57B)	108.2(2)
F(57C)-C(57)-F(57A)	102.5(3)
F(57B)-C(57)-F(57A)	105.5(2)
F(57C)-C(57)-C(54)	112.8(2)
F(57B)-C(57)-C(54)	114.29(19)
F(57A)-C(57)-C(54)	112.7(2)
C(63)-C(58)-C(59)	115.17(18)
C(63)-C(58)-B(1)	125.64(16)
C(59)-C(58)-B(1)	119.09(17)
C(60)-C(59)-C(58)	122.51(19)
C(60)-C(59)-H(59)	118.7
C(58)-C(59)-H(59)	118.7
C(61)-C(60)-C(59)	121.35(19)
C(61)-C(60)-C(64)	119.0(2)

C(59)-C(60)-C(64)	119.7(2)
C(62)-C(61)-C(60)	117.53(19)
C(62)-C(61)-H(61)	121.2
C(60)-C(61)-H(61)	121.2
C(61)-C(62)-C(63)	121.17(19)
C(61)-C(62)-C(65)	118.63(19)
C(63)-C(62)-C(65)	120.20(19)
C(62)-C(63)-C(58)	122.21(18)
C(62)-C(63)-H(63)	118.9
C(58)-C(63)-H(63)	118.9
F(64C)-C(64)-F(64A)	113.0(6)
F(64E)-C(64)-F(64A)	122.9(8)
F(64C)-C(64)-F(64B)	107.6(5)
F(64A)-C(64)-F(64B)	93.0(4)
F(64E)-C(64)-F(64F)	99.5(7)
F(64A)-C(64)-F(64F)	94.8(5)
F(64C)-C(64)-C(60)	117.4(4)
F(64E)-C(64)-C(60)	113.7(4)
F(64A)-C(64)-C(60)	112.8(2)
F(64B)-C(64)-C(60)	110.2(3)
F(64F)-C(64)-C(60)	109.0(4)
F(65C)-C(65)-F(65A)	106.8(2)
F(65C)-C(65)-F(65B)	106.1(2)
F(65A)-C(65)-F(65B)	104.5(2)
F(65C)-C(65)-C(62)	112.9(2)
F(65A)-C(65)-C(62)	112.59(19)
F(65B)-C(65)-C(62)	113.3(2)
C(71)-C(66)-C(67)	115.52(17)
C(71)-C(66)-B(1)	122.89(16)
C(67)-C(66)-B(1)	121.34(16)
C(68)-C(67)-C(66)	122.12(18)
C(68)-C(67)-H(67)	118.9
C(66)-C(67)-H(67)	118.9
C(69)-C(68)-C(67)	121.11(18)
C(69)-C(68)-C(72)	118.10(18)
C(67)-C(68)-C(72)	120.76(19)
C(70)-C(69)-C(68)	117.65(18)
C(70)-C(69)-H(69)	121.2
C(68)-C(69)-H(69)	121.2
C(69)-C(70)-C(71)	121.17(17)
C(69)-C(70)-C(73)	120.04(18)
C(71)-C(70)-C(73)	118.76(17)
C(70)-C(71)-C(66)	122.29(17)
C(70)-C(71)-H(71)	118.9

C(66)-C(71)-H(71)	118.9
F(72D)-C(72)-F(72E)	110.5(6)
F(72A)-C(72)-F(72C)	107.0(5)
F(72D)-C(72)-F(72F)	104.3(5)
F(72E)-C(72)-F(72F)	103.3(5)
F(72A)-C(72)-F(72B)	105.4(4)
F(72C)-C(72)-F(72B)	101.7(5)
F(72D)-C(72)-C(68)	114.3(3)
F(72A)-C(72)-C(68)	116.4(3)
F(72E)-C(72)-C(68)	112.7(4)
F(72C)-C(72)-C(68)	113.7(3)
F(72F)-C(72)-C(68)	110.9(3)
F(72B)-C(72)-C(68)	111.4(3)
F(73C)-C(73)-F(73B)	106.04(17)
F(73C)-C(73)-F(73A)	105.63(18)
F(73B)-C(73)-F(73A)	106.10(17)
F(73C)-C(73)-C(70)	113.48(18)
F(73B)-C(73)-C(70)	113.29(18)
F(73A)-C(73)-C(70)	111.68(16)
C(79)-C(74)-C(75)	115.77(18)
C(79)-C(74)-B(1)	122.09(17)
C(75)-C(74)-B(1)	121.81(16)
C(76)-C(75)-C(74)	122.49(18)
C(76)-C(75)-H(75)	118.8
C(74)-C(75)-H(75)	118.8
C(75)-C(76)-C(77)	120.4(2)
C(75)-C(76)-C(80)	119.70(19)
C(77)-C(76)-C(80)	119.87(19)
C(78)-C(77)-C(76)	118.02(19)
C(78)-C(77)-H(77)	121.0
C(76)-C(77)-H(77)	121.0
C(77)-C(78)-C(79)	121.20(19)
C(77)-C(78)-C(81)	121.4(2)
C(79)-C(78)-C(81)	117.4(2)
C(78)-C(79)-C(74)	122.1(2)
C(78)-C(79)-H(79)	119.0
C(74)-C(79)-H(79)	119.0
F(80C)-C(80)-F(80B)	108.8(2)
F(80C)-C(80)-F(80A)	104.6(2)
F(80B)-C(80)-F(80A)	103.4(2)
F(80C)-C(80)-C(76)	112.2(2)
F(80B)-C(80)-C(76)	113.8(2)
F(80A)-C(80)-C(76)	113.2(2)
F(81B)-C(81)-F(81A)	109.9(2)

F(81B)-C(81)-F(81C)	104.0(3)
F(81A)-C(81)-F(81C)	104.7(2)
F(81B)-C(81)-C(78)	112.4(2)
F(81A)-C(81)-C(78)	112.8(2)
F(81C)-C(81)-C(78)	112.5(2)
C(2X)-C(1X)-CL1X	109.8(2)
C(2X)-C(1X)-H(1X1)	109.7
CL1X-C(1X)-H(1X1)	109.7
C(2X)-C(1X)-H(1X2)	109.7
CL1X-C(1X)-H(1X2)	109.7
H(1X1)-C(1X)-H(1X2)	108.2
C(1X)-C(2X)-CL2X	108.0(2)
C(1X)-C(2X)-H(2X1)	110.1
CL2X-C(2X)-H(2X1)	110.1
C(1X)-C(2X)-H(2X2)	110.1
CL2X-C(2X)-H(2X2)	110.1
H(2X1)-C(2X)-H(2X2)	108.4

Table S 11: Anisotropic displacement parameters (Å^2 x 10^3) for W-14. The anisotropic displacement factor exponent takes the form: -2π^2 [h^2 a*^{2} U_{11} + ... + 2 h k a* b* U_{12}].

	U11	U22	U33	U23	U13	U12
W(1)	17(1)	13(1)	17(1)	-1(1)	-1(1)	-2(1)
BR1	27(1)	24(1)	19(1)	3(1)	-2(1)	-3(1)
N(1)	19(1)	22(1)	24(1)	-1(1)	-2(1)	-2(1)
C(1)	20(1)	20(1)	21(1)	1(1)	-4(1)	-2(1)
N(2)	21(1)	22(1)	39(1)	-7(1)	-6(1)	-4(1)
N(3)	21(1)	15(1)	19(1)	0(1)	-1(1)	-2(1)
N(4)	20(1)	19(1)	24(1)	0(1)	-1(1)	-3(1)
C(2)	19(1)	35(1)	41(1)	-3(1)	-4(1)	-2(1)
C(3)	22(1)	38(2)	46(2)	-11(1)	-1(1)	-10(1)
C(3A)	29(4)	44(5)	50(5)	-10(4)	-9(4)	-1(4)
C(4)	24(1)	39(1)	58(2)	-14(1)	-10(1)	-8(1)
C(5)	20(1)	20(1)	27(1)	-3(1)	1(1)	1(1)
C(6)	23(1)	25(1)	26(1)	-3(1)	2(1)	3(1)
C(7)	29(1)	31(1)	32(1)	-11(1)	2(1)	1(1)
C(8)	28(1)	25(1)	45(1)	-11(1)	4(1)	0(1)
C(9)	32(1)	22(1)	39(1)	1(1)	4(1)	0(1)
C(10)	23(1)	24(1)	30(1)	0(1)	1(1)	2(1)
C(11)	39(1)	32(1)	26(1)	-2(1)	2(1)	3(1)
C(12)	50(2)	31(1)	61(2)	-15(1)	5(1)	-9(1)
C(13)	34(1)	31(1)	30(1)	3(1)	-4(1)	3(1)
C(14)	23(1)	20(1)	35(1)	-7(1)	-8(1)	-5(1)
C(15)	32(1)	21(1)	32(1)	-7(1)	-11(1)	-4(1)

C(16)	36(1)	28(1)	33(1)	-10(1)	-6(1)	-5(1)
C(17)	33(1)	26(1)	44(1)	-15(1)	-12(1)	0(1)
C(18)	37(1)	18(1)	45(1)	-6(1)	-17(1)	0(1)
C(19)	31(1)	21(1)	36(1)	-4(1)	-11(1)	-8(1)
C(20)	45(1)	31(1)	36(1)	-4(1)	-18(1)	-2(1)
C(21)	42(1)	40(1)	59(2)	-23(1)	-11(1)	9(1)
C(22)	45(1)	28(1)	44(1)	0(1)	-5(1)	-15(1)
C(23)	21(1)	19(1)	24(1)	-2(1)	2(1)	-1(1)
C(24)	29(1)	25(1)	28(1)	1(1)	6(1)	1(1)
C(25)	35(1)	38(1)	40(1)	3(1)	15(1)	6(1)
C(26)	34(1)	37(1)	50(2)	2(1)	12(1)	15(1)
C(27)	34(1)	25(1)	40(1)	4(1)	4(1)	9(1)
C(28)	24(1)	19(1)	28(1)	0(1)	-1(1)	0(1)
C(29)	32(1)	27(1)	33(1)	5(1)	11(1)	1(1)
C(30)	54(2)	40(1)	33(1)	7(1)	2(1)	1(1)
C(31)	42(1)	34(1)	57(2)	4(1)	15(1)	-8(1)
C(32)	24(1)	18(1)	26(1)	4(1)	-1(1)	-1(1)
C(33)	39(1)	19(1)	43(1)	6(1)	2(1)	1(1)
C(34)	34(1)	31(1)	28(1)	1(1)	-5(1)	-5(1)
C(35)	22(1)	18(1)	17(1)	0(1)	0(1)	-3(1)
C(36)	29(1)	18(1)	19(1)	-2(1)	-3(1)	-6(1)
C(37)	26(1)	23(1)	39(1)	-2(1)	-7(1)	-4(1)
C(38)	54(2)	31(1)	20(1)	2(1)	-7(1)	-17(1)
C(39)	27(1)	19(1)	21(1)	-3(1)	-1(1)	-5(1)
C(40)	68(2)	21(1)	21(1)	-2(1)	-7(1)	-14(1)
C(41)	77(2)	22(1)	26(1)	3(1)	-7(1)	-11(1)
C(42)	41(1)	18(1)	35(1)	-3(1)	-3(1)	-6(1)
C(43)	33(1)	20(1)	32(1)	-10(1)	-7(1)	-3(1)
C(44)	31(1)	23(1)	24(1)	-5(1)	-6(1)	-4(1)
C(45)	24(1)	21(1)	28(1)	1(1)	3(1)	-1(1)
C(46)	42(1)	26(1)	27(1)	8(1)	10(1)	0(1)
C(47)	57(2)	24(1)	36(1)	9(1)	9(1)	-4(1)
C(48)	49(2)	54(2)	56(2)	23(1)	30(1)	8(1)
C(49)	79(2)	37(1)	23(1)	0(1)	1(1)	-2(1)
B(1)	16(1)	19(1)	20(1)	1(1)	-3(1)	-5(1)
C(50)	16(1)	16(1)	19(1)	-2(1)	-1(1)	-3(1)
C(51)	17(1)	19(1)	19(1)	0(1)	-2(1)	-2(1)
C(52)	18(1)	18(1)	25(1)	2(1)	0(1)	-3(1)
C(53)	16(1)	20(1)	31(1)	2(1)	-3(1)	-3(1)
C(54)	17(1)	16(1)	29(1)	0(1)	-4(1)	0(1)
C(55)	19(1)	15(1)	24(1)	0(1)	-2(1)	-3(1)
C(56)	22(1)	24(1)	34(1)	6(1)	-2(1)	-4(1)
F(56A)	57(1)	37(1)	51(1)	4(1)	26(1)	-12(1)
F(56B)	92(1)	28(1)	44(1)	-5(1)	13(1)	-30(1)

F(56C)	38(1)	63(1)	97(2)	54(1)	-23(1)	-17(1)
C(57)	23(1)	25(1)	55(2)	11(1)	-11(1)	-3(1)
F(57A)	48(1)	137(2)	74(1)	64(2)	-11(1)	11(1)
F(57B)	32(1)	50(1)	116(2)	36(1)	-38(1)	-15(1)
F(57C)	96(2)	33(1)	165(3)	-23(1)	-75(2)	32(1)
C(58)	16(1)	22(1)	19(1)	3(1)	-1(1)	-3(1)
C(59)	18(1)	28(1)	26(1)	2(1)	-3(1)	-6(1)
C(60)	16(1)	33(1)	29(1)	3(1)	-5(1)	-3(1)
C(61)	20(1)	30(1)	33(1)	4(1)	-3(1)	4(1)
C(62)	22(1)	22(1)	26(1)	2(1)	-1(1)	-1(1)
C(63)	17(1)	23(1)	22(1)	1(1)	-3(1)	-3(1)
C(64)	19(1)	50(2)	44(1)	-4(1)	-6(1)	-5(1)
F(64A)	23(1)	81(1)	97(2)	-32(1)	-2(1)	-2(1)
F(64B)	38(2)	121(6)	39(2)	15(3)	-25(1)	-18(3)
F(64C)	39(3)	48(3)	182(9)	-40(5)	-54(5)	4(2)
F(64E)	46(4)	216(13)	40(3)	15(5)	-19(2)	-60(6)
F(64F)	21(2)	51(3)	94(5)	20(3)	-14(3)	-19(2)
C(65)	32(1)	24(1)	35(1)	1(1)	-5(1)	1(1)
F(65A)	36(1)	32(1)	126(2)	-13(1)	-1(1)	-8(1)
F(65B)	63(1)	26(1)	83(1)	10(1)	-29(1)	2(1)
F(65C)	183(3)	43(1)	38(1)	-11(1)	13(1)	-40(1)
C(66)	17(1)	17(1)	21(1)	-2(1)	-1(1)	-1(1)
C(67)	19(1)	23(1)	23(1)	-2(1)	-1(1)	-5(1)
C(68)	19(1)	21(1)	27(1)	0(1)	2(1)	-5(1)
C(69)	23(1)	20(1)	21(1)	1(1)	3(1)	0(1)
C(70)	20(1)	18(1)	20(1)	-2(1)	-1(1)	1(1)
C(71)	19(1)	18(1)	21(1)	-2(1)	-2(1)	-2(1)
C(72)	30(1)	39(1)	33(1)	1(1)	2(1)	-16(1)
F(72A)	71(4)	119(6)	35(2)	-16(3)	10(2)	-75(4)
F(72B)	30(2)	99(5)	123(6)	-53(4)	34(3)	-27(2)
F(72C)	65(3)	72(4)	137(7)	63(5)	-37(4)	-48(3)
F(72D)	80(6)	130(7)	43(3)	-35(4)	38(3)	-81(5)
F(72E)	39(3)	104(6)	111(6)	53(5)	-40(4)	-43(3)
F(72F)	57(3)	33(3)	131(7)	-25(3)	12(4)	-27(2)
C(73)	30(1)	26(1)	21(1)	-2(1)	-1(1)	0(1)
F(73A)	25(1)	59(1)	28(1)	-4(1)	-7(1)	-6(1)
F(73B)	39(1)	46(1)	20(1)	6(1)	-2(1)	-3(1)
F(73C)	74(1)	33(1)	36(1)	-15(1)	-18(1)	6(1)
C(74)	17(1)	24(1)	20(1)	0(1)	-2(1)	-7(1)
C(75)	22(1)	26(1)	19(1)	-1(1)	-4(1)	-6(1)
C(76)	27(1)	26(1)	23(1)	-4(1)	-3(1)	-7(1)
C(77)	36(1)	34(1)	22(1)	-8(1)	-3(1)	-12(1)
C(78)	35(1)	33(1)	20(1)	1(1)	-6(1)	-13(1)
C(79)	27(1)	25(1)	23(1)	1(1)	-5(1)	-10(1)

F(80A)	98(2)	33(1)	48(1)	5(1)	-5(1)	6(1)
C(80)	40(1)	31(1)	35(1)	-10(1)	-8(1)	-2(1)
F(80B)	136(2)	35(1)	57(1)	-21(1)	-42(1)	8(1)
F(80C)	38(1)	44(1)	196(3)	-6(1)	8(1)	6(1)
C(81)	58(2)	46(1)	25(1)	0(1)	-11(1)	-17(1)
F(81A)	67(1)	58(1)	24(1)	-7(1)	-15(1)	-9(1)
F(81B)	111(2)	75(1)	35(1)	22(1)	-23(1)	-51(1)
F(81C)	74(1)	73(1)	45(1)	-2(1)	-24(1)	18(1)
C(1X)	45(2)	45(2)	47(2)	12(1)	19(1)	12(1)
C(2X)	45(2)	57(2)	66(2)	18(2)	16(2)	16(2)
CL1X	75(1)	61(1)	40(1)	-2(1)	13(1)	20(1)
CL2X	57(1)	56(1)	117(1)	0(1)	12(1)	-15(1)

Table S 12: Hydrogen coordinates (x 10^4) and isotropic displacement parameters (Å^2 x 10^3) for W-14.

	x	y	z	U(eq)
H(2A)	1583	8430	6107	37
H(2B)	1439	8377	6934	37
H(2C)	1462	8661	6448	37
H(2D)	1554	7932	6982	37
H(3A)	1798	7024	7017	41
H(3B)	933	7215	6426	41
H(3C)	1779	7974	5516	48
H(3D)	924	7541	6014	48
H(4A)	2338	6370	6036	47
H(4B)	2202	7123	5532	47
H(4C)	2148	6561	6375	47
H(4D)	2370	6689	5557	47
H(7)	3649	9965	8169	36
H(9)	3720	10730	6098	38
H(11A)	2480	8683	8385	49
H(11B)	3718	8466	8415	49
H(11C)	3028	8033	7902	49
H(12A)	4303	11542	6921	70
H(12B)	4684	11150	7672	70
H(12C)	3500	11513	7598	70
H(13A)	2272	9721	5452	48
H(13B)	3126	8998	5398	48
H(13C)	3454	9863	5184	48
H(16)	5811	6299	4445	38
H(18)	5705	4964	6283	39
H(20A)	4769	7579	4362	55
H(20B)	3782	7702	4906	55

H(20C)	3771	7081	4319	55
H(21A)	7494	5201	5268	70
H(21B)	6909	5132	4545	70
H(21C)	6737	4514	5210	70
H(22A)	3573	5286	6895	58
H(22B)	3747	6114	7180	58
H(22C)	4617	5405	7300	58
H(25)	9622	6732	5139	47
H(26)	10004	5555	5778	50
H(27)	8908	5161	6739	41
H(29)	7493	8128	5510	38
H(30A)	8217	7345	4276	65
H(30B)	7542	8165	4250	65
H(30C)	7031	7398	4591	65
H(31A)	9287	8352	5658	67
H(31B)	8841	8806	4950	67
H(31C)	9584	8023	4897	67
H(32)	6418	6008	7216	28
H(33A)	7141	4694	7111	52
H(33B)	6750	4858	7909	52
H(33C)	7977	4829	7689	52
H(34A)	8226	6181	8017	46
H(34B)	7062	6143	8363	46
H(34C)	7342	6858	7815	46
H(35)	5402	8977	7196	23
H(37A)	7990	7983	7287	43
H(37B)	8546	8762	7398	43
H(37C)	8030	8655	6656	43
H(38A)	6124	8977	8442	52
H(38B)	7373	8889	8504	52
H(38C)	6755	8152	8350	52
H(40)	6605	9747	6229	43
H(41)	6592	11077	5908	49
H(42)	6923	11940	6752	37
H(43)	7223	11467	7919	34
H(44)	7196	10143	8250	31
H(47A)	4364	5477	8582	60
H(47B)	4768	5462	9375	60
H(47C)	5579	5562	8707	60
H(48A)	3377	7346	9118	84
H(48B)	3327	6519	9557	84
H(48C)	3038	6614	8735	84
H(49A)	6112	6785	9226	70
H(49B)	5246	6704	9864	70

H(49C)	5247	7486	9351	70
H(51)	2147	8450	349	22
H(53)	5128	8002	995	27
H(55)	2855	6598	1725	23
H(59)	-988	7512	702	29
H(61)	-1808	9810	794	33
H(63)	1117	8929	1305	25
H(67)	-531	6321	1500	26
H(69)	-385	6010	3650	26
H(71)	1710	7292	2501	23
H(75)	1530	5736	942	27
H(77)	1037	5514	-1141	36
H(79)	457	7602	-305	29
H(1X1)	8786	9701	2912	57
H(1X2)	8602	9197	3652	57
H(2X1)	10587	9366	2922	71
H(2X2)	10433	8787	3622	71

Table S 13: Torsion angles [°] for W-14.

C(5)-N(1)-C(1)-N(2)	-169.83(18)
C(2)-N(1)-C(1)-N(2)	3.1(3)
C(5)-N(1)-C(1)-W(1)	12.9(3)
C(2)-N(1)-C(1)-W(1)	-174.13(15)
N(1)-C(1)-N(2)-C(14)	173.43(18)
W(1)-C(1)-N(2)-C(14)	-9.1(2)
N(1)-C(1)-N(2)-C(4)	-0.3(3)
W(1)-C(1)-N(2)-C(4)	177.17(18)
C(1)-N(1)-C(2)-C(3A)	-28.5(6)
C(5)-N(1)-C(2)-C(3A)	144.8(6)
C(1)-N(1)-C(2)-C(3)	22.9(3)
C(5)-N(1)-C(2)-C(3)	-163.9(2)
N(1)-C(2)-C(3)-C(4)	-50.8(3)
N(1)-C(2)-C(3A)-C(4)	48.2(9)
C(2)-C(3)-C(4)-N(2)	53.5(3)
C(1)-N(2)-C(4)-C(3)	-29.6(3)
C(14)-N(2)-C(4)-C(3)	156.5(2)
C(1)-N(2)-C(4)-C(3A)	21.1(6)
C(14)-N(2)-C(4)-C(3A)	-152.8(6)
C(2)-C(3A)-C(4)-N(2)	-45.3(10)
C(1)-N(1)-C(5)-C(6)	-94.4(2)
C(2)-N(1)-C(5)-C(6)	92.0(2)
C(1)-N(1)-C(5)-C(10)	88.7(2)

C(2)-N(1)-C(5)-C(10)	-84.9(2)
C(10)-C(5)-C(6)-C(7)	-5.6(3)
N(1)-C(5)-C(6)-C(7)	177.65(18)
C(10)-C(5)-C(6)-C(11)	174.1(2)
N(1)-C(5)-C(6)-C(11)	-2.7(3)
C(5)-C(6)-C(7)-C(8)	-0.2(3)
C(11)-C(6)-C(7)-C(8)	-179.8(2)
C(6)-C(7)-C(8)-C(9)	3.5(3)
C(6)-C(7)-C(8)-C(12)	-178.3(2)
C(7)-C(8)-C(9)-C(10)	-1.2(3)
C(12)-C(8)-C(9)-C(10)	-179.4(2)
C(8)-C(9)-C(10)-C(5)	-4.3(3)
C(8)-C(9)-C(10)-C(13)	172.4(2)
C(6)-C(5)-C(10)-C(9)	7.7(3)
N(1)-C(5)-C(10)-C(9)	-175.43(19)
C(6)-C(5)-C(10)-C(13)	-168.9(2)
N(1)-C(5)-C(10)-C(13)	8.0(3)
C(1)-N(2)-C(14)-C(19)	99.1(2)
C(4)-N(2)-C(14)-C(19)	-86.7(3)
C(1)-N(2)-C(14)-C(15)	-86.3(2)
C(4)-N(2)-C(14)-C(15)	87.9(3)
C(19)-C(14)-C(15)-C(16)	-8.2(3)
N(2)-C(14)-C(15)-C(16)	177.38(18)
C(19)-C(14)-C(15)-C(20)	167.16(19)
N(2)-C(14)-C(15)-C(20)	-7.3(3)
C(14)-C(15)-C(16)-C(17)	2.4(3)
C(20)-C(15)-C(16)-C(17)	-173.1(2)
C(15)-C(16)-C(17)-C(18)	3.6(3)
C(15)-C(16)-C(17)-C(21)	-177.9(2)
C(16)-C(17)-C(18)-C(19)	-3.9(3)
C(21)-C(17)-C(18)-C(19)	177.5(2)
C(17)-C(18)-C(19)-C(14)	-1.6(3)
C(17)-C(18)-C(19)-C(22)	174.6(2)
C(15)-C(14)-C(19)-C(18)	7.8(3)
N(2)-C(14)-C(19)-C(18)	-177.77(18)
C(15)-C(14)-C(19)-C(22)	-168.3(2)
N(2)-C(14)-C(19)-C(22)	6.1(3)
N(3)-C(23)-C(24)-C(25)	180.0(2)
C(28)-C(23)-C(24)-C(25)	2.7(3)
N(3)-C(23)-C(24)-C(29)	-1.5(3)
C(28)-C(23)-C(24)-C(29)	-178.8(2)
C(23)-C(24)-C(25)-C(26)	-1.6(4)
C(29)-C(24)-C(25)-C(26)	179.8(3)
C(24)-C(25)-C(26)-C(27)	-0.4(5)

C(25)-C(26)-C(27)-C(28)	1.3(4)
C(26)-C(27)-C(28)-C(23)	-0.2(4)
C(26)-C(27)-C(28)-C(32)	-175.2(2)
N(3)-C(23)-C(28)-C(27)	-179.1(2)
C(24)-C(23)-C(28)-C(27)	-1.8(3)
N(3)-C(23)-C(28)-C(32)	-4.1(3)
C(24)-C(23)-C(28)-C(32)	173.2(2)
C(25)-C(24)-C(29)-C(31)	-50.2(3)
C(23)-C(24)-C(29)-C(31)	131.3(2)
C(25)-C(24)-C(29)-C(30)	74.0(3)
C(23)-C(24)-C(29)-C(30)	-104.5(3)
C(27)-C(28)-C(32)-C(34)	96.5(2)
C(23)-C(28)-C(32)-C(34)	-78.4(2)
C(27)-C(28)-C(32)-C(33)	-26.0(3)
C(23)-C(28)-C(32)-C(33)	159.1(2)
N(3)-W(1)-C(35)-C(36)	-4.0(3)
N(4)-W(1)-C(35)-C(36)	93.2(2)
C(1)-W(1)-C(35)-C(36)	170.8(2)
BR1-W(1)-C(35)-C(36)	-104.9(2)
W(1)-C(35)-C(36)-C(39)	136.6(2)
W(1)-C(35)-C(36)-C(38)	-102.5(3)
W(1)-C(35)-C(36)-C(37)	17.4(3)
C(35)-C(36)-C(39)-C(44)	136.5(2)
C(38)-C(36)-C(39)-C(44)	19.2(3)
C(37)-C(36)-C(39)-C(44)	-102.3(2)
C(35)-C(36)-C(39)-C(40)	-45.3(3)
C(38)-C(36)-C(39)-C(40)	-162.5(2)
C(37)-C(36)-C(39)-C(40)	76.0(3)
C(44)-C(39)-C(40)-C(41)	-0.4(4)
C(36)-C(39)-C(40)-C(41)	-178.8(2)
C(39)-C(40)-C(41)-C(42)	1.0(5)
C(40)-C(41)-C(42)-C(43)	-0.8(4)
C(41)-C(42)-C(43)-C(44)	0.0(4)
C(40)-C(39)-C(44)-C(43)	-0.4(3)
C(36)-C(39)-C(44)-C(43)	177.9(2)
C(42)-C(43)-C(44)-C(39)	0.7(4)
C(74)-B(1)-C(50)-C(55)	-90.2(2)
C(66)-B(1)-C(50)-C(55)	31.2(2)
C(58)-B(1)-C(50)-C(55)	149.94(17)
C(74)-B(1)-C(50)-C(51)	86.2(2)
C(66)-B(1)-C(50)-C(51)	-152.41(17)
C(58)-B(1)-C(50)-C(51)	-33.7(2)
C(55)-C(50)-C(51)-C(52)	-3.7(3)
B(1)-C(50)-C(51)-C(52)	179.63(17)

C(50)-C(51)-C(52)-C(53)	1.0(3)
C(50)-C(51)-C(52)-C(56)	-176.11(18)
C(51)-C(52)-C(53)-C(54)	2.1(3)
C(56)-C(52)-C(53)-C(54)	179.26(19)
C(52)-C(53)-C(54)-C(55)	-2.3(3)
C(52)-C(53)-C(54)-C(57)	178.6(2)
C(53)-C(54)-C(55)-C(50)	-0.6(3)
C(57)-C(54)-C(55)-C(50)	178.47(19)
C(51)-C(50)-C(55)-C(54)	3.5(3)
B(1)-C(50)-C(55)-C(54)	-179.86(18)
C(51)-C(52)-C(56)-F(56B)	113.6(2)
C(53)-C(52)-C(56)-F(56B)	-63.6(3)
C(51)-C(52)-C(56)-F(56C)	-10.5(3)
C(53)-C(52)-C(56)-F(56C)	172.3(2)
C(51)-C(52)-C(56)-F(56A)	-128.5(2)
C(53)-C(52)-C(56)-F(56A)	54.4(3)
C(53)-C(54)-C(57)-F(57C)	-111.1(3)
C(55)-C(54)-C(57)-F(57C)	69.8(3)
C(53)-C(54)-C(57)-F(57B)	13.0(3)
C(55)-C(54)-C(57)-F(57B)	-166.1(2)
C(53)-C(54)-C(57)-F(57A)	133.4(2)
C(55)-C(54)-C(57)-F(57A)	-45.7(3)
C(50)-B(1)-C(58)-C(63)	-23.4(3)
C(74)-B(1)-C(58)-C(63)	-139.12(19)
C(66)-B(1)-C(58)-C(63)	100.0(2)
C(50)-B(1)-C(58)-C(59)	160.42(17)
C(74)-B(1)-C(58)-C(59)	44.7(2)
C(66)-B(1)-C(58)-C(59)	-76.1(2)
C(63)-C(58)-C(59)-C(60)	0.8(3)
B(1)-C(58)-C(59)-C(60)	177.34(18)
C(58)-C(59)-C(60)-C(61)	1.3(3)
C(58)-C(59)-C(60)-C(64)	179.2(2)
C(59)-C(60)-C(61)-C(62)	-1.7(3)
C(64)-C(60)-C(61)-C(62)	-179.6(2)
C(60)-C(61)-C(62)-C(63)	-0.1(3)
C(60)-C(61)-C(62)-C(65)	-179.4(2)
C(61)-C(62)-C(63)-C(58)	2.3(3)
C(65)-C(62)-C(63)-C(58)	-178.40(19)
C(59)-C(58)-C(63)-C(62)	-2.6(3)
B(1)-C(58)-C(63)-C(62)	-178.84(18)
C(61)-C(60)-C(64)-F(64C)	-174.4(9)
C(59)-C(60)-C(64)-F(64C)	7.6(10)
C(61)-C(60)-C(64)-F(64E)	105.7(12)
C(59)-C(60)-C(64)-F(64E)	-72.2(12)

C(61)-C(60)-C(64)-F(64A)	-40.3(3)
C(59)-C(60)-C(64)-F(64A)	141.7(2)
C(61)-C(60)-C(64)-F(64B)	62.0(5)
C(59)-C(60)-C(64)-F(64B)	-115.9(5)
C(61)-C(60)-C(64)-F(64F)	-144.3(5)
C(59)-C(60)-C(64)-F(64F)	37.8(6)
C(61)-C(62)-C(65)-F(65C)	92.7(3)
C(63)-C(62)-C(65)-F(65C)	-86.7(3)
C(61)-C(62)-C(65)-F(65A)	-146.3(2)
C(63)-C(62)-C(65)-F(65A)	34.4(3)
C(61)-C(62)-C(65)-F(65B)	-28.0(3)
C(63)-C(62)-C(65)-F(65B)	152.6(2)
C(50)-B(1)-C(66)-C(71)	28.5(2)
C(74)-B(1)-C(66)-C(71)	144.98(17)
C(58)-B(1)-C(66)-C(71)	-95.1(2)
C(50)-B(1)-C(66)-C(67)	-157.64(17)
C(74)-B(1)-C(66)-C(67)	-41.1(2)
C(58)-B(1)-C(66)-C(67)	78.8(2)
C(71)-C(66)-C(67)-C(68)	2.0(3)
B(1)-C(66)-C(67)-C(68)	-172.33(18)
C(66)-C(67)-C(68)-C(69)	-3.7(3)
C(66)-C(67)-C(68)-C(72)	178.2(2)
C(67)-C(68)-C(69)-C(70)	1.7(3)
C(72)-C(68)-C(69)-C(70)	179.87(19)
C(68)-C(69)-C(70)-C(71)	1.8(3)
C(68)-C(69)-C(70)-C(73)	-176.31(18)
C(69)-C(70)-C(71)-C(66)	-3.5(3)
C(73)-C(70)-C(71)-C(66)	174.65(18)
C(67)-C(66)-C(71)-C(70)	1.5(3)
B(1)-C(66)-C(71)-C(70)	175.74(17)
C(69)-C(68)-C(72)-F(72D)	22.2(8)
C(67)-C(68)-C(72)-F(72D)	-159.6(8)
C(69)-C(68)-C(72)-F(72A)	-175.0(6)
C(67)-C(68)-C(72)-F(72A)	3.2(6)
C(69)-C(68)-C(72)-F(72E)	149.4(7)
C(67)-C(68)-C(72)-F(72E)	-32.4(8)
C(69)-C(68)-C(72)-F(72C)	-49.9(7)
C(67)-C(68)-C(72)-F(72C)	128.3(7)
C(69)-C(68)-C(72)-F(72F)	-95.4(6)
C(67)-C(68)-C(72)-F(72F)	82.8(6)
C(69)-C(68)-C(72)-F(72B)	64.3(6)
C(67)-C(68)-C(72)-F(72B)	-117.5(6)
C(69)-C(70)-C(73)-F(73C)	-106.0(2)
C(71)-C(70)-C(73)-F(73C)	75.9(2)

C(69)-C(70)-C(73)-F(73B)	15.0(3)
C(71)-C(70)-C(73)-F(73B)	-163.14(17)
C(69)-C(70)-C(73)-F(73A)	134.74(19)
C(71)-C(70)-C(73)-F(73A)	-43.4(2)
C(50)-B(1)-C(74)-C(79)	-94.2(2)
C(66)-B(1)-C(74)-C(79)	143.67(18)
C(58)-B(1)-C(74)-C(79)	27.3(2)
C(50)-B(1)-C(74)-C(75)	78.9(2)
C(66)-B(1)-C(74)-C(75)	-43.2(2)
C(58)-B(1)-C(74)-C(75)	-159.55(17)
C(79)-C(74)-C(75)-C(76)	-0.2(3)
B(1)-C(74)-C(75)-C(76)	-173.73(18)
C(74)-C(75)-C(76)-C(77)	-1.5(3)
C(74)-C(75)-C(76)-C(80)	176.0(2)
C(75)-C(76)-C(77)-C(78)	1.4(3)
C(80)-C(76)-C(77)-C(78)	-176.2(2)
C(76)-C(77)-C(78)-C(79)	0.4(3)
C(76)-C(77)-C(78)-C(81)	180.0(2)
C(77)-C(78)-C(79)-C(74)	-2.2(3)
C(81)-C(78)-C(79)-C(74)	178.3(2)
C(75)-C(74)-C(79)-C(78)	2.0(3)
B(1)-C(74)-C(79)-C(78)	175.54(19)
C(75)-C(76)-C(80)-F(80C)	-77.8(3)
C(77)-C(76)-C(80)-F(80C)	99.8(3)
C(75)-C(76)-C(80)-F(80B)	158.1(2)
C(77)-C(76)-C(80)-F(80B)	-24.3(3)
C(75)-C(76)-C(80)-F(80A)	40.3(3)
C(77)-C(76)-C(80)-F(80A)	-142.1(2)
C(77)-C(78)-C(81)-F(81B)	-116.8(3)
C(79)-C(78)-C(81)-F(81B)	62.8(3)
C(77)-C(78)-C(81)-F(81A)	8.1(4)
C(79)-C(78)-C(81)-F(81A)	-172.3(2)
C(77)-C(78)-C(81)-F(81C)	126.3(3)
C(79)-C(78)-C(81)-F(81C)	-54.1(3)
CL1X-C(1X)-C(2X)-CL2X	-174.45(15)

9. References

[1] Z. Yang, C.-H. Yang, S. Chen, X. Chen, L. Zhang, H. Ren, *Chem. Commun.* **2017**, *53*, 12092-12095.
[2] J. L. M. Matos, S. Vásquez-Céspedes, J. Gu, T. Oguma, R. A. Shenvi, *J. Am. Chem. Soc.* **2018**, *140*, 16976-16981.
[3] R. O'Dell, D. H. McConville, G. E. Hofmeister, R. R. Schrock, *J. Am. Chem. Soc.* **1994**, *116*, 3414-3423.
[4] A. J. Arduengo, H. V. R. Dias, R. L. Harlow, M. Kline, *Journal of the American Chemical Society* **1992**, *114*, 5530-5534.
[5] A. J. Arduengo, R. Krafczyk, R. Schmutzler, H. A. Craig, J. R. Goerlich, W. J. Marshall, M. Unverzagt, *Tetrahedron* **1999**, *55*, 14523-14534.
[6] T. Schaub, M. Backes, U. Radius, *Organometallics* **2006**, *25*, 4196-4206.
[7] W. Chen, F. Liu, *Journal of Organometallic Chemistry* **2003**, *673*, 5-12.
[8] D. M. Khramov, V. M. Lynch, C. W. Bielawski, *Organometallics* **2007**, *26*, 6042-6049.
[9] R. R. Schrock, R. T. DePue, J. Feldman, K. B. Yap, D. C. Yang, W. M. Davis, L. Park, M. DiMare, M. Schofield, *Organometallics* **1990**, *9*, 2262-2275.
[10] V. Mougel, C. B. Santiago, P. A. Zhizhko, E. N. Bess, J. Varga, G. Frater, M. S. Sigman, C. Coperet, *J. Am. Chem. Soc.* **2015**, *137*, 6699-6704.
[11] R. R. Schrock, A. J. Jiang, S. C. Marinescu, J. H. Simpson, P. Müller, *Organometallics* **2010**, *29*, 5241-5251.
[12] J. V. Musso, M. J. Benedikter, P. Gebel, I. Elser, W. Frey, M. R. Buchmeiser, *Organometallics* **2020**.
[13] K. Herz, M. Podewitz, L. Stohr, D. Wang, W. Frey, K. R. Liedl, S. Sen, M. R. Buchmeiser, *J. Am. Chem. Soc.* **2019**, *141*, 8264-8276.
[14] M. Pucino, M. Inoue, C. P. Gordon, R. Schowner, L. Stohr, S. Sen, C. Hegedus, E. Robe, F. Toth, M. R. Buchmeiser, C. Coperet, *Angew. Chem. Int. Ed.* **2018**, *57*, 14566-14569.
[15] M. J. Benedikter, R. Schowner, I. Elser, P. Werner, K. Herz, L. Stöhr, D. A. Imbrich, G. M. Nagy, D. Wang, M. R. Buchmeiser, *Macromolecules* **2019**, *52*, 4059-4066.
[16] D. L. Reger, T. D. Wright, C. A. Little, J. J. S. Lamba, M. D. Smith, *Inorg. Chem.* **2001**, *40*, 3810-3814.
[17] Y. Zhang, A. M. Santos, E. Herdtweck, J. Mink, F. E. Kühn, *New Journal of Chemistry* **2005**, *29*, 366-370.
[18] V. C. Gibson, T. P. Kee, A. Shaw, *Polyhedron* **1988**, *7*, 579-580.
[19] L. P. H. Lopez, R. R. Schrock, *J. Am. Chem. Soc.* **2004**, *126*, 9526-9527.
